# Unexpected Reactivity of Nitrones: Catalytic Insertion of CS_2_

**DOI:** 10.1021/acs.orglett.5c02611

**Published:** 2025-07-24

**Authors:** Marcos López-Aguilar, Nicolás Ríos-Lombardía, Daniel Barrena-Espés, Miguel Gallegos, Joaquín García-Álvarez, Carmen Concellón, Vicente del Amo

**Affiliations:** Physical and Analytical Chemistry Department, 16763 Universidad de Oviedo, Avenida Julián Clavería 8, 33006 Oviedo, Asturias, Spain

## Abstract

A new reactivity mode for nitrones is reported, consisting of the chloride-catalyzed insertion of CS_2_ to afford thioamides. This reaction proceeds under metal-free and mild conditions, tolerates a broad family of substrates, and is robust and easily scalable, being successfully applied to the synthesis of the HIV-1 reverse transcriptase inhibitor UC-781. Mechanistic studies, supported by DFT calculations, have revealed the role of chloride anions in the activation of CS_2_ and its subsequent insertion into nitrones.

## Graphical abstract

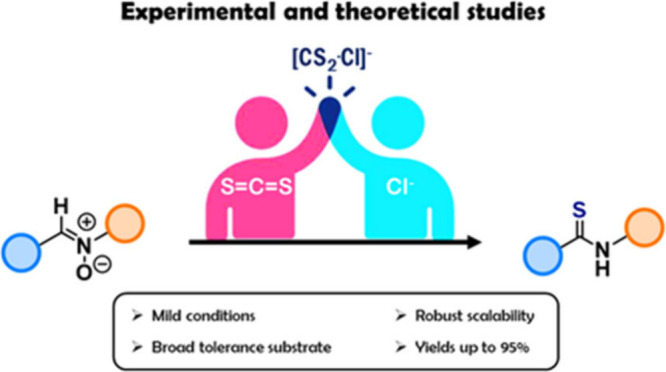

Thioamides are found in a variety of essential biomolecules (such as peptides, protein backbones, and DNA),^1^ as well as in natural products and pharmaceuticals.^2^ Beyond their biological relevance, thioamide-based chemical derivatives have attracted significant attention in synthetic organic chemistry due to their versatile applications. For example, they are used in the preparation of sulfur-based heterocycles, such as thiazoles and thiazolines, through reactions with dielectrophilic agents.^3^ Additionally, thioamides exhibit enhanced lipophilicity compared to their oxygen-based counterparts, the amides.^4^ They are also valuable in radical copolymerization processes, among other uses.^5^

The traditional synthesis of thioamides is well-documented in the literature.^6–10^ Conventional methods typically involve the use of thionating species. These include elemental sulfur (Willgerodt-Kindler reaction),^6^ sulfur-containing inorganic salts such as sodium sulfide, sodium disulfide, or calcium thioacetate,^7^ hydrogen sulfide through Thio-Ritter-type reactions,^8^ and phosphorus-based thionating agents like Lawesson’s reagent^9^ or phosphorus decasulfide (P_4_S_10_).^10^ These reagents are employed to convert a wide array of organic substrates into the corresponding thioamides.^6–10^ However, classical approaches have several limitations. They usually require harsh reaction conditions such as high temperatures. Additionally, poor chemoselectivity is observed when substrates contain multiple functional groups, often leading to undesired polythionations. Moreover, an excess of thionating agents is typically needed, which generates harmful and difficult-to-separate byproducts.

To tackle these limitations several methodologies were recently developed, offering an improved selectivity and a reduced environmental impact.^11^ Takemoto and co-workers reported a mild and chemoselective thioacylation of amines enabled by the nucleophilic activation of S_8_ (Scheme 1a).^11a^ Alternatively, Wu et al. designed a visible-light-driven synthesis of thioamides based on a multicomponent reaction involving CS_2_, amines, and olefins in the presence of stoichiometric amounts of DBU and different phosphines (PR_3_; Scheme 1b).^11b^

**Scheme 1:**
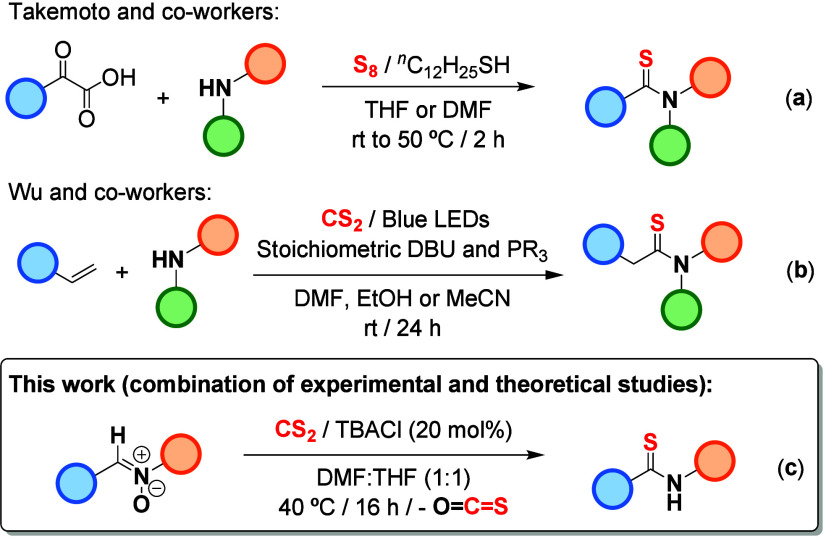
State of the Art for the Synthesis of Thioamides

Taking into account these precedents, and considering our previous knowledge on the activation of CS_2_ by Cl^–^ anions,^12a^ we decided to explore its potential in uncharted transformations. Herein we present the design and implementation of a strategy for the synthesis of thioamides using nitrones as substrates (Scheme 1c). To our knowledge, we disclose the first documented reactivity between nitrones and CS_2_.^13^ Moreover, as nitrones can be easily assembled from aldehydes and *N*-hydroxylamines (see Supporting Information for details), our approach allows a tailor-made design of the thioamide substituents by selecting the appropriate aldehyde and hydroxylamine precursors.

In a previous work^12a^ we proved the efficiency of chloride anions [coming from TBACl or choline chloride (*ChCl*)] as catalysts for the insertion of CS_2_ into epoxides. DFT studies of the reaction energy profiles revealed the transitory formation of a short-lived and highly reactive [CS_2_Cl]^−^ adduct, resulting from the nucleophilic attack of a chloride anion to the electrophilic carbon of CS_2_. It disrupts the linear geometry of the heterocumulene giving rise to an anionic near-trigonal planar synthon that exhibits a distinguished reactivity. By this means, stable di- or trithiocarbonates were rendered on demand and in high yield. Driven by our previous results,^12a^ we decided to extend this methodology to the synthesis of other five-membered sulfur-containing heterocyclic compounds (such as **2**, in Scheme 2), by choosing alternative partners for CS_2_. Notably, the reactivity between nitrones and CS_2_ had never been explored before. To our delight, upon optimization of the experimental reaction parameters (see Section III in Supporting Information for details), we found that *N*-(4-chlorobenzylidene)­aniline oxide (**1a**) and CS_2_ (1.2 equiv.) react in the presence of TBACl (20 mol %) to afford thioamide **3a** in 74% isolated yield (Scheme 2).^14^ A control experiment without chloride salt resulted in the decomposition of **1a**.

**Scheme 2:**
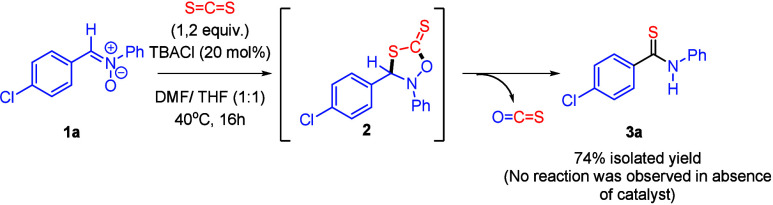
Synthesis of 4-Chloro-*N*-phenylbenzothioamide (3a) from CS_2_ and Nitrone 1a, Catalyzed by TBACl^a^

a General conditions: *N*-(4-chlorobenzylidene)­aniline oxide (**1a**, 93 mg, 0.4 mmol) and TBACl (20 mol%) were added into a 10 mL sealed tube and dissolved in 2 mL of dry DMF/THF (1:1) before CS_2_ (37 mg, 0.48 mmol) was added. The mixture was stirred at 40 °C (oil bath) for 16 h under argon atmosphere.

Although heterocycle **2** could not be characterized, we believe that it acts as an intermediate in the transformation **1a**→**3a**, evolving into the final product by an extrusion of CSO. To corroborate this hypothesis, mechanistic studies and Quantum Theory of Atoms in Molecules (QTAIM) analyses were performed at the DFT level of theory. First, to further clarify the role of chloride anions in activating CS_2_, we investigated the structural and energetic changes accompanying [CS_2_X]^−^ formation (Figure S6, CSI). Our results suggest that under the studied conditions, [CS_2_X]^−^ formation is energetically favorable only for lighter halogens (F, Cl), in agreement with experimental findings (Figure S8, CSI). Additionally, the formation of [CS_2_Cl]^−^ triggers a cascade of electronic rearrangements. As Cl^–^ approaches CS_2_, electron density shifts notably toward the latter, particularly from the central C atom to S and into the emerging C–Cl bonding region, as reflected in the QTAIM atomic charges (Table 1).

**Table 1:** Atomic Charges of the C, S, and Cl Atoms (Q_C_, Q_S_, and Q_Cl_, Respectively) of the Naked CS_2_ Skeleton and [CS_2_Cl]^−^ Adduct^a^

Entry	System	Q_c_	Q_s_	Q_Cl_
1	CS_2_	–1.40	+0.70	-
2	[CS_2_Cl]^−^	–0.88	+0.11	–0.35

a All values are given in electrons (*e*).

This electronic redistribution enhances S nucleophilicity, shifting its charge from a highly cationic state (QS = +0.70 *e*) to a nearly neutral one (QS = +0.04 *e* to +0.20 *e*, see Table S2, CSI). Simultaneously, the C atom becomes more electropositive, while the halide undergoes a slight depopulation, facilitating C–Cl bond formation. According to our mechanistic proposal (discussed below), the reaction initiates via a nucleophilic attack on the nitrone. Thus, the enhancement of electron density on the S atom promotes its nucleophilic character, which is crucial for CS_2_ activation. Moreover, the halide’s proximity also weakens intramolecular CS_2_ interactions, particularly the C–S bond, as indicated by a drop in the delocalization index from 2.0 (CS_2_) to approximately 1.6 ([CS_2_Cl]^−^) electron pairs (see CSI, Table S5). For other halogens, less electron density shifts toward S, thus decreasing its nucleophilicity and potentially reducing the overall reactivity of the system (CSI, Tables S2–S5).

Next, we investigated the reaction pathway connecting nitrones **1** to thioamides **3** through high-level computational studies, following the procedure outlined in CSI Section S1. For simplicity, we focused on the conversion of nitrone **1b** to thioamide **3b**, used as an archetypal system to model the reaction under study. The reaction initiates with the approach of the [CS_2_Cl]^−^ adduct [tetramethylammonium (TMA) was used as a counterion to facilitate the calculations] to the nitrone **1b**, followed by a concerted nucleophilic attack, as shown in Figure 1. In this step, one of the S atoms attacks the electrophilic α-carbon of the nitrone while the oxygen atom of the nitrone is simultaneously engaging the carbon of the [CS_2_Cl]^−^ moiety. This concerted interaction results in the formation of a five-membered heterocyclic intermediate **IA**, when the reaction originates from *Z*-nitrone **1b**. DFT calculations indicate a relatively low activation barrier for this step, with a value of 22 kcal/mol for *Z*-**1b** (**TS-IA**).

**Figure 1:**
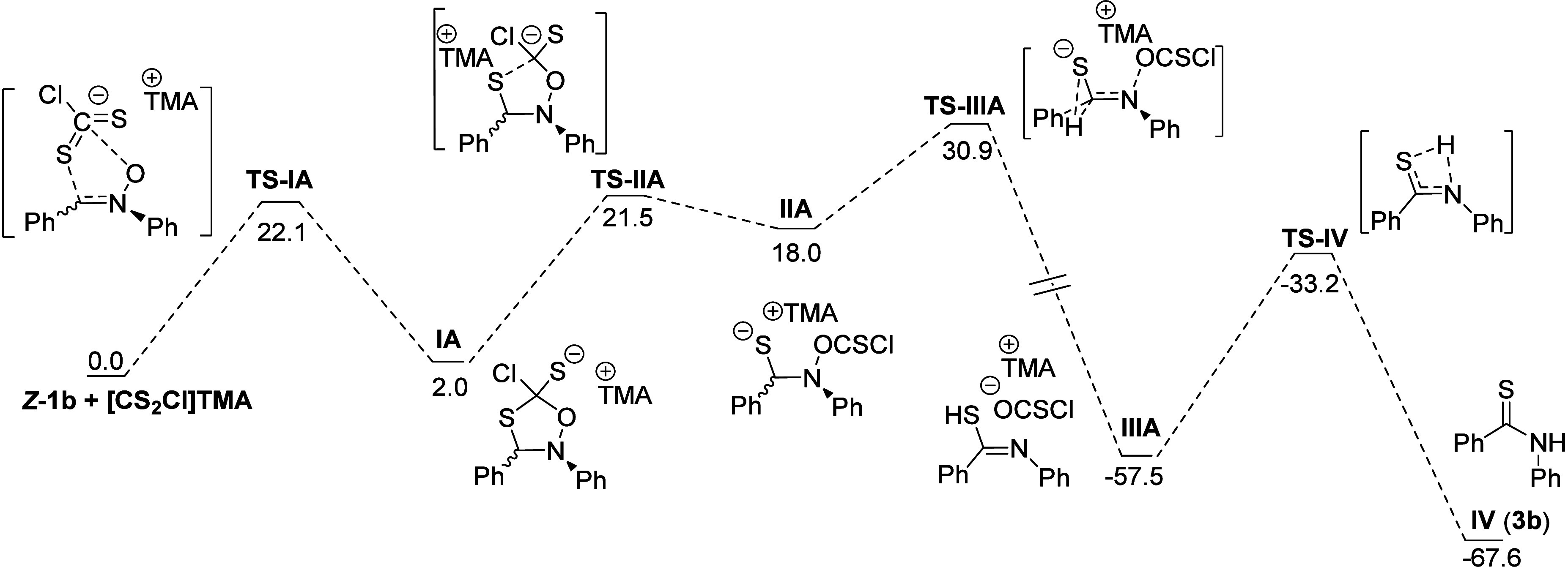
Reaction energy profile from ***Z*** **-1b** to **IV** (**3b**).

Then, the reaction evolves through a common pathway involving the cleavage of the C–S bond via the corresponding transition state (**TS-IIA**, with an activation energy of 20 kcal/mol). This leads to the formation of intermediate **IIA**, which undergoes a geminal hydrogen shift from the α-carbon to the S atom. This process is coupled with the cleavage of the N–O bond, facilitating the release of OCSCl^–^ and TMA^+^, ultimately yielding a highly stable imidothioic-acid intermediate **IIIA**, roughly 60 kcal/mol more stable than the starting reagents. This isomer **A** reacts with barriers of 20 and 15 kcal/mol, C–S bond cleavage being the rate-limiting step of this second reaction stage.

The final step involves the tautomerization of intermediate **IIIA** to afford final thioamide **IV** (**3b**). This transformation occurs via an intramolecular hydrogen shift from S to N, with an associated energy barrier of approximately 24 kcal/mol.

The nature of the halide anion impacts the energy landscape of the reaction mechanism (see CSI, Figure S9). The most notable variation occurs in the CSO extrusion process. The introduction of bulky halides such as Br^–^ or I^–^ severely hinders this step. This effect, likely due to increased steric hindrance and polarizability of Br^–^, highlights the crucial role of lighter halides (F^–^ and Cl^–^) in facilitating the reaction, consistent with experimental findings (see Table SI_4, Section III in Supporting Information) and the current mechanistic proposal.

Subsequently, to establish the scope of our transformation, a diverse set of nitrones (**1b-ah**) featuring various functional groups and substitution patterns were reacted with CS_2_ under optimized conditions (Scheme 2). All of the reactions proceeded smoothly (Scheme 3). Halide-containing substrates (**1a**, **1c**–**h**, **1ab**, and **1ac**) were well-tolerated, although a decrease in efficiency was observed for nitrone **1g** as a consequence of steric factors. Strongly deactivating substituents, such as CF_3_, NO_2_, or nitrile moieties (present in nitrones **1i**, **1j**, and **1k**, respectively), were also tolerated without impact on reactivity. The same applies to electron-donating groups (nitrones **1r** and **1t**), except for hydroxyl-substituted nitrone **1s**. Particularly noteworthy are nitrones **1l** and **1m**, bearing highly reactive terminal alkyne or boronic ester functionalities, which underwent efficient conversion to the corresponding thioamides in good yields without the formation of undesired byproducts. Moreover, the formation of thioamides can be carried out in the presence of other carbonyl functions, as evidenced by the successful conversion of compounds **1n**–**q** and **1u**. It is important to remark that these last substrates are not tolerated by classical methodologies, as they result in complex reaction mixtures (see Section VII in Supporting Information). Finally, our methodology is also capable of coping with nitrones containing heterocyclic scaffolds (**1v**, **1w**, **1x**),^15^ polyaromatic systems (**1y**), alkenylic (**1z**), or aliphatic frameworks (**1aa**, **1ad**,^15^ **1ae**,^15^ and **1ah**). Moreover, our protocol can be applied to the synthesis of thiolactams **3af** and **3ag**, which are considered as prevalence motifs in pharmaceutical products.^2^ This broad collection of examples highlights the potential of our transformation.

**Scheme 3:**
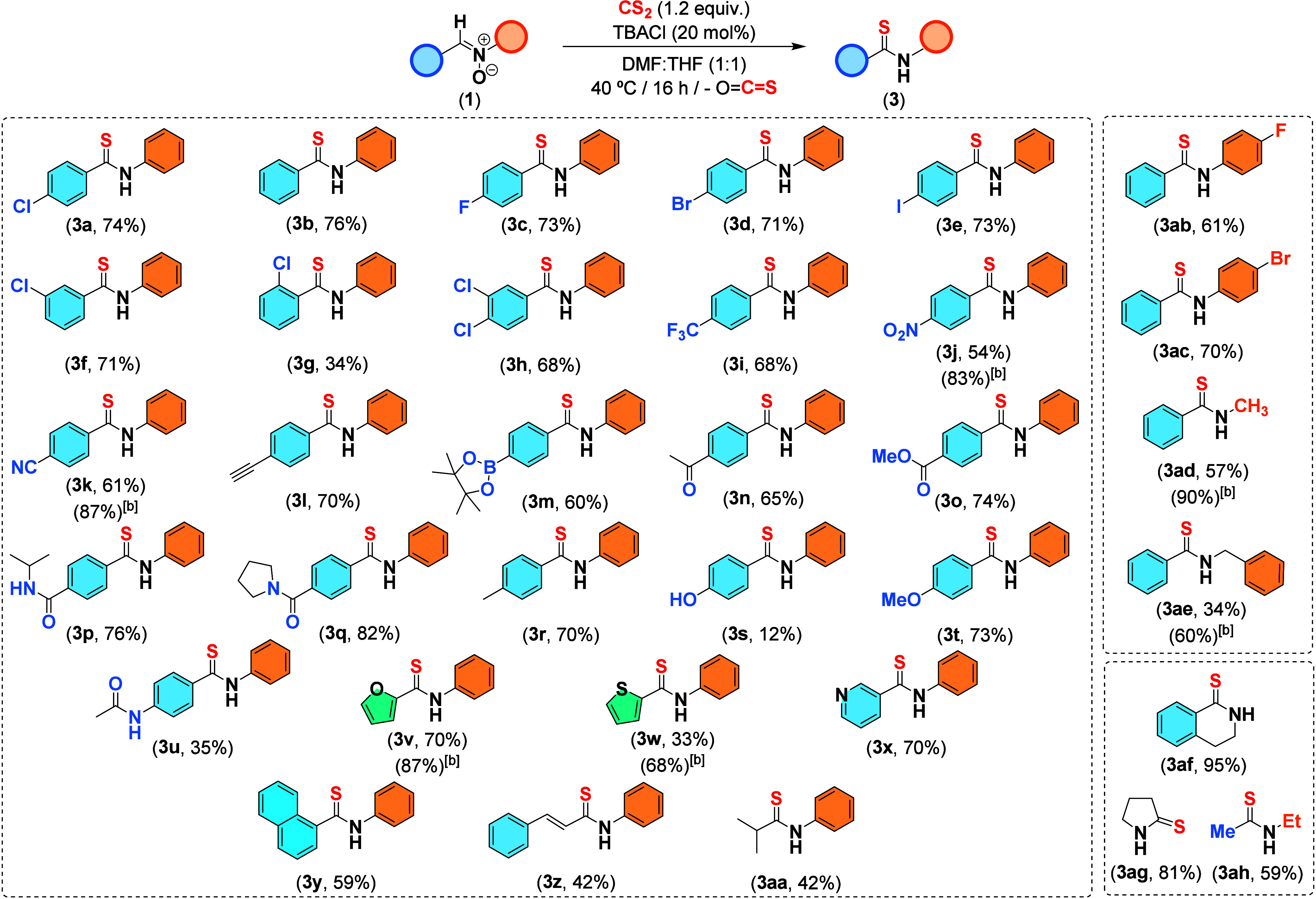
Chloride-Catalyzed Synthesis of Thioamides **3a**–**3ah** by Insertion of CS_2_ in Nitrones **1a-1ah** ^a^

a General conditions: to a solution of the desired nitrone **1a-ah** (0.4 mmol) and TBACl (22 mg, 0.08 mmol) in a mixture of anhydrous DMF:THF 1:1 (2 mL), CS_2_ (29 μL, 0.48 mmol) was added, and the reaction mixture was stirred for 16 h at 40 °C (oil bath) in a 10 mL sealed tube under argon atmosphere. Yields of pure analytical products **3a-ah** isolated by flash chromatography are given.
b Three equivalents of CS_2_ (73 μL, 1.2 mmol) were used, and the reaction mixture was stirred for 40 h at 40 °C (oil bath).

Nonetheless, to validate the robustness of our strategy, a gram-scale reaction between CS_2_ and nitrone **1a** was set up, yielding thioamide **3a** without a loss of efficiency (74%, Scheme 4A). Also, to showcase the synthetic utility of our protocol, we applied it to the synthesis of UC-781 (**3ai**, Scheme 4B), a potent and selective HIV-1 nonnucleoside reverse transcriptase inhibitor.^16^

**Scheme 4:**
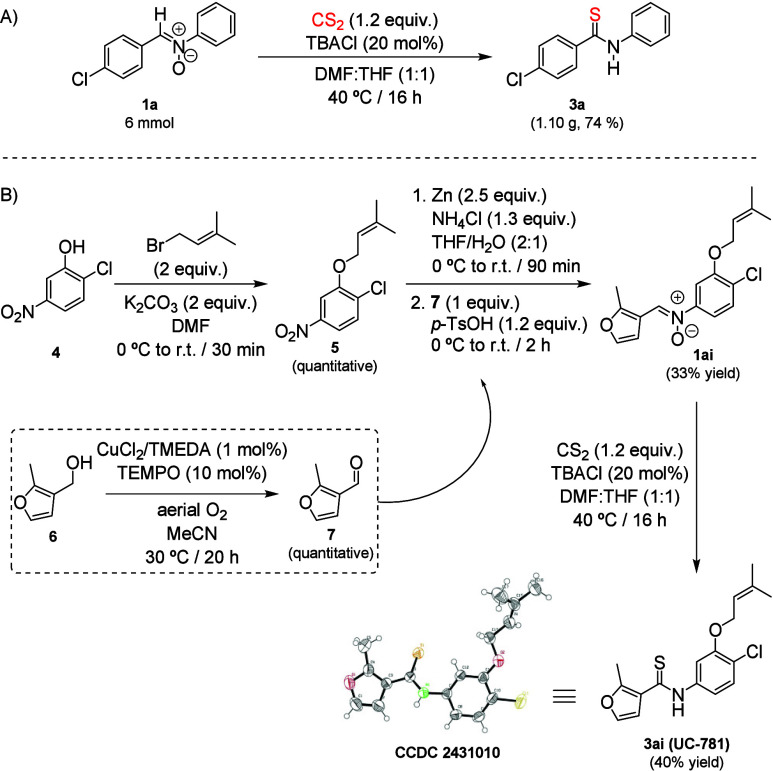
Scalability and Synthetic Application of the Insertion of CS_2_ in Nitrones

In summary, we have developed a novel strategy for the synthesis of thioamides from nitrones and carbon disulfide (CS_2_), using a simple chloride anion as a catalyst. It implies an unprecedented reactivity mode for nitrones, which have a broad audience in synthetic organic chemistry. Our transformation proceeds under mild conditions and in the absence of metals, exhibiting high chemoselectivity and broad tolerance for substrates. Thus, this methodology enables the tailored synthesis of thioamides, allowing their preparation in the presence of functional groups sensitive to thiolating agents. This represents an advantage over most previously reported methods. The synthetic utility of this method has been demonstrated in the preparation of UC-781, a potent HIV-1 non-nucleoside reverse transcriptase inhibitor.

Importantly, mechanistic insights gained through DFT calculations have revealed a key role of the chloride anion in the activation of CS_2_, facilitating the formation of a reactive [CS_2_Cl]^−^ intermediate, which undergoes a cascade of electronic rearrangements. This boost in reactivity opens up new conceptual avenues for the functionalization of this simple and underexploited reagent, paving the way for further developments in heterocyclic chemistry.

## Supplementary Material





## Data Availability

The data underlying this study are available in the published article and its .

## Notes

Computational Supporting Information (PDF)
Experimental procedures, NMR data and spectra, and X-ray crystal data (PDF)

## †

Physical and Analytical Chemistry Department, Universidad de Oviedo. Avenida Julián Clavera 8, 33006 Oviedo, Asturias (Spain)

## Notes

In memory of Dr. Iván Lavandera, a brilliant bioorganic chemist whose kindness and dedication profoundly impacted our Department.
