# Supplementary material for: Unexpected Reactivity of Nitrones: Catalytic Insertion of CS2
Source: Org Lett. 2025 Jul 24;27(30):8338–43. doi: 10.1021/acs.orglett.5c02611 (PMC12322958; doi:10.1021/acs.orglett.5c02611)
Supplement: Supplementary file 1 [file ol5c02611_si_001.pdf]

Computational Supporting Information

Unexpected Reactivity of Nitrones: Catalytic Insertion of CS_2

Marcos López-Aguilar, Nicolás Ríos-Lombardía, Daniel Barrena-Espés, Miguel Gallegos, Joaquín García-Álvarez,* Carmen Concellón,* and Vicente del Amo.*

Contents

1. Computational Details 3
2. Mechanistic Studies 4
3. Formation of the $[CS_2X]^-$ adduct 10
4. Optimized Geometries 17
5. References 69

1. Computational Details

DFT calculations were performed using Gaussian16 (C.01) **[1]**. Geometry optimizations were conducted at the M06-2X/def2-SVP level, followed by single-point energy refinements at M06-2X/def2-TZVP. Reaction intermediates and products were confirmed as local minima, while transition states were identified as first-order saddle points via Hessian eigenvalue analysis. Such a level of theory was selected due to its proven accuracy in similar mechanistic studies **[2]** .Solvent effects (THF) were modeled with the SMD implicit solvation method **[1]**, applied in both energy and force evaluations. Reaction progress was analyzed via Intrinsic Reaction Coordinate (IRC) calculations, and flat energy surfaces were explored using Local Quadratic Approximation (LQA). To reduce computational costs, halide (X^-) anions were paired with TMA cations instead of the bulkier, and more expensive to compute, TBA. This approximation is justified, as TMA and TBA exhibit similar behavior, and the counter cation acts primarily as a spectator. Unless stated otherwise, all reported reaction barriers correspond to Gibbs free energies (353.15 K, M06-2X/def2-TZVP) relative to the reaction complex, with translational entropy corrections following the Morokuma scheme. Molecular visualizations were generated using Jmol **[3]**. Finally, Quantum Theory of Atoms in Molecules (QTAIM) **[4]** calculations were performed wih the aid of the AIMAll **[5]** and PROMOLDEN **[6]** suites using the default integration parameters. The corresponding wavefunctions were obtained at the M06-2X/def2-SVP level of theory in the gas-phase using Gaussian16. **[1]**

2. Mechanistic Studies

Cyclization of the starting nitrone **Z-1b** (competitive reaction)

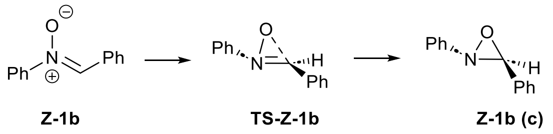

Scheme S1. Structures of the different stationary states involved in the transformation of **Z-1b** to **Z-1b (c)**.

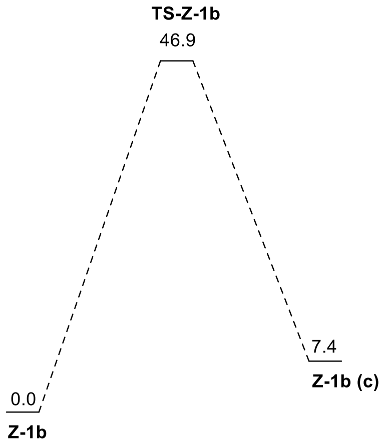

Figure S1. Reaction energy profile for the transformation of **Z-1b** to **Z-1b (c)**. All energies are reported, relative to the reactant complex, as the Morokuma-corrected Gibbs free energies (353.15 K).

Nucleophilic attack to the nitrone and cyclization.

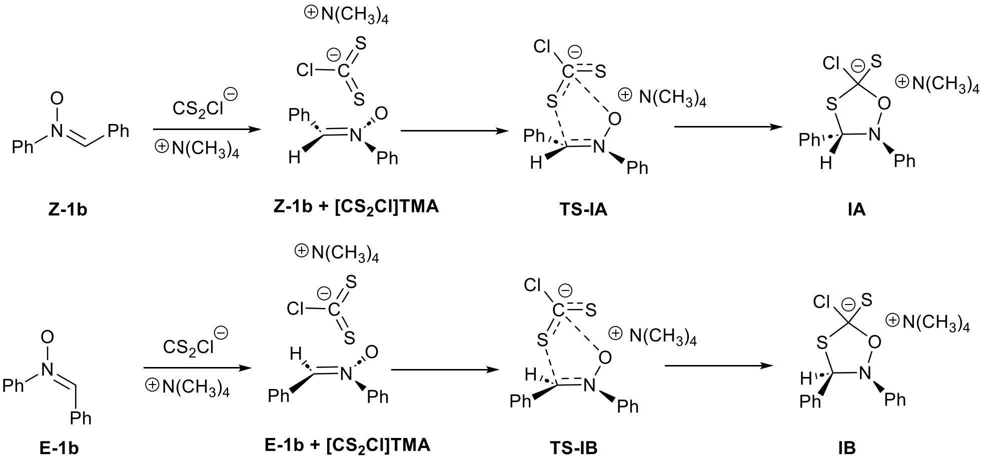

Scheme S2. Structures of the different stationary states involved in the transformation of **Z-1b**/**E-1b** to **IA**/**IB**.

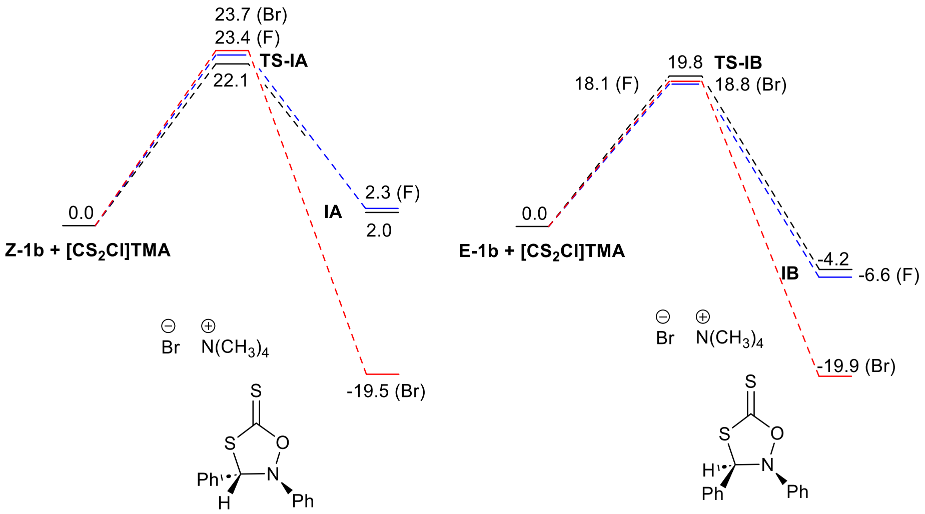

Figure S2. Reaction energy profile for the transformation of **Z-1b/E-1b** to **IA/IB**. All energies are reported, relative to the reactant complex, as the Morokuma-corrected Gibbs free energies (353.15 K).

<u>COS extrusion starting from **IA**</u>

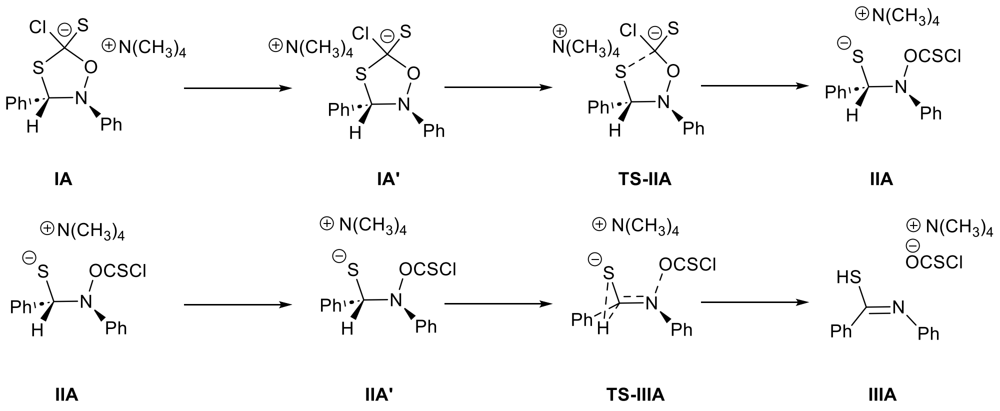

Scheme S3. Structures of the different stationary states involved in the transformation of **IA** to **IIIA**.

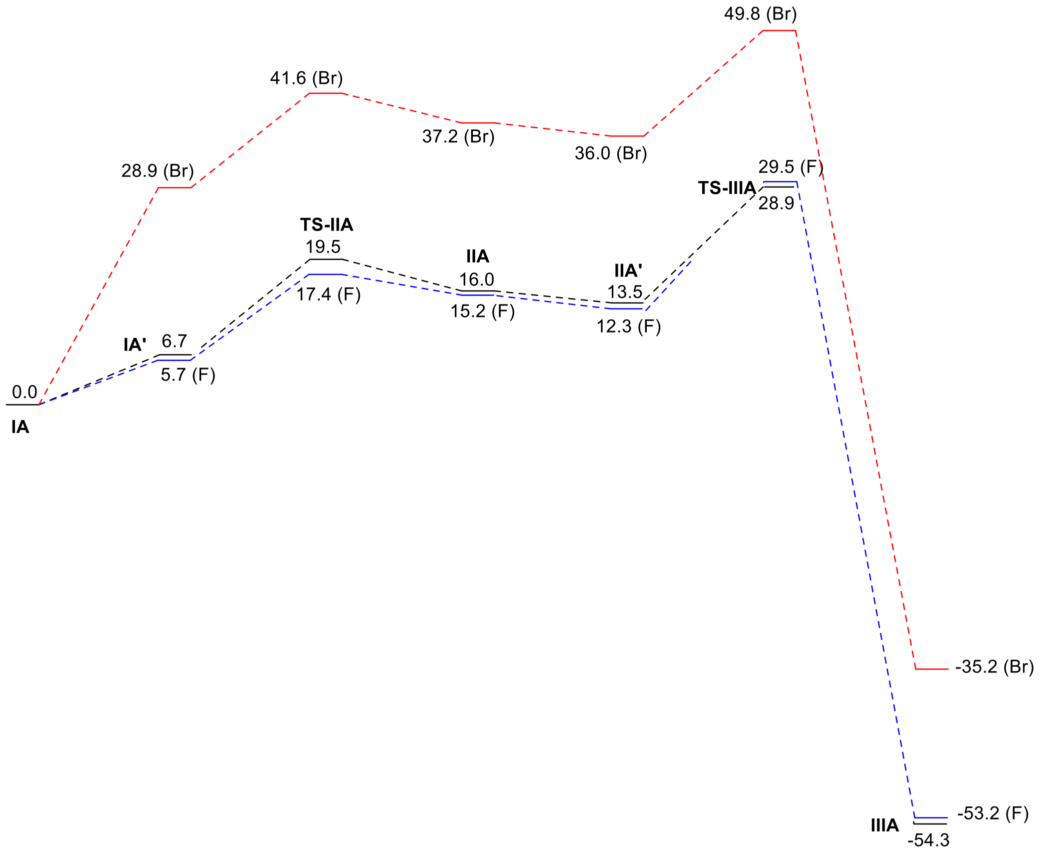

Figure S3. Reaction energy profile for the transformation of **IA** to **IIIA**. All energies are reported, relative to the reactant complex, as the Morokuma-corrected Gibbs free energies (353.15 K).

<u>COS extrusion starting from **IB**</u>

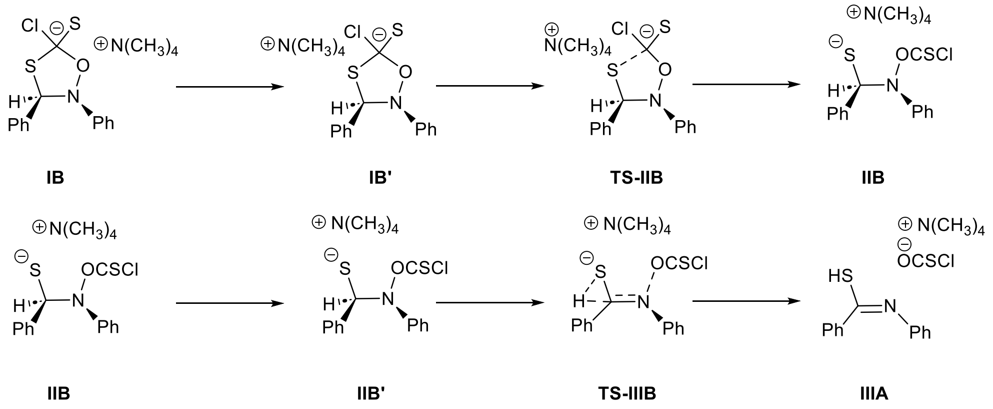

Scheme S4. Structures of the different stationary states involved in the transformation of **IB** to **IIIA**.

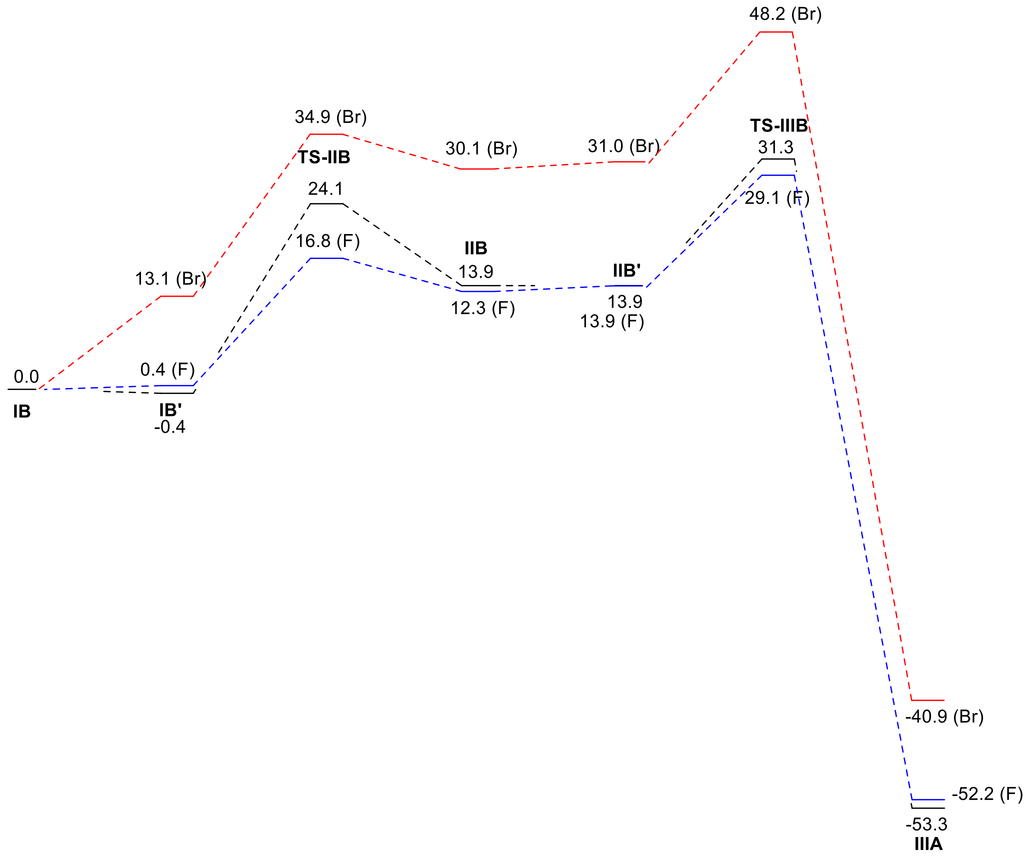

Figure S4. Reaction energy profile for the transformation of **IB** to **IIIA**. All energies are reported, relative to the reactant complex, as the Morokuma-corrected Gibbs free energies (353.15 K).

The H-migration step is, on the one hand, the rate-limiting step according to the free energy profile. On the other hand, it is also the step involving the greatest rearrangement in the system, where several factors could affect it. For such a reason, the IRC involving the TS-III for the isomers is included (Figures S5 and S6).

As observed in this process, and clarified by the IRC, the process is not induced by the H-migration but the N-O bond rupture instead. The release of the adduct CSO-X triggers and electron rearrangement mainly on the N atom. This is, at the same time, assisted by the rearrangement in the C and S atoms, which ultimately leads to the unexpected H-migration.

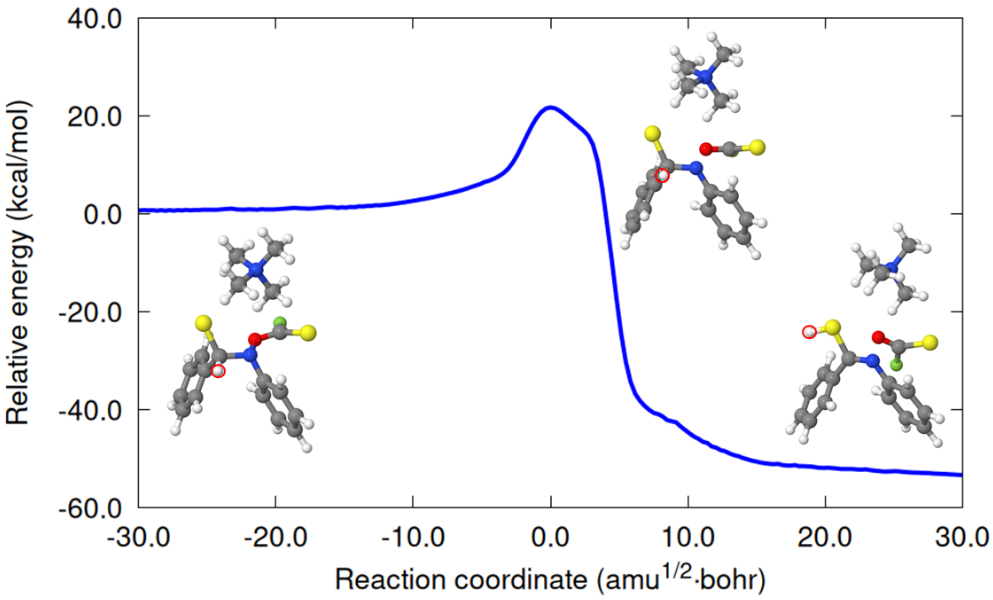

Figure S5. Full IRC for the TS-III (isomer A). Energies, M06-2X/def2-SVP(SMD=THF), relative to the first stage of the IRC (in kcal/mol).

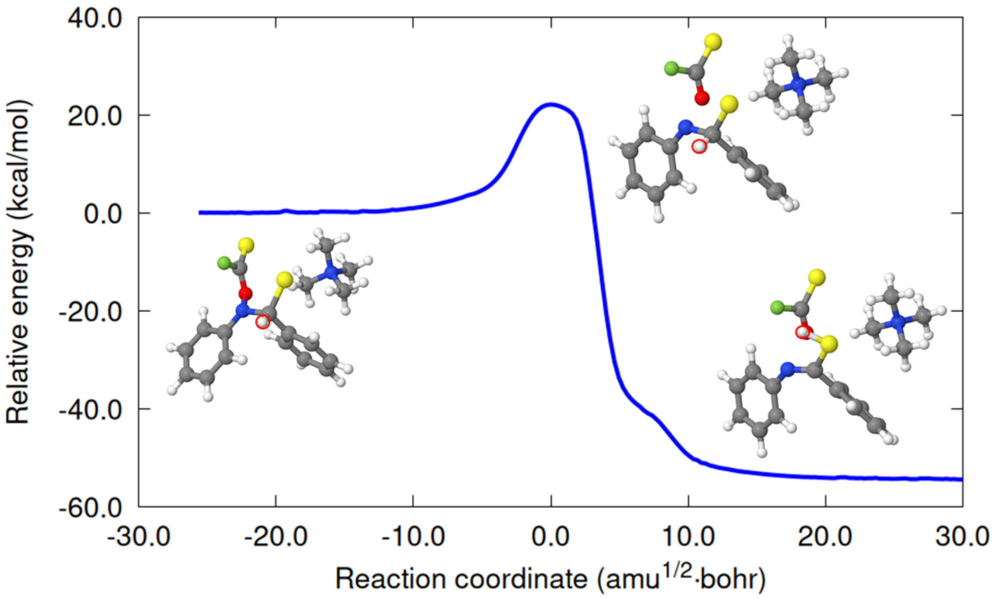

Figure S6. Full IRC for the TS-III (isomer B). Energies, M06-2X/def2-SVP(SMD=THF), relative to the first stage of the IRC (in kcal/mol).

Final Tautomerism

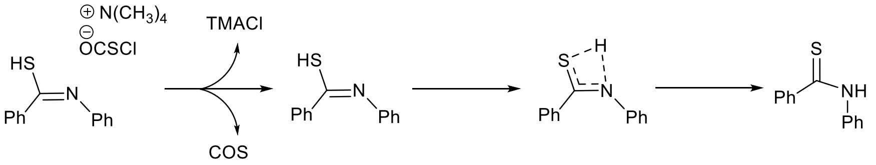

Scheme S5. Structures of the different stationary states involved in the transformation of **IIIA** to **IV**.

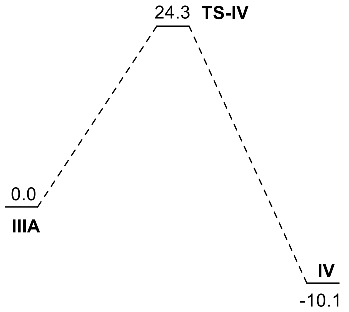

Figure S7. Reaction energy profile for the transformation of **IIIA** to **IV**. All energies are reported, relative to the reactant complex, as the Morokuma-corrected Gibbs free energies (353.15 K).

3. Formation of the $[CS_2X]^-$ adduct

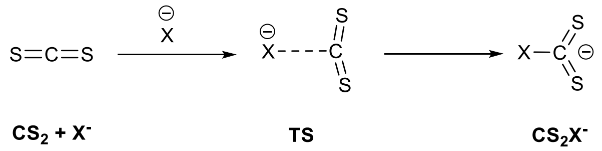

Scheme S6. Representation of the linear collision between a free X^- anion and CS_2 to produce the resultant $[CS_2X]^-$ adduct.

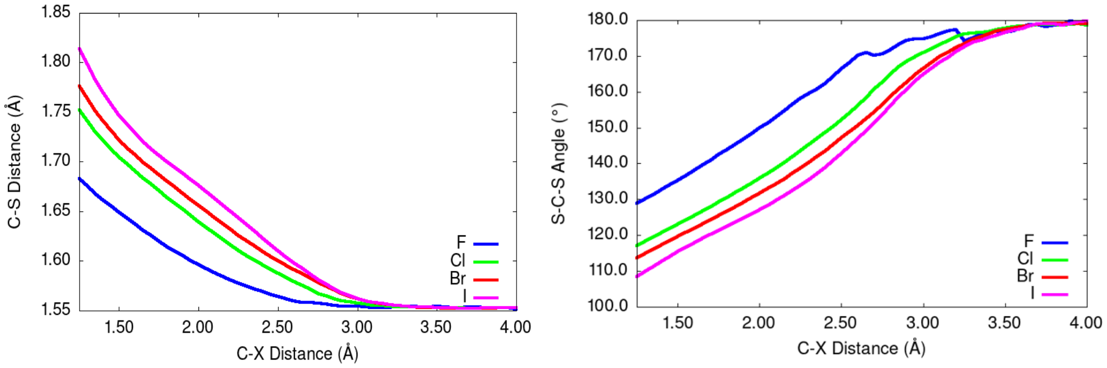

Figure S8. Evolution of the C-S distance (left) and S-C-S angle (right) with the C-X distance throughout the formation of the CS_2X^- adduct. The results for X= F, Cl, Br and I, are shown. The energies are reported relative to the global minimum of each potential energy surface.

Halogen (X)	C-X Distance (Å)	C-S Distance (Å)	S-C-S Angle (º)
F^-	1.35	1.67	131.4
Cl^-	1.85	1.66	131.8
Br^-	2.05	1.65	133.0
I^-	2.35	1.63	137.2

Table S1. Main geometrical features of the optimized $[CS_2X]^-$ adducts.

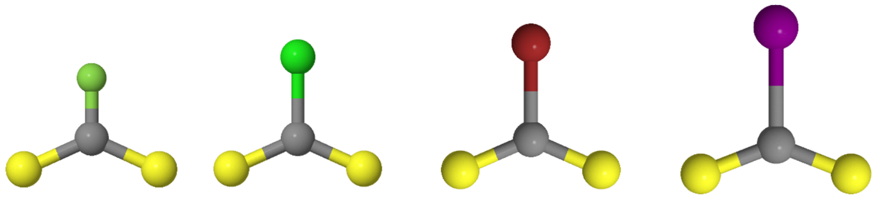

Figure S9. Optimized geometries of the $[CS_2X]^-$ complexes for X= F, Cl, Br and I, respectively. For $[CS_2I]^-$, the metastable structure corresponding to a local plateau in the evolution of the energy gradient is shown.

Q	CS_2F	CS_2Cl	CS_2Br
X	-0.66	-0.35	-0.37
C	-0.41	-0.88	-1.02
S	0.04	0.11	0.20

Table S2. Atomic charges (Q) (in a.u.) where X = F, Cl, Br.

LI	CS_2F	CS_2Cl	CS_2Br
X	9.07	16.67	34.73
C	4.36	4.70	4.84
S	14.93	14.79	14.67

Table S3. Localization indices (LI) (in a.u.) where X = F, Cl, Br.

$\nabla^2\rho$	CS_2F	CS_2Cl	CS_2Br
BCP(X−C)	-0.02	-0.08	-0.01
BCP(S−C)	0.28	0.30	0.36

Table S4. Laplacian of the electron density at the bond critical point (BCP) where X = F, Cl, Br.

DI	CS_2F	CS_2Cl	CS_2Br
X-C	0.84	0.97	0.86
X-S	0.17	0.19	0.21
C-S	1.63	1.69	1.75
S-S	0.27	0.30	0.31

Table S5. Delocalization indices (DI) (in a.u.) for each interaction where X = F, Cl, Br.

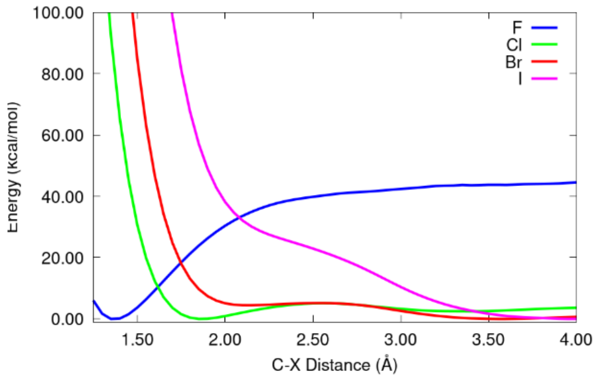

Figure S10. Evolution of the total electronic energy (M06-2X/def2-SVP in the gas-phase) with the C–X distance throughout the formation of the $[CS_2X]^-$ adduct. The energies are reported, in kcal/mol, relative to the global minimum of each potential energy surface.

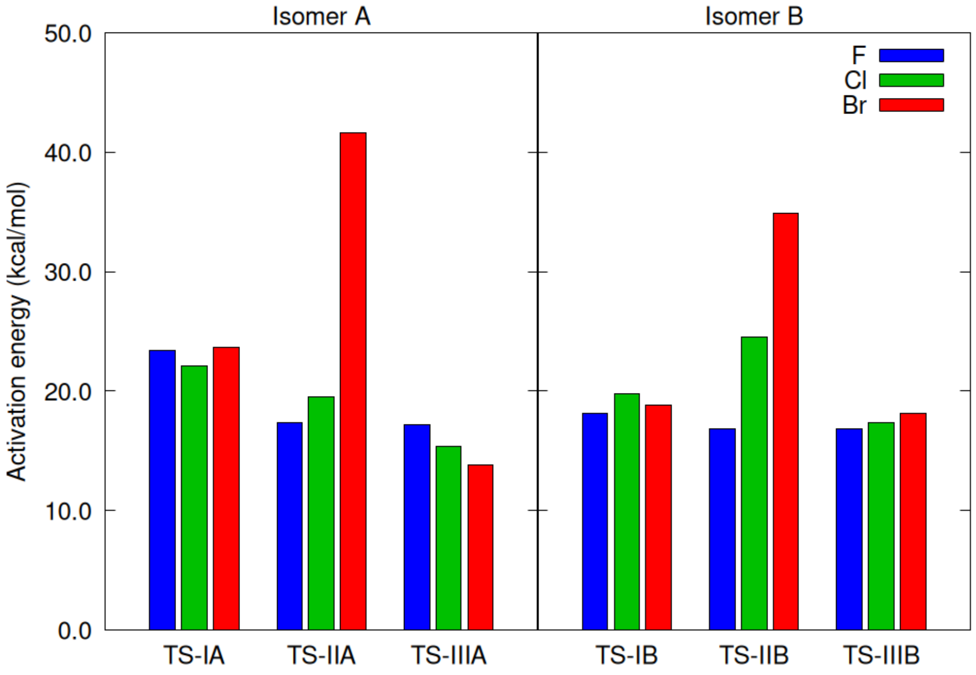

Figure S11. Activation Energies, reported in kcal/mol, for each of the halogen-involving steps involved in the reaction mechanism. The impact of the halogen (X) on both approximations (isomers A and B) is shown.

To complete the study, we decided to evaluate the impact of diffuse functions on our system. For such a purpose, calculations were performed at the at the M062X/6-31+G(d) level of theory, followed by M062X/6-311+G(d,p) single point energy evaluation for the refinement of the reaction energy profiles. In both cases, THF was used as a solvent, modeled according to the SMD solvation scheme.

The following figure (Figure S12) shows the dissociation curves of the various adducts after incorporating diffuse functions. The results are qualitatively very similar to those obtained with the baseline (without diffuse functions). In particular, the positions of the energy minima are virtually identical for both basis sets, which showcases the validity of the level of theory employed in our calculations. That said, and as expected, differences arise in the potential barrier observed at the limits of extreme compression and dissociation.

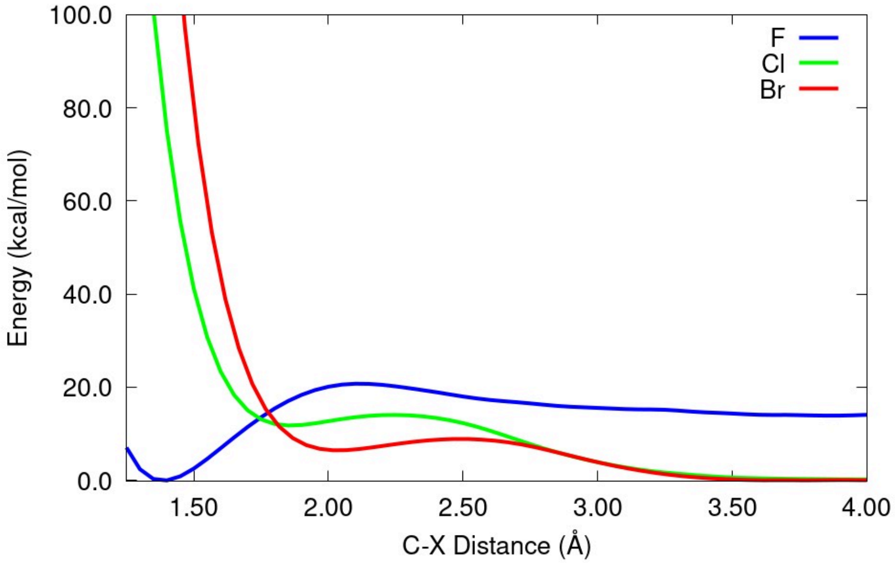

Figure S12. Potential energy landscape for the dissociation of the CS2X- adducts, computed at the M062X/6-31+G(d) level of theory, using THF as implicit solvent (SMD).

In addition to evaluating the accuracy of adduct formation, we also assessed the impact of diffuse basis sets on the reaction profile. The following tables (Tables S6 to S8) present the Gibbs free energies of the intermediates and transition states involved in the studied transformation, calculated with and without diffuse functions. For the diffuse basis sets, both fully optimized geometries and single-point energy evaluations on geometries optimized without diffuse functions are included.

Compound	def2-SVP//def2-TZVP	6-31+G(d)//6-311+G(d,p)	def2-SVP//6-311+G(d,p) (S-P)
Z-1.TMA CS2F	0.00	0.00	0.00
TS-1A	23.38	22.14	21.88
1A	2.32	-0.88	-0.63
E-1. TMA CS2F	0.00	0.00	0.00
TS-1B	18.06	17.61	16.95
1B	-6.63	-6.60	-7.19
1A	0.00	0.00	0.00
1A’	5.69	4.14	5.89
TS-2A	17.44	18.92	18.13
2A	15.24	15.84	16.12
2A’	12.13	13.70	13.64
TS-3A	29.53	29.72	29.87

3A	-53.20	-51.76	-51.86
1B	0.00	0.00	0.00
1B’	0.36	0.64	0.51
TS-2B	16.84	17.76	17.70
2B	12.26	13.15	13.50
TS-3B	29.06	27.48	28.23
3A	-52.15	-51.02	-51.25

Table S6. Gibbs free energies at 313 K for the transformation of compound 1 into 3A, mediated by CS_2F–TMA. The reaction pathway is divided into separate blocks, each with its own reference point for relative free energies. Results are shown both with and without diffuse functions. For calculations including diffuse functions, both geometrically optimized structures and single-point energy evaluations are presented.

Compound	def2-SVP//def2-TZVP	6-31+G(d)//6-311+G(d,p)	def2-SVP//6-311+G(d,p) (S-P)
Z-1.TMA CS2Cl	0.00	0.00	0.00
TS-1A	22.14	22.19	23.16
1A	2.01	1.85	1.47
E-1. TMA CS2Cl	0.00	0.00	0.00
TS-1B	19.75	18.42	18.94
1B	-4.16	-5.15	-4.65
1A	0.00	0.00	0.00
1A’	6.71	5.03	6.75
TS-2A	19.50	17.98	19.73
2A	15.98	16.30	15.61
2A’	13.54	12.89	13.46
TS-3A	28.89	26.61	29.12
3A	-54.28	-57.16	-53.45
1B	0.00	0.00	0.00
1B’	-0.42	0.91	-0.24
TS-2B	24.09	21.50	23.39
2B	13.92	15.02	14.63

TS-3B	31.31	29.64	28.47
3A	-53.29	-55.36	-53.14

Table S7. Gibbs free energies at 313 K for the transformation of compound 1 into 3A, mediated by CS_2Cl–TMA. The reaction pathway is divided into separate blocks, each with its own reference point for relative free energies. Results are shown both with and without diffuse functions. For calculations including diffuse functions, both geometrically optimized structures and single-point energy evaluations are presented.

Compound	def2-SVP//def2-TZVP	6-31+G(d)//6-311+G(d,p)	def2-SVP//6-311+G(d,p) (S-P)
Z-1.TMA CS2Br	0.00	0.00	0.00
TS-1A	23.72	24.31	23.27
1A	-19.54	-18.30	-20.56
E-1. TMA CS2Br	0.00	0.00	0.00
TS-1B	18.84	15.29	16.63
1B	-19.93	-27.51	-21.82
1A	0.00	0.00	0.00
1A’	28.92	27.07	28.23
TS-2A	41.58	39.97	41.47
2A	37.22	35.72	37.50
2A’	36.04	33.58	36.09
TS-3A	49.82	48.49	47.21
3A	-35.20	-36.44	-36.15
1B	0.00	0.00	0.00
1B’	13.14	19.62	14.58
TS-2B	34.89	39.13	35.27
2B	30.12	35.22	30.65
TS-3B	48.20	50.89	45.85
3A	-40.87	-35.42	-41.23

Table S8. Gibbs free energies at 313 K for the transformation of compound 1 into 3A, mediated by CS_2Br–TMA. The reaction pathway is divided into separate blocks, each with its own reference point for relative free energies. Results are shown both with and without diffuse functions. For calculations including diffuse functions, both geometrically optimized structures and single-point energy evaluations are presented.

Given that the observed energy differences are minimal, this confirms the reliability of our approximation with the original basis set.

4. Optimized Geometries

The current section gathers the energetically minimized geometries of the different stationary points found across the computational studies. All values are reported, as XYZ Cartesian coordinates, in Angstroms.

[E-1b]

N	0.731719	1.715442	0.002852
O	1.459418	2.747681	0.064393
C	-0.576435	1.760120	0.003356
H	-0.967822	2.778163	-0.050420
C	-1.517840	0.638620	0.099655
C	-1.277650	-0.528527	0.846755
C	-2.762357	0.785780	-0.535581
C	-2.243124	-1.528358	0.920164
H	-0.337497	-0.649637	1.386579
C	-3.724598	-0.218530	-0.461300
H	-2.969103	1.698563	-1.098502
C	-3.466068	-1.382879	0.261752
H	-2.041447	-2.426456	1.506500
H	-4.681563	-0.089064	-0.969701
H	-4.218947	-2.170456	0.323207
C	1.466908	0.465396	-0.104922
C	2.481090	0.234073	0.819389
C	1.195311	-0.425829	-1.138267
C	3.213604	-0.947282	0.730167
H	2.679578	0.977883	1.591259
C	1.943779	-1.598668	-1.224586
H	0.410199	-0.204223	-1.862521
C	2.943643	-1.864317	-0.287988
H	4.002828	-1.149889	1.455687
H	1.745821	-2.305683	-2.031478
H	3.522992	-2.786329	-0.357283

[Z-1b]

N	-0.472437	0.494494	-0.095719
O	-0.240394	1.713989	-0.329543
C	0.438163	-0.423616	0.121468
H	0.063282	-1.413967	0.371917
C	-1.880059	0.122958	-0.045436
C	-2.324430	-1.120040	-0.492403
C	-2.768888	1.081634	0.436862
C	-3.685984	-1.413201	-0.424871
H	-1.626001	-1.844222	-0.912587
C	-4.124917	0.773822	0.504270
H	-2.380256	2.050495	0.747453
C	-4.585739	-0.472875	0.076795
H	-4.043807	-2.380573	-0.780399
H	-4.826596	1.514405	0.891072
H	-5.650063	-0.708086	0.125199
C	1.878031	-0.223075	0.072054
C	2.675321	-1.355140	0.341415
C	2.516485	0.997599	-0.225013

C	4.061717	-1.275506	0.315231
H	2.189021	-2.305731	0.572229
C	3.908944	1.065235	-0.251007
H	1.910861	1.875341	-0.431016
C	4.685770	-0.060978	0.017703
H	4.660211	-2.163045	0.527415
H	4.392120	2.015985	-0.483763
H	5.774956	0.005124	-0.003559

[Z-1b (c)]

N	-0.481139	-0.496534	-0.551442
O	-0.061002	-1.659202	0.190916
C	0.422898	-0.406951	0.561038
H	0.011697	-0.009188	1.497816
C	1.853413	-0.099540	0.292248
C	2.554117	0.755281	1.144193
C	2.493439	-0.666064	-0.815585
C	3.893643	1.051552	0.887459
H	2.049509	1.188965	2.010558
C	3.829578	-0.370267	-1.068852
H	1.933108	-1.335742	-1.471108
C	4.530587	0.489273	-0.217905
H	4.440412	1.720724	1.553802
H	4.330277	-0.811217	-1.932455
H	5.578648	0.718674	-0.418564
C	-1.842853	-0.147972	-0.286308
C	-2.713597	-0.979950	0.417778
C	-2.286088	1.070877	-0.808976
C	-4.037016	-0.577093	0.605840
H	-2.353452	-1.932539	0.804661
C	-3.604609	1.466384	-0.603542
H	-1.589994	1.693297	-1.374116
C	-4.485410	0.643216	0.102902
H	-4.722266	-1.228378	1.151466
H	-3.949729	2.420481	-1.005657
H	-5.520840	0.951806	0.254614

[TS-Z-1b]

N	0.499067	-0.487680	0.005132
O	0.088926	-1.638378	-0.691150
C	-0.453651	0.251081	-0.555145
H	-0.161647	1.082692	-1.219175
C	-1.868005	0.137339	-0.240312
C	-2.362150	-0.794536	0.693070
C	-2.741441	1.054002	-0.841637
C	-3.709465	-0.793445	1.015438
H	-1.672849	-1.519881	1.126403
C	-4.097903	1.045124	-0.518250
H	-2.349993	1.775997	-1.561688
C	-4.579545	0.125048	0.409628
H	-4.096640	-1.513643	1.737712
H	-4.776092	1.757820	-0.989287
H	-5.640382	0.115975	0.666262

C	1.848715	-0.141069	0.058501
C	2.820349	-1.077520	-0.313317
C	2.219124	1.130853	0.516789
C	4.166214	-0.732459	-0.219114
H	2.491943	-2.047513	-0.684533
C	3.569378	1.465001	0.583259
H	1.450069	1.837914	0.835914
C	4.548343	0.536328	0.222473
H	4.926787	-1.460445	-0.507891
H	3.859327	2.456938	0.934582
H	5.604831	0.800452	0.286680

[Z-1b + [CS_2F]TMA]

N	0.090582	-0.105952	-1.549819
C	-0.542886	-1.069923	-0.937324
O	-0.478311	0.902499	-2.092502
S	2.438336	-0.196077	2.136397
C	1.200266	-1.283079	1.931776
S	1.200144	-2.937106	1.706474
F	-0.058352	-0.726168	1.952730
C	1.545944	-0.120110	-1.592361
C	2.268869	-1.308914	-1.489678
C	2.193809	1.104831	-1.748771
C	3.660201	-1.256706	-1.501562
H	1.767797	-2.272593	-1.408292
C	3.585976	1.141976	-1.758569
H	1.599919	2.010689	-1.859282
C	4.322272	-0.035667	-1.628952
H	4.228095	-2.183949	-1.413495
H	4.097736	2.099145	-1.870259
H	5.412852	-0.003583	-1.635948
C	-1.988020	-1.215354	-0.836707
C	-2.446096	-2.105105	0.154360
C	-2.924296	-0.559618	-1.658693
C	-3.808053	-2.325357	0.329065
H	-1.711194	-2.604249	0.792303
C	-4.285500	-0.799549	-1.484043
H	-2.570206	0.126935	-2.424660
C	-4.732058	-1.673048	-0.490745
H	-4.151531	-3.010409	1.105940
H	-5.006866	-0.296255	-2.130437
H	-5.801143	-1.847968	-0.356770
H	0.072895	-1.804945	-0.420631
N	-0.949625	3.008812	0.851812
C	-1.286916	3.939612	1.960557
H	-2.177947	4.512301	1.679533
H	-0.437183	4.612109	2.123506
H	-1.481320	3.349073	2.863001
C	-0.706121	3.772821	-0.403957
H	-0.468516	3.048446	-1.194033
H	0.127925	4.463988	-0.236143
H	-1.616341	4.329365	-0.655488

C	0.275070	2.232445	1.206733
H	0.096270	1.688578	2.138282
H	1.114654	2.928309	1.318846
H	0.476770	1.516489	0.403136
C	-2.071783	2.054482	0.623859
H	-2.187430	1.425581	1.514526
H	-1.813119	1.448937	-0.252977
H	-2.986904	2.627998	0.434980

[Z-1b + [CS_2Cl]TMA]

N	0.376292	-0.789988	-1.215155
C	-0.104972	-1.812018	-0.564124
O	-0.324312	0.160664	-1.706783
S	1.955874	1.811628	2.097338
C	1.420892	0.242465	2.081223
S	2.228381	-1.199717	2.069269
Cl	-0.443775	0.068981	2.104730
C	1.812638	-0.671862	-1.376726
C	2.594200	-1.799003	-1.624758
C	2.378337	0.596363	-1.280036
C	3.974886	-1.652345	-1.739770
H	2.129765	-2.779133	-1.739517
C	3.760555	0.728107	-1.383372
H	1.738202	1.457860	-1.097019
C	4.560440	-0.393028	-1.608445
H	4.593967	-2.528869	-1.936544
H	4.215396	1.714047	-1.276978
H	5.642993	-0.283502	-1.689901
C	-1.520948	-2.102895	-0.378320
C	-1.857859	-2.951154	0.691596
C	-2.542114	-1.621976	-1.216960
C	-3.183008	-3.299254	0.929362
H	-1.064951	-3.319120	1.346497
C	-3.866022	-1.987870	-0.981404
H	-2.282879	-0.967722	-2.047307
C	-4.192304	-2.817890	0.092155
H	-3.431099	-3.948876	1.770339
H	-4.652312	-1.618433	-1.642504
H	-5.232701	-3.091982	0.275981
H	0.631504	-2.449034	-0.076514
N	-1.893524	2.993384	-0.512340
C	-2.609938	4.225579	-0.094737
H	-3.560890	4.280140	-0.637274
H	-1.984759	5.093274	-0.335014
H	-2.790014	4.174865	0.985130
C	-1.632525	3.029118	-1.978988
H	-1.074254	2.123601	-2.245143
H	-1.044826	3.927190	-2.202284
H	-2.594162	3.065887	-2.504277
C	-0.590779	2.890658	0.210971
H	-0.778694	2.879782	1.289075
H	0.030894	3.750223	-0.067133

H	-0.123089	1.944971	-0.084248
C	-2.712453	1.790759	-0.186482
H	-2.892969	1.775068	0.894402
H	-2.137264	0.908831	-0.492912
H	-3.660793	1.856074	-0.733364

[Z-1b + [CS_2Br]TMA]

N	0.353814	-0.819396	-1.384042
C	-0.199127	-1.799621	-0.724457
O	-0.283607	0.153268	-1.916624
S	2.149206	1.725019	1.889892
C	1.605869	0.179350	1.938665
S	2.287048	-1.305366	1.892724
Br	-0.537358	0.076859	2.145450
C	1.796888	-0.784846	-1.524485
C	2.522082	-1.962812	-1.698964
C	2.427794	0.455934	-1.498700
C	3.908710	-1.894107	-1.810287
H	2.009869	-2.923519	-1.761536
C	3.815473	0.511045	-1.601629
H	1.831653	1.359026	-1.379810
C	4.558041	-0.660433	-1.752318
H	4.482694	-2.811554	-1.948527
H	4.319669	1.477433	-1.554583
H	5.645047	-0.611530	-1.833306
C	-1.630783	-2.037465	-0.597220
C	-2.034132	-2.930819	0.412216
C	-2.607945	-1.475162	-1.438635
C	-3.376852	-3.244169	0.588636
H	-1.277651	-3.365184	1.069237
C	-3.950920	-1.805096	-1.264074
H	-2.299544	-0.789916	-2.225205
C	-4.341419	-2.680649	-0.250293
H	-3.674289	-3.930756	1.382945
H	-4.701319	-1.371248	-1.927769
H	-5.396173	-2.926552	-0.114555
H	0.490363	-2.462782	-0.203647
N	-1.604466	3.130858	-0.762717
C	-2.202595	4.438508	-0.389436
H	-3.126554	4.578800	-0.962501
H	-1.484671	5.232554	-0.624798
H	-2.418670	4.429962	0.684950
C	-1.313220	3.106141	-2.223915
H	-0.836697	2.146806	-2.457961
H	-0.640803	3.940177	-2.456310
H	-2.257575	3.218856	-2.769032
C	-0.333792	2.914703	-0.007254
H	-0.542670	2.964436	1.066584
H	0.383572	3.690259	-0.301568
H	0.033307	1.912761	-0.256883
C	-2.546209	2.024137	-0.429050
H	-2.740720	2.047086	0.649231

H	-2.059831	1.081469	-0.708254
H	-3.475285	2.174700	-0.991811

[E-1b + [CS_2F]TMA]

N	0.535048	0.233888	-0.850835
C	1.445063	-0.569910	-1.323961
O	-0.705824	0.088663	-1.091121
H	1.044511	-1.314406	-2.015301
S	-2.321787	-1.279200	2.077996
C	-1.070634	-2.010810	1.232739
S	0.548987	-2.117033	1.573683
F	-1.463519	-2.663267	0.103237
C	2.875785	-0.593274	-0.998858
C	3.762741	-0.993617	-2.010939
C	3.389177	-0.311817	0.279387
C	5.132411	-1.070081	-1.768107
H	3.368787	-1.241309	-2.999083
C	4.758066	-0.392665	0.517862
H	2.710043	-0.054077	1.093324
C	5.635295	-0.763303	-0.503892
H	5.808244	-1.374787	-2.568999
H	5.142235	-0.176256	1.516163
H	6.707398	-0.825765	-0.309534
C	0.854462	1.373925	-0.011920
C	1.749198	2.342609	-0.456218
C	0.191944	1.482339	1.208492
C	2.007681	3.442424	0.361262
H	2.238014	2.233928	-1.425710
C	0.468647	2.581740	2.018916
H	-0.508151	0.701206	1.514991
C	1.374440	3.558833	1.599207
H	2.705423	4.211290	0.026117
H	-0.027561	2.673932	2.986380
H	1.584200	4.417992	2.238534
N	-4.253887	0.428201	-1.063139
C	-3.339368	1.377459	-0.364275
H	-3.942735	2.200186	0.036846
H	-2.596809	1.736254	-1.084035
H	-2.825860	0.825793	0.432331
C	-3.459200	-0.702480	-1.624940
H	-2.939877	-1.204938	-0.801104
H	-2.715692	-0.295491	-2.317006
H	-4.151932	-1.382869	-2.134101
C	-4.963601	1.136716	-2.159720
H	-5.547599	1.957184	-1.727027
H	-5.625193	0.425044	-2.666824
H	-4.217963	1.528565	-2.860686
C	-5.242683	-0.113473	-0.090170
H	-4.687861	-0.652038	0.688601
H	-5.917046	-0.794453	-0.622251
H	-5.806193	0.722537	0.339958

[E-1b + [CS_2Cl]TMA]

N	-0.589737	-0.313842	-0.856355
C	-1.515933	0.480259	-1.315927
O	0.643704	-0.163162	-1.122229
H	-1.133878	1.227224	-2.014991
S	2.278238	1.248492	1.983509
C	0.984503	2.002530	1.239134
S	-0.624525	1.987392	1.616999
Cl	1.432176	3.071428	-0.182544
C	-2.942051	0.486500	-0.971126
C	-3.844355	0.888890	-1.969132
C	-3.440123	0.183733	0.308486
C	-5.212232	0.946751	-1.712041
H	-3.463341	1.152839	-2.958096
C	-4.807443	0.246093	0.561329
H	-2.751628	-0.077969	1.113188
C	-5.699192	0.618867	-0.446901
H	-5.899279	1.253139	-2.502686
H	-5.178996	0.012708	1.560578
H	-6.769963	0.666281	-0.241317
C	-0.883477	-1.455824	-0.009720
C	-1.762748	-2.442466	-0.444906
C	-0.206762	-1.549892	1.204197
C	-1.991260	-3.546649	0.375837
H	-2.262967	-2.344279	-1.409699
C	-0.453458	-2.654051	2.017930
H	0.478187	-0.752428	1.503823
C	-1.343510	-3.649495	1.607414
H	-2.676977	-4.329474	0.048284
H	0.053136	-2.735415	2.981008
H	-1.529930	-4.512030	2.249424
N	4.142866	-0.780027	-0.992408
C	3.149332	-1.633239	-0.277356
H	3.674216	-2.509986	0.119975
H	2.363414	-1.916058	-0.984517
H	2.703609	-1.032696	0.524527
C	3.455428	0.422529	-1.548642
H	2.983175	0.963715	-0.720576
H	2.678413	0.090662	-2.243833
H	4.209074	1.040987	-2.050526
C	4.764585	-1.558424	-2.095054
H	5.277011	-2.427871	-1.667620
H	5.480423	-0.914315	-2.618576
H	3.973355	-1.882110	-2.780539
C	5.192141	-0.337171	-0.033599
H	4.705792	0.262965	0.745440
H	5.927450	0.268157	-0.576312
H	5.671107	-1.223751	0.397805

[E-1b + [CS_2Br]TMA]

N	0.704344	0.525900	-0.844720
C	1.555445	-0.368750	-1.263540
O	-0.540205	0.464155	-1.091829

H	1.099901	-1.122695	-1.908934
S	-2.196979	-0.742962	2.046644
C	-0.912792	-1.575392	1.397485
S	0.685087	-1.598409	1.779163
Br	-1.425868	-2.868121	-0.105675
C	2.982364	-0.475522	-0.940367
C	3.815019	-1.054523	-1.912314
C	3.544908	-0.104569	0.294252
C	5.177804	-1.222069	-1.677907
H	3.382812	-1.371467	-2.863966
C	4.906645	-0.277000	0.525022
H	2.910813	0.297473	1.085271
C	5.729988	-0.827854	-0.459451
H	5.809149	-1.666916	-2.448989
H	5.326783	0.011109	1.490189
H	6.796557	-0.961190	-0.270492
C	1.101571	1.688218	-0.071977
C	2.040976	2.578041	-0.583132
C	0.463384	1.904000	1.147527
C	2.371488	3.707773	0.164756
H	2.508896	2.384370	-1.549809
C	0.811760	3.032090	1.888090
H	-0.270970	1.178791	1.508270
C	1.763388	3.931295	1.400529
H	3.105730	4.415655	-0.222772
H	0.336481	3.208589	2.854403
H	2.028780	4.813481	1.985534
N	-3.995214	1.251654	-0.979935
C	-2.962741	2.097134	-0.312897
H	-3.448590	3.013954	0.041369
H	-2.168840	2.310413	-1.035387
H	-2.538124	1.520137	0.517318
C	-3.355429	0.006782	-1.500811
H	-2.866406	-0.506572	-0.664655
H	-2.594545	0.286380	-2.235800
H	-4.139847	-0.614812	-1.948968
C	-4.610347	2.008706	-2.101369
H	-5.101748	2.901260	-1.697269
H	-5.343394	1.363329	-2.599026
H	-3.818785	2.294825	-2.802959
C	-5.041620	0.879308	0.011571
H	-4.565497	0.278441	0.796094
H	-5.814217	0.293194	-0.499337
H	-5.472399	1.796276	0.430015

[TS-IA (F)]

N	1.189507	-0.388563	-0.528974
C	0.827570	-1.562285	0.062298
O	0.283782	0.590093	-0.488754
S	-0.398634	1.326270	3.282381
C	-0.408968	0.044246	2.256532
S	0.795164	-1.162758	2.132341

F	-1.522237	-0.201113	1.551779
C	2.539125	-0.018402	-0.648781
C	3.566782	-0.960888	-0.844240
C	2.866786	1.346585	-0.609724
C	4.885548	-0.538048	-0.975148
H	3.341328	-2.024970	-0.920384
C	4.192196	1.753186	-0.747779
H	2.059065	2.059436	-0.459118
C	5.212800	0.819706	-0.927927
H	5.668263	-1.283845	-1.127879
H	4.429362	2.818555	-0.708003
H	6.249084	1.142969	-1.036205
C	-0.483825	-2.167862	-0.357292
C	-1.140033	-3.125386	0.422833
C	-0.998815	-1.855527	-1.621091
C	-2.292680	-3.752870	-0.046880
H	-0.752668	-3.367416	1.413788
C	-2.154005	-2.481052	-2.089442
H	-0.489979	-1.104000	-2.226406
C	-2.807067	-3.430675	-1.303366
H	-2.793849	-4.496154	0.575869
H	-2.545301	-2.224133	-3.075972
H	-3.712275	-3.919185	-1.668529
H	1.634342	-2.300994	0.096076
N	-2.315726	2.507282	-0.916515
C	-3.493745	3.395699	-1.078496
H	-3.966498	3.182016	-2.044204
H	-3.155825	4.437833	-1.043143
H	-4.196359	3.197808	-0.260783
C	-1.317735	2.774226	-1.991967
H	-0.486726	2.070290	-1.842035
H	-0.979661	3.813323	-1.901202
H	-1.804079	2.618520	-2.962383
C	-1.672253	2.745280	0.409636
H	-2.381596	2.466322	1.196633
H	-1.417668	3.809569	0.483100
H	-0.776201	2.112187	0.461327
C	-2.733878	1.076306	-0.992762
H	-3.490069	0.895263	-0.219889
H	-1.834827	0.471528	-0.813512
H	-3.155769	0.893674	-1.988666

[TS-IA (Cl)]

N	1.110935	-0.562251	-0.584435
C	0.618165	-1.733863	-0.050973
O	0.313461	0.496307	-0.446114
S	-0.011780	1.182796	3.202690
C	-0.388264	-0.056232	2.192258
S	0.592762	-1.447060	1.960117
Cl	-2.042059	-0.154553	1.542983
C	2.493252	-0.328473	-0.633200
C	3.413153	-1.348553	-0.943532

C	2.971073	0.972566	-0.405911
C	4.774233	-1.067674	-1.000492
H	3.065773	-2.358229	-1.166860
C	4.337269	1.237872	-0.472256
H	2.246276	1.747356	-0.163070
C	5.250698	0.225177	-0.765818
H	5.471963	-1.871360	-1.244428
H	4.692726	2.253247	-0.283602
H	6.319345	0.438433	-0.816851
C	-0.722278	-2.200111	-0.551892
C	-1.391649	-3.252154	0.081150
C	-1.268193	-1.648254	-1.715151
C	-2.596667	-3.732404	-0.427171
H	-0.974949	-3.680948	0.994934
C	-2.477198	-2.125677	-2.221236
H	-0.741940	-0.830183	-2.207447
C	-3.148613	-3.165119	-1.577018
H	-3.110289	-4.550880	0.080495
H	-2.897179	-1.681800	-3.126264
H	-4.096695	-3.535435	-1.971346
H	1.353826	-2.546064	-0.068039
N	-1.574057	3.097108	-0.938553
C	-2.442844	4.288104	-1.112249
H	-2.806046	4.311879	-2.146259
H	-1.854550	5.187261	-0.895420
H	-3.285485	4.210477	-0.415568
C	-0.395095	3.184178	-1.847437
H	0.191812	2.267147	-1.698863
H	0.184131	4.075214	-1.577652
H	-0.758050	3.266074	-2.878813
C	-1.087111	3.017116	0.471764
H	-1.950565	2.884502	1.133809
H	-0.563718	3.951944	0.706699
H	-0.413771	2.151656	0.540384
C	-2.337784	1.855017	-1.257664
H	-3.228448	1.824701	-0.618886
H	-1.674709	1.004454	-1.047217
H	-2.628882	1.892623	-2.314569
	**[TS-IA (Br)]**		
N	1.174484	-0.783113	-0.594962
C	0.454089	-1.866905	-0.121323
O	0.543758	0.384866	-0.483590
S	0.151523	1.128255	3.060037
C	-0.397499	-0.014347	2.020008
S	0.292456	-1.580396	1.855019
Br	-2.182633	0.216027	1.250910
C	2.574216	-0.775045	-0.503812
C	3.341297	-1.924133	-0.776842
C	3.233626	0.420264	-0.173252
C	4.728919	-1.873038	-0.698138
H	2.854468	-2.853359	-1.076996

C	4.624884	0.455790	-0.104542
H	2.625925	1.296998	0.043973
C	5.384922	-0.685253	-0.362459
H	5.306632	-2.773746	-0.915562
H	5.121576	1.390860	0.163616
H	6.473924	-0.651489	-0.307633
C	-0.885246	-2.120868	-0.760807
C	-1.750720	-3.080514	-0.227409
C	-1.239973	-1.465174	-1.943548
C	-2.960341	-3.369389	-0.855020
H	-1.482685	-3.588442	0.701670
C	-2.452796	-1.751291	-2.569612
H	-0.560800	-0.718792	-2.355730
C	-3.319486	-2.699760	-2.025836
H	-3.628323	-4.118145	-0.425169
H	-2.722851	-1.228638	-3.489624
H	-4.270171	-2.920310	-2.514941
H	1.055233	-2.783351	-0.092448
N	-0.517065	3.394865	-1.154037
C	-1.013236	4.773034	-1.396139
H	-1.217376	4.891422	-2.466715
H	-0.244068	5.484890	-1.075406
H	-1.931620	4.921362	-0.816327
C	0.748842	3.164350	-1.908139
H	1.050740	2.126287	-1.713454
H	1.502602	3.872144	-1.543196
H	0.554857	3.333384	-2.974067
C	-0.252037	3.192826	0.302686
H	-1.194144	3.317805	0.849182
H	0.478534	3.944347	0.625967
H	0.137778	2.173819	0.430866
C	-1.532804	2.398846	-1.603970
H	-2.469303	2.596303	-1.069366
H	-1.144736	1.400633	-1.356893
H	-1.679642	2.521771	-2.683842

[IA (F)]

N	1.235051	-0.273889	-0.515247
C	1.244873	-1.477480	0.291066
O	0.258453	0.587174	-0.007342
S	-0.174100	1.933909	2.264465
C	-0.049637	0.421151	1.386573
S	1.166588	-0.807867	2.012671
F	-1.282666	-0.253314	1.350588
C	2.484434	0.389549	-0.676864
C	3.473321	-0.262015	-1.425479
C	2.730584	1.658597	-0.143980
C	4.710032	0.345937	-1.622360
H	3.260340	-1.241582	-1.859077
C	3.969940	2.261898	-0.362558
H	1.960195	2.148235	0.455329
C	4.963802	1.614148	-1.095550

H	5.475882	-0.170542	-2.203941
H	4.160749	3.250577	0.059528
H	5.930523	2.093595	-1.257110
C	0.112686	-2.423501	-0.061560
C	-0.217662	-3.476449	0.797789
C	-0.583519	-2.285125	-1.265646
C	-1.225074	-4.376869	0.458930
H	0.309412	-3.579662	1.748628
C	-1.600813	-3.181233	-1.600226
H	-0.324194	-1.465515	-1.937231
C	-1.924097	-4.230138	-0.741017
H	-1.471049	-5.194118	1.139333
H	-2.140714	-3.058272	-2.541360
H	-2.718616	-4.931155	-1.002783
H	2.215426	-1.976487	0.168606
N	-3.264346	1.994390	-0.777234
C	-4.411244	2.484166	-1.586639
H	-4.088407	2.577232	-2.629886
H	-4.724481	3.459238	-1.196418
H	-5.230620	1.760474	-1.505712
C	-2.135035	2.965545	-0.863112
H	-1.320573	2.611757	-0.215649
H	-2.494863	3.940695	-0.514292
H	-1.812777	3.029703	-1.908847
C	-3.678570	1.852451	0.649230
H	-4.476722	1.102998	0.703459
H	-4.043524	2.825327	0.998500
H	-2.804432	1.542605	1.237073
C	-2.816784	0.670311	-1.298428
H	-3.655333	-0.031993	-1.224617
H	-1.972090	0.326203	-0.692792
H	-2.515915	0.797281	-2.345120

[IA (Cl)]

N	-0.972726	0.722090	0.467035
C	-2.014765	-0.010891	-0.225054
O	0.267322	0.393230	-0.094538
S	1.439308	0.701758	-2.470836
C	0.238029	-0.024001	-1.451876
S	-1.495217	0.117034	-1.991474
Cl	0.582732	-1.970650	-1.277692
C	-1.145339	2.133492	0.555044
C	-2.174187	2.602821	1.381912
C	-0.321551	3.037089	-0.122528
C	-2.389630	3.971573	1.514361
H	-2.793376	1.885049	1.924781
C	-0.539367	4.407147	0.032833
H	0.462443	2.661253	-0.783385
C	-1.569112	4.881726	0.844276
H	-3.195405	4.328953	2.158121
H	0.102150	5.110748	-0.501309
H	-1.732850	5.954693	0.955752

C	-2.203736	-1.423453	0.291426
C	-2.959380	-2.341548	-0.444148
C	-1.672986	-1.809100	1.524184
C	-3.175471	-3.628438	0.042378
H	-3.363334	-2.051253	-1.416638
C	-1.884037	-3.100919	2.008741
H	-1.084060	-1.091368	2.096895
C	-2.633997	-4.014741	1.270229
H	-3.763596	-4.336916	-0.543823
H	-1.456645	-3.394331	2.969589
H	-2.796257	-5.025915	1.647738
H	-2.953292	0.551974	-0.127471
N	3.837487	-0.476530	0.906901
C	5.039321	-0.597543	1.774145
H	4.831668	-0.102861	2.730064
H	5.884090	-0.113327	1.271402
H	5.250244	-1.661081	1.934279
C	3.515171	0.964282	0.691006
H	2.670776	1.029893	-0.007994
H	4.397735	1.453852	0.263088
H	3.262918	1.411877	1.659305
C	4.100204	-1.121246	-0.412809
H	4.324253	-2.180603	-0.240971
H	4.957985	-0.619500	-0.875904
H	3.207271	-1.006188	-1.042129
C	2.680954	-1.150213	1.564303
H	2.931475	-2.208654	1.701544
H	1.801572	-1.047040	0.918314
H	2.509706	-0.669880	2.534700

[IA (Br)]

N	1.740193	-0.250702	-0.250078
C	1.598727	-1.584796	0.320568
O	0.896431	0.613848	0.457573
S	0.121470	1.380194	2.819491
C	0.802897	0.333985	1.783516
S	1.457220	-1.242081	2.141126
Br	-3.861785	0.034452	0.888143
C	3.063226	0.283715	-0.337460
C	3.977701	-0.397911	-1.150126
C	3.441482	1.458781	0.316765
C	5.276493	0.082014	-1.281220
H	3.659434	-1.294772	-1.685598
C	4.745496	1.934582	0.164571
H	2.732809	2.005437	0.938327
C	5.667143	1.251583	-0.624933
H	5.984899	-0.456082	-1.913158
H	5.037882	2.851428	0.679155
H	6.684230	1.629884	-0.735358
C	0.430592	-2.360580	-0.232254
C	-0.896270	-2.003485	0.035166
C	0.697309	-3.447532	-1.071556

C	-1.941079	-2.717681	-0.548944
H	-1.159142	-1.170764	0.696596
C	-0.349957	-4.167362	-1.646965
H	1.732503	-3.733442	-1.274334
C	-1.670360	-3.800196	-1.388644
H	-2.962280	-2.397767	-0.327676
H	-0.131320	-5.017124	-2.295986
H	-2.491116	-4.362716	-1.837688
H	2.535065	-2.128632	0.151388
N	-1.859647	2.291332	-1.651889
C	-1.085994	3.132307	-2.600234
H	-0.017018	2.986466	-2.404971
H	-1.360046	4.181948	-2.442993
H	-1.330079	2.825134	-3.623744
C	-1.548467	2.695771	-0.248540
H	-2.118546	2.039638	0.425702
H	-1.841434	3.745060	-0.122215
H	-0.472760	2.573065	-0.084302
C	-3.320859	2.460538	-1.895504
H	-3.537892	2.148675	-2.923984
H	-3.573910	3.518357	-1.757518
H	-3.855294	1.829256	-1.170765
C	-1.501642	0.853532	-1.837195
H	-1.732324	0.572077	-2.871822
H	-2.103905	0.270570	-1.125330
H	-0.430209	0.729906	-1.637494

[TS-IB (F)]

N	0.476746	-0.008861	-0.595179
C	1.395890	-0.961537	-0.743304
O	-0.757392	-0.423414	-0.553280
H	1.044684	-1.796380	-1.350768
S	-2.110612	-1.291043	1.988617
C	-0.917401	-1.860014	0.902940
S	0.738866	-2.092995	1.267781
F	-1.386751	-2.725367	-0.045391
C	2.856719	-0.743054	-0.656518
C	3.658905	-1.172948	-1.720511
C	3.459583	-0.155100	0.465232
C	5.040707	-0.986279	-1.682691
H	3.193713	-1.651425	-2.585089
C	4.838696	0.026565	0.501871
H	2.839383	0.141156	1.313192
C	5.631553	-0.381883	-0.574032
H	5.656284	-1.318460	-2.520329
H	5.300533	0.482240	1.379321
H	6.712993	-0.238010	-0.540921
C	0.732227	1.278776	-0.025734
C	1.582492	2.176101	-0.672768
C	0.090268	1.618941	1.167355
C	1.815313	3.425664	-0.100688
H	2.057269	1.891050	-1.613077

C	0.330268	2.873431	1.726148
H	-0.569205	0.885796	1.641726
C	1.192458	3.775524	1.098622
H	2.481333	4.131732	-0.599402
H	-0.156261	3.145662	2.664555
H	1.376434	4.754984	1.543342
N	-4.258863	0.426956	-0.947123
C	-3.245978	1.348733	-0.353367
H	-3.774300	2.226289	0.038410
H	-2.538665	1.635823	-1.138947
H	-2.714091	0.803205	0.435919
C	-3.578394	-0.787727	-1.483903
H	-3.088666	-1.306803	-0.652804
H	-2.820897	-0.471963	-2.208568
H	-4.339841	-1.419994	-1.955881
C	-4.983565	1.120512	-2.043504
H	-5.478224	2.007947	-1.632535
H	-5.725191	0.431795	-2.464331
H	-4.257024	1.411358	-2.810720
C	-5.219612	0.011883	0.113293
H	-4.648437	-0.502952	0.896464
H	-5.959689	-0.663107	-0.332118
H	-5.710267	0.908103	0.510432

[TS-IB (Cl)]

N	-0.533804	-0.117505	-0.705898
C	-1.388610	0.944473	-0.567359
O	0.730704	0.246727	-0.703037
H	-1.052519	1.777711	-1.192893
S	2.064444	1.189855	2.071589
C	0.884243	1.714909	0.992581
S	-0.806858	1.697761	1.311609
Cl	1.368037	3.004552	-0.195475
C	-2.870711	0.752836	-0.534604
C	-3.632888	1.245259	-1.599630
C	-3.515970	0.104359	0.527026
C	-5.017483	1.074542	-1.616836
H	-3.134060	1.762656	-2.422126
C	-4.898473	-0.062557	0.510600
H	-2.927825	-0.268507	1.368255
C	-5.651686	0.418096	-0.562855
H	-5.601367	1.459375	-2.454723
H	-5.391775	-0.568293	1.342357
H	-6.735118	0.285959	-0.572901
C	-0.815603	-1.392898	-0.129790
C	-1.755084	-2.238596	-0.724002
C	-0.120445	-1.795604	1.016258
C	-2.019688	-3.483841	-0.154546
H	-2.273731	-1.913022	-1.627403
C	-0.387904	-3.045058	1.572680
H	0.599367	-1.104629	1.462743
C	-1.337743	-3.890441	0.993091

H	-2.756500	-4.143077	-0.616954
H	0.142920	-3.357918	2.473986
H	-1.544010	-4.866715	1.435170
N	4.197497	-0.691475	-0.883810
C	3.137694	-1.535069	-0.257334
H	3.584019	-2.499234	0.014231
H	2.318620	-1.651836	-0.973838
H	2.757190	-0.999808	0.621330
C	3.623620	0.640257	-1.240131
H	3.324004	1.138211	-0.310610
H	2.730798	0.488332	-1.855374
H	4.395436	1.213628	-1.767526
C	4.711906	-1.361988	-2.105907
H	5.112446	-2.343492	-1.826918
H	5.500350	-0.738526	-2.542991
H	3.883538	-1.476433	-2.813875
C	5.306122	-0.495672	0.089245
H	4.893839	-0.005921	0.979460
H	6.071420	0.138029	-0.373540
H	5.725680	-1.474730	0.347369

[TS-IB (Br)]

N	0.658638	0.242617	-0.572899
C	1.521274	-0.774799	-0.622630
O	-0.595097	-0.091690	-0.526009
H	1.119590	-1.636503	-1.161070
S	-2.017394	-0.742506	2.041393
C	-0.817574	-1.416006	1.058179
S	0.811757	-1.659876	1.452383
Br	-1.508079	-2.876186	-0.247347
C	2.992879	-0.632193	-0.551146
C	3.765934	-1.219207	-1.560463
C	3.632997	0.032663	0.505442
C	5.156479	-1.114233	-1.535846
H	3.271082	-1.757189	-2.371908
C	5.020859	0.132253	0.529414
H	3.036049	0.456424	1.315163
C	5.785313	-0.434534	-0.493654
H	5.748975	-1.569772	-2.330996
H	5.510995	0.648360	1.356565
H	6.873602	-0.354784	-0.470434
C	0.990312	1.549549	-0.087173
C	1.852094	2.366486	-0.818116
C	0.414559	1.985726	1.108246
C	2.161674	3.635925	-0.331712
H	2.276882	2.003226	-1.755425
C	0.729798	3.258651	1.580947
H	-0.254461	1.311801	1.651825
C	1.602937	4.082759	0.866601
H	2.837495	4.280355	-0.896281
H	0.295275	3.607101	2.519622
H	1.846425	5.077295	1.244151

N	-3.937817	1.338902	-0.844002
C	-2.757536	2.074941	-0.304285
H	-3.101765	3.045559	0.072400
H	-2.030677	2.203605	-1.113754
H	-2.312430	1.461797	0.488325
C	-3.503085	-0.004033	-1.330563
H	-3.103213	-0.563720	-0.476891
H	-2.710141	0.130380	-2.073556
H	-4.375565	-0.507285	-1.764314
C	-4.545162	2.112478	-1.957338
H	-4.841584	3.098293	-1.580218
H	-5.421320	1.567875	-2.327474
H	-3.799986	2.218907	-2.753972
C	-4.934565	1.151850	0.246267
H	-4.452891	0.573395	1.044347
H	-5.794478	0.605200	-0.157517
H	-5.244969	2.137540	0.611492

[IB (F)]

N	1.362618	0.252167	-0.151991
C	0.794508	0.959447	0.971454
O	0.681957	-0.941482	-0.348182
H	1.585008	1.537153	1.467397
S	-0.468025	-3.045762	0.780559
C	0.621620	-1.680442	0.866707
S	0.230733	-0.370801	2.140140
F	1.927767	-2.062817	1.132737
C	2.732849	0.246337	-0.447629
C	3.337369	-0.863246	-1.060342
C	3.495819	1.405229	-0.227071
C	4.682869	-0.814207	-1.412391
H	2.747864	-1.759917	-1.239743
C	4.843848	1.432567	-0.579062
H	3.034341	2.296422	0.200409
C	5.451144	0.326186	-1.171441
H	5.137575	-1.690243	-1.879343
H	5.420240	2.340949	-0.393164
H	6.506025	0.353611	-1.447338
C	-0.348949	1.876617	0.578610
C	-1.161592	2.445945	1.567184
C	-0.601603	2.175037	-0.761999
C	-2.216194	3.286665	1.220394
H	-0.975786	2.216954	2.619664
C	-1.664163	3.013619	-1.111654
H	0.035483	1.738478	-1.532743
C	-2.476546	3.568392	-0.123863
H	-2.841710	3.721421	2.002108
H	-1.853604	3.234406	-2.164194
H	-3.305973	4.223364	-0.396516
N	-3.470746	-1.009978	-0.937358
C	-3.084613	-0.333220	0.335202
H	-2.796367	0.697906	0.098849

H	-2.242455	-0.885358	0.775716
H	-3.950884	-0.342549	1.007784
C	-4.586483	-0.268525	-1.579654
H	-4.252672	0.756323	-1.781261
H	-5.442016	-0.260517	-0.894432
H	-4.852454	-0.774562	-2.514731
C	-2.290671	-1.039235	-1.849729
H	-1.484663	-1.592111	-1.348301
H	-1.982870	-0.004909	-2.044228
H	-2.589695	-1.533091	-2.781637
C	-3.893061	-2.407303	-0.640625
H	-4.763576	-2.373821	0.024217
H	-3.049012	-2.911225	-0.153344
H	-4.151703	-2.900962	-1.584605

[IB (Cl)]

N	-0.666941	-0.438057	0.163122
C	-1.244532	0.401075	-0.896253
O	0.607112	-0.928292	-0.148297
H	-2.070364	-0.145556	-1.382022
S	2.480591	-1.264297	-1.969400
C	0.825072	-0.984950	-1.530894
S	0.104562	0.599738	-2.116252
Cl	-0.370010	-2.356913	-2.159292
C	-1.488103	-1.379700	0.800823
C	-0.967846	-2.587364	1.294682
C	-2.844470	-1.079524	1.011294
C	-1.796411	-3.469247	1.982165
H	0.077598	-2.830445	1.115879
C	-3.657990	-1.976548	1.701945
H	-3.265277	-0.141071	0.648275
C	-3.144660	-3.175062	2.194694
H	-1.377923	-4.407391	2.352340
H	-4.709689	-1.726392	1.854351
H	-3.786185	-3.872762	2.734515
C	-1.732784	1.751167	-0.421500
C	-2.576916	2.490424	-1.258494
C	-1.350113	2.285101	0.811043
C	-3.018004	3.755226	-0.874365
H	-2.887511	2.070248	-2.218789
C	-1.797995	3.549239	1.197236
H	-0.714846	1.693165	1.471786
C	-2.627840	4.289160	0.355205
H	-3.673639	4.324529	-1.535647
H	-1.500009	3.956046	2.165420
H	-2.977000	5.277528	0.658993
N	3.632794	0.788048	1.309183
C	2.478296	1.490832	0.678022
H	1.629266	1.450256	1.369731
H	2.232834	0.975414	-0.258044
H	2.770478	2.530478	0.488059
C	4.028276	1.489899	2.558121

H	3.174679	1.485963	3.245667
H	4.310209	2.519189	2.308787
H	4.877616	0.960099	3.004569
C	3.247098	-0.618894	1.623406
H	2.979513	-1.116998	0.682088
H	2.386711	-0.598097	2.302162
H	4.102752	-1.107804	2.103596
C	4.773488	0.778137	0.350171
H	5.053426	1.815346	0.133068
H	4.437700	0.267444	-0.561316
H	5.612093	0.243857	0.811357

[IB (Br)]

N	0.008128	0.260754	0.266824
C	-0.993793	0.154959	-0.808076
O	0.642858	-1.028351	0.360776
H	-1.827548	-0.512819	-0.522644
S	1.806711	-2.892309	-1.021692
C	0.811546	-1.607053	-0.818763
S	-0.044009	-0.761941	-2.084284
Br	-2.365648	-2.999035	-0.065772
C	-0.556932	0.569734	1.554041
C	-1.334254	-0.357738	2.254628
C	-0.327105	1.849260	2.060351
C	-1.873343	0.013532	3.484904
H	-1.522747	-1.345567	1.815995
C	-0.888383	2.214276	3.284448
H	0.282675	2.549875	1.486596
C	-1.656008	1.294617	3.998632
H	-2.478958	-0.702784	4.043059
H	-0.719641	3.216270	3.682112
H	-2.089398	1.577020	4.959739
C	-1.461408	1.512844	-1.249651
C	-2.830072	1.789268	-1.259651
C	-0.549449	2.505634	-1.629834
C	-3.287555	3.048869	-1.651016
H	-3.535999	1.013013	-0.956731
C	-1.007226	3.762078	-2.016535
H	0.520695	2.285217	-1.623188
C	-2.378073	4.034956	-2.028294
H	-4.358400	3.258370	-1.657656
H	-0.294736	4.533410	-2.313961
H	-2.735249	5.019976	-2.333931
N	4.144338	0.565274	0.224224
C	3.187630	0.690702	-0.913097
H	2.218202	1.026305	-0.522271
H	3.085614	-0.294673	-1.382780
H	3.592072	1.412715	-1.631912
C	4.436039	1.913969	0.782481
H	3.490798	2.371829	1.095886
H	4.913664	2.518652	0.003292
H	5.106687	1.799364	1.641524

C	3.541685	-0.291786	1.286286
H	3.273066	-1.260172	0.845906
H	2.642732	0.208196	1.665588
H	4.278305	-0.418259	2.088214
C	5.406694	-0.058527	-0.261197
H	5.810295	0.557207	-1.072813
H	5.175937	-1.065946	-0.626194
H	6.116308	-0.105931	0.572311

[IA' (F)]

N	-0.342471	0.301959	0.354489
C	-0.683419	-0.552718	-0.786192
O	-0.442928	-0.475283	1.500546
H	-0.316924	-0.060620	-1.697172
S	2.085756	-1.294134	2.033357
C	0.475351	-1.555665	1.394944
S	0.385211	-2.003192	-0.454246
F	-0.197983	-2.550623	2.064732
C	-0.862979	1.586340	0.516971
C	-1.155102	2.378255	-0.607704
C	-1.033821	2.129074	1.801073
C	-1.598961	3.687411	-0.440761
H	-1.032523	1.976387	-1.613360
C	-1.480430	3.440310	1.946407
H	-0.803381	1.516237	2.670149
C	-1.767566	4.230757	0.833314
H	-1.821383	4.287233	-1.325474
H	-1.607028	3.846205	2.952074
H	-2.120865	5.255315	0.956125
C	-2.158438	-0.866869	-0.916290
C	-2.891804	-0.317166	-1.972161
C	-2.816064	-1.668817	0.027227
C	-4.263404	-0.554143	-2.084796
H	-2.384067	0.298998	-2.718505
C	-4.185642	-1.895557	-0.079838
H	-2.241393	-2.123044	0.835670
C	-4.913533	-1.339212	-1.135093
H	-4.822502	-0.122996	-2.917152
H	-4.688713	-2.519352	0.661245
H	-5.985748	-1.525688	-1.218679
N	4.004659	0.335271	-1.073270
C	4.785444	0.876428	-2.215855
H	4.331532	0.522810	-3.148431
H	5.817563	0.516373	-2.136169
H	4.760052	1.971443	-2.172024
C	4.605314	0.795571	0.211406
H	3.984744	0.404602	1.027645
H	4.609035	1.891707	0.217648
H	5.628917	0.409046	0.275350
C	2.595102	0.818561	-1.145659
H	2.603769	1.914302	-1.099401
H	2.044684	0.394229	-0.294864

H	2.164582	0.479764	-2.095770
C	4.014491	-1.156671	-1.114641
H	5.056984	-1.493731	-1.072926
H	3.546256	-1.477368	-2.052617
H	3.443668	-1.522699	-0.248729

[IA' (Cl)]

N	-0.324137	0.333189	0.394034
C	-0.614895	-0.252763	-0.916215
O	-0.470063	-0.676342	1.343513
H	-0.182166	0.405486	-1.681850
S	2.099840	-1.443326	1.702957
C	0.502145	-1.670213	1.044942
S	0.394331	-1.783279	-0.847849
Cl	-0.339026	-3.189778	1.714886
C	-0.855006	1.555450	0.806882
C	-1.092585	2.574565	-0.132733
C	-1.093185	1.807330	2.167755
C	-1.550603	3.819085	0.291660
H	-0.915914	2.399756	-1.193974
C	-1.552648	3.058771	2.571233
H	-0.905071	1.018839	2.893577
C	-1.786270	4.073797	1.643160
H	-1.729334	4.598474	-0.451684
H	-1.732355	3.237816	3.633206
H	-2.149795	5.049603	1.967631
C	-2.084328	-0.478119	-1.199012
C	-2.717779	0.274147	-2.192546
C	-2.834634	-1.401030	-0.457033
C	-4.082662	0.116686	-2.442368
H	-2.135613	0.988807	-2.780482
C	-4.197688	-1.549347	-0.699555
H	-2.337866	-2.010977	0.299914
C	-4.825802	-0.791988	-1.692016
H	-4.563678	0.706436	-3.224735
H	-4.774208	-2.269229	-0.115732
H	-5.893134	-0.917422	-1.883061
N	4.002288	0.674488	-1.088079
C	4.754721	1.411533	-2.136430
H	4.275245	1.225562	-3.104436
H	5.788468	1.047541	-2.148657
H	4.730533	2.481355	-1.899387
C	4.636621	0.898667	0.242003
H	4.034189	0.371162	0.991867
H	4.647069	1.976241	0.442534
H	5.659006	0.505009	0.211936
C	2.592929	1.161539	-1.037914
H	2.606135	2.231295	-0.796180
H	2.060893	0.590018	-0.264081
H	2.141404	0.999327	-2.024159
C	4.006407	-0.785802	-1.395028
H	5.048011	-1.125695	-1.431019

H	3.522087	-0.934549	-2.367050
H	3.447821	-1.299792	-0.599447

[IA’ (Br)]

N	-0.254428	0.554528	0.392791
C	-0.542796	0.309405	-1.019805
O	-0.461814	-0.641849	1.084713
H	-0.039244	1.090794	-1.605907
S	2.047694	-1.617155	1.252729
C	0.464150	-1.579090	0.560522
S	0.359127	-1.267418	-1.297536
Br	-0.592824	-3.334187	0.900687
C	-0.729311	1.670982	1.081350
C	-0.946083	2.883715	0.403477
C	-0.928438	1.615666	2.470891
C	-1.343261	4.014780	1.112621
H	-0.802239	2.949158	-0.675015
C	-1.328164	2.757433	3.160331
H	-0.756181	0.676731	2.993196
C	-1.539712	3.964321	2.492791
H	-1.507030	4.948088	0.570344
H	-1.478075	2.696638	4.240130
H	-1.856279	4.853115	3.040020
C	-2.010818	0.263865	-1.383746
C	-2.528772	1.211604	-2.270822
C	-2.870506	-0.692409	-0.825410
C	-3.886733	1.214413	-2.596497
H	-1.861193	1.954930	-2.714496
C	-4.226198	-0.682356	-1.142942
H	-2.465479	-1.451428	-0.152835
C	-4.738681	0.269897	-2.028374
H	-4.277052	1.957998	-3.293695
H	-4.888845	-1.429330	-0.702048
H	-5.801254	0.269510	-2.278022
N	4.105671	0.985567	-0.975365
C	4.912151	1.896058	-1.830291
H	4.461685	1.929849	-2.828836
H	5.935138	1.507119	-1.888942
H	4.911043	2.894657	-1.378701
C	4.711151	0.903479	0.384519
H	4.070311	0.257885	0.997789
H	4.756294	1.914632	0.804827
H	5.718592	0.482002	0.292243
C	2.712669	1.505599	-0.852399
H	2.753407	2.498583	-0.388694
H	2.140558	0.805323	-0.227660
H	2.283487	1.572984	-1.859435
C	4.069247	-0.378022	-1.581087
H	5.099551	-0.742781	-1.667028
H	3.606784	-0.303140	-2.571885
H	3.472146	-1.025520	-0.922940

[TS-IIA (F)]

N	-0.282202	0.238260	0.439099
C	-0.422353	-0.642771	-0.786111
O	-0.517854	-0.577485	1.550018
H	-0.103392	0.026583	-1.599701
S	2.022894	-0.413642	2.388226
C	0.585210	-1.120468	2.046624
S	0.686937	-2.054781	-0.649494
F	0.229184	-2.252595	2.615627
C	-1.077552	1.404290	0.513776
C	-0.939127	2.358760	-0.507625
C	-1.968979	1.650104	1.564519
C	-1.690457	3.528764	-0.477603
H	-0.232142	2.185029	-1.320333
C	-2.707694	2.834060	1.587290
H	-2.082242	0.918719	2.362432
C	-2.580540	3.776784	0.570073
H	-1.570877	4.259800	-1.279460
H	-3.397411	3.011777	2.414633
H	-3.164720	4.697726	0.592314
C	-1.882146	-1.001577	-1.007220
C	-2.644156	-0.312128	-1.956832
C	-2.506777	-1.989583	-0.233123
C	-3.999969	-0.599599	-2.134102
H	-2.169014	0.458109	-2.570062
C	-3.861405	-2.267314	-0.397194
H	-1.900386	-2.546448	0.483033
C	-4.613805	-1.574022	-1.350058
H	-4.576162	-0.057753	-2.886782
H	-4.334779	-3.037251	0.215627
H	-5.673639	-1.799588	-1.483114
N	3.995801	0.426077	-1.079282
C	4.795651	0.647676	-2.313589
H	4.381746	0.022785	-3.112789
H	5.836020	0.368018	-2.112100
H	4.731941	1.707438	-2.586018
C	4.534152	1.273582	0.019592
H	3.924523	1.103057	0.914231
H	4.470992	2.323688	-0.287578
H	5.576642	0.990377	0.203170
C	2.571310	0.796951	-1.330331
H	2.547656	1.847147	-1.646181
H	2.001520	0.640164	-0.404016
H	2.177623	0.133709	-2.108852
C	4.078593	-1.010540	-0.686552
H	5.128471	-1.245256	-0.474676
H	3.694389	-1.617098	-1.513061
H	3.441190	-1.170881	0.190853

[TS-IIA (Cl)]

N	0.287624	0.271687	-0.445590
C	0.422740	-0.153124	1.012621
O	0.498104	-0.893742	-1.213191

H	0.104466	0.744204	1.563433
S	-2.064300	-1.060401	-1.970738
C	-0.628039	-1.605837	-1.360263
S	-0.720423	-1.522852	1.309303
Cl	-0.133604	-3.282019	-1.602779
C	1.132992	1.308052	-0.901364
C	1.029236	2.558449	-0.269846
C	2.040918	1.144135	-1.953839
C	1.832378	3.617388	-0.679424
H	0.308407	2.699136	0.537389
C	2.831341	2.219607	-2.362461
H	2.123556	0.179634	-2.450958
C	2.739463	3.457020	-1.729649
H	1.739787	4.582498	-0.177777
H	3.533524	2.077743	-3.186260
H	3.363897	4.291298	-2.052221
C	1.869963	-0.450470	1.352681
C	2.640228	0.495501	2.037643
C	2.475583	-1.647664	0.946319
C	3.987858	0.254991	2.316033
H	2.177933	1.431545	2.361840
C	3.822512	-1.882944	1.210775
H	1.864553	-2.393170	0.433865
C	4.583659	-0.933275	1.898497
H	4.571688	1.000469	2.859538
H	4.282840	-2.818276	0.885948
H	5.637076	-1.124348	2.111998
N	-4.037767	0.963135	0.900638
C	-4.783665	1.634229	1.998748
H	-4.321991	1.354993	2.952587
H	-5.827467	1.301307	1.968712
H	-4.724235	2.718232	1.849402
C	-4.642825	1.327378	-0.410144
H	-4.062728	0.837875	-1.200824
H	-4.599309	2.416310	-0.525836
H	-5.682797	0.982106	-0.424481
C	-2.612468	1.407572	0.921769
H	-2.599152	2.495553	0.783177
H	-2.069668	0.892527	0.116847
H	-2.182260	1.125242	1.889348
C	-4.108158	-0.515748	1.084083
H	-5.163023	-0.812887	1.053704
H	-3.651419	-0.768022	2.046496
H	-3.532987	-0.992960	0.281373
	**[TS-IIA (Br)]**		
N	0.300494	-0.413278	0.400173
C	0.440323	-0.415395	-1.103717
O	0.536247	0.898183	0.832207
H	0.103520	-1.425978	-1.378672
S	-2.010997	1.215008	1.575595
C	-0.576057	1.627617	0.911565

S	-0.651952	0.828187	-1.810843
Br	-0.046524	3.464264	0.716458
C	1.052791	-1.326697	1.165829
C	0.986128	-2.691458	0.833811
C	1.824219	-0.930274	2.266211
C	1.684820	-3.629806	1.585945
H	0.376372	-3.018162	-0.009306
C	2.512112	-1.885146	3.015985
H	1.881840	0.122566	2.535338
C	2.453686	-3.236438	2.683584
H	1.620439	-4.684695	1.312305
H	3.106864	-1.557320	3.870814
H	2.997447	-3.976674	3.271978
C	1.898957	-0.273711	-1.502100
C	2.632062	-1.390590	-1.916644
C	2.547742	0.966846	-1.423916
C	3.984871	-1.277015	-2.248386
H	2.136330	-2.362301	-1.987244
C	3.899336	1.079228	-1.738940
H	1.961731	1.840363	-1.131543
C	4.623502	-0.042516	-2.154387
H	4.538913	-2.157769	-2.579174
H	4.392903	2.050879	-1.670344
H	5.680898	0.050130	-2.409691
N	-3.972253	-1.455982	-0.764609
C	-4.738181	-2.371877	-1.652157
H	-4.287720	-2.345171	-2.650695
H	-5.778349	-2.029380	-1.694978
H	-4.687936	-3.385637	-1.238801
C	-4.557420	-1.482112	0.604110
H	-3.967698	-0.812810	1.240860
H	-4.508311	-2.507999	0.986331
H	-5.598092	-1.143772	0.547869
C	-2.547659	-1.898660	-0.697918
H	-2.531168	-2.926870	-0.316438
H	-1.998464	-1.213806	-0.036774
H	-2.126420	-1.838930	-1.707874
C	-4.041765	-0.068088	-1.306849
H	-5.093393	0.241862	-1.316802
H	-3.616678	-0.068625	-2.315866
H	-3.431977	0.585643	-0.672137

[IIA (F)]

N	-0.269853	0.219806	0.404367
C	-0.433508	-0.726606	-0.769850
O	-0.531821	-0.516918	1.565535
H	-0.123168	-0.091796	-1.614740
S	1.966583	-0.118779	2.440359
C	0.540770	-0.876280	2.236842
S	0.653032	-2.152830	-0.604099
F	0.202928	-1.919881	2.958566
C	-1.027122	1.414804	0.404617

C	-0.826440	2.311358	-0.657665
C	-1.936897	1.742578	1.416138
C	-1.536462	3.506256	-0.707389
H	-0.104171	2.070506	-1.439059
C	-2.633104	2.951109	1.359621
H	-2.100758	1.055406	2.244328
C	-2.444831	3.836510	0.301139
H	-1.369256	4.192218	-1.539935
H	-3.338230	3.193560	2.156971
H	-2.996020	4.777018	0.261857
C	-1.903735	-1.067400	-0.955240
C	-2.672571	-0.395558	-1.912224
C	-2.529859	-2.020460	-0.139764
C	-4.035679	-0.667095	-2.056810
H	-2.197205	0.348297	-2.557113
C	-3.891105	-2.282428	-0.271630
H	-1.916604	-2.564279	0.580672
C	-4.650114	-1.607335	-1.232468
H	-4.616958	-0.139677	-2.815874
H	-4.365137	-3.025749	0.372836
H	-5.715407	-1.820494	-1.340219
N	3.981625	0.268128	-1.111474
C	4.770129	0.350682	-2.370377
H	4.322980	-0.329879	-3.103505
H	5.803501	0.054710	-2.156742
H	4.737583	1.382298	-2.738821
C	4.556108	1.197885	-0.101368
H	3.959965	1.120270	0.814766
H	4.507871	2.218753	-0.496906
H	5.596107	0.910319	0.089766
C	2.563869	0.652666	-1.383050
H	2.564346	1.669417	-1.794600
H	1.999926	0.596936	-0.441782
H	2.143576	-0.072189	-2.089223
C	4.031557	-1.127782	-0.587819
H	5.074883	-1.365206	-0.348344
H	3.634369	-1.798732	-1.356121
H	3.383729	-1.194660	0.293782

[IIA (Cl)]

N	-0.258419	0.124928	0.316997
C	-0.459882	-0.129023	-1.158059
O	-0.600991	-1.054087	0.993670
H	-0.121815	0.821022	-1.599593
S	1.839096	-1.119838	2.063946
C	0.395480	-1.679608	1.585432
S	0.560046	-1.497996	-1.734347
Cl	-0.172160	-3.281235	1.966637
C	-0.881463	1.236285	0.927320
C	-0.672002	2.509784	0.369343
C	-1.650157	1.122118	2.093354
C	-1.232135	3.635371	0.964774

H	-0.060898	2.619471	-0.527090
C	-2.196490	2.262114	2.684102
H	-1.823582	0.144278	2.539532
C	-1.998053	3.522971	2.127121
H	-1.059422	4.615281	0.515626
H	-2.791769	2.152495	3.592652
H	-2.431664	4.409249	2.592176
C	-1.943244	-0.275180	-1.460776
C	-2.671633	0.800432	-1.980466
C	-2.617850	-1.474207	-1.189402
C	-4.042353	0.685617	-2.228819
H	-2.158105	1.740137	-2.200721
C	-3.985866	-1.586450	-1.421648
H	-2.035369	-2.317775	-0.815143
C	-4.704452	-0.506595	-1.944691
H	-4.591814	1.532711	-2.644496
H	-4.497825	-2.525971	-1.202907
H	-5.775443	-0.599738	-2.134975
N	3.966546	0.742142	-1.005335
C	4.804349	1.329647	-2.085084
H	4.356450	1.069542	-3.050812
H	5.815761	0.914327	-2.009764
H	4.828845	2.417577	-1.956068
C	4.521557	1.127368	0.320261
H	3.902683	0.667364	1.098935
H	4.488420	2.219248	0.409159
H	5.554713	0.768127	0.387413
C	2.569297	1.256796	-1.119000
H	2.609697	2.351835	-1.074419
H	1.978471	0.838085	-0.292598
H	2.148486	0.898295	-2.065346
C	3.964930	-0.744293	-1.132887
H	4.986639	-1.102622	-0.959969
H	3.614074	-1.004266	-2.136703
H	3.254760	-1.157997	-0.407550

[IIA (Br)]

N	0.208884	-0.349155	0.272209
C	0.400820	-0.693476	-1.186476
O	0.614572	0.986192	0.439989
H	0.028612	-1.728806	-1.223453
S	-1.785470	1.564286	1.444122
C	-0.331859	1.828580	0.787707
S	-0.581776	0.377649	-2.248730
Br	0.393351	3.581135	0.546072
C	0.805894	-1.164551	1.260962
C	0.523325	-2.541795	1.246391
C	1.621001	-0.647122	2.276079
C	1.058168	-3.376061	2.222578
H	-0.122829	-2.957736	0.472384
C	2.141146	-1.495605	3.254309
H	1.851630	0.416624	2.302826

C	1.870454	-2.861442	3.235483
H	0.828974	-4.442997	2.193598
H	2.773897	-1.073579	4.037467
H	2.283900	-3.519275	4.001047
C	1.885944	-0.726520	-1.516030
C	2.582356	-1.940026	-1.540347
C	2.593959	0.458855	-1.758870
C	3.954620	-1.974386	-1.803606
H	2.042254	-2.872807	-1.357499
C	3.963492	0.428952	-2.007282
H	2.033555	1.395362	-1.771108
C	4.650126	-0.789144	-2.032102
H	4.479129	-2.931738	-1.828863
H	4.501856	1.360922	-2.192436
H	5.722469	-0.811461	-2.235902
N	-4.071953	-1.203641	-0.667618
C	-4.934281	-2.149089	-1.427070
H	-4.471837	-2.331447	-2.403577
H	-5.924479	-1.696365	-1.552992
H	-5.012139	-3.084358	-0.861102
C	-4.663598	-0.962588	0.676110
H	-4.013608	-0.265932	1.217260
H	-4.721045	-1.918151	1.209825
H	-5.664424	-0.534356	0.548140
C	-2.706993	-1.788744	-0.506002
H	-2.811415	-2.745360	0.020252
H	-2.087723	-1.079287	0.060083
H	-2.270885	-1.914751	-1.503585
C	-3.978569	0.086815	-1.409688
H	-4.982259	0.525053	-1.459769
H	-3.576874	-0.116427	-2.407309
H	-3.269231	0.740319	-0.889009

[IIA’ (F)]

N	-0.311125	0.483091	0.347742
C	-0.243000	-0.946498	-0.110032
O	-0.781425	0.487667	1.663151
H	0.220967	-0.859810	-1.103851
S	1.462688	1.683986	2.489316
C	0.117570	0.789373	2.579346
S	0.815049	-1.928282	0.981758
F	-0.354780	0.334869	3.718362
C	-1.639671	-1.517957	-0.291979
C	-2.200009	-1.631137	-1.568728
C	-2.404746	-1.912537	0.815249
C	-3.495291	-2.126882	-1.742005
H	-1.612560	-1.335815	-2.441793
C	-3.700526	-2.392336	0.646397
H	-1.949773	-1.856536	1.805435
C	-4.251394	-2.502761	-0.634222
H	-3.911984	-2.217363	-2.747127
H	-4.284869	-2.693128	1.518434

H	-5.264607	-2.887602	-0.764940
C	-0.980914	1.453391	-0.425153
C	-1.820778	2.414432	0.156181
C	-0.751717	1.504274	-1.811961
C	-2.412375	3.399217	-0.634690
H	-2.013014	2.395814	1.227300
C	-1.353228	2.490657	-2.587337
H	-0.094150	0.778213	-2.288491
C	-2.188860	3.447158	-2.008610
H	-3.062381	4.136582	-0.159721
H	-1.160288	2.509718	-3.661673
H	-2.657252	4.218266	-2.621458
N	3.840601	-0.540623	-1.118345
C	4.426901	-1.290805	0.029248
H	5.071967	-0.608778	0.594937
H	5.010454	-2.131029	-0.364771
H	3.592986	-1.646161	0.648948
C	4.924802	-0.001458	-1.979105
H	5.530509	-0.838290	-2.345691
H	5.540271	0.680702	-1.381522
H	4.470598	0.535848	-2.819599
C	2.976764	-1.464137	-1.908976
H	2.232017	-1.893701	-1.221439
H	3.610709	-2.251184	-2.333627
H	2.501319	-0.889418	-2.712504
C	3.007368	0.577858	-0.584327
H	3.660074	1.247076	-0.011281
H	2.232649	0.146892	0.066504
H	2.565262	1.116904	-1.431262

[IIA’ (Cl)]

N	0.258783	0.225392	0.388652
C	0.181906	-0.529932	-0.908713
O	0.817806	1.480961	0.110521
H	-0.308247	-1.467373	-0.604661
S	-1.276224	2.626047	1.289472
C	0.045385	2.520141	0.367348
S	-0.833248	0.343769	-2.122964
Cl	0.795186	3.895615	-0.400706
C	1.577128	-0.875170	-1.405173
C	2.100817	-2.158341	-1.215174
C	2.379532	0.093157	-2.026135
C	3.394789	-2.474648	-1.638672
H	1.485692	-2.924023	-0.734976
C	3.673705	-0.215333	-2.435432
H	1.954744	1.083439	-2.197832
C	4.186740	-1.502342	-2.244951
H	3.782635	-3.484608	-1.491288
H	4.287293	0.549924	-2.915421
H	5.198865	-1.744602	-2.574740
C	0.907683	-0.363681	1.497547
C	1.890167	0.309600	2.236084

C	0.514586	-1.651370	1.902609
C	2.463264	-0.297301	3.354102
H	2.211985	1.305936	1.937543
C	1.102359	-2.246844	3.014014
H	-0.261039	-2.184263	1.352047
C	2.080880	-1.576311	3.750593
H	3.226847	0.244577	3.915468
H	0.783408	-3.247768	3.310935
H	2.536189	-2.046154	4.623320
N	-4.006617	-1.109082	-0.309818
C	-4.515687	-0.209317	-1.383950
H	-5.136076	0.566811	-0.921418
H	-5.109977	-0.806004	-2.085628
H	-3.642706	0.231409	-1.883046
C	-5.145480	-1.711272	0.429418
H	-5.766592	-2.272000	-0.278453
H	-5.728591	-0.906240	0.891275
H	-4.748257	-2.381773	1.200085
C	-3.178070	-2.181717	-0.932507
H	-2.386415	-1.690130	-1.519227
H	-3.824259	-2.784642	-1.581472
H	-2.761371	-2.804553	-0.131953
C	-3.156035	-0.308563	0.621631
H	-3.780106	0.477701	1.062204
H	-2.333565	0.129386	0.036290
H	-2.776704	-0.973956	1.406986

[IIA’ (Br)]

N	0.123743	-0.163218	0.411594
C	-0.129856	-0.761343	-0.941733
O	1.056095	0.874406	0.253726
H	-0.905515	-1.510729	-0.723654
S	-0.666718	2.539872	1.413114
C	0.620258	2.081287	0.560246
S	-0.764111	0.469455	-2.103151
Br	1.937305	3.302187	-0.103525
C	1.103053	-1.500067	-1.436248
C	1.179620	-2.894057	-1.348088
C	2.199025	-0.797524	-1.957440
C	2.323038	-3.577294	-1.771743
H	0.330317	-3.455381	-0.949754
C	3.344429	-1.474749	-2.366611
H	2.118801	0.286531	-2.054241
C	3.410692	-2.868808	-2.276996
H	2.361025	-4.666424	-1.704460
H	4.191117	-0.914238	-2.768416
H	4.306681	-3.398425	-2.606465
C	0.484202	-0.988232	1.497730
C	1.523717	-0.650633	2.376656
C	-0.262392	-2.154382	1.746982
C	1.802968	-1.461631	3.476643
H	2.118672	0.244280	2.201352

C	0.033609	-2.956092	2.844924
H	-1.084255	-2.432262	1.087156
C	1.067262	-2.618854	3.720395
H	2.616861	-1.178624	4.146918
H	-0.558435	-3.856734	3.018298
H	1.293770	-3.250040	4.580567
N	-4.268213	-0.230366	-0.442756
C	-4.504588	0.895759	-1.390939
H	-4.892189	1.750511	-0.825014
H	-5.233396	0.571565	-2.142537
H	-3.540324	1.139175	-1.856423
C	-5.535919	-0.598479	0.239443
H	-6.267688	-0.903514	-0.517202
H	-5.902001	0.274007	0.792665
H	-5.334061	-1.426642	0.928662
C	-3.743030	-1.399646	-1.205778
H	-2.837782	-1.069435	-1.739266
H	-4.514036	-1.723790	-1.914763
H	-3.522747	-2.205525	-0.495601
C	-3.253727	0.200190	0.565471
H	-3.664337	1.053245	1.118356
H	-2.338498	0.489610	0.027908
H	-3.066042	-0.635785	1.250725

[TS-IIIA (F)]

N	-0.216147	0.146965	-0.727313
C	-0.534357	-1.238106	-0.849858
O	0.182544	0.178373	1.188910
H	-0.619457	-1.156091	-1.966997
S	1.081962	2.678229	1.165007
C	0.362798	1.281192	1.718930
S	0.806827	-2.402743	-0.621125
F	-0.136582	1.323152	2.981046
C	-1.865135	-1.722664	-0.313438
C	-2.980391	-1.817019	-1.149049
C	-1.993455	-2.058696	1.040702
C	-4.216546	-2.222980	-0.639834
H	-2.885207	-1.559010	-2.207314
C	-3.227519	-2.451774	1.550711
H	-1.110019	-1.998199	1.677352
C	-4.344886	-2.532496	0.712525
H	-5.080694	-2.293027	-1.303274
H	-3.321654	-2.703041	2.609102
H	-5.310236	-2.845249	1.114805
C	-1.153730	1.166956	-0.803597
C	-2.389578	1.222355	-0.116988
C	-0.755416	2.262036	-1.595534
C	-3.214240	2.325001	-0.284763
H	-2.672776	0.431738	0.574932
C	-1.610459	3.341514	-1.793413
H	0.228155	2.220210	-2.063543
C	-2.840090	3.371186	-1.137804

H	-4.159891	2.376449	0.256577
H	-1.306752	4.170046	-2.434406
H	-3.506543	4.225717	-1.268772
N	4.318863	-0.585858	-0.303418
C	4.195481	-1.704594	0.673523
H	4.399780	-1.310548	1.675819
H	4.925155	-2.479907	0.412362
H	3.169106	-2.089825	0.614477
C	5.673129	0.017092	-0.208340
H	6.418591	-0.755695	-0.429032
H	5.814366	0.402926	0.807642
H	5.744468	0.833320	-0.936509
C	4.091744	-1.103323	-1.682536
H	3.079574	-1.530986	-1.717072
H	4.849376	-1.866057	-1.897569
H	4.185611	-0.265663	-2.383468
C	3.280232	0.443195	0.010117
H	3.453161	0.811752	1.027242
H	2.290358	-0.028157	-0.070230
H	3.379270	1.265160	-0.707937

[TS-IIIA (Cl)]

N	0.074206	0.023373	0.355635
C	0.349672	-1.316302	-0.094958
O	0.500726	0.986054	-1.246295
H	-0.021431	-1.781426	0.853998
S	-1.409327	2.730839	-0.623121
C	-0.154224	2.022016	-1.426616
S	-0.735594	-1.908994	-1.414338
Cl	0.504841	2.887085	-2.897840
C	1.799617	-1.718393	-0.272562
C	2.491290	-2.365686	0.754110
C	2.473210	-1.411923	-1.462649
C	3.842937	-2.688417	0.607988
H	1.970743	-2.609817	1.684122
C	3.822910	-1.721651	-1.604029
H	1.916032	-0.926275	-2.264968
C	4.514055	-2.358722	-0.567578
H	4.371369	-3.195320	1.417708
H	4.342397	-1.470585	-2.531019
H	5.571399	-2.604071	-0.683485
C	0.880611	0.711926	1.269285
C	2.281887	0.858563	1.196883
C	0.172153	1.325396	2.318201
C	2.946725	1.564228	2.191561
H	2.834345	0.458895	0.348902
C	0.851590	1.996955	3.329751
H	-0.914782	1.242777	2.320007
C	2.239735	2.116197	3.265374
H	4.028557	1.690272	2.129302
H	0.297732	2.447043	4.154652
H	2.776328	2.659471	4.045338

N	-4.124429	-1.122523	0.489881
C	-4.493165	-1.827503	-0.769726
H	-5.162970	-1.179586	-1.346620
H	-4.998349	-2.765219	-0.510523
H	-3.566044	-2.022395	-1.323517
C	-5.348129	-0.807173	1.269970
H	-5.875152	-1.741747	1.493944
H	-5.983892	-0.146552	0.669374
H	-5.052051	-0.304677	2.197957
C	-3.220606	-1.998347	1.289193
H	-2.364802	-2.259510	0.649068
H	-3.777164	-2.897062	1.580755
H	-2.898239	-1.444589	2.178943
C	-3.401386	0.140626	0.146195
H	-4.060969	0.756349	-0.476292
H	-2.482125	-0.124452	-0.394536
H	-3.162034	0.663375	1.078933

[TS-IIIA (Br)]

N	0.535025	0.249235	-1.215655
C	0.960147	1.577192	-0.912549
O	-0.247812	-0.165367	0.508783
H	1.263825	1.760138	-1.979782
S	-1.323514	-2.469696	-0.277847
C	-0.811116	-1.235487	0.680657
S	-0.323452	2.791001	-0.629869
Br	-1.169718	-1.437720	2.713194
C	2.183479	1.750957	-0.035291
C	3.456540	1.869225	-0.597638
C	2.046722	1.758770	1.359110
C	4.585304	1.976380	0.218680
H	3.569040	1.864647	-1.685119
C	3.172561	1.852919	2.172451
H	1.045794	1.683339	1.786517
C	4.446666	1.959239	1.605007
H	5.574984	2.069211	-0.232580
H	3.058313	1.847993	3.258289
H	5.327115	2.036672	2.245589
C	1.416345	-0.818021	-1.380628
C	2.435867	-1.197823	-0.481472
C	1.190690	-1.586592	-2.538385
C	3.234263	-2.294353	-0.778668
H	2.565721	-0.666802	0.459798
C	2.026104	-2.653086	-2.849719
H	0.358975	-1.306230	-3.185755
C	3.047432	-3.007914	-1.967578
H	4.010500	-2.600292	-0.075956
H	1.864746	-3.224424	-3.764762
H	3.690508	-3.860191	-2.194443
N	-3.893430	1.055772	-0.874644
C	-3.318554	0.834909	0.485360
H	-3.266665	-0.246817	0.659143

H	-3.982324	1.304880	1.220870
H	-2.315994	1.286496	0.507490
C	-5.219801	0.392488	-0.968074
H	-5.879337	0.815648	-0.201504
H	-5.082971	-0.682174	-0.800469
H	-5.633405	0.572169	-1.967036
C	-4.040372	2.517451	-1.118633
H	-3.041214	2.967264	-1.067971
H	-4.699600	2.936619	-0.349897
H	-4.475597	2.659972	-2.114587
C	-2.965786	0.484752	-1.895403
H	-2.840756	-0.585504	-1.692869
H	-2.001000	1.002900	-1.798223
H	-3.406692	0.645658	-2.886587

[IIIA (F)]

N	0.383776	1.128236	1.167228
C	0.977659	0.063975	1.529635
O	-0.699744	-1.632318	-0.252885
S	-2.380443	-2.511537	-2.121180
C	-0.943520	-2.003878	-1.374957
S	0.275107	-0.761125	2.935703
F	0.157040	-2.012118	-2.218060
C	2.231996	-0.509410	0.954982
C	3.355536	0.316265	0.824248
C	2.299606	-1.849617	0.557597
C	4.539240	-0.197469	0.299104
H	3.299709	1.360743	1.137786
C	3.483298	-2.353813	0.021687
H	1.410287	-2.475836	0.638277
C	4.603271	-1.531286	-0.106563
H	5.414442	0.447430	0.204996
H	3.528496	-3.394684	-0.302850
H	5.529078	-1.930880	-0.524155
C	0.733552	1.840489	0.004376
C	0.885848	1.208232	-1.240328
C	0.835522	3.235823	0.085958
C	1.159468	1.969137	-2.375054
H	0.777341	0.124128	-1.309312
C	1.127576	3.985532	-1.051960
H	0.690806	3.717959	1.054567
C	1.289832	3.356515	-2.287454
H	1.270946	1.467609	-3.338305
H	1.220411	5.070437	-0.973497
H	1.508869	3.945358	-3.179611
N	-3.497748	0.556312	0.682290
C	-3.272971	-0.839499	1.161471
H	-3.421727	-1.524010	0.315887
H	-3.978989	-1.039222	1.976037
H	-2.234815	-0.926199	1.500380
C	-4.921183	0.723477	0.285564
H	-5.554480	0.550978	1.163515

H	-5.149680	-0.007938	-0.498077
H	-5.061588	1.742822	-0.092031
C	-3.156350	1.512893	1.770628
H	-2.084131	1.415802	1.985174
H	-3.756594	1.267902	2.654686
H	-3.381209	2.529288	1.426620
C	-2.618566	0.820915	-0.493381
H	-2.888243	0.120769	-1.293972
H	-1.585547	0.641254	-0.182012
H	-2.761846	1.861938	-0.807011
H	1.217342	-1.716970	3.056214

[IIIA (Cl)]

N	0.253998	-1.505987	-0.935135
C	0.917828	-0.622207	-1.563731
O	-0.639586	1.549523	-0.207950
S	-2.318458	3.044213	1.216716
C	-0.920148	2.289979	0.686229
S	0.251378	-0.134920	-3.135613
Cl	0.642942	2.706766	1.785742
C	2.218410	-0.010938	-1.156339
C	3.296335	-0.851363	-0.850106
C	2.368690	1.377405	-1.076748
C	4.518699	-0.302454	-0.470629
H	3.173006	-1.934631	-0.912546
C	3.590295	1.919630	-0.681130
H	1.514547	2.023041	-1.284871
C	4.665628	1.083231	-0.381599
H	5.359038	-0.959106	-0.239492
H	3.696480	3.002306	-0.595490
H	5.621544	1.511503	-0.074920
C	0.576325	-1.912807	0.374582
C	0.825984	-0.988455	1.401627
C	0.540167	-3.281459	0.672874
C	1.054837	-1.438531	2.699693
H	0.824867	0.080897	1.180395
C	0.790083	-3.722629	1.971341
H	0.321472	-3.989717	-0.128562
C	1.046306	-2.804216	2.990936
H	1.238803	-0.706589	3.488830
H	0.774633	-4.792211	2.189445
H	1.230131	-3.150722	4.009206
N	-3.551745	-0.714084	-0.541643
C	-3.214811	0.436988	-1.428461
H	-3.342870	1.365541	-0.857128
H	-3.879480	0.405069	-2.299691
H	-2.164815	0.346531	-1.728109
C	-4.991328	-0.653237	-0.173933
H	-5.591508	-0.726084	-1.088181
H	-5.178803	0.302262	0.329861
H	-5.217055	-1.488787	0.498386
C	-3.259326	-1.986740	-1.257971

H	-2.181912	-2.017188	-1.467703
H	-3.838390	-2.005842	-2.188551
H	-3.546554	-2.825227	-0.612973
C	-2.717892	-0.644346	0.693271
H	-2.955516	0.286875	1.222177
H	-1.667632	-0.637835	0.387310
H	-2.941530	-1.520755	1.313291
H	1.259249	0.684190	-3.494326

[IIIA (Br)]

N	-1.093724	-1.639176	-1.084964
C	0.025717	-1.365781	-1.622752
O	0.207539	1.461239	-0.312265
S	0.056614	2.112867	2.264824
C	0.604700	1.906013	0.717849
S	-0.053948	-0.581401	-3.215537
Br	2.658400	2.677575	0.433420
C	1.383144	-1.682698	-1.085329
C	1.665906	-2.991331	-0.672451
C	2.360694	-0.688097	-0.973219
C	2.919819	-3.299362	-0.150798
H	0.901032	-3.765422	-0.762192
C	3.605880	-0.998274	-0.428606
H	2.135680	0.338504	-1.268742
C	3.888910	-2.301975	-0.021443
H	3.139465	-4.321355	0.162456
H	4.348029	-0.206661	-0.312734
H	4.866106	-2.542983	0.400916
C	-1.205304	-2.157212	0.218913
C	-0.569576	-1.558376	1.317486
C	-2.060550	-3.247079	0.431768
C	-0.780376	-2.054570	2.601573
H	0.071084	-0.688696	1.161416
C	-2.247573	-3.752175	1.717527
H	-2.568286	-3.691973	-0.426062
C	-1.611501	-3.157409	2.808498
H	-0.289508	-1.567054	3.446140
H	-2.904707	-4.610781	1.868482
H	-1.769213	-3.545762	3.815932
N	-3.358647	1.649230	-0.551483
C	-2.898128	3.004128	-0.137048
H	-3.422328	3.283852	0.783842
H	-3.129376	3.711922	-0.941298
H	-1.818257	2.960755	0.044530
C	-4.831771	1.658720	-0.755010
H	-5.074645	2.393257	-1.531108
H	-5.313527	1.930578	0.191242
H	-5.145482	0.656158	-1.067850
C	-2.674089	1.262749	-1.819894
H	-1.592739	1.287135	-1.642729
H	-2.959772	1.978190	-2.599934
H	-2.986928	0.246690	-2.087229

C	-3.004586	0.665406	0.512088
H	-3.498011	0.967586	1.443390
H	-1.916129	0.677900	0.639543
H	-3.349126	-0.325905	0.194991
H	1.268360	-0.600876	-3.472966

[IB’ (F)]

N	1.476412	0.185257	-0.209700
C	0.789341	0.821764	0.893292
O	0.800912	-0.959414	-0.611725
H	1.534543	1.253337	1.574894
S	-0.528854	-3.124114	0.147359
C	0.532346	-1.783830	0.516057
S	-0.078891	-0.560867	1.782471
F	1.773483	-2.198432	0.974634
C	2.871752	0.155330	-0.325274
C	3.624253	1.267588	0.087944
C	3.527107	-0.923837	-0.940156
C	5.007964	1.275420	-0.076524
H	3.127913	2.138836	0.517650
C	4.908895	-0.894107	-1.102900
H	2.945668	-1.782379	-1.270096
C	5.663812	0.197378	-0.669561
H	5.575074	2.146927	0.256406
H	5.402296	-1.746464	-1.574348
H	6.746774	0.209698	-0.798899
C	-0.194086	1.890003	0.455739
C	-0.259411	2.310219	-0.874043
C	-1.060402	2.462446	1.396063
C	-1.191585	3.276039	-1.263353
H	0.416801	1.866185	-1.606178
C	-1.988821	3.425595	1.008476
H	-1.016187	2.141352	2.440326
C	-2.061390	3.832001	-0.327422
H	-1.236926	3.591723	-2.307432
H	-2.659248	3.862503	1.750969
H	-2.790330	4.584674	-0.632889
N	-3.774287	-0.859986	-0.566450
C	-5.029013	-0.090598	-0.771319
H	-5.001396	0.799185	-0.131448
H	-5.879187	-0.727351	-0.500915
H	-5.092882	0.202425	-1.825609
C	-3.779155	-2.078172	-1.425035
H	-2.824581	-2.597555	-1.269180
H	-3.886718	-1.764143	-2.469804
H	-4.623610	-2.710453	-1.127531
C	-2.602286	-0.009957	-0.927694
H	-2.694267	0.275496	-1.982637
H	-1.686182	-0.592608	-0.759939
H	-2.617741	0.880465	-0.288433
C	-3.659600	-1.267572	0.863909
H	-4.541359	-1.866664	1.120603

H	-3.619725	-0.360706	1.478835
H	-2.736666	-1.854748	0.974330

[IB' (Cl)]

N	-1.069412	-0.186295	0.291398
C	-0.862490	0.644565	-0.900229
O	-0.217369	-1.292658	0.357499
H	-1.741872	0.558280	-1.560402
S	1.487947	-2.866639	-0.882738
C	0.199358	-1.705817	-0.915881
S	0.572396	-0.111451	-1.752665
Cl	-1.393069	-2.366088	-1.763829
C	-2.375828	-0.447789	0.723975
C	-3.365840	0.535423	0.558541
C	-2.700265	-1.646156	1.381165
C	-4.655916	0.312471	1.037972
H	-3.128833	1.480742	0.068545
C	-3.993205	-1.848357	1.853659
H	-1.937013	-2.412826	1.498188
C	-4.982101	-0.876043	1.688854
H	-5.412451	1.087268	0.898942
H	-4.230942	-2.788604	2.355402
H	-5.993027	-1.044165	2.062413
C	-0.587826	2.099381	-0.592009
C	-0.155268	2.511252	0.671352
C	-0.747332	3.048337	-1.608049
C	0.124971	3.857097	0.911498
H	-0.055822	1.769697	1.466567
C	-0.461888	4.391499	-1.367995
H	-1.097052	2.729821	-2.593661
C	-0.023100	4.799153	-0.107025
H	0.454755	4.172615	1.903255
H	-0.588473	5.123952	-2.167237
H	0.194510	5.851538	0.083378
N	3.981563	-0.254131	1.006530
C	4.993566	0.646358	1.618897
H	4.864920	1.652147	1.202790
H	5.992736	0.262124	1.384327
H	4.836256	0.661780	2.703366
C	4.136662	-1.633666	1.549767
H	3.361140	-2.262283	1.092389
H	4.013257	-1.592830	2.638013
H	5.137761	-1.998149	1.291991
C	2.611465	0.241667	1.324062
H	2.499949	0.269942	2.414601
H	1.880874	-0.445068	0.878486
H	2.502363	1.246870	0.900803
C	4.162637	-0.286287	-0.473999
H	5.181122	-0.630961	-0.688528
H	4.011697	0.726937	-0.863763
H	3.419134	-0.980167	-0.891170

[IB' (Br)]

N	-0.854125	0.175511	0.487563
C	-0.647502	0.801187	-0.822413
O	-0.191446	-1.046812	0.642974
H	-1.569934	0.717238	-1.421500
S	1.233907	-2.945574	-0.453148
C	0.195755	-1.609757	-0.548530
S	0.639441	-0.224416	-1.620688
Br	-1.876881	-2.223154	-1.417502
C	-2.134891	0.189026	1.060359
C	-2.979908	1.285768	0.826617
C	-2.559382	-0.836791	1.919718
C	-4.228419	1.345813	1.444591
H	-2.664343	2.099542	0.172753
C	-3.807927	-0.757106	2.527783
H	-1.913207	-1.696368	2.084557
C	-4.653185	0.330995	2.299722
H	-4.873062	2.205064	1.249616
H	-4.126935	-1.567016	3.186981
H	-5.630687	0.384131	2.780813
C	-0.197344	2.243288	-0.741987
C	0.385122	2.768766	0.414744
C	-0.351458	3.059656	-1.867618
C	0.818393	4.094856	0.439632
H	0.478912	2.135022	1.298889
C	0.088293	4.382415	-1.843104
H	-0.820430	2.653867	-2.767824
C	0.675833	4.902933	-0.688968
H	1.264037	4.501526	1.349434
H	-0.035377	5.011229	-2.726525
H	1.014042	5.940284	-0.666503
N	4.153913	-0.461334	1.004591
C	5.316200	0.352411	1.450846
H	5.311819	1.298992	0.898644
H	6.234808	-0.206987	1.240139
H	5.220469	0.538185	2.526767
C	4.132693	-1.757185	1.741363
H	3.256796	-2.324925	1.401385
H	4.064423	-1.545030	2.814394
H	5.058126	-2.299505	1.516280
C	2.889828	0.279315	1.282212
H	2.844080	0.493397	2.356650
H	2.043710	-0.349528	0.979944
H	2.901415	1.211110	0.705098
C	4.262519	-0.728789	-0.459239
H	5.204896	-1.258736	-0.641584
H	4.253338	0.230844	-0.989105
H	3.405728	-1.347678	-0.758592

[TS-IIB (F)]

N	-1.565702	-0.554041	-0.249538
C	-0.694232	0.236384	-1.163953
O	-1.421257	-1.920752	-0.431531

H	-1.302293	0.547935	-2.032116
S	0.427547	-2.066638	1.510392
C	-0.257539	-2.378543	0.044171
S	0.693033	-0.766418	-1.784095
F	-0.021896	-3.514934	-0.586936
C	-2.912120	-0.215336	-0.075656
C	-3.376563	1.071109	-0.401211
C	-3.811976	-1.143391	0.479597
C	-4.707620	1.412090	-0.163220
H	-2.705442	1.813475	-0.830313
C	-5.137762	-0.786121	0.702045
H	-3.460905	-2.140722	0.739400
C	-5.599381	0.492934	0.385704
H	-5.046656	2.417327	-0.420960
H	-5.818796	-1.522902	1.132633
H	-6.639655	0.767632	0.564838
C	-0.207002	1.479561	-0.441726
C	-0.198332	1.583751	0.952995
C	0.290277	2.546747	-1.201945
C	0.308454	2.728687	1.573212
H	-0.574238	0.748954	1.546625
C	0.799038	3.686601	-0.583499
H	0.289689	2.469174	-2.292268
C	0.810531	3.782947	0.811027
H	0.308166	2.795063	2.663327
H	1.183194	4.507830	-1.192080
H	1.202693	4.678090	1.297387
N	4.085801	-0.182935	0.193053
C	5.365676	0.033937	0.916364
H	5.531675	1.112947	1.015856
H	6.177601	-0.423808	0.339657
H	5.291754	-0.431810	1.905533
C	3.822720	-1.644553	0.055449
H	2.852463	-1.769286	-0.443497
H	3.787988	-2.085871	1.057748
H	4.633031	-2.090419	-0.533103
C	2.958059	0.440738	0.946338
H	2.934213	0.014398	1.956053
H	2.031690	0.206191	0.405553
H	3.127349	1.523854	0.988667
C	4.159640	0.438735	-1.160275
H	4.968038	-0.043555	-1.721938
H	4.364373	1.508601	-1.036995
H	3.189995	0.287390	-1.653200

[TS-IIB (Cl)]

N	-0.531216	-0.881836	0.102851
C	-0.807856	-0.561463	-1.362048
O	-0.167637	-2.245033	0.067598
H	-1.597952	-1.269831	-1.671173
S	2.413580	-1.854849	0.678991
C	1.133743	-2.444221	-0.184677

S	0.706884	-0.869257	-2.301101
Cl	1.255578	-4.019003	-0.973683
C	-1.671753	-0.772278	0.960281
C	-1.911155	0.467805	1.562542
C	-2.555702	-1.834306	1.182709
C	-3.037567	0.651632	2.360438
H	-1.212731	1.287193	1.385326
C	-3.672280	-1.646280	1.997791
H	-2.361350	-2.804595	0.726552
C	-3.922141	-0.405209	2.582946
H	-3.219385	1.625108	2.819274
H	-4.354567	-2.480310	2.172439
H	-4.799447	-0.263201	3.216105
C	-1.372169	0.840088	-1.475282
C	-0.537708	1.961377	-1.508002
C	-2.757538	1.032778	-1.506595
C	-1.072689	3.248045	-1.538052
H	0.539008	1.791157	-1.531345
C	-3.298771	2.318706	-1.549365
H	-3.419800	0.163391	-1.481808
C	-2.457700	3.431163	-1.555243
H	-0.407804	4.114661	-1.561490
H	-4.382069	2.450863	-1.574390
H	-2.878525	4.437946	-1.586528
N	3.013442	2.076020	0.942846
C	3.313524	3.526477	1.084955
H	2.626805	4.088943	0.441810
H	4.351138	3.700003	0.777789
H	3.172884	3.811150	2.134007
C	3.922182	1.297311	1.829460
H	3.689983	0.234362	1.709987
H	3.749844	1.610331	2.865267
H	4.957516	1.501419	1.534197
C	1.596252	1.817200	1.334439
H	1.477811	2.078878	2.392571
H	1.372177	0.754232	1.163088
H	0.950510	2.446666	0.711233
C	3.217951	1.659084	-0.476543
H	4.288722	1.717690	-0.704039
H	2.661726	2.349537	-1.120675
H	2.837055	0.636692	-0.611005

[TS-IIB (Br)]

N	-1.586600	-0.077791	-0.121786
C	-0.728080	0.649414	-1.103728
O	-1.390296	-1.454765	-0.158932
H	-1.354795	0.886250	-1.981780
S	0.460336	-1.285386	1.767306
C	-0.216321	-1.819846	0.372780
S	0.652588	-0.385199	-1.675059
Br	0.149293	-3.592730	-0.303385
C	-2.948271	0.222862	-0.013160

C	-3.435619	1.477042	-0.423601
C	-3.843538	-0.700939	0.556576
C	-4.783616	1.790142	-0.255632
H	-2.767371	2.216408	-0.862779
C	-5.186665	-0.371292	0.708789
H	-3.477342	-1.673965	0.879138
C	-5.670937	0.874811	0.307540
H	-5.139533	2.770473	-0.578451
H	-5.863639	-1.105009	1.150956
H	-6.724707	1.127402	0.431953
C	-0.246479	1.952817	-0.489918
C	-0.275147	2.195762	0.886830
C	0.284055	2.935436	-1.337431
C	0.232221	3.390465	1.405312
H	-0.679381	1.429633	1.549767
C	0.789623	4.126042	-0.821003
H	0.313737	2.748548	-2.413877
C	0.767838	4.358867	0.557428
H	0.204616	3.563712	2.483266
H	1.199685	4.879320	-1.496871
H	1.160222	5.292817	0.964137
N	4.051759	0.372962	0.212953
C	5.337128	0.633163	0.911977
H	5.531228	1.711840	0.894794
H	6.136333	0.094424	0.389941
H	5.251318	0.278358	1.945308
C	3.753422	-1.088744	0.230328
H	2.780248	-1.241939	-0.255959
H	3.708649	-1.420387	1.273668
H	4.553788	-1.611990	-0.305779
C	2.941341	1.102024	0.894078
H	2.900017	0.775665	1.939725
H	2.009713	0.844525	0.373127
H	3.144573	2.178410	0.833434
C	4.140681	0.844615	-1.198829
H	4.938910	0.287196	-1.702671
H	4.369802	1.916689	-1.189408
H	3.168345	0.662586	-1.675858

[IIB (F)]

N	-0.888335	-0.680074	-0.077710
C	-0.732003	0.489394	-1.012436
O	-0.081513	-1.720745	-0.609922
H	-1.190928	0.199957	-1.976386
S	1.307007	-1.774880	1.677122
C	0.959007	-2.035952	0.114844
S	0.998976	0.948005	-1.272297
F	1.741486	-2.769857	-0.656300
C	-2.231787	-1.186729	-0.007272
C	-2.795174	-1.945924	-1.038723
C	-2.989498	-0.861496	1.119060
C	-4.117382	-2.374950	-0.936518

H	-2.193670	-2.207996	-1.910671
C	-4.317674	-1.277035	1.205896
H	-2.522545	-0.282593	1.916934
C	-4.883159	-2.035245	0.180383
H	-4.554122	-2.973894	-1.737695
H	-4.908966	-1.016362	2.085356
H	-5.920192	-2.367300	0.253358
C	-1.548888	1.617309	-0.420358
C	-2.624564	2.166906	-1.120147
C	-1.236267	2.121782	0.849715
C	-3.371587	3.213257	-0.570423
H	-2.883230	1.769239	-2.104842
C	-1.986028	3.153905	1.404431
H	-0.392689	1.684924	1.388832
C	-3.056956	3.707262	0.693129
H	-4.206612	3.636860	-1.132065
H	-1.736885	3.534899	2.397080
H	-3.641944	4.520518	1.126884
N	4.610963	0.502638	0.043095
C	4.561891	1.603619	-0.960184
H	3.517044	1.752072	-1.260051
H	5.175611	1.317352	-1.822138
H	4.956698	2.513938	-0.495001
C	6.005072	0.329854	0.529237
H	6.321129	1.255143	1.024783
H	6.652561	0.117035	-0.329217
H	6.025102	-0.506771	1.236776
C	4.136376	-0.762405	-0.591508
H	4.108161	-1.545566	0.175297
H	4.837076	-1.030371	-1.391112
H	3.129812	-0.578325	-0.994450
C	3.715601	0.844542	1.187243
H	4.050174	1.798772	1.611197
H	3.791728	0.045534	1.933496
H	2.689419	0.921113	0.796580

[IIB (Cl)]

N	0.891879	0.588853	-0.073194
C	0.818426	-0.518860	-1.083776
O	0.094143	1.646702	-0.601600
H	1.298459	-0.150763	-2.010104
S	-1.271664	1.657316	1.683775
C	-0.931175	1.990403	0.136691
S	-0.882402	-1.024993	-1.438771
Cl	-1.929446	3.043548	-0.848049
C	2.214115	1.122804	0.115122
C	2.837571	1.921935	-0.849477
C	2.886446	0.789155	1.291702
C	4.135695	2.379907	-0.631393
H	2.299946	2.192178	-1.759900
C	4.192108	1.234708	1.495374
H	2.371958	0.179789	2.035890

C	4.817741	2.031291	0.536080
H	4.619630	3.009327	-1.380379
H	4.718010	0.967140	2.413481
H	5.836573	2.386537	0.699958
C	1.660806	-1.651935	-0.538821
C	2.766319	-2.124073	-1.248985
C	1.342058	-2.244491	0.691194
C	3.535526	-3.180018	-0.751105
H	3.029605	-1.658115	-2.201928
C	2.114077	-3.286260	1.195555
H	0.475873	-1.870897	1.242033
C	3.214131	-3.761768	0.472775
H	4.393269	-3.541995	-1.321551
H	1.859269	-3.736115	2.157472
H	3.816370	-4.582727	0.866556
N	-4.379958	-0.942704	0.236233
C	-4.304827	-2.047043	-0.762231
H	-3.270353	-2.114255	-1.122343
H	-4.992674	-1.822163	-1.585352
H	-4.595061	-2.980129	-0.265478
C	-5.740916	-0.896602	0.831122
H	-5.929414	-1.843285	1.350384
H	-6.470902	-0.752408	0.026522
H	-5.783457	-0.060414	1.538368
C	-4.078513	0.356559	-0.435267
H	-4.075704	1.145478	0.326796
H	-4.856983	0.547508	-1.183701
H	-3.087389	0.271794	-0.905987
C	-3.366617	-1.185716	1.304059
H	-3.567417	-2.165796	1.752878
H	-3.464147	-0.393459	2.055174
H	-2.374236	-1.161347	0.827784

[IIB (Br)]

N	-0.957280	-0.429013	-0.007371
C	-1.033951	0.581999	-1.115354
O	-0.029552	-1.419062	-0.462514
H	-1.445511	0.063372	-2.001753
S	1.248601	-1.188862	1.859501
C	0.985874	-1.634899	0.329844
S	0.579685	1.292898	-1.517771
Br	2.207995	-2.753450	-0.651748
C	-2.197006	-1.112031	0.250980
C	-2.710234	-2.079571	-0.619606
C	-2.908943	-0.748090	1.395078
C	-3.939569	-2.674599	-0.341089
H	-2.141171	-2.370378	-1.504204
C	-4.147125	-1.333010	1.657530
H	-2.478987	-0.003526	2.066351
C	-4.663516	-2.297367	0.791595
H	-4.337261	-3.434178	-1.016548
H	-4.705050	-1.041550	2.549056

H	-5.628825	-2.761001	1.002075
C	-2.043519	1.619778	-0.672179
C	-3.233407	1.806417	-1.378692
C	-1.803748	2.399123	0.467954
C	-4.166076	2.762125	-0.964651
H	-3.434919	1.193178	-2.260659
C	-2.736441	3.340904	0.890766
H	-0.869247	2.250736	1.013485
C	-3.922714	3.528721	0.172354
H	-5.089065	2.900178	-1.531357
H	-2.540890	3.937424	1.784354
H	-4.651882	4.271520	0.501420
N	3.933436	1.839888	0.358039
C	3.744193	2.858608	-0.714095
H	2.727743	2.748943	-1.113285
H	4.494436	2.688959	-1.494796
H	3.873683	3.852537	-0.270305
C	5.251511	2.040666	1.014521
H	5.266061	3.034763	1.475890
H	6.037065	1.962343	0.253902
H	5.382686	1.265097	1.777942
C	3.870974	0.471706	-0.236908
H	3.950106	-0.259486	0.576941
H	4.708640	0.361172	-0.935800
H	2.904106	0.371739	-0.754052
C	2.835868	1.985374	1.358372
H	2.848431	3.014976	1.735276
H	3.018036	1.277625	2.175136
H	1.889706	1.762991	0.841352

[IIB’ (F)]

N	1.447389	-0.562378	0.596243
C	0.592588	0.334542	1.393225
O	0.699391	-1.353344	-0.289038
H	1.254765	0.654320	2.211571
S	-0.723628	-3.504684	-0.586835
C	0.348158	-2.545645	0.170508
S	-0.800197	-0.573661	2.138882
F	1.083944	-2.957003	1.167674
C	0.140711	1.573366	0.636284
C	-0.367392	2.656715	1.368979
C	0.184297	1.674133	-0.757591
C	-0.828713	3.802578	0.728114
H	-0.416523	2.578832	2.457903
C	-0.283177	2.823015	-1.404651
H	0.581930	0.850010	-1.353504
C	-0.791741	3.890254	-0.667580
H	-1.217925	4.634730	1.318363
H	-0.240282	2.881620	-2.494395
H	-1.150238	4.788923	-1.172900
C	2.668944	-0.175324	0.035577
C	3.346151	0.958958	0.518994

C	3.267984	-0.943461	-0.978623
C	4.598013	1.293032	0.008217
H	2.898670	1.584873	1.289766
C	4.516585	-0.587136	-1.481745
H	2.757645	-1.822481	-1.370828
C	5.195565	0.529131	-0.994025
H	5.106630	2.175892	0.400294
H	4.962430	-1.199098	-2.268327
H	6.173893	0.802141	-1.391291
N	-3.667608	-0.067814	-0.616306
C	-4.787588	0.177693	-1.560911
H	-5.696128	0.381795	-0.982690
H	-4.924405	-0.714714	-2.182454
H	-4.533950	1.041213	-2.186743
C	-3.424743	1.148877	0.215028
H	-2.639586	0.900936	0.945517
H	-4.361202	1.414995	0.719478
H	-3.102221	1.961625	-0.448319
C	-3.994349	-1.215334	0.277972
H	-3.119439	-1.395290	0.917034
H	-4.208755	-2.091490	-0.345238
H	-4.871888	-0.950049	0.878880
C	-2.425506	-0.391168	-1.374083
H	-2.197446	0.451660	-2.036924
H	-2.597830	-1.305319	-1.953523
H	-1.620773	-0.545848	-0.642870
	**[IIB' (Cl)]**		
N	1.409287	-0.286768	0.479366
C	0.464424	0.439774	1.344830
O	0.768049	-1.125839	-0.431658
H	1.100910	0.793758	2.169666
S	-0.306736	-3.411179	-0.940860
C	0.666329	-2.414262	-0.110691
S	-0.788089	-0.672292	2.061563
Cl	1.804010	-2.975779	1.074334
C	-0.148350	1.655944	0.667803
C	-0.750075	2.639338	1.467181
C	-0.158416	1.828596	-0.719832
C	-1.352711	3.757080	0.897302
H	-0.757210	2.504092	2.551599
C	-0.767881	2.948268	-1.295729
H	0.310545	1.083643	-1.366031
C	-1.367197	3.916147	-0.492480
H	-1.812391	4.511846	1.538726
H	-0.763967	3.064213	-2.381757
H	-1.836565	4.793159	-0.942013
C	2.566357	0.283665	-0.056972
C	3.096984	1.467335	0.488007
C	3.252679	-0.345375	-1.111511
C	4.292092	1.986462	-0.003739
H	2.577515	1.987505	1.291482

C	4.442043	0.194665	-1.593868
H	2.856691	-1.259001	-1.553749
C	4.975957	1.360821	-1.045960
H	4.686179	2.904826	0.435798
H	4.957091	-0.311623	-2.412640
H	5.908504	1.778018	-1.427752
N	-3.767943	-0.305288	-0.577893
C	-4.895599	-0.098792	-1.522492
H	-5.816287	0.043242	-0.944845
H	-4.981771	-0.982206	-2.165595
H	-4.685432	0.791276	-2.126937
C	-3.599581	0.900622	0.286967
H	-2.798272	0.681137	1.008938
H	-4.550988	1.092355	0.797847
H	-3.328276	1.750443	-0.352713
C	-4.033378	-1.490141	0.286565
H	-3.156617	-1.628513	0.933049
H	-4.182696	-2.365089	-0.356934
H	-4.933147	-1.294208	0.881110
C	-2.505998	-0.541994	-1.336399
H	-2.321175	0.326190	-1.979483
H	-2.627713	-1.449244	-1.939044
H	-1.695783	-0.669855	-0.604593

[IIB’ (Br)]

N	1.256902	0.196045	0.338710
C	0.224918	0.643919	1.292622
O	0.754821	-0.680154	-0.621312
H	0.814947	1.047930	2.128806
S	0.090546	-3.075809	-1.288595
C	0.896733	-1.985082	-0.404330
S	-0.751820	-0.750339	1.937292
Br	2.307559	-2.470713	0.789307
C	-0.649048	1.762276	0.746286
C	-1.398943	2.530387	1.649739
C	-0.761546	2.043268	-0.618818
C	-2.244738	3.541104	1.202546
H	-1.325915	2.307155	2.716998
C	-1.614540	3.055216	-1.071724
H	-0.183557	1.466967	-1.344256
C	-2.359577	3.807475	-0.166042
H	-2.817366	4.127881	1.923800
H	-1.687614	3.258157	-2.142431
H	-3.020038	4.601467	-0.519423
C	2.236255	1.042678	-0.190036
C	2.548353	2.256458	0.448999
C	2.965373	0.670818	-1.333914
C	3.576851	3.057973	-0.040477
H	1.988772	2.580178	1.325258
C	3.984248	1.490788	-1.812175
H	2.736460	-0.261337	-1.849249
C	4.303618	2.687906	-1.171835

H	3.803013	3.994332	0.473346
H	4.536433	1.181294	-2.701760
H	5.103792	3.324818	-1.550781
N	-3.851370	-0.824726	-0.586837
C	-5.051055	-0.809297	-1.462523
H	-5.942799	-0.943155	-0.839490
H	-4.966651	-1.627733	-2.186864
H	-5.091951	0.155050	-1.981998
C	-3.922074	0.296608	0.397135
H	-3.048975	0.209867	1.062062
H	-4.858264	0.200951	0.960373
H	-3.902813	1.244366	-0.155896
C	-3.772202	-2.117753	0.150619
H	-2.850621	-2.100215	0.747535
H	-3.747054	-2.933478	-0.581620
H	-4.655304	-2.207866	0.793838
C	-2.619465	-0.671298	-1.412423
H	-2.687197	0.276507	-1.959305
H	-2.560010	-1.516105	-2.107975
H	-1.757701	-0.672375	-0.730387

[TS-IIIB (F)]

N	1.593075	-0.534494	0.949848
C	0.618283	0.356166	1.417070
O	0.505917	-1.482962	-0.487509
H	1.236186	0.663748	2.310418
S	-1.123898	-3.536036	-0.700905
C	0.145126	-2.609461	-0.127319
S	-0.780000	-0.392539	2.261029
F	0.940668	-3.189825	0.794051
C	0.251560	1.581944	0.601816
C	-0.157844	2.742501	1.270747
C	0.262422	1.573202	-0.796429
C	-0.542786	3.875925	0.558100
H	-0.185353	2.744649	2.362931
C	-0.130353	2.707710	-1.510578
H	0.573333	0.671434	-1.325854
C	-0.531254	3.862627	-0.839213
H	-0.851774	4.774778	1.095196
H	-0.118321	2.686433	-2.602415
H	-0.829881	4.750122	-1.400091
C	2.739836	-0.171584	0.267220
C	3.245027	1.143334	0.165895
C	3.488860	-1.239635	-0.274309
C	4.461242	1.366730	-0.469903
H	2.714867	1.976271	0.625583
C	4.675660	-0.995823	-0.948940
H	3.090512	-2.248655	-0.163156
C	5.168265	0.309219	-1.046556
H	4.859915	2.381195	-0.519079
H	5.232082	-1.826809	-1.384915
H	6.112282	0.500341	-1.559556

N	-3.663154	0.073977	-0.512628
C	-4.794419	0.314210	-1.445846
H	-5.692061	0.541315	-0.859175
H	-4.950316	-0.589132	-2.046557
H	-4.539450	1.161237	-2.093353
C	-3.393032	1.306699	0.284146
H	-2.600146	1.072430	1.009597
H	-4.317658	1.597254	0.796974
H	-3.070955	2.099128	-0.403456
C	-3.995347	-1.049290	0.409575
H	-3.113058	-1.244786	1.032601
H	-4.237341	-1.932611	-0.192060
H	-4.854087	-0.754206	1.023709
C	-2.438280	-0.284170	-1.283306
H	-2.212988	0.541022	-1.968901
H	-2.625582	-1.214363	-1.830268
H	-1.618429	-0.439662	-0.570095

[TS-IIIB (Cl)]

N	1.596187	0.312016	-0.862074
C	0.533656	-0.443530	-1.383347
O	0.644059	1.306309	0.580677
H	1.129804	-0.771202	-2.283472
S	-0.844960	3.405486	1.054173
C	0.349766	2.483988	0.351683
S	-0.774128	0.487308	-2.187365
Cl	1.457213	3.352453	-0.809949
C	0.034499	-1.664438	-0.632676
C	-0.472169	-2.747126	-1.363044
C	0.020220	-1.723460	0.764058
C	-0.976950	-3.870570	-0.712417
H	-0.480249	-2.694586	-2.454365
C	-0.492660	-2.847477	1.416466
H	0.407181	-0.882875	1.342184
C	-0.989962	-3.925060	0.684037
H	-1.361435	-4.708484	-1.297213
H	-0.498659	-2.878924	2.508116
H	-1.382996	-4.804997	1.196517
C	2.682962	-0.233959	-0.194955
C	3.034973	-1.601099	-0.222677
C	3.535109	0.680172	0.461390
C	4.202581	-2.027074	0.400842
H	2.425619	-2.317865	-0.771108
C	4.671077	0.232981	1.120759
H	3.260687	1.735261	0.450007
C	5.011694	-1.122445	1.091114
H	4.480671	-3.080947	0.349466
H	5.307377	0.947210	1.645492
H	5.915614	-1.471264	1.593122
N	-3.757510	0.041393	0.475652
C	-4.911134	-0.189277	1.383617
H	-5.806315	-0.366929	0.776485

H	-5.046301	0.699455	2.010633
H	-4.694669	-1.064429	2.007237
C	-3.520027	-1.171382	-0.361089
H	-2.711934	-0.938369	-1.069778
H	-4.448158	-1.413367	-0.892447
H	-3.231146	-1.997283	0.301663
C	-4.034516	1.206307	-0.411923
H	-3.140026	1.382246	-1.023142
H	-4.246285	2.079802	0.215445
H	-4.899115	0.967142	-1.042048
C	-2.533672	0.327941	1.277883
H	-2.346144	-0.529296	1.934717
H	-2.700329	1.240206	1.860240
H	-1.696317	0.481803	0.583659

[TS-IIIB (Br)]

N	1.463949	0.245997	0.722157
C	0.292693	0.719456	1.332977
O	0.694497	-0.834452	-0.741816
H	0.844962	1.094049	2.242434
S	-0.407242	-3.151206	-1.261477
C	0.621037	-2.052018	-0.566151
S	-0.757441	-0.518730	2.096432
Br	2.074127	-2.830394	0.600900
C	-0.479280	1.860273	0.694690
C	-1.197149	2.736441	1.518950
C	-0.537380	2.033715	-0.691240
C	-1.953524	3.769594	0.971044
H	-1.169269	2.589374	2.601149
C	-1.302766	3.065106	-1.241224
H	0.012979	1.353712	-1.343809
C	-2.010728	3.937545	-0.415335
H	-2.501375	4.447746	1.628211
H	-1.340474	3.186961	-2.325770
H	-2.601476	4.747291	-0.847430
C	2.377527	1.064545	0.071573
C	2.466383	2.458868	0.272980
C	3.334045	0.428350	-0.747536
C	3.484118	3.183418	-0.337972
H	1.772655	2.964403	0.943599
C	4.314422	1.172556	-1.388872
H	3.264535	-0.651571	-0.877928
C	4.397443	2.552700	-1.184463
H	3.560271	4.255916	-0.151886
H	5.032152	0.670565	-2.039558
H	5.181274	3.133253	-1.673499
N	-3.836572	-0.619075	-0.500006
C	-5.071508	-0.638487	-1.326078
H	-5.931060	-0.810926	-0.668090
H	-4.987066	-1.446539	-2.061821
H	-5.168757	0.328311	-1.833315
C	-3.900086	0.505637	0.479106

H	-3.010983	0.442876	1.123902
H	-4.817661	0.398471	1.070060
H	-3.913283	1.449719	-0.080257
C	-3.698400	-1.909803	0.233773
H	-2.738185	-1.893301	0.765999
H	-3.714051	-2.726309	-0.497045
H	-4.536149	-2.003488	0.934669
C	-2.645248	-0.433970	-1.376062
H	-2.760020	0.514434	-1.913902
H	-2.584023	-1.278696	-2.070123
H	-1.751720	-0.419869	-0.737862

[IIIA’]

N	0.470458	1.665006	-0.017154
C	-0.750142	1.315998	-0.028921
S	-2.015680	2.563885	-0.022034
H	-1.153510	3.592613	0.117734
C	1.533342	0.751846	0.096272
C	2.590702	0.837612	-0.820124
C	1.601216	-0.185157	1.138422
C	3.677202	-0.028454	-0.719312
H	2.539538	1.586410	-1.612451
C	2.699598	-1.035835	1.241459
H	0.790157	-0.234990	1.867392
C	3.737819	-0.968530	0.310895
H	4.488074	0.038664	-1.447110
H	2.743255	-1.758475	2.058565
H	4.594228	-1.639476	0.393439
C	-1.304488	-0.070610	-0.105821
C	-0.780338	-0.973649	-1.040720
C	-2.352881	-0.469855	0.731490
C	-1.297544	-2.262944	-1.129381
H	0.029074	-0.660302	-1.702591
C	-2.858040	-1.767041	0.647803
H	-2.765327	0.231098	1.460119
C	-2.332721	-2.663511	-0.281984
H	-0.890680	-2.959265	-1.864334
H	-3.666994	-2.075998	1.311635
H	-2.732504	-3.676699	-0.350252

[TS-IV]

N	-0.573397	1.496747	0.079079
C	0.712955	1.291934	0.048361
S	1.485207	2.841990	-0.031400
H	-0.135020	2.837790	0.002878
C	-1.617339	0.562202	-0.055013
C	-2.731843	0.675385	0.785185
C	-1.590110	-0.430531	-1.045754
C	-3.795143	-0.215513	0.654752
H	-2.747025	1.463565	1.539820
C	-2.657819	-1.315865	-1.168100
H	-0.733051	-0.496793	-1.718406
C	-3.760622	-1.216183	-0.317403

H	-4.657321	-0.125815	1.317899
H	-2.630311	-2.086518	-1.940572
H	-4.595209	-1.911618	-0.418645
C	1.429985	0.000876	0.110549
C	0.948466	-1.037838	0.921478
C	2.614876	-0.174764	-0.615625
C	1.645890	-2.240124	0.994809
H	0.038059	-0.896802	1.506428
C	3.297890	-1.387295	-0.552476
H	2.991913	0.641486	-1.234769
C	2.815030	-2.419466	0.252471
H	1.275442	-3.041766	1.635495
H	4.214355	-1.523430	-1.128484
H	3.355351	-3.366074	0.307798

[IV]

N	0.478180	1.580002	-0.126707
C	-0.853603	1.395542	-0.031170
S	-1.890754	2.688920	0.083520
H	0.759034	2.552231	-0.246291
C	1.541754	0.660396	0.049943
C	2.700532	0.843856	-0.711883
C	1.479854	-0.374738	0.990258
C	3.784786	-0.015358	-0.547532
H	2.740193	1.659871	-1.436285
C	2.563674	-1.237342	1.135254
H	0.590935	-0.498310	1.609516
C	3.717637	-1.064513	0.369263
H	4.684736	0.134616	-1.146207
H	2.508406	-2.044804	1.867280
H	4.564437	-1.741067	0.493005
C	-1.374853	0.000782	-0.111927
C	-0.889122	-0.875908	-1.092176
C	-2.388583	-0.423756	0.754325
C	-1.410870	-2.162817	-1.197831
H	-0.109907	-0.543920	-1.781255
C	-2.891037	-1.719676	0.660239
H	-2.773654	0.271891	1.501520
C	-2.404435	-2.590699	-0.315742
H	-1.038535	-2.835500	-1.972157
H	-3.670911	-2.049139	1.348698
H	-2.804330	-3.603220	-0.393327

5. References

[1] Gaussian 16, Revision C.01, M. J. Frisch, G. W. Trucks, H. B. Schlegel, G. E. Scuseria, M. A. Robb, J. R. Cheeseman, G. Scalmani, V. Barone, G. A. Petersson, H. Nakatsuji, X. Li, M. Caricato, A. V. Marenich, J. Bloino, B. G. Janesko, R. Gomperts, B. Mennucci, H. P. Hratchian, J. V. Ortiz, A. F. Izmaylov, J. L. Sonnenberg, D. Williams-Young, F. Ding, F. Lipparini, F. Egidi, J. Goings, B. Peng, A. Petrone, T. Henderson, D. Ranasinghe, V. G. Zakrzewski, J. Gao, N. Rega, G. Zheng, W. Liang, M. Hada, M. Ehara, K. Toyota, R. Fukuda, J. Hasegawa, M. Ishida, T. Nakajima, Y. Honda, O. Kitao, H. Nakai, T. Vreven, K. Throssell, J. A. Montgomery, Jr., J. E. Peralta, F. Ogliaro, M. J. Bearpark, J. J. Heyd, E. N. Brothers, K. N. Kudin, V. N. Staroverov, T. A. Keith, R. Kobayashi, J. Normand, K. Raghavachari, A. P. Rendell, J. C. Burant, S. S. Iyengar, J. Tomasi, M. Cossi, J. M. Millam, M. Klene, C. Adamo, R. Cammi, J. W. Ochterski, R. L. Martin, K. Morokuma, O. Farkas, J. B. Foresman, and D. J. Fox, Gaussian, Inc., Wallingford CT, 2016.

[2] López-Aguilar, M., Ríos-Lombardía, N., Gallegos, M., Barrena-Espés, D., García-Álvarez, J., Concellón, C., & del Amo, V. (**2025**). Organocatalytic CS_2 insertion into epoxides in neat conditions: a straightforward approach for the efficient synthesis of di- and tri-thiocarbonates. *Chemical Communications*, 61(3), 3488–3491.

[3] *Jmol: an open-source Java viewer for chemical structures in 3D*. http://www.jmol.org/.

[4] Bader, R. F. W. (**1994**). *Atoms in molecules: A quantum theory*. Oxford University Press.

[5] Keith, T.A. *AIMAll (Version 19.10. 12)*; TK Gristmill Software: Overland Park, KS, USA, 2019; p. 23

[6] *Promolden: A QTAIM/IQA code*. Available from the authors upon request by writing to ampendas@uniovi.es
