# Supplementary material for: Unexpected Reactivity of Nitrones: Catalytic Insertion of CS2
Source: Org Lett. 2025 Jul 24;27(30):8338–43. doi: 10.1021/acs.orglett.5c02611 (PMC12322958; doi:10.1021/acs.orglett.5c02611)
Supplement: Supplementary file 2 [file ol5c02611_si_002.pdf]

UNEXPECTED REACTIVITY OF NITRONES: CATALYTIC INSERTION OF CS_2

Marcos López-Aguilar,[a] Nicolás Ríos-Lombardía,[a] Daniel Barrena-Espés,[b] Miguel Gallegos,[b] Joaquín García-Álvarez*,[a] Carmen Concellón*,[a] and Vicente del Amo*.[a]

[a] Laboratorio de Química Sintética Sostenible (*QuimSinSos*), Departamento de Química Orgánica e Inorgánica, Instituto de Química Organometálica Enrique Moles (*IUQOEM*), Centro de Innovación en Química Avanzada (*ORFEO-CINQA*), Facultad de Química, Universidad de Oviedo, E33071, Oviedo, Asturias (Spain).

[b] Departamento de Química Física y Analítica, Facultad de Química, Universidad de Oviedo, E33071, Oviedo, Asturias (Spain).

Electronic Supplementary Information (page S1 of S182)

Table of Contents

I. **Compounds described in this contribution** SI_3

II. **General information** SI_5

III. **Optimization of the experimental procedures** SI_7

IV. **General procedures for the synthesis of nitrones 1a-1ah and spectroscopic data** SI_17

- **IV.1.** Standard procedure for the synthesis of nitrones 1a-1ac SI_17
- **IV.2.** Standard procedure for the synthesis of nitrones 1j-q and 1x-y SI_25
- **IV.3.** Standard procedure for the synthesis of nitrone 1ad SI_30
- **IV.4.** Standard procedure for the synthesis of nitrones 1ae and 1ah SI_31
- **IV.5.** Standard procedure for the synthesis of nitrone 1af SI_32
- **IV.6.** Standard procedure for the synthesis of nitrone 1ag SI_33

V. **General procedures for the synthesis of thioamides 3a-3ah** SI_34

- **V.1.** Standard procedures for the synthesis of thioamides 3a-3ah and spectroscopic data SI_34
- **V.2.** Gram-scale synthesis of thioamide 3a SI_47

VI. **Total synthesis of UC-781** SI_48

- **VI.1.** Aerial oxidation of (2-methyl-3-furyl)methanol (6) SI_48

VI.2. Synthesis of 1-chloro-2-((3-methylbut-2-en-1-yl)oxy)-4-nitrobenzene (5) SI_49

VI.3. Synthesis of nitrone 1ai SI_49

VI.4. Synthesis of 3ai (UC-781) SI_50

VII. Use of Lawesson's reagent SI_52

VIII. X-Ray Data SI_54

IX. References SI_57

X. Copy of NMR and HRMS spectra SI_60

I.- Compounds described in this contribution.

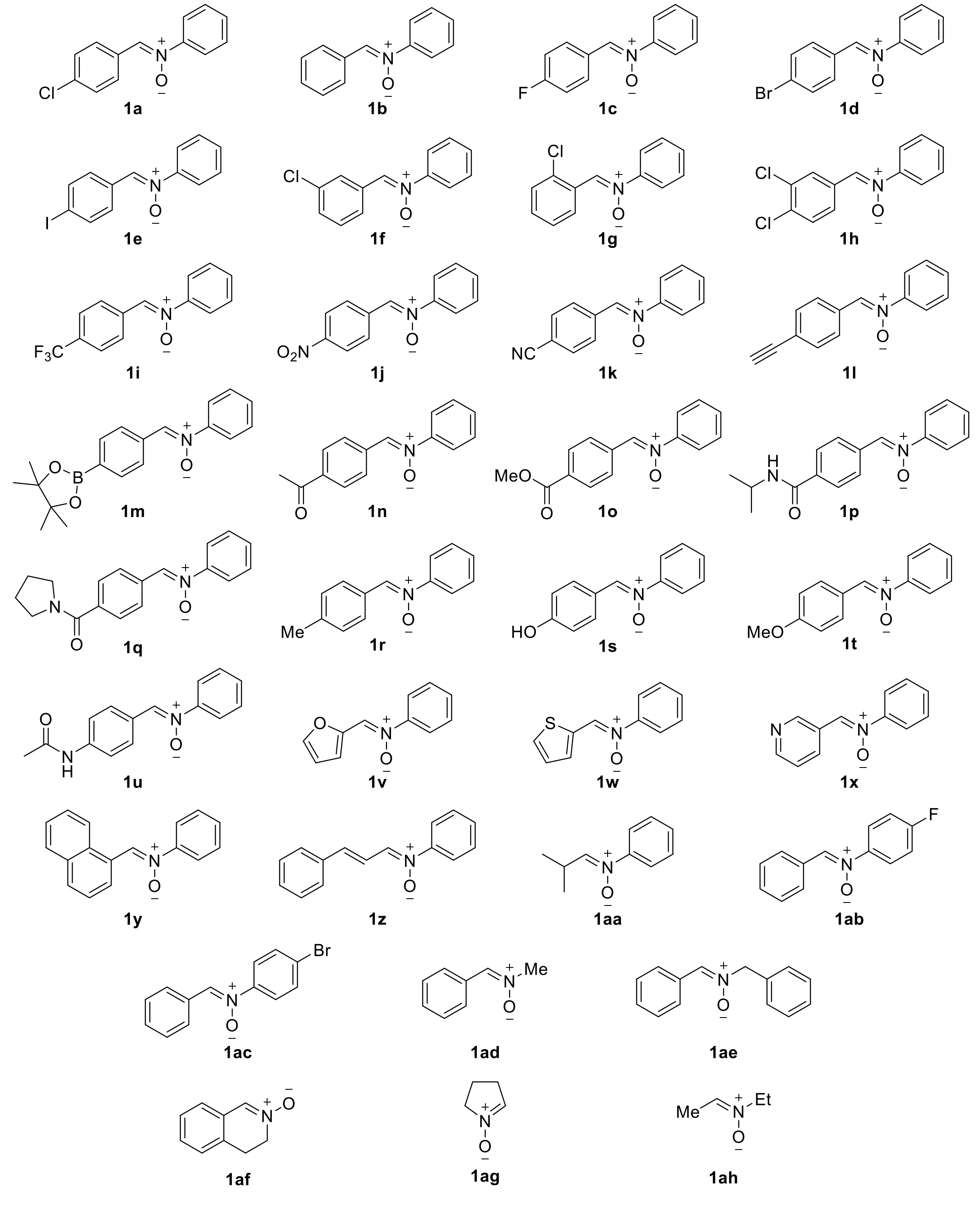

Figure SI_1: Chemical structure of nitrones studied in this contribution

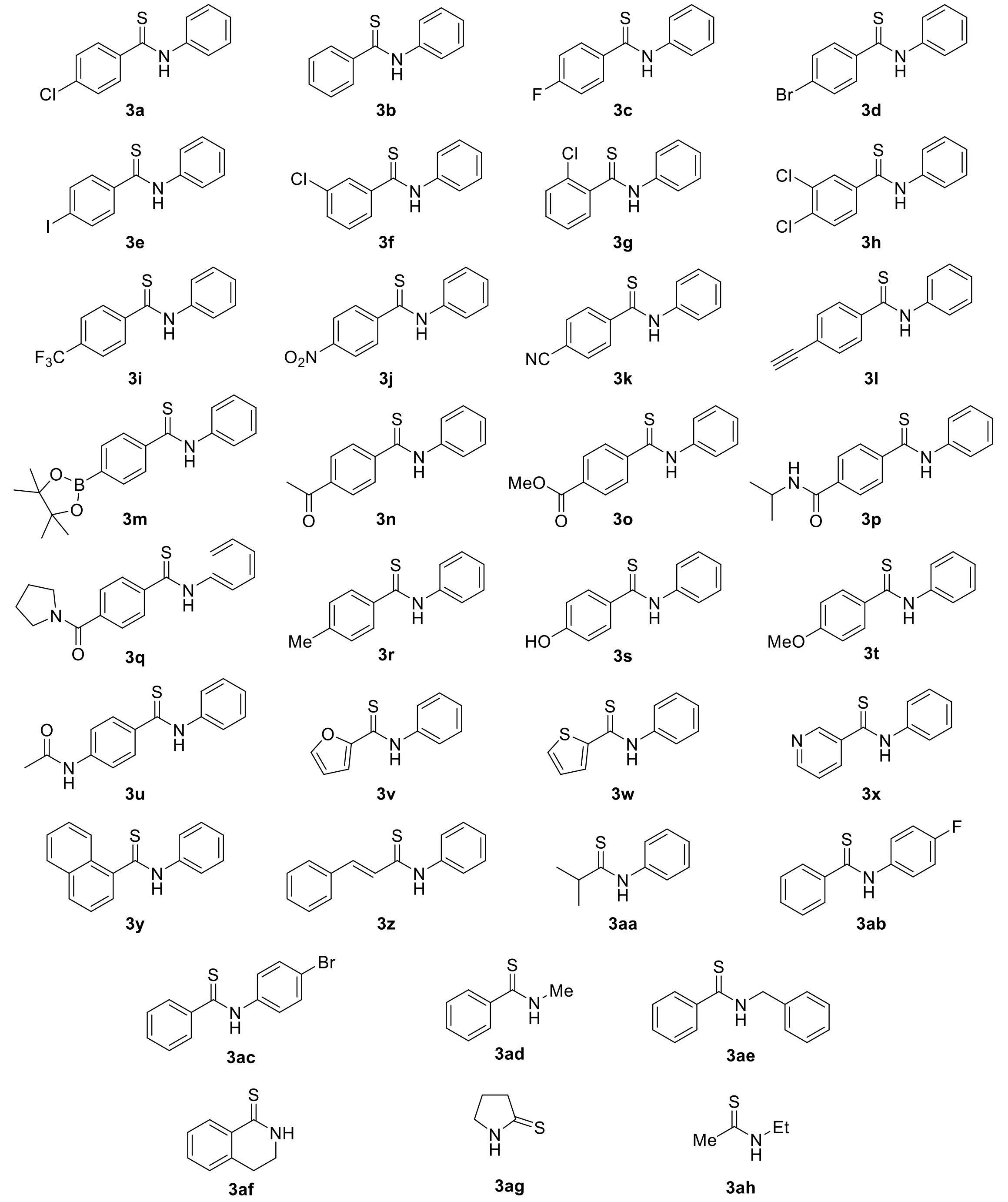

Figure SI_2: Chemical structure of thioamides studied in this contribution

II.- General information.

All commercially available reagents and solvents were used without further purification, unless otherwise noted. Commercial available tetrahydrofuran (THF) was dried by distilling from sodium/benzophenone. Dry N,N-Dimethylformamide (DMF) was purchased from Sigma-Aldrich (biotech. grade, ≥99.9% purity, <0.005% water by *Karl-Fischer* tritation) and stored under an argon atmosphere. Nitrones **1a-ac** were synthesized via *N*-arylhydroxylamines-aldehydes condensation. *N*-Phenylhydroxylamine was obtained by reduction of nitrobenzene with zinc dust.[1] Benzaldehyde and furfural were distilled over reduced pressure before used. 4-Formyl-*N*-isopropylbenzamide and 4-(pyrrolidine-1-carbonyl)benzaldehyde were respectively obtained by condensation of isopropylamine and pyrrolidine with 4-formylbenzoic acid using *N*,*N'*-dicyclohexylcarbodiimide (DCC) as a reagent. Nitrone **1ad** was obtained by condensation of *N*-methylhydroxylamine and benzaldehyde.[2] Nitrones **1ae** and **1ah** were respectively obtained by oxidation of *N*,*N*-dibenzylhydroxylamine and *N*,*N*-diethylhydroxylamine with manganese (IV) oxide.[3] Nitrones **1af-ag** were respectively obtained by oxidation of 1,2,3,4-tetrahydroisoquinoline and pyrrolidine with hydrogen peroxide.[4,5] Details of experimental protocols are included in Section IV.

Flash chromatography of reaction products was carried out using Silica gel 60, particle size 400-630 micron (VWR). Analytical thin layer chromatography (TLC) was performed on DC-Alufolien Kieselgel Silica Gel 60 F254 0.2 mm plates (Merck) and compounds were visualized by UV fluorescence or using either $KMnO_4$ or vanillin stains followed by heating.

^{1}H, $^{13}C\{^{1}H\}$, DEPT and $^{19}F\{^{1}H\}$-NMR spectra ($CDCl_3$, DMSO-d^6) were obtained using a Bruker NAV-300 (^{1}H, 300 MHz; ^{13}C, 75 MHz; ^{19}F, 282 MHz). ^{11}B-NMR spectra ($CDCl_3$) were obtained using a Bruker NAV-400 (^{11}B, 129 MHz). All chemical shifts (δ) are given in parts per million (ppm). Calibration was made on the signal of the solvent ($CDCl_3$ ^{1}H: 7.26, ^{13}C: 77.16, DMSO-d^6 ^{1}H: 2.50; ^{13}C: 39.52).[6] Coupling constants (*J*-values) are given in hertzs (Hz). Chemical shifts are reported as follows: value (description of absorption, coupling constant(s) where applicable, number of protons). The ^{1}H-NMR spectra are presented over the 0-10 ppm region, unless significant signals of the compound are observed outside this range.

High resolution mass spectra (HRMS) experiments were carried out by ESI^+ (electrospray ionization) using a Micro TOF Q spectrometer. Melting points were taken on samples in open capillary tubes and are uncorrected.

X-Ray diffraction data were collected on a Oxford Diffraction Xcalibur Onyx Nova Geminy single crystal diffractometer (details in Section VIII). Crystals were obtained by dissolving **3a** (32 mg)

and **3ai** (49 mg) in the minimum amount of THF at room temperature, inside a 6 mL vial. A layer of *n*-Hexane was then carefully added on top of the solution until the vial was filled. The vial was capped and the solvents were allowed to slowly diffuse. After diffusion was complete, the vial was uncapped and the solvents were allowed to slowly evaporate at room temperature.

III.- Optimization of the experimental procedures.

Table SI_1: Inicial results in the reaction of CS_2 with nitrone **1a**.[a]

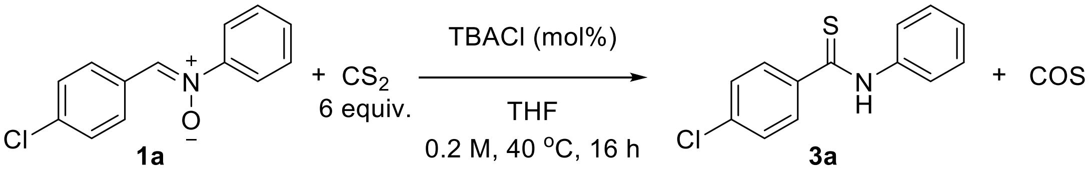

entry	TBACl (mol%)	Yield (%)[b]
**1**	100%	83
**2**	10%	54
**3**	-	0

[a]General conditions: To a solution of *N*-(4-chlorobenzylidene)aniline oxide **1a** (93 mg, 0.4 mmol) and the indicated amount of tetrabutylammonium chloride (TBACl) in 2 mL of THF, CS_2 (145 µL, 2.4 mmol) was added and the reaction mixture was stirred at 40 °C (oil bath) for 16 hours. [b]Yield of pure analytical product 4-chloro-*N*-phenylbenzothioamide **3a** isolated by flash chromatography is given.

Table SI_2: Influence of the solvent in the reaction of CS_2 with nitrone **1a**.[a]

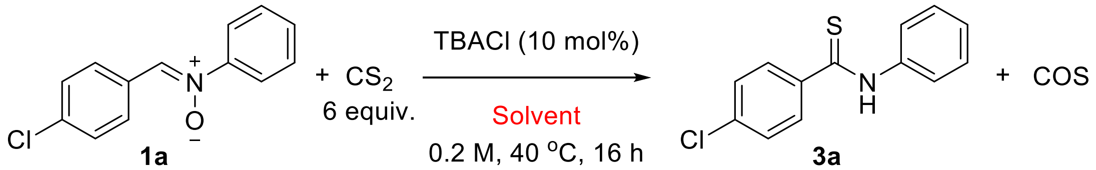

entry	Solvent	Yield (%)[b]
**1**	THF	54
**2**	1,4-Dioxane	46
**3**	2-MeTHF	22
**4**	CPME	17
**5**	DMF	61
**6**	dry DMF	64

[a] General conditions: To a solution of *N*-(4-chlorobenzylidene)aniline oxide **1a** (93 mg, 0.4 mmol) and tetrabutylammonium chloride (TBACl, 11 mg, 0.04 mmol) in 2 mL of the indicated solvent, CS_2 (145 µL, 2.4 mmol) was added and the reaction mixture was stirred at 40 °C (oil bath) for 16 hours. [b] Yield of pure analytical product 4-chloro-*N*-phenylbenzothioamide **3a** isolated by flash chromatography is given.

Table SI_3: Influence of equivalents of CS_2 in its reaction with nitrone **1a**.[a]

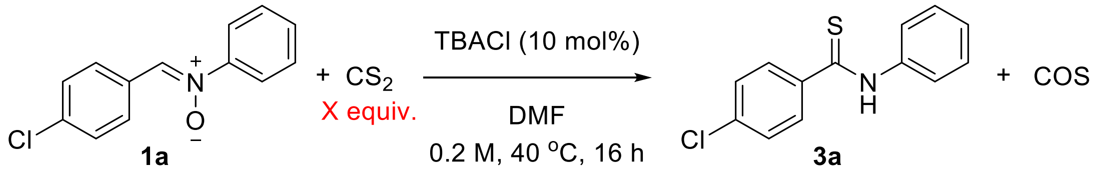

entry	CS_2 (equiv.)	Yield (%)[b]
**1**	1.2	57
**2**	2	58
**3**	6	64

[a] General conditions: To a solution of *N*-(4-chlorobenzylidene)aniline oxide **1a** (93 mg, 0.4 mmol) and tetrabutylammonium chloride (TBACl, 11 mg, 0.04 mmol) in 2 mL of dry DMF, the indicated amount of CS_2 was added and the reaction mixture was stirred at 40 °C (oil bath) for 16 hours under argon atmosphere. [b] Yield of pure analytical product 4-chloro-*N*-phenylbenzothioamide **3a** isolated by flash chromatography is given.

Table SI_4: Influence of the anion of tetrabutylammonium salts in the reaction of CS_2 with nitrone **1a**.[a]

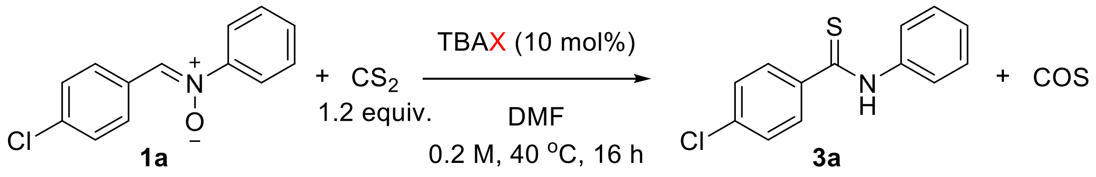

entry	TBAX (10 mol%)	Yield (%)[b]
**1**	TBAF	54
**2**	TBACl	57
**3**	TBABr	40
**4**	TBAI	24
**5**	TBAOTf	0

[a] General conditions: To a solution of *N*-(4-chlorobenzylidene)aniline oxide **1a** (93 mg, 0.4 mmol) and the indicated tetrabutylammonium salt (0.04 mmol) in 2 mL of dry DMF, CS_2 (29 µL, 0.48 mmol) was added and the reaction mixture was stirred at 40 °C (oil bath) for 16 hours under argon atmosphere. [b] Yield of pure analytical product 4-chloro-*N*-phenylbenzothioamide **3a** isolated by flash chromatography is given.

Table SI_5: Influence of the chloride salt in the reaction of CS_2 with nitrone **1a**.[a]

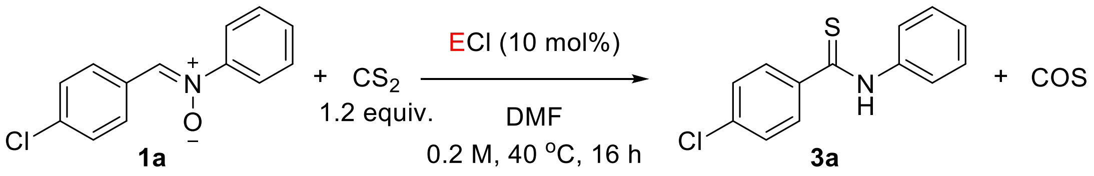

entry	ECl (10 mol%)	Yield (%)[b]
**1**	NaCl	31
**2**	LiCl	53
**3**	ChCl	47
**4**	BmimCl	41
**5**	TMACl	54
**6**	TBACl	57

[a] General conditions: To a solution of *N*-(4-chlorobenzylidene)aniline oxide **1a** (93 mg, 0.4 mmol) and the indicated chloride salt (0.04 mmol) in 2 mL of dry DMF, CS_2 (29 µL, 0.48 mmol) was added and the reaction mixture was stirred at 40 °C (oil bath) for 16 hours under argon atmosphere. [b] Yield of pure analytical product 4-chloro-*N*-phenylbenzothioamide **3a** isolated by flash chromatography is given.

Table SI_6: Influence of catalyst charge in the reaction of CS_2 with nitrone **1a**.[a]

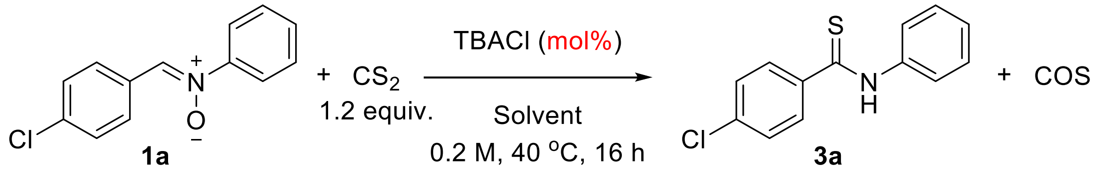

entry	Solvent	TBACl (mol%)	Yield (%)[b]
**1**	dry DMF	10	57
**2**	dry DMF	20	68
**3**	dry DMF	50	67
**4**	dry DMF	120	64
**5**	dry THF	120	71

[a] General conditions: To a solution of *N*-(4-chlorobenzylidene)aniline oxide **1a** (93 mg, 0.4 mmol) and the indicated amount of tetrabutylammonium chloride (TBACl) in 2 mL of dry DMF or THF, CS_2 (29 µL, 0.48 mmol) was added and the reaction mixture was stirred at 40 °C (oil bath) for 16 hours under argon atmosphere. [b] Yield of pure analytical product 4-chloro-*N*-phenylbenzothioamide **3a** isolated by flash chromatography is given.

Table SI_7: Influence of temperature in the reaction of CS_2 with nitrone **1a**. [a]

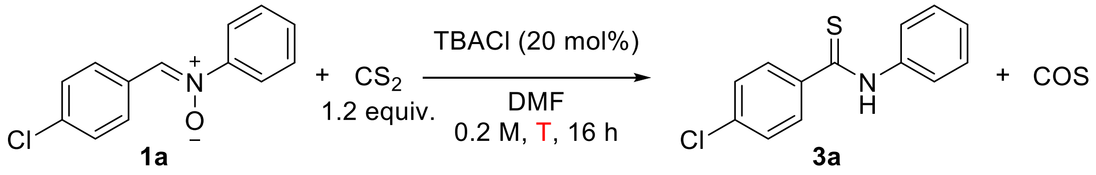

entry	T (ºC)	Yield (%)[b]
**1**	r.t.	62
**2**	40	68
**3**	60	68

[a] General conditions: To a solution of *N*-(4-chlorobenzylidene)aniline oxide **1a** (93 mg, 0.4 mmol) and tetrabutylammonium chloride (TBACl, 22 mg, 0.08 mmol) in 2 mL of dry DMF, CS_2 (29 µL, 0.48 mmol) was added and the reaction mixture was stirred at the indicated temperature (oil bath) for 16 hours under argon atmosphere. [b] Yield of pure analytical product 4-chloro-*N*-phenylbenzothioamide **3a** isolated by flash chromatography is given.

Table SI_8: Influence of concentration in the reaction of CS_2 with nitrone **1a**. [a]

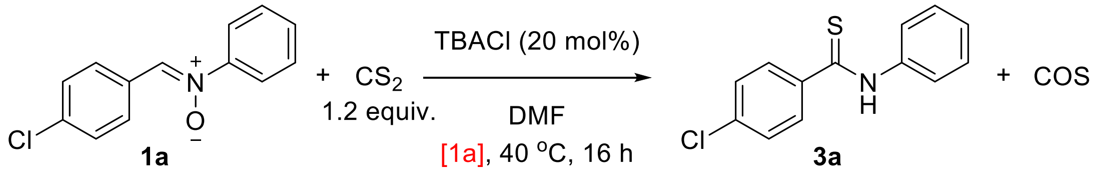

Entry	[1a] (M)	Yield (%)[b]
**1**	0.1 M	63
**2**	0.2 M	68
**3**	0.4 M	67

[a] General conditions: To a solution of *N*-(4-chlorobenzylidene)aniline oxide **1a** (93 mg, 0.4 mmol) and tetrabutylammonium chloride (TBACl, 22 mg, 0.08 mmol) in the appropriate volume of dry DMF, CS_2 (29 µL, 0.48 mmol) was added and the reaction mixture was stirred at 40 °C (oil bath) for 16 hours under argon atmosphere. [b] Yield of pure analytical product 4-chloro-*N*-phenylbenzothioamide **3a** isolated by flash chromatography is given.

Table SI_9: Use of a mixture of solvents in the reaction of CS_2 with nitrone **1a**.[a]

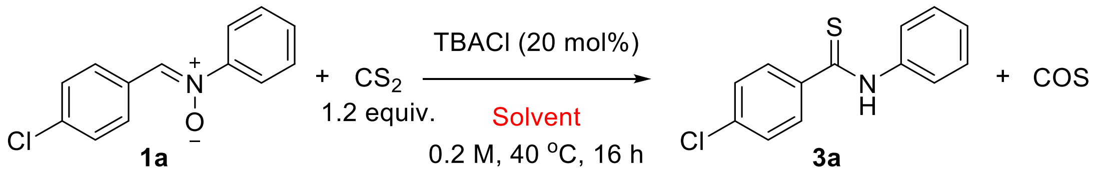

entry	Solvent	Yield (%)[b]
**1**	dry DMF	68
**2**	dry DMF / dry THF (1:1)	74

[a] General conditions: To a solution of *N*-(4-chlorobenzylidene)aniline oxide **1a** (93 mg, 0.4 mmol) and tetrabutylammonium chloride (TBACl, 22 mg, 0.08 mmol) in 2 mL of the indicated solvent, CS_2 (29 µL, 0.48 mmol) was added and the reaction mixture was stirred at 40 °C (oil bath) for 16 hours under argon atmosphere. [b] Yield of pure analytical product 4-chloro-*N*-phenylbenzothioamide **3a** isolated by flash chromatography is given.

Table SI_10: Use of different tetrabutylammonium salts in optimal reaction conditions. [a]

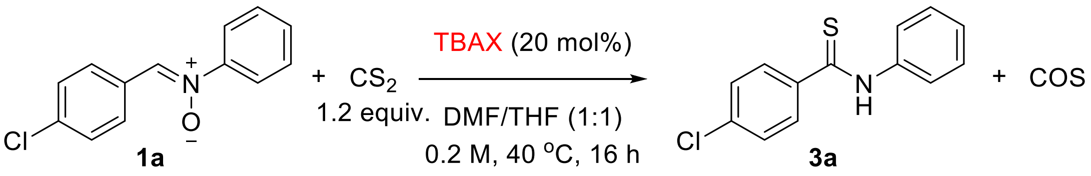

entry	TBAX (20 mol%)	Yield (%)[b]
**1**	TBAF	49
**2**	TBACl	74
**3**	TBAOTf	0
**4**	-	0

[a] General conditions: To a solution of *N*-(4-chlorobenzylidene)aniline oxide **1a** (93 mg, 0.4 mmol) and the corresponding tetrabutylammonium salt (0.08 mmol) in 2 mL of a mixture 1:1 of dry THF and dry DMF, CS_2 (29 µL, 0.48 mmol) was added and the reaction mixture was stirred at 40 °C (oil bath) for 16 hours under argon atmosphere. [b] Yield of pure analytical product 4-chloro-*N*-phenylbenzothioamide **3a** isolated by flash chromatography is given.

IV.- General procedures for the synthesis of nitrones 1a-1ah and spectroscopic data.

IV.1.- Standard procedure for the synthesis of nitrones 1a-1ac.[1]

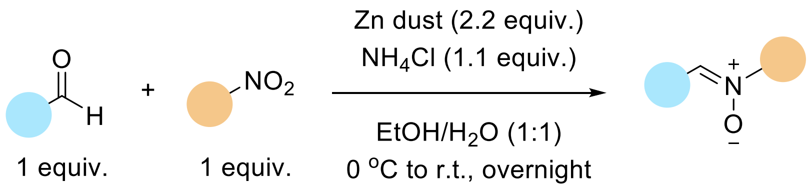

Scheme SI_1: Synthesis of nitrones **1a-i**, **1r-w** and **1z-ac**.

The corresponding nitro-compound (1 equiv.), the corresponding aldehyde (1 equiv.) and NH_4Cl (1.1 equiv.) were added sequentially to a mixture EtOH/H_2O (1:1, 0.55 M) which was stirred at room temperature for 5 minutes before it was cooled to 0 °C (ice bath) and zinc dust (2.2 equiv.) added in portions over 10 minutes. Then, the reaction mixture was allowed to slowly reach room temperature and stirred overnight. The mixture was then filtered through a pad of Celite® and extracted with CH_2Cl_2. The aqueous phase was washed with CH_2Cl_2 twice, the organic layers were combined, dried with $MgSO_4$ and filtered. Solvents and volatiles were removed under vacuum. Pure products were generally obtained by recrystallization from hot EtOAc/*n*-Hexane in low to moderate yields (10-60%).

_N_-(4-Chlorobenzylidene)aniline oxide (1a)

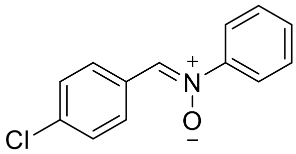

Synthetized from 4-chlorobenzaldehyde (3.37 g, 24 mmol) and nitrobenzene (2.47 mL, 24 mmol). Purified by recrystallization from hot EtOAc (white solid, 2.40 g, 43% yield). ^{1}H NMR (300 MHz, $CDCl_3$): δ (ppm) = 8.36 (d, *J* = 8.8 Hz, 2H), 7.91 (s, 1H), 7.78 – 7.74 (m, 2H), 7.51 – 7.42 (m, 5H). ^{13}C NMR (75 MHz, $CDCl_3$): δ (ppm) = 148.9 (C), 136.3 (C), 133.4 (CH), 130.2 (2CH), 130.1 (CH), 129.2 (2CH), 129.2 (C), 128.9 (2CH), 121.7 (2CH). The spectroscopic data were matched with the one reported in the literature.[7]

N-Benzylideneaniline oxide (1b)

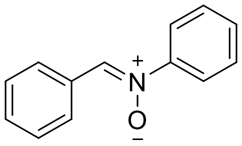

Synthetized from benzaldehyde (3.24 mL, 32 mmol) and nitrobenzene (3.29 mL, 32 mmol). Purified by recrystallization from hot EtOAc/*n*-Hexane (white solid, 1.28 g, 20% yield). ^{1}H NMR (300 MHz, $CDCl_3$): δ (ppm) = 8.42 – 8.39 (m, 2H), 7.93 (s, 1H), 7.80 – 7.76 (m, 2H), 7.52 – 7.47 (m, 6H). ^{13}C NMR (75 MHz, $CDCl_3$): δ (ppm) = 149.1 (C), 134.7 (CH), 131.0 (CH), 130.7 (C), 130.0 (CH), 129.2 (2CH), 129.1 (2CH), 128.7 (2CH), 121.8 (2CH). The spectroscopic data were matched with the one reported in the literature.[7]

N-(4-Fluorobenzylidene)aniline oxide (1c)

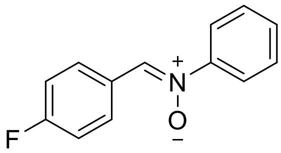

Synthetized from 4-fluorobenzaldehyde (1.69 mL, 16 mmol) and nitrobenzene (1.65 mL, 16 mmol). Purified by recrystallization from hot EtOAc (white solid, 736 mg, 21% yield). ^{1}H NMR (300 MHz, $CDCl_3$): δ (ppm) = 8.44 (dd, *J* = 9.0, 5.6 Hz, 2H), 7.90 (s, 1H), 7.76 – 7.73 (m, 2H), 7.50 – 7.43 (m, 3H), 7.14 (t, *J* = 8.8 Hz, 2H). ^{19}F NMR (282 MHz, $CDCl_3$): δ (ppm) = -106.6. ^{13}C NMR (75 MHz, $CDCl_3$): δ (ppm) = 163.6 (d, *J* = 253.7 Hz, C), 148.9 (C), 133.4 (CH), 131.3 (d, *J* = 8.5 Hz, 2CH), 130.0 (CH), 129.2 (2CH), 127.2 (d, *J* = 3.4 Hz, C), 121.7 (2CH), 115.8 (d, *J* = 21.8 Hz, 2CH). The spectroscopic data were matched with the one reported in the literature.[8]

N-(4-Bromobenzylidene)aniline oxide (1d)

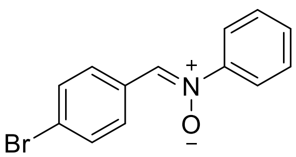

Synthetized from 4-bromobenzaldehyde (2.96 g, 16 mmol) and nitrobenzene (1.65 mL, 16 mmol). Purified by recrystallization from hot EtOAc (white solid, 1.53 g, 35% yield). ^{1}H NMR (300 MHz, $CDCl_3$): δ (ppm) = 8.27 (d, *J* = 8.8 Hz, 2H), 7.89 (s, 1H), 7.77 – 7.71 (m, 2H), 7.58 (d, *J* = 8.7 Hz, 2H), 7.50 – 7.43 (m, 3H). ^{13}C NMR (75 MHz, $CDCl_3$): δ (ppm) = 148.9 (C), 133.5 (CH), 131.9 (2CH), 130.3 (2CH), 130.2 (CH), 129.6 (C), 129.2 (2CH), 124.8 (C), 121.7 (2CH). The spectroscopic data were matched with the one reported in the literature.[8]

***N*-(4-Iodobenzylidene)aniline oxide (1e)**

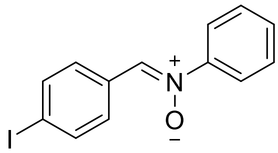

Synthetized from 4-iodobenzaldehyde (3.71 g, 16 mmol) and nitrobenzene (1.65 mL, 16 mmol). Purified by recrystallization from hot EtOAc (white solid, 1.03 g, 20% yield). ^{1}H NMR (300 MHz, $CDCl_3$): δ (ppm) = 8.12 (d, *J* = 8.6 Hz, 2H), 7.87 (s, 1H), 7.79 (d, *J* = 8.6 Hz, 2H), 7.76 – 7.71 (m, 2H), 7.49 – 7.44 (m, 3H). ^{13}C NMR (75 MHz, $CDCl_3$): δ (ppm) = 149.0 (C), 137.9 (2CH), 133.6 (CH), 130.2 (2CH), 130.2 (CH), 130.0 (C), 129.3 (2CH), 121.7 (2CH), 97.1 (C). The spectroscopic data were matched with the one reported in the literature.[7]

***N*-(3-Chlorobenzylidene)aniline oxide (1f)**

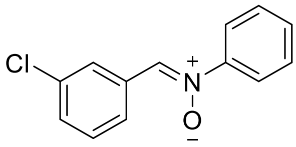

Synthetized from 3-chlorobenzaldehyde (1.81 mL, 16 mmol) and nitrobenzene (1.65 mL, 16 mmol). Purified by recrystallization from hot EtOAc/*n*-Hexane (pale yellow solid, 1.22 g, 33% yield). ^{1}H NMR (300 MHz, $CDCl_3$): δ (ppm) = 8.56 (s, 1H), 8.16 (dt, *J* = 6.8, 1.9 Hz, 1H), 7.90 (s, 1H), 7.80 – 7.73 (m, 2H), 7.52 – 7.38 (m, 5H). ^{13}C NMR (75 MHz, $CDCl_3$): δ (ppm) = 148.9 (C), 134.7 (C), 133.2 (CH), 132.2 (C), 130.8 (CH), 130.3 (CH), 129.8 (CH), 129.3 (2CH), 128.4 (CH), 127.1 (CH), 121.7 (2CH). The spectroscopic data were matched with the one reported in the literature.[8]

***N*-(2-Chlorobenzylidene)aniline oxide (1g)**

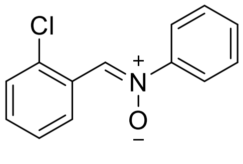

Synthetized from 2-chlorobenzaldehyde (1.80 mL, 16 mmol) and nitrobenzene (1.65 mL, 16 mmol). Purified by recrystallization from hot EtOAc/*n*-Hexane (pale yellow solid, 1.83 g, 49% yield). ^{1}H NMR (300 MHz, $CDCl_3$): δ (ppm) = 9.53 (dd, *J* = 7.5, 2.4 Hz, 1H), 8.42 (s, 1H), 7.80 – 7.77 (m, 2H), 7.52 – 7.36 (m, 6H). ^{13}C NMR (75 MHz, $CDCl_3$): δ (ppm) = 149.5 (C), 133.6 (C), 131.6 (CH), 130.5 (CH), 130.2 (CH), 129.6 (CH), 129.3 (2CH), 129.2 (CH), 128.4 (C), 127.2 (CH), 121.9 (2CH). The spectroscopic data were matched with the one reported in the literature.[8]

***N*-(3,4-Dichlorobenzylidene)aniline oxide (1h)**

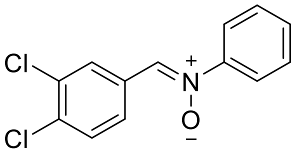

Synthetized from 3,4-dichlorobenzaldehyde (2.80 g, 16 mmol) and nitrobenzene (1.65 mL, 16 mmol). Purified by recrystallization from hot EtOAc/*n*-Hexane (white solid, 992 mg, 23% yield). ^{1}H NMR (300 MHz, $CDCl_3$): δ (ppm) = 8.68 (d, *J* = 2.1 Hz, 1H), 8.08 (dd, *J* = 8.5, 2.1 Hz, 1H), 7.88 (s, 1H), 7.74 – 7.71 (m, 2H), 7.51 – 7.45 (m, 4H). ^{13}C NMR (75 MHz, $CDCl_3$): δ (ppm) = 148.8 (C), 134.3 (C), 133.0 (C), 132.2 (CH), 130.5 (CH), 130.5 (C), 130.4 (CH), 130.0 (CH), 129.3 (2CH), 128.0 (CH), 121.6 (2CH). The spectroscopic data were matched with the one reported in the literature.[9]

***N*-[4-(Trifluoromethyl)benzylidene]aniline oxide (1i)**

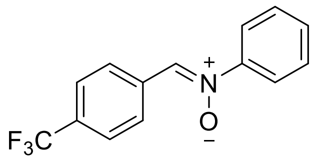

Synthetized from 4-(trifluoromethyl)benzaldehyde (2.19 mL, 16 mmol) and nitrobenzene (1.65 mL, 16 mmol). Purified by recrystallization from hot EtOAc (white solid, 1.45 g, 34% yield). ^{1}H NMR (300 MHz, $CDCl_3$): δ (ppm) = 8.49 (d, *J* = 8.3 Hz, 2H), 8.00 (s, 1H), 7.80 – 7.73 (m, 2H), 7.70 (d, *J* = 8.6 Hz, 2H), 7.51 – 7.46 (m, 3H). ^{19}F NMR (282 MHz, $CDCl_3$): δ (ppm) = -69.9. ^{13}C NMR (75 MHz, $CDCl_3$): δ (ppm) = 148.9 (C), 133.7 (C), 133.1 (CH), 131.8 (q, *J* = 32.8 Hz, C), 130.4 (CH), 129.3 (2CH), 128.9 (2CH), 125.6 (q, *J* = 4.0 Hz, 2CH), 124.4 (q, *J* = 272.2 Hz, C), 121.7 (2CH). The spectroscopic data were matched with the one reported in the literature.[10]

N-(4-Methylbenzylidene)aniline oxide (1r)

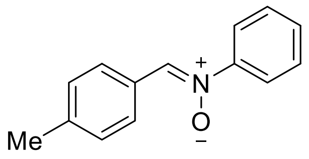

Synthetized from *p*-tolualdehyde (1.89 mL, 16 mmol) and nitrobenzene (1.65 mL, 16 mmol). Purified by recrystallization from hot EtOAc/*n*-Hexane (pale yellow solid, 1.71 g, 50% yield). ^{1}H NMR (300 MHz, $CDCl_3$): δ (ppm) = 8.30 (d, *J* = 8.3 Hz, 2H), 7.88 (s, 1H), 7.77 – 7.74 (m, 2H), 7.49 – 7.40 (m, 3H), 7.27 (d, *J* = 7.8 Hz, 2H), 2.40 (s, 3H). ^{13}C NMR (75 MHz, $CDCl_3$): δ (ppm) = 149.1 (C), 141.6 (C), 134.7 (CH), 129.8 (2CH), 129.4 (2CH), 129.1 (3CH), 128.1 (C), 121.7 (2CH), 21.8 (CH_3). The spectroscopic data were matched with the one reported in the literature.[7]

N-(4-Hydroxybenzylidene)aniline oxide (1s)

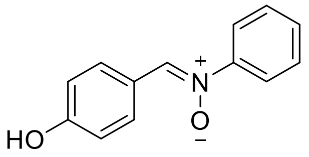

Synthetized from 4-hydroxybenzaldehyde (1.95 g, 16 mmol) and nitrobenzene (1.65 mL, 16 mmol). Purified by recrystallization from hot EtOAc (pale yellow solid, 1.03 g, 29% yield). ^{1}H NMR (300 MHz, DMSO-*d*^{6}): δ (ppm) = 10.25 (*br* s, 1H), 8.39 (d, *J* = 8.7 Hz, 2H), 8.34 (s, 1H), 7.86 (d, *J* = 7.5 Hz, 2H), 7.53 – 7.42 (m, 3H), 6.87 (d, *J* = 8.7 Hz, 2H). ^{13}C NMR (75 MHz, DMSO-*d*^{6}): δ (ppm) = 160.2 (C), 148.9 (C), 133.8 (CH), 131.7 (2CH), 129.8 (CH), 129.5 (2CH), 123.0 (C), 121.7 (2CH), 115.8 (2CH). HMRS (ESI$^+$, m/z) calculated for $(C_{13}H_{12}NO_2)^+$ [(M+H)$^+$]: 214.0863; found: 214.0871.

N-(4-Methoxybenzylidene)aniline oxide (1t)

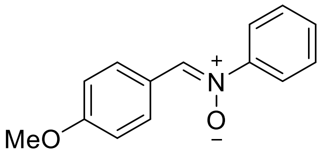

Synthetized from p-anisaldehyde (1.95 mL, 16 mmol) and nitrobenzene (1.65 mL, 16 mmol). Purified by recrystallization from hot EtOAc/*n*-Hexane (pale yellow solid,1.14 g, 31% yield). ^{1}H NMR (300 MHz, $CDCl_3$): δ (ppm) = 8.41 – 8.36 (m, 2H), 7.83 (s, 1H), 7.76 – 7.71 (m, 2H), 7.46 – 7.39 (m, 3H), 6.98 – 6.93 (m, 2H), 3.83 (s, 3H). ^{13}C NMR (75 MHz, $CDCl_3$): δ (ppm) = 161.5 (C), 148.9 (C), 134.2 (CH), 131.2 (2CH), 129.6 (CH), 129.1 (2CH), 123.8 (C), 121.6 (2CH), 114.0 (2CH), 55.4 (CH_3). The spectroscopic data were matched with the one reported in the literature.[11]

N-(4-Acetamidobenzylidene)aniline oxide (1u)

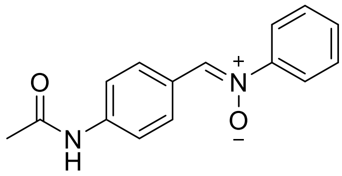

Synthetized from 4-acetamidobenzaldehyde (2.61 g, 16 mmol) and nitrobenzene (1.65 mL, 16 mmol). Purified by recrystallization from hot EtOAc (yellow solid, 437 mg, 11% yield). ^{1}H NMR (300 MHz, DMSO-d^{6}): δ (ppm) = 10.25 (s, 1H), 8.43 (d, *J* = 8.7 Hz, 2H), 8.40 (s, 1H), 7.88 (d, *J* = 8.1 Hz, 2H), 7.70 (d, *J* = 8.7 Hz, 2H), 7.55 – 7.44 (m, 3H), 2.07 (s, 3H). ^{13}C NMR (75 MHz, DMSO-d^{6}): δ (ppm) = 169.2 (C), 148.9 (C), 141.7 (C), 133.5 (CH), 130.3 (2CH), 130.1 (CH), 129.5 (2CH), 126.4 (C), 121.8 (2CH), 118.7 (2CH), 24.6 (CH_3). HMRS (ESI^+, m/z) calculated for $(C_{15}H_{15}N_2O_2)^+$ [$(M+H)^+$]: 255.1128; found: 255.1129.

N-(Furan-2-ylmethylidene)aniline oxide (1v)

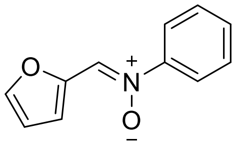

Synthetized from furfural (1.33 mL, 16 mmol) and nitrobenzene (1.65 mL, 16 mmol). Purified by recrystallization from hot EtOAc (orangish solid, 951 mg, 32% yield). ^{1}H NMR (300 MHz, $CDCl_3$): δ (ppm) = 8.14 (s, 1H), 7.99 (d, *J* = 3.6 Hz, 1H), 7.79 – 7.75 (m, 2H), 7.55 (d, *J* = 1.9 Hz, 1H), 7.48 – 7.41 (m, 3H), 6.61 (dd, *J* = 3.7, 1.9 Hz, 1H). ^{13}C NMR (75 MHz, $CDCl_3$): δ (ppm) = 147.5 (C), 147.3 (C), 144.7 (CH), 130.0 (CH), 129.2 (2CH), 124.4 (CH), 121.0 (2CH), 116.6 (CH), 112.7 (CH). The spectroscopic data were matched with the one reported in the literature.[7]

N-(Thiophen-2-ylmethylidene)aniline oxide (1w)

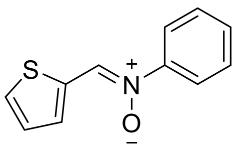

Synthetized from 2-thiophenecarboxaldehyde (1.50 mL, 16 mmol) and nitrobenzene (1.65 mL, 16 mmol). Purified by recrystallization from hot EtOAc/*n*-Hexane (yellow solid, 1.96 g, 60% yield). ^{1}H NMR (300 MHz, $CDCl_3$): δ (ppm) = 8.48 (s, 1H), 7.84 – 7.78 (m, 2H), 7.60 (d, *J* = 4.0 Hz, 1H), 7.54 (d, *J* = 5.1 Hz, 1H), 7.50 – 7.40 (m, 3H), 7.19 (dd, *J* = 5.2, 3.8 Hz, 1H). ^{13}C NMR (75 MHz, $CDCl_3$): δ (ppm) = 146.5 (C), 133.0 (C), 131.1 (CH), 130.1 (CH), 129.9 (CH), 129.2 (2CH), 128.9 (CH),

127.1 (CH), 121.0 (2CH). The spectroscopic data were matched with the one reported in the literature.[7]

***N*-Cinnamylidineaniline oxide (1z)**

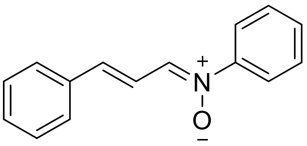

Synthetized from (*Z*)-cinnamaldehyde (2.01 mL, 16 mmol) and nitrobenzene (1.65 mL, 16 mmol). Purified by recrystallization from hot EtOAc (yellow solid, 1.27 g, 36% yield). ^{1}H NMR (300 MHz, $CDCl_3$): δ (ppm) = 7.84 (d, *J* = 9.7 Hz, 1H), 7.77 – 7.65 (m, 3H), 7.59 – 7.50 (m, 2H), 7.47 – 7.40 (m, 3H), 7.38 – 7.28 (m, 3H), 7.14 (d, *J* = 16.0 Hz, 1H). ^{13}C NMR (75 MHz, $CDCl_3$): δ (ppm) = 147.4 (C), 140.0 (CH), 136.3 (CH), 136.1 (C), 130.0 (CH), 129.5 (CH), 129.1 (2CH), 128.9 (2CH), 127.5 (2CH), 121.4 (2CH), 119.1 (CH). The spectroscopic data were matched with the one reported in the literature.[12]

***N*-(2-Methylpropylidine)aniline oxide (1aa)**

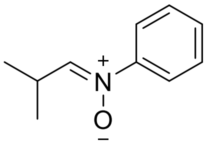

Synthetized from isobutyraldehyde (1.46 mL, 16 mmol) and nitrobenzene (1.65 mL, 16 mmol). Purified by flash chromatography on silica gel (CH_2Cl_2/MeOH 99:1 to 97:3, yellow oil, 255 mg, 10% yield). ^{1}H NMR (300 MHz, $CDCl_3$): δ (ppm) = 7.66 – 7.60 (m, 2H), 7.42 – 7.37 (m, 3H), 7.05 (d, *J* = 7.3 Hz, 1H), 3.35 (hept, *J* = 6.9 Hz, 1H), 1.20 (d, *J* = 6.9 Hz, 6H). ^{13}C NMR (75 MHz, $CDCl_3$): δ (ppm) = 147.7 (C), 145.1 (CH), 129.8 (CH), 129.0 (2CH), 121.7 (2CH), 26.7 (CH), 19.0 ($2CH_3$). The spectroscopic data were matched with the one reported in the literature.[13]

4-Fluoro-*N*-benzylideneaniline oxide (1ab)

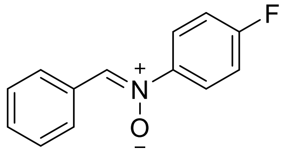

Synthetized from benzaldehyde (1.62 mL, 16 mmol) and 1-fluoro-4-nitrobenzene (2.26 g, 16 mmol). Purified by recrystallization from hot EtOAc (white solid, 923 mg, 27% yield). ^{1}H NMR (300 MHz, $CDCl_3$): δ (ppm) = 8.40 – 8.34 (m, 2H), 7.87 (s, 1H), 7.80 – 7.73 (m, 2H), 7.47– 7.45 (m, 3H), 7.17 – 7.09 (m, 2H). ^{19}F NMR (282 MHz, $CDCl_3$): δ (ppm) = -110.4. ^{13}C NMR (75 MHz, $CDCl_3$): δ (ppm) = 163.1 (d, *J* = 250.6 Hz, C), 145.3 (C), 134.6 (CH), 131.1 (CH), 130.5 (C), 129.1 (2CH), 128.7 (2CH), 123.7 (d, *J* = 8.8 Hz, 2CH), 116.0 (d, *J* = 23.5 Hz, 2CH). The spectroscopic data were matched with the one reported in the literature.[14]

4-Bromo-*N*-benzylideneaniline oxide (1ac)

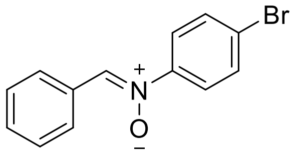

Synthetized from benzaldehyde (1.62 mL, 16 mmol) and 1-bromo-4-nitrobenzene (3.23 g, 16 mmol). Purified by recrystallization from hot EtOAc (white solid, 1.00 g, 23% yield). ^{1}H NMR (300 MHz, $CDCl_3$): δ (ppm) = 8.41 – 8.36 (m, 2H), 7.90 (s, 1H), 7.67 (d, *J* = 8.8 Hz, 2H), 7.59 (d, *J* = 8.9 Hz, 2H), 7.49 – 7.46 (m, 3H). ^{13}C NMR (75 MHz, $CDCl_3$): δ (ppm) = 147.9 (C), 134.6 (CH), 132.3 (2CH), 131.3 (CH), 130.4 (C), 129.1 (2CH), 128.7 (2CH), 123.9 (C), 123.3 (2CH). The spectroscopic data were matched with the one reported in the literature.[14]

IV.2.- Standard procedure for the synthesis of nitrones 1j-q and 1x-y.[1]

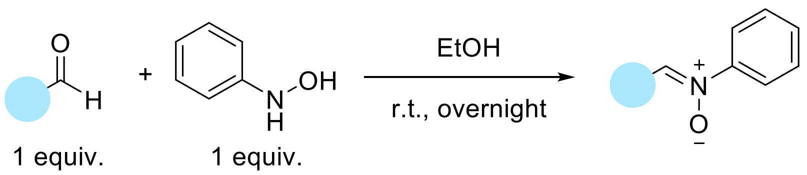

Scheme SI_2: Synthesis of nitrones **1j-q** and **1x-y**.

N-Phenylhydroxylamine (1 equiv.) and the corresponding aldehyde (1 equiv.) were dissolved in dry EtOH (0.6 M) and the mixture was stirred overnight under argon at room temperature. Then, solvent and volatiles were removed under vacuum and pure products were generally obtained by recrystallization from hot EtOAc/*n*-Hexane in low to high yields (22-88%).

N-(4-Nitrobenzylidene)aniline oxide (1j)

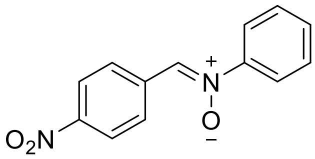

Synthetized from 4-nitrobenzaldehyde (907 mg, 6 mmol) and *N*-phenylhydroxylamine (655 mg, 6 mmol). Purified by recrystallization from hot EtOAc (yellow solid, 1.28 g, 88% yield). ^{1}H NMR (300 MHz, $CDCl_3$): δ (ppm) = 8.55 (d, *J* = 9.0 Hz, 2H), 8.30 (d, *J* = 9.0 Hz, 2H), 8.07 (s, 1H), 7.82 – 7.75 (m, 2H), 7.55 – 7.49 (m, 3H). ^{13}C NMR (75 MHz, $CDCl_3$): δ (ppm) = 148.9 (C), 148.0 (C), 136.2 (C), 132.3 (CH), 130.8 (CH), 129.4 (2CH), 129.2 (2CH), 123.9 (2CH), 121.7 (2CH). The spectroscopic data were matched with the one reported in the literature.[10]

N-(4-Cyanobenzylidene)aniline oxide (1k)

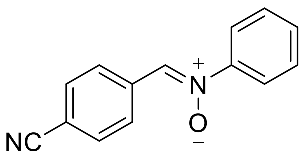

Synthetized from 4-cyanobenzaldehyde (393 mg, 3 mmol) and *N*-phenylhydroxylamine (327 mg, 3 mmol). Purified by recrystallization from hot EtOAc/*n*-Hexane (white solid, 250 mg, 38% yield). ^{1}H NMR (300 MHz, $CDCl_3$): δ (ppm) = 8.47 (d, *J* = 8.6 Hz, 2H), 8.00 (s, 1H), 7.78 – 7.73 (m, 2H), 7.71 (d, *J* = 8.6 Hz, 2H), 7.51 – 7.47 (m, 3H). ^{13}C NMR (75 MHz, $CDCl_3$): δ (ppm) = 148.9 (C), 134.5 (C), 132.7 (CH), 132.3 (2CH), 130.6 (CH), 129.4 (2CH), 128.9 (2CH), 121.7 (2CH), 118.5 (C), 113.3 (C). The spectroscopic data were matched with the one reported in the literature.[7]

***N*-(4-Ethynylbenzylidene)aniline oxide (1l)**

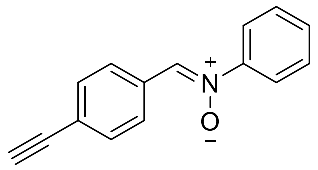

Synthetized from 4-ethynylbenzaldehyde (390 mg, 3 mmol) and *N*-phenylhydroxylamine (327 mg, 3 mmol). Purified by flash chromatography on silica gel (0.1% MeOH/CH_2Cl_2, yellow solid, 144 mg, 22% yield). ^{1}H NMR (300 MHz, $CDCl_3$): δ (ppm) = 8.35 (d, *J* = 8.6 Hz, 2H), 7.92 (s, 1H), 7.78 – 7.74 (m, 2H), 7.57 (d, *J* = 8.6 Hz, 2H), 7.50 – 7.45 (m, 3H), 3.23 (s, 1H). ^{13}C NMR (75 MHz, $CDCl_3$): δ (ppm) = 149.0 (C), 133.7 (CH), 132.3 (2CH), 130.9 (C), 130.1 (CH), 129.2 (2CH), 128.7 (2CH), 124.3 (C), 121.7 (2CH), 83.4 (C), 79.6 (CH). The spectroscopic data were matched with the one reported in the literature.[9]

***N*-[4-(4,4,5,5-Tetramethyl-1,3,2-dioxaborolan-2-yl)benzylidene]aniline oxide (1m)**

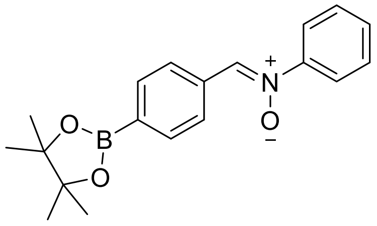

Synthetized from 4-formylbenzeneboronic acid pinacol ester (696 mg, 3 mmol) and *N*-phenylhydroxylamine (327 mg, 3 mmol). Purified by recrystallization from hot EtOAc/*n*-Hexane (white solid, 549 mg, 57% yield). ^{1}H NMR (300 MHz, $CDCl_3$): δ (ppm) = 8.36 (d, *J* = 8.3 Hz, 2H), 7.94 (s, 1H), 7.90 (d, *J* = 8.3 Hz, 2H), 7.80 – 7.74 (m, 2H), 7.51 – 7.43 (m, 3H), 1.36 (s, 12H). ^{13}C NMR (75 MHz, $CDCl_3$): δ (ppm) = 149.1 (C), 135.0 (2CH), 134.6 (CH), 132.9 (C), 130.0 (CH), 129.2 (2CH), 128.0 (2CH), 121.8 (2CH), 84.1 (2C), 24.9 (4CH_3). ^{11}B NMR (129 MHz, $CDCl_3$): δ (ppm) = 30.5. The spectroscopic data were matched with the one reported in the literature.[7]

***N*-(4-Acetylbenzylidene)aniline oxide (1n)**

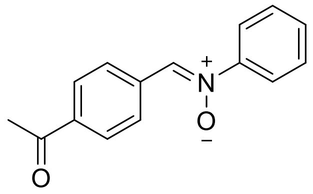

Synthetized from 4-acetylbenzaldehyde (444 mg, 3 mmol) and *N*-phenylhydroxylamine (327 mg, 3 mmol). Purified by recrystallization from hot EtOAc (pale yellow solid, 575 mg, 80% yield). ^{1}H NMR (300 MHz, $CDCl_3$): δ (ppm) = 8.46 (d, *J* = 8.6 Hz, 2H), 8.03 (d, *J* = 8.6 Hz, 2H), 8.00 (s, 1H), 7.79 – 7.75 (m, 2H), 7.51 – 7.46 (m, 3H), 2.63 (s, 3H). ^{13}C NMR (75 MHz, $CDCl_3$): δ (ppm) = 197.3 (C), 149.0 (C), 138.0 (C), 134.6 (C), 133.5 (CH), 130.4 (CH), 129.3 (2CH), 128.9 (2CH), 128.6 (2CH), 121.7 (2CH), 26.8 (CH_3). HMRS (ESI$^+$, m/z) calculated for $(C_{15}H_{14}NO_2)^+$ [$(M+H)^+$]: 240.1019; found: 240.1018.

***N*-[4-(Methoxycarbonyl)benzylidene]aniline oxide (1o)**

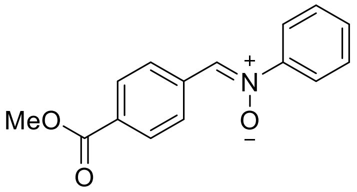

Synthetized from methyl 4-formylbenzoate (985 mg, 6 mmol) and *N*-phenylhydroxylamine (655 mg, 6 mmol). Purified by recrystallization from hot EtOAc (white solid, 1.30 g, 85% yield). ^{1}H NMR (300 MHz, $CDCl_3$): δ (ppm) = 8.43 (d, *J* = 8.6 Hz, 2H), 8.11 (d, *J* = 8.7 Hz, 2H), 7.99 (s, 1H), 7.79 – 7.73 (m, 2H), 7.52 – 7.45 (m, 3H), 3.92 (s, 3H). ^{13}C NMR (75 MHz, $CDCl_3$): δ (ppm) = 166.4 (C), 149.0 (C), 134.5 (C), 133.6 (CH), 131.5 (C), 130.3 (CH), 129.8 (2CH), 129.3 (2CH), 128.6 (2CH), 121.7 (2CH), 52.3 (CH_3). The spectroscopic data were matched with the one reported in the literature.[7]

***N*-[4-(Isopropylcarbamoyl)benzylidene]aniline oxide (1p)**

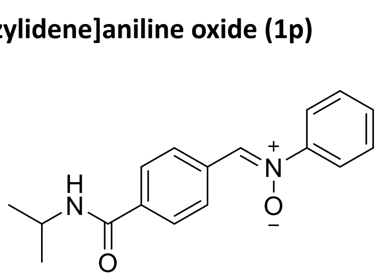

Synthetized from 4-formyl-*N*-isopropylbenzamide (287 mg, 1.5 mmol) and *N*-phenylhydroxylamine (164 mg, 1.5 mmol). Purified by recrystallization from hot EtOAc (white

solid, 313 mg, 74% yield). ^{1}H NMR (300 MHz, $CDCl_3$): δ (ppm) = 8.43 (d, *J* = 8.6 Hz, 2H), 7.97 (s, 1H), 7.85 (d, *J* = 8.6 Hz, 2H), 7.80 – 7.73 (m, 2H), 7.53 – 7.46 (m, 3H), 6.14 (d, *J* = 7.6 Hz, 1H), 4.31 (hept, *J* = 6.6 Hz, 1H), 1.28 (d, *J* = 6.5 Hz, 6H). ^{13}C NMR (75 MHz, $CDCl_3$): δ (ppm) = 165.8 (C), 149.0 (C), 136.4 (C), 133.7 (CH), 133.1 (C), 130.3 (CH), 129.3 (2CH), 128.9 (2CH), 127.2 (2CH), 121.7 (2CH), 42.1, 22.8 ($2CH_3$). HMRS (ESI$^+$, m/z) calculated for $(C_{17}H_{19}N_2O_2)^+$ [$(M+H)^+$]: 283.1441; found: 283.1438.

N-[4-(Pyrrolidine-1-carbonyl)benzylidene]aniline oxide (1q)

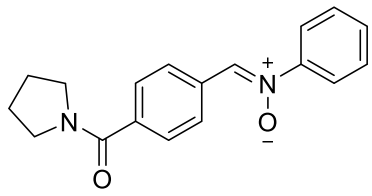

Synthetized from 4-(pyrrolidine-1-carbonyl)benzaldehyde (203 mg, 1 mmol) and *N*-phenylhydroxylamine (109 mg, 1 mmol). Purified by recrystallization from hot EtOAc/*n*-Hexane (white solid, 177 mg, 60% yield). ^{1}H NMR (300 MHz, $CDCl_3$): δ (ppm) = 8.40 (d, *J* = 8.3 Hz, 2H), 7.95 (s, 1H), 7.79 - 7.68 (m, 2H), 7.58 (d, *J* = 8.3 Hz, 2H), 7.50 – 7.37 (m, 3H), 3.61 (t, *J* = 7.1 Hz, 2H), 3.40 (t, *J* = 6.7 Hz, 2H), 2.00 – 1.77 (m, 4H). ^{13}C NMR (75 MHz, $CDCl_3$): δ (ppm) = 168.8 (C), 149.0 (C), 139.0 (C), 133.8 (CH), 131.8 (C), 130.2 (CH), 129.2 (2CH), 128.8 (2CH), 127.5 (2CH), 121.7 (2CH), 49.5 (CH_2), 46.3 (CH_2), 26.4 (CH_2), 24.4 (CH_2). HMRS (ESI$^+$, m/z) calculated for $(C_{18}H_{19}N_2O_2)^+$ [$(M+H)^+$]: 295.1441; found: 295.1440.

N-(Pyridin-3-ylmethylidene)aniline oxide (1x)

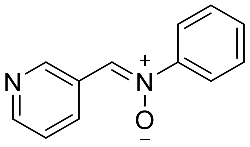

Synthetized from 3-pyridinecarboxaldehyde (283 µL, 3 mmol) and *N*-phenylhydroxylamine (327 mg, 3 mmol). Purified by recrystallization from hot EtOAc (brownish solid, 374 mg, 63% yield). ^{1}H NMR (300 MHz, $CDCl_3$): δ (ppm) = 9.16 (d, *J* = 8.8 Hz, 1H), 9.08 (s, 1H), 8.65- 8.58 (m, 1H), 7.97 (s, 1H), 7.80 – 7.70 (m, 2H), 7.51 – 7.43 (m, 3H), 7.42 – 7.37 (m, 1H). ^{13}C NMR (75 MHz, $CDCl_3$): δ (ppm) = 150.9 (CH), 150.4 (CH), 148.7 (C), 134.8 (CH), 131.5 (CH), 130.4 (CH), 129.3 (2CH), 127.3 (C), 123.7 (CH), 121.6 (2CH). The spectroscopic data were matched with the one reported in the literature.[8]

N-(Naphthalen-1-ylmethylidene)aniline oxide (1y)

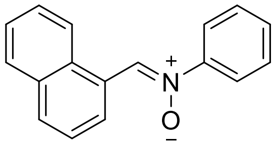

Synthetized from 1-naphthaldehyde (407 µL, 3 mmol) and *N*-phenylhydroxylamine (327 mg, 3 mmol). Purified by recrystallization from hot EtOAc/*n*-Hexane (yellow solid, 381 mg, 51% yield). ^{1}H NMR (300 MHz, $CDCl_3$): δ (ppm) = 9.78 (d, *J* = 7.5 Hz, 1H), 8.71 (s, 1H), 8.10 – 8.07 (m, 1H), 7.96 (d, *J* = 8.3 Hz, 1H), 7.93 – 7.90 (m, 1H), 7.87 – 7.84 (m, 2H), 7.66 – 7.49 (m, 6H). ^{13}C NMR (75 MHz, $CDCl_3$): δ (ppm) = 149.9 (C), 133.6 (C), 131.6 (CH), 130.9 (C), 130.5 (CH), 130.0 (CH), 129.5 (CH), 129.3 (2CH), 127.1 (2CH), 126.0 (CH), 125.9 (CH), 125.8 (C), 122.0 (2CH), 121.7 (CH). The spectroscopic data were matched with the one reported in the literature.[7]

IV.3- Standard procedure for the synthesis of nitrone 1ad.[2]

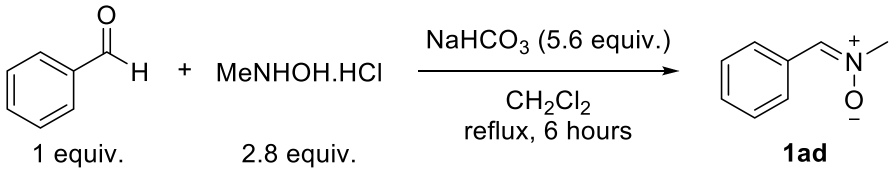

Scheme SI_3: Synthesis of nitrone **1ad**

Benzaldehyde (507 µL, 5 mmol, 1 equiv.) was added to a heterogeneous mixture of *N*-methylhydroxylamine hydrochloride (1.17 g, 14 mmol, 2.8 equiv.) and $NaHCO_3$ (2.35 g, 28 mmol, 5.6 equiv.) in 10 mL of dry CH_2Cl_2. The reaction mixture was stirred to reflux (oil bath) for 6 hours. Then, the organic liquor was decanted and filtered in a short plug of Celite®. Solvents and volatiles were removed under vacuum and the crude was purified by flash chromatography on silica gel (CH_2Cl_2/MeOH 99:1) to afford nitrone **1ad** (605 mg, 90% yield).

***N*-Benzylidenemethylamine oxide (1ad)**

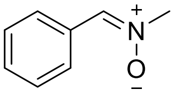

White solid. ^{1}H NMR (300 MHz, $CDCl_3$): δ (ppm) = 8.20 – 8.18 (m, 2H), 7.39 – 7.37 (m, 3H), 7.34 (s, 1H), 3.84 (s, 3H). ^{13}C NMR (75 MHz, $CDCl_3$): δ (ppm) = 135.2 (CH), 130.5 (C), 130.4 (CH), 128.5 (2CH), 128.4 (2CH), 54.4 (CH_3). The spectroscopic data were matched with the one reported in the literature.[15]

IV.4- Standard procedure for the synthesis of nitrones 1ae and 1ah.[3]

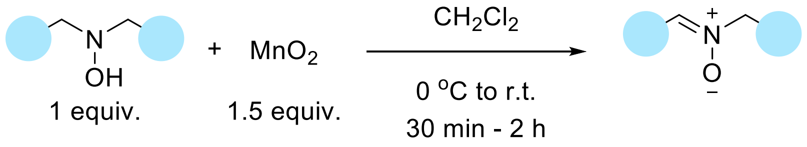

Scheme SI_4: Synthesis of nitrones **1ae** and **1ah**

Activated MnO_2 (≥90%, 1.5 equiv.) was added portion-wise to a solution of the corresponding secondary hydroxylamine (1 equiv.) in CH_2Cl_2 (0.5 M) cooled at 0 °C (ice-bath). Once the addition was completed, the solution was allowed to slowly reach room temperature and stirred until reaction was completed (TLC monitored). Then, the organic liquor was filtered through a cotton, solvents were removed under vacuum and crudes mixtures were purified by flash chromatography on silica gel to afford nitrones **1ae** and **1ah** in moderate yields (45-68%).

_N_-Benzylidenebenzylamine oxide (1ae)

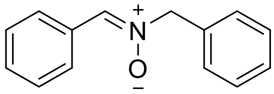

Synthetized from *N*,*N*-dibenzylhydroxylamine (213 mg, 1 mmol). Reaction completed in 30 minutes. Purified by flash chromatography on silica gel (CH_2Cl_2/MeOH 99:1, white solid, 144 mg, 68% yield). ^{1}H NMR (300 MHz, $CDCl_3$): δ (ppm) = 8.24 – 8.18 (m, 2H), 7.51 – 7.38 (m, 9H), 5.06 (s, 2H). ^{13}C NMR (75 MHz, $CDCl_3$): δ (ppm) = 134.3 (CH), 133.2 (C), 130.5 (2CH), 130.4 (C), 129.3 (2CH), 129.0 (2CH), 128.6 (2CH), 128.5 (2CH), 71.3 (CH_2). The spectroscopic data were matched with the one reported in the literature.[15]

_N_-Ethylethanimine oxide (1ah)

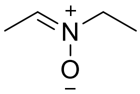

Synthetized from *N*,*N*-diethylhydroxylamine (309 µL, 3 mmol). Reaction completed in 2 hours. Purified by flash chromatography on silica gel (CH_2Cl_2/MeOH 99:1 to 95:5, colourless oil, 118 mg, 45% yield). ^{1}H NMR (300 MHz, $CDCl_3$): δ (ppm) = 6.76 (q, *J* = 5.9 Hz, 1H), 3.76 (q, *J* = 7.3 Hz, 2H), 1.95 (d, *J* = 5.8 Hz, 3H), 1.41 (t, *J* = 7.3 Hz, 3H). ^{13}C NMR (75 MHz, $CDCl_3$): δ (ppm) = 133.5 (CH), 59.9 (CH_2), 13.3 (CH_3), 12.6 (CH_3). The spectroscopic data were matched with the one reported in the literature.[16]

IV.5- Standard procedure for the synthesis of nitrone 1af.[4]

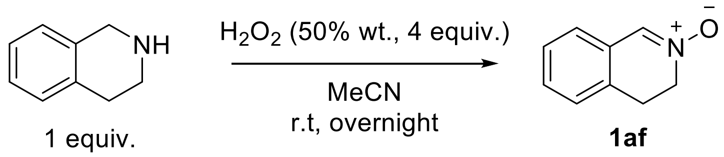

Scheme SI_5: Synthesis of nitrone **1af**

H_2O_2 (50% wt. in H_2O, 1.14 mL, 20 mmol, 4 equiv.) was added dropwise to a solution of 1,2,3,4-tetrahydroisoquinoline (626 µL, 5 mmol, 1 equiv.) in MeCN (15 mL) and the reaction mixture was stirred overnight at room temperature. Then, MeCN was removed under vacuum and the resulting mixture was extracted using 50 mL of brine and 50 mL of CH_2Cl_2. The aqueous phase was washed with CH_2Cl_2 (2 x 50 mL), the organic layers were combined, dried with $MgSO_4$ and filtered. Solvent was removed under vacuum and the crude was purified by flash chromatography on silica gel (CH_2Cl_2/MeOH 99:1 to 95:5) to afford nitrone **1af** (258 mg, 35% yield).

3,4-Dihydroisoquinoline *N*-Oxide (1af)

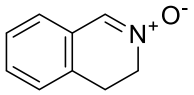

Brownish oil. ^{1}H NMR (300 MHz, $CDCl_3$): δ (ppm) = 7.75 (s, 1H), 7.30 – 7.19 (m, 3H), 7.14 – 7.09 (m, 1H), 4.10 (t, *J* = 7.8 Hz, 2H), 3.18 (t, *J* = 7.8 Hz, 2H). ^{13}C NMR (75 MHz, $CDCl_3$): δ (ppm) = 134.1 (CH), 130.1 (C), 129.4 (CH), 128.4 (C), 127.7 (CH), 127.3 (CH), 125.5 (CH), 58.0 (CH_2), 27.8 (CH_2). The spectroscopic data were matched with the one reported in the literature.[17]

IV.6- Standard procedure for the synthesis of nitrone 1ag.[5]

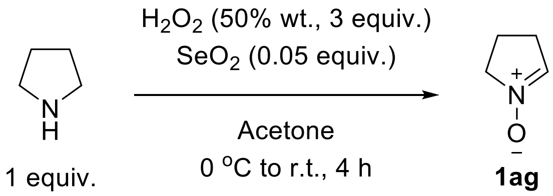

Scheme SI_6: Synthesis of nitrone **1ag**

Under argon atmosphere, pyrrolidine (822 µL, 10 mmol, 1 equiv.) was added to a solution of SeO_2 (56 mg, 0.5 mmol, 0.05 equiv.) in 20 mL of deoxygenated acetone and the reaction vessel was rapidly closed-cup. The mixture was stirred 5 min at room temperature, cooled to 0 °C (ice-bath) and H_2O_2 (50% wt. in H_2O, 1.71 mL, 30 mmol, 3 equiv.) was added dropwise for 10 minutes. Then, the reaction mixture was stirred at 0 °C for 1 hour, allowed to slowly reach room temperature and stirred for 3 hours more. Once the reaction was completed, acetone was removed under vacuum and the resulting mixture was extracted with 50 mL of brine and 50 mL of CH_2Cl_2. The aqueous phase was washed with CH_2Cl_2 (2 x 50 mL), the organic layers were combined, dried with $MgSO_4$ and filtered. Solvent and volatiles were removed under vacuum and the crude was purified by flash chromatography on silica gel (CH_2Cl_2/MeOH 97:3 to 95:5) to afford nitrone **1ag** (31 mg, 4% yield).

3,4-Dihydropyrrole *N*-Oxide (1ag)

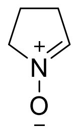

Yellow oil. ^1H NMR (300 MHz, $CDCl_3$): δ (ppm) = 6.87 – 6.84 (m, 1H), 3.95 – 3.87 (m, 2H), 2.72 – 2.64 (m, 2H), 2.25 – 2.14 (m, 2H). ^{13}C NMR (75 MHz, $CDCl_3$): δ (ppm) = 135.7 (CH), 62.0 (CH_2), 28.7 (CH_2), 19.0 (CH_2). The spectroscopic data were matched with the one reported in the literature.[18]

V.- General procedures for the synthesis of thioamides 3a-3ah.

V.1.- Standard procedure for the synthesis of thioamides 3a-3ah and spectroscopic data.

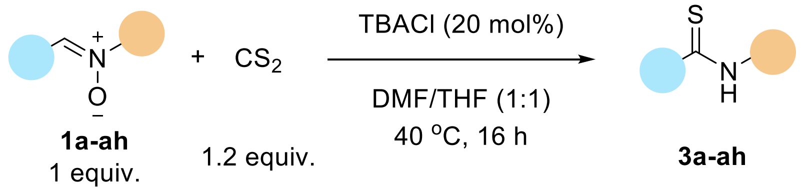

Scheme SI_7: Synthesis of thioamides **3a-3ah**

To a solution of tetrabutylammonium chloride (22 mg, 0.08 mmol, 0.2 equiv.) and the corresponding nitrone **1a-ah** (0.4 mmol, 1 equiv.) in 2 mL of a mixture of dry DMF and dry THF (1:1) in a 10 mL high-pressure close-capped tube, CS_2 (29 µL, 0.48 mmol, 1.2 equiv.) was added under argon. The resulting mixture was stirred for 16 hours at 40 °C (oil bath). Then, the crude was extracted with EtOAc (20 mL) and distilled water (20 mL), the organic phase dried with $MgSO_4$, filtered and solvent and volatiles were removed under vacuum. Finally, the crude was purified by flash chromatography on silica gel to afford secondary thioamides **3a-ah** (12-95% yield).

In the case of nitrones **1j-k**, **1v-w** and **1ad-ae** an analogous procedure was followed using 3 equiv. of CS_2 (72 µL, 1.2 mmol) and stirring the reaction mixture for 40 hours to afford thioamides **3j-k**, **3v-w** and **3ad-ae** in higher yields (60-90%).

4-Chloro-*N*-phenylbenzothioamide (3a)

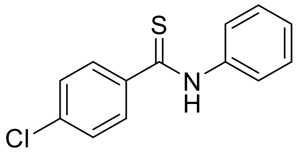

Yellow solid (73 mg, 74% yield, purified in *n*-Hexane/EtOAc 5:1). ^{1}H NMR (300 MHz, $CDCl_3$): δ (ppm) = 8.98 (*br* s, 1H), 7.81 – 7.73 (m, 4H), 7.48 – 7.39 (m, 4H), 7.31 (t, *J* = 7.6 Hz, 1H). ^{13}C NMR (75 MHz, $CDCl_3$): δ (ppm) = 196.9 (C), 141.4 (C), 138.8 (C), 137.6 (C), 129.2 (2CH), 128.8 (2CH), 128.1 (2CH), 127.2 (CH), 123.8 (2CH). The spectroscopic data were matched with the one reported in the literature.[19]

***N*-Phenylbenzothioamide (3b)**

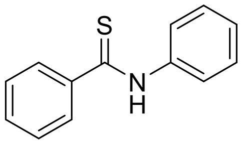

Yellow solid (65 mg, 76% yield, purified in *n*-Hexane/EtOAc 5:1). ^{1}H NMR (300 MHz, $CDCl_3$): δ (ppm) = 9.03 (*br* s, 1H), 7.85 (d, *J* = 7.7 Hz, 2H), 7.77 (d, *J* = 8.1 Hz, 2H), 7.54 – 7.38 (m, 5H), 7.31 (t, *J* = 7.4 Hz, 1H). ^{13}C NMR (75 MHz, $CDCl_3$): δ (ppm) = 198.5 (C), 143.2 (C), 139.0 (C), 131.3 (CH), 129.1 (2CH), 128.7 (2CH), 127.1 (CH), 126.7 (2CH), 123.7 (2CH). The spectroscopic data were matched with the one reported in the literature.[19]

4-Fluoro-*N*-phenylbenzothioamide (3c)

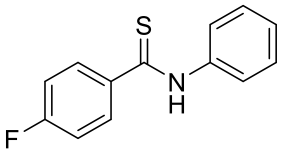

Yellow solid (68 mg, 73% yield, purified in *n*-Hexane/EtOAc 5:1). ^{1}H NMR (300 MHz, $CDCl_3$): δ (ppm) = 9.00 (*br* s, 1H), 7.89 – 7.84 (m, 2H), 7.73 (d, *J* = 8.1 Hz, 2H), 7.44 (t, *J* = 7.8 Hz, 2H), 7.31 (t, *J* = 7.3 Hz, 1H), 7.10 (t, *J* = 8.4 Hz, 2H). ^{19}F NMR (282 MHz, $CDCl_3$): δ (ppm) = -108.4. ^{13}C NMR (75 MHz, $CDCl_3$): δ (ppm) = 197.1 (C), 164.6 (d, *J* = 254.3 Hz, C), 139.3 (C), 138.9 (C), 129.2 (2CH), 129.0 (2CH), 127.2 (CH), 123.9 (2CH), 115.6 (d, *J* = 22.3 Hz, 2CH). The spectroscopic data were matched with the one reported in the literature.[20]

4-Bromo-*N*-phenylbenzothioamide (3d)

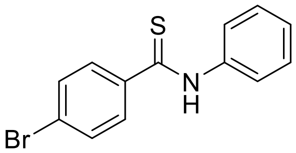

Yellow solid (83 mg, 71% yield, purified in *n*-Hexane/EtOAc 5:1). Melting point: 168 – 170 °C. R_f (*n*-Hexane/EtOAc 5:1) = 0.51. ^{1}H NMR (300 MHz, $CDCl_3$): δ (ppm) = 8.98 (*br* s, 1H), 7.75 – 7.71 (m, 4H), 7.56 (d, *J* = 8.5 Hz, 2H), 7.45 (t, *J* = 7.8 Hz, 2H), 7.31 (t, *J* = 7.4 Hz, 1H). ^{13}C NMR (75 MHz, $CDCl_3$): δ (ppm) = 197.0 (C), 141.8 (C), 138.8 (C), 131.8 (2CH), 129.2 (2CH), 128.2 (2CH), 127.2 (CH), 126.0 (C), 123.7 (2CH). HMRS (ESI$^+$, m/z) calculated for $(C_{13}H_{11}BrNS)^+$ $[(M+H)^+]$: 291.9790; found: 291.9782.

4-Iodo-*N*-phenylbenzothioamide (3e)

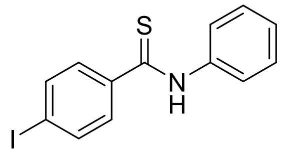

Yellow solid (99 mg, 73% yield, purified in *n*-Hexane/EtOAc 5:1). Melting point: 186 – 188 °C. R_f (*n*-Hexane/EtOAc 5:1) = 0.54. ^{1}H NMR (300 MHz, $CDCl_3$): δ (ppm) = 8.98 (*br* s, 1H), 7.79 – 7.73 (m, 4H), 7.57 (d, *J* = 8.3 Hz, 2H), 7.45 (t, *J* = 7.8 Hz, 2H), 7.31 (t, *J* = 7.4 Hz, 1H). ^{13}C NMR (75 MHz, $CDCl_3$): δ (ppm) = 197.2 (C), 142.4 (C), 138.8 (C), 137.8 (2CH), 129.2 (2CH), 128.2 (2CH), 127.2 (CH), 123.7 (2CH), 98.2 (C). HMRS (ESI$^+$, m/z) calculated for $(C_{13}H_{11}INS)^+$ [$(M+H)^+$]: 339.9651; found: 339.9642.

3-Chloro-*N*-phenylbenzothioamide (3f)

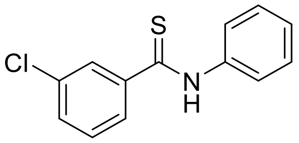

Yellow solid (70 mg, 71% yield, purified in *n*-Hexane/EtOAc 5:1). ^{1}H NMR (300 MHz, $CDCl_3$): δ (ppm) = 9.03 (*br* s, 1H), 7.82 (s, 1H), 7.76 – 7.68 (m, 3H), 7.45 (t, *J* = 7.9 Hz, 3H), 7.39 – 7.29 (m, 2H). ^{13}C NMR (75 MHz, $CDCl_3$): δ (ppm) = 196.6 (C), 144.6 (C), 138.7 (C), 134.7 (C), 131.2 (CH), 129.9 (CH), 129.2 (2CH), 127.3 (CH), 127.0 (CH), 124.8 (CH), 123.7 (2CH). The spectroscopic data were matched with the one reported in the literature.[21]

2-Chloro-*N*-phenylbenzothioamide (3g)

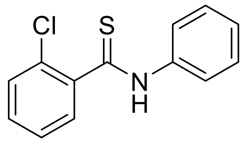

Pale yellow solid (34 mg, 34% yield, purified in *n*-Hexane/EtOAc 5:1). Melting point: 150 – 152 °C. R_f (*n*-Hexane/EtOAc 5:1) = 0.28. ^{1}H NMR (300 MHz, DMSO-d^6): δ (ppm) = 12.07 (*br* s, 1H), 7.92 (d, *J* = 8.3 Hz, 2H), 7.53 – 7.37 (m, 6H), 7.27 (t, *J* = 7.4 Hz, 1H). ^{13}C NMR (75 MHz, DMSO-d^6): δ (ppm) = 195.1 (C), 143.8 (C), 139.8 (C), 130.4 (CH), 129.9 (CH), 129.4 (CH), 129.1 (2CH), 128.6 (C), 127.6 (CH), 126.9 (CH), 123.4 (2CH). HMRS (ESI$^+$, m/z) calculated for $(C_{13}H_{11}ClNS)^+$ [$(M+H)^+$]: 248.0295; found: 248.0293.

3,4-Dichloro-*N*-phenylbenzothioamide (3h)

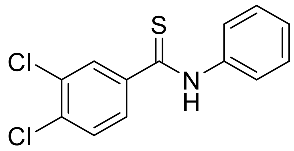

Yellow solid (77 mg, 68% yield, purified in *n*-Hexane/EtOAc 5:1). Melting point: 138 – 140 °C. R_f (*n*-Hexane/EtOAc 5:1) = 0.43. ^{1}H NMR (300 MHz, $CDCl_3$): δ (ppm) = 8.98 (*br* s, 1H), 7.93 (s, 1H), 7.74 – 7.66 (m, 3H), 7.51 – 7.42 (m, 3H), 7.32 (t, *J* = 7.3 Hz, 1H). ^{13}C NMR (75 MHz, $CDCl_3$): δ (ppm) = 195.3 (C), 142.5 (C), 138.6 (C), 135.6 (C), 133.0 (C), 130.5 (CH), 129.2 (2CH), 128.7 (CH), 127.4 (CH), 125.9 (CH), 123.7 (2CH). HMRS (ESI$^+$, m/z) calculated for $(C_{13}H_{10}Cl_2NS)^+$ $[(M+H)^+]$: 281.9906; found: 281.9900.

***N*-Phenyl-4-(trifluoromethyl)benzothioamide (3i)**

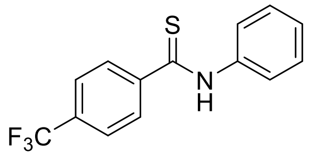

Yellow solid (76 mg, 68% yield, purified in *n*-Hexane/EtOAc 5:1). ^{1}H NMR (300 MHz, $CDCl_3$): δ (ppm) = 9.02 (*br* s, 1H), 7.93 (d, *J* = 8.2 Hz, 2H), 7.77 (d, *J* = 7.9 Hz, 2H), 7.70 (d, *J* = 8.2 Hz, 2H), 7.47 (t, *J* = 7.8 Hz, 2H), 7.34 (d, *J* = 7.6 Hz, 1H). ^{19}F NMR (282 MHz, $CDCl_3$): δ (ppm) = -62.9. ^{13}C NMR (75 MHz, $CDCl_3$): δ (ppm) = 196.7 (C), 146.2 (C), 138.6 (C), 132.8 (q, *J* = 33.3 Hz, C), 129.2 (2CH), 127.4 (CH), 127.1 (2CH), 125.8 (d, *J* = 3.4 Hz, 2CH), 123.7 (q, *J* = 270.1 Hz, C), 123.6 (2CH). The spectroscopic data were matched with the one reported in the literature.[21]

4-Nitro-*N*-phenylbenzothioamide (3j)

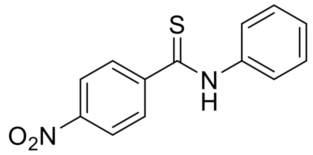

Orangish solid (56 mg, 54% yield using 1.2 equiv. CS_2 for 16 h; 86 mg, 83% yield using 3 equiv. CS_2 for 40 h, purified in *n*-Hexane/CH_2Cl_2 1:1). ^{1}H NMR (300 MHz, $CDCl_3$): δ (ppm) = 9.16 (*br* s, 1H), 8.26 (d, *J* = 8.6 Hz, 2H), 7.95 (d, *J* = 8.6 Hz, 2H), 7.78 (d, *J* = 8.1 Hz, 2H), 7.47 (t, *J* = 7.6 Hz, 2H), 7.34 (t, *J* = 7.5 Hz, 1H). ^{13}C NMR (75 MHz, $CDCl_3$): δ (ppm) = 195.5 (C), 148.9 (C), 148.2 (C), 138.5 (C), 129.2 (2CH), 127.7 (2CH), 127.5 (CH), 123.9 (2CH), 123.6 (2CH). The spectroscopic data were matched with the one reported in the literature.[22]

4-Cyano-*N*-phenylbenzothioamide (3k)

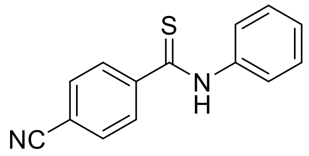

Yellow solid (58 mg, 61% yield using 1.2 equiv. CS_2 for 16 h; 83 mg, 87% yield using 3 equiv. CS_2 for 40 h, purified in *n*-Hexane/EtOAc 5:1). ^1H NMR (300 MHz, $CDCl_3$): δ (ppm) = 9.11 (*br* s, 1H), 7.91 (d, *J* = 8.3 Hz, 2H), 7.76 (d, *J* = 8.0 Hz, 2H), 7.70 (d, *J* = 8.4 Hz, 2H), 7.46 (t, *J* = 7.8 Hz, 2H), 7.33 (t, *J* = 7.3 Hz, 1H). ^{13}C NMR (75 MHz, $CDCl_3$): δ (ppm) = 195.9 (C), 146.6 (C), 138.6 (C), 132.5 (2CH), 129.2 (2CH), 127.5 (CH), 127.4 (2CH), 123.6 (2CH), 118.1 (C), 114.3 (C). The spectroscopic data were matched with the one reported in the literature.[21]

4-Ethynyl-*N*-phenylbenzothioamide (3l)

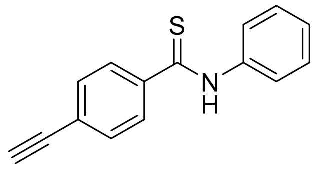

Yellow solid (66 mg, 70% yield, purified in *n*-Hexane/EtOAc 5:1). Melting point: 156 – 158 °C. R_f (*n*-Hexane/EtOAc 5:1) = 0.44. ^1H NMR (300 MHz, $CDCl_3$): δ (ppm) = 9.00 (*br* s, 1H), 7.82 – 7.74 (m, 4H), 7.54 (d, *J* = 8.1 Hz, 2H), 7.44 (t, *J* = 7.6 Hz, 2H), 7.30 (t, *J* = 7.5 Hz, 1H), 3.23 (s, 1H). ^{13}C NMR (75 MHz, $CDCl_3$): δ (ppm) = 197.2 (C), 142.9 (C), 138.9 (C), 132.3 (2CH), 129.1 (2CH), 127.1 (CH), 126.7 (2CH), 125.1 (C), 123.7 (2CH), 82.7 (C), 79.8 (CH). HMRS (ESI^+, m/z) calculated for $(C_{15}H_{12}NS)^+$ $[(M+H)^+]$: 238.0685; found: 238.0686.

***N*-Phenyl-4-(4,4,5,5-tetramethyl-1,3,2-dioxaborolan-2-yl)benzothioamide (3m)**

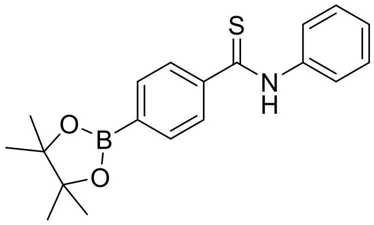

Yellow solid (81 mg, 60% yield, purified in *n*-Hexane/EtOAc 5:1). Melting point: 128 – 130 °C. R_f (*n*-Hexane/EtOAc 5:1) = 0.36. ^1H NMR (300 MHz, $CDCl_3$): δ (ppm) = 9.04 (*br* s, 1H), 7.90 – 7.76 (m, 6H), 7.45 (t, *J* = 7.8 Hz, 2H), 7.30 (t, *J* = 7.1 Hz, 1H), 1.36 (s, 12H). ^{13}C NMR (75 MHz, $CDCl_3$): δ (ppm) = 198.3 (C), 145.3 (C), 139.0 (C), 135.1 (2CH), 129.1 (2CH), 127.1 (CH), 125.8 (2CH), 123.7

(2CH), 84.2 (2C), 24.9 (4CH_3). ^{11}B NMR (129 MHz, $CDCl_3$): δ (ppm) = 30.7. HMRS (ESI$^+$, m/z) calculated for $(C_{19}H_{23}BNO_2S)^+$ [(M+H)$^+$]: 340.1537; found: 340.1534.

4-Acetyl-*N*-phenylbenzothioamide (3n)

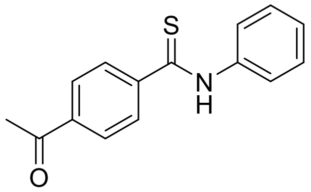

Yellow solid (66 mg, 65% yield, purified in *n*-Hexane/EtOAc 4:1). Melting point: 170 – 172 °C. R_f (*n*-Hexane/EtOAc 5:1) = 0.11. ^{1}H NMR (300 MHz, $CDCl_3$): δ (ppm) = 9.21 (*br* s, 1H), 7.96 (d, *J* = 8.4 Hz, 2H), 7.89 (d, *J* = 8.3 Hz, 2H), 7.80 (d, *J* = 8.2 Hz, 2H), 7.46 (t, *J* = 7.8 Hz, 2H), 7.32 (t, *J* = 7.1 Hz, 1H), 2.62 (s, 3H). ^{13}C NMR (75 MHz, $CDCl_3$): δ (ppm) = 197.5 (C), 197.0 (C), 146.8 (C), 138.8 (C), 138.6 (C), 129.2 (2CH), 128.6 (2CH), 127.3 (CH), 127.0 (2CH), 123.6 (2CH), 26.9 (CH_3). HMRS (ESI$^+$, m/z) calculated for $(C_{15}H_{14}NOS)^+$ [(M+H)$^+$]: 256.0791; found: 256.0788.

4-(Methoxycarbonyl)-*N*-phenylbenzothioamide (3o)

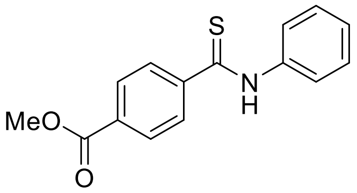

Orangish solid (80 mg, 74% yield, purified in *n*-Hexane/EtOAc 5:1). Melting point: 168 – 170 °C. R_f (*n*-Hexane/EtOAc 5:1) = 0.31. ^{1}H NMR (300 MHz, $CDCl_3$): δ (ppm) = 9.16 (*br* s, 1H), 8.05 (d, *J* = 8.3 Hz, 2H), 7.86 (d, *J* = 8.3 Hz, 2H), 7.79 (d, *J* = 8.0 Hz, 2H), 7.46 (t, *J* = 7.8 Hz, 2H), 7.32 (t, *J* = 7.3 Hz, 1H), 3.94 (s, 3H). ^{13}C NMR (75 MHz, $CDCl_3$): δ (ppm) = 197.2 (C), 166.3 (C), 146.8 (C), 138.8 (C), 132.1 (C), 129.9 (2CH), 129.2 (2CH), 127.3 (CH), 126.7 (2CH), 123.6 (2CH), 52.5 (CH_3). HMRS (ESI$^+$, m/z) calculated for $(C_{15}H_{14}NO_2S)^+$ [(M+H)$^+$]: 272.0740; found: 272.0736.

4-(Isopropylcarbamoyl)-*N*-phenylbenzothioamide (3p)

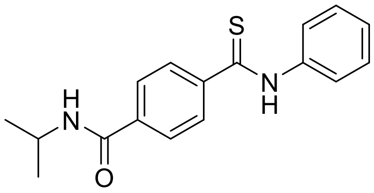

Yellow solid (91 mg, 76% yield, purified in *n*-Hexane/EtOAc 1:1). Melting point: 188 – 190 °C. R_f (*n*-Hexane/EtOAc 1:1) = 0.29. ^{1}H NMR (300 MHz, DMSO-d^6): δ (ppm) = 11.85 (*br* s, 1H), 8.35 (d, *J* = 7.8 Hz, 1H), 7.92 – 7.84 (m, 4H), 7.81 (d, *J* = 7.8 Hz, 2H), 7.44 (t, *J* = 7.8 Hz, 2H), 7.28 (t, *J* = 7.4 Hz, 1H), 4.10 (hept, *J* = 6.8 Hz, 1H), 1.17 (d, *J* = 6.6 Hz, 6H). ^{13}C NMR (75 MHz, DMSO-d^6): δ (ppm) = 197.2 (C), 165.1 (C), 144.9 (C), 140.4 (C), 136.9 (C), 129.0 (2CH), 127.7 (2CH), 127.4 (2CH), 126.9 (CH), 124.7 (2CH), 41.6 (CH), 22.8 (2CH_3). HMRS (ESI$^+$, m/z) calculated for $(C_{17}H_{19}N_2OS)^+$ [$(M+H)^+$]: 299.1213; found: 299.1208.

***N*-Phenyl-4-(pyrrolidine-1-carbonyl)benzothioamide (3q)**

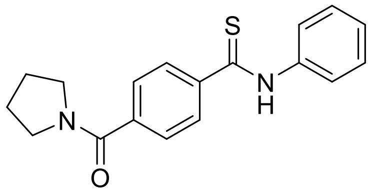

Yellow solid (102 mg, 82% yield, purified in *n*-Hexane/EtOAc/CH_2Cl_2 2:1:1). Melting point: 205 – 207 °C. R_f (CH_2Cl_2/MeOH 98:2) = 0.18. ^{1}H NMR (300 MHz, $CDCl_3$): δ (ppm) = 10.29 (*br* s, 1H), 7.92 (d, *J* = 8.0 Hz, 2H), 7.81 (d, *J* = 8.3 Hz, 2H), 7.46 (t, *J* = 7.8 Hz, 2H), 7.35 – 7.28 (m, 3H), 3.57 (t, *J* = 6.9 Hz, 2H), 3.37 (t, *J* = 6.5 Hz, 2H), 2.99 – 1.82 (m, 4H). ^{13}C NMR (75 MHz, $CDCl_3$): δ (ppm) = 197.4 (C), 168.9 (C), 144.3 (C), 139.4 (C), 138.8 (C), 128.9 (2CH), 127.1 (2CH), 127.0 (2CH), 126.9 (CH), 123.9 (2CH), 49.6 (CH_2), 46.4 (CH_2), 26.3 (CH_2), 24.4 (CH_2). HMRS (ESI$^+$, m/z) calculated for $(C_{18}H_{19}N_2OS)^+$ [$(M+H)^+$]: 311.1213; found: 311.1208.

4-Methyl-*N*-phenylbenzothioamide (3r)

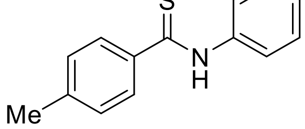

Yellow solid (64 mg, 70% yield, purified in *n*-Hexane/EtOAc 5:1). ^{1}H NMR (300 MHz, $CDCl_3$): δ (ppm) = 9.01 (*br* s, 1H), 7.83 – 7.70 (m, 4H), 7.44 (t, *J* = 7.9 Hz, 2H), 7.32 – 7.22 (m, 3H), 2.40 (s, 3H). ^{13}C NMR (75 MHz, $CDCl_3$): δ (ppm) = 198.3 (C), 142.0 (C), 140.4 (C), 139.1 (C), 129.3 (2CH),

129.1 (2CH), 126.9 (CH), 126.8 (2CH), 123.8 (2CH), 21.4 (CH_3). The spectroscopic data were matched with the one reported in the literature.[21]

4-Hydroxy-*N*-phenylbenzothioamide (3s)

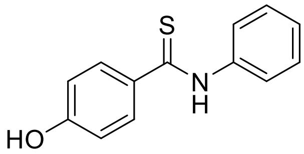

Yellow solid (11 mg, 12% yield, purified in *n*-Hexane/EtOAc 2:1). ^{1}H NMR (300 MHz, DMSO-d^6): δ (ppm) = 11.41 (*br* s, 1H), 10.10 (*br* s, 1H), 7.81 – 7.71 (m, 4H), 7.45 – 7.35 (m, 2H), 7.27 – 7.19 (m, 1H), 6.80 (d, *J* = 8.1 Hz, 2H). ^{13}C NMR (75 MHz, DMSO-d^6): δ (ppm) = 197.1 (C), 160.9 (C), 140.8 (C), 133.5 (C), 130.2 (2CH), 128.9 (2CH), 126.4 (CH), 125.0 (2CH), 115.0 (2CH). The spectroscopic data were matched with the one reported in the literature.[23]

4-Methoxy-*N*-phenylbenzothioamide (3t)

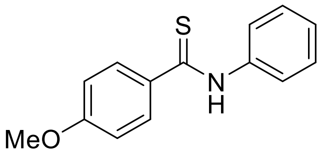

Yellow solid (71 mg, 73% yield, purified in *n*-Hexane/EtOAc 4:1). ^{1}H NMR (300 MHz, $CDCl_3$): δ (ppm) = 8.95 (*br* s, 1H), 7.92 – 7.81 (m, 2H), 7.78 – 7.68 (m, 2H), 7.43 (t, *J* = 7.7 Hz, 2H), 7.28 (t, *J* = 7.3 Hz, 1H), 6.92 (d, *J* = 7.8 Hz, 2H), 3.86 (s, 3H). ^{13}C NMR (75 MHz, $CDCl_3$): δ (ppm) = 197.5 (C), 162.3 (C), 139.2 (C), 135.3 (C), 129.1 (2CH), 128.7 (2CH), 126.8 (CH), 123.9 (2CH), 113.8 (2CH), 55.6 (CH_3). The spectroscopic data were matched with the one reported in the literature.[24]

4-Acetamido-*N*-phenylbenzothioamide (3u)

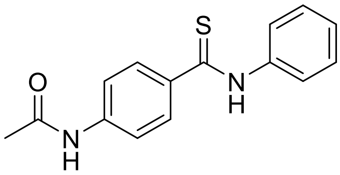

Yellow solid (38 mg, 35% yield, purified in *n*-Hexane/EtOAc 1:3). Melting point: 221 – 223 ^{o}C. R_f (*n*-Hexane/EtOAc 1:1) = 0.17. ^{1}H and ^{13}C NMR show mixture of rotamers (approximately 1:1). ^{1}H NMR (300 MHz, DMSO-d^6): δ (ppm) = 11.57 (*br* s, 1H), 10.36 (*br* s, 1H), 10.21 (*br* s, 1H), 9.85 (*br* s, 1H), 7.88 – 7.70 (m, 9H), 7.66 – 7.63 (m, 2H), 7.44 – 7.35 (m, 3H), 7.27 – 7.19 (m, 2H), 2.08 (s, 6H). ^{13}C NMR (75 MHz, DMSO-d^6): δ (ppm) = 197.0, 192.0, 169.6, 169.2, 145.3, 142.3, 140.6,

137.0, 131.6, 131.3, 130.0, 129.6, 129.0, 128.9, 126.6, 124.9, 121.4, 119.1, 119.0, 118.2, 24.7, 24.6. HMRS (ESI$^+$, m/z) calculated for $(C_{15}H_{15}N_2OS)^+$ [$(M+H)^+$]: 271.0900; found: 271.0897.

***N*-Phenylfuran-2-carbothioamide (3v)**

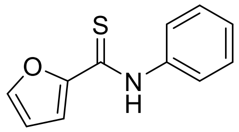

Yellow solid (57 mg, 70% yield using 1.2 equiv. CS_2 for 16 h; 71 mg, 87% yield using 3 equiv. CS_2 for 40 h, purified in *n*-Hexane/EtOAc 5:1). ^{1}H NMR (300 MHz, DMSO-*d*^{6}): δ (ppm) = 11.51 (*br* s, 1H), 7.95 (s, 1H), 7.67 (d, *J* = 7.9 Hz, 2H), 7.40 (t, *J* = 7.8 Hz, 2H), 7.34 (d, *J* = 3.6 Hz, 1H), 7.25 (t, *J* = 7.4 Hz, 1H), 6.68 (dd, *J* = 3.4, 1.7 Hz, 1H). ^{13}C NMR (75 MHz, DMSO-*d*^{6}): δ (ppm) = 181.4 (C), 153.0 (C), 146.1 (CH), 139.4 (C), 128.9 (2CH), 126.8 (CH), 125.5 (2CH), 117.6 (CH), 113.6 (CH). The spectroscopic data were matched with the one reported in the literature.[23]

***N*-Phenylthiophen-2-carbothioamide (3w)**

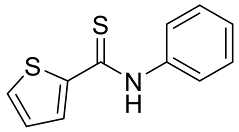

Yellow oil (29 mg, 33% yield using 1.2 equiv. CS_2 for 16 h; 60 mg, 68% yield using 3 equiv. CS_2 for 40 h, purified in *n*-Hexane/EtOAc 5:1). ^{1}H NMR (300 MHz, $CDCl_3$): δ (ppm) = 9.07 (*br* s, 1H), 7.64 (d, *J* = 8.0 Hz, 2H), 7.54 – 7.51 (m, 2H), 7.40 (t, *J* = 7.8 Hz, 2H), 7.30 – 7.25 (m, 1H), 7.07 (t, *J* = 4.4 Hz, 1H). ^{13}C NMR (75 MHz, $CDCl_3$): δ (ppm) = 187.8 (C), 148.0 (C), 138.5 (C), 133.2 (CH), 129.1 (2CH), 128.0 (CH), 127.1 (CH), 124.7 (CH), 124.4 (2CH). The spectroscopic data were matched with the one reported in the literature.[25]

***N*-Phenylpyridin-3-carbothioamide (3x)**

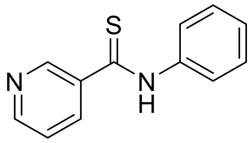

Yellow solid (60 mg, 70% yield, purified in *n*-Hexane/EtOAc 1:1). Melting point: 126 – 128 ^{o}C. R$_f$ (*n*-Hexane/EtOAc 1:1) = 0.23. ^{1}H NMR (300 MHz, $CDCl_3$): δ (ppm) = 9.87 (*br* s, 1H), 8.88 (s, 1H), 8.59 (d, *J* = 5.0 Hz, 1H), 8.21 (d, *J* = 8.0 Hz, 1H), 7.78 (d, *J* = 8.0 Hz, 2H), 7.46 (t, *J* = 7.8 Hz, 2H), 7.37 – 7.30 (m, 2H). ^{13}C NMR (75 MHz, $CDCl_3$): δ (ppm) = 195.3 (C), 151.4 (CH), 146.2 (CH), 139.0

(C), 138.9 (C), 135.9 (CH), 129.2 (2CH), 127.3 (CH), 123.8 (2CH), 123.6 (CH). HMRS (ESI$^+$, m/z) calculated for $(C_{12}H_{11}N_2S)^+$ [(M+H)$^+$]: 215.0637; found: 215.0647.

_N_-Phenylnaphthalen-1-carbothioamide (3y)

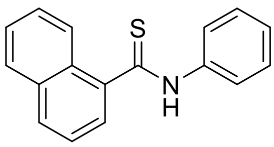

Yellow solid (62 mg, 59% yield, purified in *n*-Hexane/EtOAc 5:1). Melting point: 109 – 111 °C. R_f (*n*-Hexane/EtOAc 5:1) = 0.44. ^{1}H NMR (300 MHz, DMSO-*d*^{6}): δ (ppm) = 12.18 (*br* s, 1H), 8.05 – 8.02 (m, 1H), 7.99 – 7.94 (m, 4H), 7.59 – 7.51 (m, 4H), 7.46 (t, *J* = 7.9 Hz, 2H), 7.29 (t, *J* = 7.4 Hz, 1H). ^{13}C NMR (75 MHz, DMSO-*d*^{6}): δ (ppm) = 198.2 (C), 142.9 (C), 139.9 (2C), 133.5 (C), 129.2 (2CH), 129.1 (CH), 128.7 (CH), 127.3 (CH), 126.9 (CH), 126.8 (CH), 125.8 (CH), 125.2 (CH), 124.3 (CH), 123.8 (2CH). HMRS (ESI$^+$, m/z) calculated for $(C_{17}H_{14}NS)^+$ [(M+H)$^+$]: 264.0841; found: 264.0838.

(_E_)-_N_-Phenylcinnamthioamide (3z)

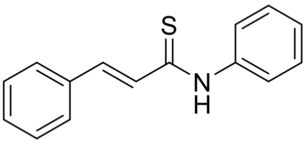

Orangish solid (40 mg, 42% yield, purified in *n*-Hexane/EtOAc 5:1). ^{1}H NMR (300 MHz, DMSO-*d*^{6}): δ (ppm) = 11.65 (*br* s, 1H), 7.90 (d, *J* = 8.1 Hz, 2H), 7.79 (d, *J* = 15.3 Hz, 1H), 7.64 (d, *J* = 7.3 Hz, 2H), 7.47 – 7.21 (m, 7H). ^{13}C NMR (75 MHz, DMSO-*d*^{6}): δ (ppm) = 192.5 (C), 142.2 (CH), 140.0 (C), 135.4 (C), 130.4 (CH), 129.6 (2CH), 129.5 (CH), 129.0 (2CH), 128.3 (2CH), 126.5 (CH), 123.8 (2CH). HMRS (ESI$^+$, m/z) calculated for $(C_{15}H_{14}NS)^+$ [(M+H)$^+$]: 240.0841; found: 240.0847. The compound has been previously described in the literature.[26]

2-Methyl-_N_-phenylpropanthioamide (3aa)

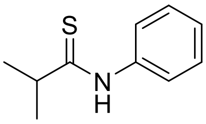

Pale yellow oil (30 mg, 42% yield, purified in *n*-Hexane/EtOAc 5:1). ^{1}H and ^{13}C NMR show mixture of rotamers (6:1), signal description refers to major *Z*- isomer. ^{1}H NMR (300 MHz, $CDCl_3$): δ (ppm) = 8.71 (*br* s, 1H), 7.66 (d, *J* = 8.0 Hz, 2H), 7.39 (t, *J* = 7.8 Hz, 2H), 7.26 (t, *J* = 7.7 Hz, 1H), 2.96 (hept, *J* = 6.9 Hz, 1H), 1.35 (d, *J* = 6.8 Hz, 6H). ^{13}C NMR (75 MHz, $CDCl_3$): δ (ppm) = 211.2 (C), 138.5 (C),

128.9 (2CH), 126.9 (CH), 124.1 (2CH), 46.0 (CH), 22.8 (2CH_3). The spectroscopic data were matched with the one reported in the literature.[27]

***N*-(4-Fluorophenyl)benzothioamide (3ab)**

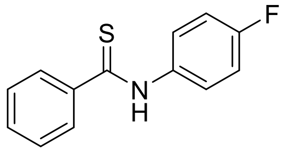

Yellow solid (56 mg, 61% yield, purified in *n*-Hexane/EtOAc 5:1). ^{1}H NMR (300 MHz, $CDCl_3$): δ (ppm) = 9.01 (*br* s, 1H), 7.87 – 7.79 (m, 2H), 7.72 – 7.64 (m, 2H), 7.55 – 7.39 (m, 3H), 7.17 – 7.07 (m, 2H). ^{19}F NMR (282 MHz, $CDCl_3$): δ (ppm) = -113.6. ^{13}C NMR (75 MHz, $CDCl_3$): δ (ppm) = 199.0 (C), 160.9 (d, *J* = 247.5 Hz, C), 142.7 (C), 134.9 (d, *J* = 2.5 Hz, C), 131.5 (CH), 128.7 (2CH), 126.7 (2CH), 126.1 (d, *J* = 8.2 Hz, 2CH), 116.0 (d, *J* = 22.9 Hz, 2CH). The spectroscopic data were matched with the one reported in the literature.[21]

***N*-(4-Bromophenyl)benzothioamide (3ac)**

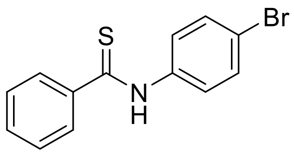

Yellow solid (82 mg, 70% yield, purified in *n*-Hexane/EtOAc 5:1). ^{1}H NMR (300 MHz, $CDCl_3$): δ (ppm) = 8.97 (*br* s, 1H), 7.83 (d, *J* = 7.9 Hz, 2H), 7.67 (d, *J* = 7.8 Hz, 2H) 7.56 – 7.41 (m, 5H). ^{13}C NMR (75 MHz, $CDCl_3$): δ (ppm) = 198.6 (C), 142.9 (C), 138.0 (C), 132.2 (2CH), 131.5 (CH), 128.7 (2CH), 126.7 (2CH), 125.3 (2CH), 120.0 (C). The spectroscopic data were matched with the one reported in the literature.[21]

***N*-Methylbenzothioamide (3ad)**

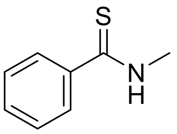

Pale yellow solid (34 mg, 57% yield using 1.2 equiv. CS_2 for 16 h; 54 mg, 90% yield using 3 equiv. CS_2 for 40 h, purified in *n*-Hexane/EtOAc 5:1). ^{1}H NMR (300 MHz, $CDCl_3$): δ (ppm) = 7.76 – 7.59 (m + *br* s, 3H), 7.49 – 7.43 (m, 1H), 7.41 – 7.35 (m, 2H), 3.35 (d, *J* = 4.9 Hz, 3H). ^{13}C NMR (75 MHz, $CDCl_3$): δ (ppm) = 200.3 (C), 141.8 (C), 131.0 (CH), 128.5 (2CH), 126.6 (2CH), 33.7 (CH_3). The spectroscopic data were matched with the one reported in the literature.[28]

_N_-Benzylbenzothioamide (3ae)

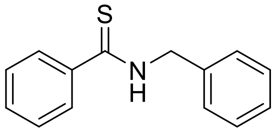

Pale yellow solid (31 mg, 34% yield using 1.2 equiv. CS_2 for 16 h; 55 mg, 60% yield using 3 equiv. CS_2 for 40 h, purified in *n*-Hexane/EtOAc 5:1). ^{1}H NMR (300 MHz, $CDCl_3$): δ (ppm) = 7.79 – 7.66 (m + *br* s, 3H), 7.49 – 7.32 (m, 8H), 5.00 (d, *J* = 5.1 Hz, 2H). ^{13}C NMR (75 MHz, $CDCl_3$): δ (ppm) = ^{13}C NMR (75 MHz, $CDCl_3$) δ 199.2 (C), 141.7 (C), 136.2 (C), 131.2 (CH), 129.1 (2CH), 128.6 (2CH), 128.4 (2CH), 128.3 (CH), 126.7 (2CH), 51.1 (CH_2). The spectroscopic data were matched with the one reported in the literature.[25]

3,4-Dihydroisoquinoline-1-thione (3af)

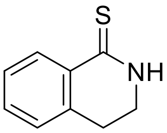

Yellow solid (62 mg, 95% yield, purified in *n*-Hexane/EtOAc 4:1). ^{1}H NMR (300 MHz, $CDCl_3$): δ (ppm) = 8.92 (*br* s, 1H), 8.52 (d, *J* = 7.8 Hz, 1H), 7.45 (td, *J* = 7.5, 1.6 Hz, 1H), 7.34 (td, *J* = 7.6, 1.5 Hz, 1H), 7.17 (d, *J* = 7.5 Hz, 1H), 3.55 (td, *J* = 6.9, 3.5 Hz, 2H), 3.01 (t, *J* = 6.9 Hz, 2H). ^{13}C NMR (75 MHz, $CDCl_3$): δ (ppm) = 194.1 (C), 133.9 (C), 132.7 (CH), 132.3 (C), 132.0 (CH), 127.3 (CH), 127.1 (CH), 41.8 (CH_2), 27.9 (CH_2). The spectroscopic data were matched with the one reported in the literature.[29]

Pyrrolidine-2-thione (3ag)

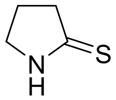

Pale yellow solid (33 mg, 81% yield, purified in *n*-Hexane/EtOAc 2:1). ^{1}H NMR (300 MHz, $CDCl_3$): δ (ppm) = 8.90 (*br* s, 1H), 3.64 (t, *J* = 7.3 Hz, 2H), 2.88 (t, *J* = 8.0 Hz, 2H), 2.23 – 2.13 (m, 2H). ^{13}C NMR (75 MHz, $CDCl_3$): δ (ppm) = 205.7 (C), 49.8 (CH_2), 43.4 (CH_2), 22.9 (CH_2). The spectroscopic data were matched with the one reported in the literature.[30]

N-Ethylthioacetamide (3ah)

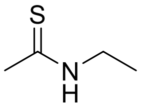

Yellow oil (24 mg, 59% yield, purified in *n*-Hexane/EtOAc 2:1). ^{1}H NMR (300 MHz, DMSO-d^6): δ (ppm) = 9.92 (*br* s, 1H), 3.45 (qd, *J* = 7.3, 5.0 Hz, 2H), 2.34 (s, 3H), 1.10 (t, *J* = 7.3 Hz, 3H). ^{13}C NMR (75 MHz, DMSO-d^6): δ (ppm) = 198.9 (C), 40.7 (CH_2), 33.3 (CH_3), 13.2 (CH_3). The spectroscopic data were matched with the one reported in the literature.[31]

V.2- Gram-scale synthesis of thioamide 3a.

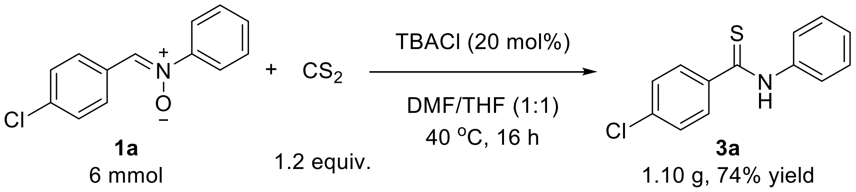

Scheme SI_8: Scale-up procedure for the synthesis of **3a**.

To a solution of tetrabutylammonium chloride (334 mg, 1.2 mmol, 0.2 equiv.) and *N*-(4-chlorobenzylidene)aniline oxide (**1a**, 1.39 g, 6 mmol, 1 equiv.) in 30 mL of a mixture of dry DMF and dry THF (1:1) in a 45 mL high-pressure close-capped tube, CS_2 (433 µL, 7.2 mmol, 1.2 equiv.) was added under argon. The resulting mixture was stirred for 16 hours at 40 °C (oil bath). Then, the crude was extracted with EtOAc (200 mL) and distilled water (200 mL), the aqueous phase was washed with EtOAc (200 mL), the organic phases combined, dried with $MgSO_4$, filtered and solvent and volatiles were removed under vacuum. Finally, the crude was purified by flash chromatography on silica gel (*n*-Hexane/EtOAc 5:1) to afford 1.10 g of 4-chloro-*N*-phenylbenzothioamide (**3a**, 74% yield).

VI.- Total synthesis of UC-781.

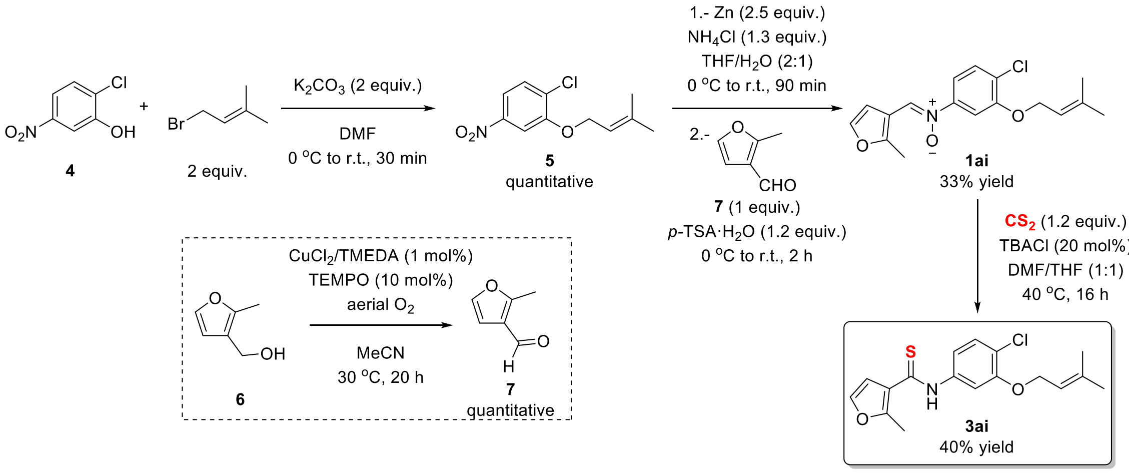

Scheme SI_9: Total synthesis of **UC-781 (3ai)**.

VI.1- Aerial oxidation of (2-methyl-3-furyl)methanol (6).[32,33]

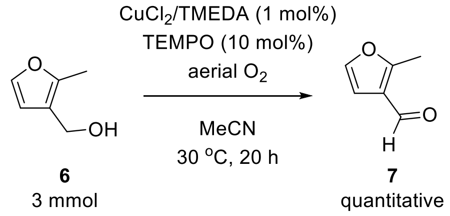

Scheme SI_10: Aerial oxidation of **6**.

To a solution of $CuCl_2 \cdot 2H_2O$ (5 mg, 0.03 mmol, 0.01 equiv.) and (2,2,6,6-tetramethylpiperidin-1-yl)oxyl (TEMPO, 47 mg, 0.3 mmol, 0.1 equiv.) in 2 mL of MeCN, *N,N,N',N'*-tetramethylethylenediamine (5 µL, 0.03 mmol, 0.01 equiv.) was added and the resulting mixture was stirred for a minute. Then, (2-methyl-3-furyl)methanol (**6**, 306 µL, 3 mmol, 1 equiv.) was added and the reaction mixture vigorously stirred open-to-air at 30 °C (oil bath) for 20 hours. Upon completion, the mixture was extracted with HCl 2M (20 mL) and Et_2O (20 mL), the aqueous phase washed with Et_2O (2 × 20 mL) and the organic phases were combined. To this solution, Dowex® Marathon™ MSC hydrogen form was added (570 mg) and the mixture vigorously stirred for 15 minutes, dried with $MgSO_4$ and filtered. Finally, solvent was carefully removed under vacuum (900 to 300 mbar, 40°C) leading to 2-methylfuran-3-carbaldehyde (**7**, 329 mg, >99% yield) as a reddish oil which was used in the next step without further purification.

2-Methylfuran-3-carbaldehyde (7): ^1H NMR (300 MHz, $CDCl_3$): δ (ppm) = 10.02 (s, 1H), 7.37 (d, *J* = 2.0 Hz, 1H), 6.76 (d, *J* = 2.0 Hz, 1H), 2.67 (s, 3H). ^{13}C NMR (75 MHz, $CDCl_3$): δ (ppm) = 185.0

(CHO), 162.0 (C), 142.2 (CH), 122.7 (C), 108.2 (CH), 12.8 (CH_3). The spectroscopic data were matched with the one reported in the literature.[34]

VI.2.-Synthesis of 1-chloro-2-((3-methylbut-2-en-1-yl)oxy)-4-nitrobenzene (5).

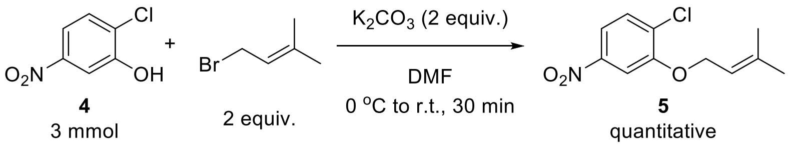

Scheme SI_11: Synthesis of **5**.

A solution of 2-chloro-5-nitrophenol (**4**, 521 mg, 3 mmol, 1 equiv.) in 30 mL of DMF was cooled down to 0 °C (ice bath) before K_2CO_3 (829 mg, 6 mmol, 2 equiv.) was added by portions and the resulting mixure stirred for 10 minutes. Then, 1-bromo-3-methylbut-2-ene (693 µL, 6 mmol, 2 equiv.) was added dropwise and the mixture was stirred and allowed to slowly reach room temperature (30 minutes, TLC monitored). Upon completion, the reaction mixture was extracted with distilled water (200 mL) and EtOAc (200 mL), the aqueous phase washed with EtOAc (200 mL), the organic liquors combined, washed with brine (200 mL), dried with $MgSO_4$ and filtered. Finally, solvent and volatiles were removed under vacuum and the crude was purified by flash chromatography on silica (*n*-Hexane/EtOAc 10:1) to afford 1-chloro-2-((3-methylbut-2-en-1-yl)oxy)-4-nitrobenzene (**5**, 723 mg, >99% yield) as a pale yellow solid.

1-chloro-2-((3-methylbut-2-en-1-yl)oxy)-4-nitrobenzene (5): ^{1}H NMR (300 MHz, $CDCl_3$): δ (ppm) = 7.79 – 7.75 (m, 2H), 7.51 – 7.48 (m, 1H), 5.52 – 5.44 (m, 1H), 4.70 (d, *J* = 6.7 Hz, 2H), 1.80 (s, 6H). ^{13}C NMR (75 MHz, $CDCl_3$): δ (ppm) = 154.7 (C), 147.2 (C), 139.9 (C), 130.4 (CH + C), 118.1 (CH), 116.0 (CH), 108.1 (CH), 66.6 (CH_2), 25.9 (CH_3), 18.4 (CH_3).

VI.3.- Synthesis of nitrone 1ai.

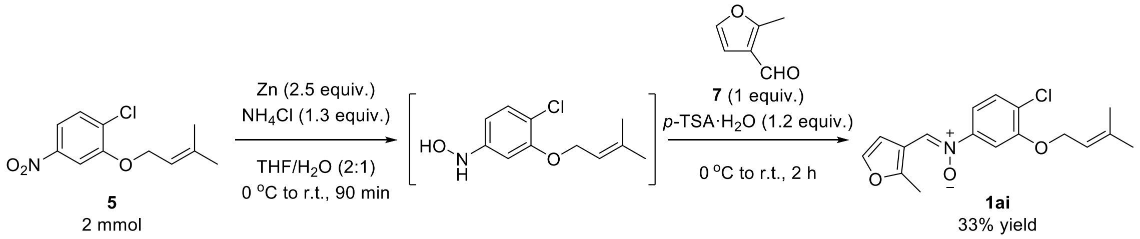

Scheme SI_12: Synthesis of nitrone **1ai**.

To a solution of 1-chloro-2-((3-methylbut-2-en-1-yl)oxy)-4-nitrobenzene (**5**, 483 mg, 2 mmol, 1 equiv.) in 16 mL of THF, distilled water (8 mL) was added dropwise under vigorous stirring. Then, NH_4Cl (139 mg, 2.6 mmol, 1.3 equiv.) was added and the reaction mixture was cooled down to 0

°C (ice bath) before Zn dust (327 mg, 5 mmol, 2.5 equiv.) was added by portions over 15 minutes. The heterogeneous mixture was allowed to reach room temperature and vigorously stirred for 90 minutes. After that time, the mixture was filtered through a pad of Celite®. The filtered liquor was cooled down to 0 °C (ice bath) and a solution of 2-methylfuran-3-carbaldehyde (**7**, 220 mg, 2 mmol, 1 equiv.) in 2 mL of THF added under stirring. Sequently, *p*-toluenesulfonic acid monohydrate (*p*-TSA·H_2O, 457 mg, 2.4 mmol, 1.2 equiv.) was added by portions over 15 minutes and the resulting mixture was allowed to reach room temperature, stirred for 2 hours and extracted with EtOAc (50 mL) and brine (50 mL). The aqueous phase was washed with EtOAc (50 mL), the organic liquors combined, dried with $MgSO_4$ and filtered. Finally, solvent and volatiles were removed under vacuum and the crude was purified by flash chromatography on silica (*n*-Hexane/EtOAc 3:1 to 2:1) to afford 4-chloro-3-((3-methylbut-2-en-1-yl)oxy)-*N*-(2-methylfuran-3-yl-methylidine)aniline oxide (**1ai**, 208 mg, 33% yield) as a yellow oil.

4-chloro-3-((3-methylbut-2-en-1-yl)oxy)-*N*-(2-methylfuran-3-yl-methylidine)aniline oxide (1ai): ^{1}H NMR (300 MHz, $CDCl_3$): δ (ppm) = 7.77 (s, 1H), 7.66 (d, *J* = 2.1 Hz, 1H), 7.57 (d, *J* = 2.4 Hz, 1H), 7.41 – 7.38 (m, 2H), 7.15 (dd, *J* = 8.5, 2.4 Hz, 1H), 5.56 – 5.49 (m, 1H), 4.66 (d, *J* = 6.7 Hz, 2H), 2.46 (s, 3H), 1.80 (s, 3H), 1.75 (s, 3H). ^{13}C NMR (75 MHz, $CDCl_3$): δ (ppm) = 155.9 (C), 155.0 (C), 147.7 (C), 141.1 (CH), 139.1 (C), 129.9 (CH), 127.3 (CH), 124.3 (C), 118.6 (CH), 114.3 (C), 112.8 (CH), 110.5 (CH), 107.7 (CH), 66.4 (CH_2), 25.8 (CH_3), 18.4 (CH_3), 12.7 (CH_3). HMRS (ESI$^+$, m/z) calculated for $(C_{17}H_{19}ClNO_3)^+$ [(M+H)$^+$]: 320.1048; found: 320.1050.

VI.4.- Synthesis of 3ai (UC-781).

3ai (UC-781) was obtained by applying the general procedure for the synthesis of thioamides from nitrones (see Section V.1) to 4-chloro-3-((3-methylbut-2-en-1-yl)oxy)-*N*-(2-methylfuran-3-yl-methylidine)aniline oxide (**1ai**, 128 mg, 0.4 mmol).

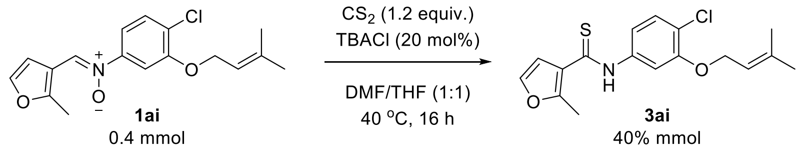

Scheme SI_13: Synthesis of **3ai** (UC-781).

3ai (UC-781)**:** yellow solid (54 mg, 40% yield, purified in *n*-Hexane/EtOAc 6:1). Melting point: 130 – 132 °C. R_f (*n*-Hexane/EtOAc 5:1) = 0.46. ^{1}H NMR (300 MHz, DMSO-*d*^{6}): δ (ppm) = 11.24 (*br* s, 1H), 7.66 (*br* s, 1H), 7.57 (d, *J* = 2.1 Hz, 1H), 7.42 (d, *J* = 8.5 Hz, 1H), 7.32 – 7. 27 (m, 1H), 6.83 (*br* s, 1H), 5.44 (t, *J* = 6.8 Hz, 1H), 4.57 (d, *J* = 6.6 Hz, 2H), 2.54 (s, 3H), 1.74 (s, 3H), 1.70 (s, 3H). ^{13}C NMR (75 MHz, DMSO-*d*^{6}): δ (ppm) = 190.5 (C), 154.7 (C), 153.8 (C), 140.9 (CH), 139.8 (C), 138.5

(C), 129.9 (CH), 125.0 (C), 119.6 (CH), 118.9 (C), 117.7 (CH), 110.9 (CH), 110.7 (CH), 65.9 (CH_2), 26.0 (CH_3), 18.6 (CH_3), 14.8 (CH_3). HMRS (ESI^+, m/z) calculated for $(C_{17}H_{19}ClNO_2S)^+$ $[(M+H)^+]$: 336.0820; found: 336.0821.

VII.- Use of Lawesson's reagent.

A typical procedure for the synthesis of thioamides from amides using the Lawesson's reagent,[35] was performed with diamide *N*-phenyl-4-(pyrrolidine-1-carbonyl)benzamide to study the feasibility of obtaining thioamide **3q** by these methodology.

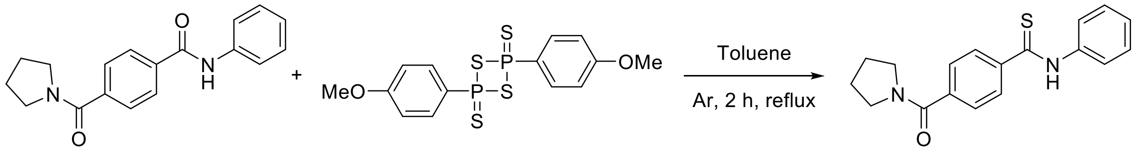

0.2 mmol 0.12 mmol **3q, n.d.**

Scheme SI_14: Use of Lawesson's reagent with *N*-phenyl-4-(pyrrolidine-1-carbonyl)benzamide.

Product **3q** was not detected by TLC (CH_2Cl_2/MeOH 97:3), comparing the crude reaction mixture with a sample of pure product **3q**.

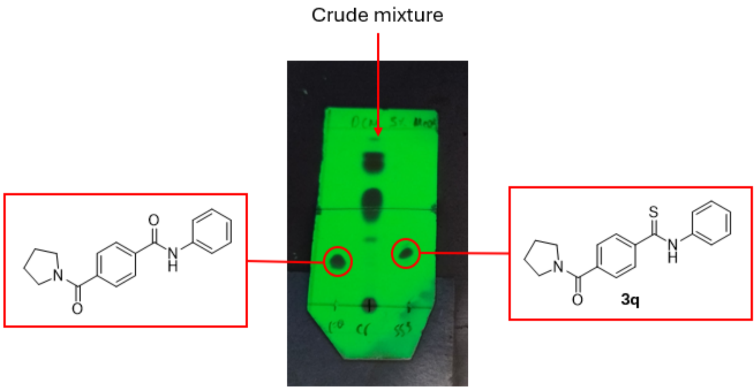

Figure SI_3: TLC (VIS-UV) of the crude obtained using Lawesson's reagent.

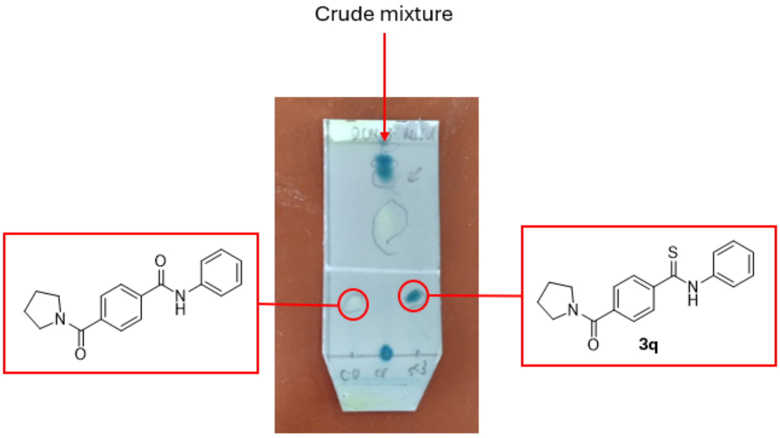

Figure SI_4: TLC (vanillin stain) of the crude obtained using Lawesson's reagent.

An analogous experiment was performed with nitrone **1a**. Again, thioamide **3a** was not detected by TLC (*n*-Hexane/EtOAc 4:1).

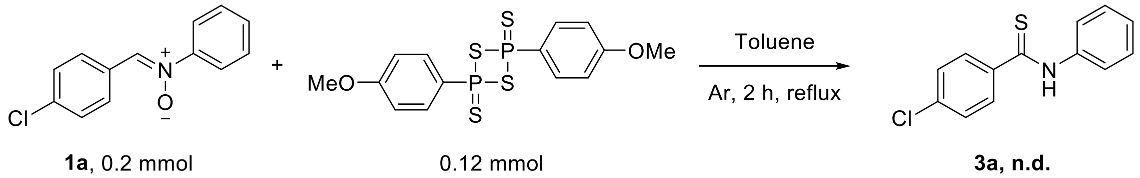

Scheme SI_15: Use of Lawesson's reagent with nitrone **1a**.

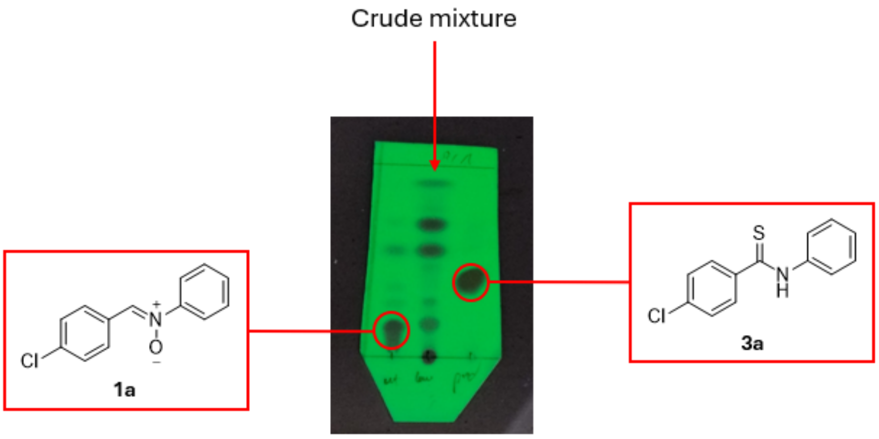

Figure SI_5: TLC (VIS-UV) of the crude obtained using Lawesson's reagent.

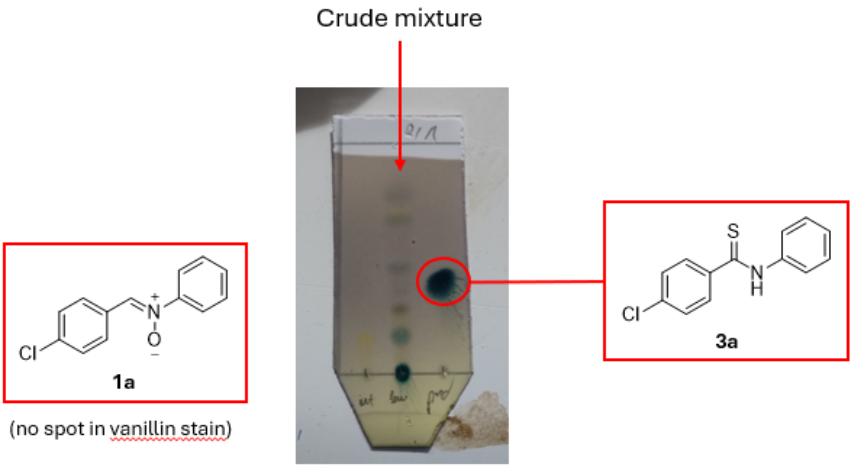

Figure SI_6: TLC (vanillin stain) of the crude obtained when Lawesson's reagent.

VIII.- X-Ray Data.

Crystals of **3a** and **3ai** (see Figures SI_7 and SI_8) were analysed by X-ray diffraction. A selection of crystal, measurement and refinement data is given in Table SI_11. Diffraction data were collected on an Oxford Diffraction Xcalibur Onyx Nova Geminy single crystal diffractometer. Empirical absorption corrections were applied using the SCALE3 ABSPACK algorithm as implemented in Chrysalis RED.[36] The structures were solved with SIR-2019.[37] Isotropic and full matrix anisotropic least square refinements were carried out using SHELXL-2018/3.[38] All non-H atoms were refined anisotropically. All H atoms were set in calculated positions and were refined riding on their parent atoms except for those of the NH groups of **3a** and **3ai** and the allylic CH of **3ai**, which were located in the corresponding Fourier map and refined freely. The WINGX program system (version 2021.1)[39] was used throughout the structure determination. The molecular plots were made with ORTEP.[39] The X-ray crystallographic coordinates for the structures reported in this study have been deposited at the Cambridge Crystallographic Data Centre (CCDC) under deposition numbers 2431009 and 2431010 for **3a** and **3ai**, respectively. These data can be obtained free of charge via http://www.ccdc.cam.ac.uk/data_request/cif.

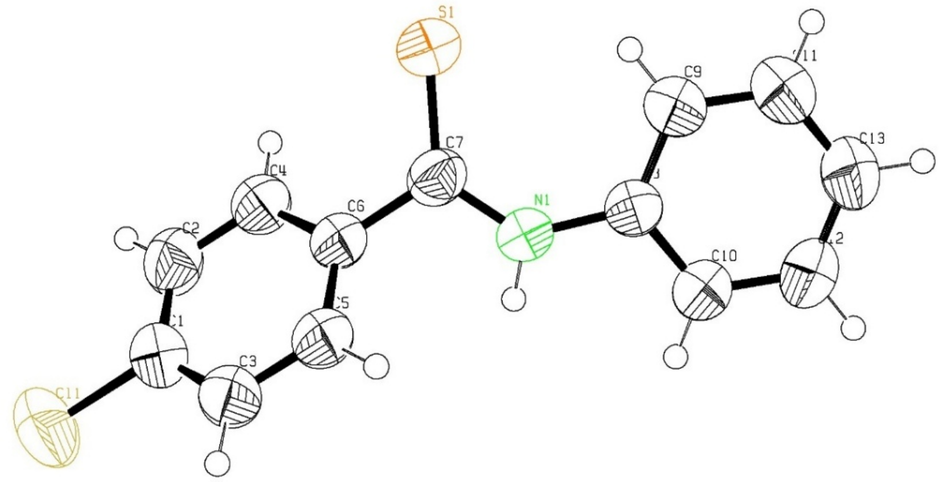

Figure SI_7: SCXRD molecular structure of **3a** (50% displacement ellipsoids).

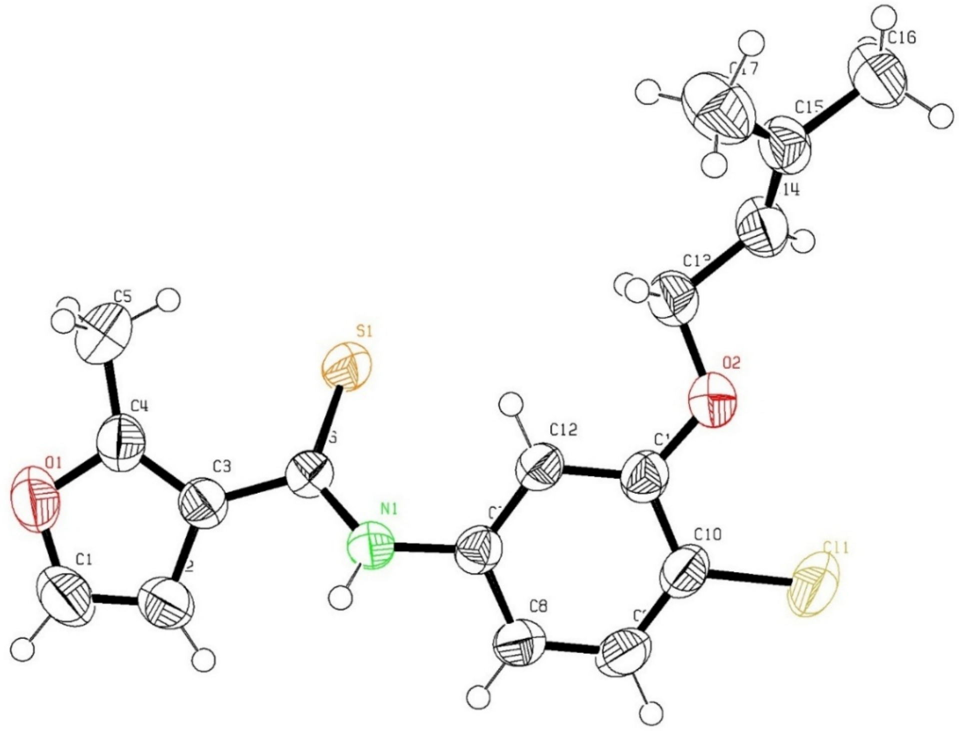

Figure SI_8: SCXRD molecular structure of **3ai** (50% displacement ellipsoids).

Table SI_11. Crystal, measurement and refinement data for the compounds studied by X-ray diffraction.

	**3a**	**3ai**
formula	$C_{13}H_{10}ClNS$	$C_{17}H_{18}ClNO_2S$
fw	247.73	335.83
cryst syst	monoclinic	orthorhombic
space group	Cc	Pbca
a, Å	6.1074(10)	17.6452(3)
b, Å	26.5202(19)	8.1606(2)
c, Å	10.3342(16)	23.6260(5)
α, deg	90	90
β, deg	134.10(3)	90
γ, deg	90	90
V, Å^3	1202.0(5)	3402.04(12)
Z	4	8
F(000)	512	1408
D_{calcd}, g cm^{-3}	1.369	1.311
μ, mm^{-1} (CuKα)	4.179	3.182
cryst size, mm	0.32 x 0.26 x 0.14	0.34 x 0.23 x 0.06
T, K	298(2)	298(2)
ϑ range, deg	3.33 to 69.39	3.74 to 69.57
min./max. *h*, *k*, *l*	-6/7, -32/30, -11/12	-20/21, -9/7, -28/15
no. collected reflns	3566	9960
no. unique reflns	1507	3147
no. reflns with $I > 2\sigma(I)$	1441	2785
no. params/restraints	150/2	207/0
GOF (on F^2)	1.068	1.080
R_1 (on F, $I > 2\sigma(I)$)	0.041	0.047
wR_2 (on F^2, all data)	0.117	0.153
min./max. $\Delta\rho$, e Å^{-3}	-0.143/0.306	-0.293/0.329
CCDC dep. no.	2431009	2431010

IX.- References.

[1] D. A. Bilodeau, K. D. Margison, N. Ahmed, M. Strmiskova, A. R. Sherratt, J. P. Pezacki, *Chem. Commun.* **2020**, *56*, 1988.

[2] D. P. Canterbury, I. R. Herrick, J. Um, K. N. Houk, A. J. Frontier, *Tetrahedron* **2009**, *65*, 3165.

[3] S. Cicchi, M. Marradi, A. Goti, A. Brandi, *Tetrahedron Lett.* **2001**, *42*, 6503.

[4] Á. S. Granato, G. W. Amarante, J. Adrio, *J. Org. Chem.* **2021**, *86*, 13817.

[5] V. G. Lisnyak, T. Lynch-Colameta, S. A. Snyder, *Angew. Chem. Int. Ed.* **2018**, *57*, 15162.

[6] H. E. Gottlieb, V. Kotlyar, A. Nudelman, *J. Org. Chem.* **1997**, *62*, 7512.

[7] X.-G. Zhang, Z.-Y. Zhou, J.-X. Li, J.-J. Chen, Q.-L. Zhou, *J. Am. Chem. Soc.* **2024**, *146*, 27274.

[8] M. Choi, M. Viji, D. Kim, Y. H. Lee, J. Sim, Y.-S. Kwak, K. Lee, H. Lee, J.-K. Jung, *Tetrahedron,* **2018***, 74,* 4182.

[9] J. Zhang, J.-Y. Su, H. Zheng, H. Li, W.-P. Deng, *Angew. Chem. Int. Ed.* **2024**, *63*, e202318476.

[10] P. Jiao, D. Nakashima, H. Yamamoto, *Angew. Chem. Int. Ed.* **2008**, *47*, 2411.

[11] Z. Tian, J. Xu, B. Liu, Q. Tan, B. Xu, *Org. Lett.* **2018**, *20*, 2603.

[12] G. K. S. Prakash, Z. Zhang, F. Wang, M. Rahm, C. Ni, M. Iuliucci, R. Haiges, G. A. Olah, *Chem. Eur. J.* **2014**, *20*, 831.

[13] H. G. Aurich, J. Eidel, M. Schmidt, *Chem. Ber.* **1986**, *119*, 18.

[14] N. Arumugam, P. Manisankar, S. Sivasubramanian, D. A. Wilson, *Org. Magn. Reson.* **1984**, *22*, 592.

[15] P. Das, A. T. Hamme II, *Tetrahedron Lett.* **2017***, 58,* 1086.

[16] C. M. Dicken, P. DeShong, *J. Org. Chem*. **1982**, *47*, 2047.

[17] Y. Imada, H. Iida, S. Ono, S.-I. Murahashi, *J. Am. Chem. Soc.* **2003**, *125*, 2868.

[18] I. Delso, A. Melicchio, A. Isasi, T. Tejero, P. Merino, *Eur. J. Org. Chem.* **2013***,* 5721.

[19] H. Xu, H. Deng, Z. Li, H. Xiang, X. Zhou, *Eur. J. Org. Chem.* **2013**, 7054.

[20] K. Waisser, J. Kuneš, L. Kubicová, M. Buděšínský, O. Exner, *Magn. Reson. Chem.* **1997**, *35*, 543.

[21] M. Qiao, J. Zhang, L. Chen, F. Zhou, Y. Zhang, L. Zhou, Y. Wu, *Org. Biomol. Chem.* **2019,** *17*, 3790.

[22] W.-P. Hu, Y.-K. Chen, C.-C. Liao, H.-S. Yu, Y.-M. Tsai, S.-M. Huang, F.-Y. Tsai, H.-C. Shen, L.-S. Chang, J.-J. Wang, *Bioorg. Med. Chem.* **2010**, *18*, 6197.

[23] K. Kumar, D. Konar, S. Goyal, M. Gangar, M. Chouhan, R. K. Rawal, V. A. Nair, *ChemistrySelect* **2016**, *1*, 3228.

[24] T. Guntreddi, R. Vanjari, K. N. Singh, *Org. Lett.* **2014**, *16*, 3624.

[25] V. Pace, L. Castoldi, S. Monticelli, S. Safranek, A. Roller, T. Langer, W. Holzer, *Chem. Eur. J.* **2015**, *21*, 18966.

[26] T. Nishio, H. Sekiguchi, *Tetrahedron* **1999***, 55,* 5017.

[27] A. R. Katritzky, J.-L. Moutou, Z. Yang, *Synthesis* **1995**, 1497.

[28] K. Xu, Z. Li, F. Cheng, Z. Zuo, T. Wang, M. Wang, L. Liu, *Org. Lett.* **2018**, *20*, 2228.

[29] C.-L. Zhao, Q.-Y. Han, C.-P. Zhang, *Org. Lett.* **2018**, *20*, 6480.

[30] N. D. Koduri, H. Scott, B. Hileman, J. D. Cox, M. Coffin, L. Glicksberg, S. R. Hussaini, *Org. Lett.* **2012**, *14*, 440.

[31] U. Pathak, L. K. Pandey, S. Mathur, M. V. S. Suryanarayana, *Chem. Commun.* **2009**, 5409.

[32] M. López-Aguilar, M. Ramos-Martín, N. Ríos-Lombardía, L. Cicco, J. García-Álvarez, C. Concellón, V. del Amo, *ChemCatChem* **2024,** *16*, e202400437.

[33] E. D. Silva, O. A. L. Alves, R. T. Ribeiro, R. C. R. Chagas, J. A. F. P. Villar, J. L. Princival, *Appl. Catal. A: Gen.* **2021**, *623*, 118289.

[34] N. Charbonnet, E. Riguet, C. G. Bochet, *Synlett* **2011**, *15*, 2231.

[35] Y.-Y. Cheng, J. Xu, Z. Lin, Y. Li, L. Ackermann, *Chem. Eur. J.* **2024**, *30*, e202402333.

[36] *CrysAlisPro RED, version 1.171.38.46: Oxford Diffraction Ltd, Oxford, U.K., 2015.*

[37] M. C. Burla, R. Caliandro, B. Carrozzini, G. L. Cascarano, C. Cuocci, C. Giacovazzo, M. Mallamo, A. Mazzone, G. Polidori, *J. Appl. Crystallogr.* **2015**, *48*, 306.

[38] G. M. Sheldrick, *Acta Cryst.* **2015**, *C71*, 3.

[39] L. J. Farrugia, *J. Appl. Cryst.* **2012**, *45*, 849.

X.- Copy of NMR and HRMS spectra.

N-(4-Chlorobenzylidene)aniline oxide (1a)

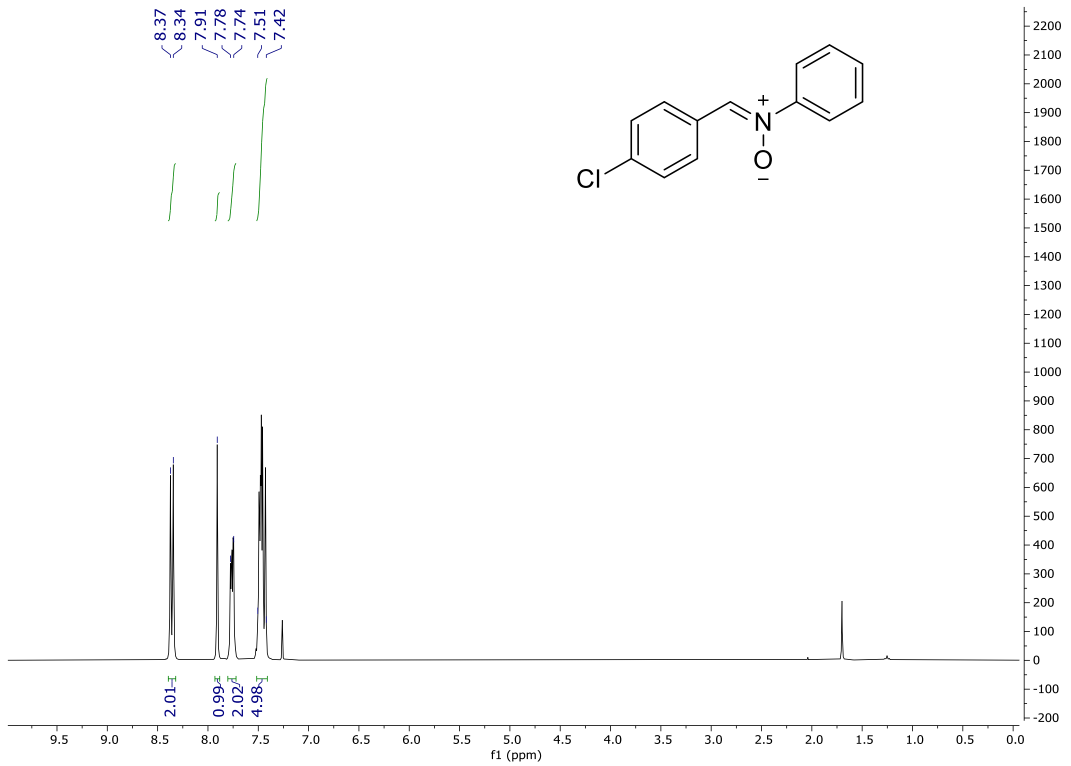

Figure SI_9: ^{1}H-NMR for **1a** in $CDCl_3$ (300 MHz).

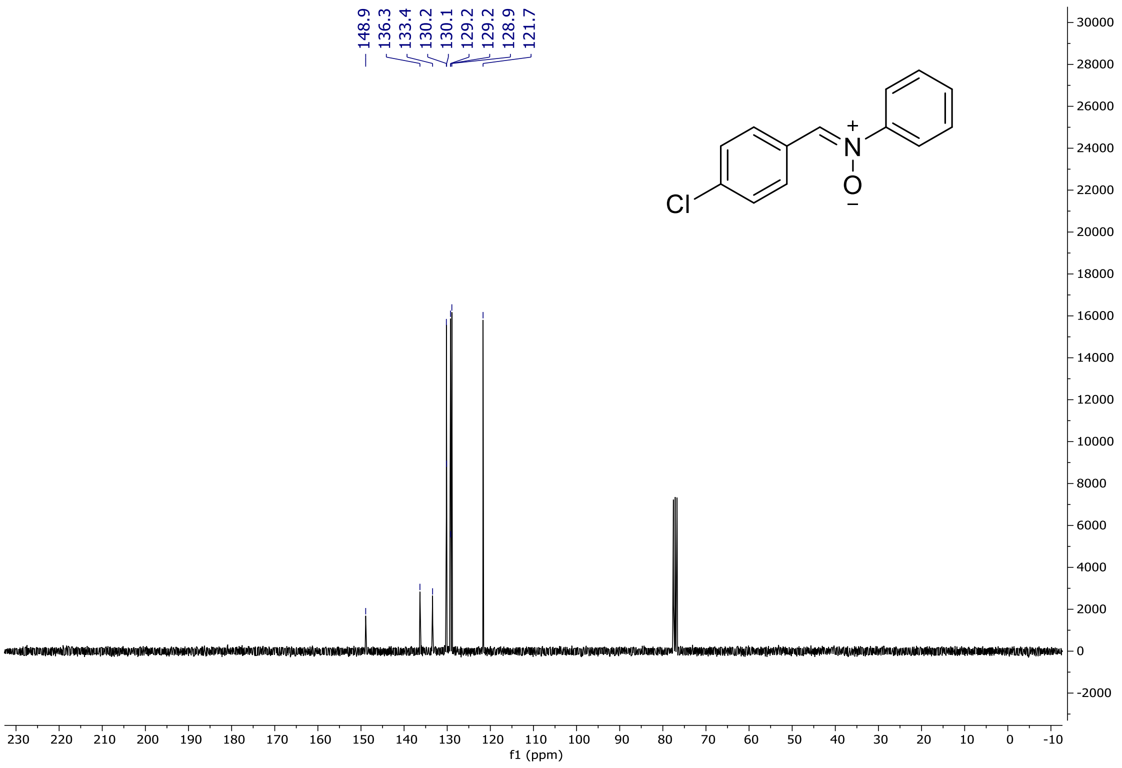

Figure SI_10: ^{13}C-NMR for **1a** in $CDCl_3$ (75 MHz).

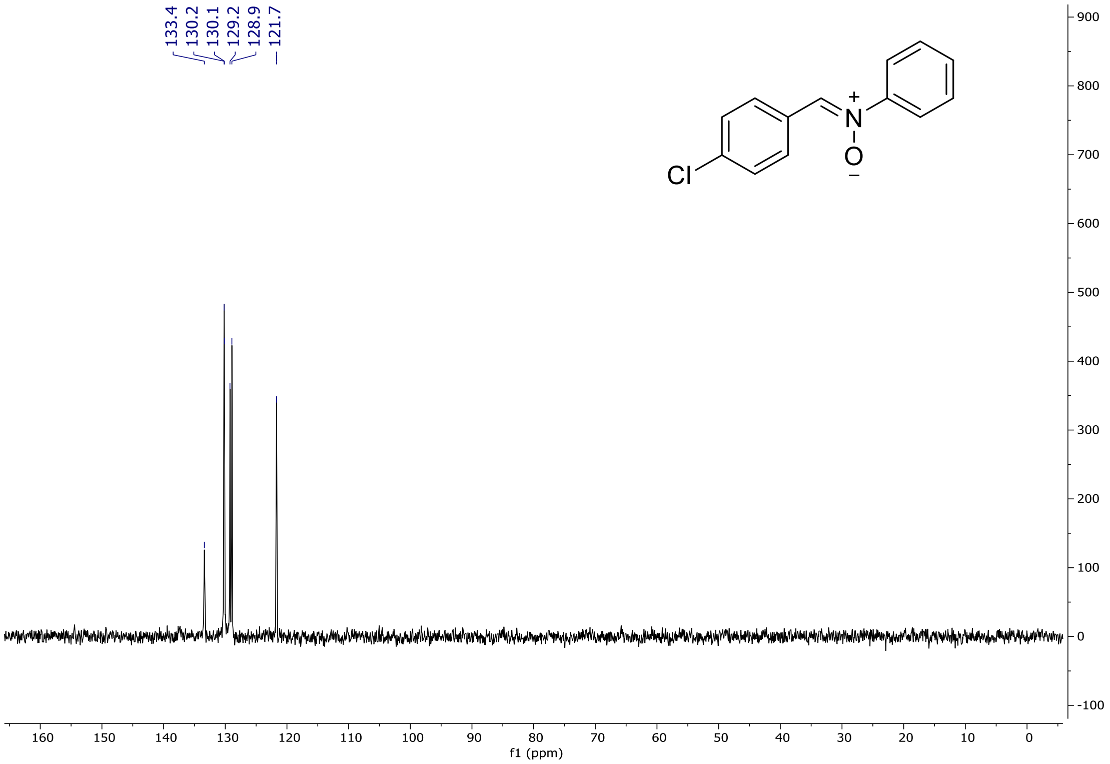

Figure SI_11: DEPT 135-NMR for **1a** in $CDCl_3$ (75 MHz).

N-Benzylideneaniline oxide (1b)

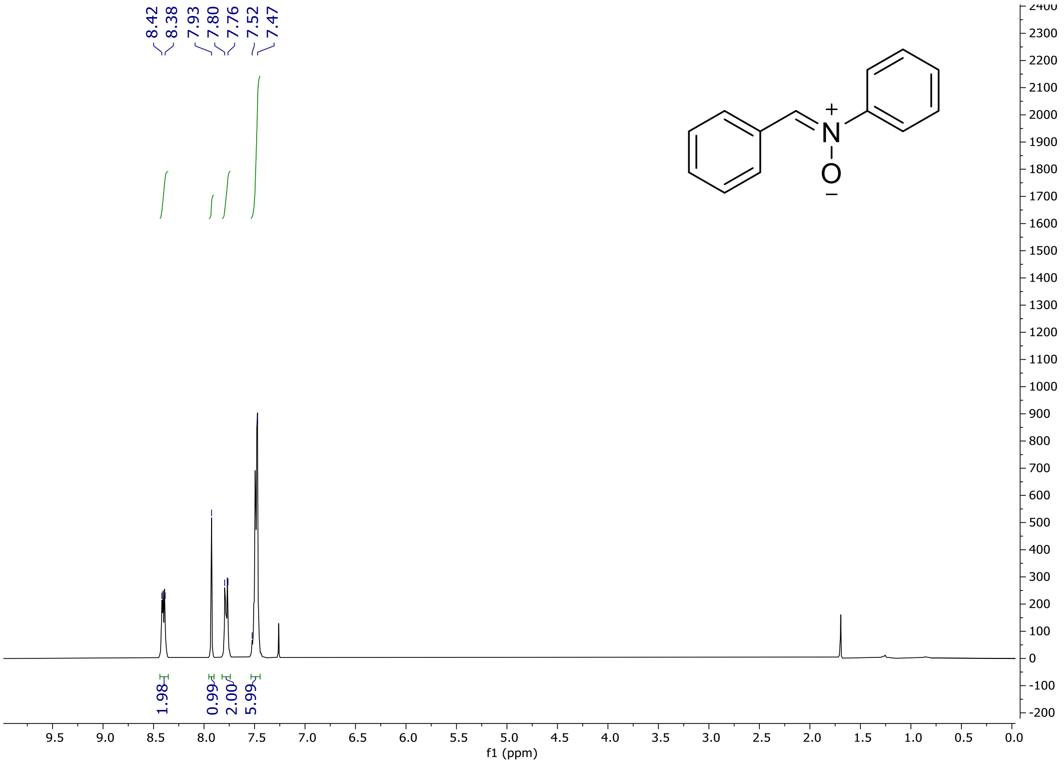

Figure SI_12: ^{1}H-NMR for **1b** in $CDCl_3$ (300 MHz).

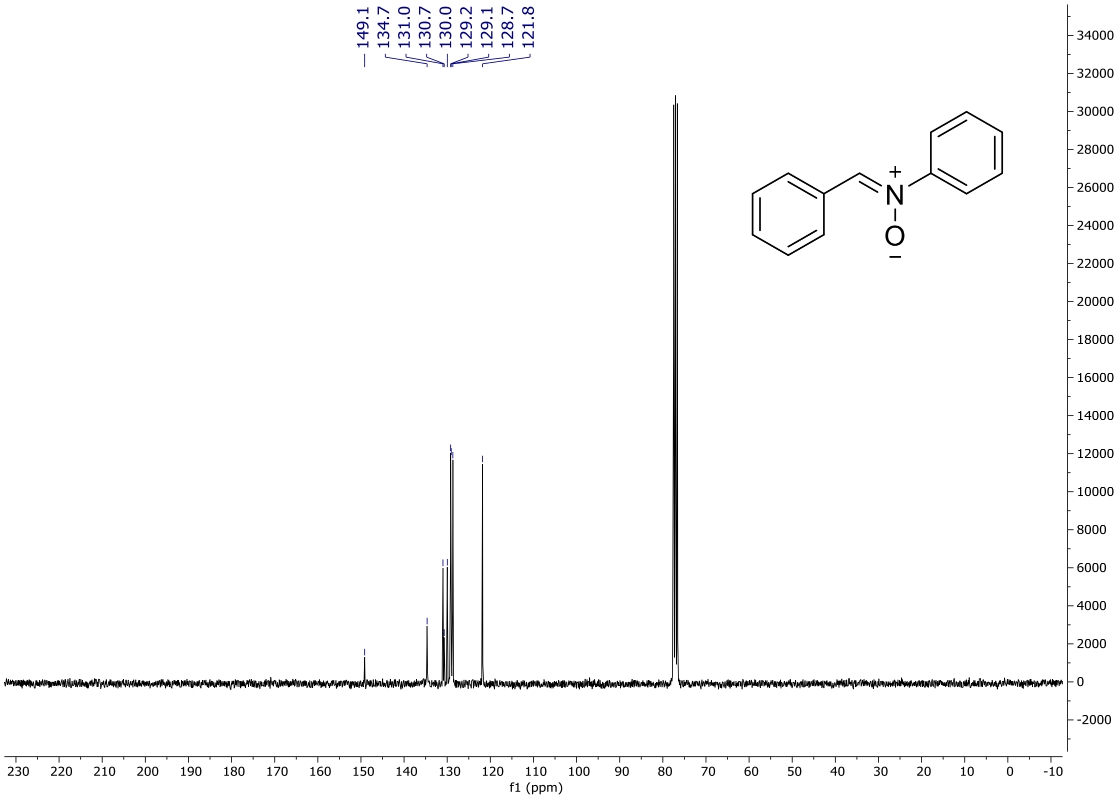

Figure SI_13: ^{13}C-NMR for **1b** in $CDCl_3$ (75 MHz).

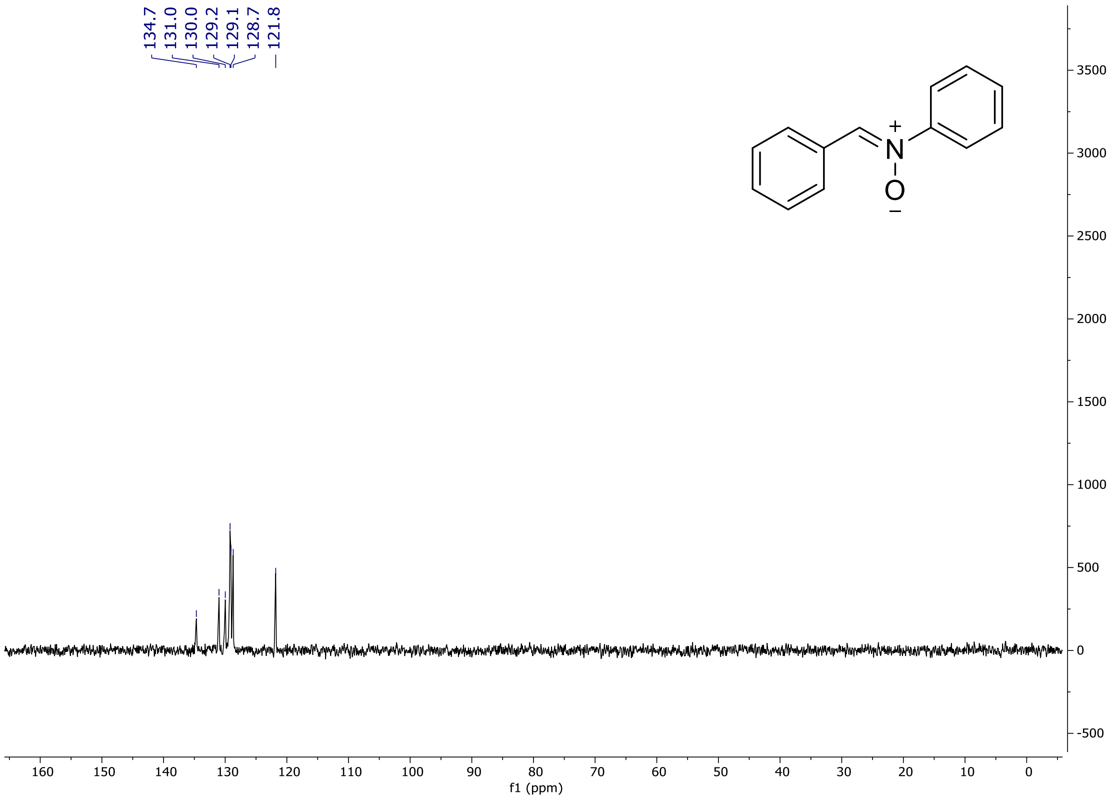

Figure SI_14: DEPT 135-NMR for **1b** in $CDCl_3$ (75 MHz).

_N_-(4-Fluorobenzylidene)aniline oxide (1c)

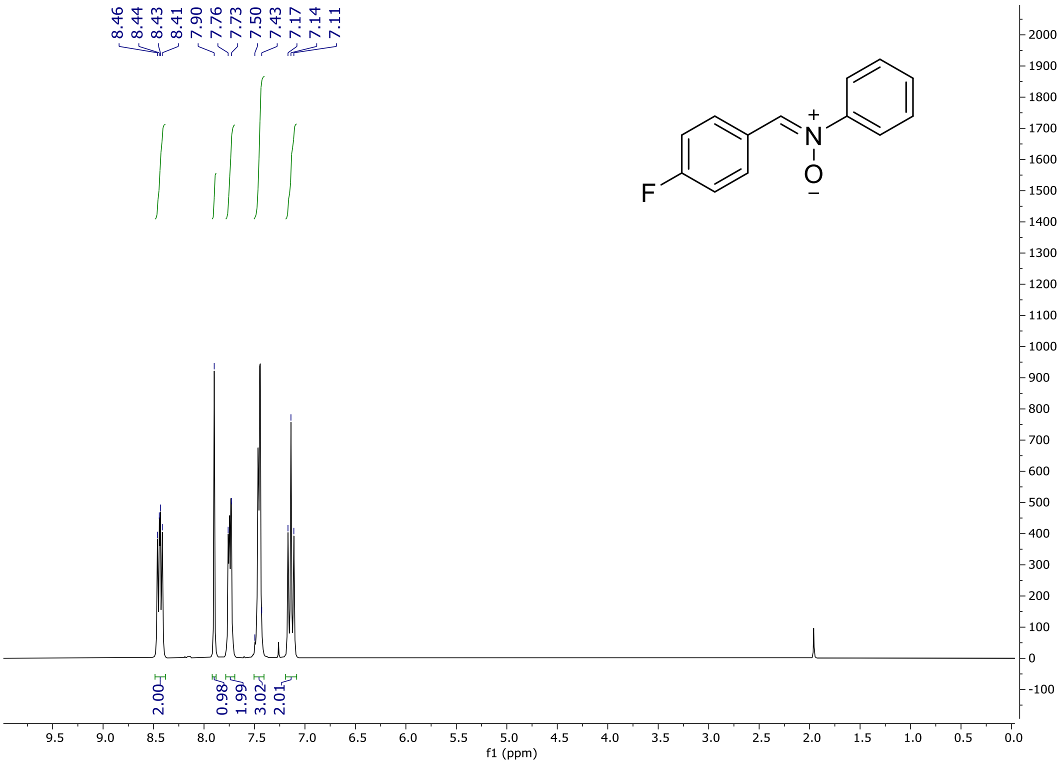

Figure SI_15: ^{1}H-NMR for **1c** in $CDCl_3$ (300 MHz).

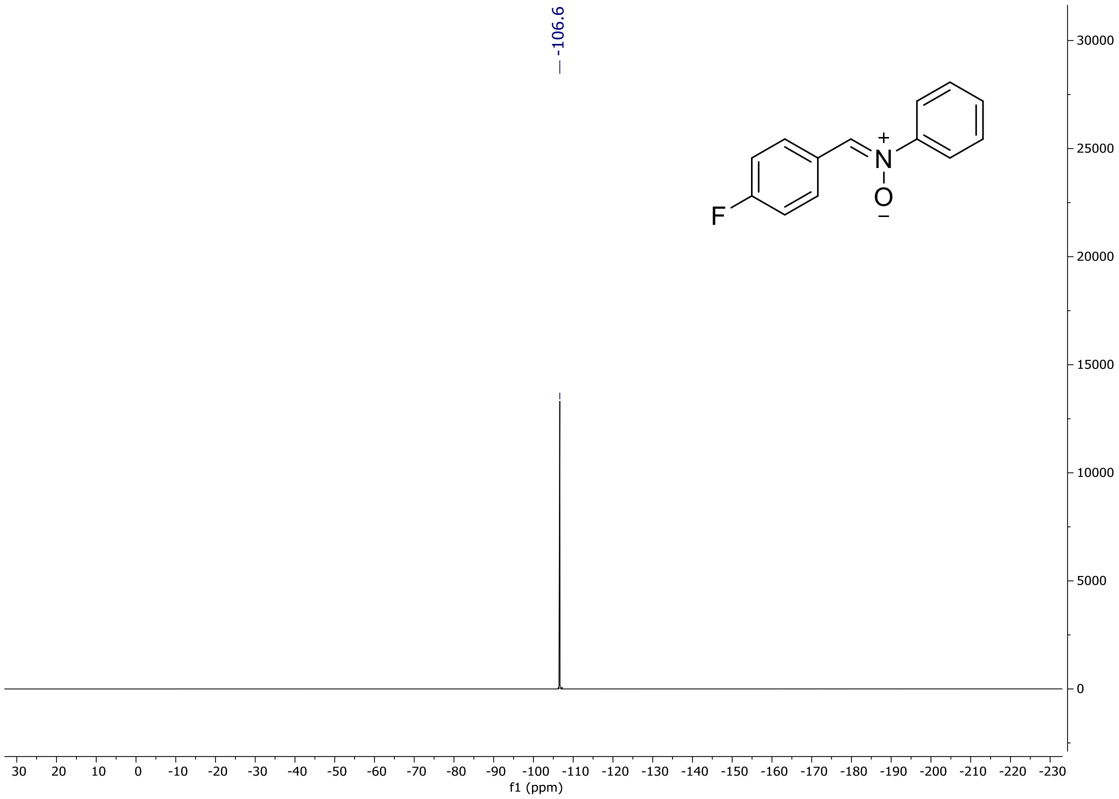

Figure SI_16: ^{19}F-NMR for **1c** in $CDCl_3$ (282 MHz).

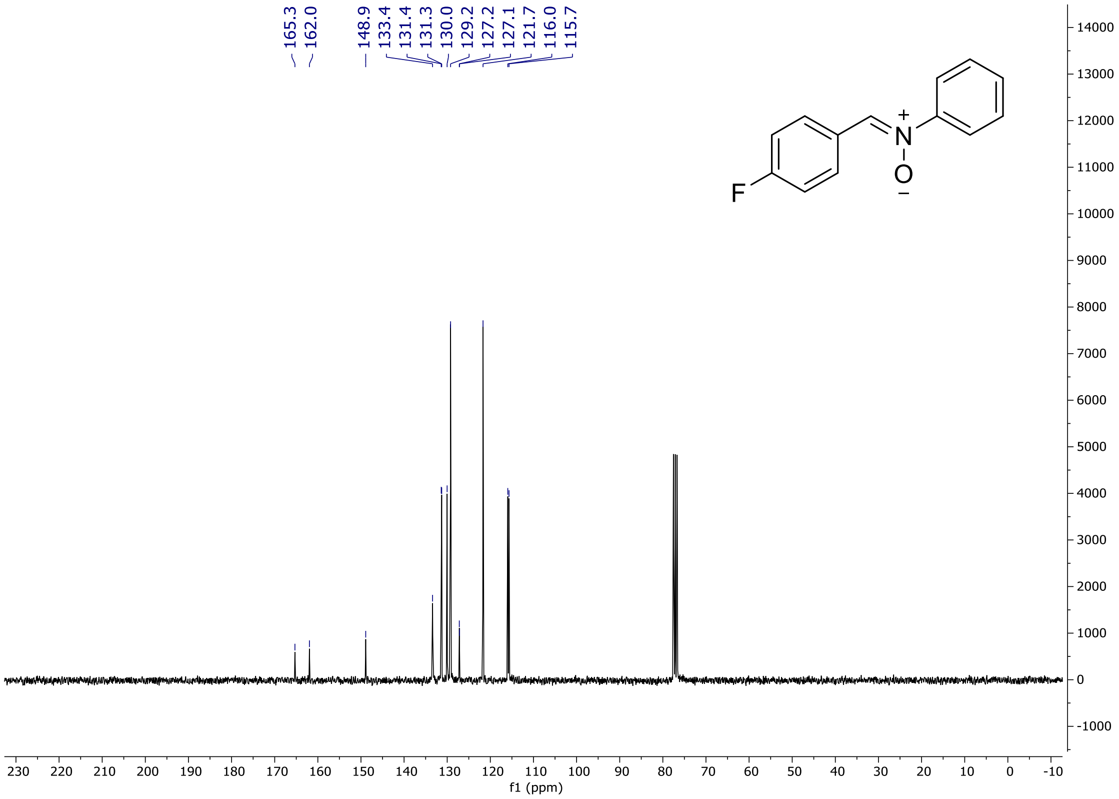

Figure SI_17: ^{13}C-NMR for **1c** in $CDCl_3$ (75 MHz).

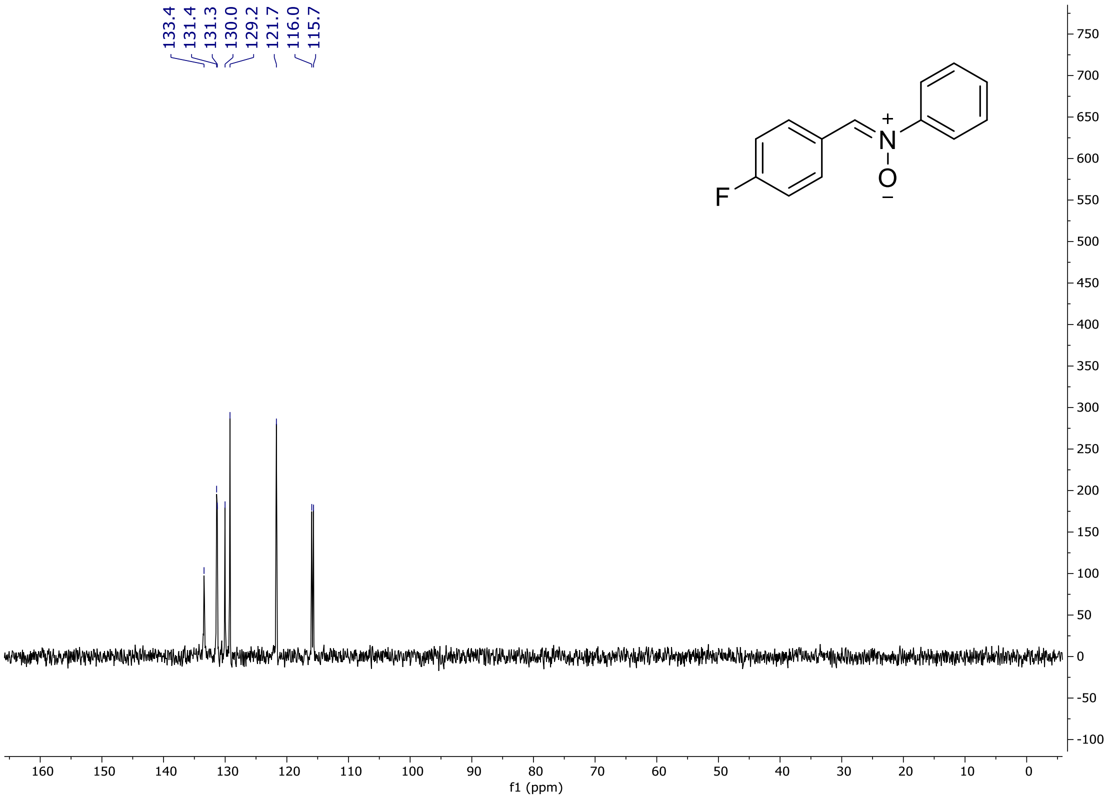

Figure SI_18: DEPT 135-NMR for **1c** in $CDCl_3$ (75 MHz).

N-(4-Bromobenzylidene)aniline oxide (1d)

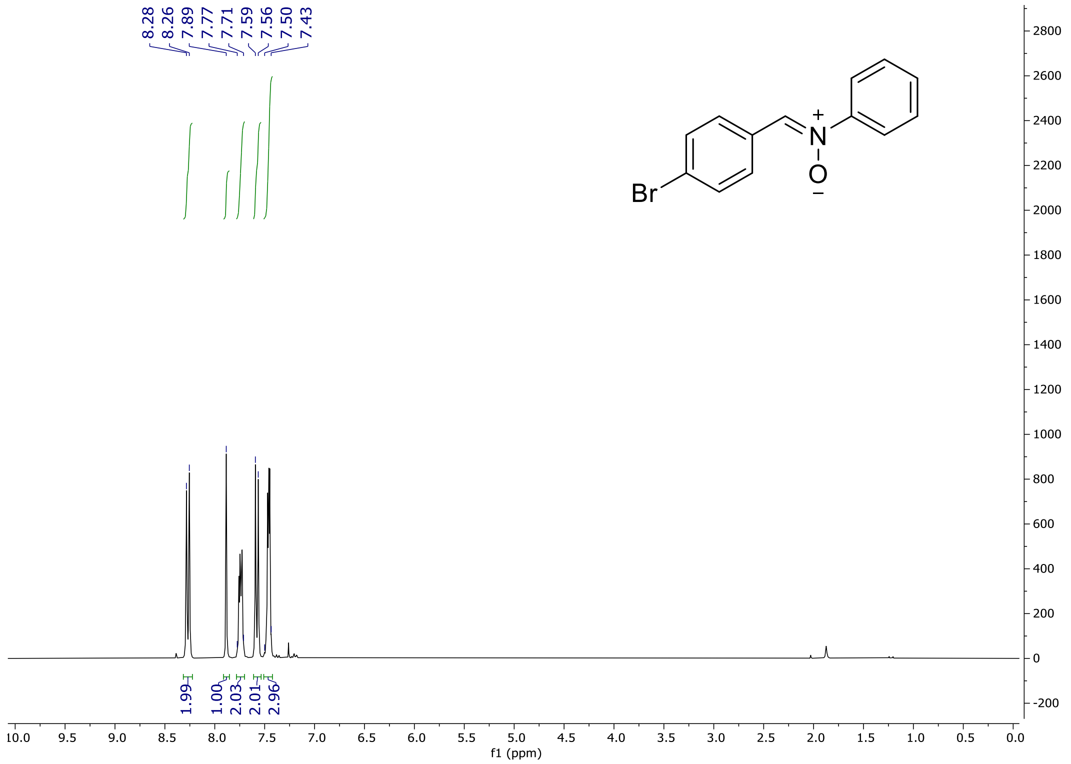

Figure SI_19: ^{1}H-NMR for **1d** in $CDCl_3$ (300 MHz).

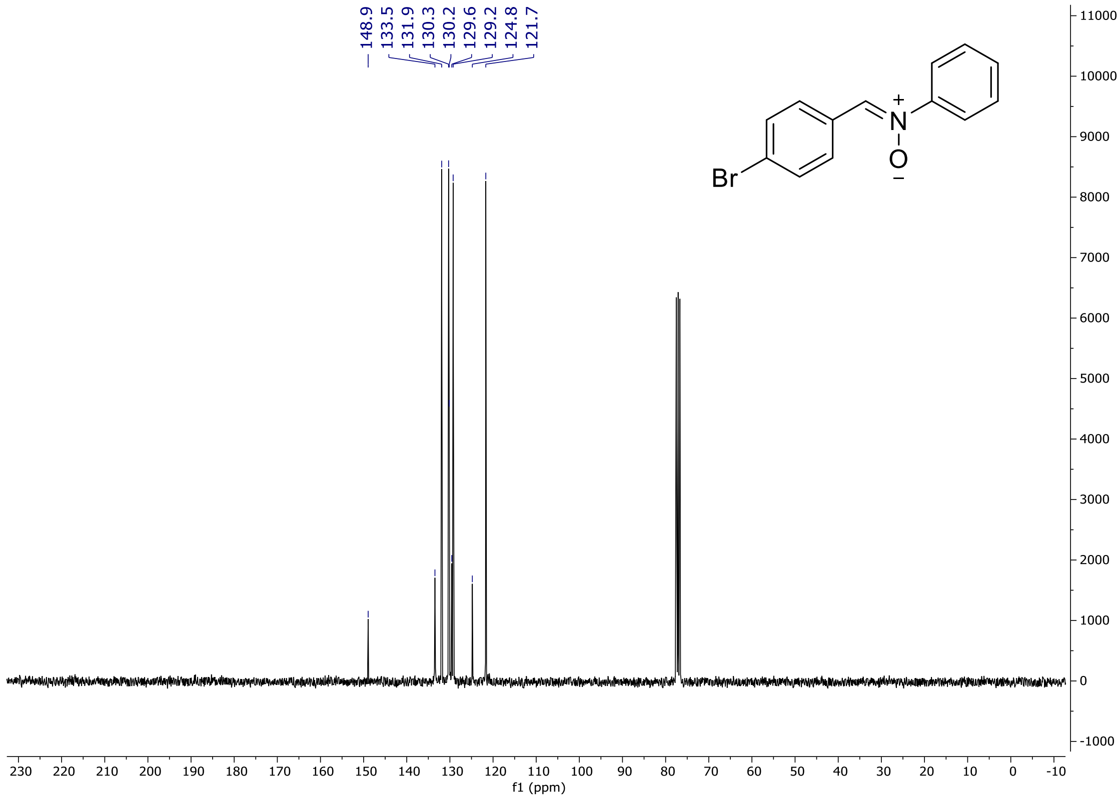

Figure SI_20: ^{13}C-NMR for **1d** in $CDCl_3$ (75 MHz).

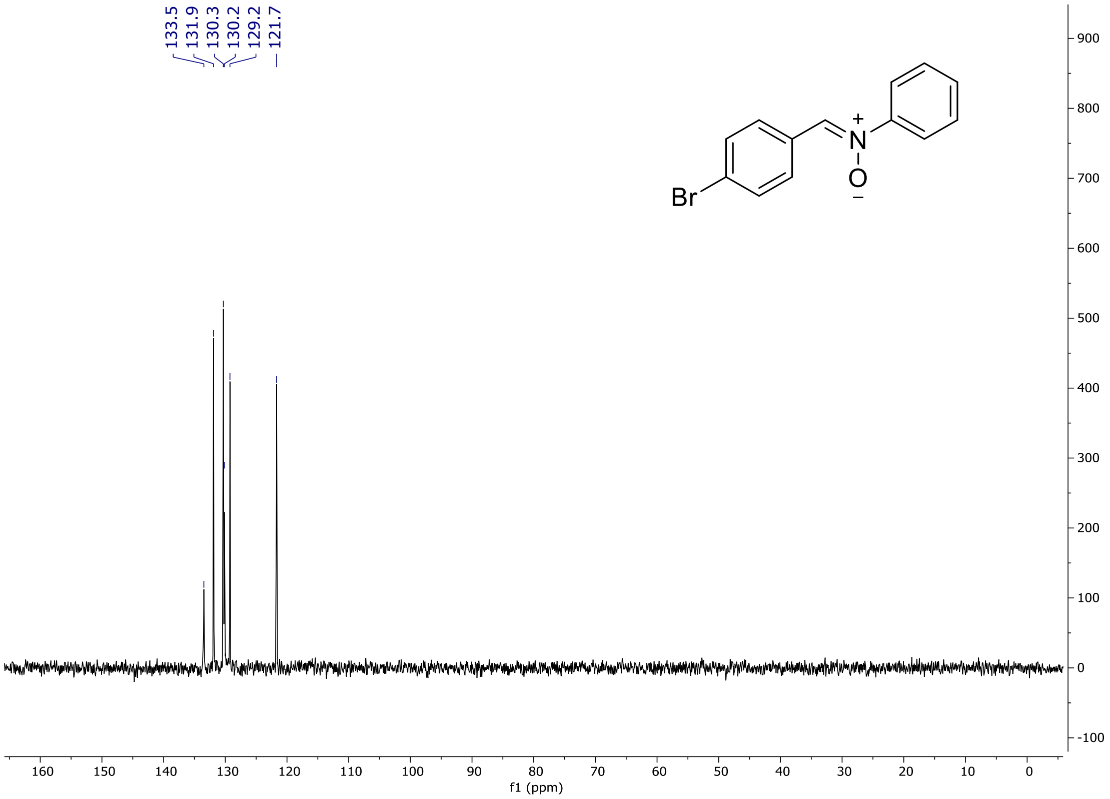

Figure SI_21: DEPT 135-NMR for **1d** in $CDCl_3$ (75 MHz).

***N*-(4-Iodobenzylidene)aniline oxide (1e)**

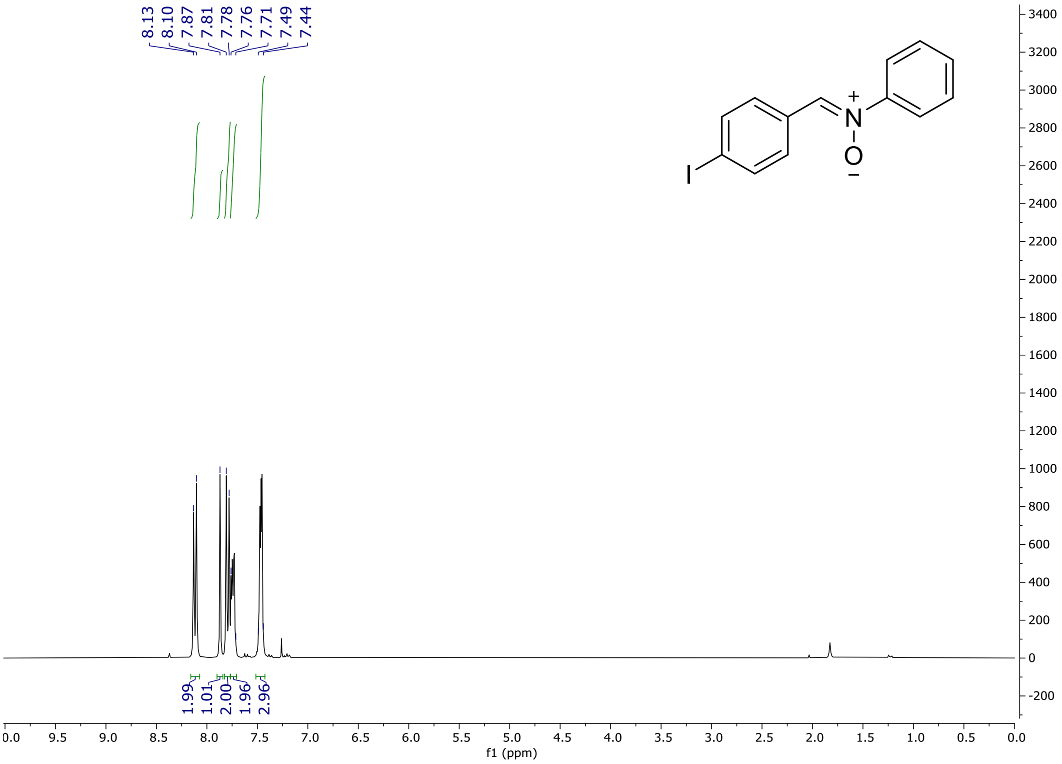

Figure SI_22: ^{1}H-NMR for **1e** in $CDCl_3$ (300 MHz).

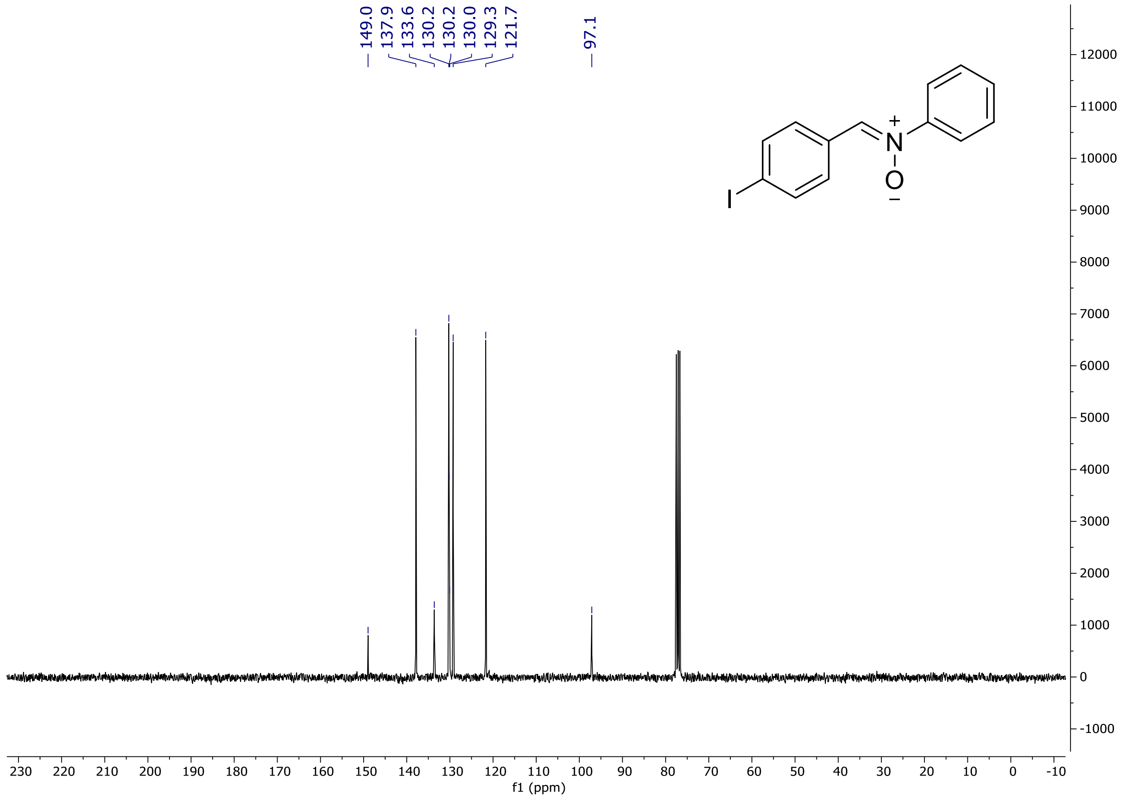

Figure SI_23: ^{13}C-NMR for **1e** in $CDCl_3$ (75 MHz).

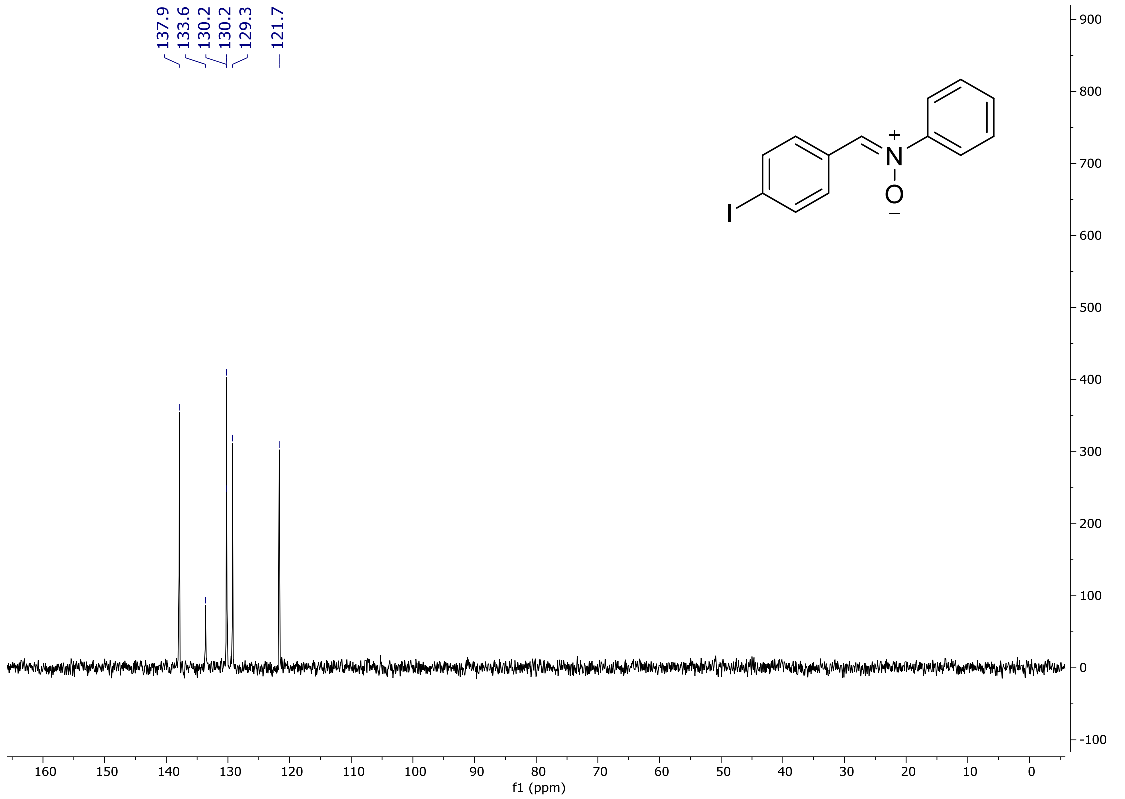

Figure SI_24: DEPT 135-NMR for **1e** in $CDCl_3$ (75 MHz).

N-(3-Chlorobenzylidene)aniline oxide (1f)

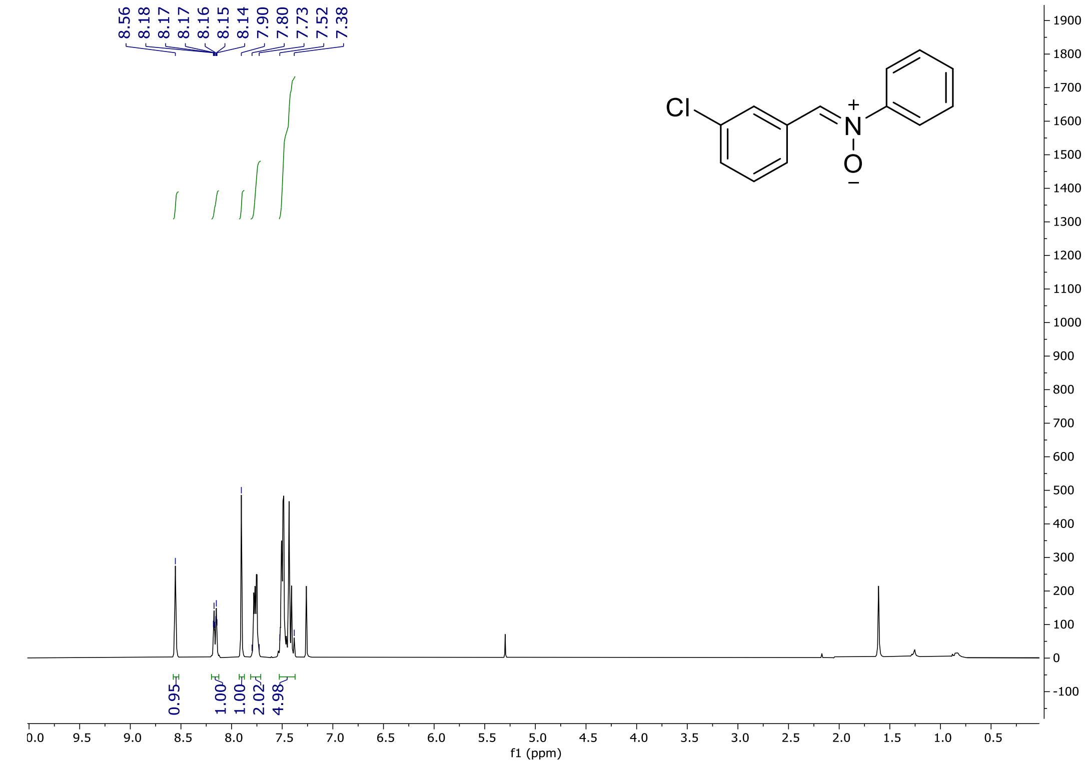

Figure SI_25: ^{1}H-NMR for **1f** in $CDCl_3$ (300 MHz).

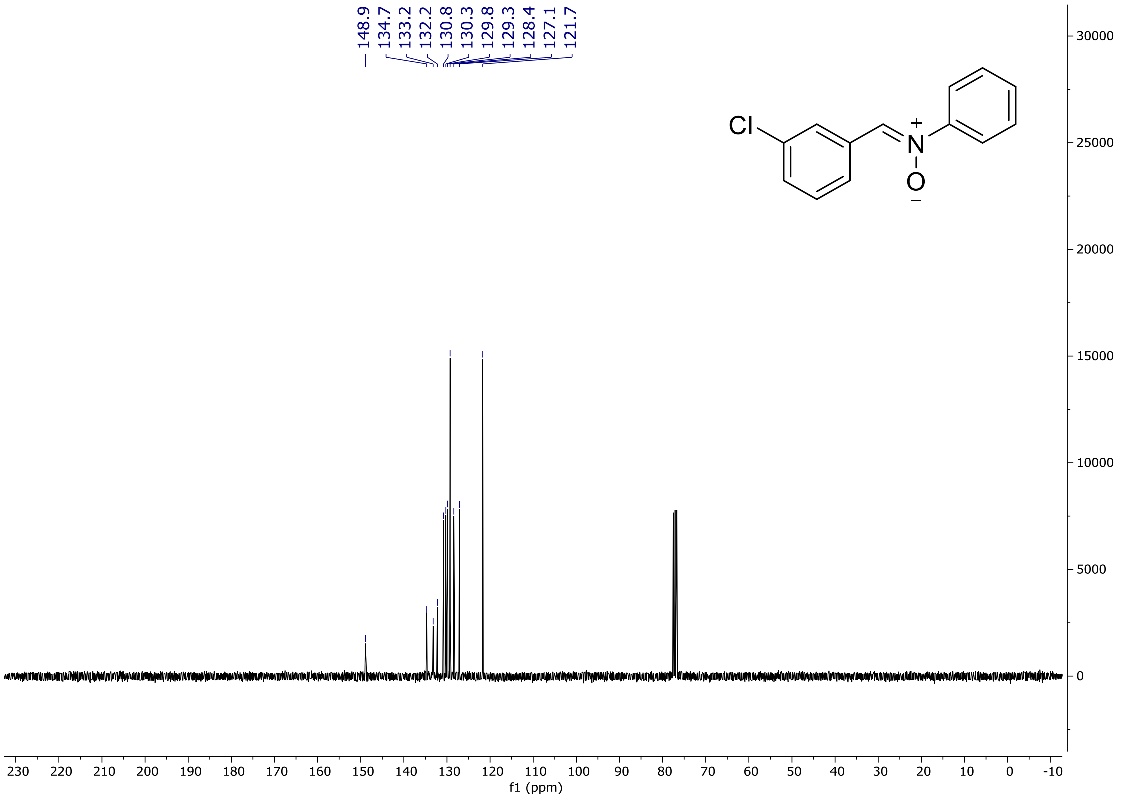

Figure SI_26: ^{13}C-NMR for **1f** in $CDCl_3$ (75 MHz).

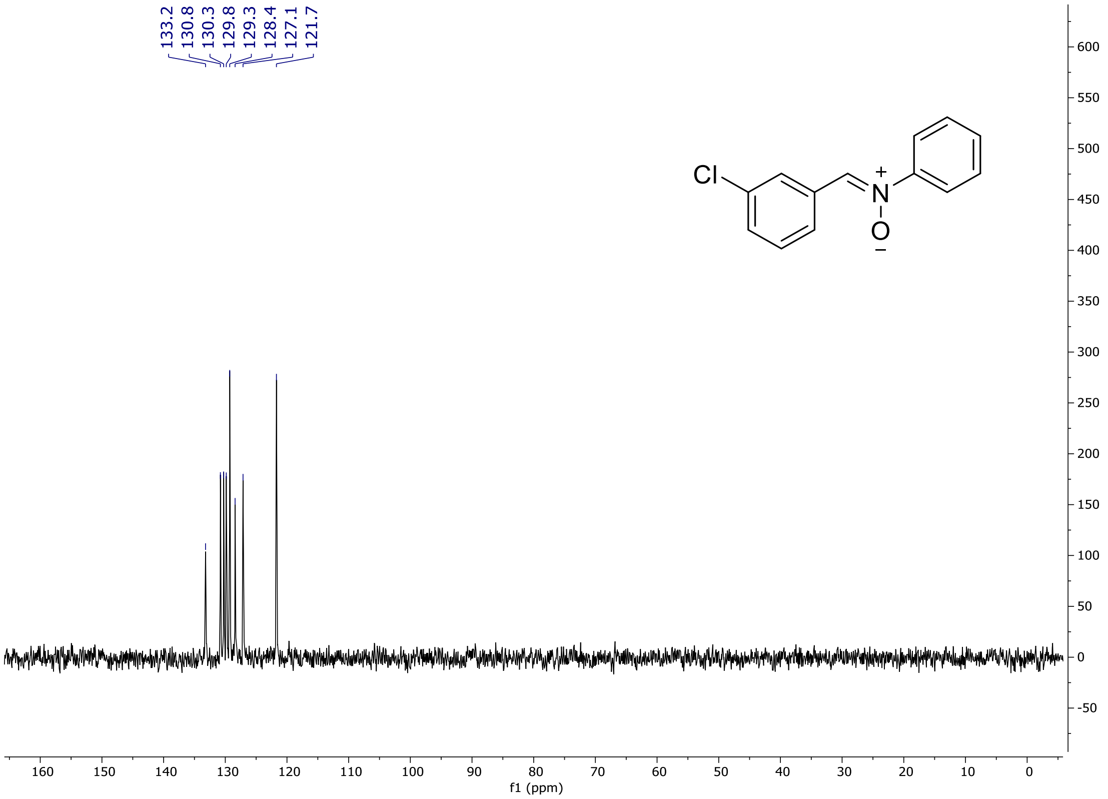

Figure SI_27: DEPT 135-NMR for **1f** in $CDCl_3$ (75 MHz).

***N*-(2-Chlorobenzylidene)aniline oxide (1g)**

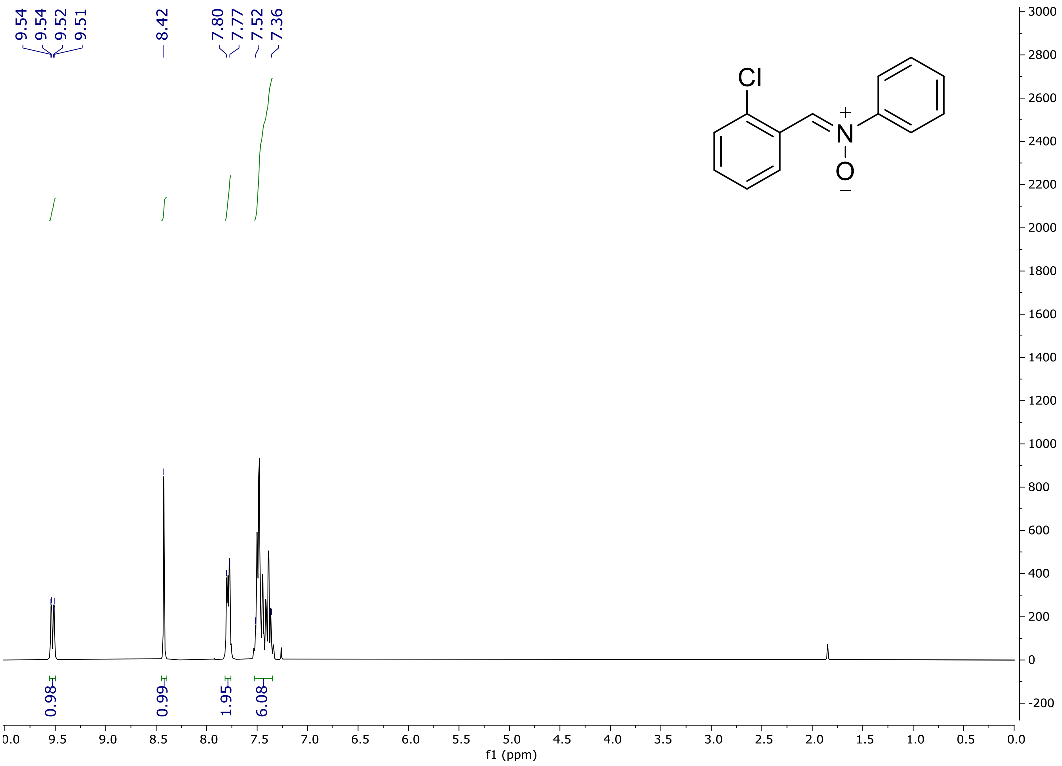

Figure SI_28: ^{1}H-NMR for **1g** in $CDCl_3$ (300 MHz).

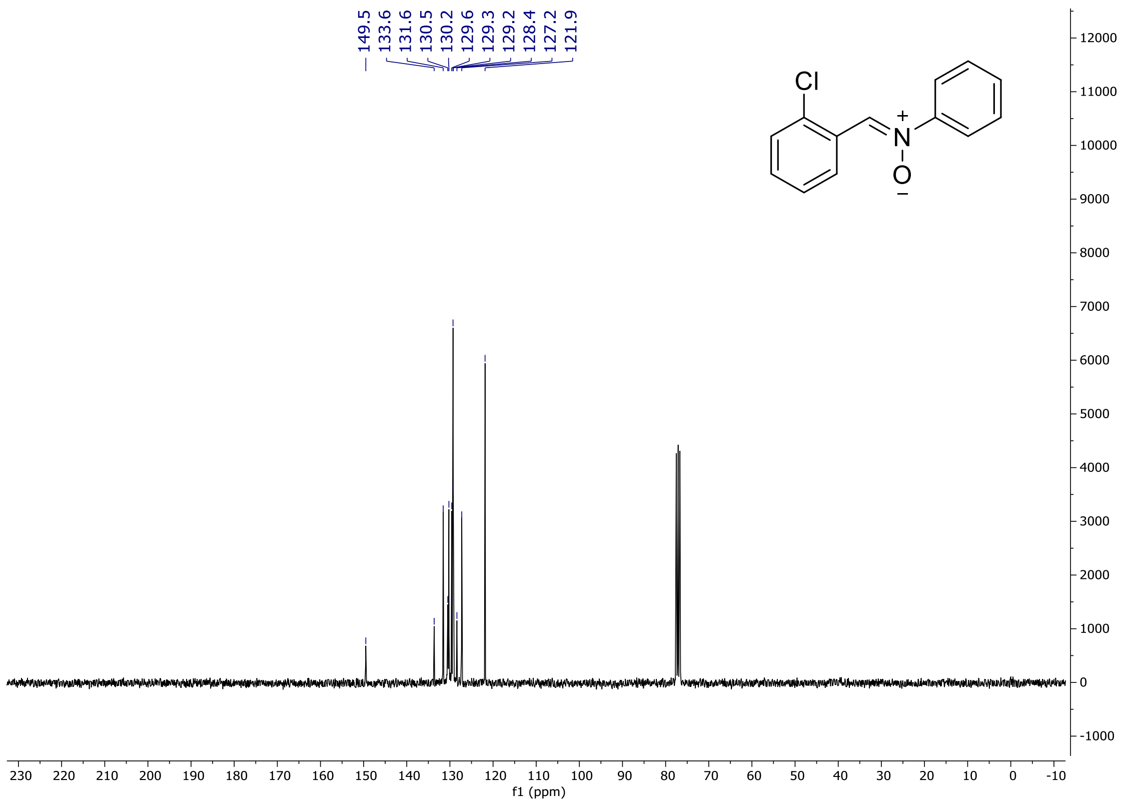

Figure SI_29: ^{13}C-NMR for **1g** in $CDCl_3$ (75 MHz).

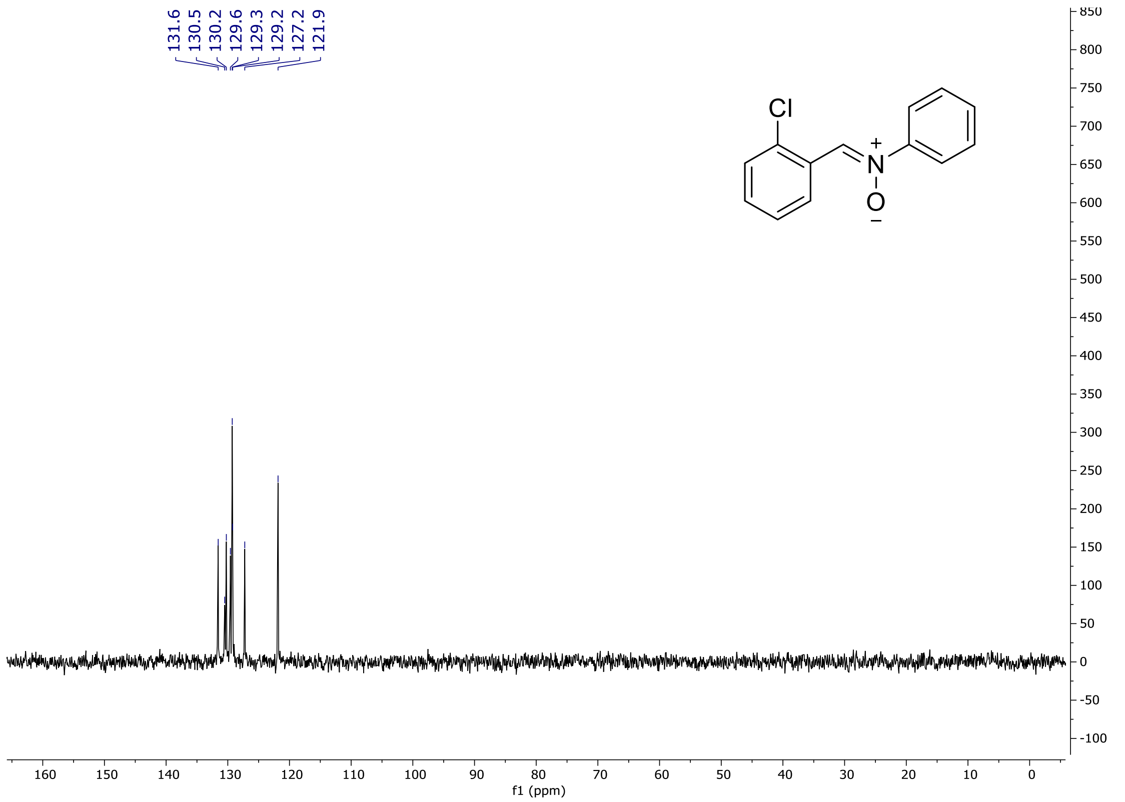

Figure SI_30: DEPT 135-NMR for **1g** in $CDCl_3$ (75 MHz).

N-(3,4-Dichlorobenzylidene)aniline oxide (1h)

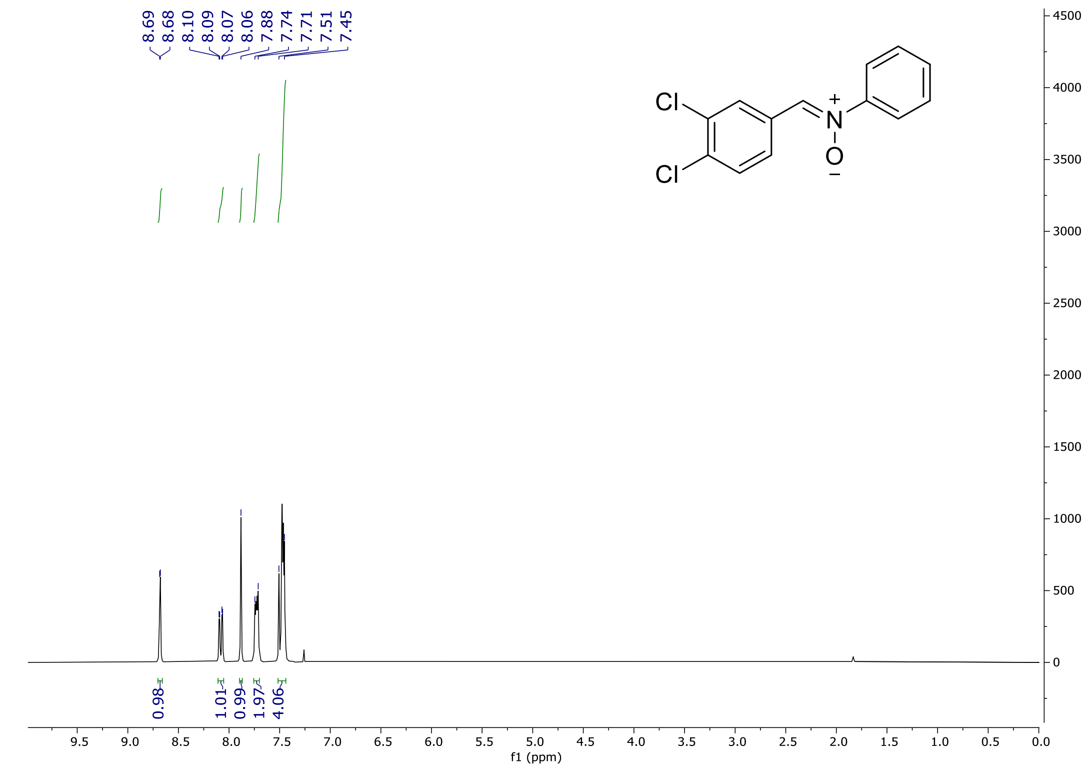

Figure SI_31: ^{1}H-NMR for **1h** in $CDCl_3$ (300 MHz).

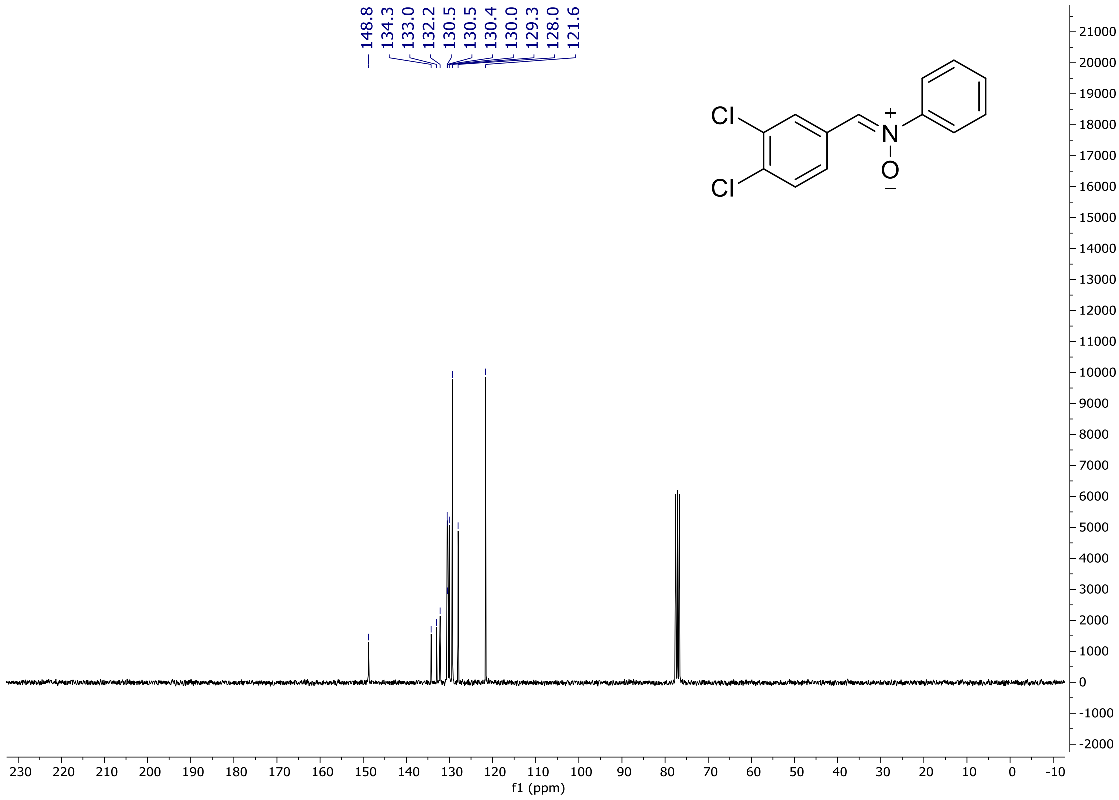

Figure SI_32: ^{13}C-NMR for **1h** in $CDCl_3$ (75 MHz).

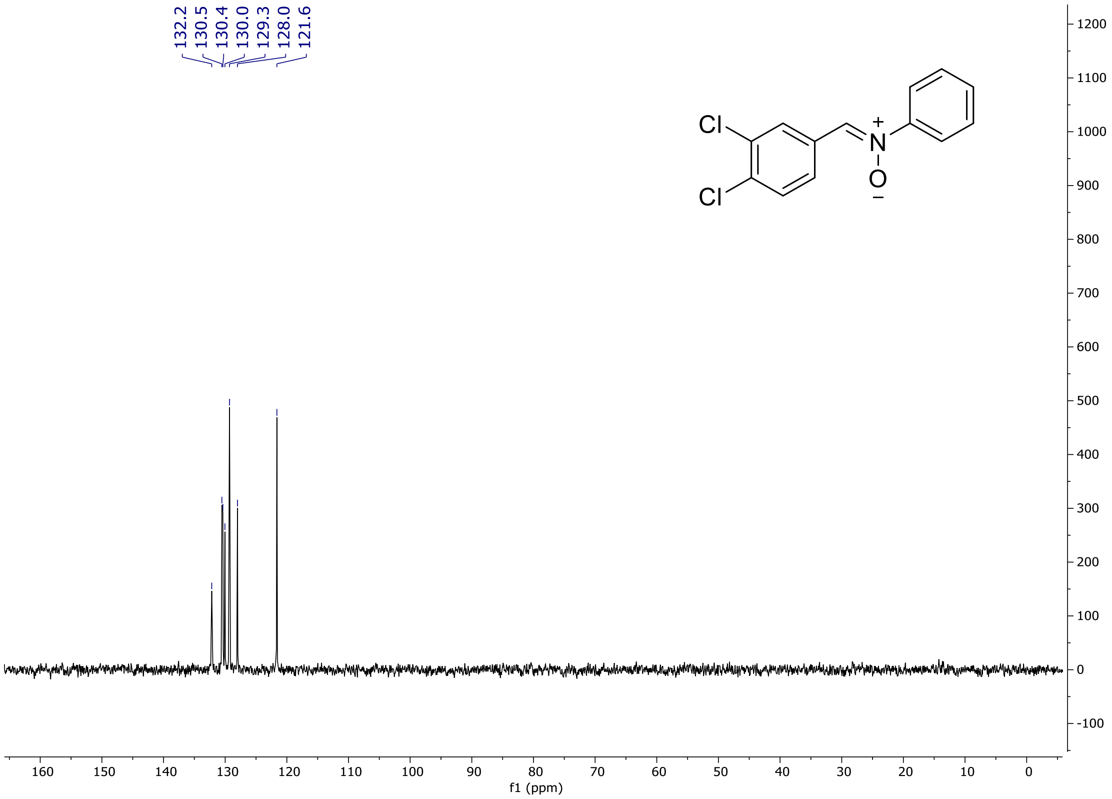

Figure SI_33: DEPT 135-NMR for **1h** in $CDCl_3$ (75 MHz).

***N*-[4-(Trifluoromethyl)benzylidene]aniline oxide (1i)**

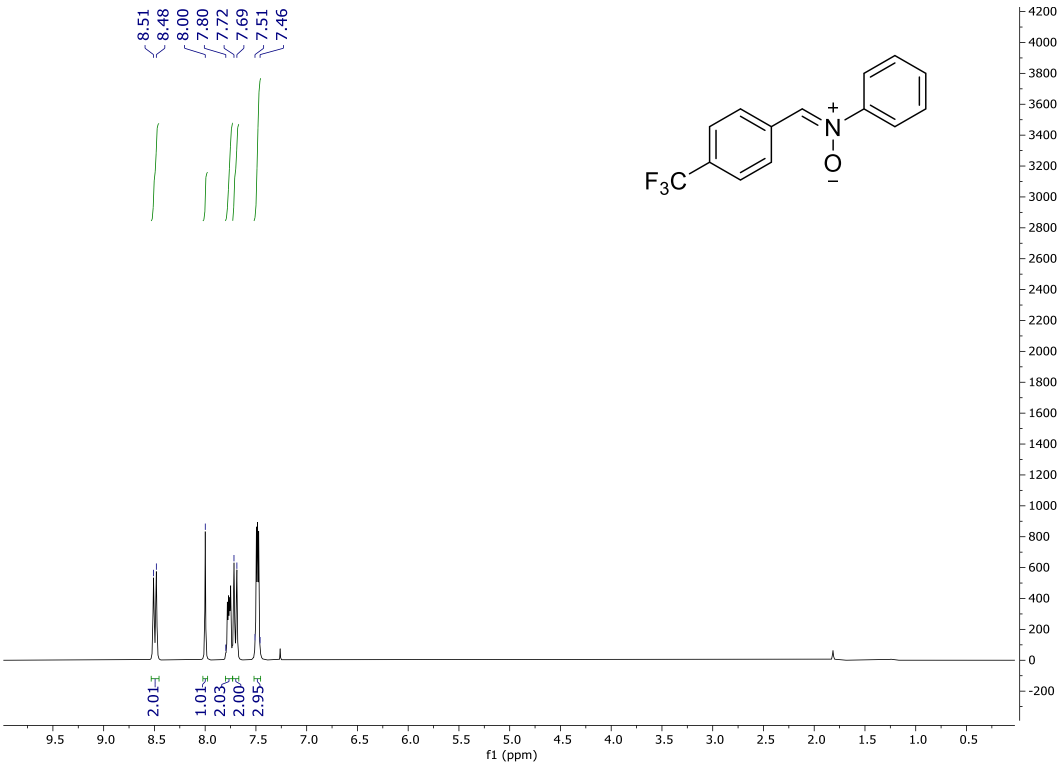

Figure SI_34: ^{1}H-NMR for **1i** in $CDCl_3$ (300 MHz).

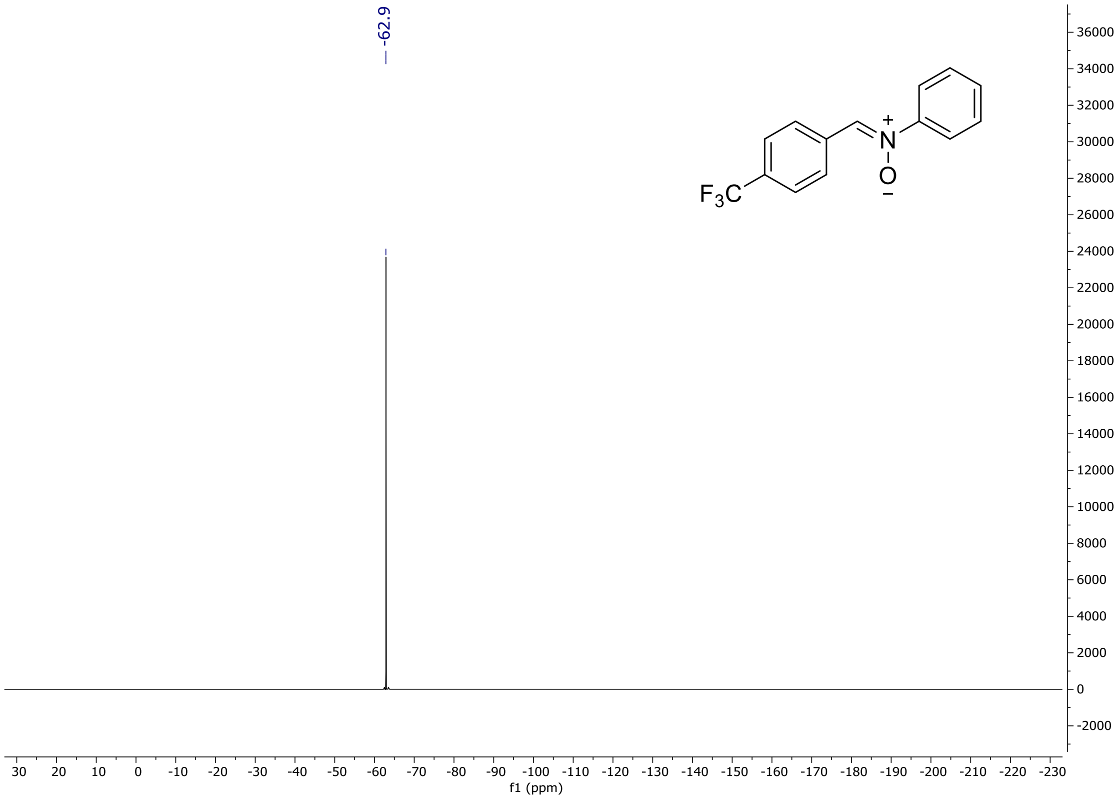

Figure SI_35: ^{19}F-NMR for **1i** in $CDCl_3$ (282 MHz).

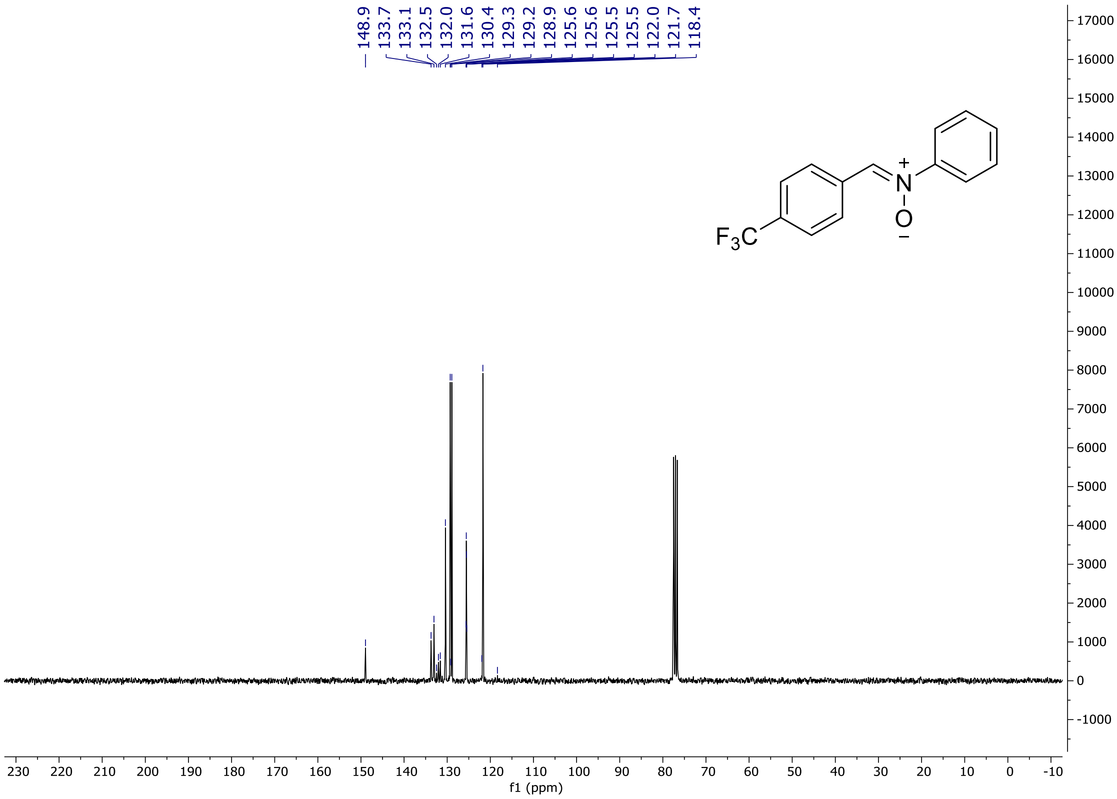

Figure SI_36: ^{13}C-NMR for **1i** in $CDCl_3$ (75 MHz).

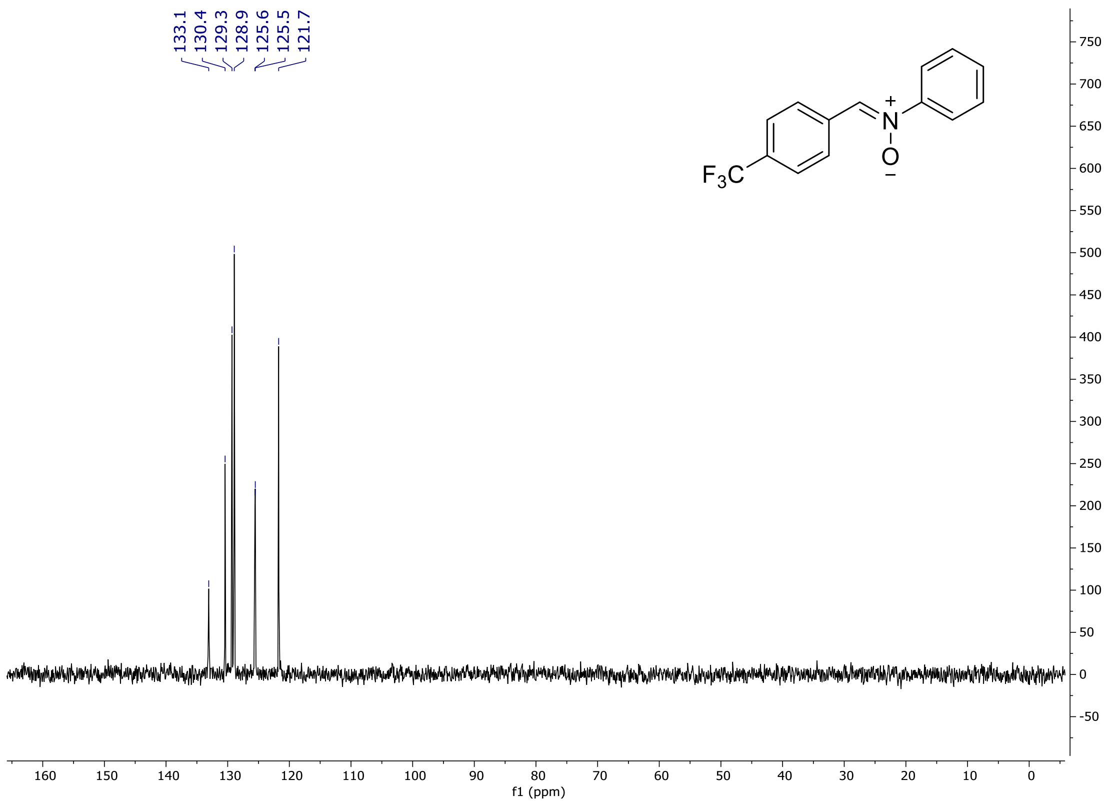

Figure SI_37: DEPT 135-NMR for **1i** in $CDCl_3$ (75 MHz).

***N*-(4-Nitrobenzylidene)aniline oxide (1j)**

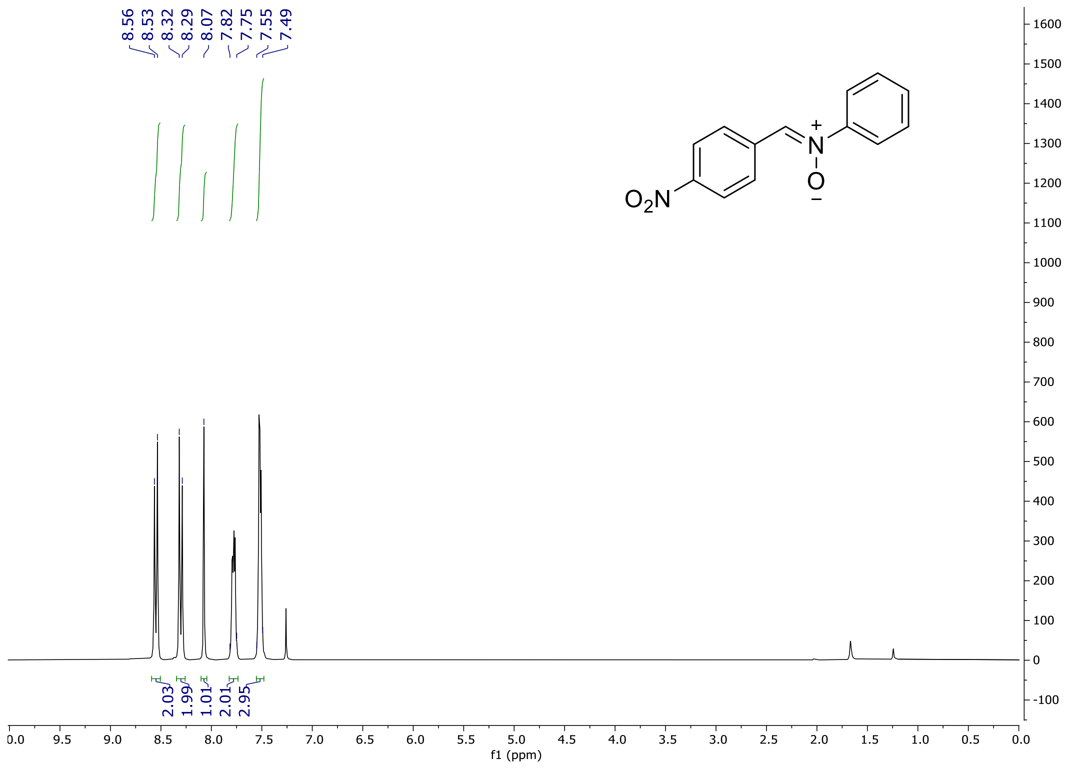

Figure SI_38: ^{1}H-NMR for **1j** in $CDCl_3$ (300 MHz).

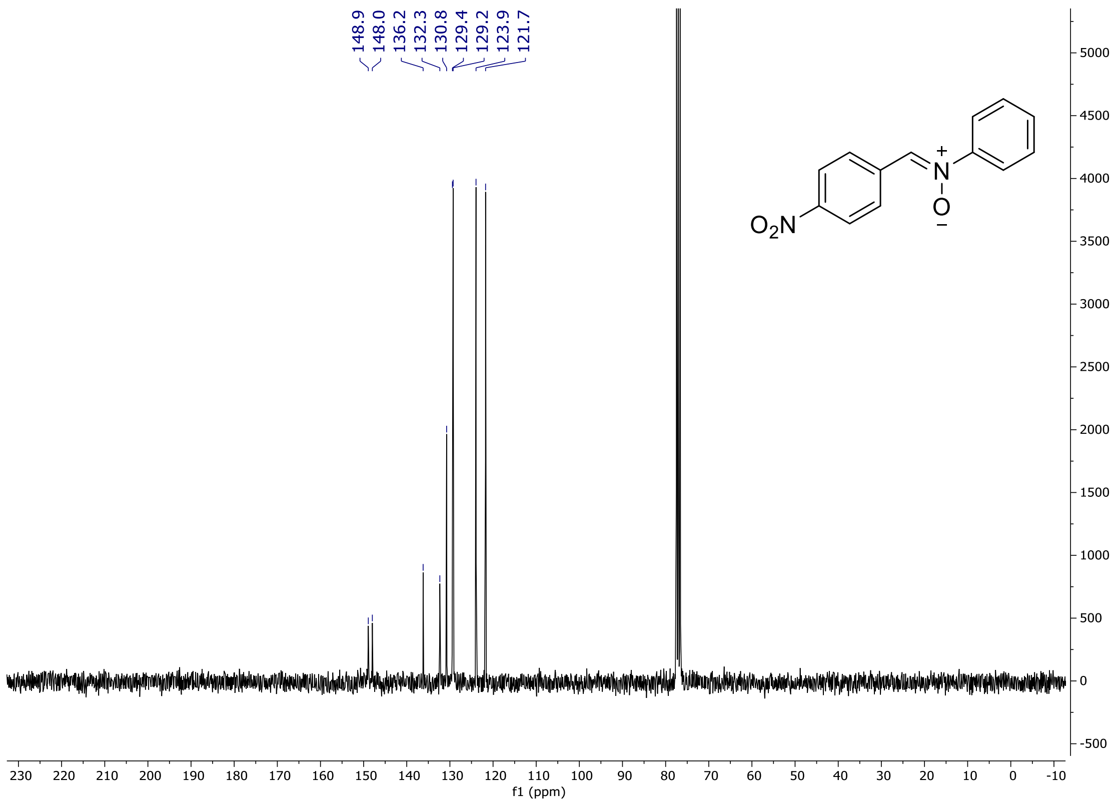

Figure SI_39: ^{13}C-NMR for **1j** in $CDCl_3$ (75 MHz).

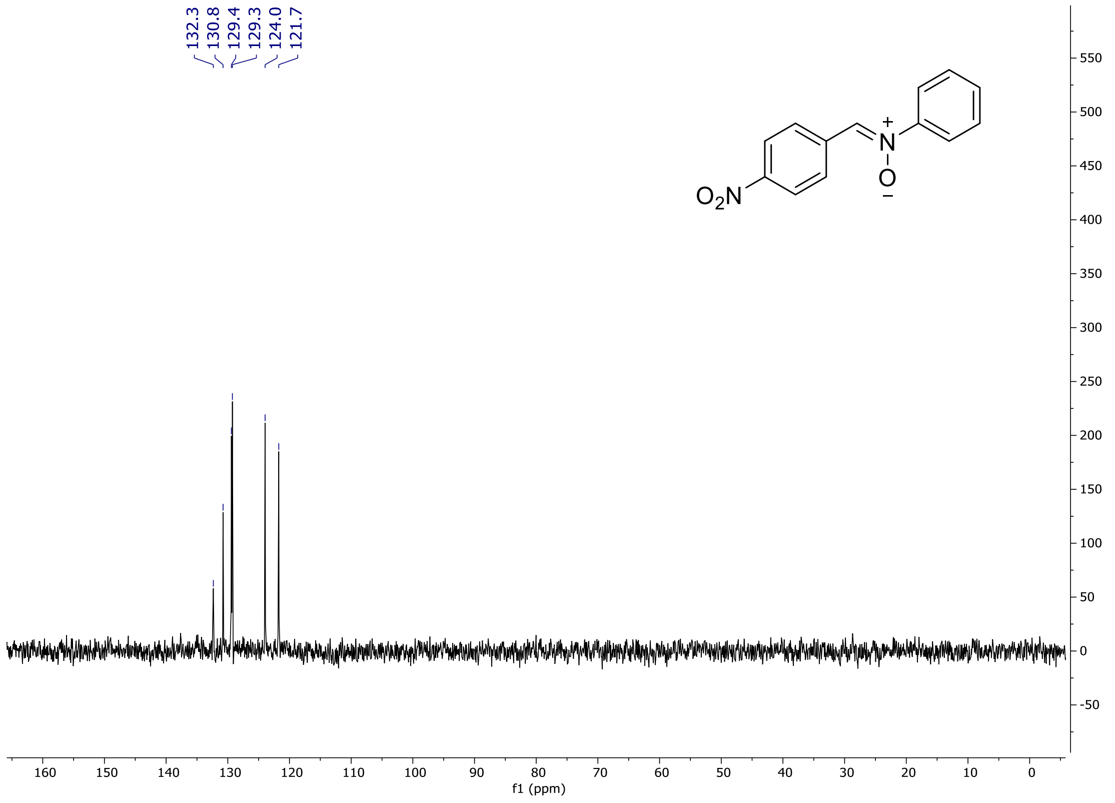

Figure SI_40: DEPT 135-NMR for **1j** in $CDCl_3$ (75 MHz).

***N*-(4-Cyanobenzylidene)aniline oxide (1k)**

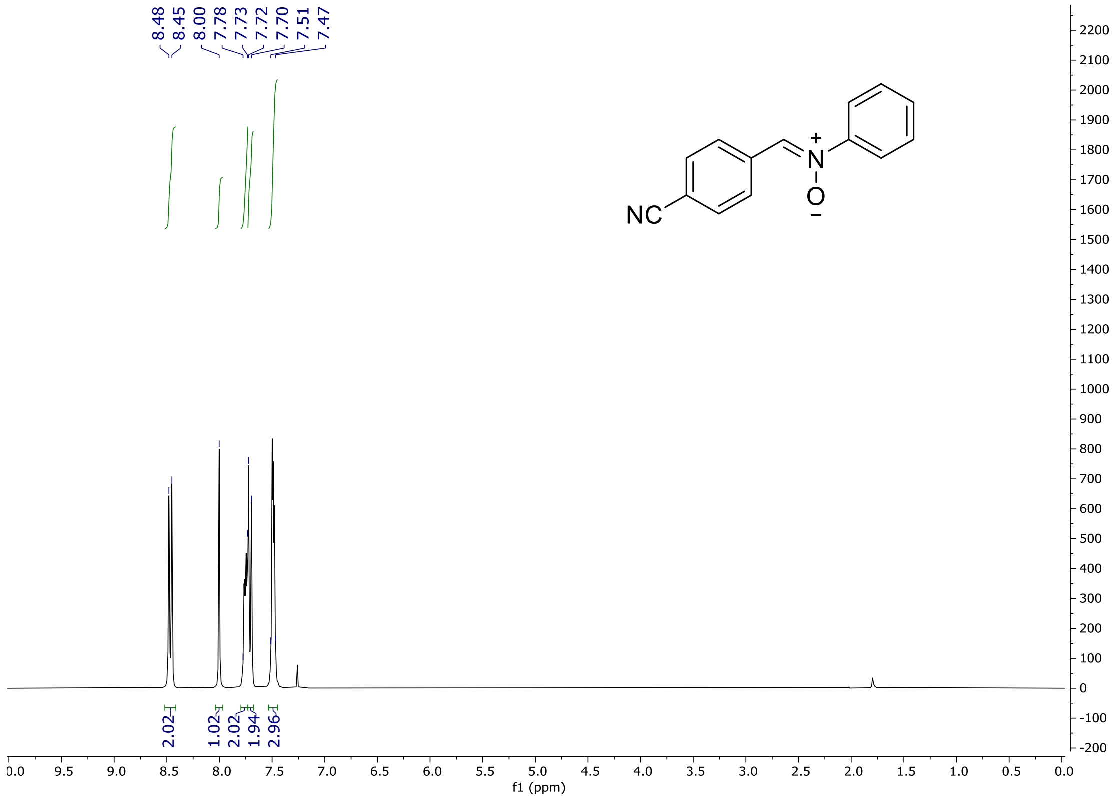

Figure SI_41: ^{1}H-NMR for **1k** in $CDCl_3$ (300 MHz).

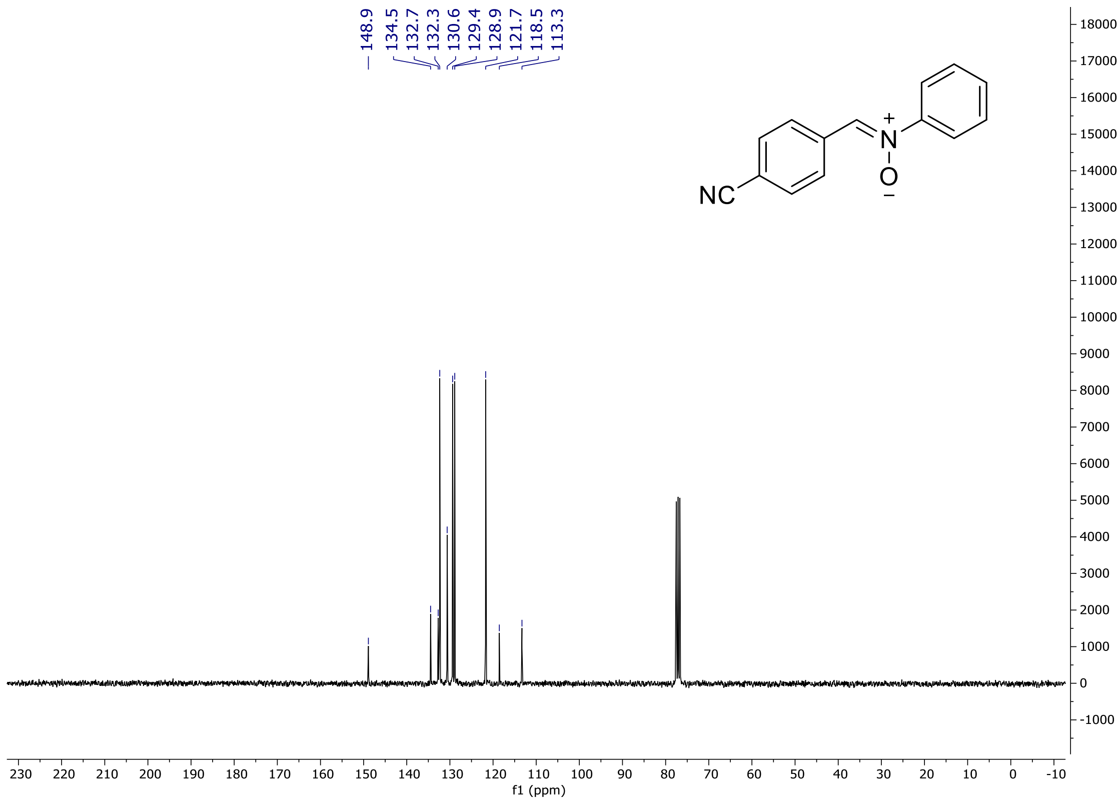

Figure SI_42: ^{13}C-NMR for **1k** in $CDCl_3$ (75 MHz).

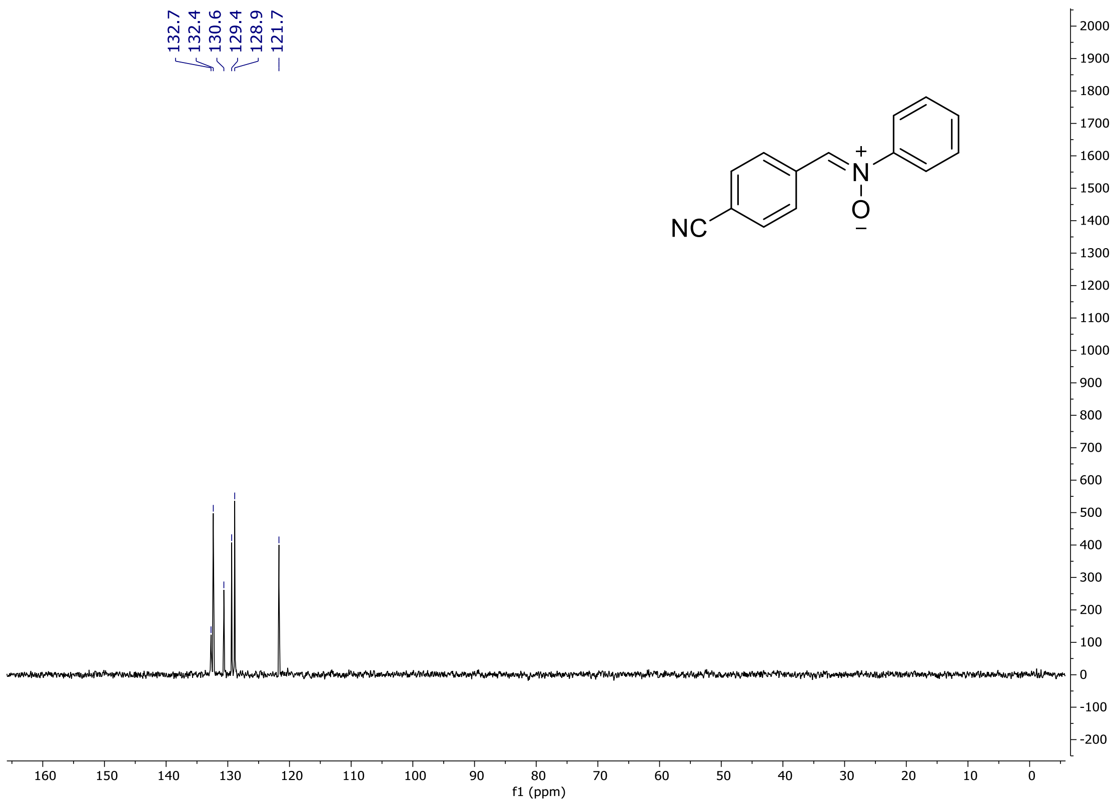

Figure SI_43: DEPT 135-NMR for **1k** in $CDCl_3$ (75 MHz).

***N*-(4-Ethynylbenzylidene)aniline oxide (1l)**

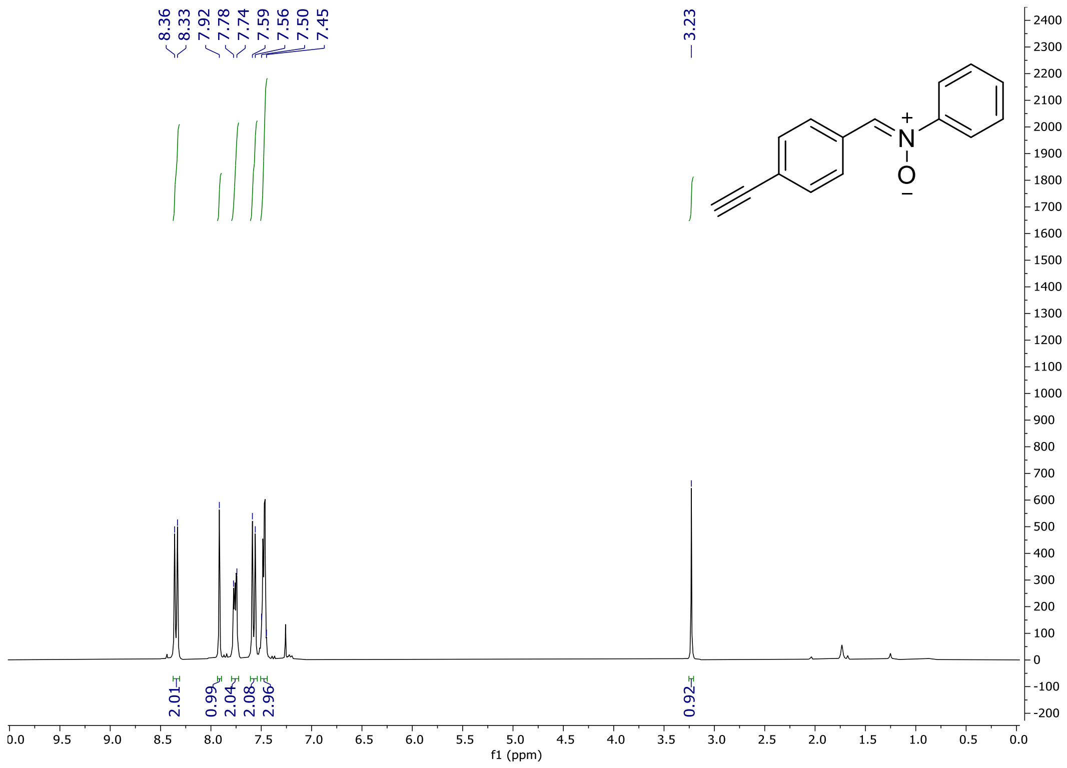

Figure SI_44: ^{1}H-NMR for **1l** in $CDCl_3$ (300 MHz).

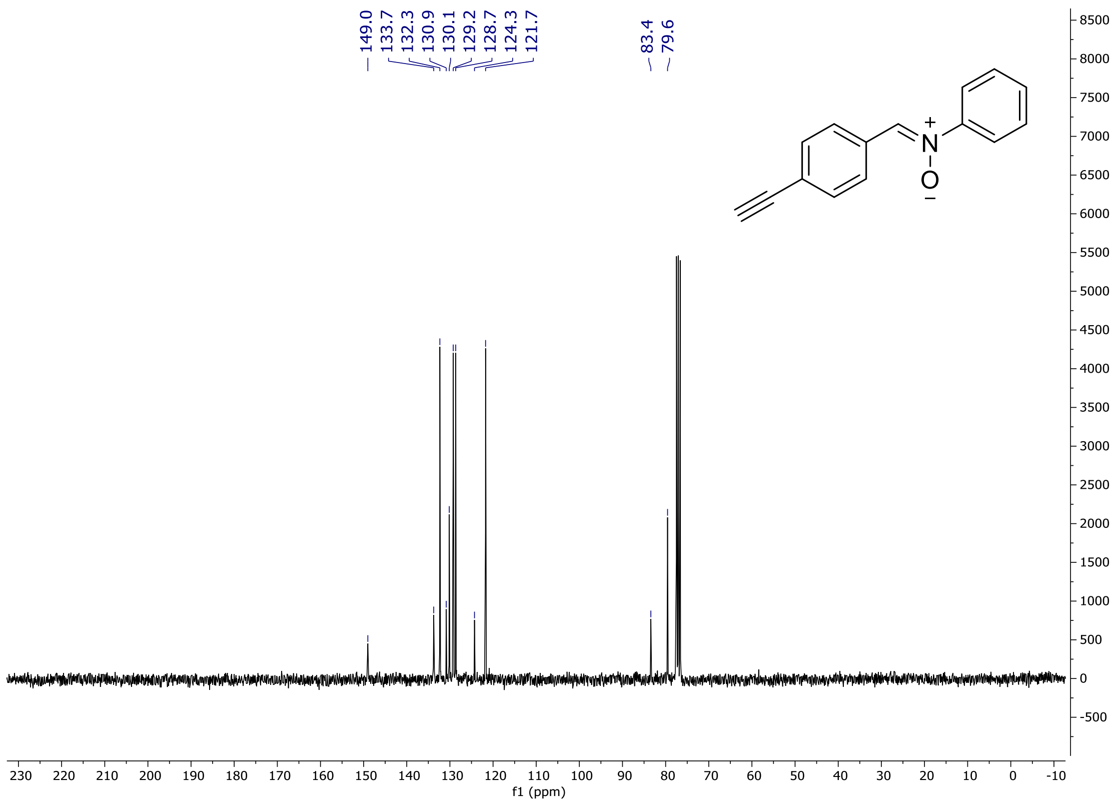

Figure SI_45: ^{13}C-NMR for **1l** in $CDCl_3$ (75 MHz).

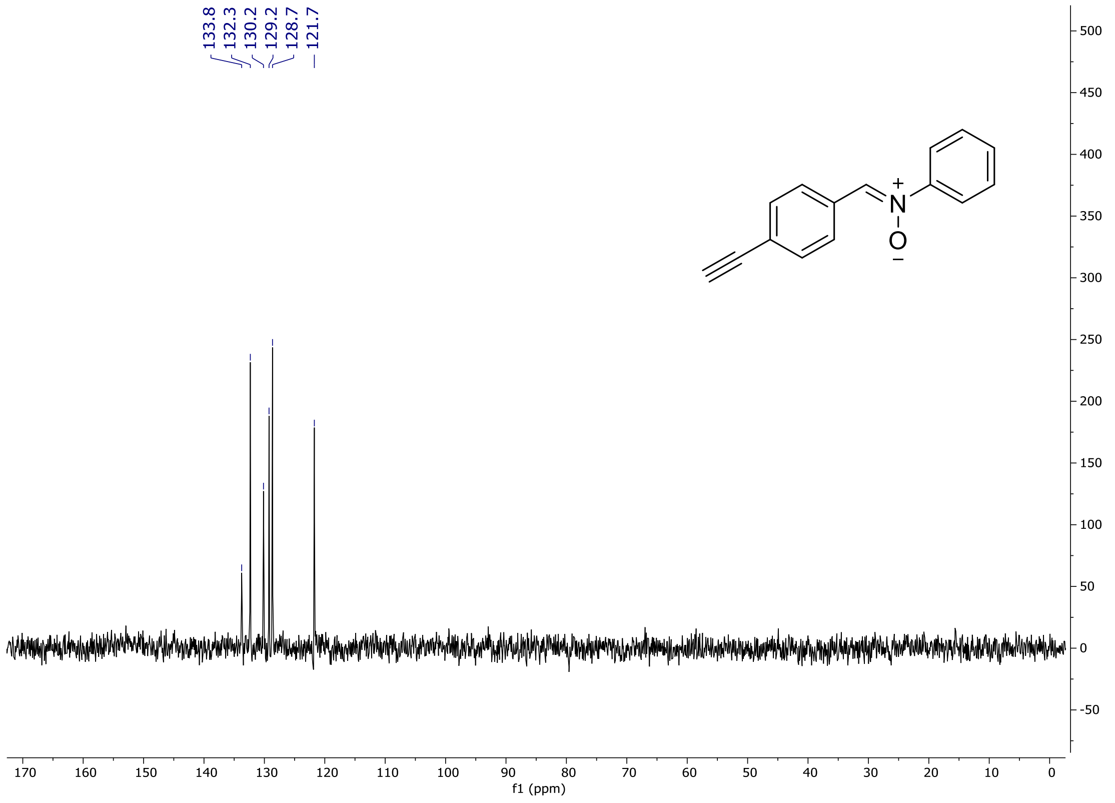

Figure SI_46: DEPT 135-NMR for **1l** in $CDCl_3$ (75 MHz).

N-[4-(4,4,5,5-Tetramethyl-1,3,2-dioxaborolan-2-yl)benzylidene]aniline oxide (1m)

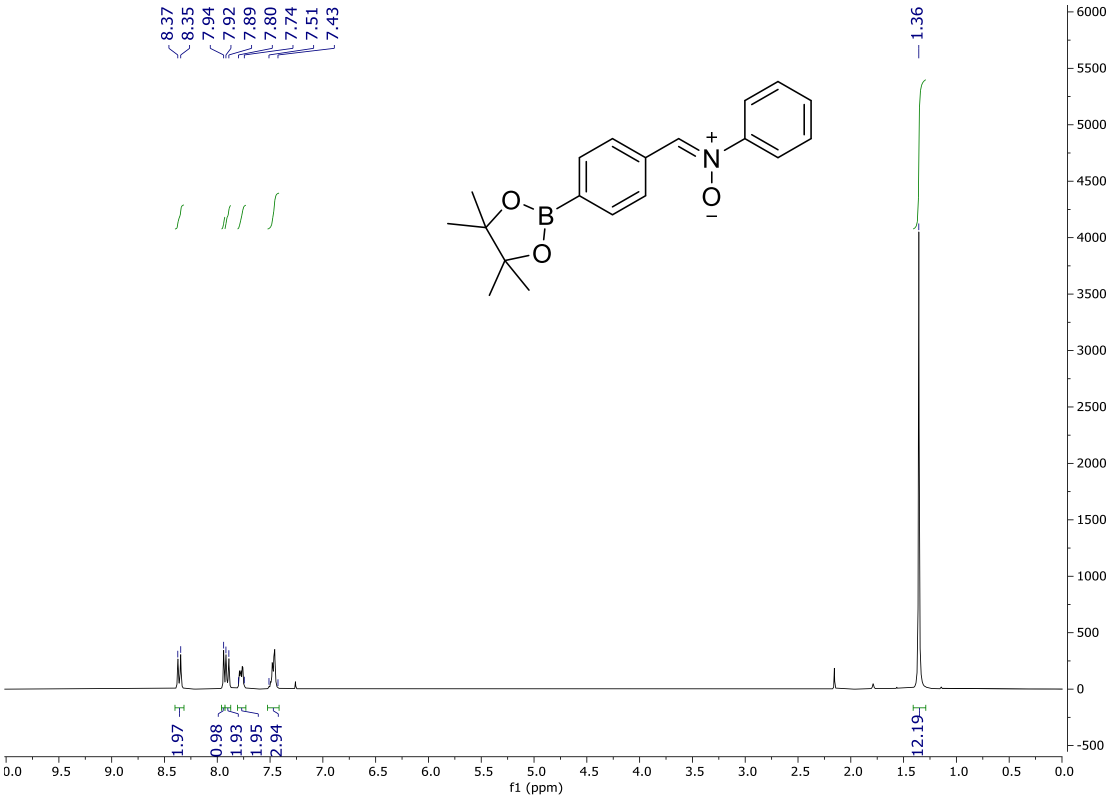

Figure SI_47: ^{1}H-NMR for **1m** in $CDCl_3$ (300 MHz).

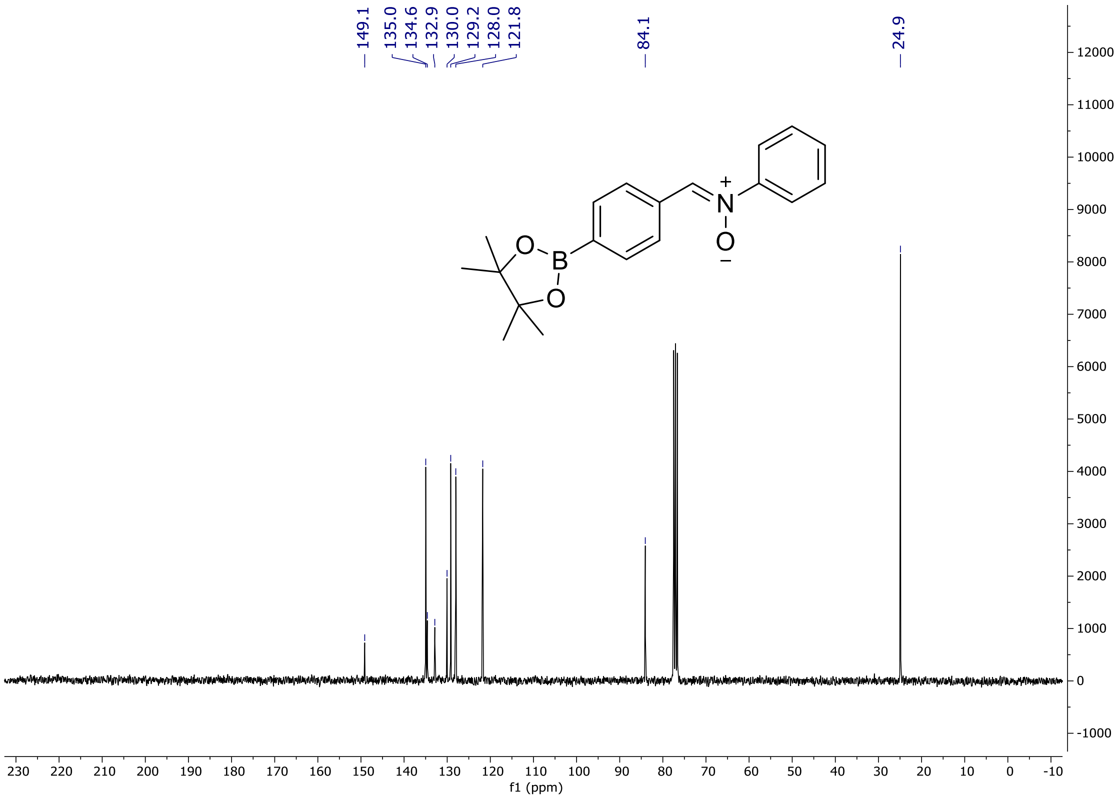

Figure SI_48: ^{13}C-NMR for **1m** in $CDCl_3$ (75 MHz).

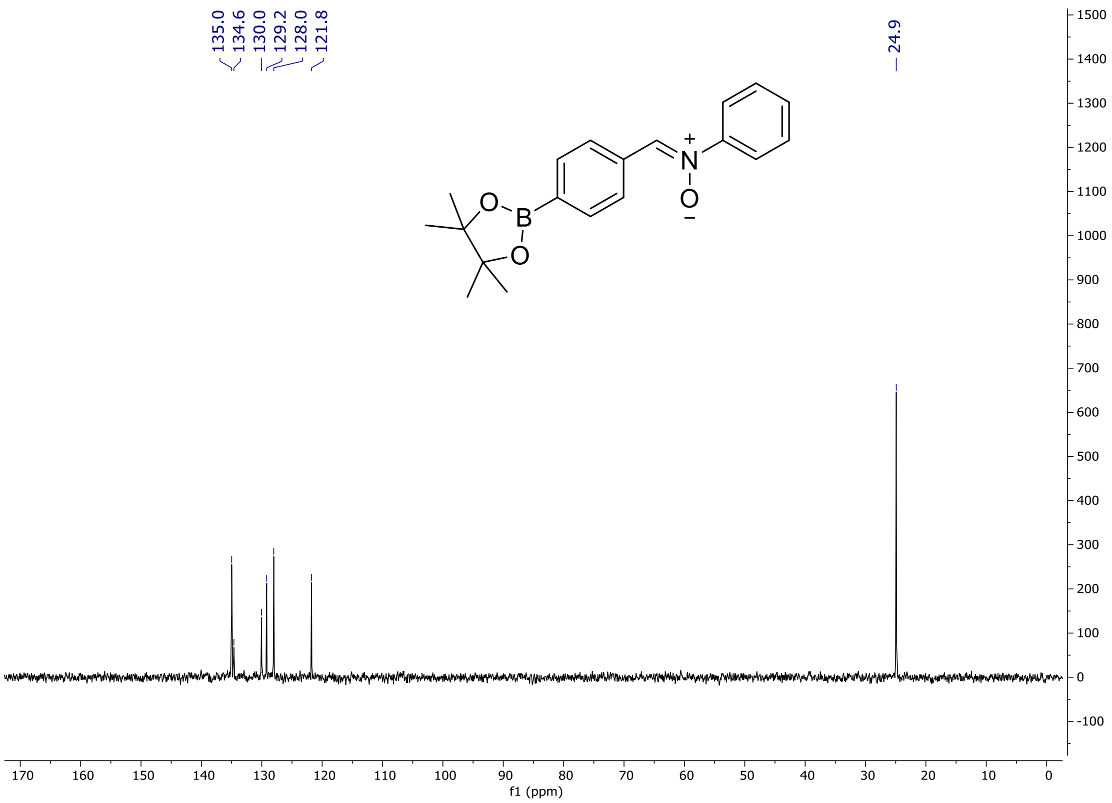

Figure SI_49: DEPT 135-NMR for **1m** in $CDCl_3$ (75 MHz).

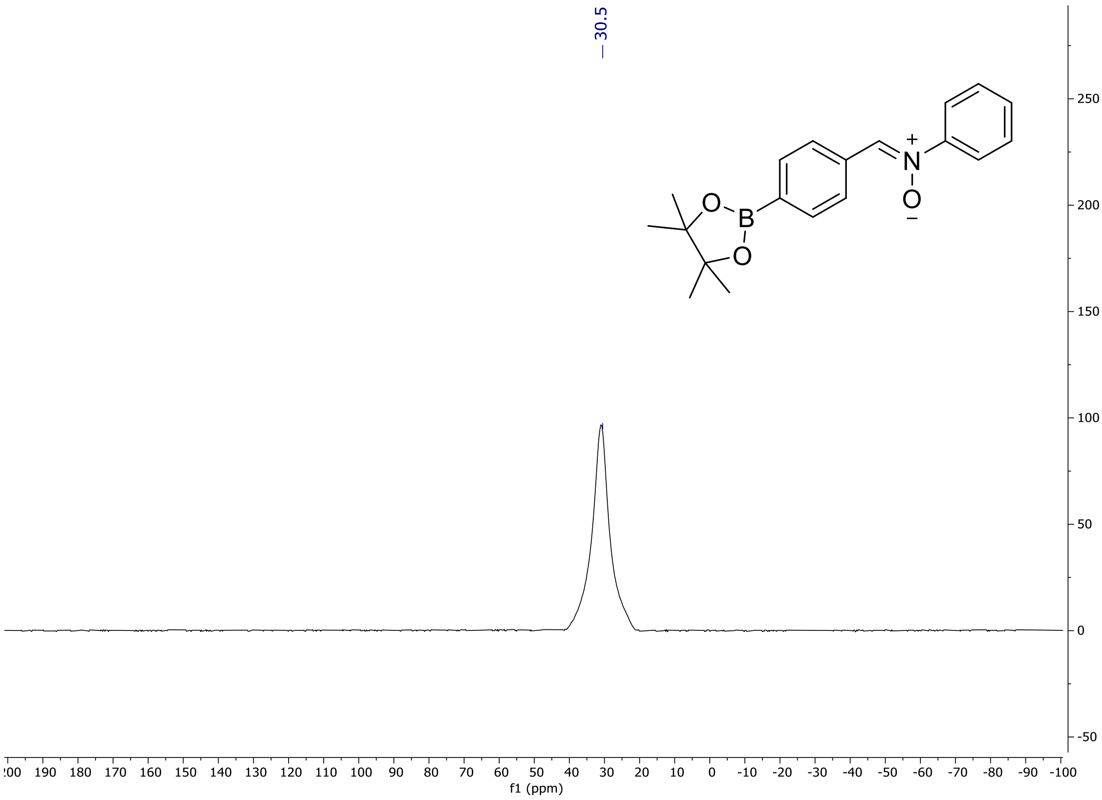

Figure SI_50: ^{11}B-NMR for **1m** in $CDCl_3$ (129 MHz)**.**

N-(4-Acetylbenzylidene)aniline oxide (1n)

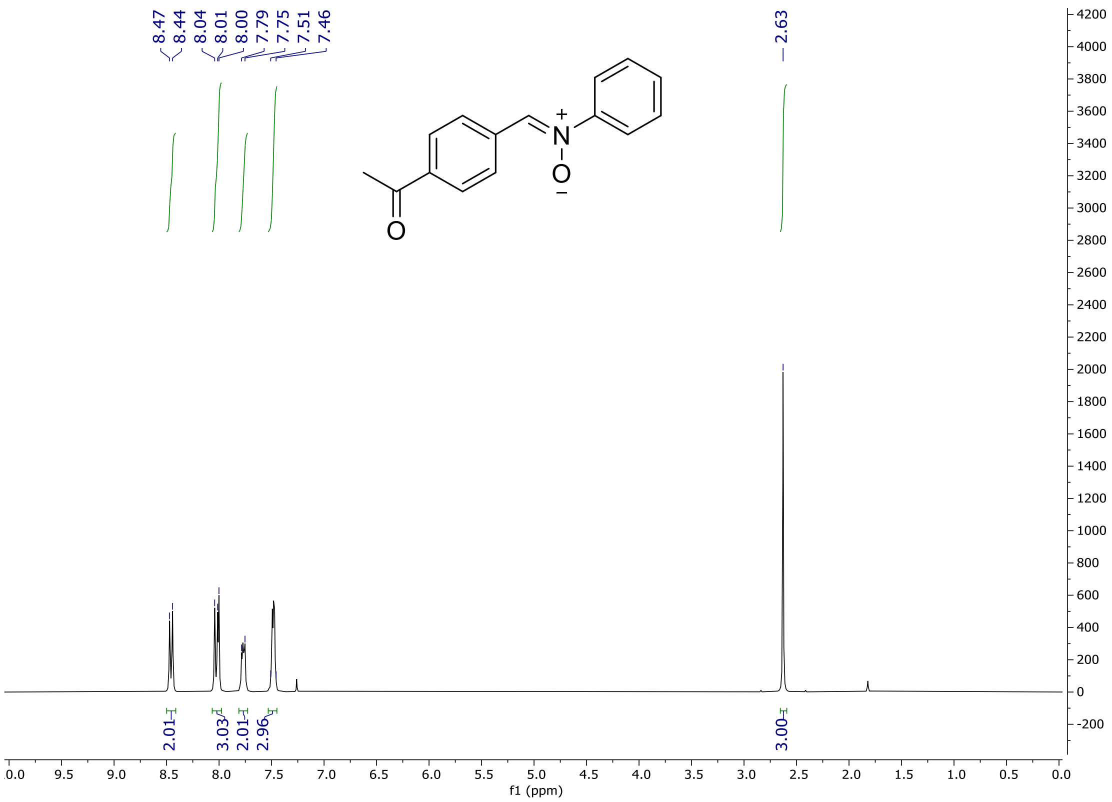

Figure SI_51: ^{1}H-NMR for **1n** in $CDCl_3$ (300 MHz).

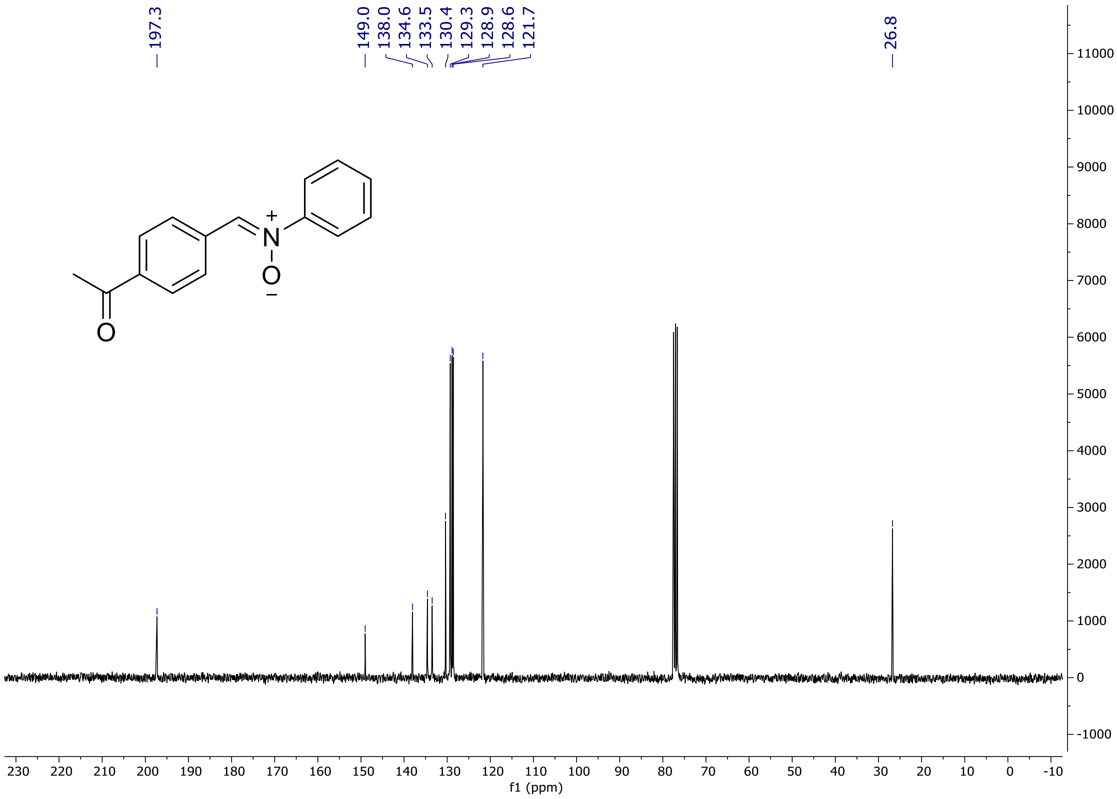

Figure SI_52: ^{13}C-NMR for **1n** in $CDCl_3$ (75 MHz).

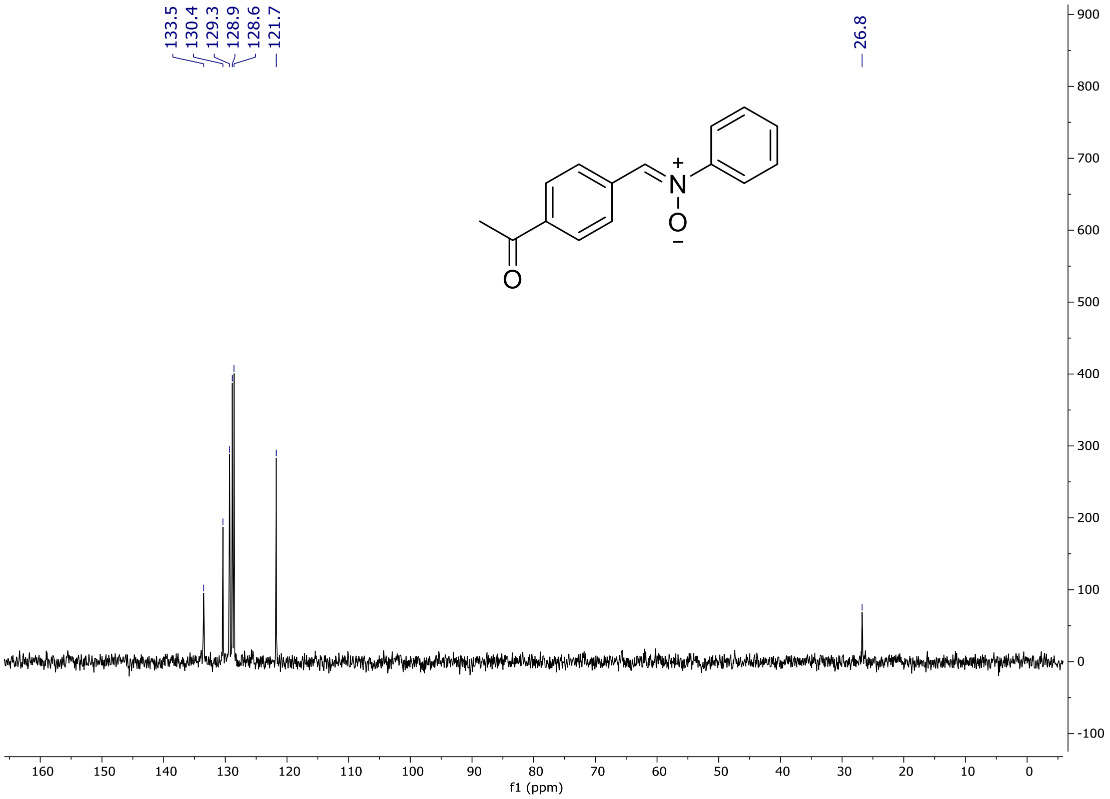

Figure SI_53: DEPT 135-NMR for **1n** in $CDCl_3$ (75 MHz).

Acquisition Parameter

Source Type	ESI	Ion Polarity	Positive	Set Nebulizer	2.4 Bar
Focus	Active	Set Capillary	3500 V	Set Dry Heater	250 °C
Scan Begin	50 m/z	Set End Plate Offset	-500 V	Set Dry Gas	6.0 l/min
Scan End	1500 m/z	Set Charging Voltage	2000 V	Set Divert Valve	Source
		Set Corona	0 nA	Set APCI Heater	0 °C

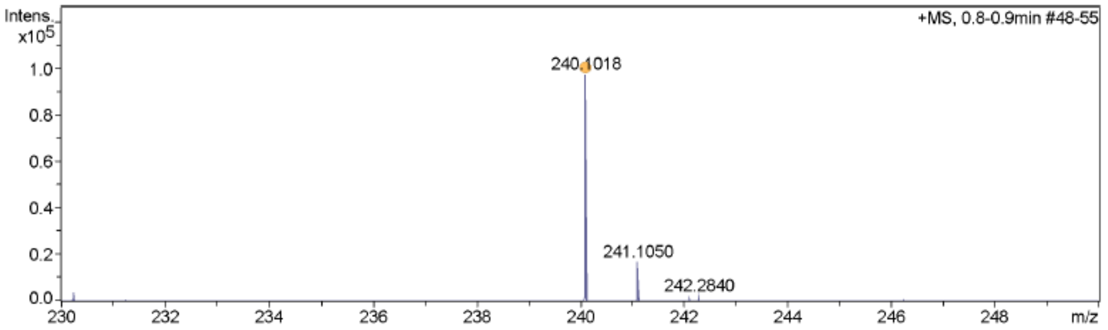

Meas. m/z	#	Ion Formula	m/z	err [ppm]	mSigma	# mSigma	Score	rdb	e⁻ Conf	N-Rule	Adduct
240.1018	1	C15H14NO2	240.1019	0.6	3.5	2	100.00	10.0	even	ok	M+H

Figure SI_54: HRMS (ESI^+, m/z) analysis of **1n**.

N-[4-(Methoxycarbonyl)benzylidene]aniline oxide (1o)

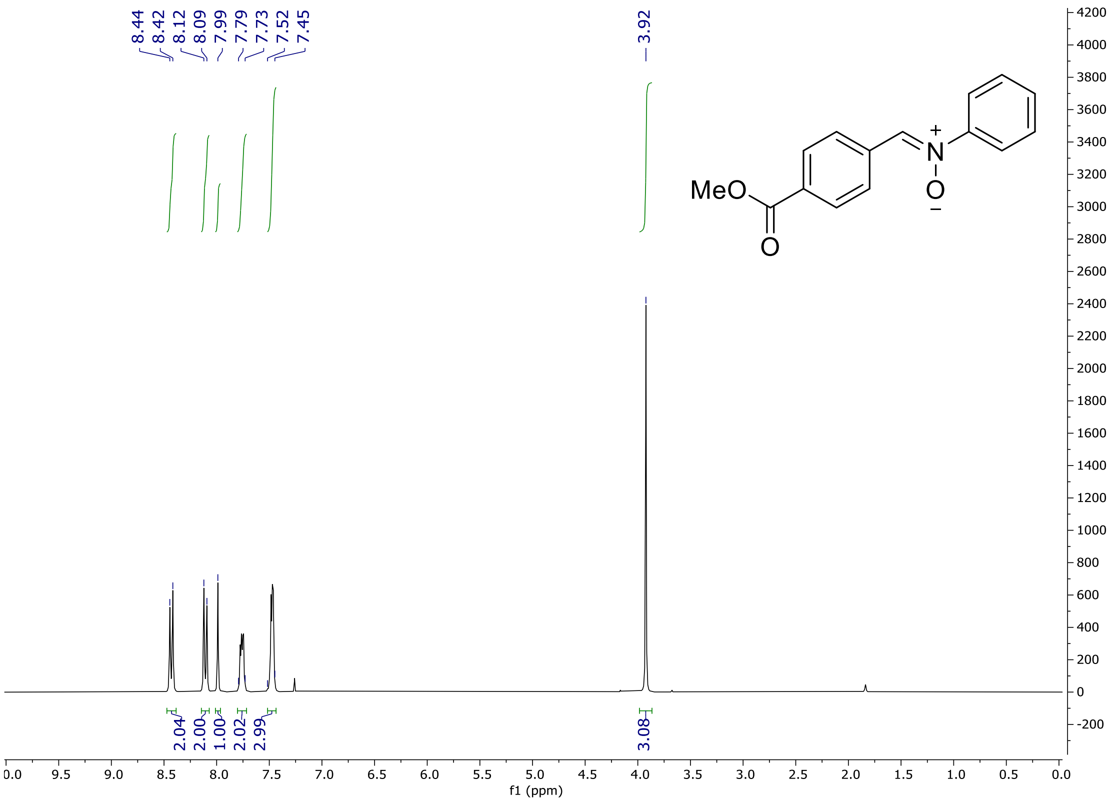

Figure SI_55: ^{1}H-NMR for **1o** in $CDCl_3$ (300 MHz).

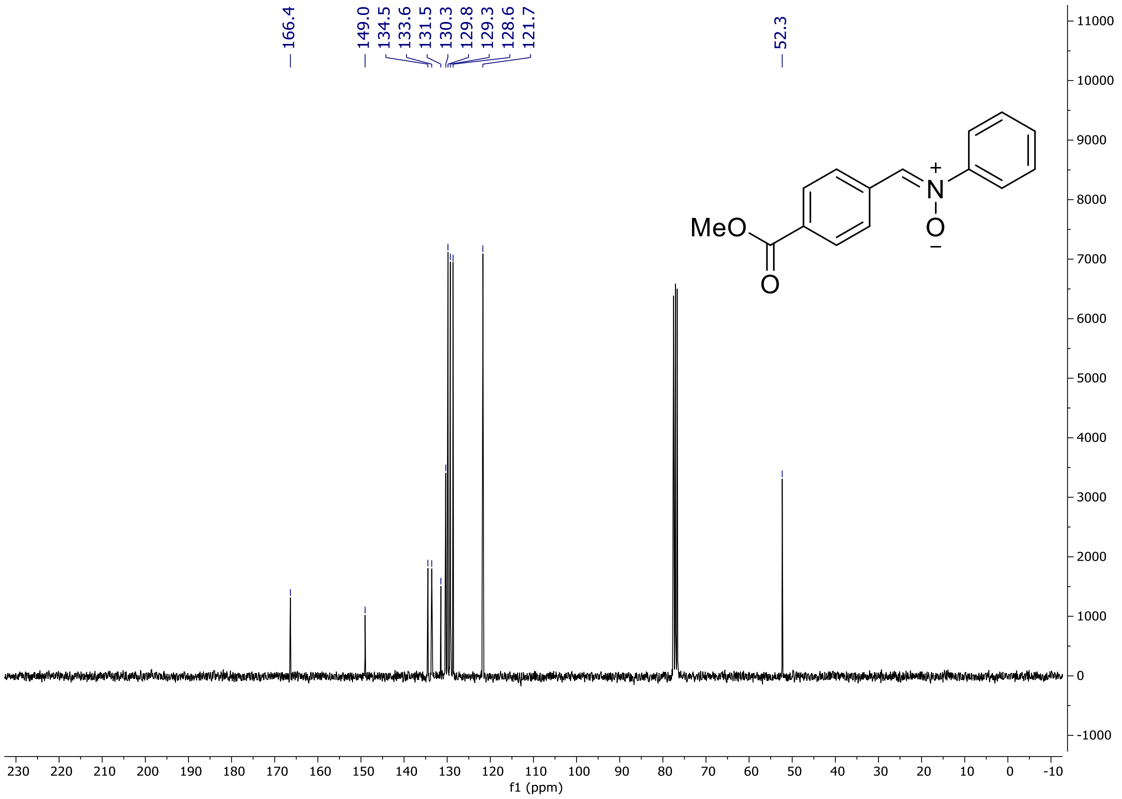

Figure SI_56: ^{13}C-NMR for **1o** in $CDCl_3$ (75 MHz).

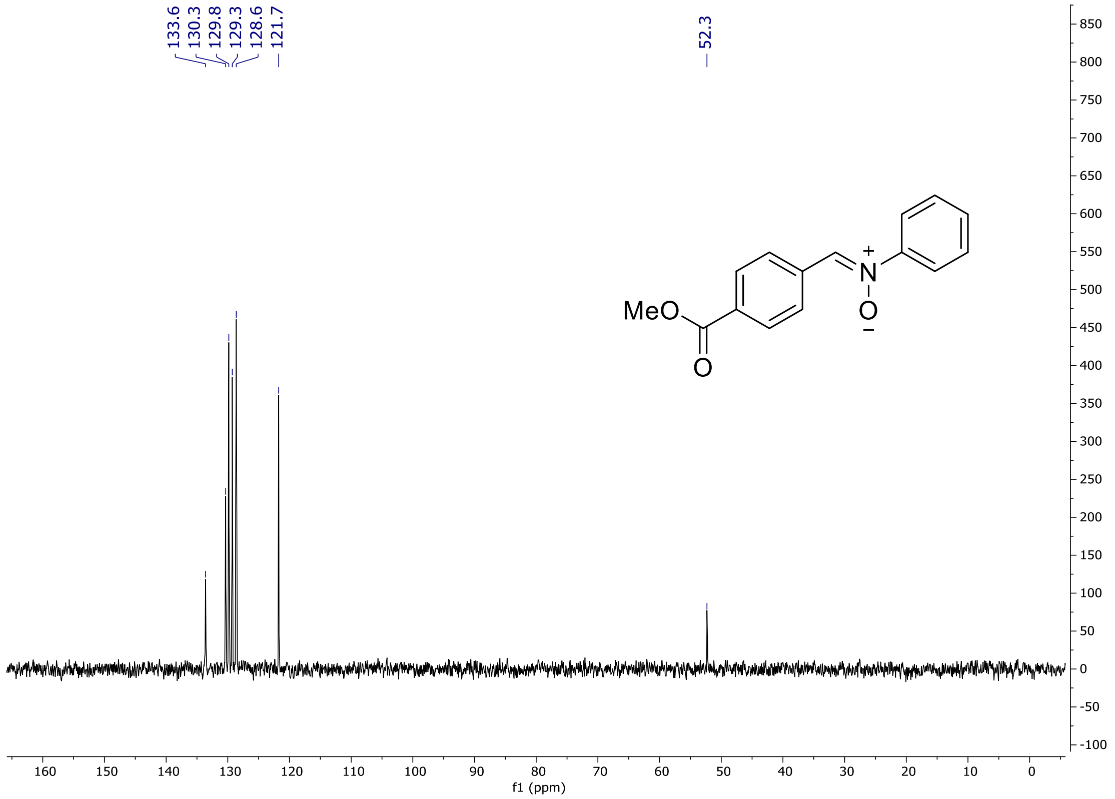

Figure SI_57: DEPT 135-NMR for **1o** in $CDCl_3$ (75 MHz).

***N*-[4-(Isopropylcarbamoyl)benzylidene]aniline oxide (1p)**

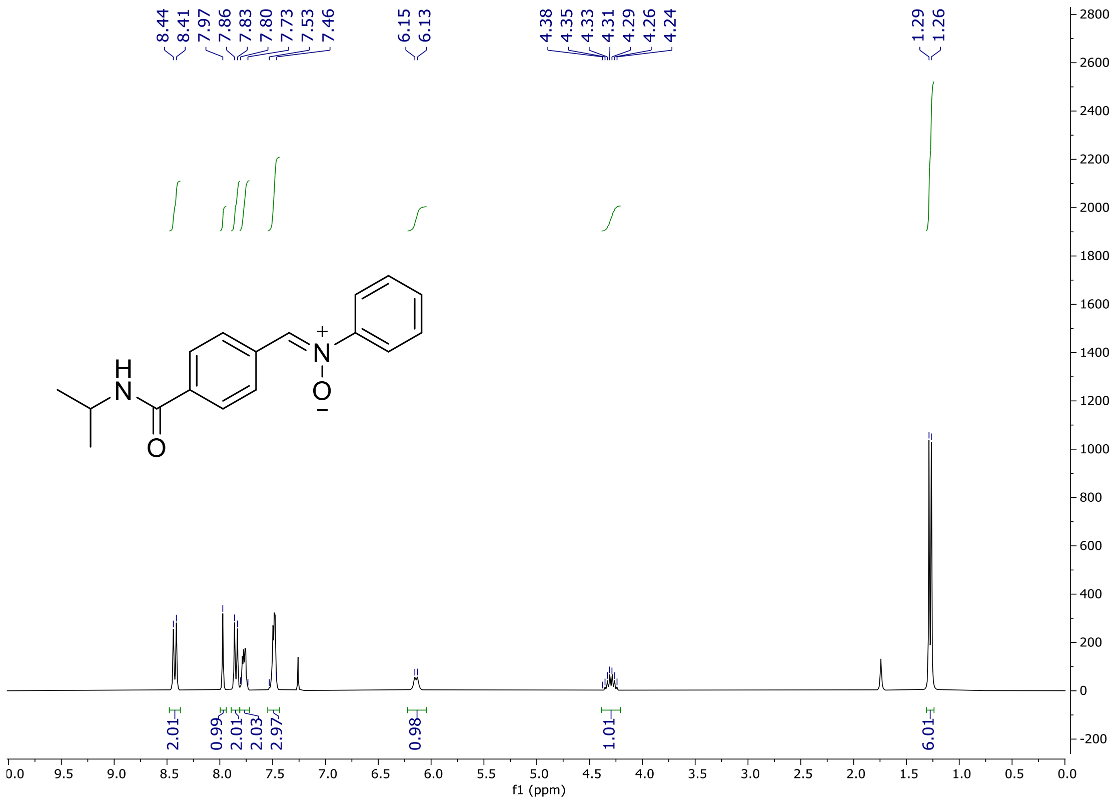

Figure SI_58: ^{1}H-NMR for **1p** in $CDCl_3$ (300 MHz).

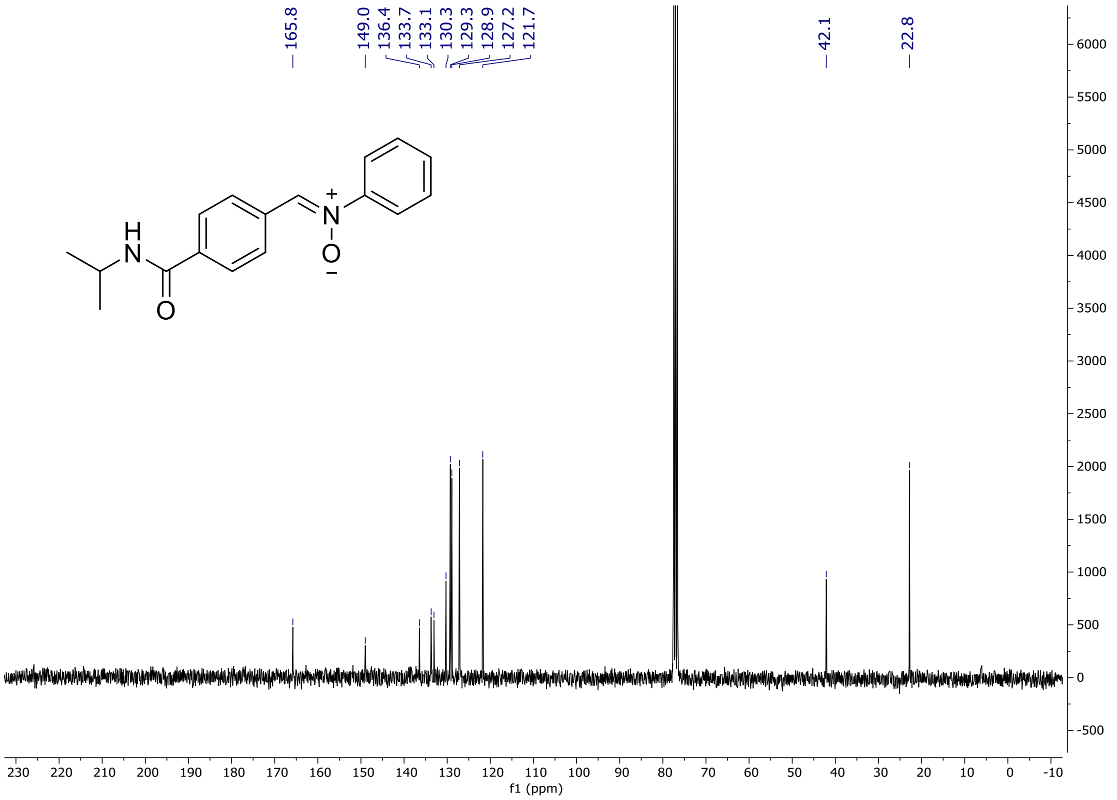

Figure SI_59: ^{13}C-NMR for **1p** in $CDCl_3$ (75 MHz).

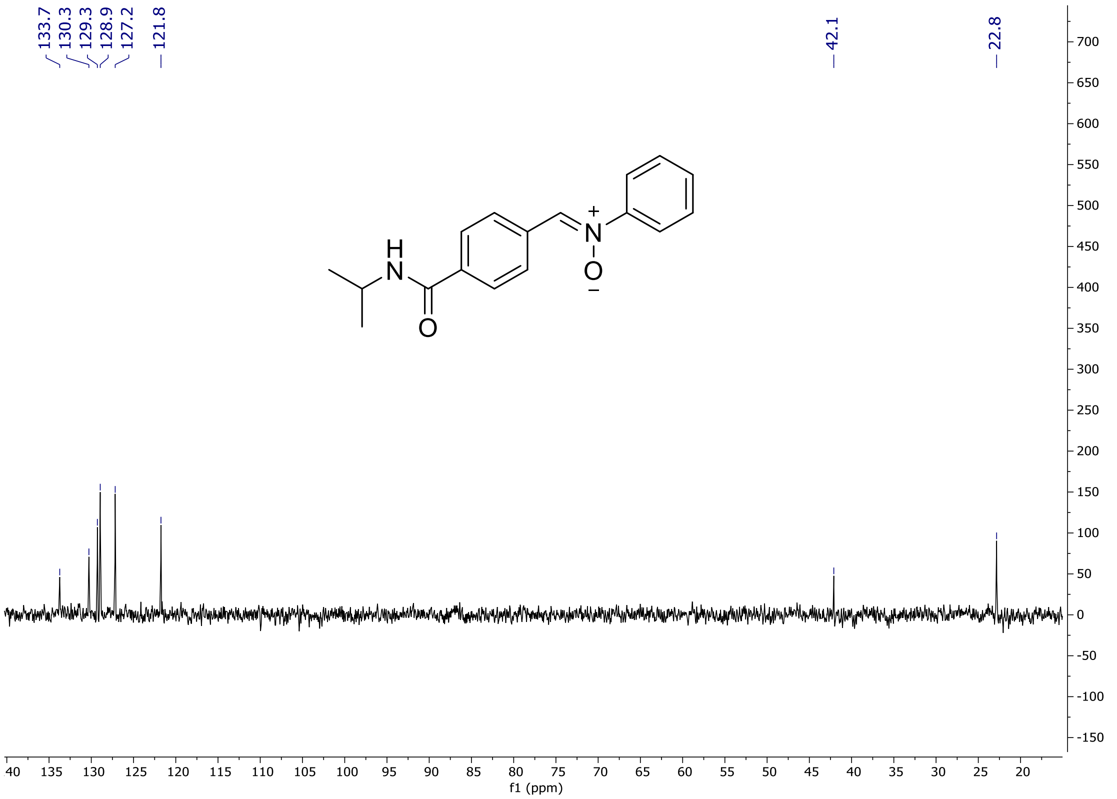

Figure SI_60: DEPT 135-NMR for **1p** in $CDCl_3$ (75 MHz).

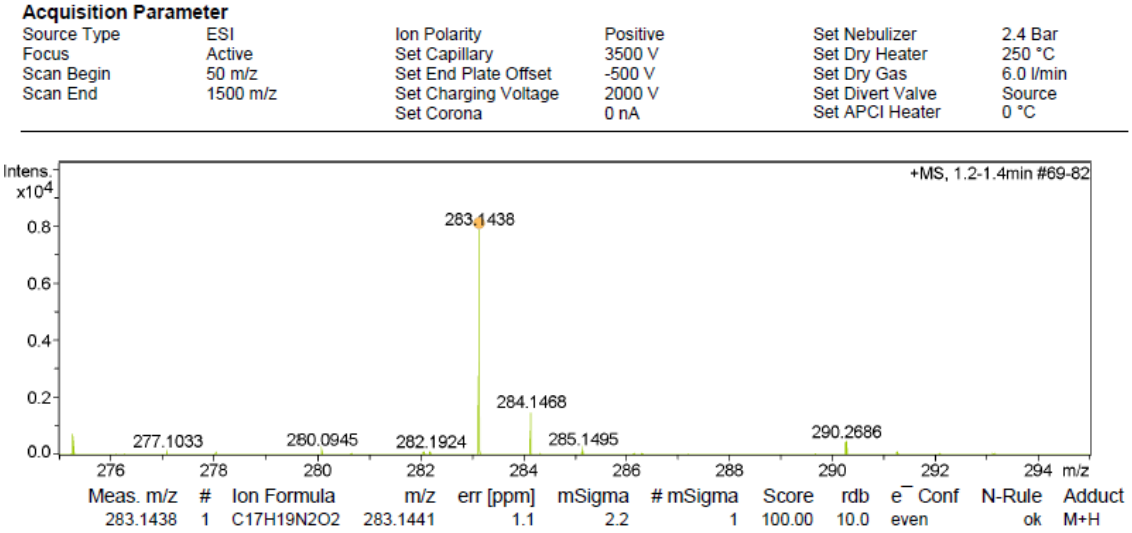

Figure SI_61: HRMS (ESI$^+$, m/z) analysis of **1p**.

N-[4-(Pyrrolidine-1-carbonyl)benzylidene]aniline oxide (1q)

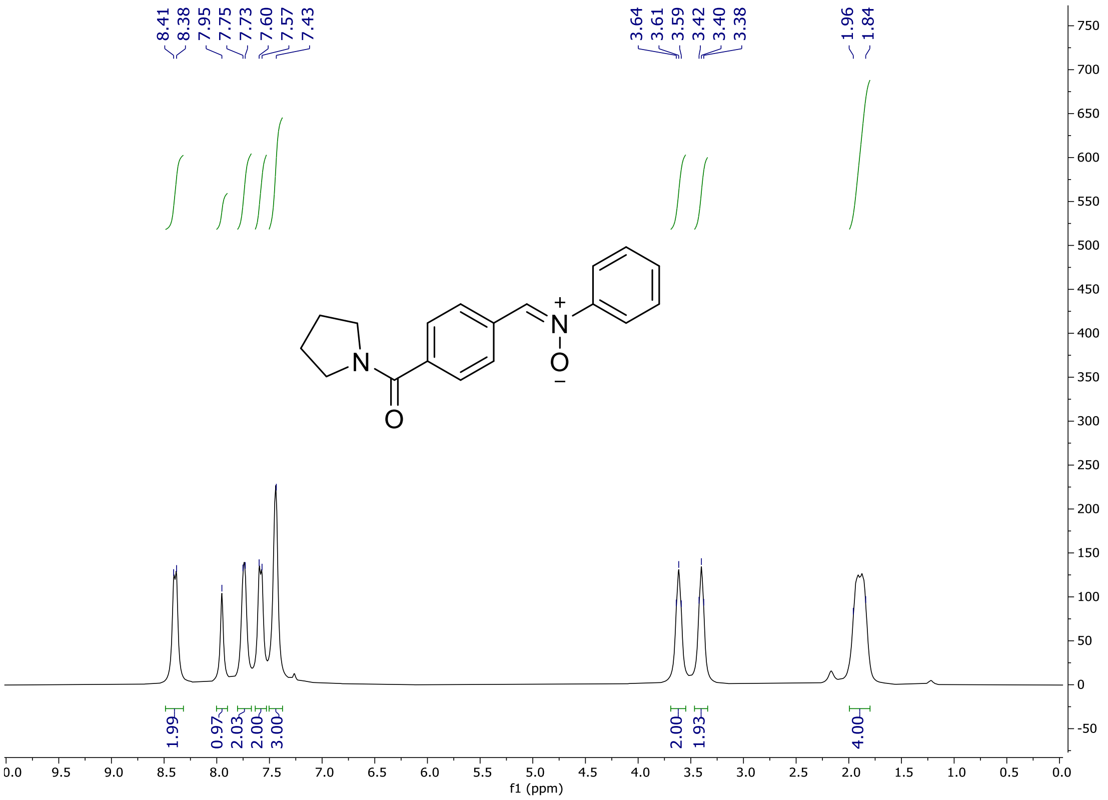

Figure SI_62: ^{1}H-NMR for **1q** in $CDCl_3$ (300 MHz).

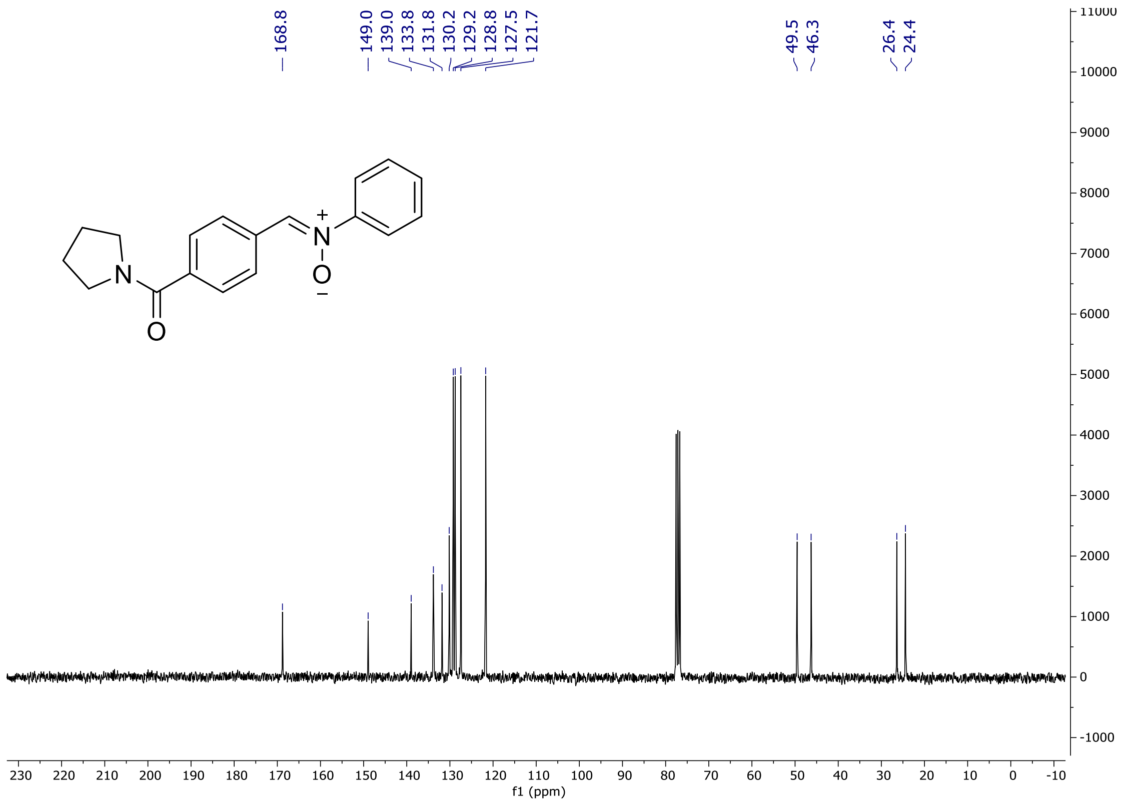

Figure SI_63: ^{13}C-NMR for **1q** in $CDCl_3$ (75 MHz).

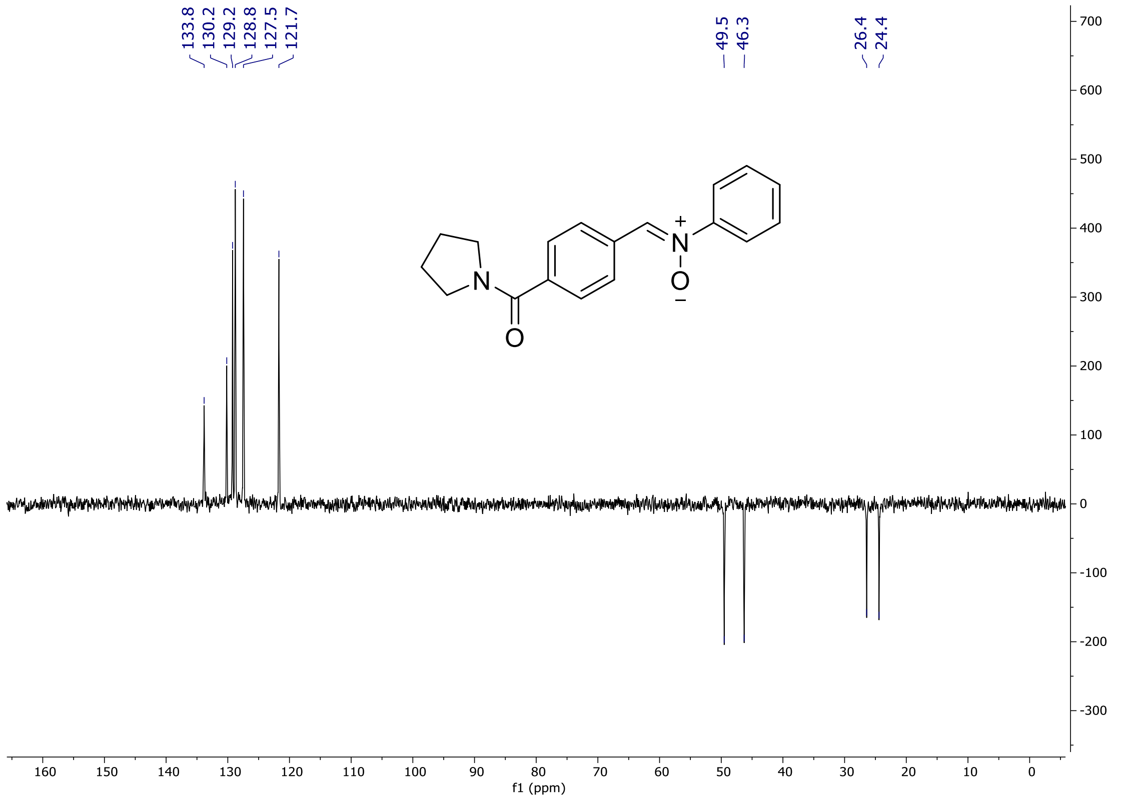

Figure SI_64: DEPT 135-NMR for **1q** in $CDCl_3$ (75 MHz).

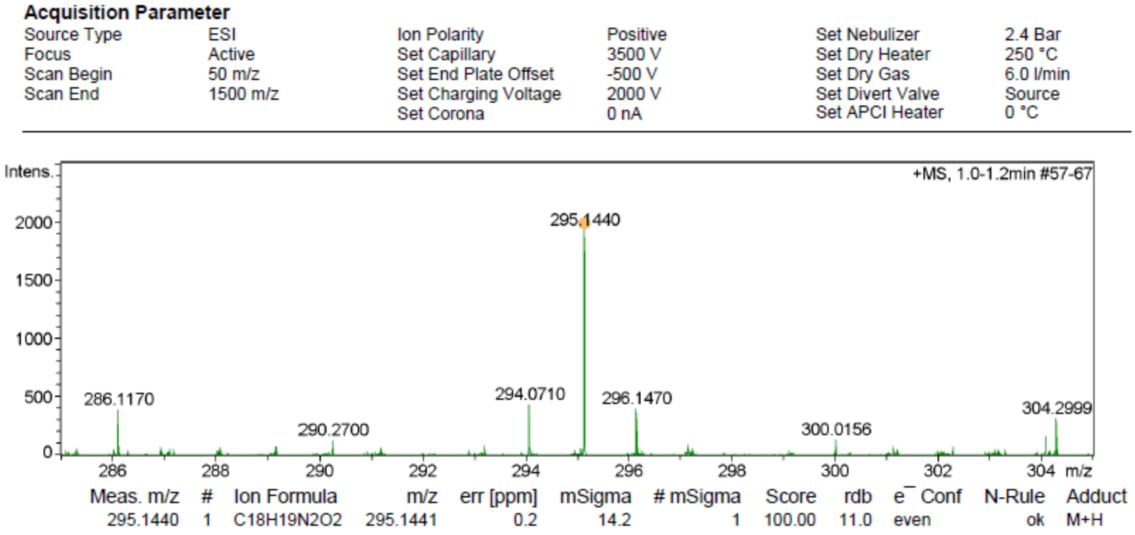

Figure SI_65: HRMS (ESI$^+$, m/z) analysis of **1q**.

N-(4-Methylbenzylidene)aniline oxide (1r)

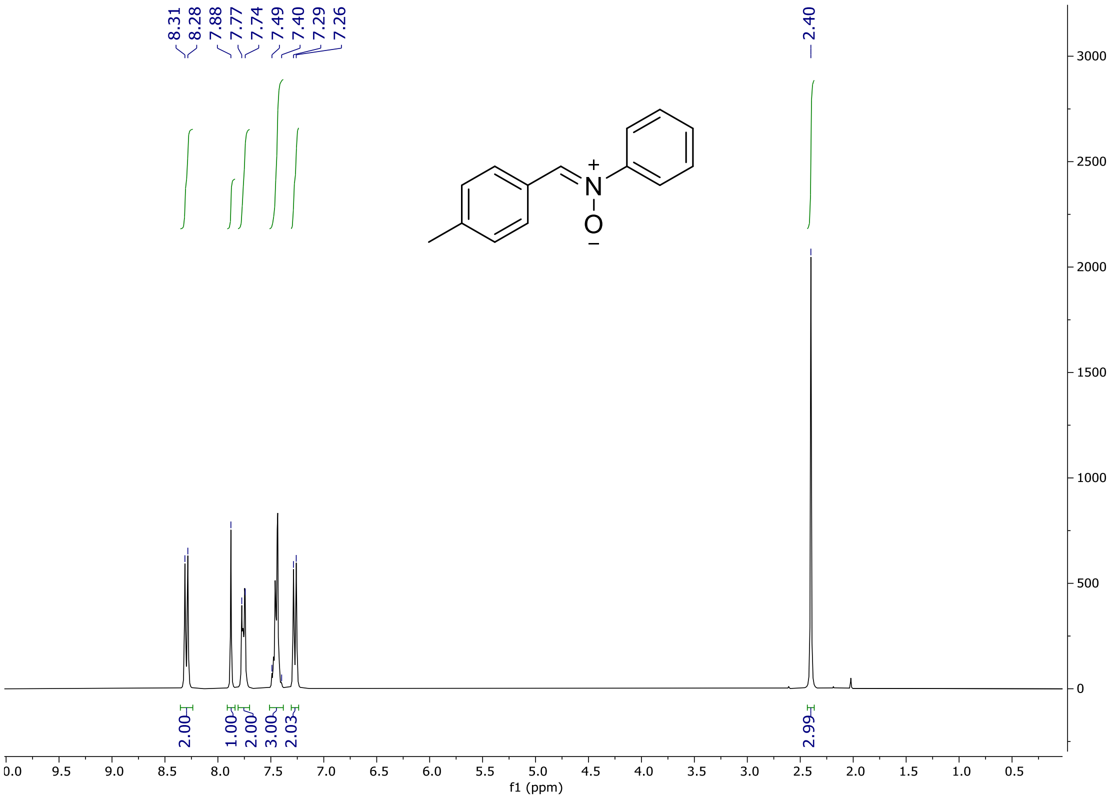

Figure SI_66: ^{1}H-NMR for **1r** in $CDCl_3$ (300 MHz).

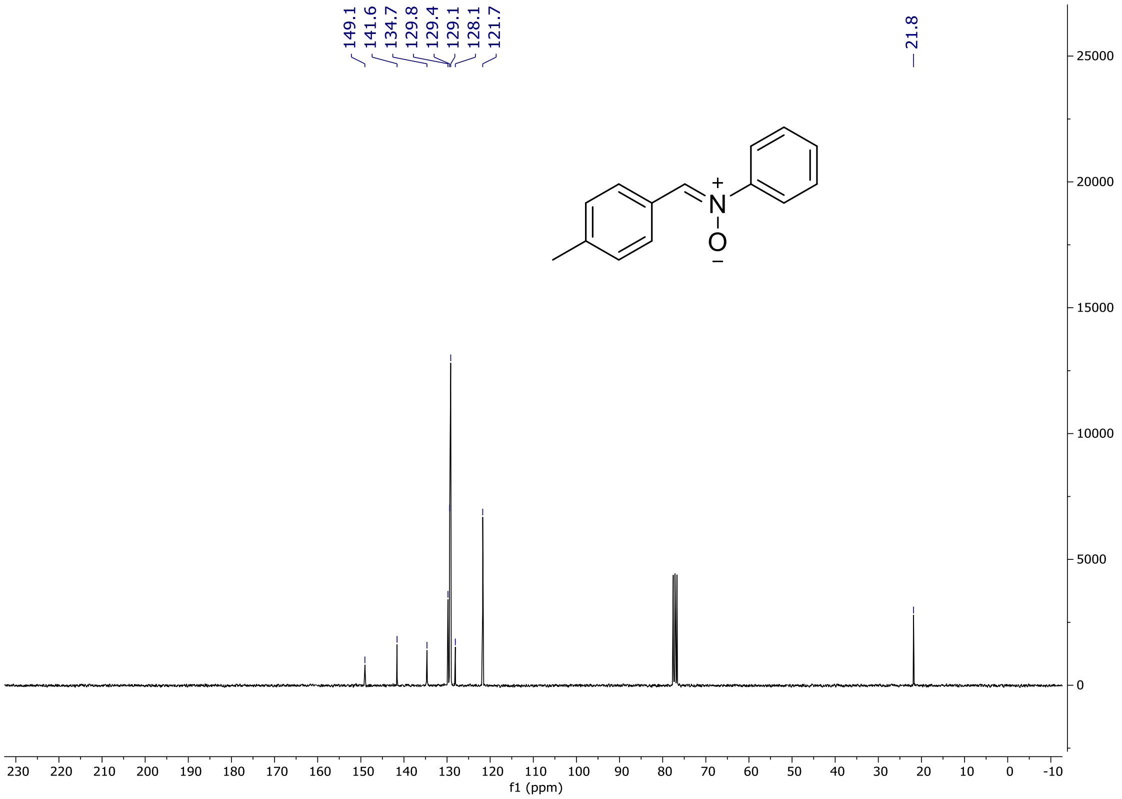

Figure SI_67: ^{13}C-NMR for **1r** in $CDCl_3$ (75 MHz).

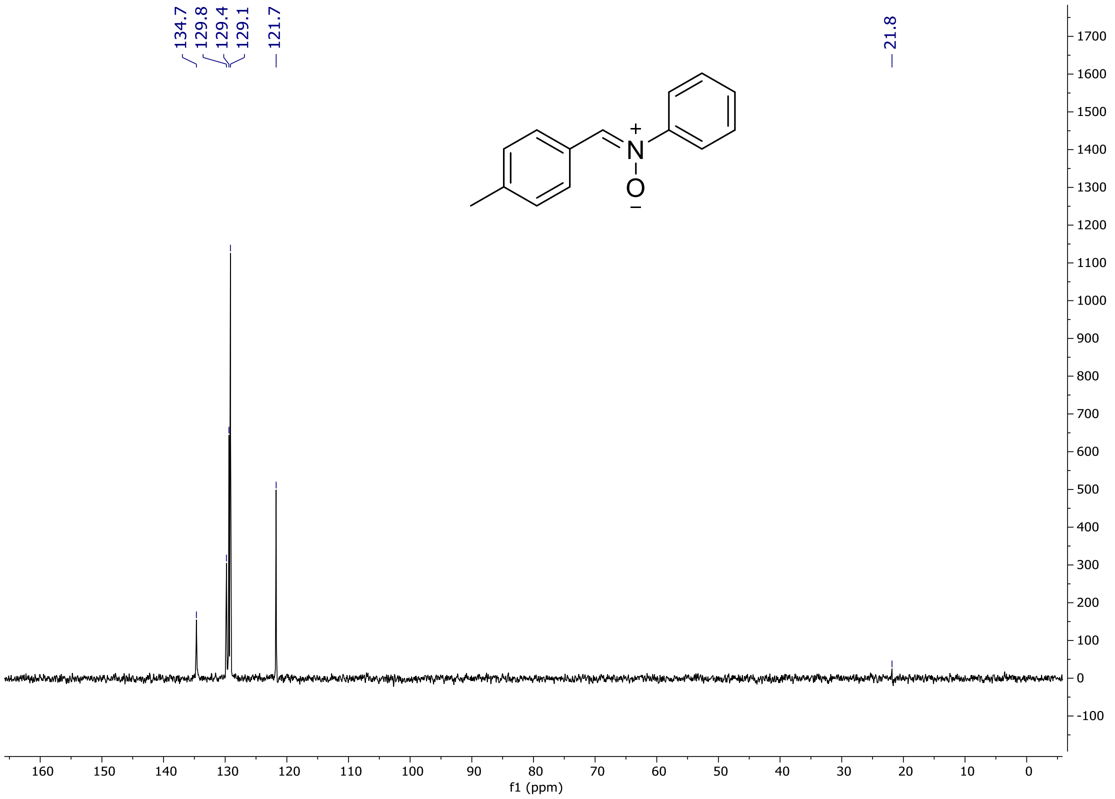

Figure SI_68: DEPT 135-NMR for **1r** in $CDCl_3$ (75 MHz).

***N*-(4-Hydroxybenzylidene)aniline oxide (1s)**

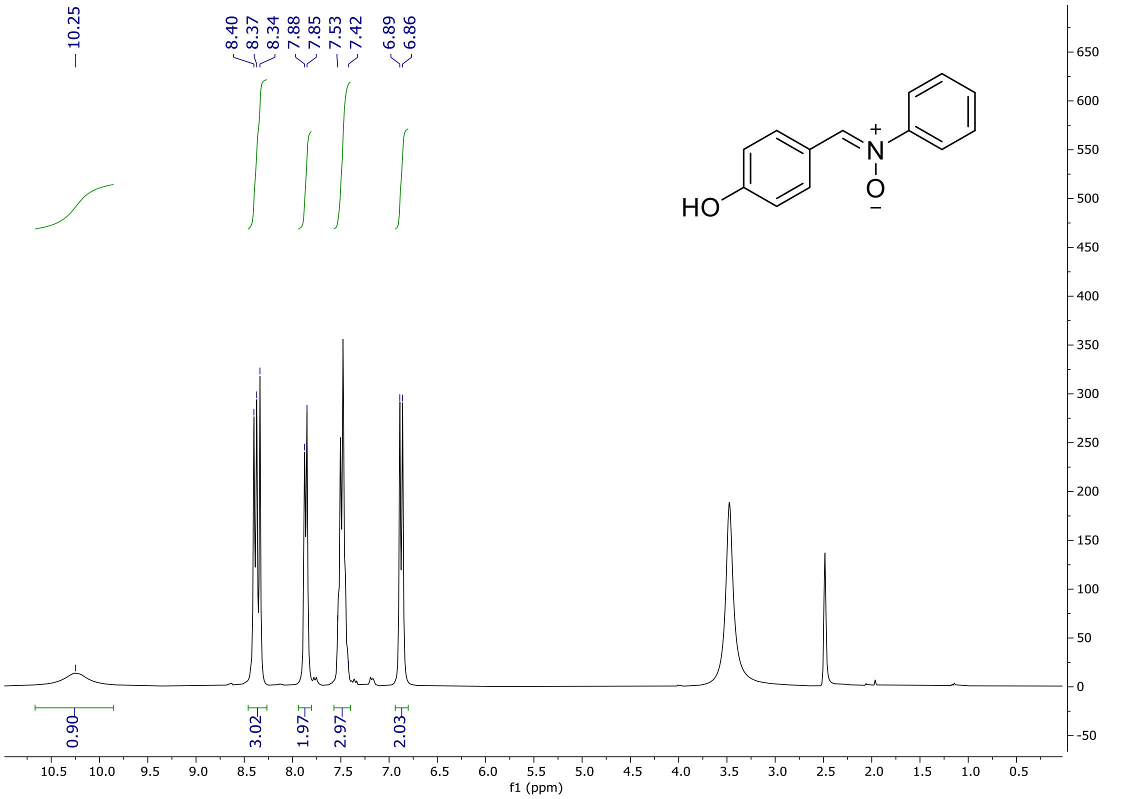

Figure SI_69: ^{1}H-NMR for **1s** in DMSO-*d*^{6} (300 MHz).

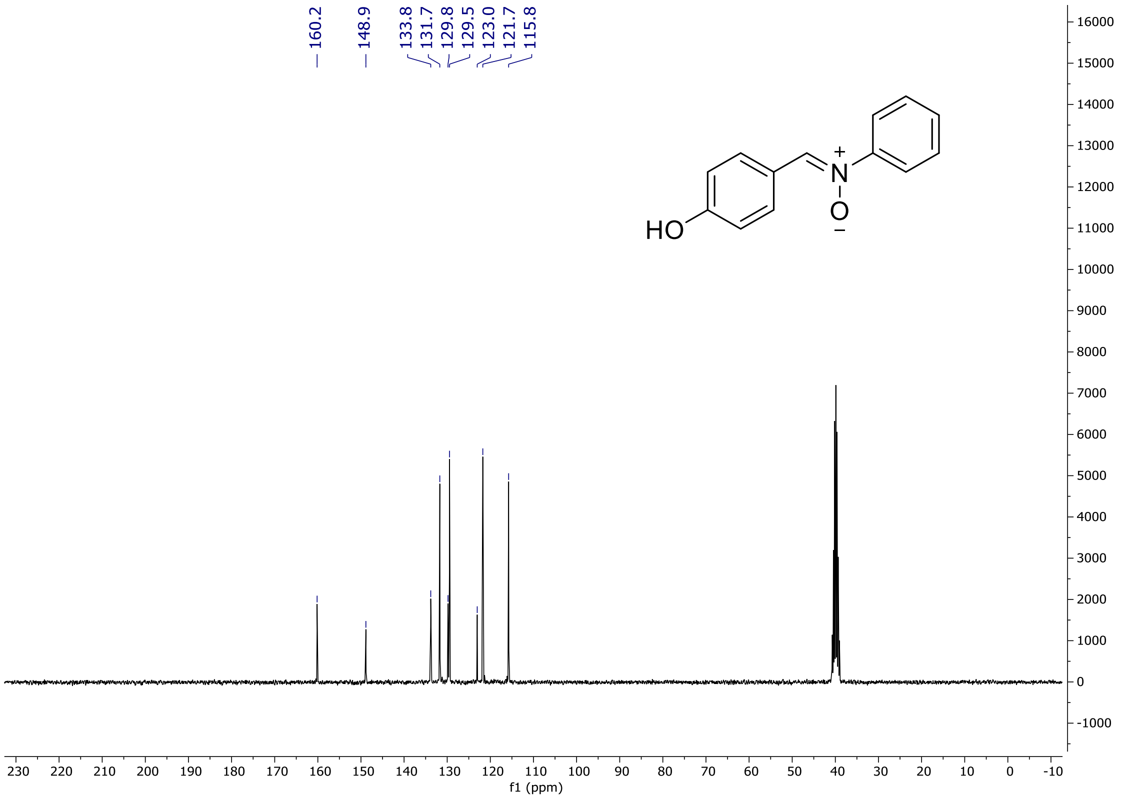

Figure SI_70: ^{13}C-NMR for **1s** in DMSO-*d*^{6} (75 MHz).

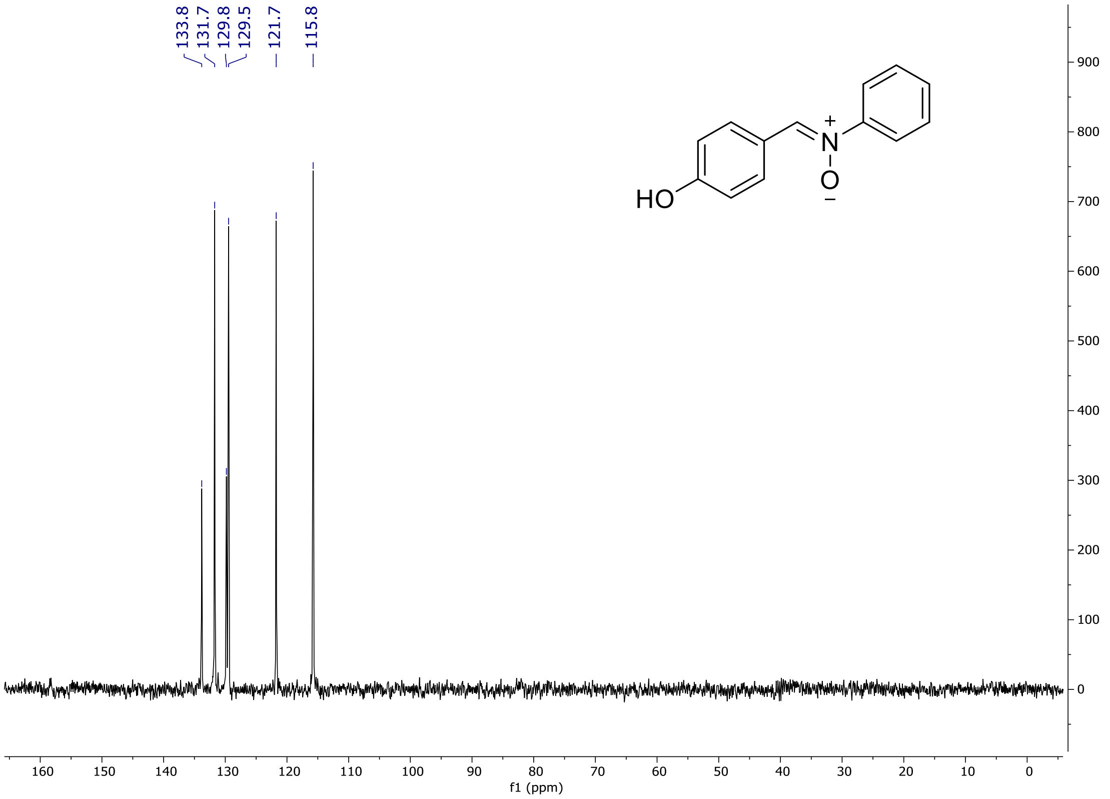

Figure SI_71: DEPT 135-NMR for **1s** in DMSO-d^6 (75 MHz).

Acquisition Parameter

Source Type	ESI	Ion Polarity	Positive	Set Nebulizer	2.4 Bar
Focus	Active	Set Capillary	3500 V	Set Dry Heater	250 °C
Scan Begin	50 m/z	Set End Plate Offset	-500 V	Set Dry Gas	6.0 l/min
Scan End	1500 m/z	Set Charging Voltage	2000 V	Set Divert Valve	Source
		Set Corona	0 nA	Set APCI Heater	0 °C

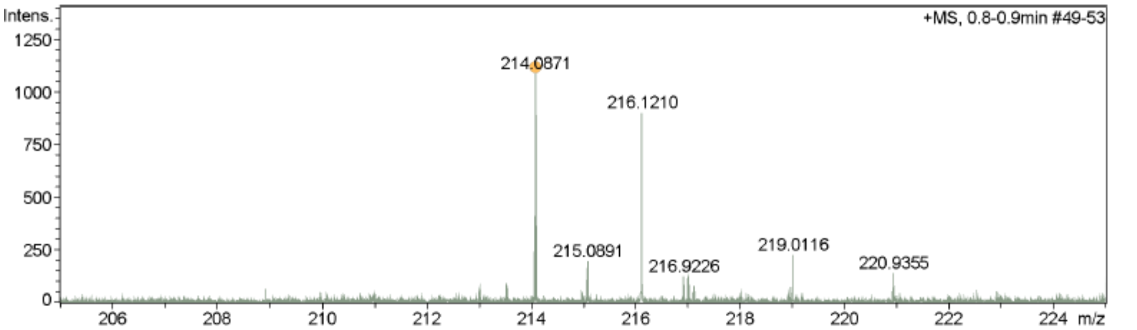

Meas. m/z	#	Ion Formula	m/z	err [ppm]	mSigma	# mSigma	Score	rdb	e⁻ Conf	N-Rule	Adduct
214.0871	1	C13H12NO2	214.0863	-3.8	23.0	1	100.00	9.0	even	ok	M+H

Figure SI_72: HRMS (ESI$^+$, m/z) analysis of **1s**.

N-(4-Methoxybenzylidene)aniline oxide (1t)

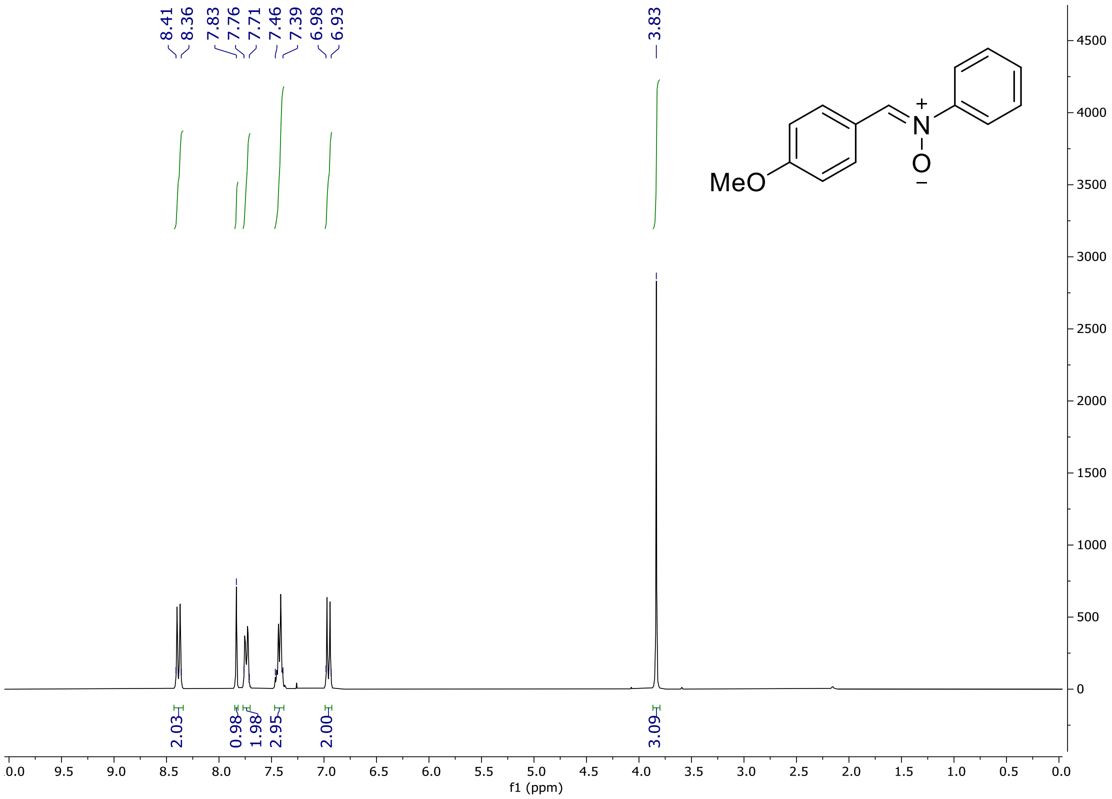

Figure SI_73: ^{1}H-NMR for **1t** in $CDCl_3$ (300 MHz).

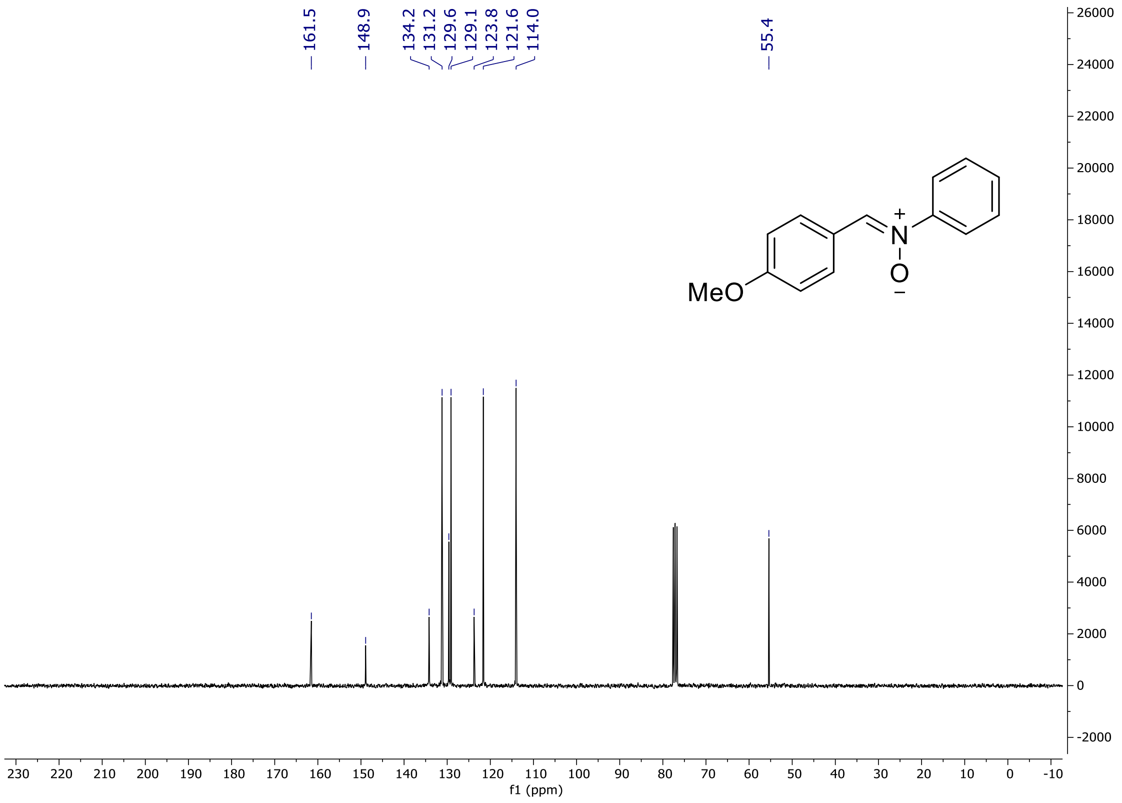

Figure SI_74: ^{13}C-NMR for **1t** in $CDCl_3$ (75 MHz).

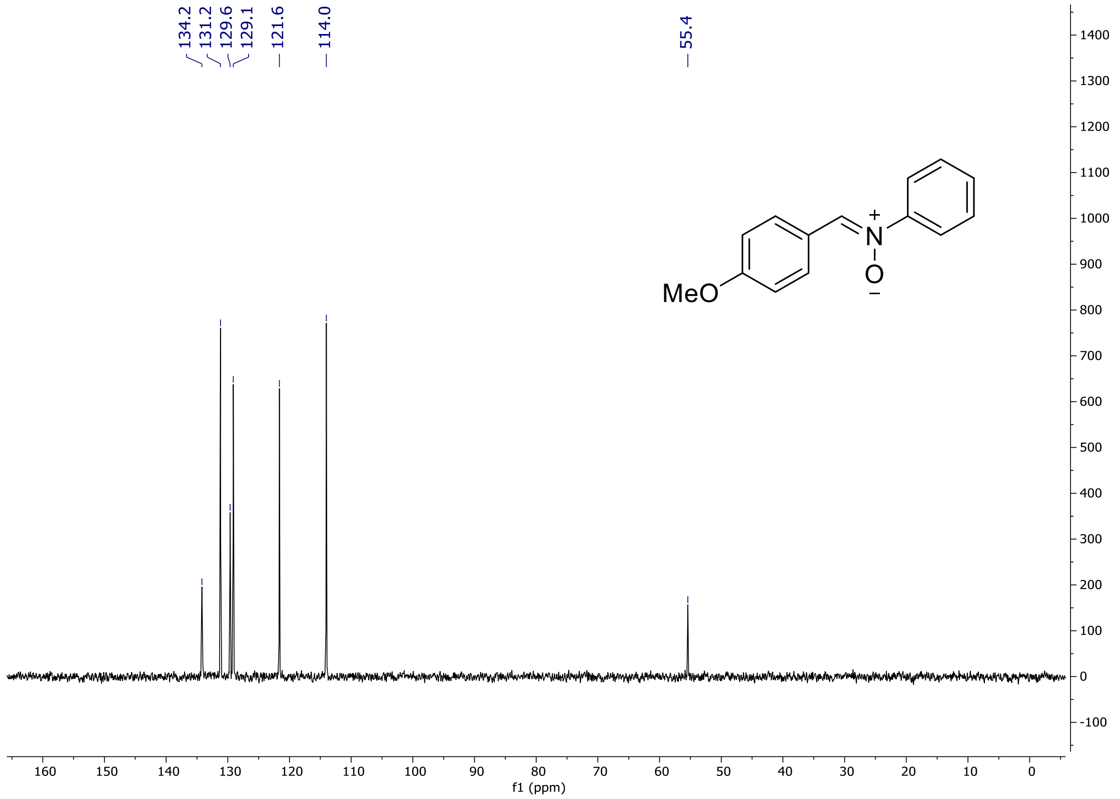

Figure SI_75: DEPT 135-NMR for **1t** in $CDCl_3$ (75 MHz).

***N*-(4-Acetamidobenzylidene)aniline oxide (1u)**

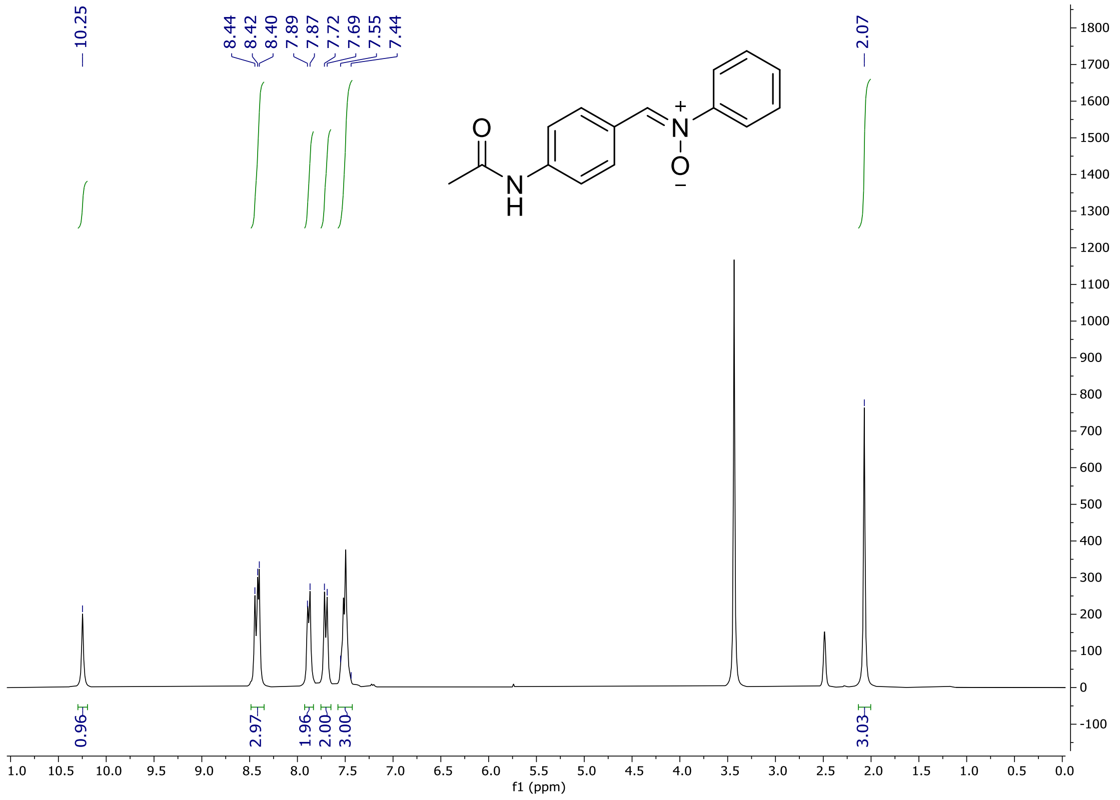

Figure SI_76: ^{1}H-NMR for **1u** in DMSO-d^6 (300 MHz).

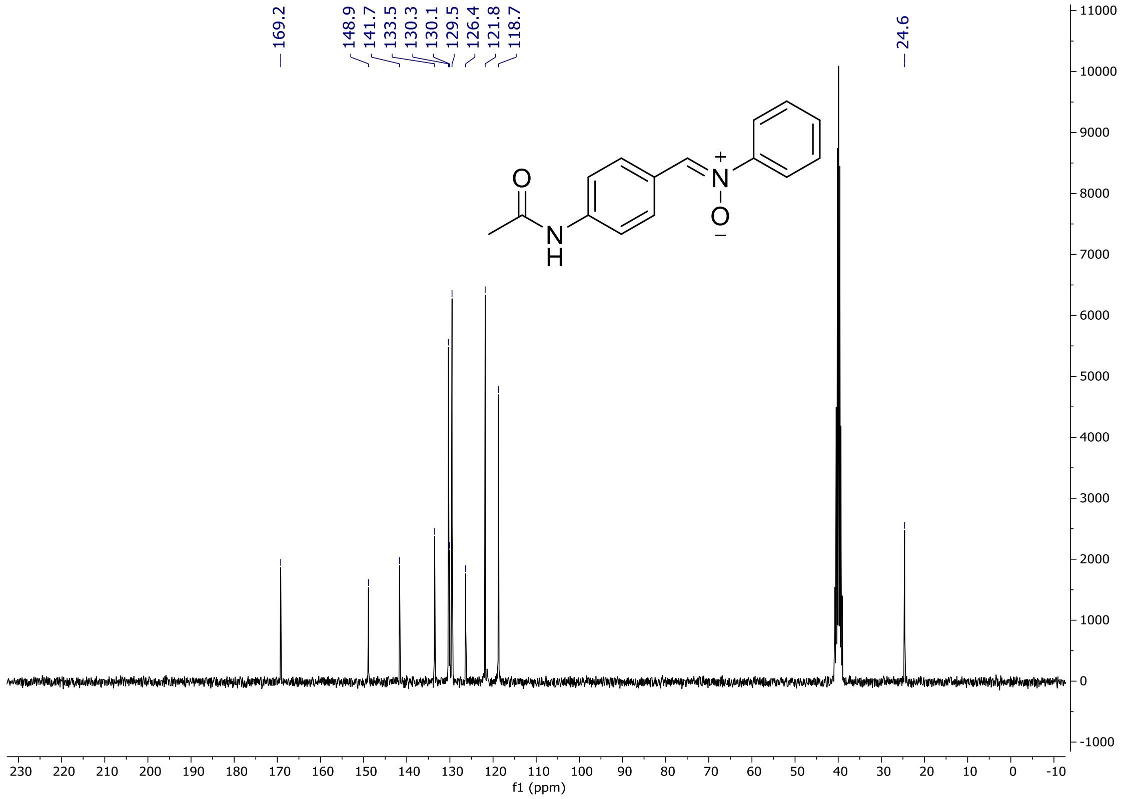

Figure SI_77: ^{13}C-NMR for **1u** in DMSO-d^6 (75 MHz).

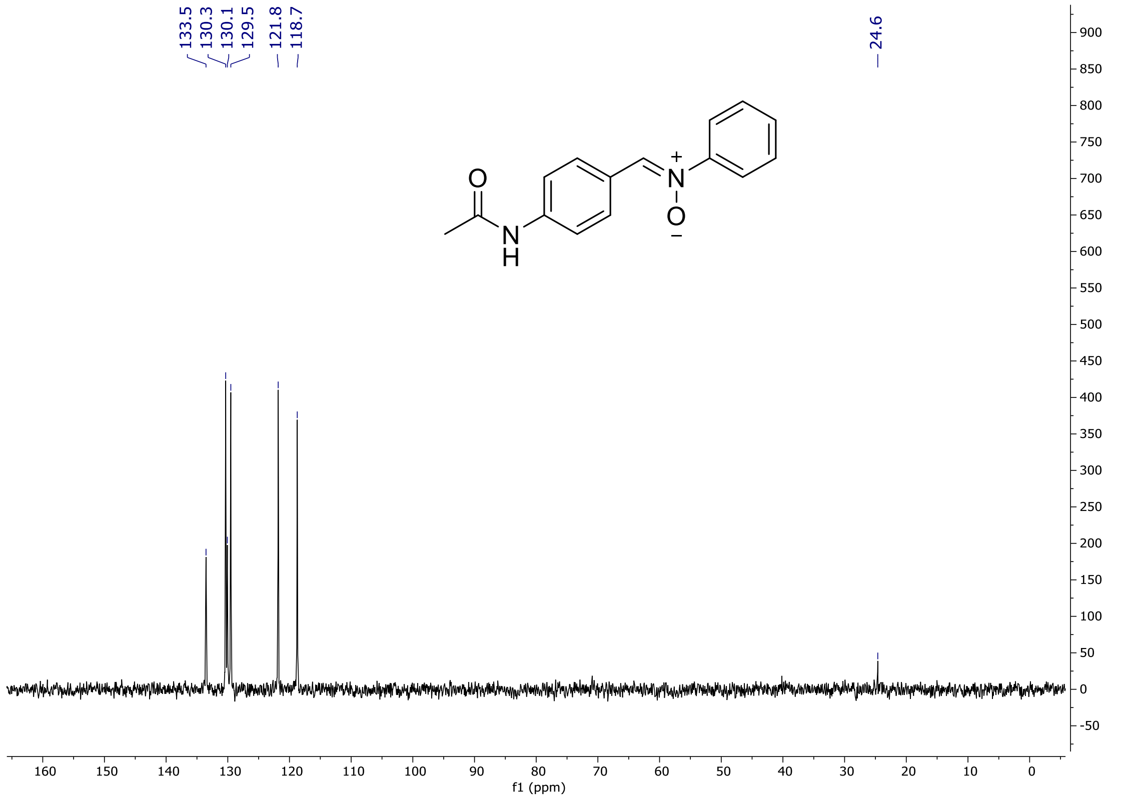

Figure SI_78: DEPT 135-NMR for **1u** in DMSO-d^6 (75 MHz).

Acquisition Parameter

Source Type	ESI	Ion Polarity	Positive	Set Nebulizer	2.4 Bar
Focus	Active	Set Capillary	3500 V	Set Dry Heater	250 °C
Scan Begin	50 m/z	Set End Plate Offset	-500 V	Set Dry Gas	6.0 l/min
Scan End	1500 m/z	Set Charging Voltage	2000 V	Set Divert Valve	Source
		Set Corona	0 nA	Set APCI Heater	0 °C

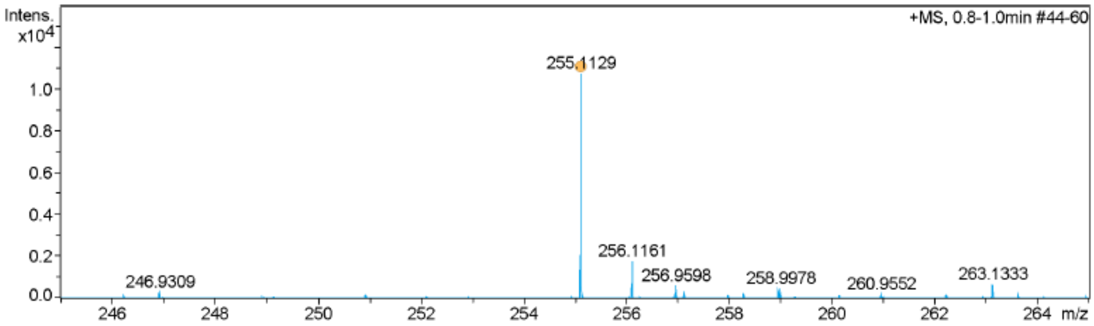

Meas. m/z	#	Ion Formula	m/z	err [ppm]	mSigma	# mSigma	Score	rdb	e⁻ Conf	N-Rule	Adduct
255.1129	1	C15H15N2O2	255.1128	-0.3	2.5	1	100.00	10.0	even	ok	M+H

Figure SI_79: HRMS (ESI^+, m/z) analysis of **1u**.

***N*-(Furan-2-ylmethylidene)aniline oxide (1v)**

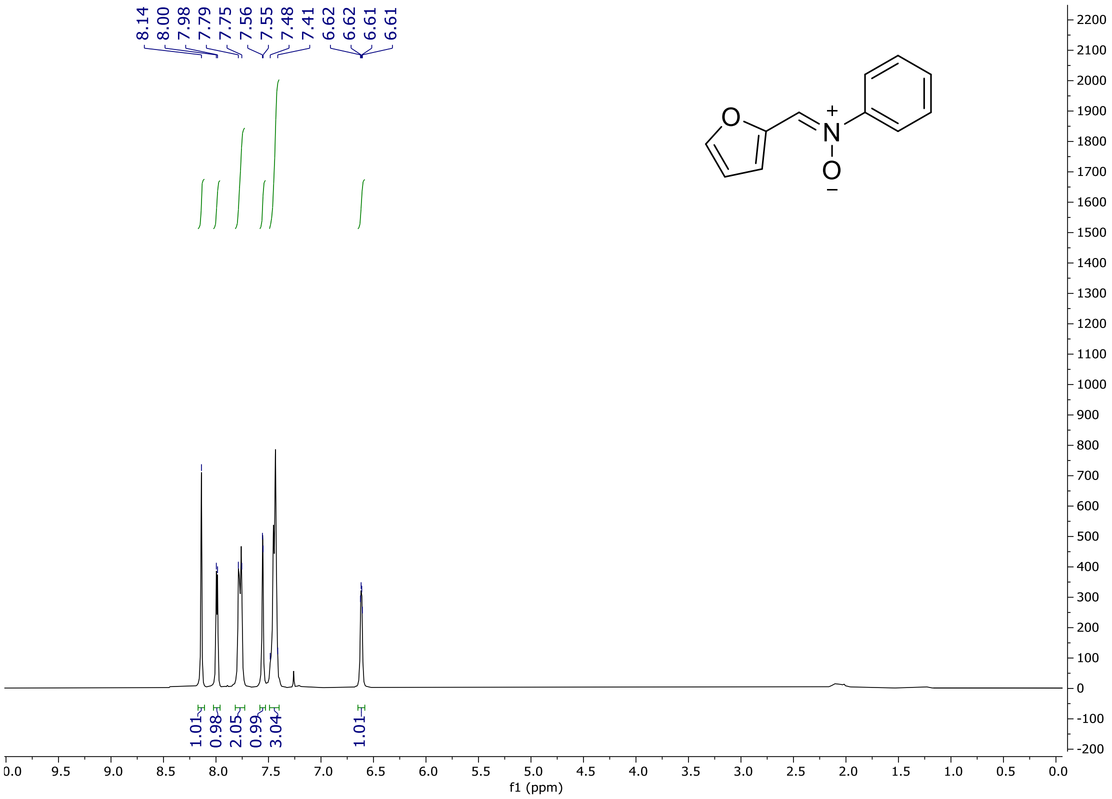

Figure SI_80: ^{1}H-NMR for **1v** in $CDCl_3$ (300 MHz).

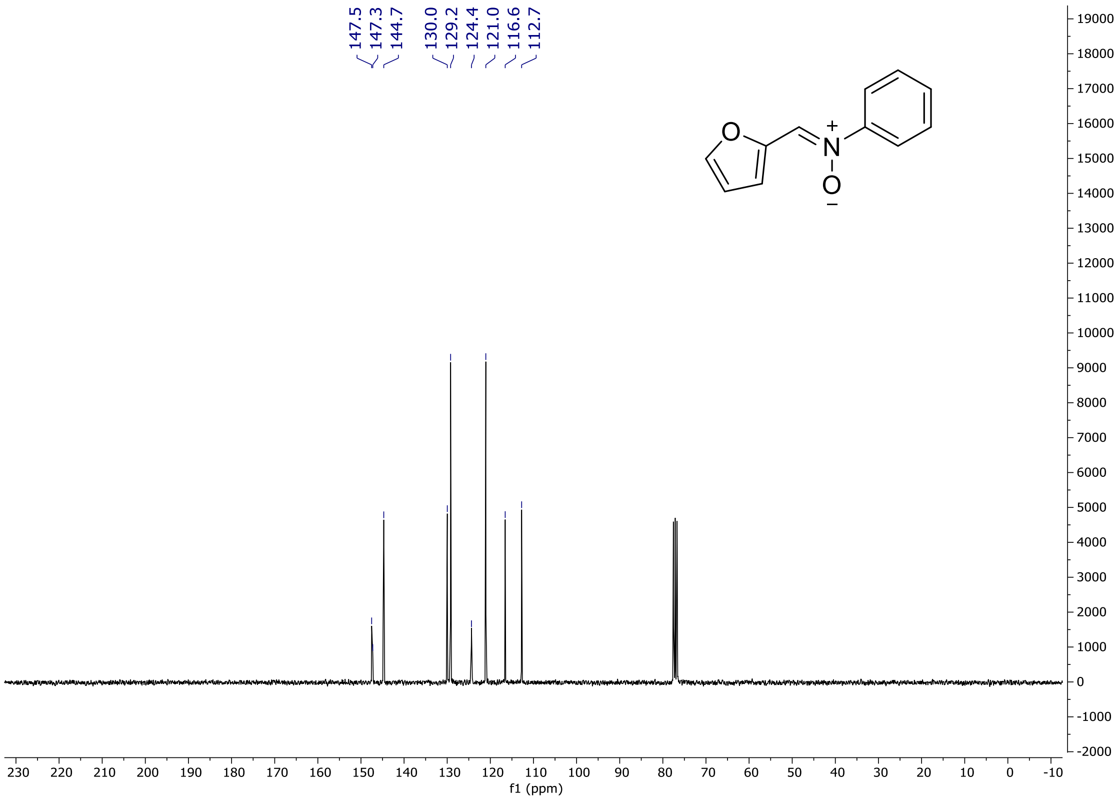

Figure SI_81: ^{13}C-NMR for **1v** in $CDCl_3$ (75 MHz).

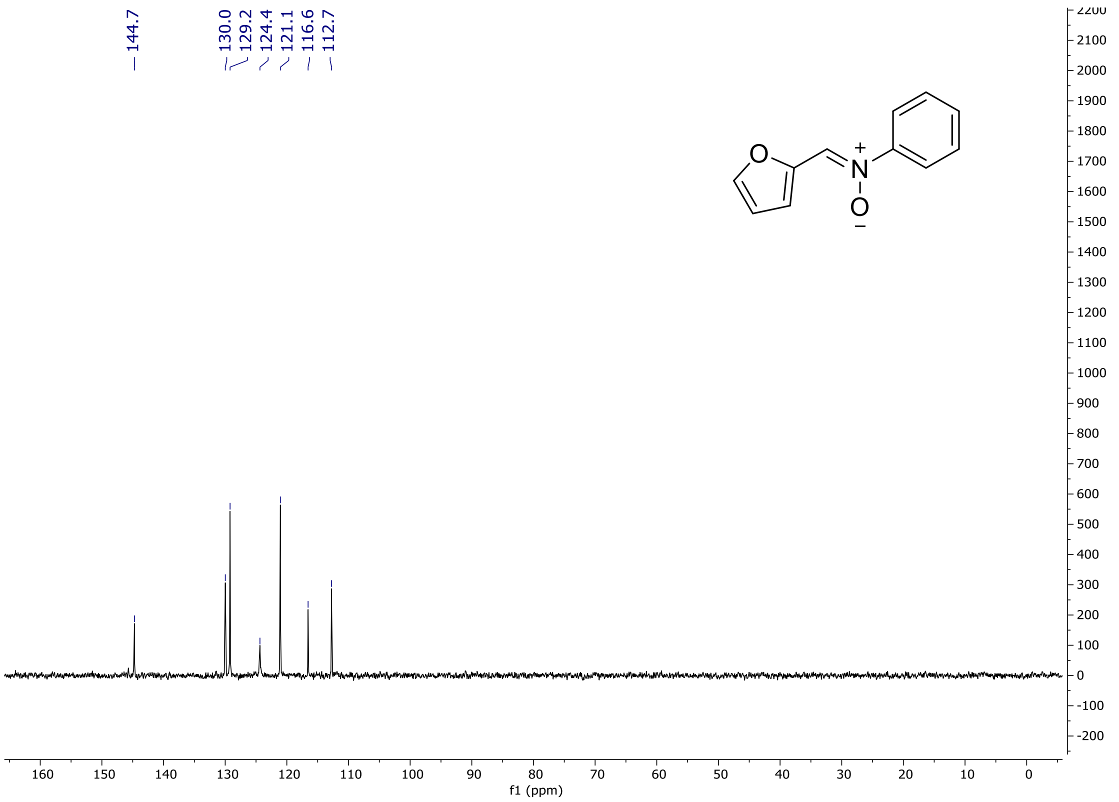

Figure SI_82: DEPT 135-NMR for **1v** in in $CDCl_3$ (75 MHz).

N-(Thiophen-2-ylmethylidene)aniline oxide (1w)

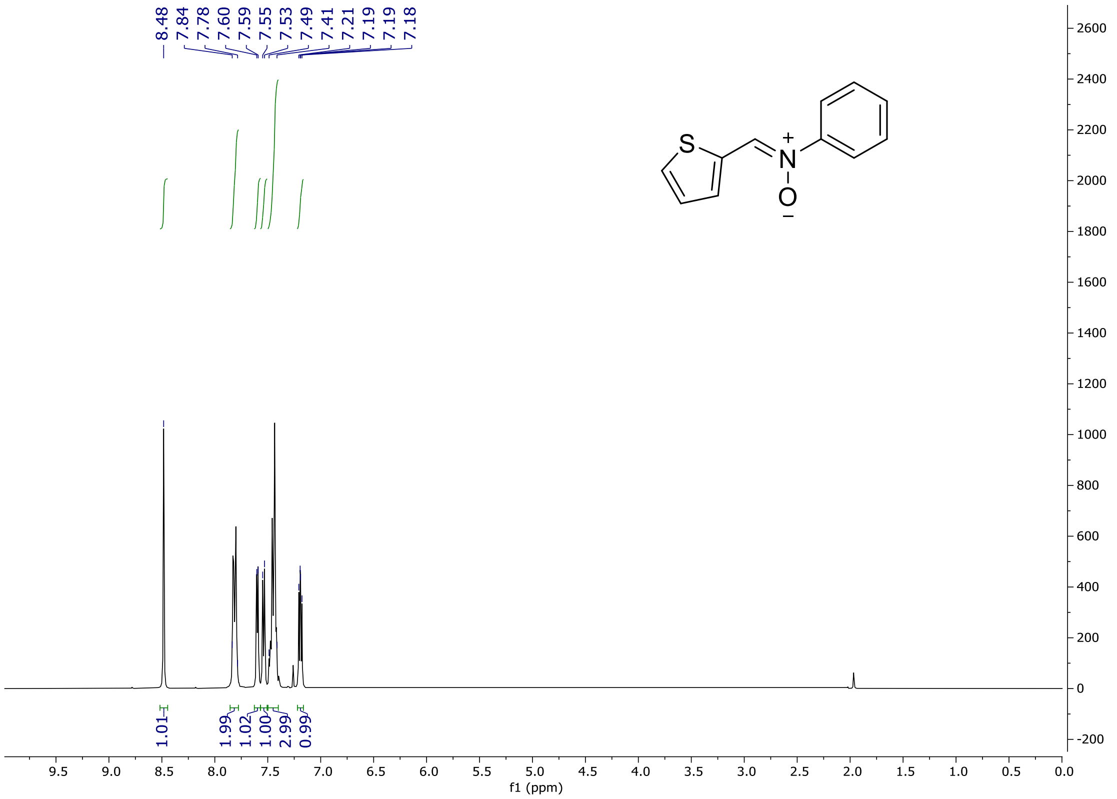

Figure SI_83: ^{1}H-NMR for **1w** in $CDCl_3$ (300 MHz).

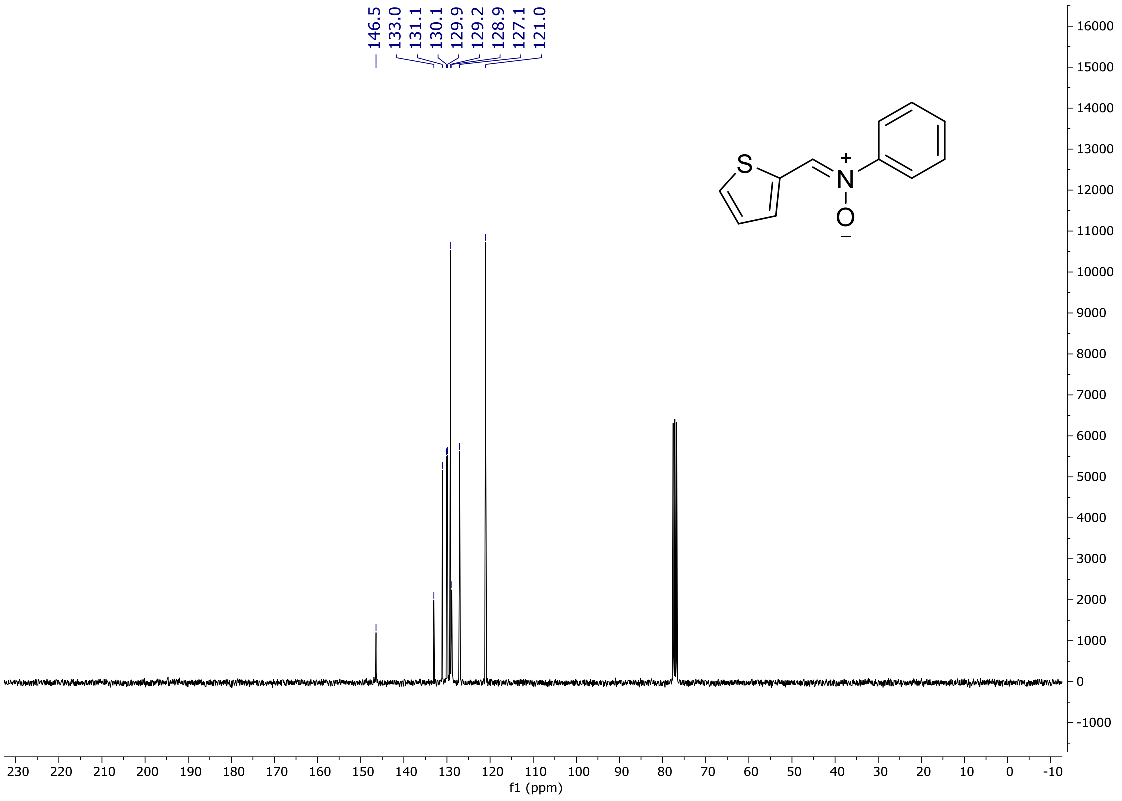

Figure SI_84: ^{13}C-NMR for **1w** in $CDCl_3$ (75 MHz).

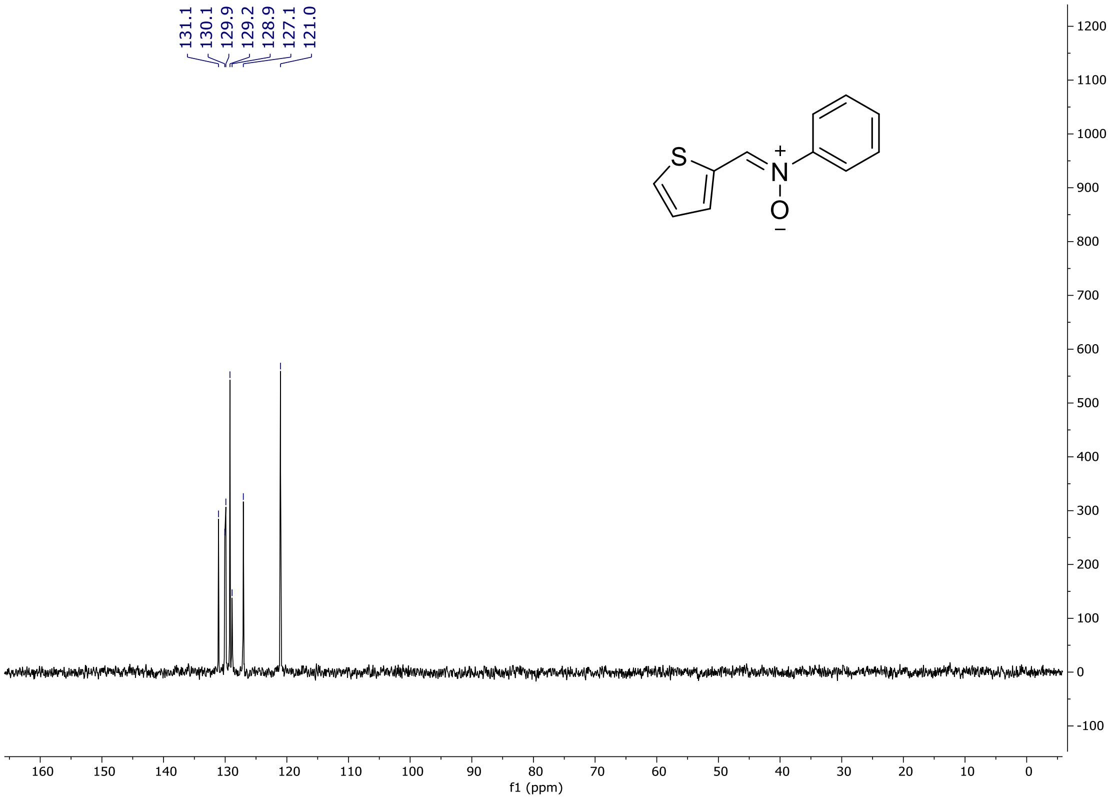

Figure SI_85: DEPT 135-NMR for **1w** in $CDCl_3$ (75 MHz).

***N*-(Pyridin-3-ylmethylidene)aniline oxide (1x)**

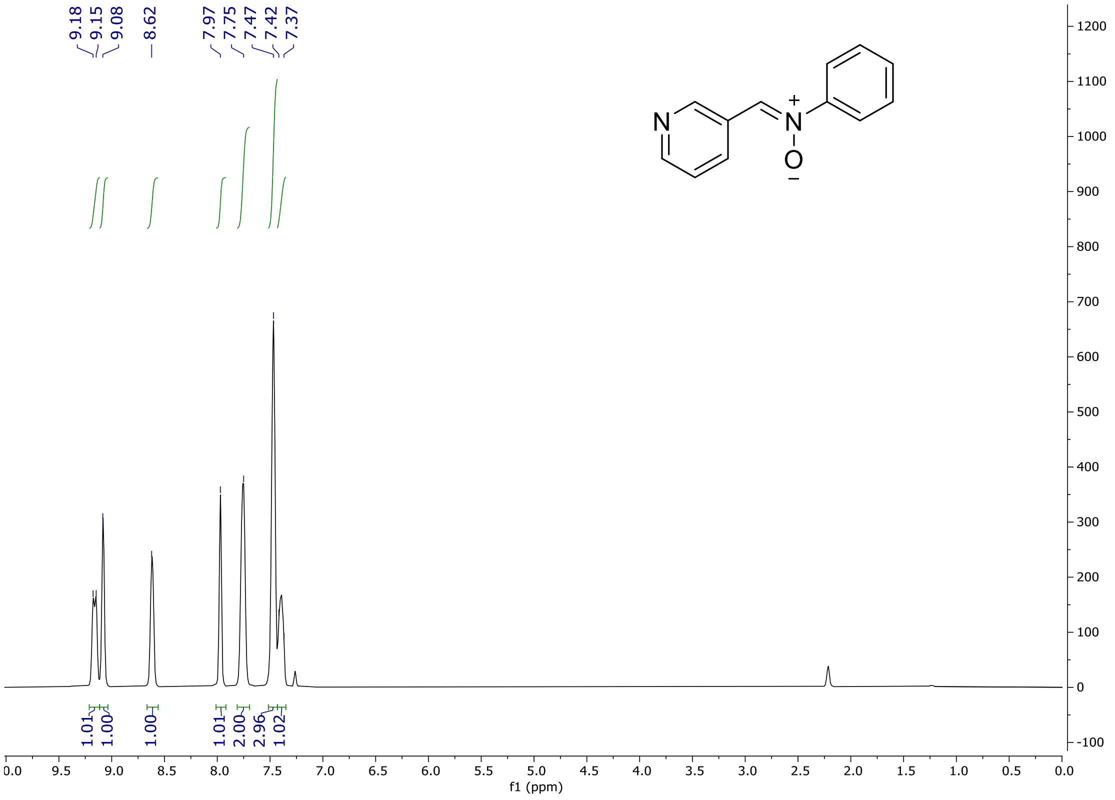

Figure SI_86: ^{1}H-NMR for **1x** in $CDCl_3$ (300 MHz).

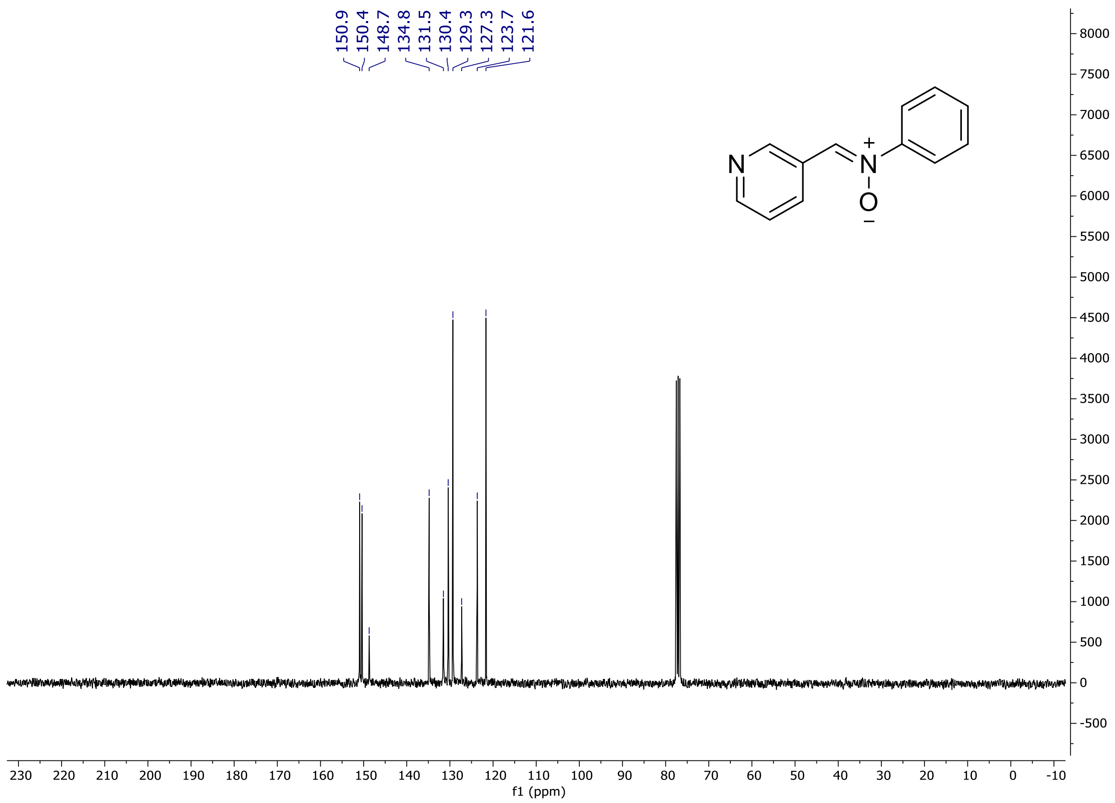

Figure SI_87: ^{13}C-NMR for **1x** in $CDCl_3$ (75 MHz).

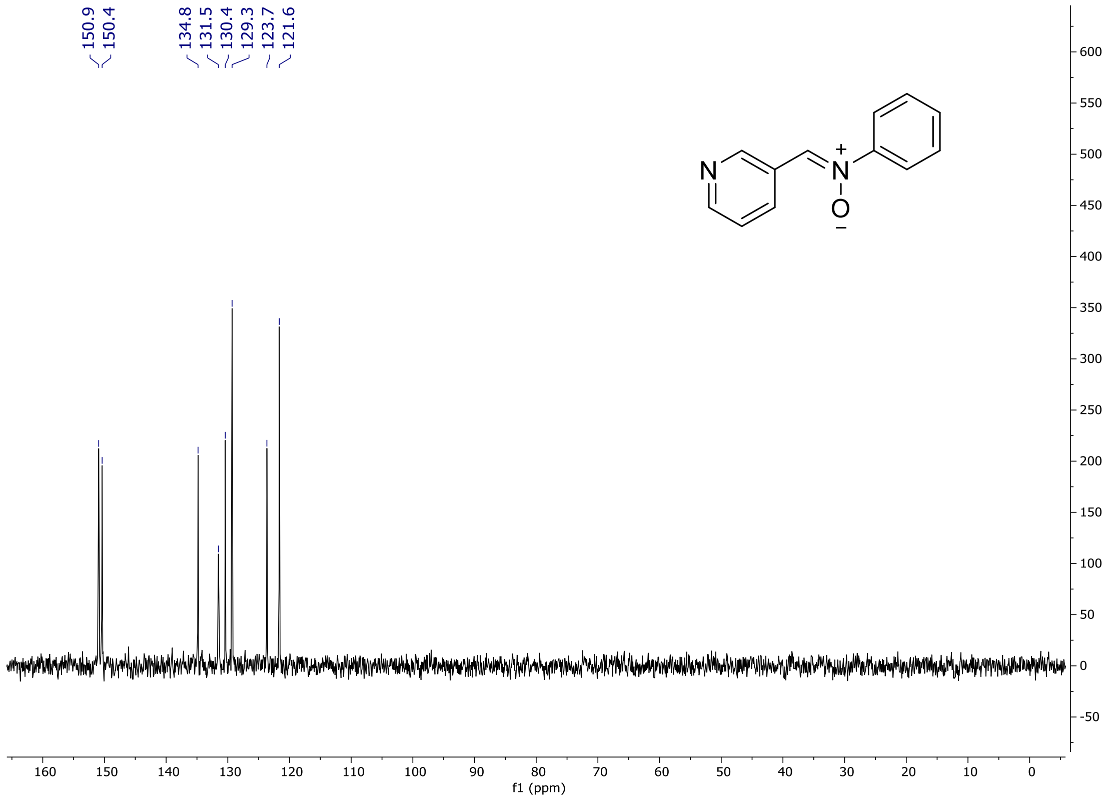

Figure SI_88: DEPT 135-NMR for **1x** in $CDCl_3$ (75 MHz).

N-(Naphthalen-1-ylmethylidene)aniline oxide (1y)

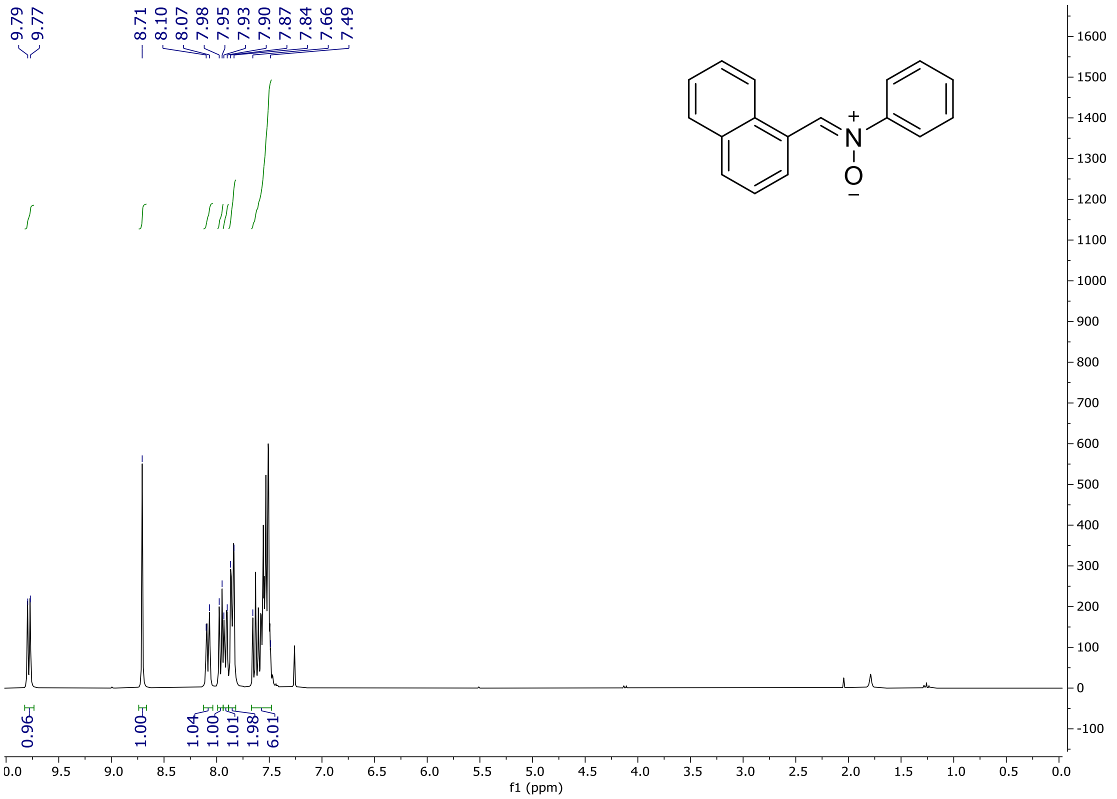

Figure SI_89: ^{1}H-NMR for **1y** in $CDCl_3$ (300 MHz).

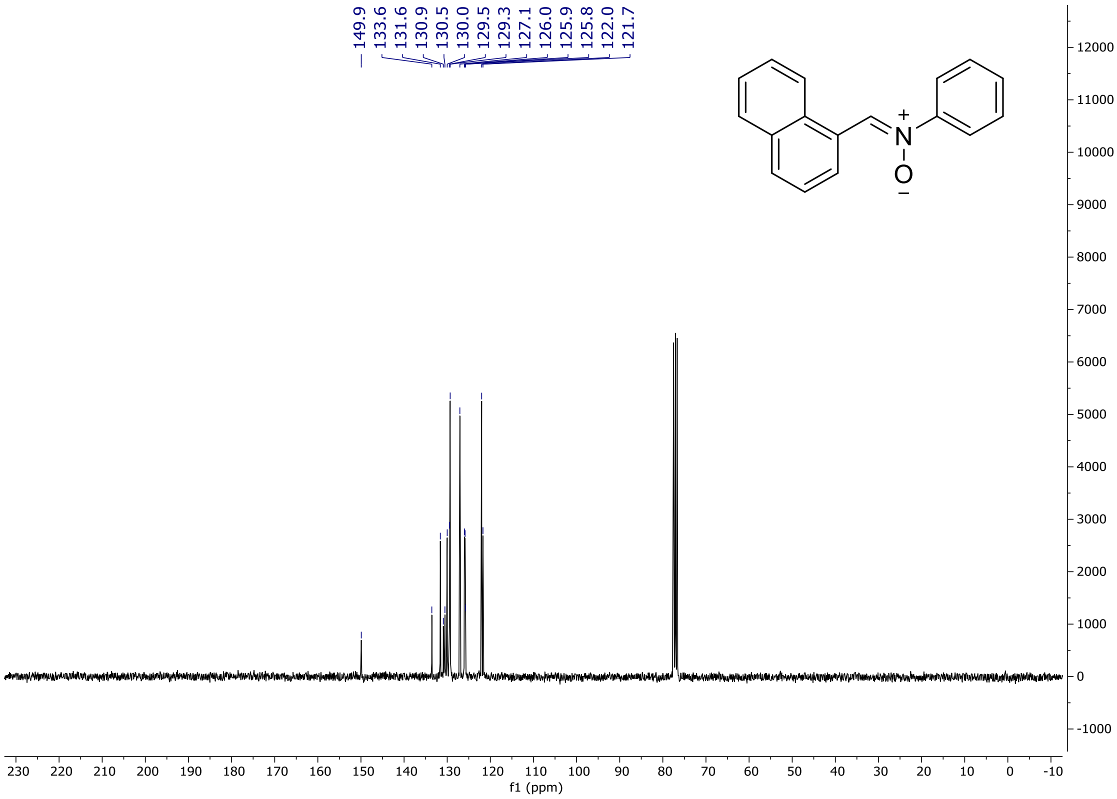

Figure SI_90: ^{13}C-NMR for **1y** in $CDCl_3$ (75 MHz).

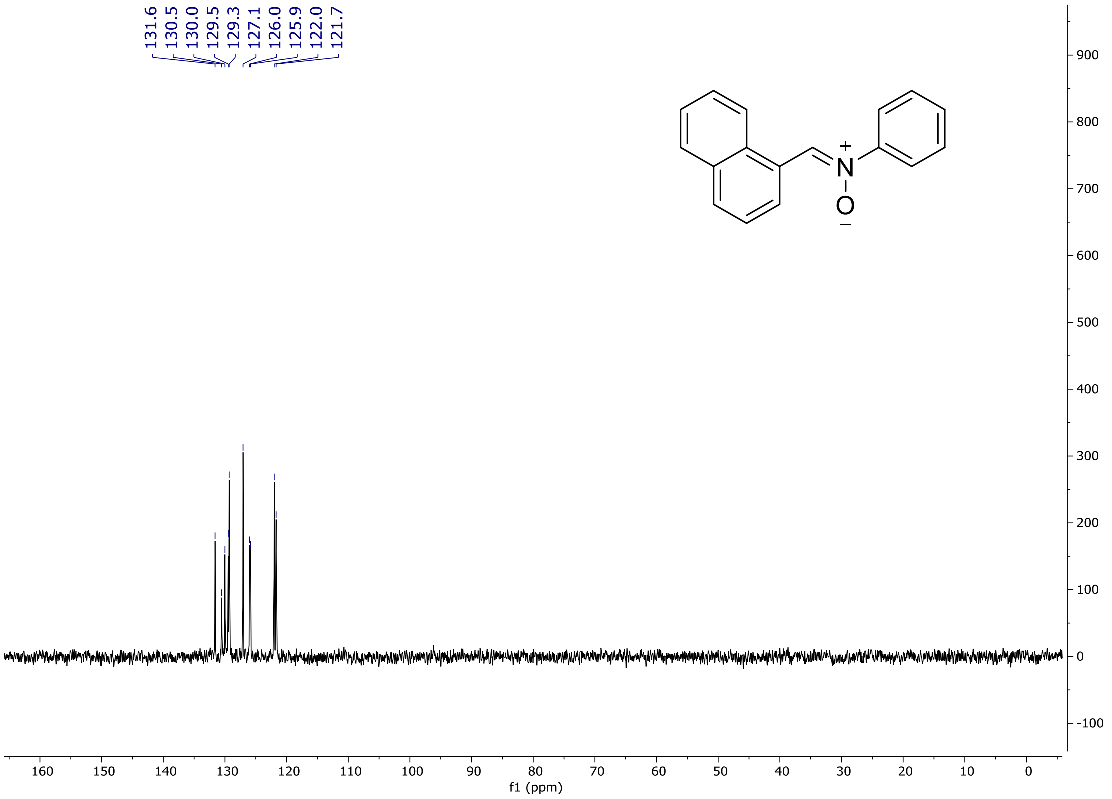

Figure SI_91: DEPT 135-NMR for **1y** in $CDCl_3$ (75 MHz).

***N*-Cinnamylidineaniline oxide (1z)**

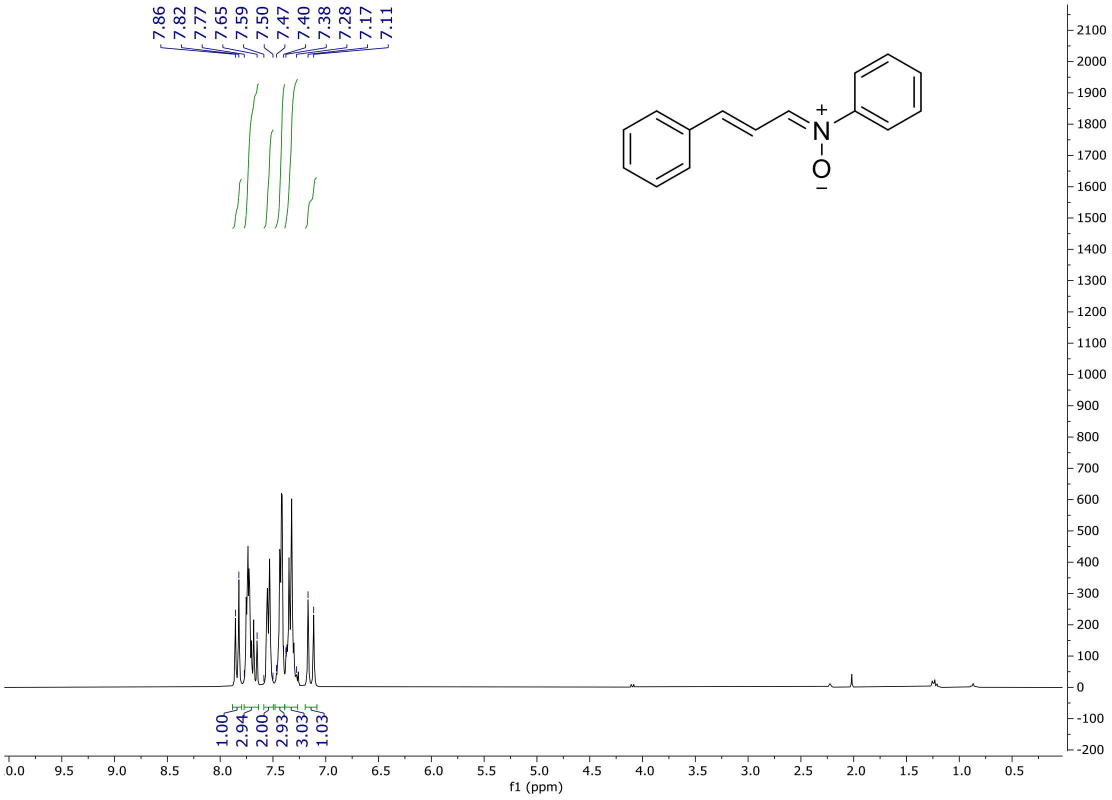

Figure SI_92: ^{1}H-NMR for **1z** in $CDCl_3$ (300 MHz).

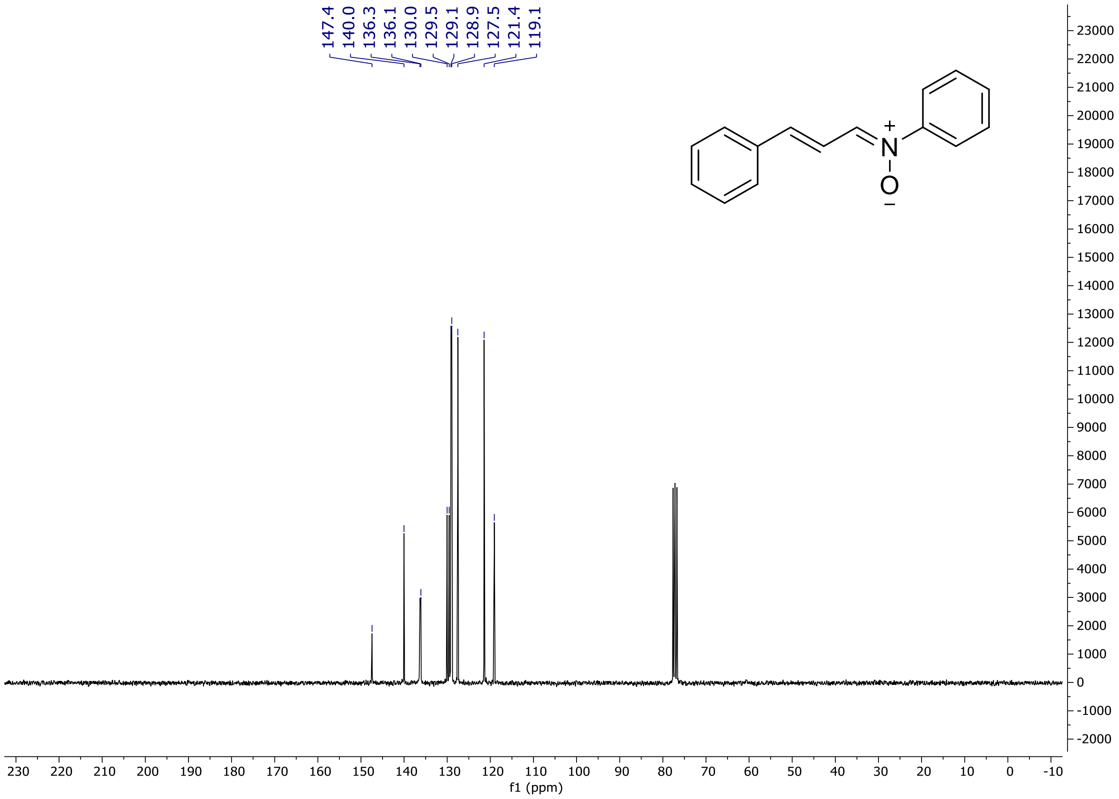

Figure SI_93: ^{13}C-NMR for **1z** in $CDCl_3$ (75 MHz).

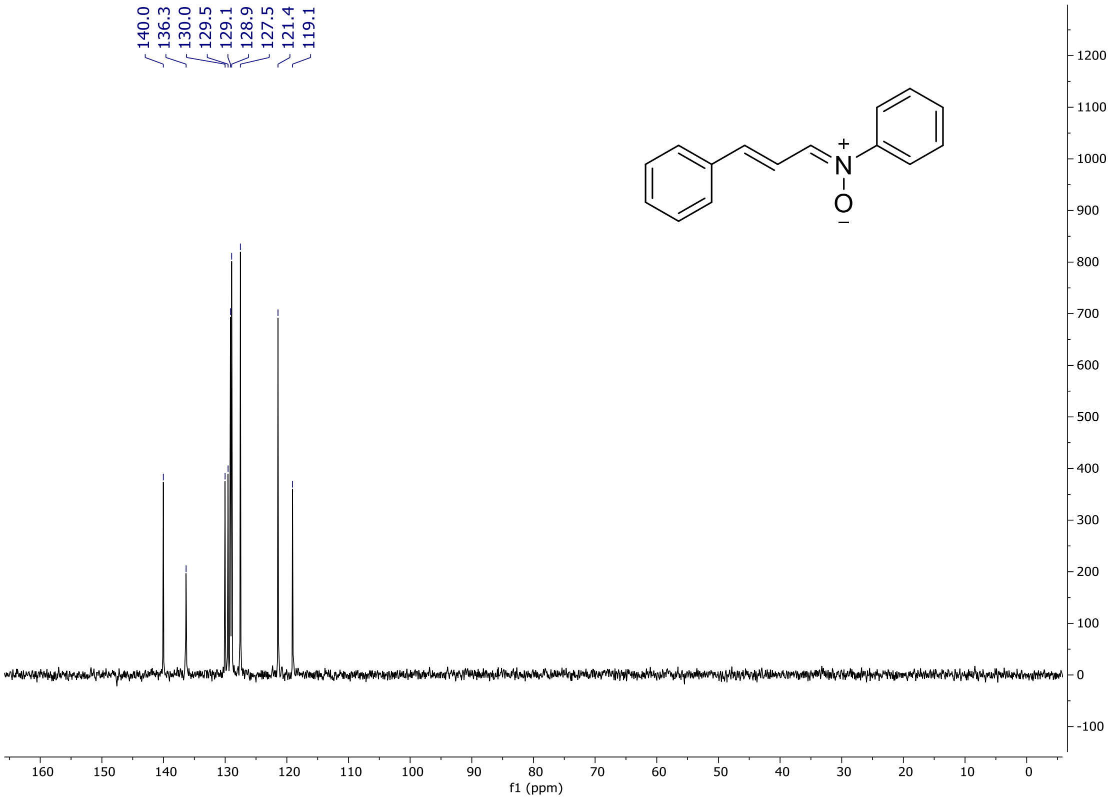

Figure SI_94: DEPT 135-NMR for **1z** in $CDCl_3$ (75 MHz).

N-(2-Methylpropylidine)aniline oxide (1aa)

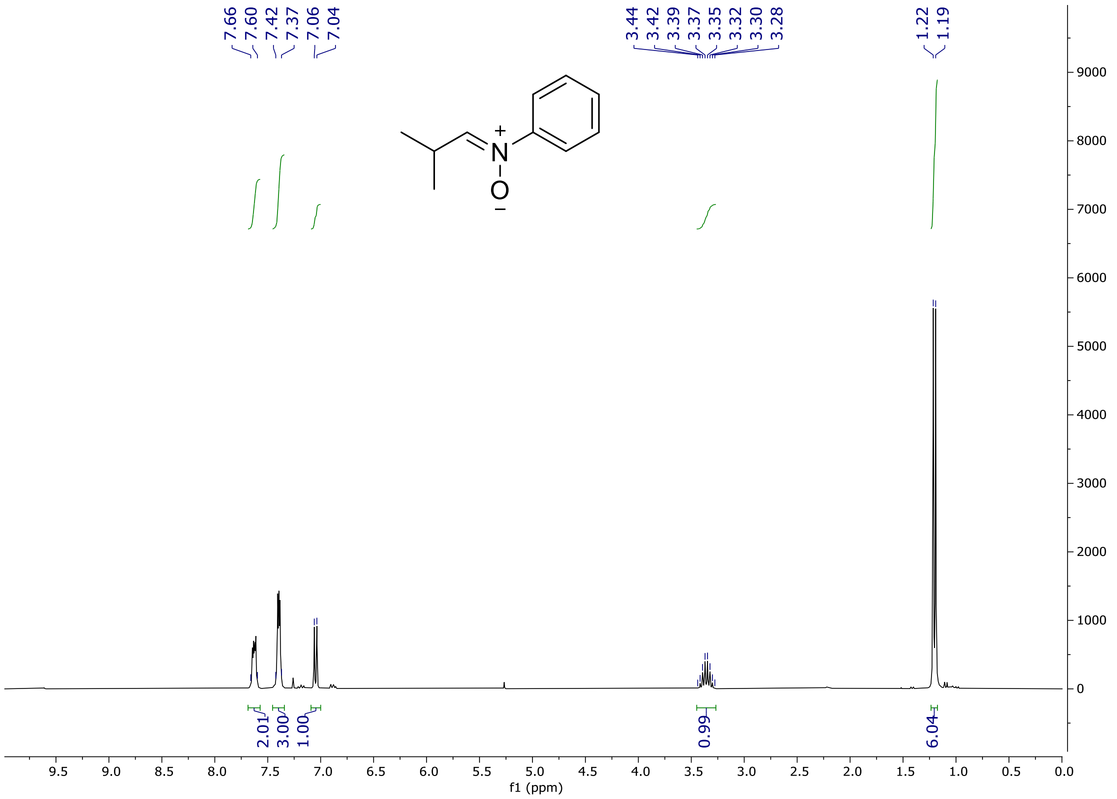

Figure SI_95: ^{1}H-NMR for **1aa** in $CDCl_3$ (300 MHz).

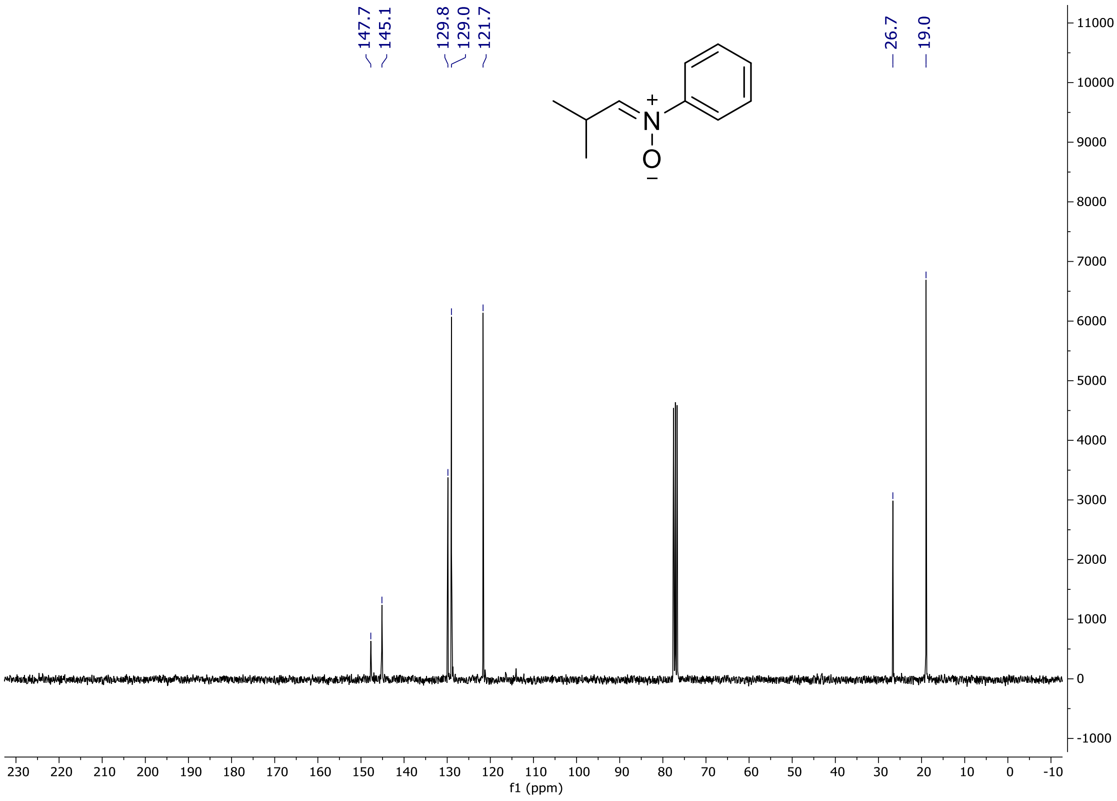

Figure SI_96: ^{13}C-NMR for **1aa** in $CDCl_3$ (75 MHz).

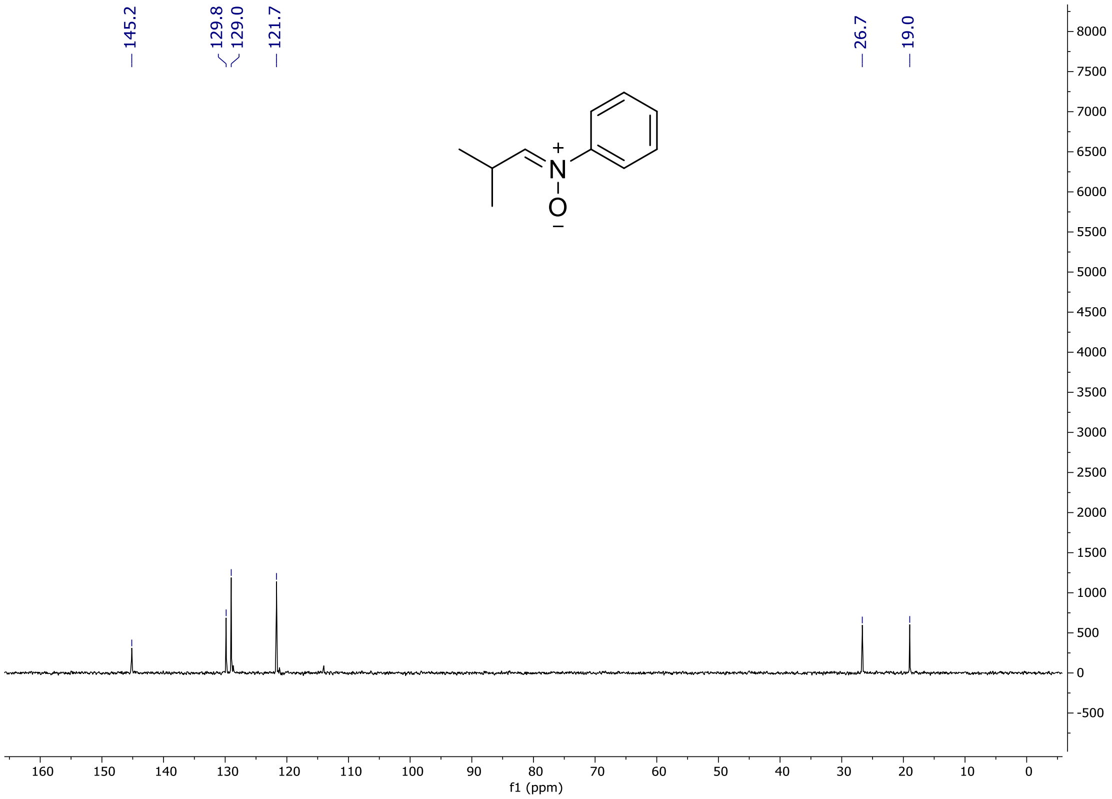

Figure SI_97: DEPT 135-NMR for **1aa** in $CDCl_3$ (75 MHz).

4-Fluoro-*N*-benzylideneaniline oxide (1ab)

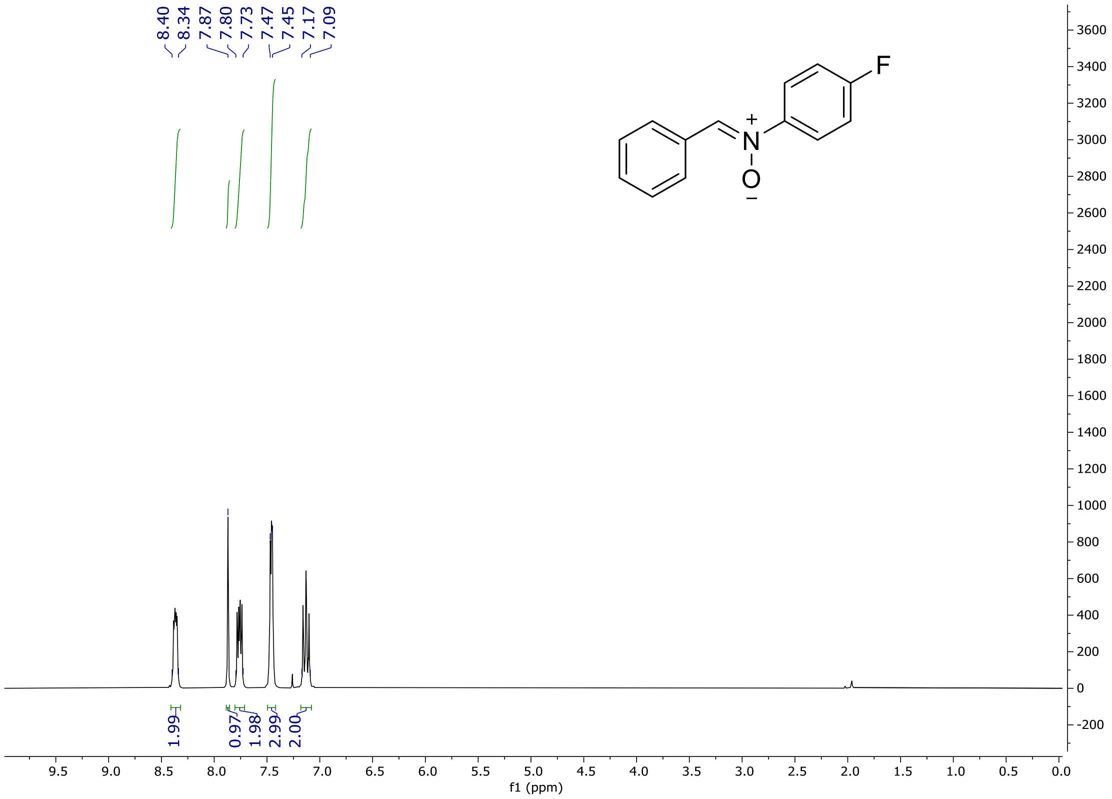

Figure SI_98: ^{1}H-NMR for **1ab** in $CDCl_3$ (300 MHz).

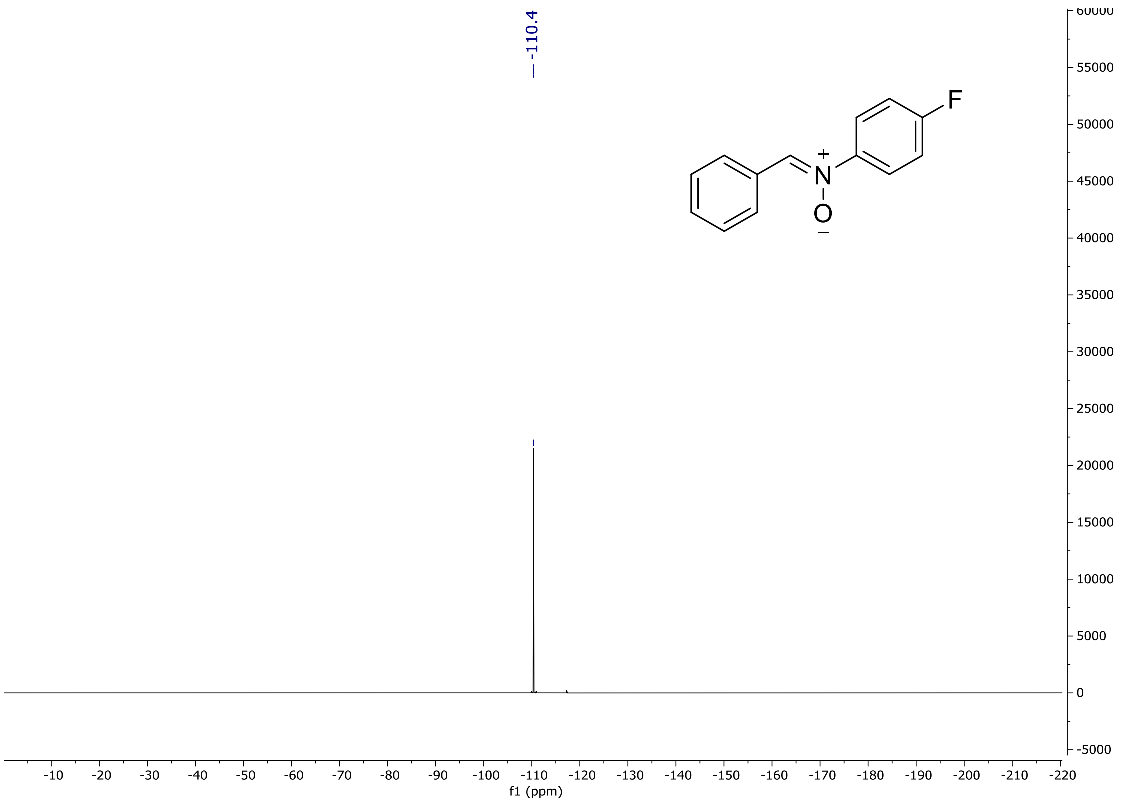

Figure SI_99: ^{19}F-NMR for **1ab** in $CDCl_3$ (282 MHz).

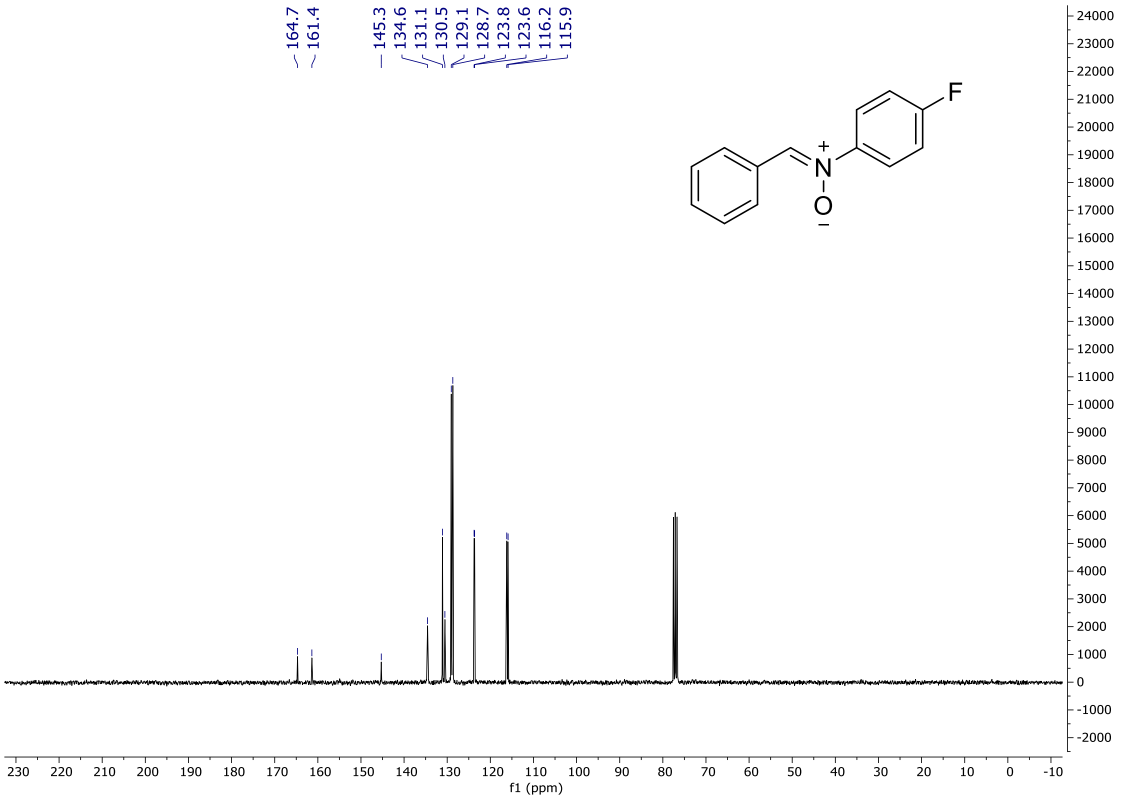

Figure SI_100: ^{13}C-NMR for **1ab** in $CDCl_3$ (75 MHz).

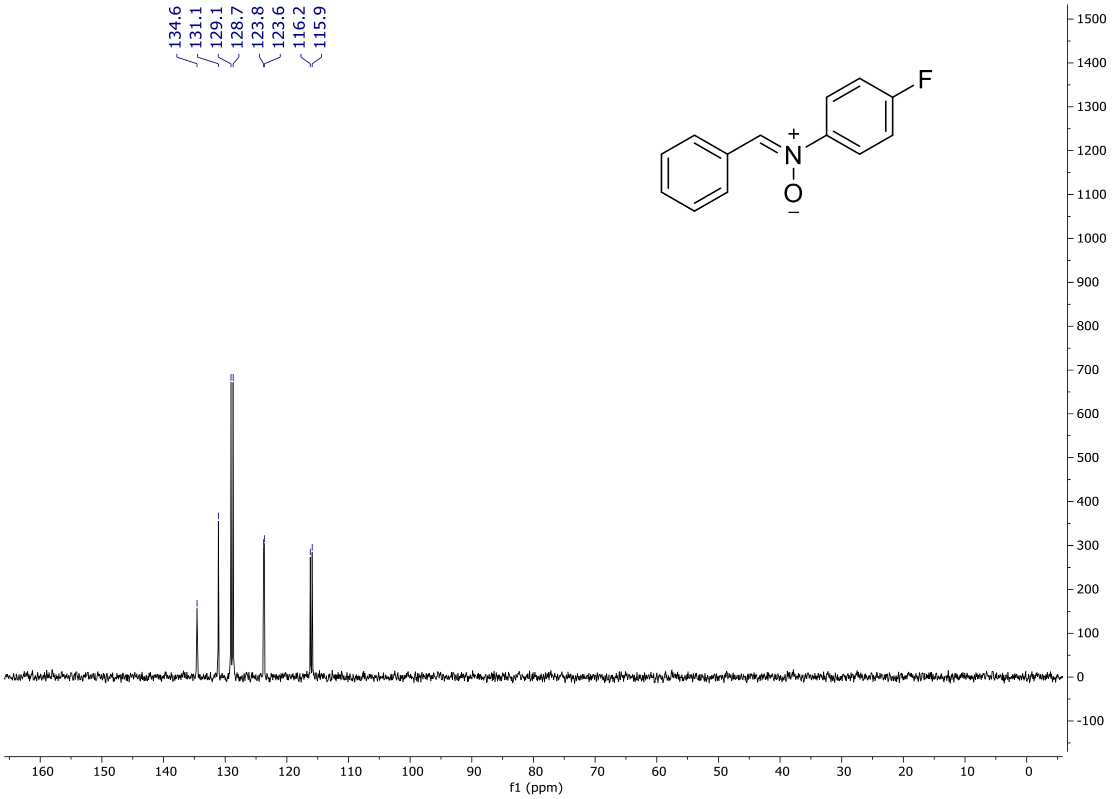

Figure SI_101: DEPT 135-NMR for **1ab** in $CDCl_3$ (75 MHz).

4-Bromo-*N*-benzylideneaniline oxide (1ac)

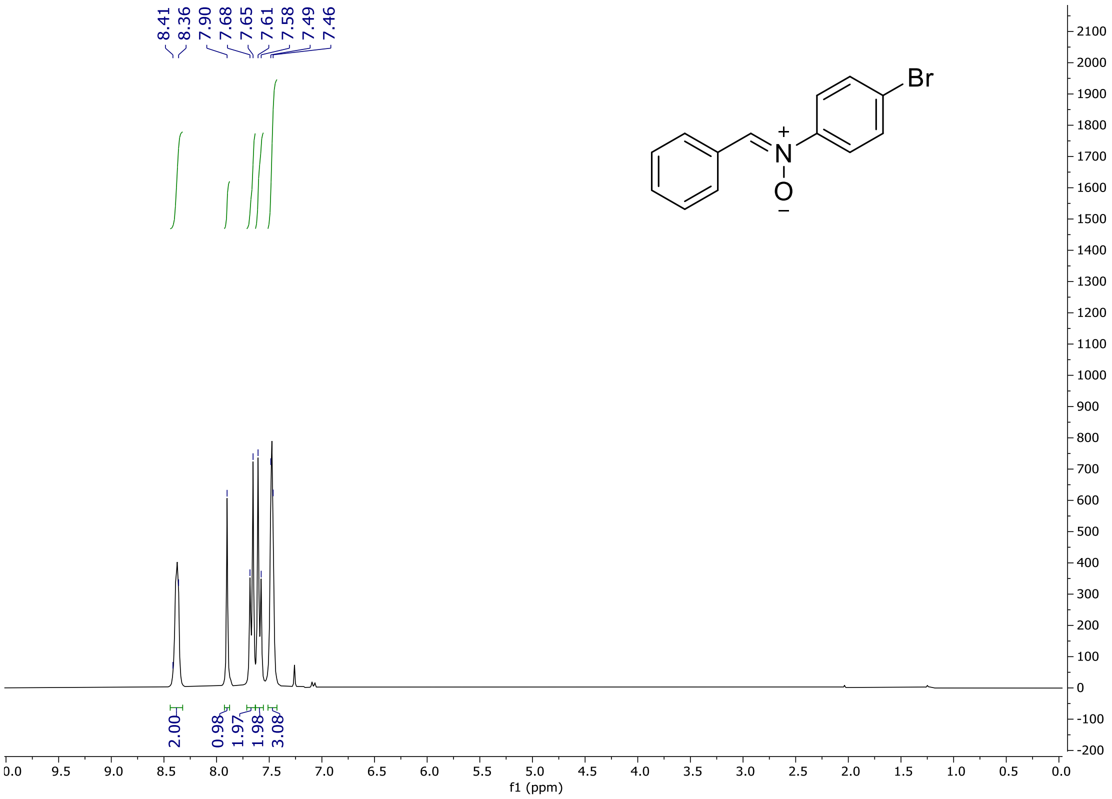

Figure SI_102: ^{1}H-NMR for **1ac** in $CDCl_3$ (300 MHz).

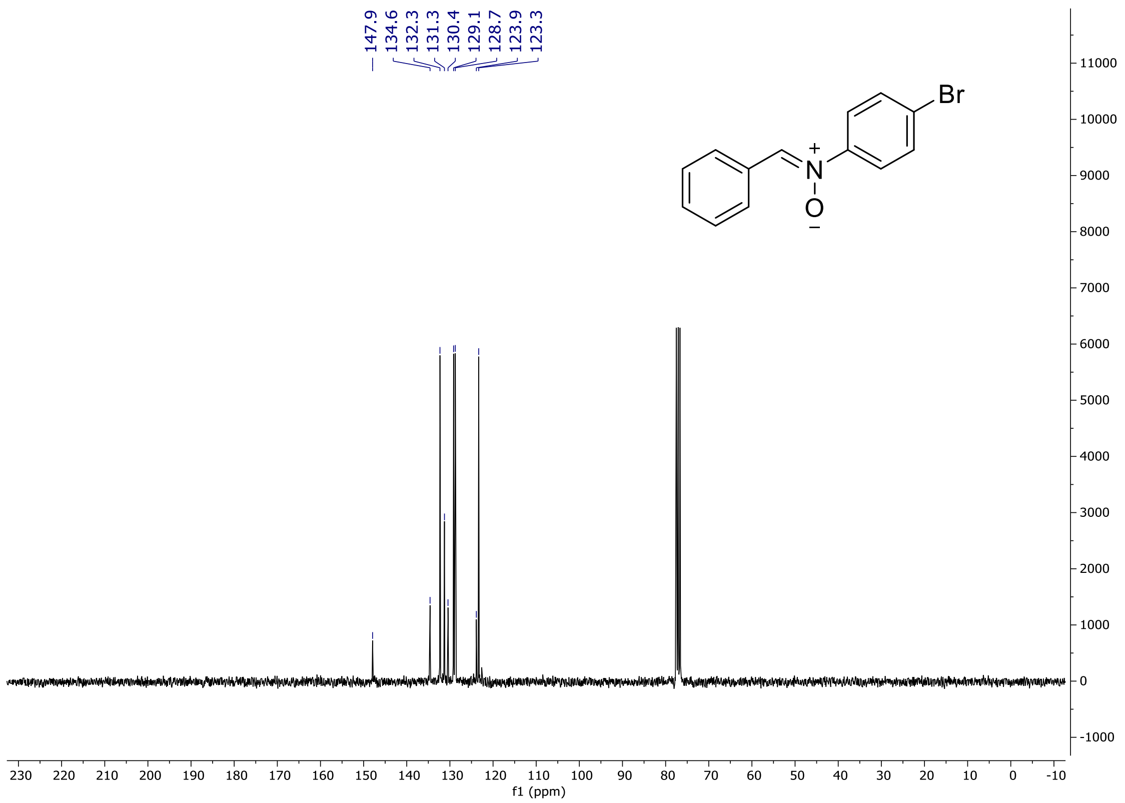

Figure SI_103: ^{13}C-NMR for **1ac** in $CDCl_3$ (75 MHz).

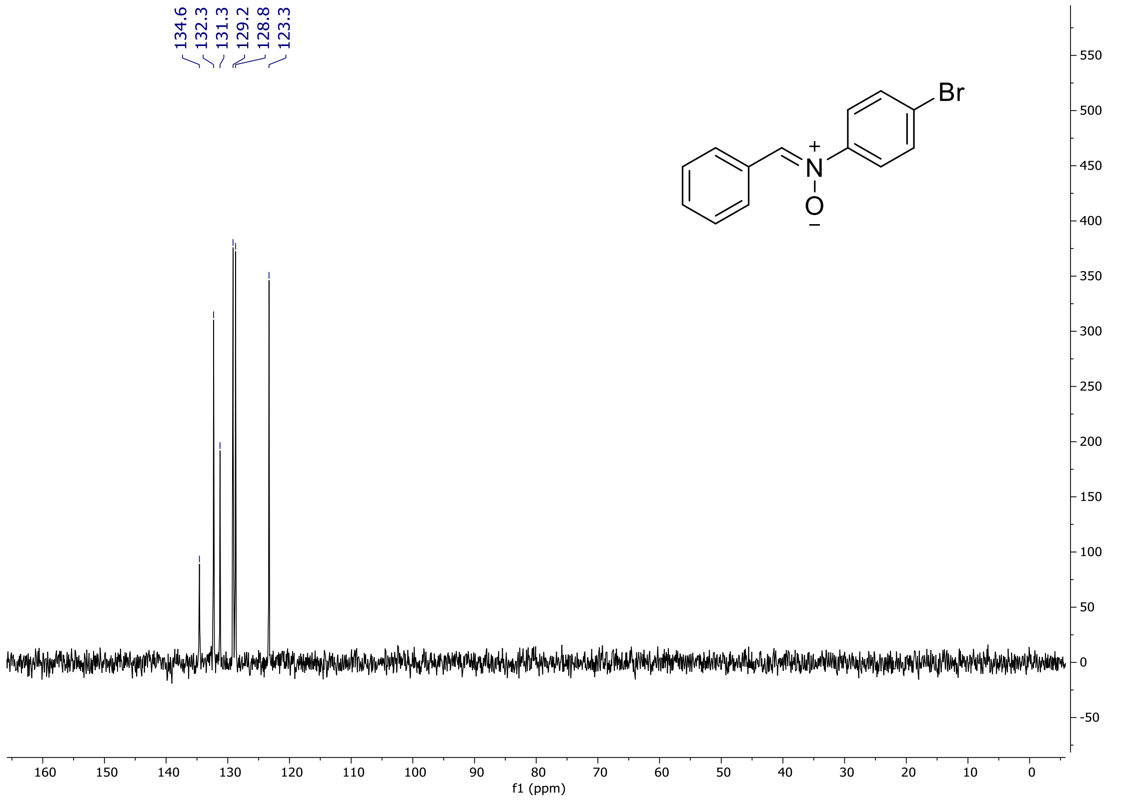

Figure SI_104: DEPT 135-NMR for **1ac** in $CDCl_3$ (75 MHz).

***N*-Benzylidenemethylamine oxide (1ad)**

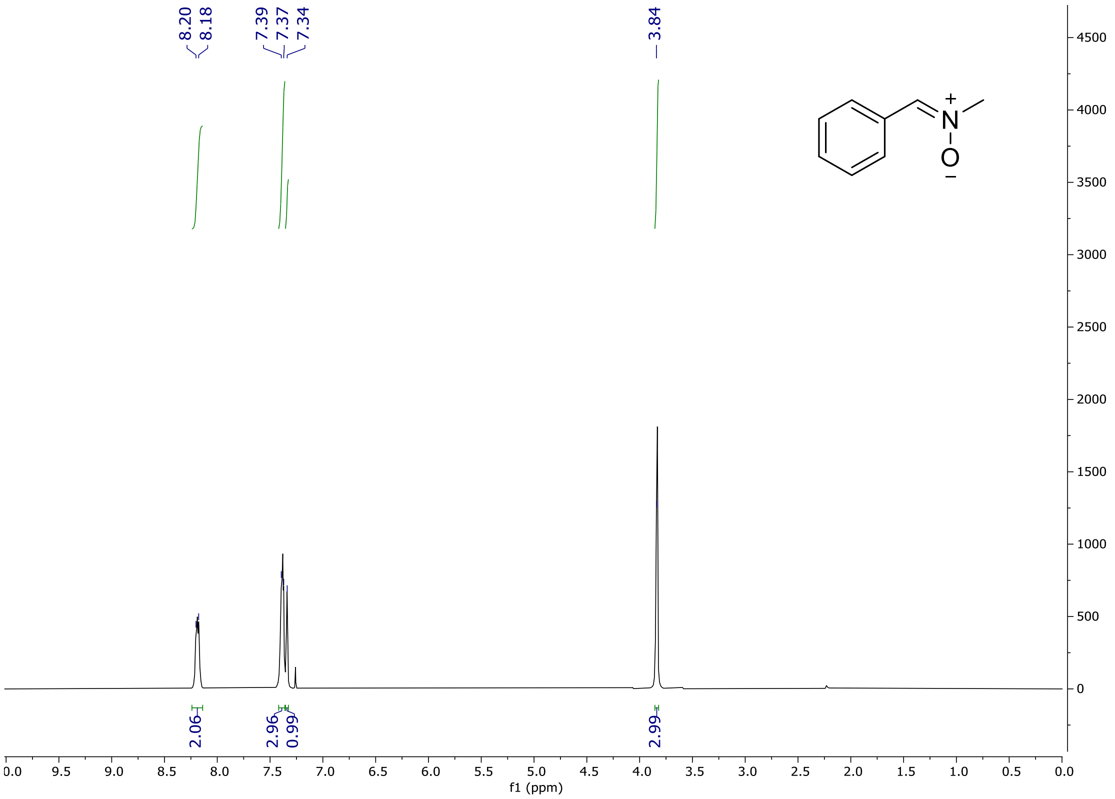

Figure SI_105: ^{1}H-NMR for **1ad** in $CDCl_3$ (300 MHz).

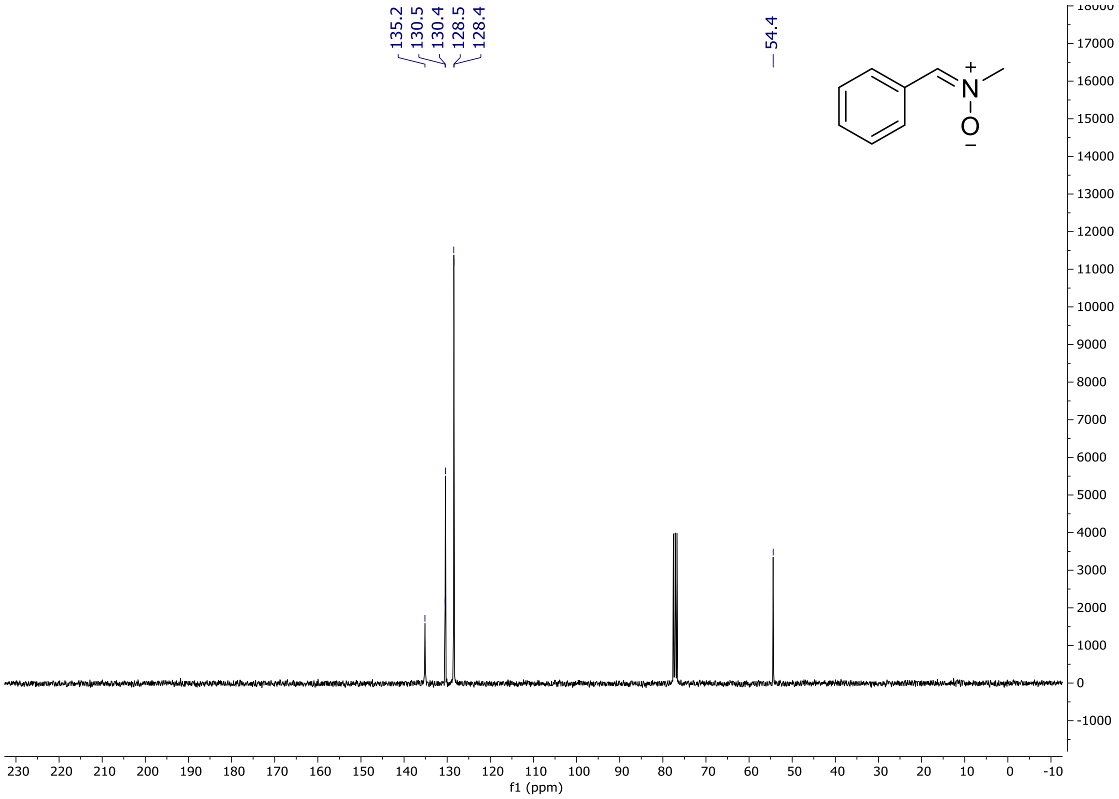

Figure SI_106: ^{13}C-NMR for **1ad** in $CDCl_3$ (75 MHz).

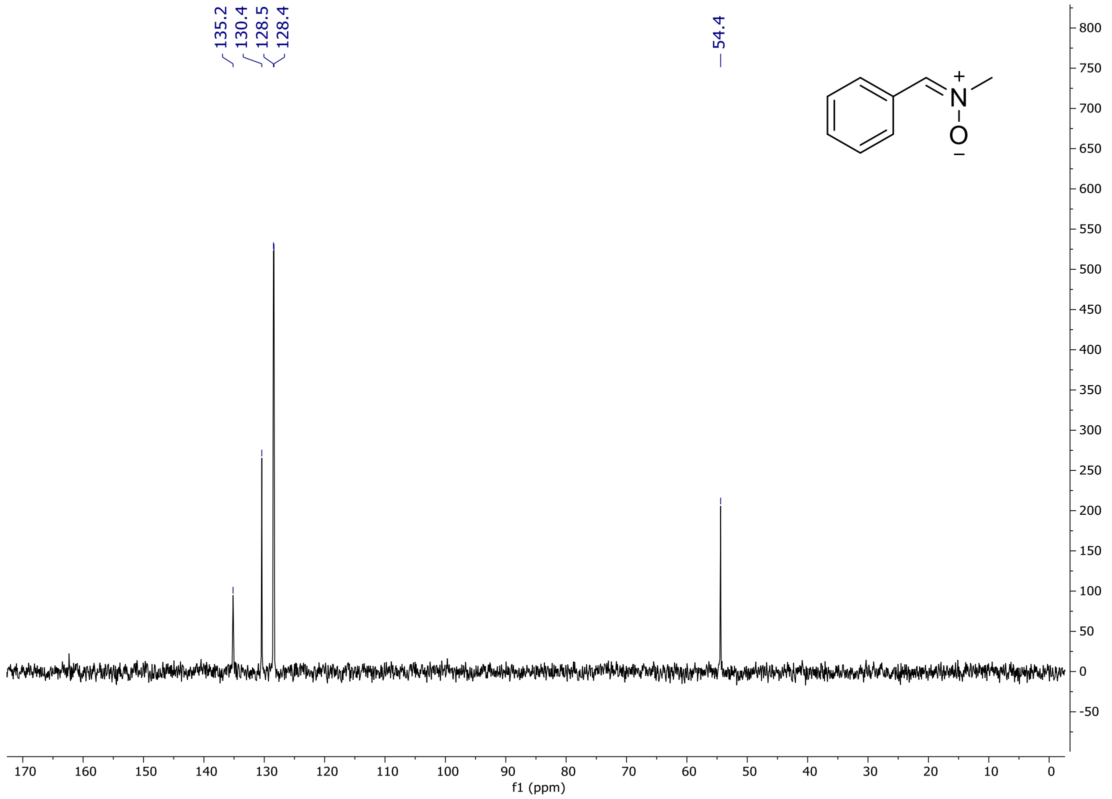

Figure SI_107: DEPT 135-NMR for **1ad** in $CDCl_3$ (75 MHz).

***N*-Benzylidenebenzylamine oxide (1ae)**

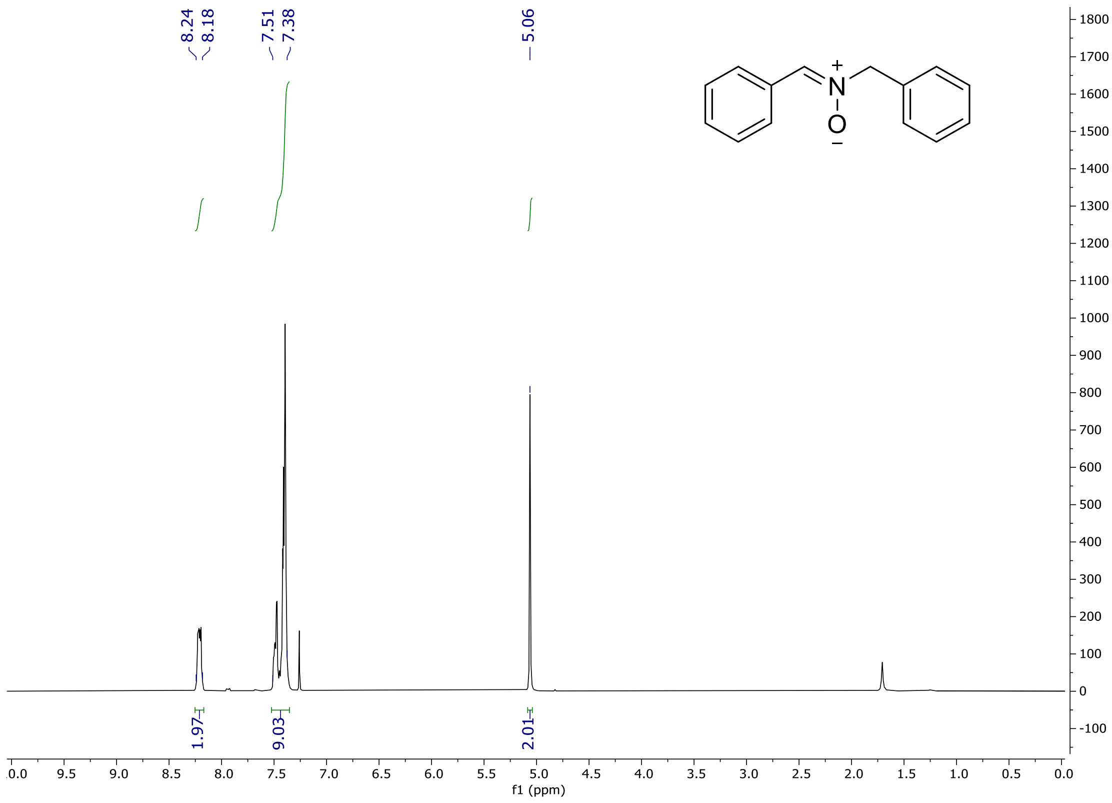

Figure SI_108: ^{1}H-NMR for **1ae** in $CDCl_3$ (300 MHz).

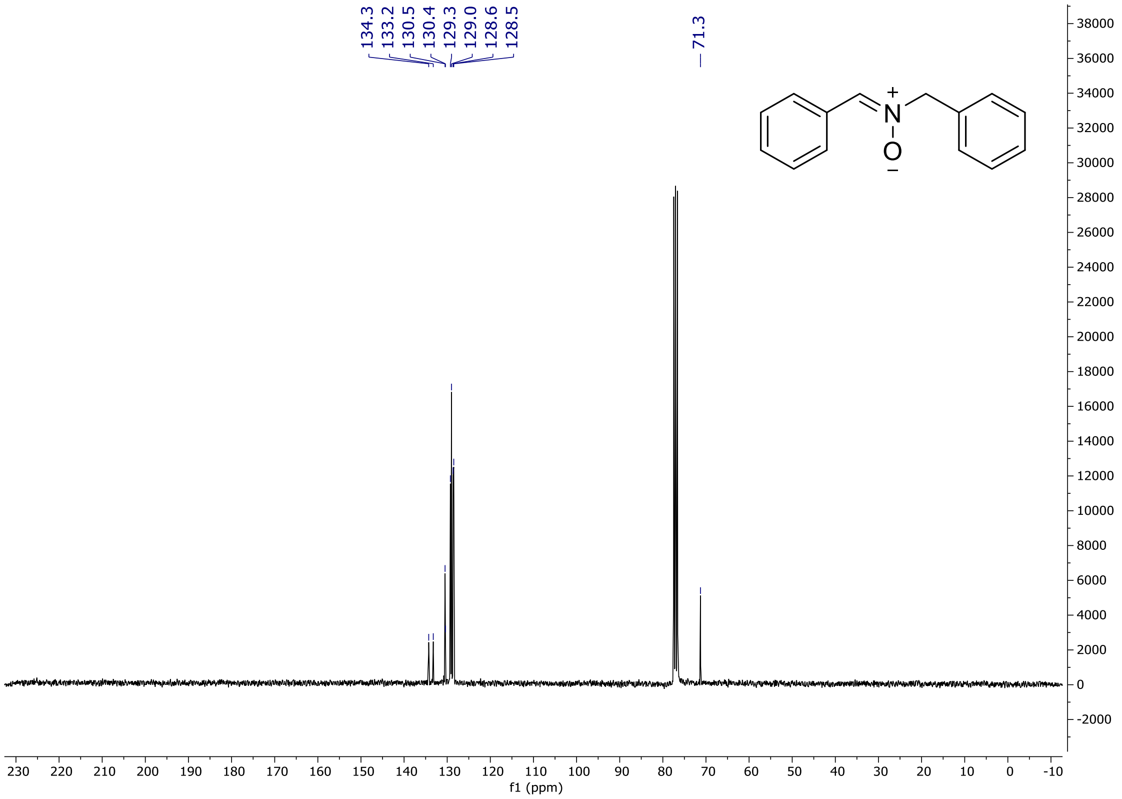

Figure SI_109: ^{13}C-NMR for **1ae** in $CDCl_3$ (75 MHz).

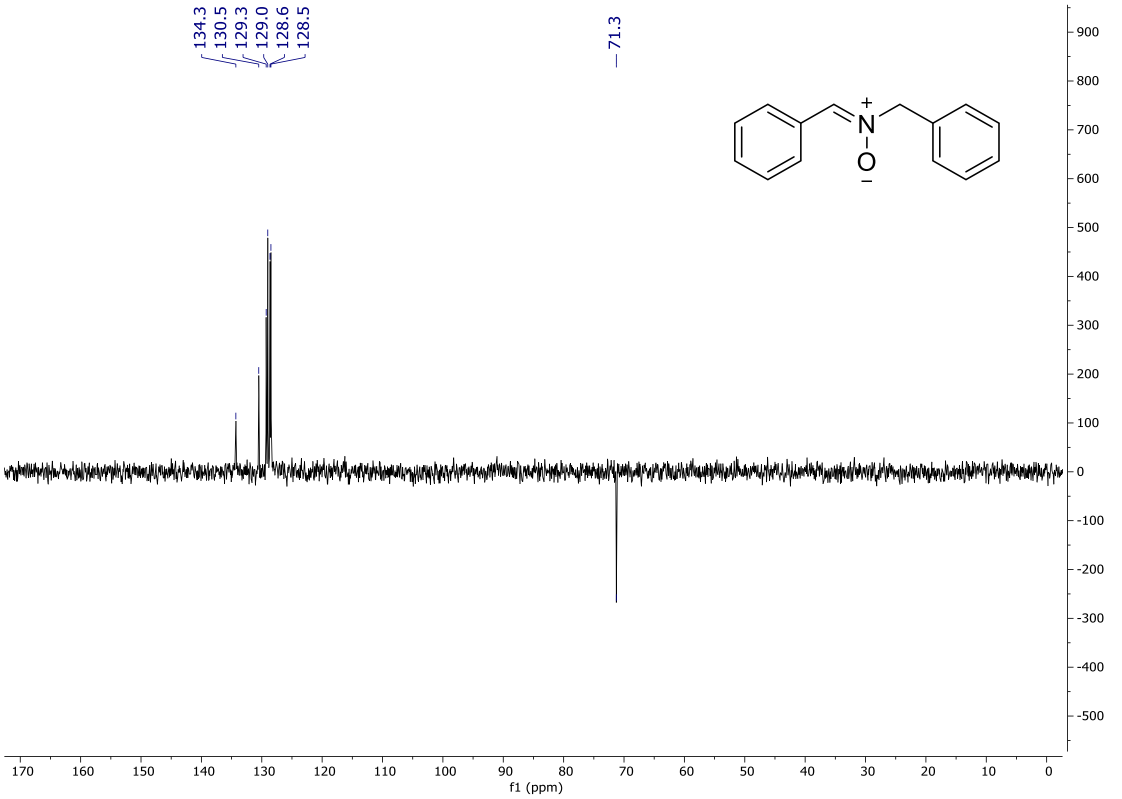

Figure SI_110: DEPT 135-NMR for **1ae** in $CDCl_3$ (75 MHz).

3,4-Dihydroisoquinoline *N*-Oxide (1af)

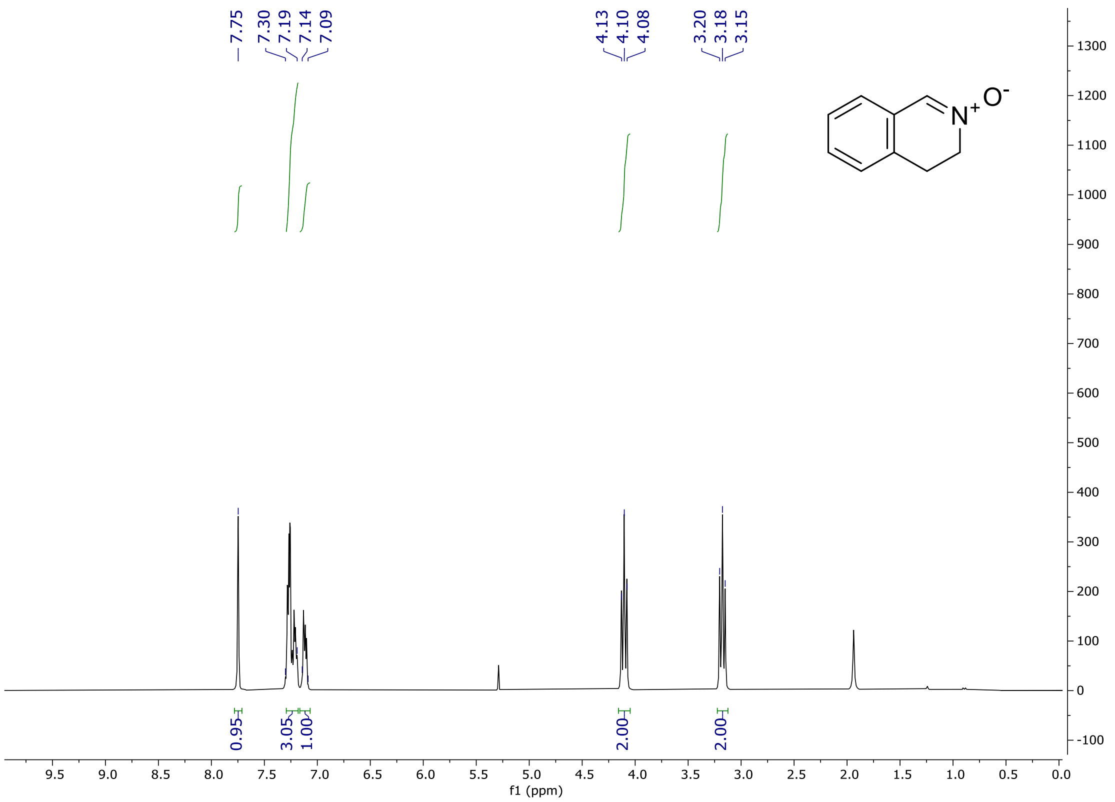

Figure SI_111: ^{1}H-NMR for **1af** in $CDCl_3$ (300 MHz).

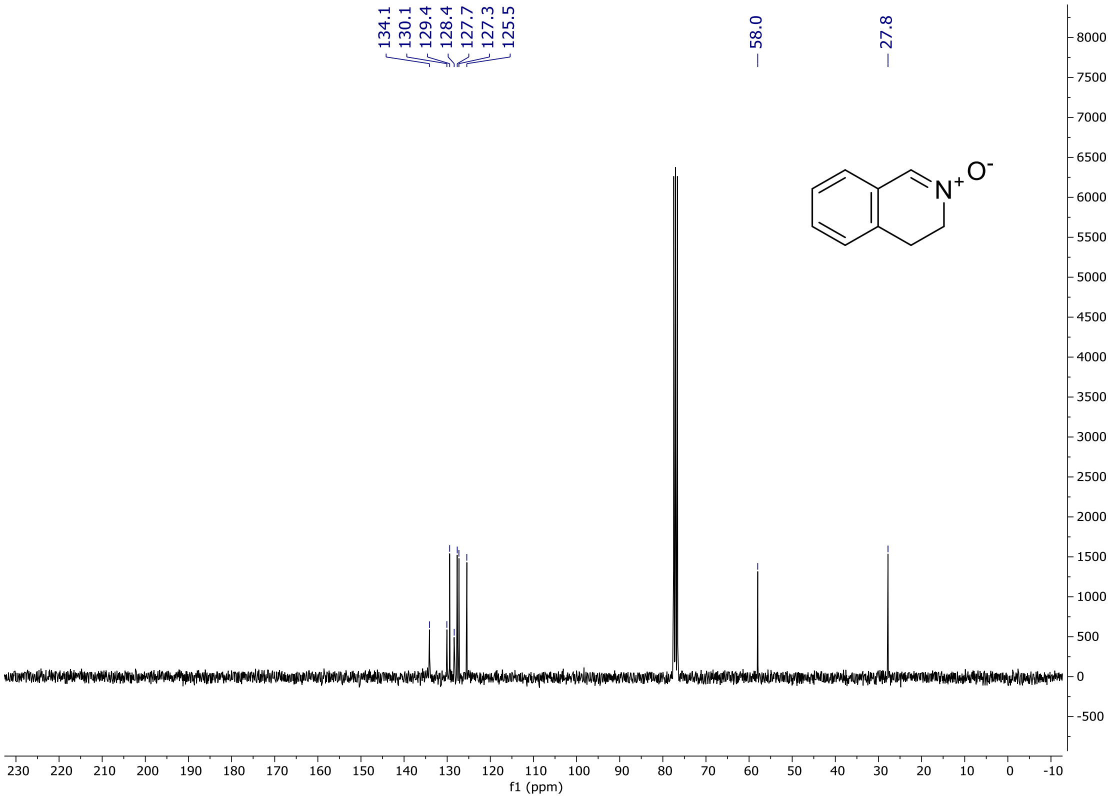

Figure SI_112: ^{13}C-NMR for **1af** in $CDCl_3$ (75 MHz).

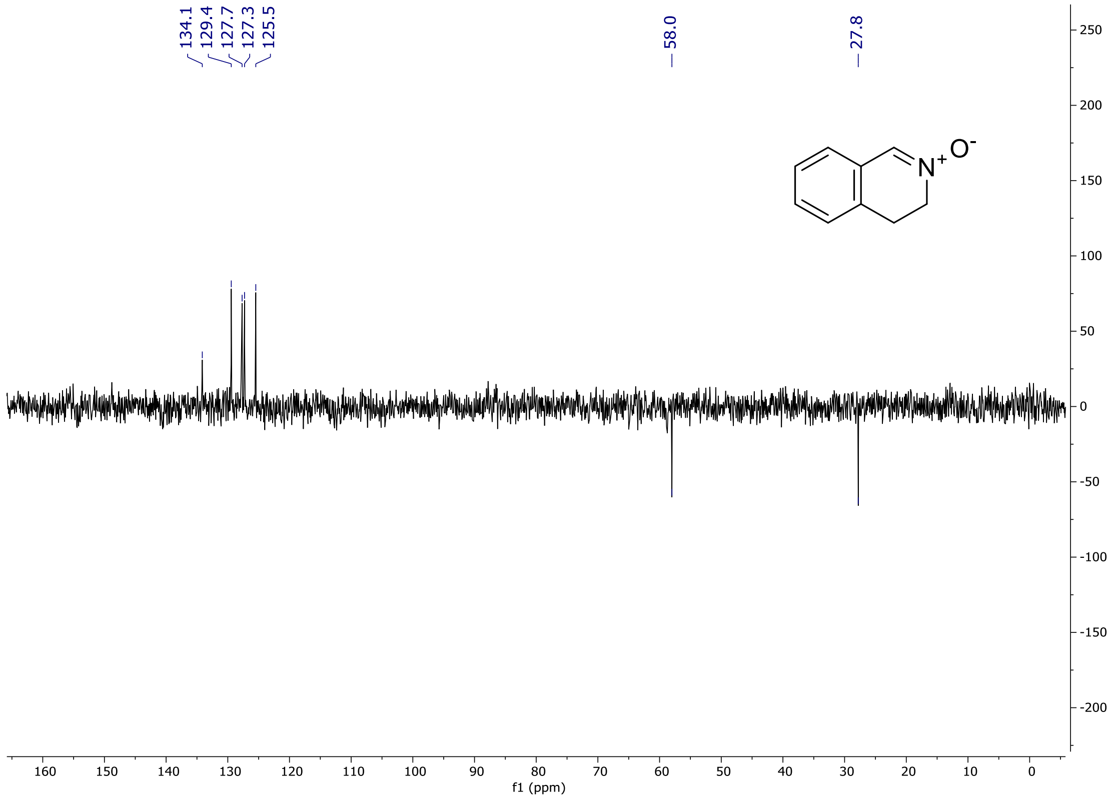

Figure SI_113: DEPT 135-NMR for **1af** in $CDCl_3$ (75 MHz).

3,4-Dihydropyrrole *N*-Oxide (1ag)

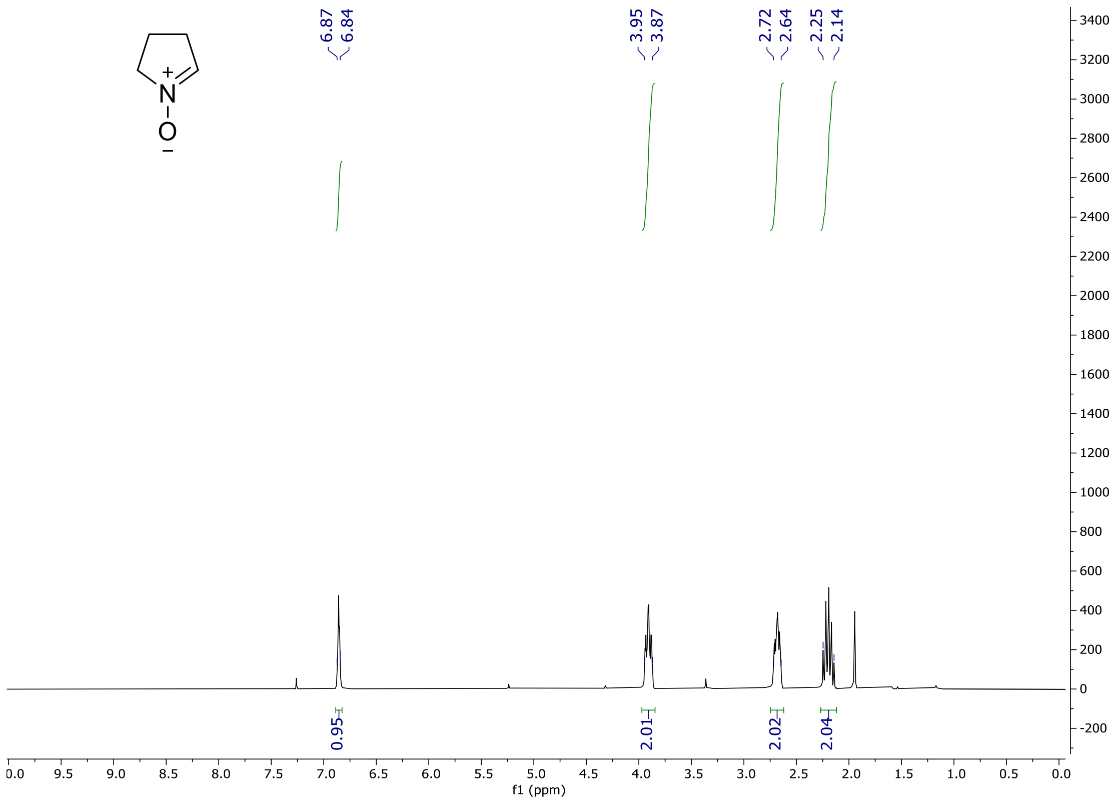

Figure SI_114: ^{1}H-NMR for **1ag** in $CDCl_3$ (300 MHz).

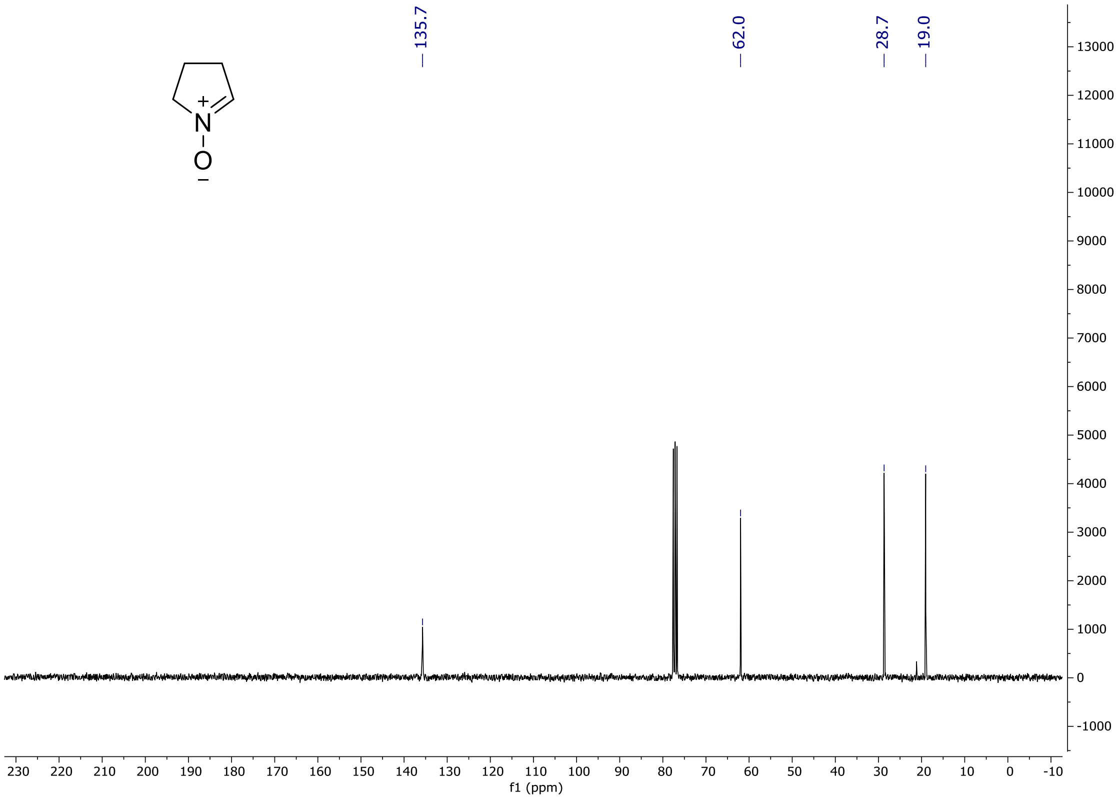

Figure SI_115: ^{13}C-NMR for **1ag** in $CDCl_3$ (75 MHz).

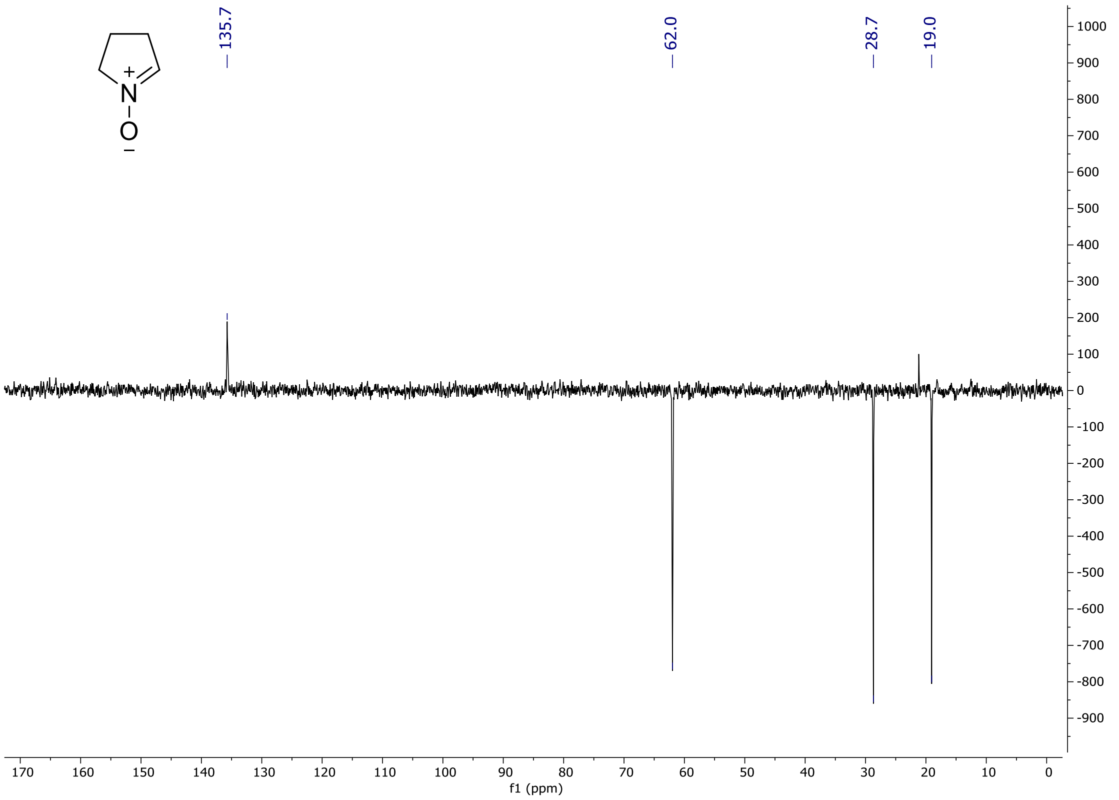

Figure SI_116: DEPT 135-NMR for **1ag** in $CDCl_3$ (75 MHz).

N-Ethylethanimine oxide (1ah)

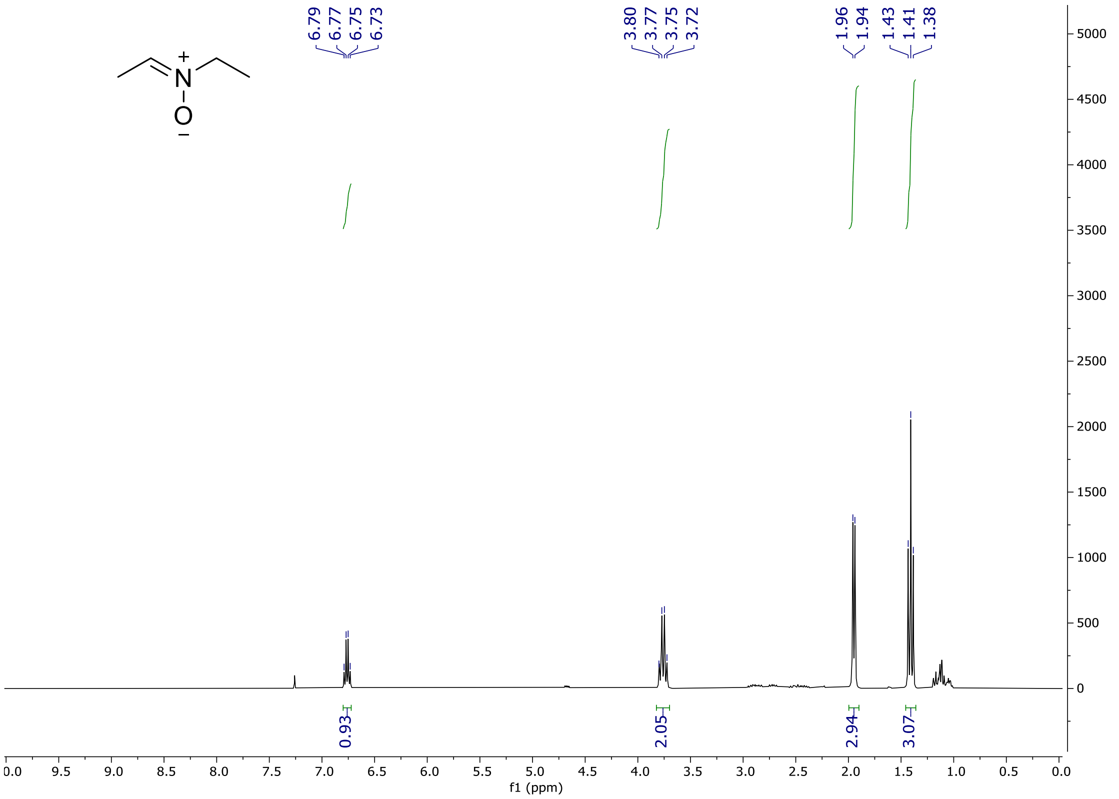

Figure SI_117: ^{1}H-NMR for **1ah** in $CDCl_3$ (300 MHz).

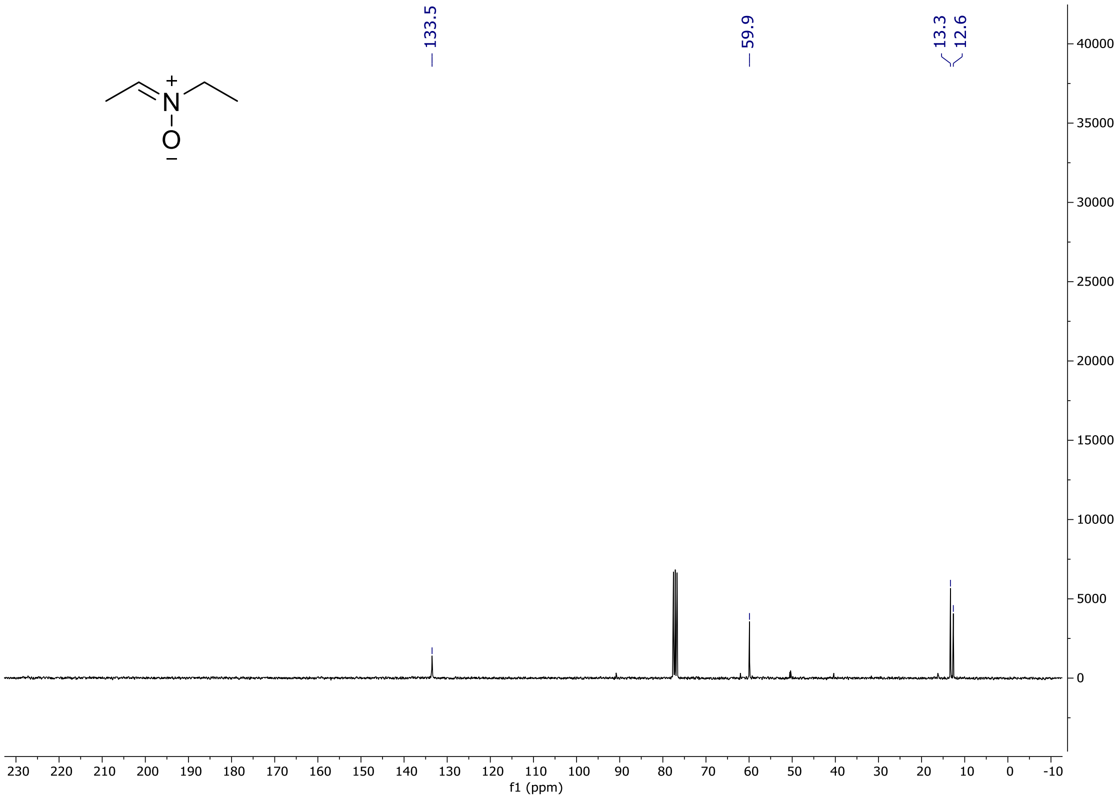

Figure SI_118: ^{13}C-NMR for **1ah** in $CDCl_3$ (75 MHz).

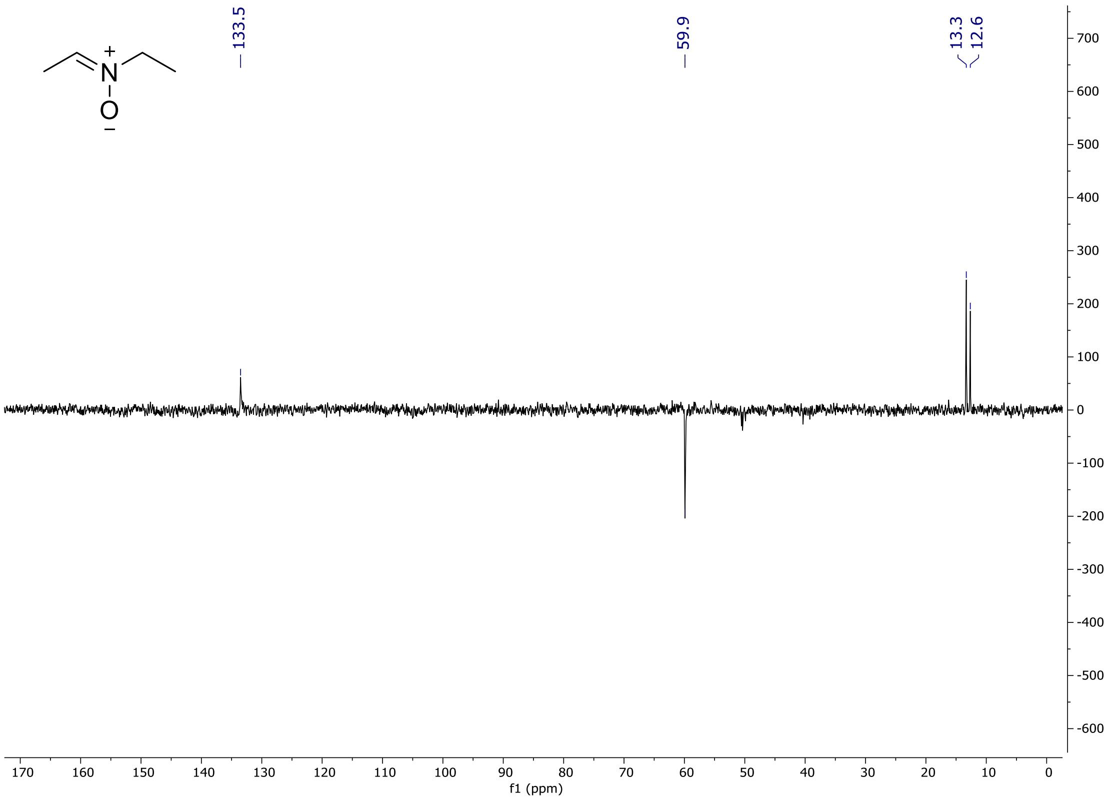

Figure SI_119: DEPT 135-NMR for **1ah** in $CDCl_3$ (75 MHz).

4-Chloro-*N*-phenylbenzothioamide (3a)

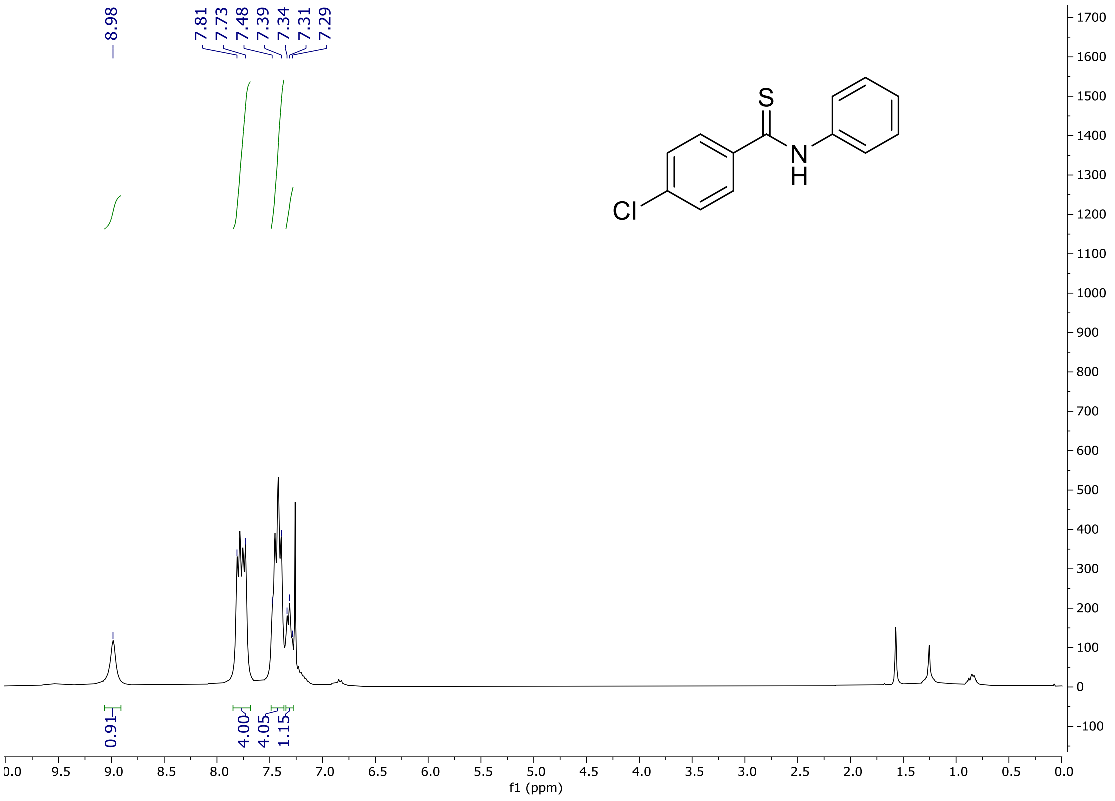

Figure SI_120: ^{1}H-NMR for **3a** in $CDCl_3$ (300 MHz).

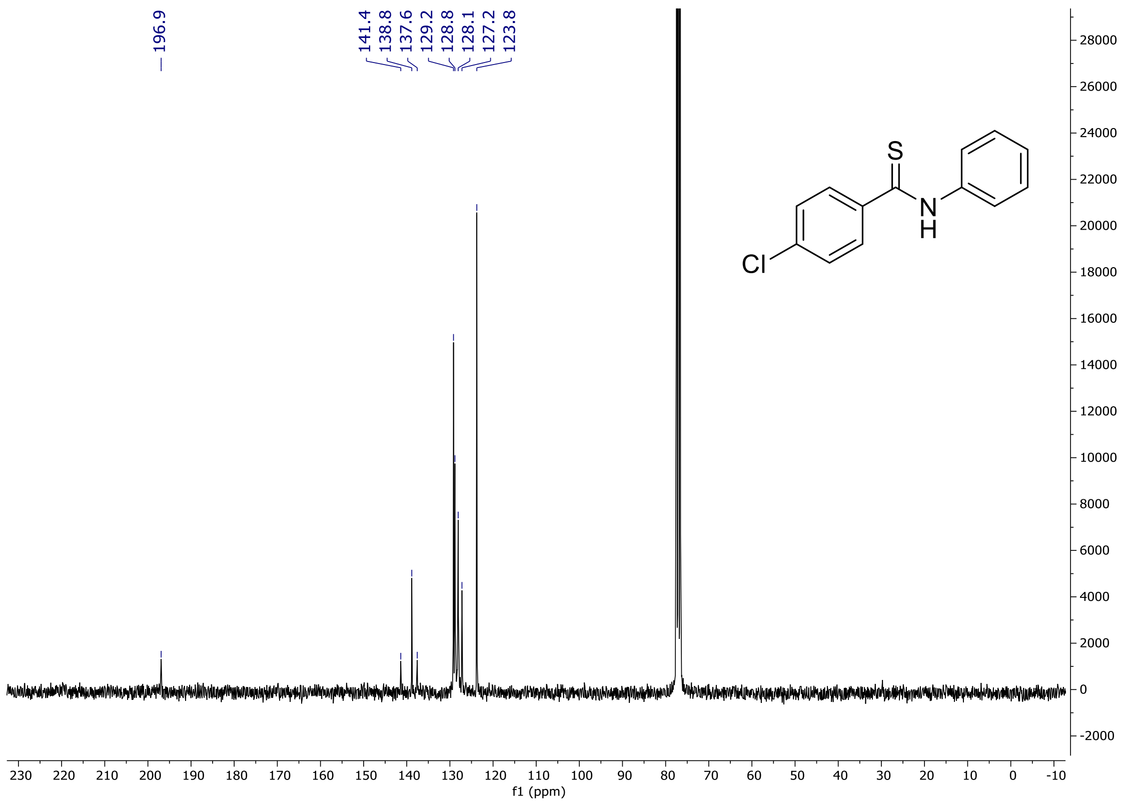

Figure SI_121: ^{13}C-NMR for **3a** in $CDCl_3$ (75 MHz).

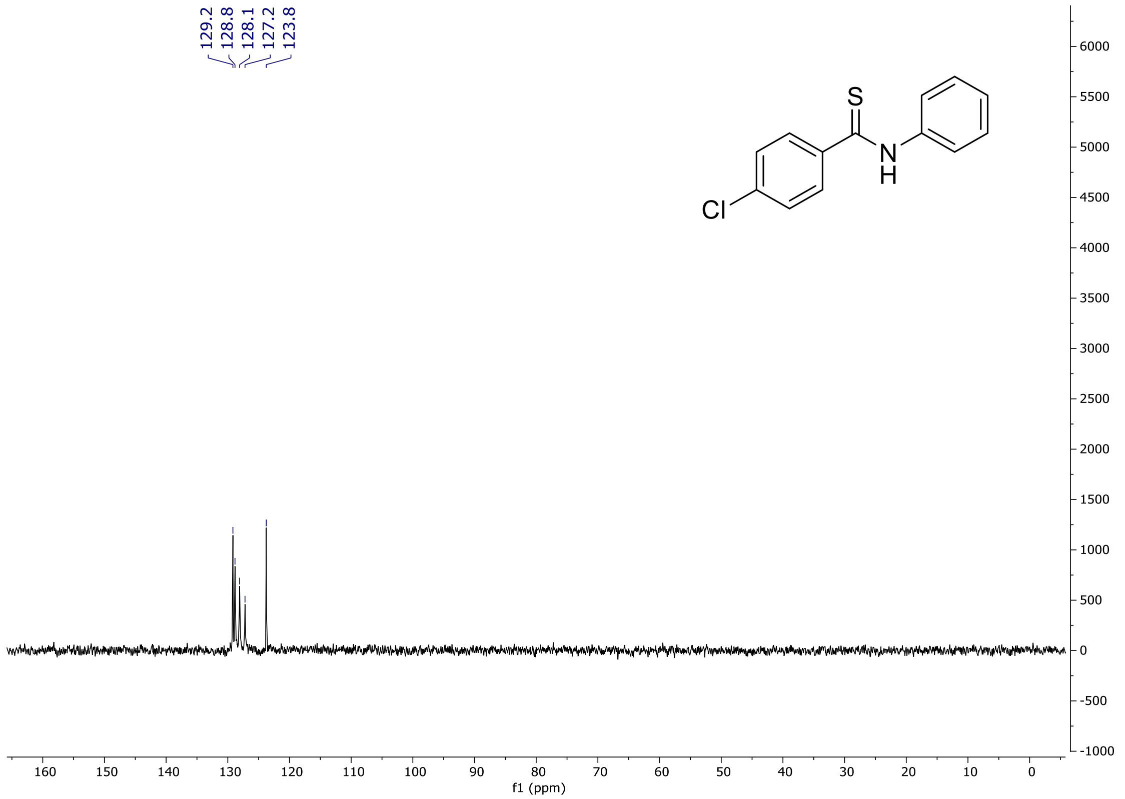

Figure SI_122: DEPT 135-NMR for **3a** in $CDCl_3$ (75 MHz).

N-Phenylbenzothioamide (3b)

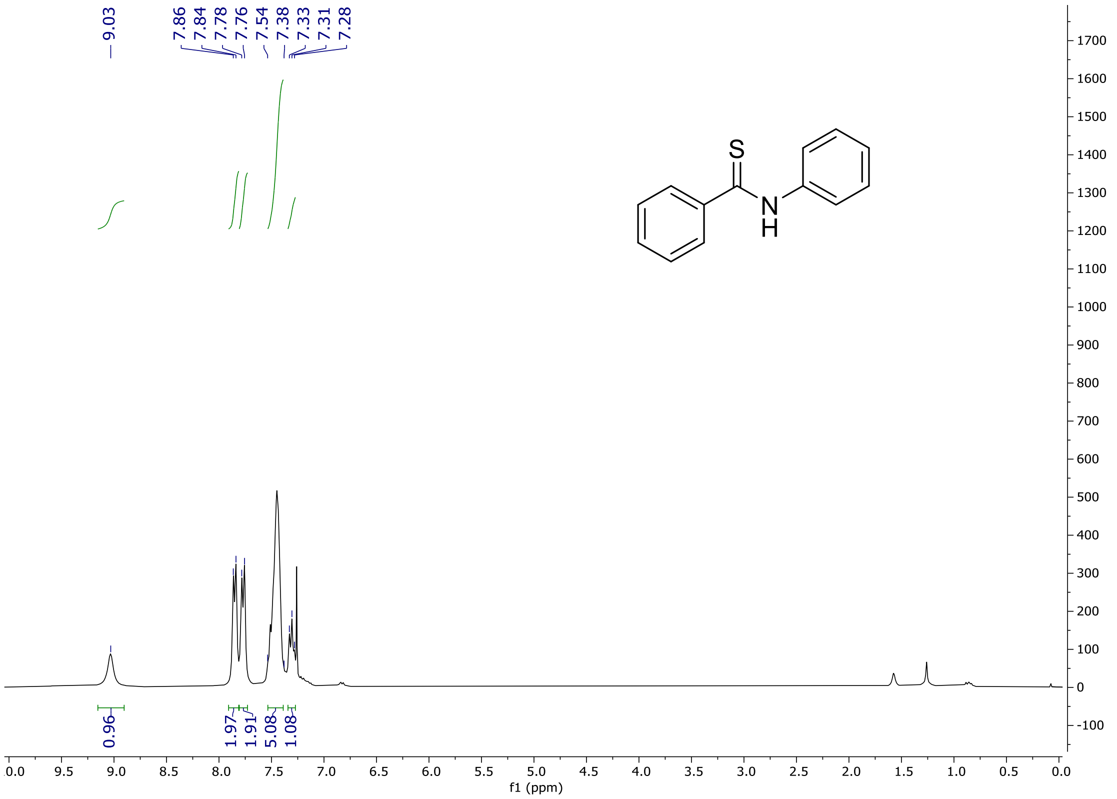

Figure SI_123: ^{1}H-NMR for **3b** in $CDCl_3$ (300 MHz).

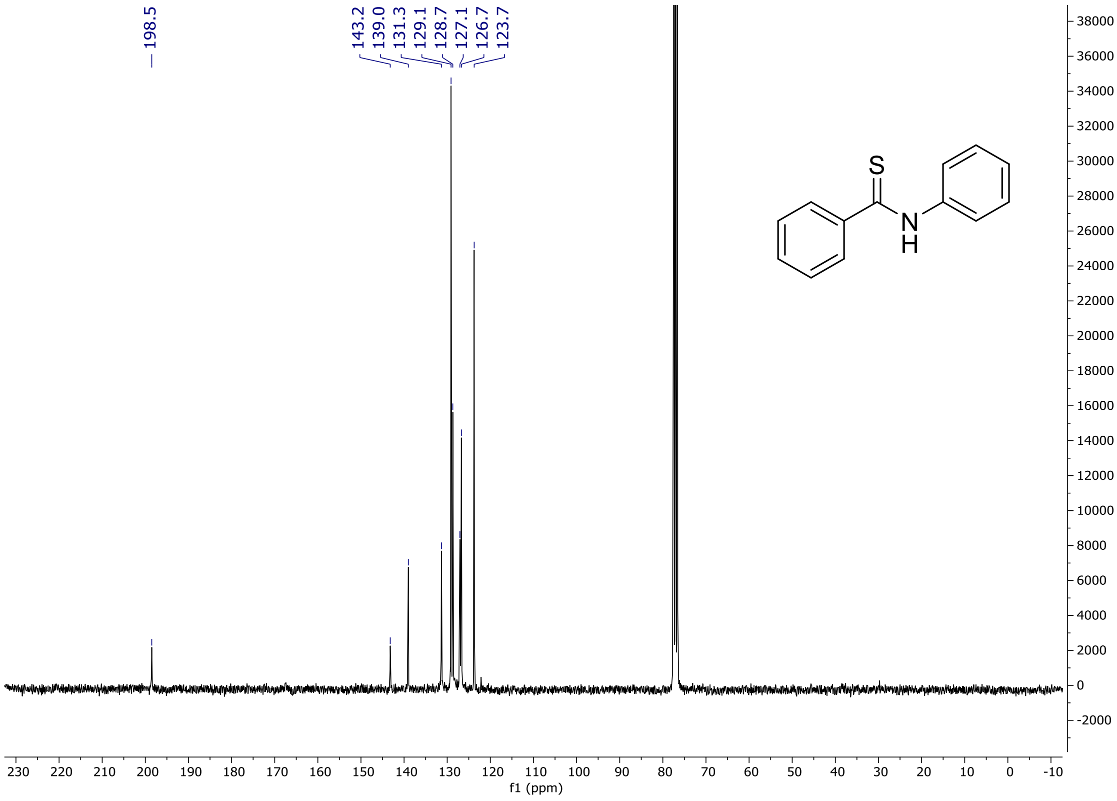

Figure SI_124: ^{13}C-NMR for **3b** in $CDCl_3$ (75 MHz).

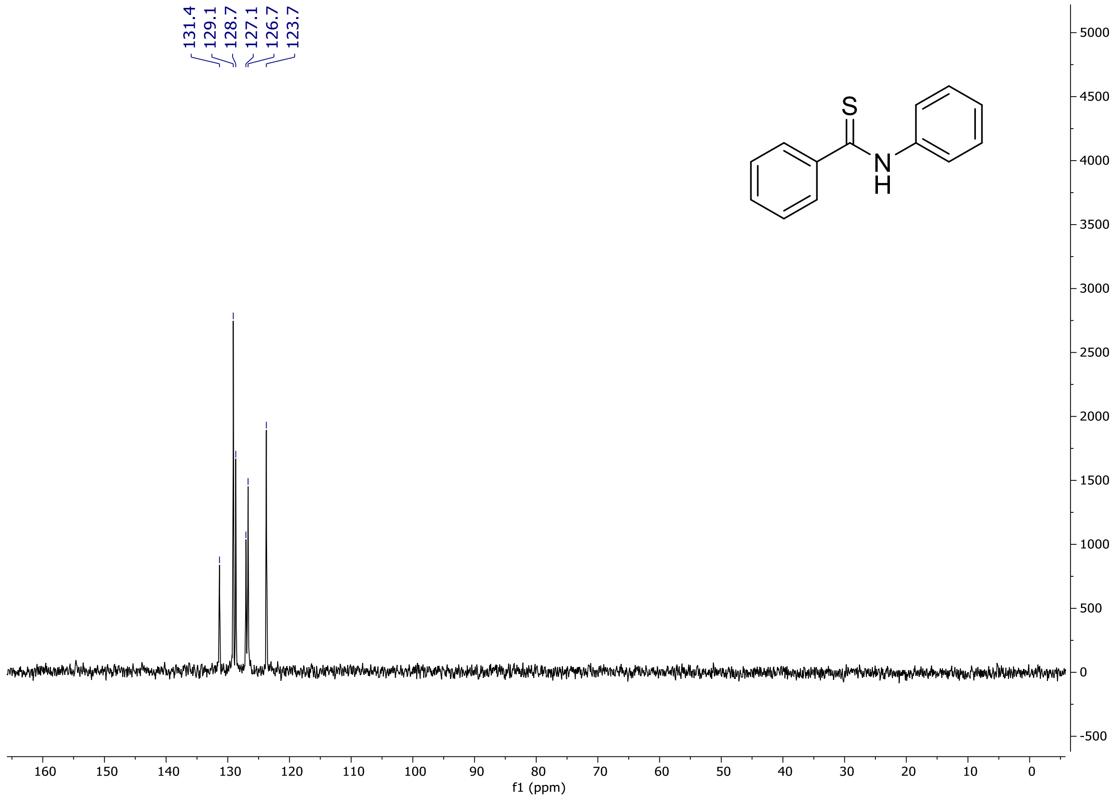

Figure SI_125: DEPT 135-NMR for **3b** in $CDCl_3$ (75 MHz).

4-Fluoro-*N*-phenylbenzothioamide (3c)

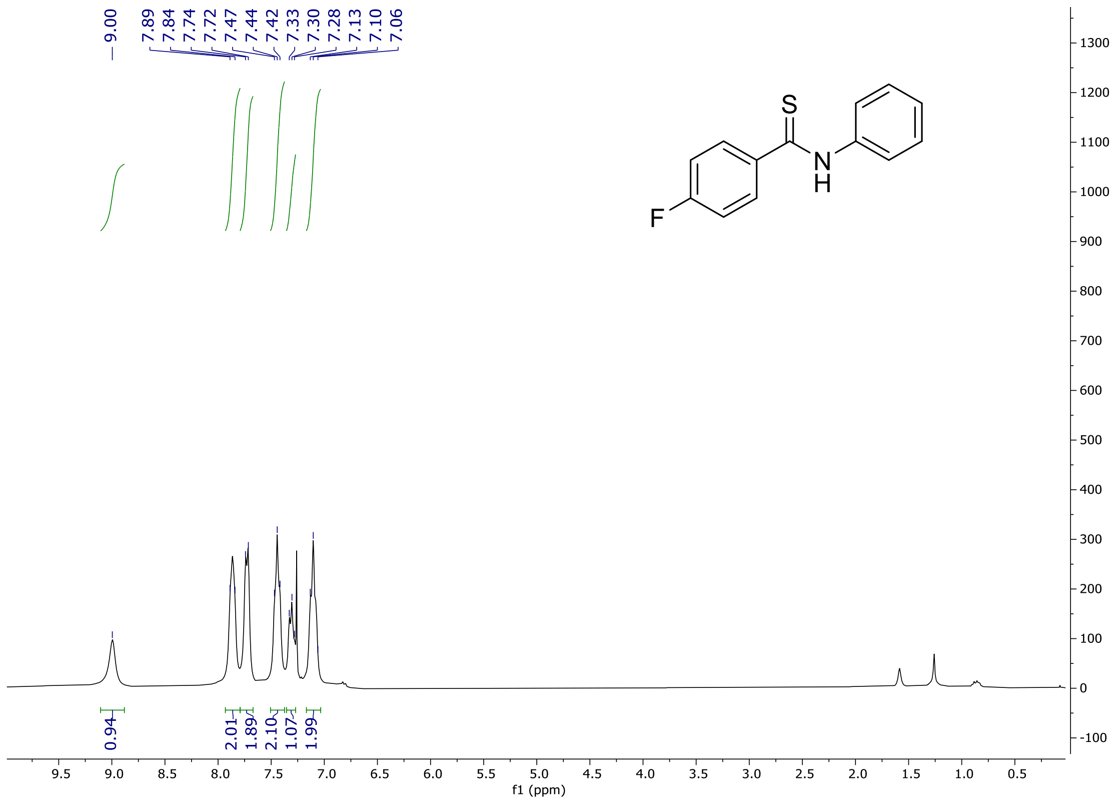

Figure SI_126: ^{1}H-NMR for **3c** in $CDCl_3$ (300 MHz).

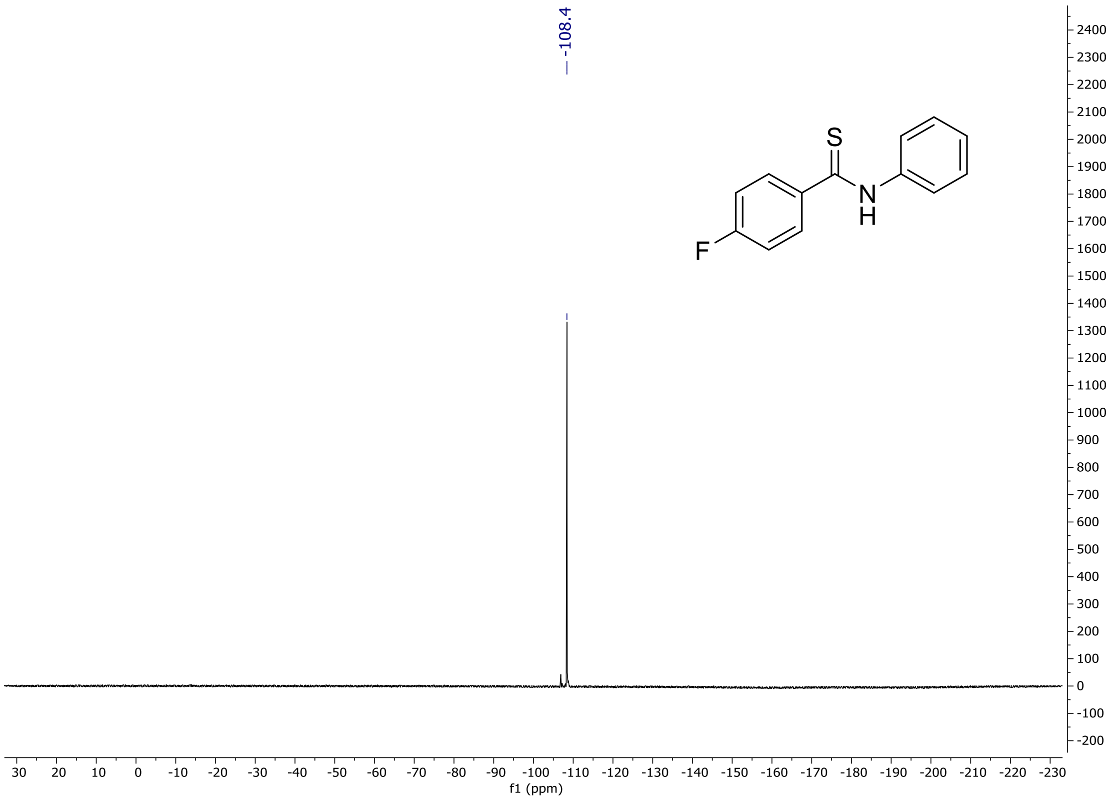

Figure SI_127: ^{19}F-NMR for **3c** in $CDCl_3$ (282 MHz).

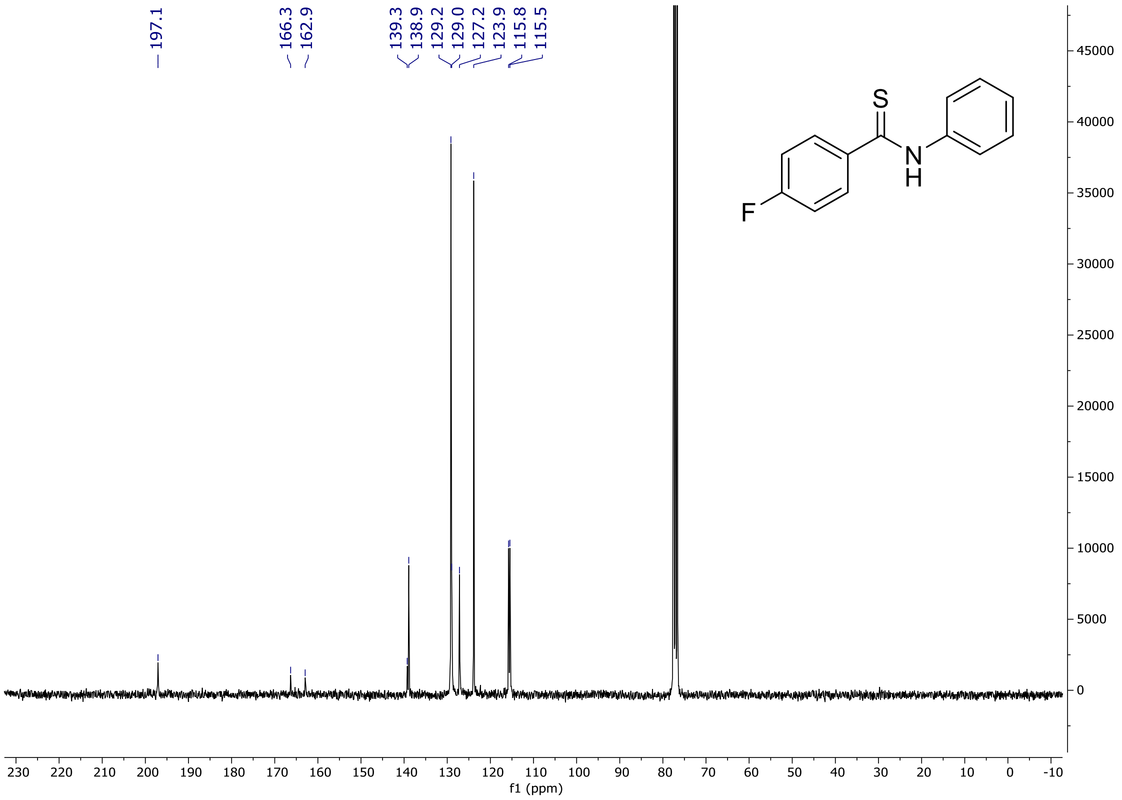

Figure SI_128: ^{13}C-NMR for **3c** in $CDCl_3$ (75 MHz).

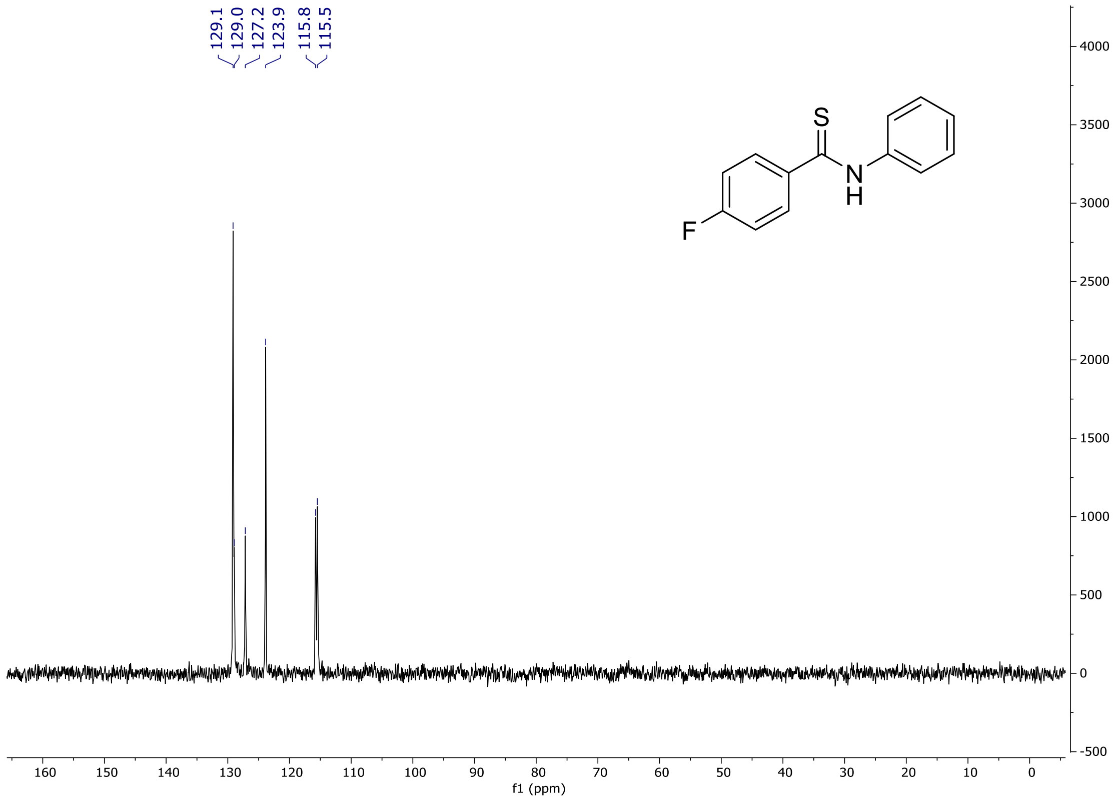

Figure SI_129: DEPT 135-NMR for **3c** in $CDCl_3$ (75 MHz).

4-Bromo-*N*-phenylbenzothioamide (3d)

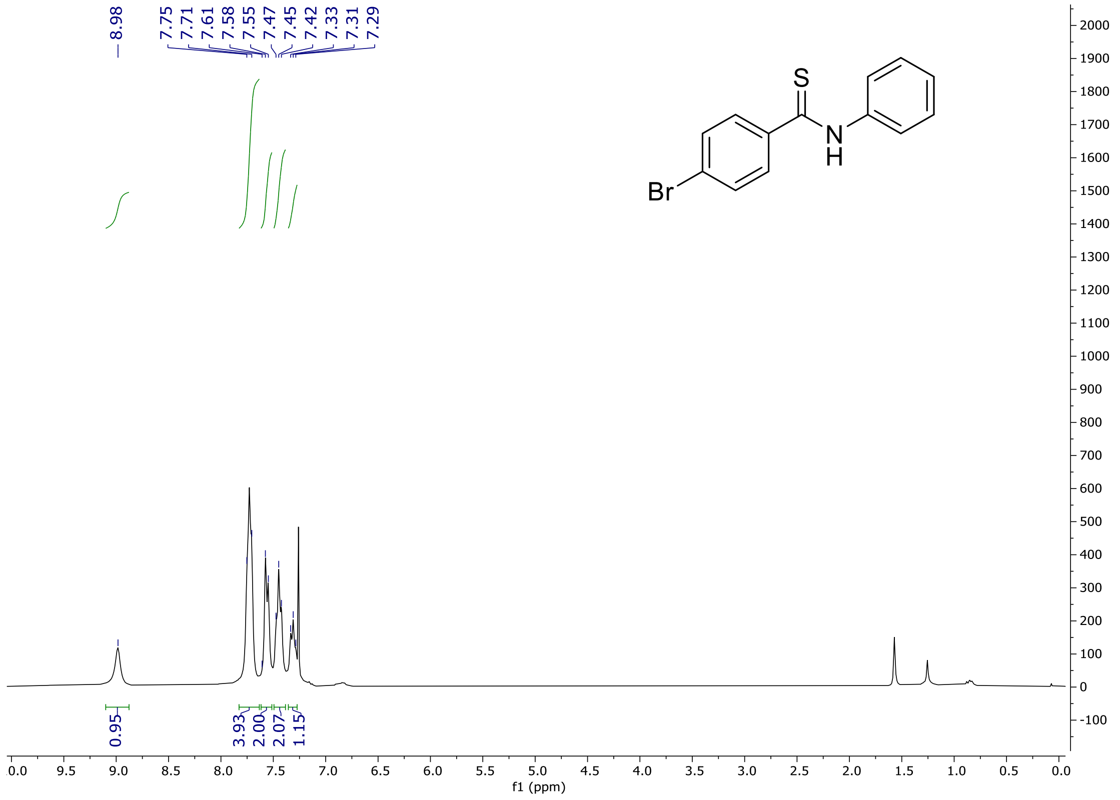

Figure SI_130: ^1H-NMR for **3d** in $CDCl_3$ (300 MHz).

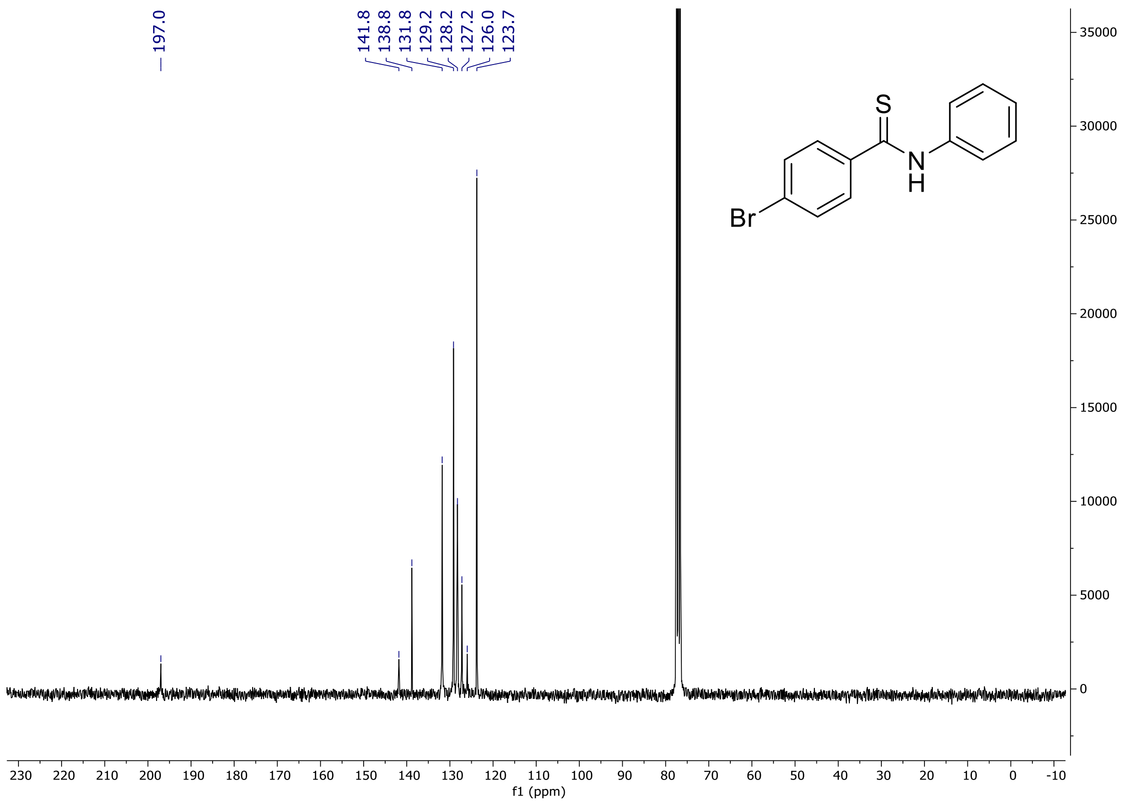

Figure SI_131: ^{13}C-NMR for **3d** in $CDCl_3$ (75 MHz).

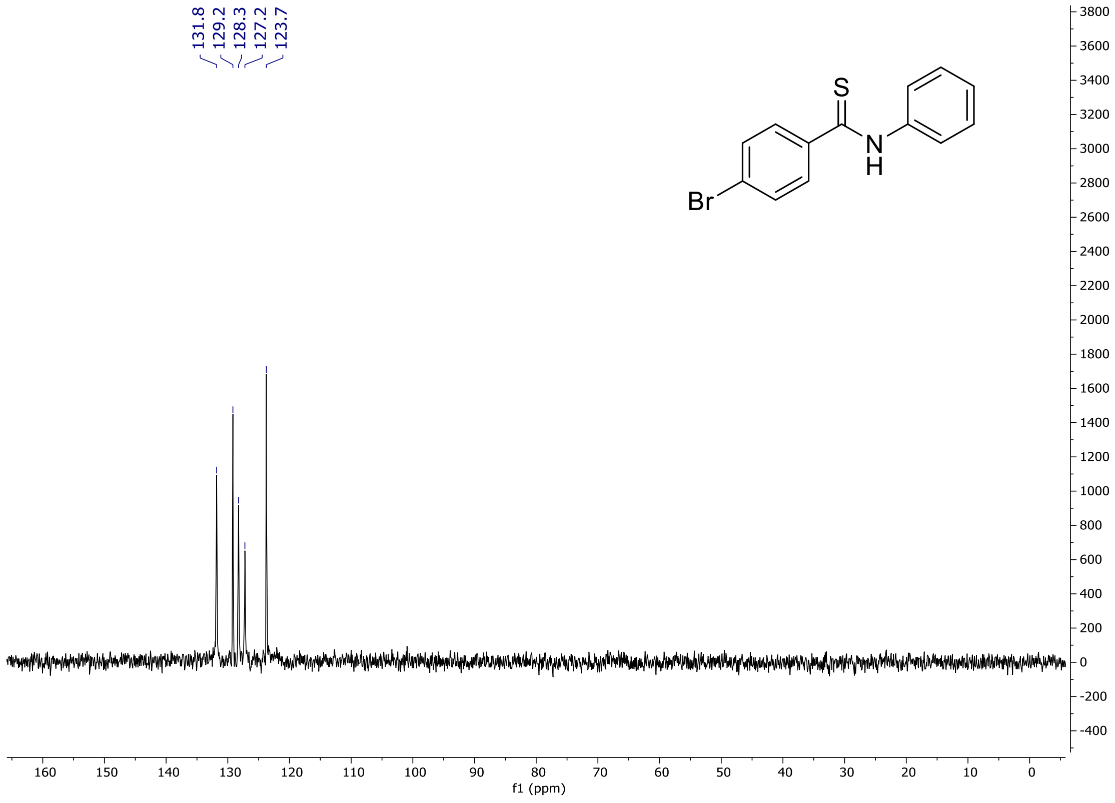

Figure SI_132: DEPT 135-NMR for **3d** in $CDCl_3$ (75 MHz).

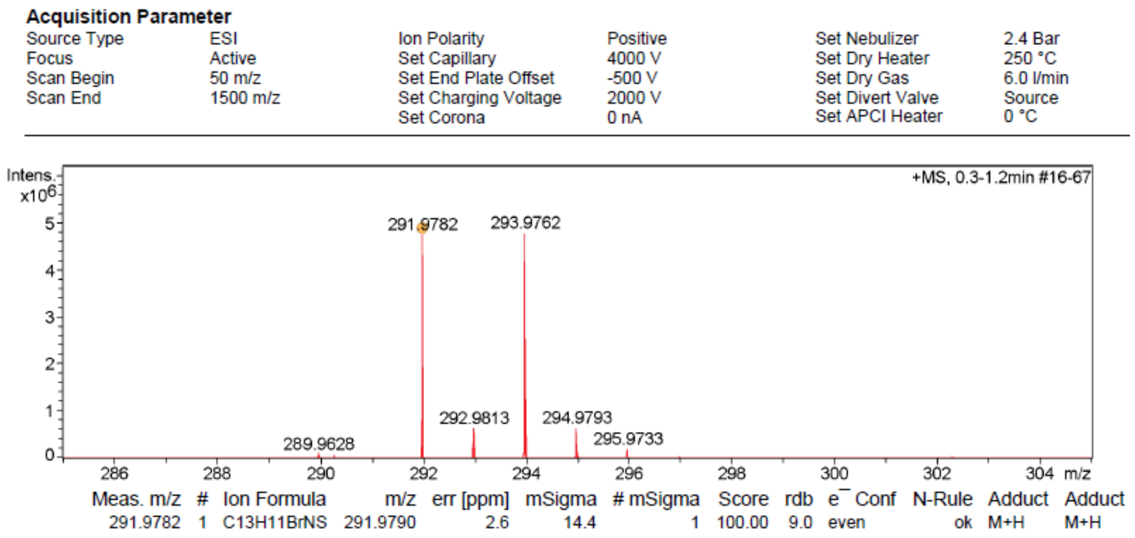

Figure SI_133: HRMS (ESI$^+$, m/z) analysis of **3d**.

4-Iodo-*N*-phenylbenzothioamide (3e)

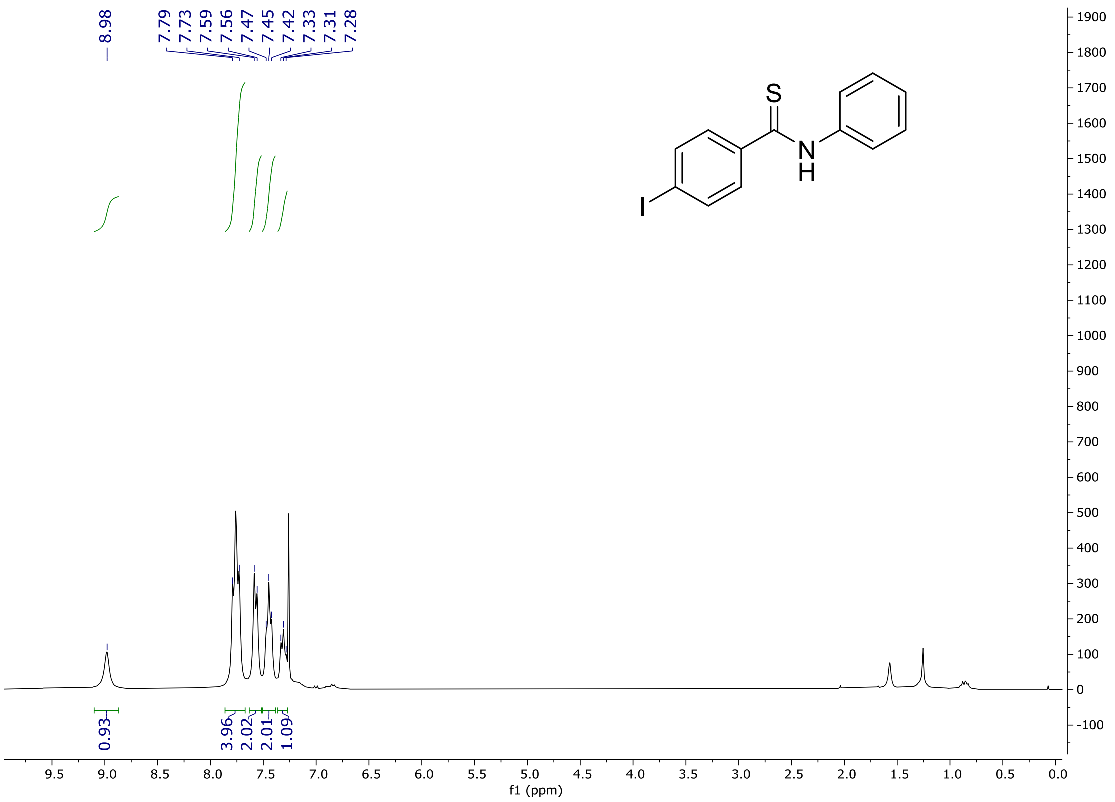

Figure SI_134: ^{1}H-NMR for **3e** in $CDCl_3$ (300 MHz).

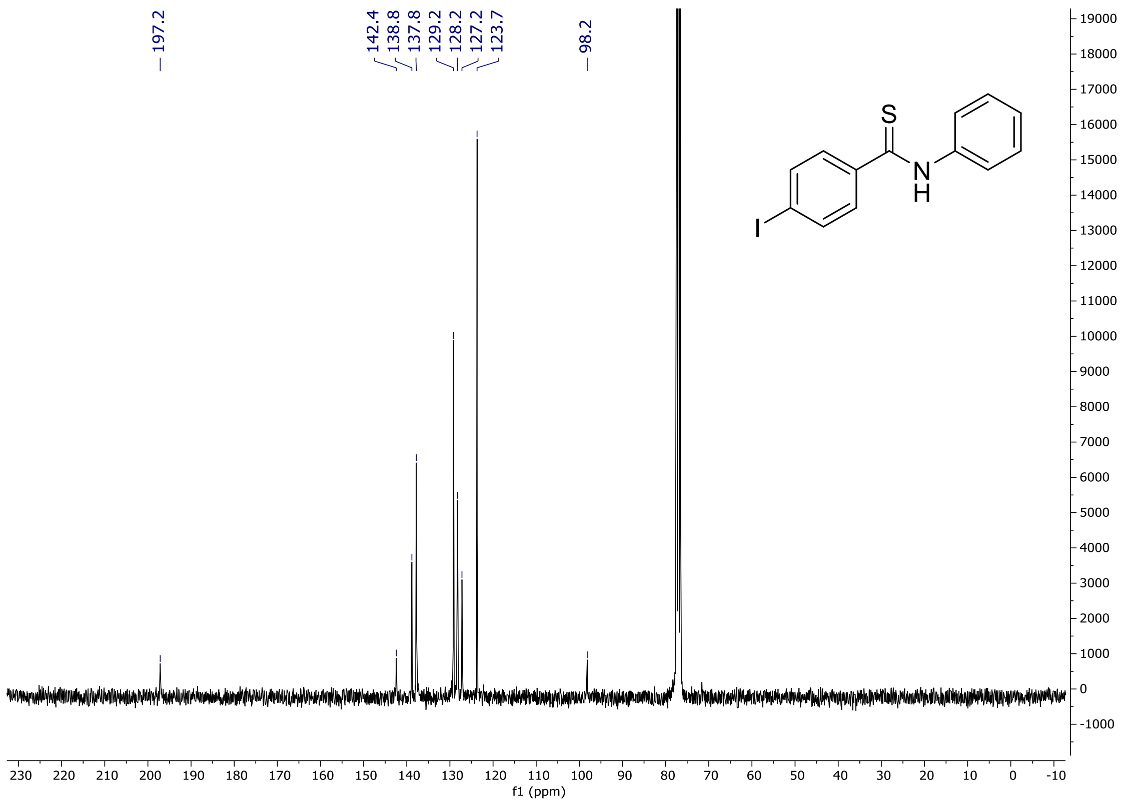

Figure SI_135: ^{13}C-NMR for **3e** in $CDCl_3$ (75 MHz).

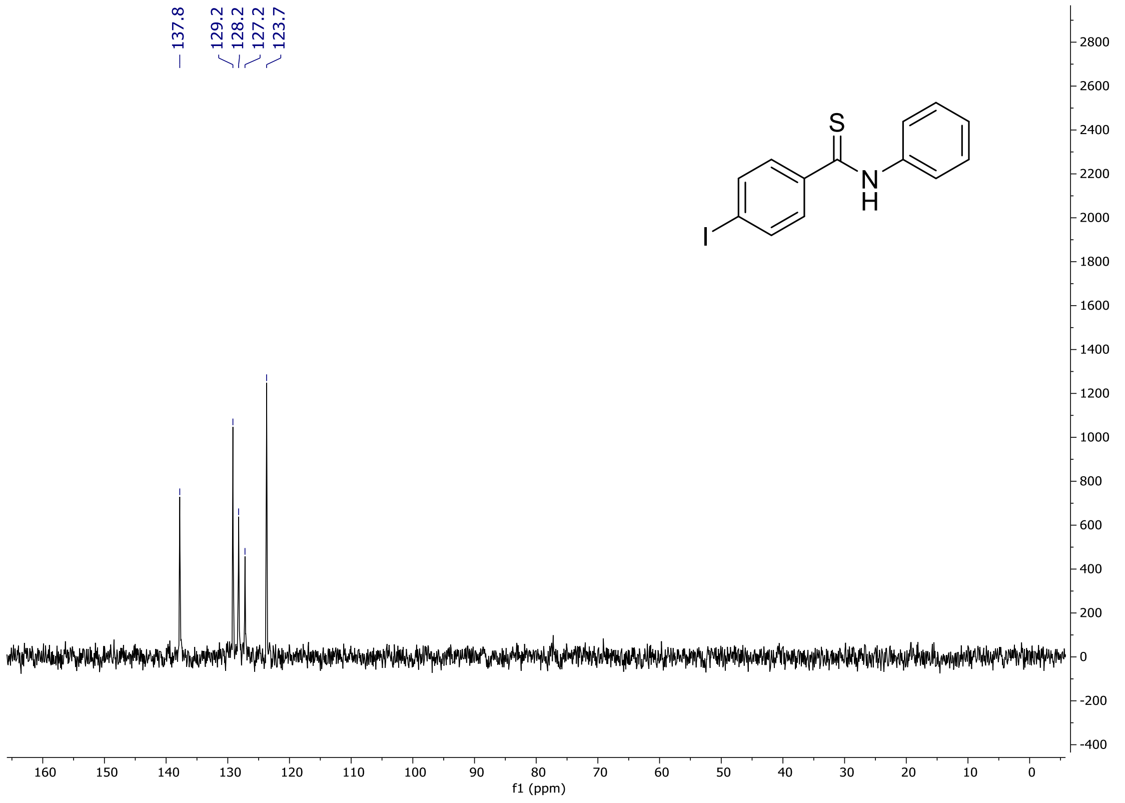

Figure SI_136: DEPT 135-NMR for **3e** in $CDCl_3$ (75 MHz).

Acquisition Parameter

Source Type	ESI	Ion Polarity	Positive	Set Nebulizer	2.4 Bar
Focus	Active	Set Capillary	4000 V	Set Dry Heater	250 °C
Scan Begin	50 m/z	Set End Plate Offset	-500 V	Set Dry Gas	6.0 l/min
Scan End	1500 m/z	Set Charging Voltage	2000 V	Set Divert Valve	Source
		Set Corona	0 nA	Set APCI Heater	0 °C

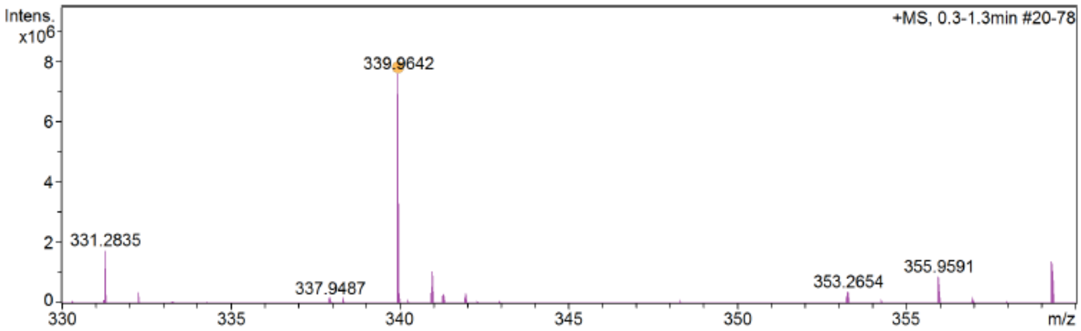

Meas. m/z	#	Ion Formula	m/z	err [ppm]	mSigma	# mSigma	Score	rdb	e⁻ Conf	N-Rule	Adduct
339.9642	1	C13H11INS	339.9651	2.8	9.1	1	100.00	9.0	even	ok	M+H

Figure SI_137: HRMS (ESI$^+$, m/z) analysis of **3e**.

3-Chloro-*N*-phenylbenzothioamide (3f)

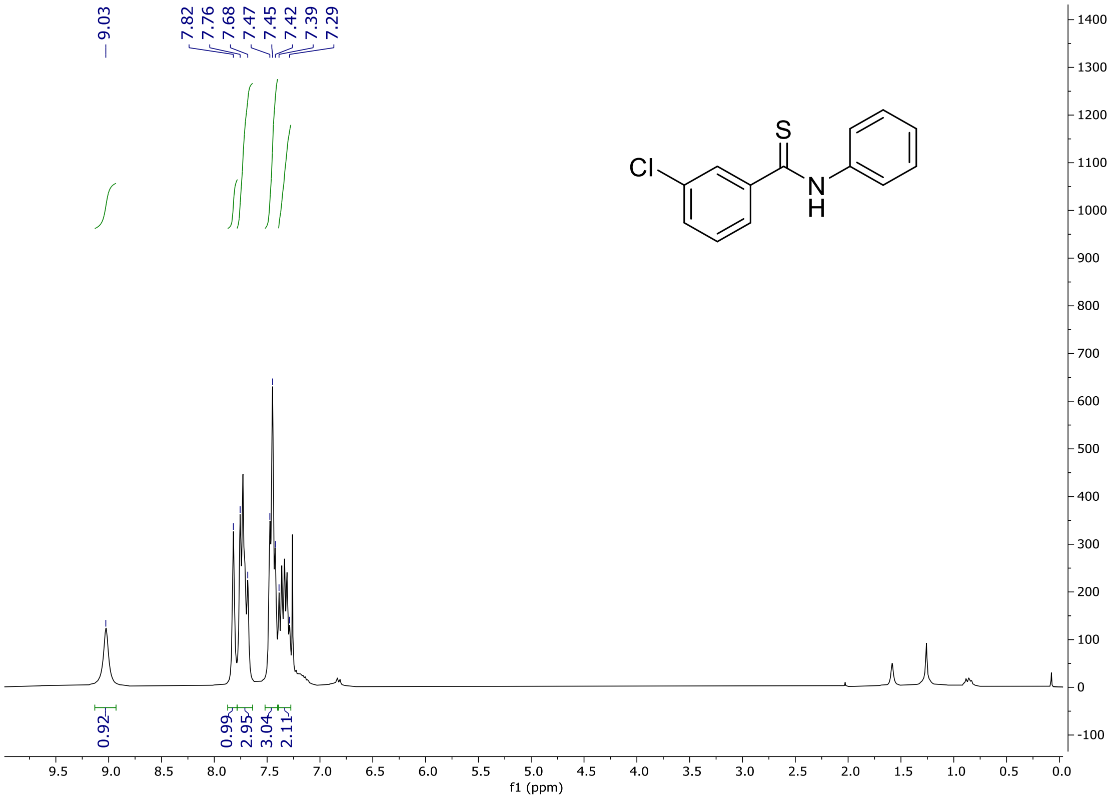

Figure SI_138: ^{1}H-NMR for **3f** in $CDCl_3$ (300 MHz).

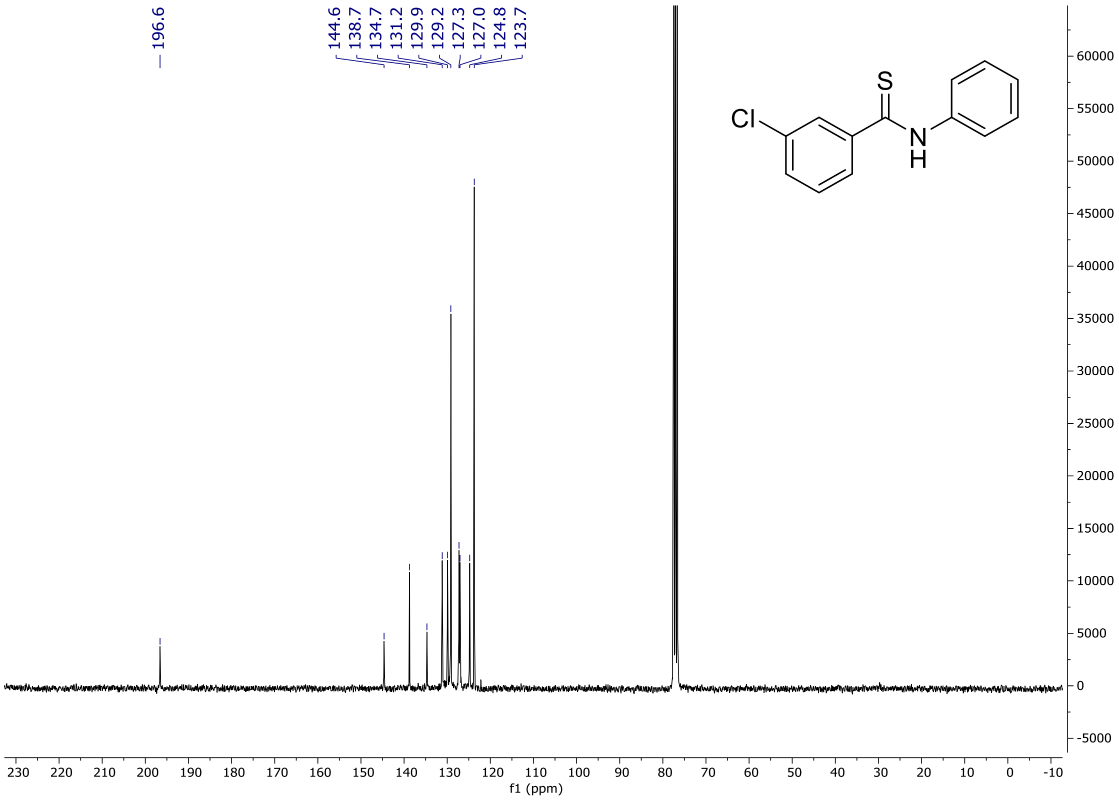

Figure SI_139: ^{13}C-NMR for **3f** in $CDCl_3$ (75 MHz).

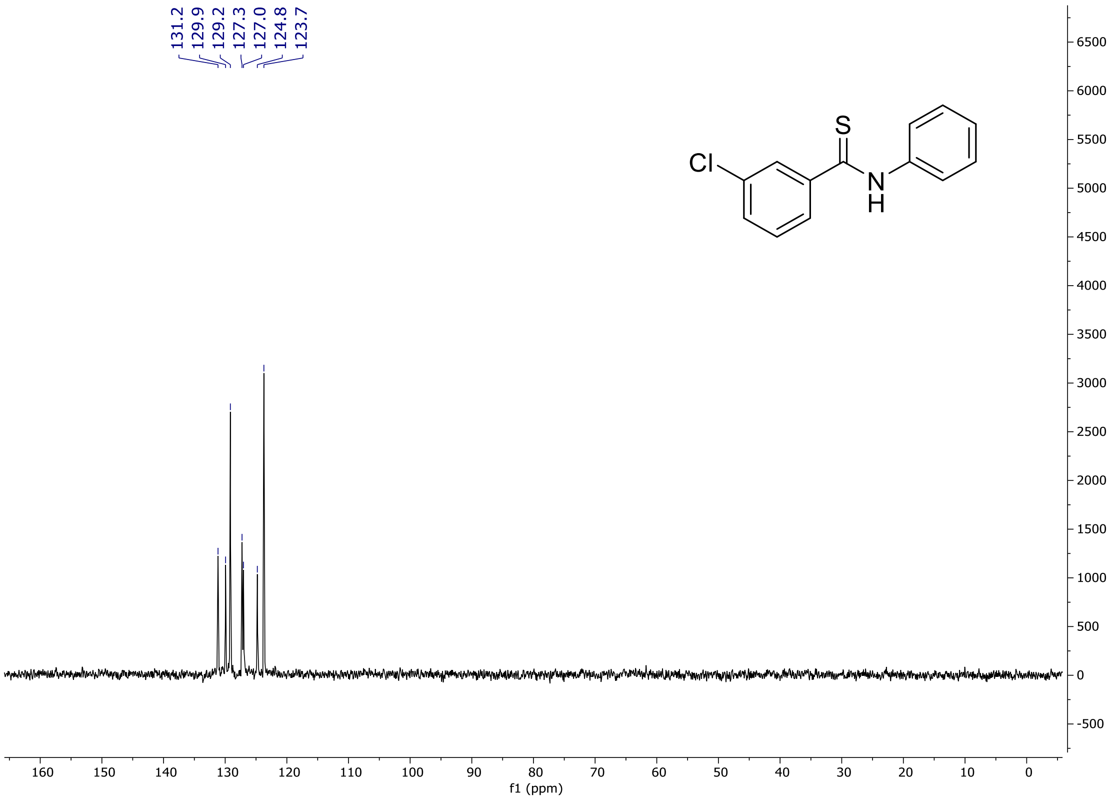

Figure SI_140: DEPT 135-NMR for **3f** in $CDCl_3$ (75 MHz).

2-Chloro-*N*-phenylbenzothioamide (3g)

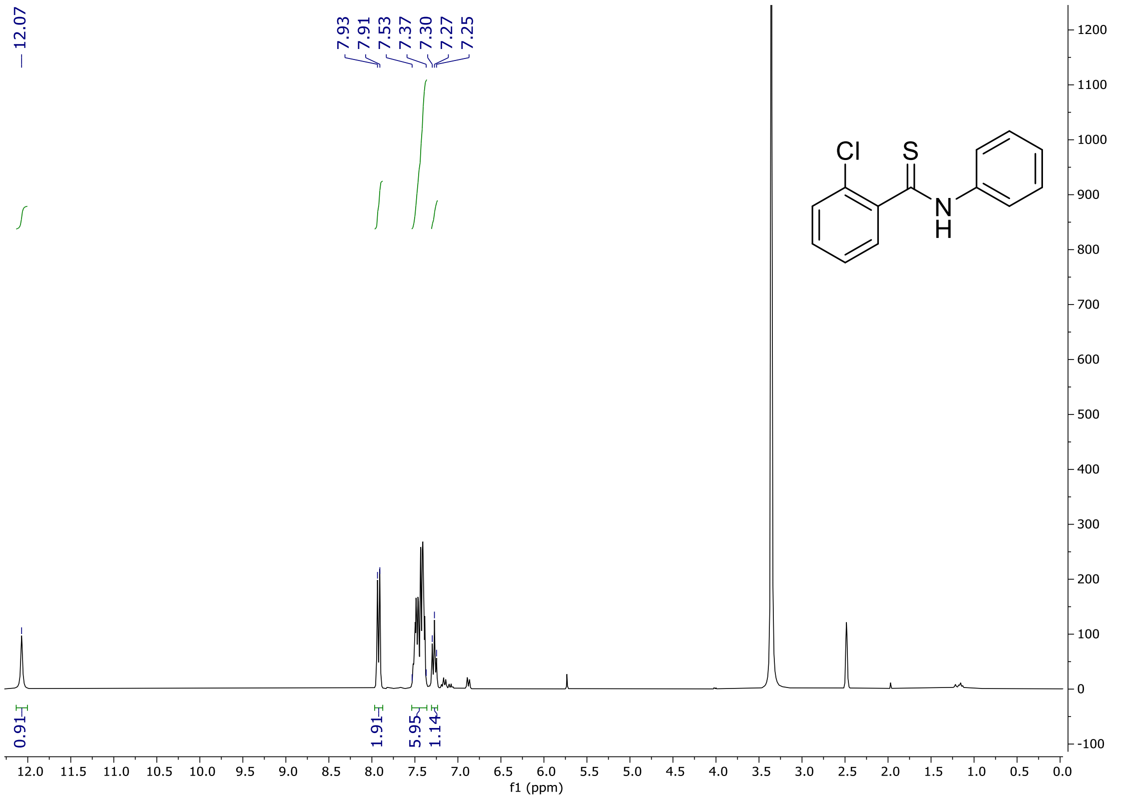

Figure SI_141: ^{1}H-NMR for **3g** in DMSO-d^{6} (300 MHz).

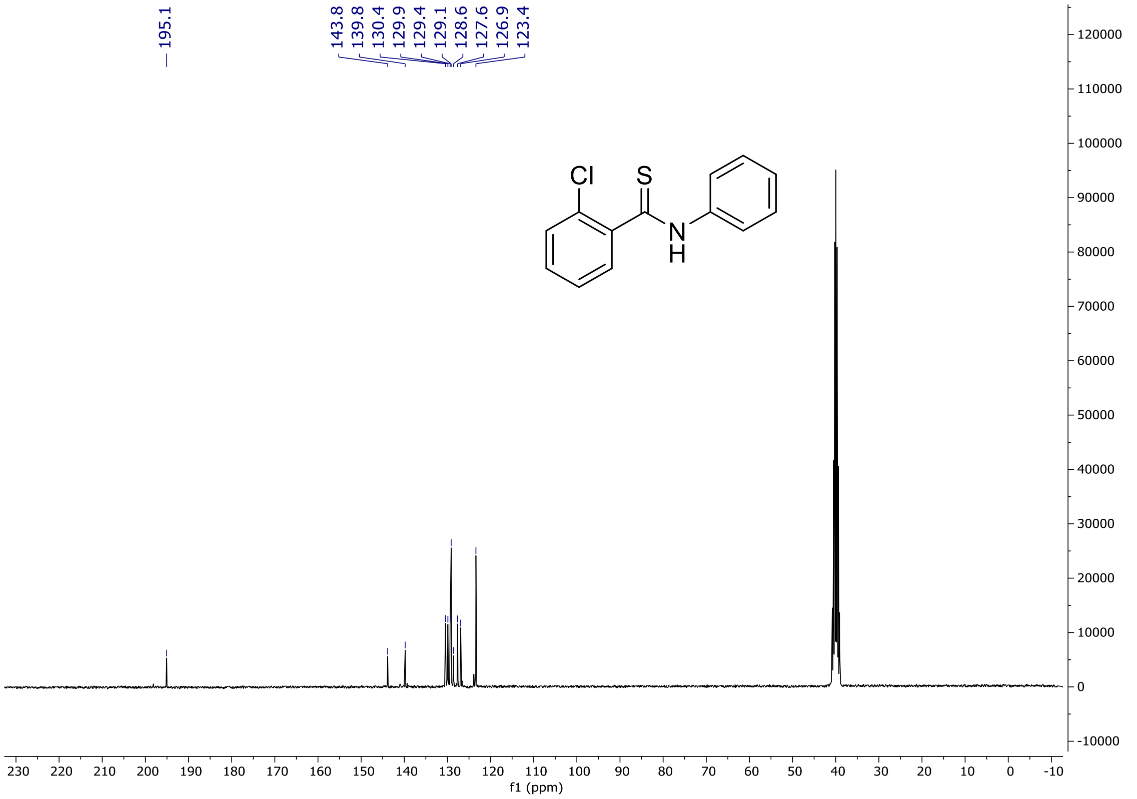

Figure SI_142: ^{13}C-NMR for **3g** in DMSO-d^{6} (75 MHz).

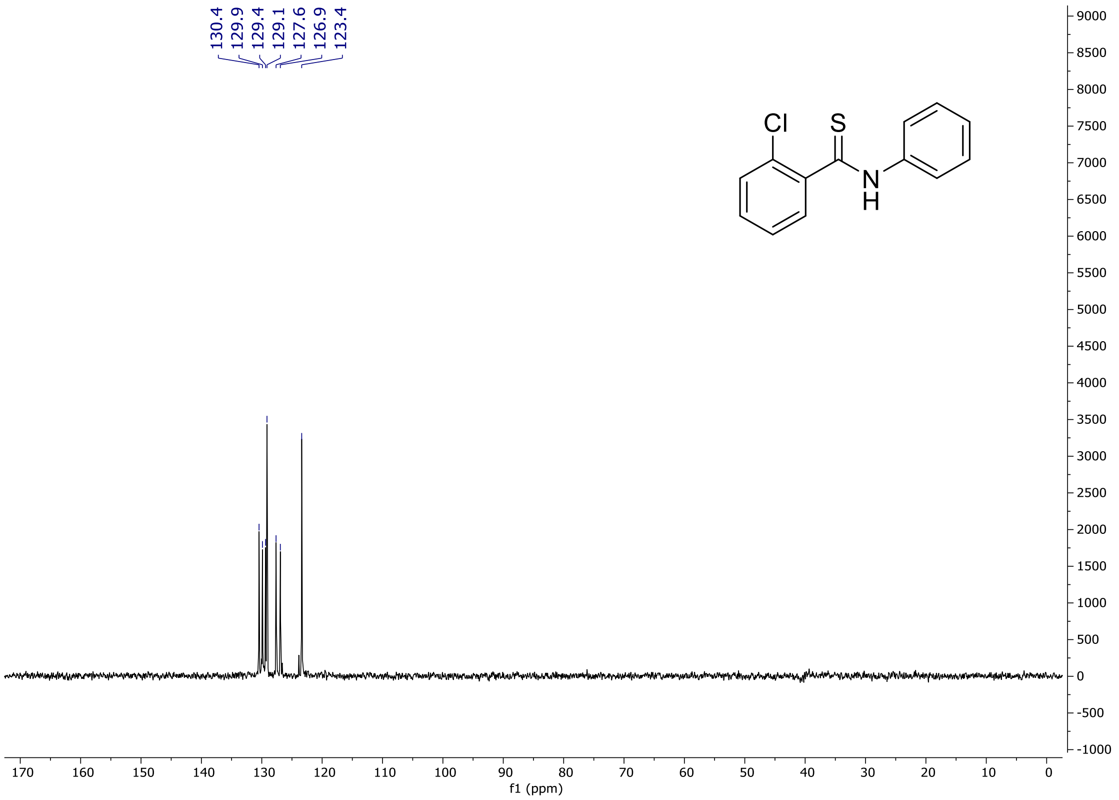

Figure SI_143: DEPT 135-NMR for **3g** in DMSO-d^6 (75 MHz).

Acquisition Parameter

Source Type	ESI	Ion Polarity	Positive	Set Nebulizer	2.4 Bar
Focus	Active	Set Capillary	4000 V	Set Dry Heater	250 °C
Scan Begin	50 m/z	Set End Plate Offset	-500 V	Set Dry Gas	6.0 l/min
Scan End	1500 m/z	Set Charging Voltage	2000 V	Set Divert Valve	Source
		Set Corona	0 nA	Set APCI Heater	0 °C

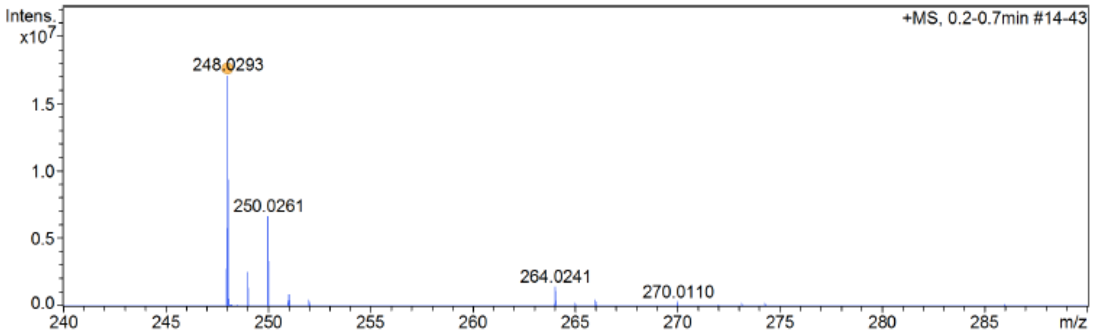

Meas. m/z	#	Ion Formula	m/z	err [ppm]	mSigma	# mSigma	Score	rdb	e⁻ Conf	N-Rule	Adduct
248.0293	1	C13H11ClNS	248.0295	1.0	7.2	1	100.00	9.0	even	ok	M+H

Figure SI_144: HRMS (ESI$^+$, m/z) analysis of **3g**.

3,4-Dichloro-*N*-phenylbenzothioamide (3h)

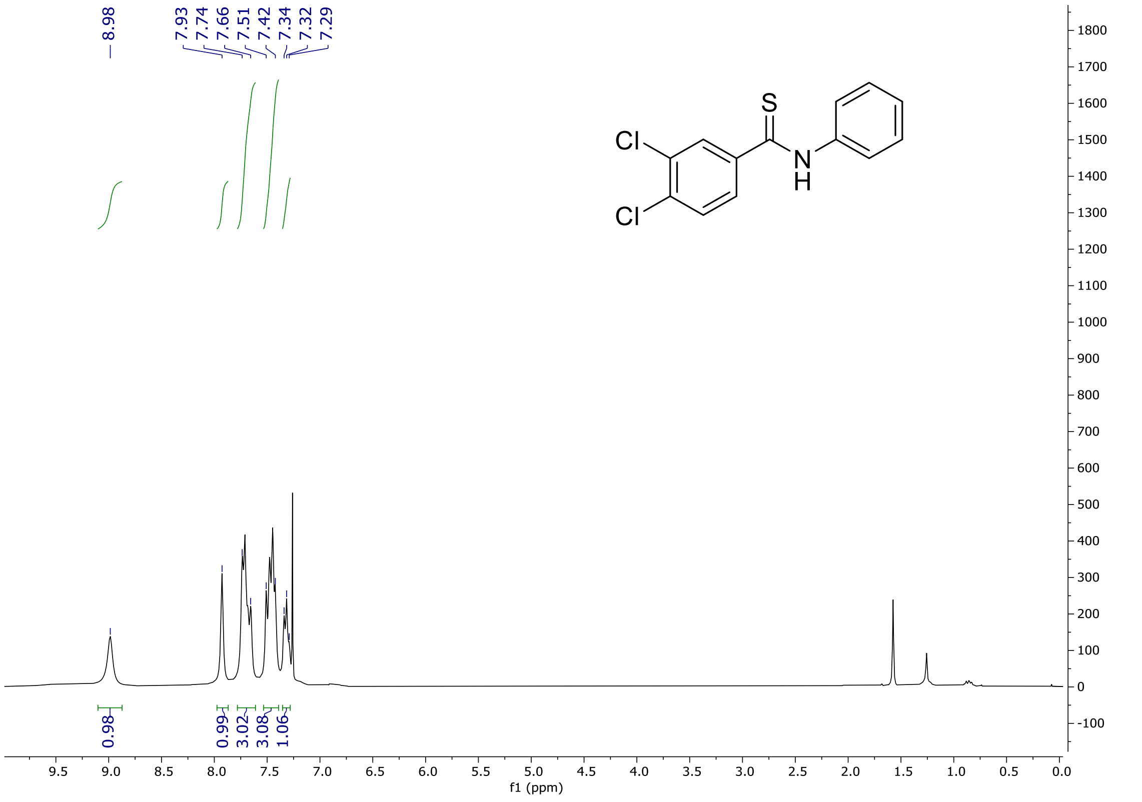

Figure SI_145: ^{1}H-NMR for **3h** in $CDCl_3$ (300 MHz).

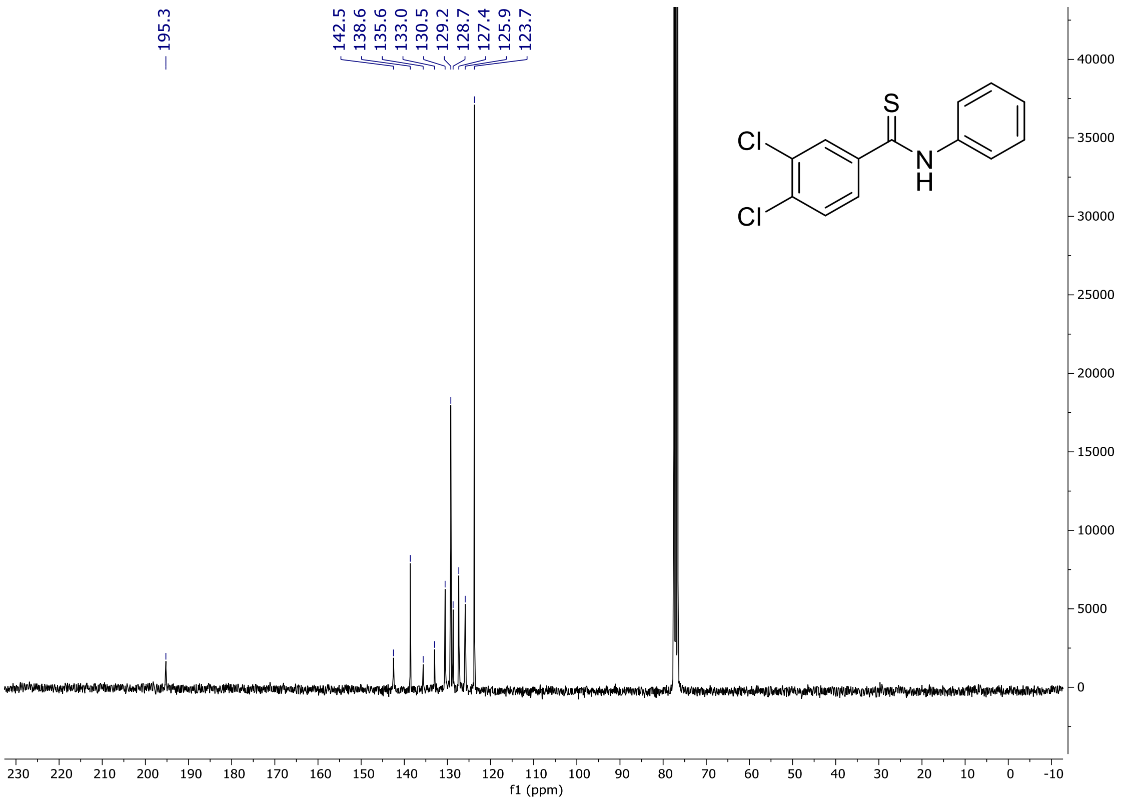

Figure SI_146: ^{13}C-NMR for **3h** in $CDCl_3$ (75 MHz).

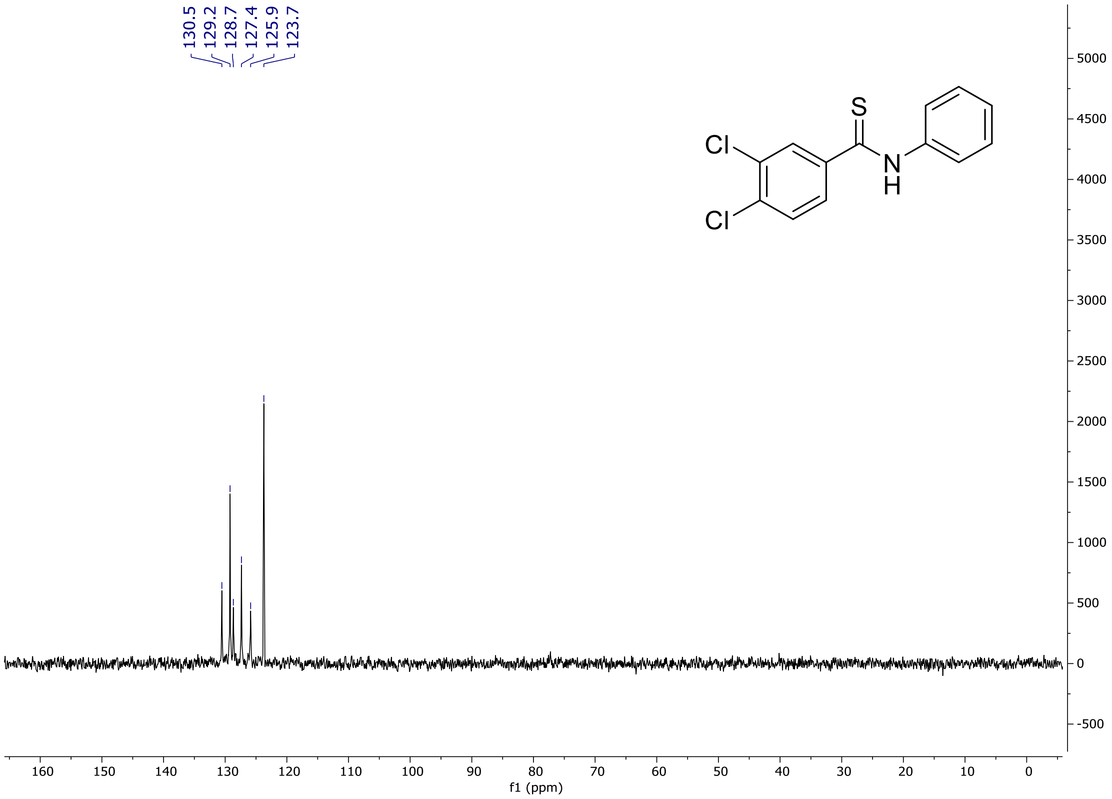

Figure SI_147: DEPT 135-NMR for **3h** in $CDCl_3$ (75 MHz).

Acquisition Parameter

Source Type	ESI	Ion Polarity	Positive	Set Nebulizer	2.4 Bar
Focus	Active	Set Capillary	4000 V	Set Dry Heater	250 °C
Scan Begin	50 m/z	Set End Plate Offset	-500 V	Set Dry Gas	6.0 l/min
Scan End	1500 m/z	Set Charging Voltage	2000 V	Set Divert Valve	Source
		Set Corona	0 nA	Set APCI Heater	0 °C

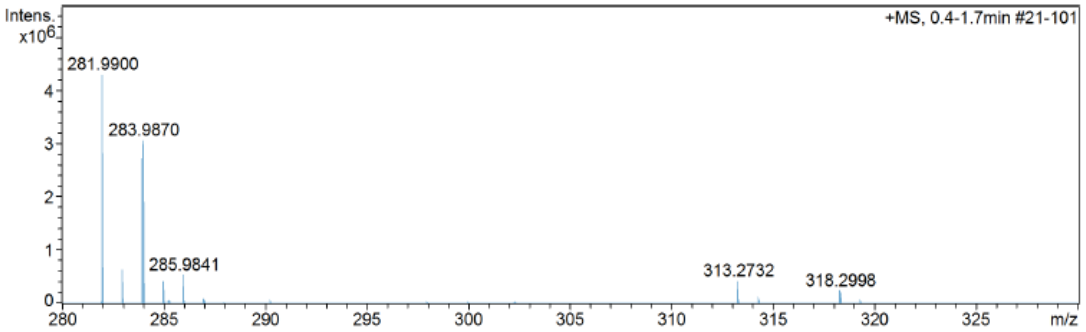

Meas. m/z	#	Ion Formula	m/z	err [ppm]	mSigma	# mSigma	Score	rdb	e⁻ Conf	N-Rule	Adduct
281.9900	1	C13H10Cl2NS	281.9906	2.0	8.7	1	100.00	9.0	even	ok	M+H

Figure SI_148: HRMS (ESI^+, m/z) analysis of **3h**.

_N_-Phenyl-4-(trifluoromethyl)benzothioamide (3i)

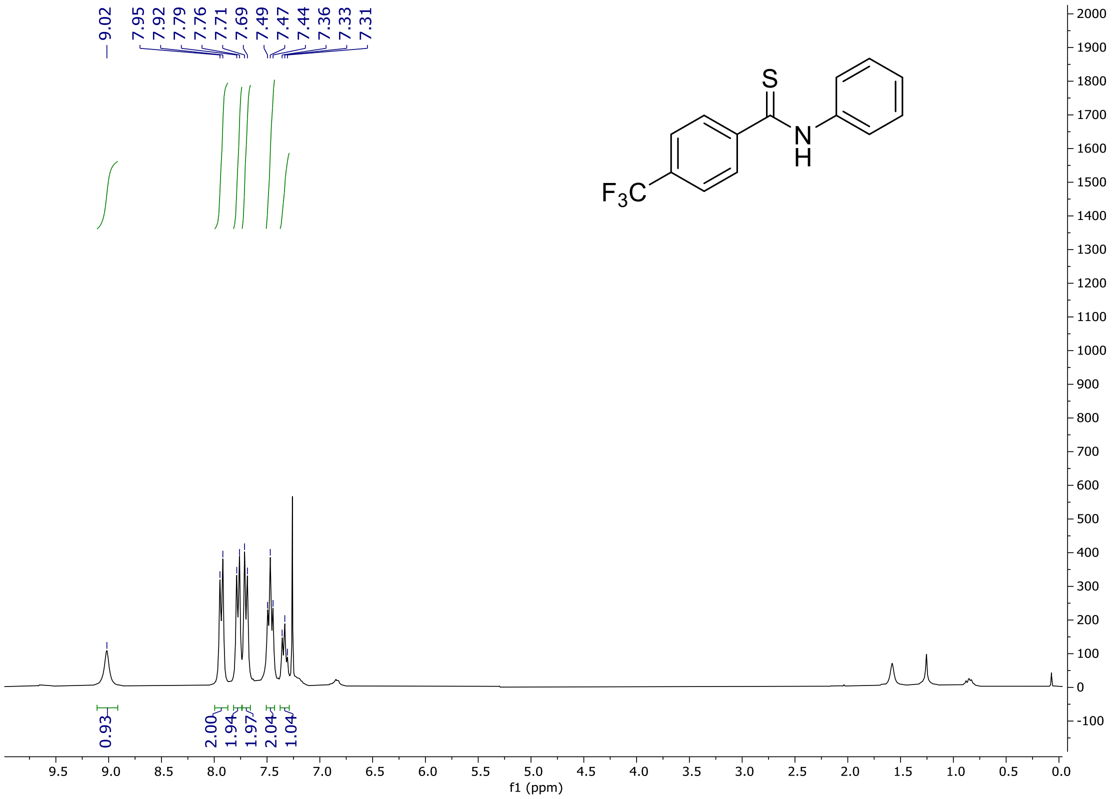

Figure SI_149: ^{1}H-NMR for **3i** in $CDCl_3$ (300 MHz).

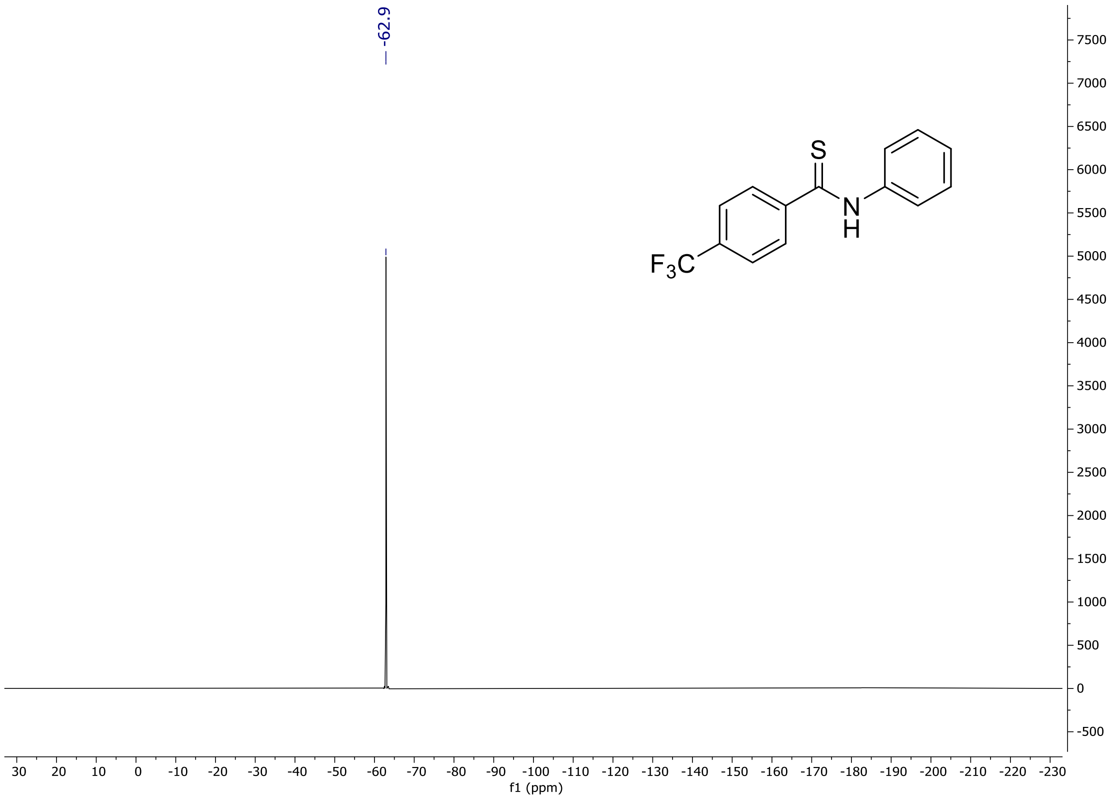

Figure SI_150: ^{19}F-NMR for **3i** in $CDCl_3$ (282 MHz).

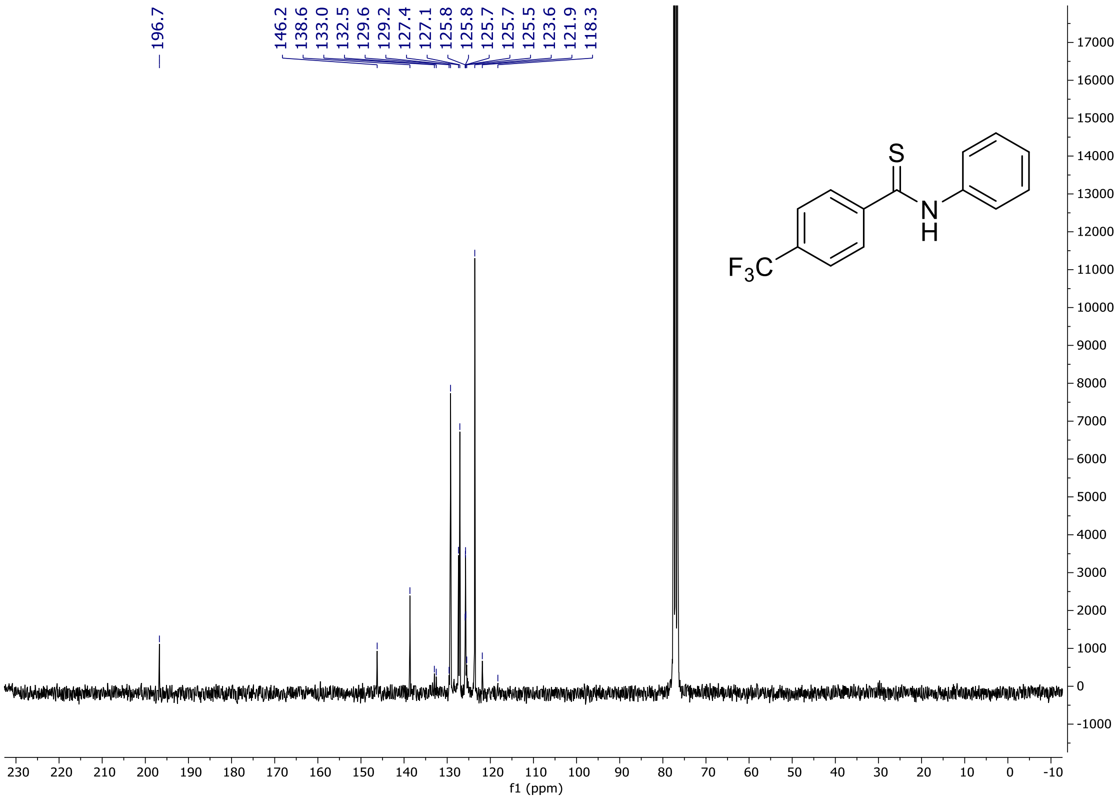

Figure SI_151: ^{13}C-NMR for **3i** in $CDCl_3$ (75 MHz).

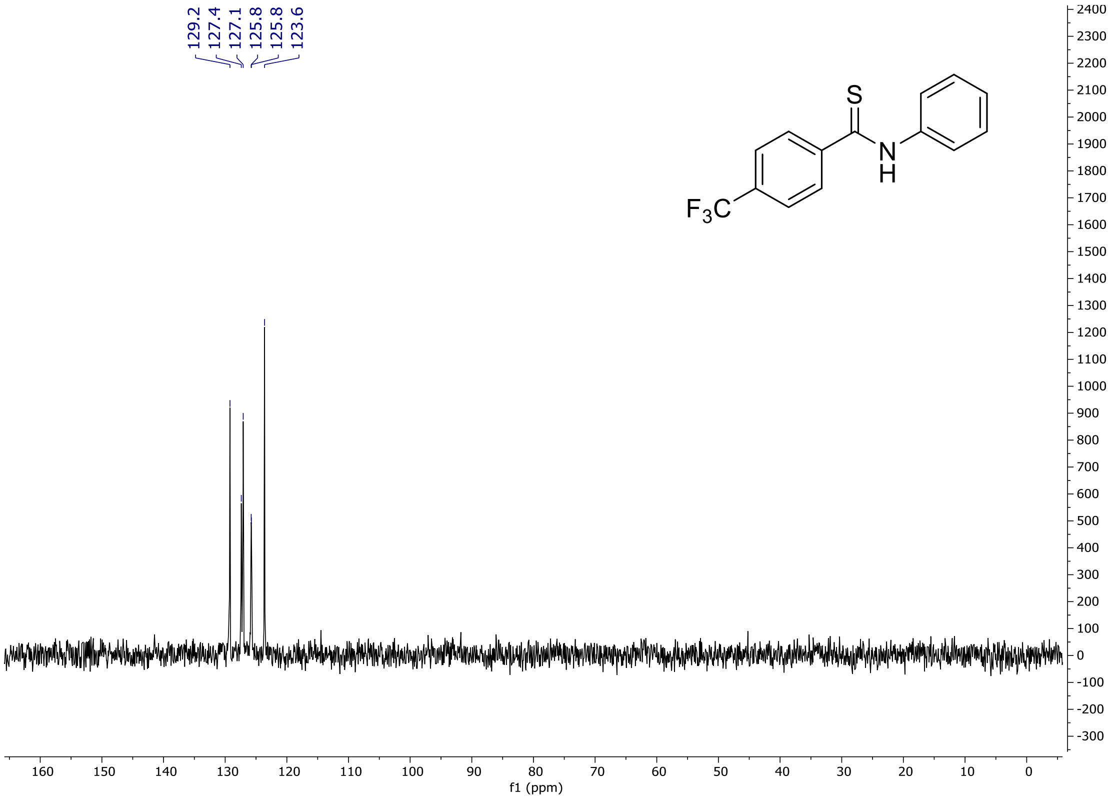

Figure SI_152: DEPT 135-NMR for **3i** in $CDCl_3$ (75 MHz).

4-Nitro-*N*-phenylbenzothioamide (3j)

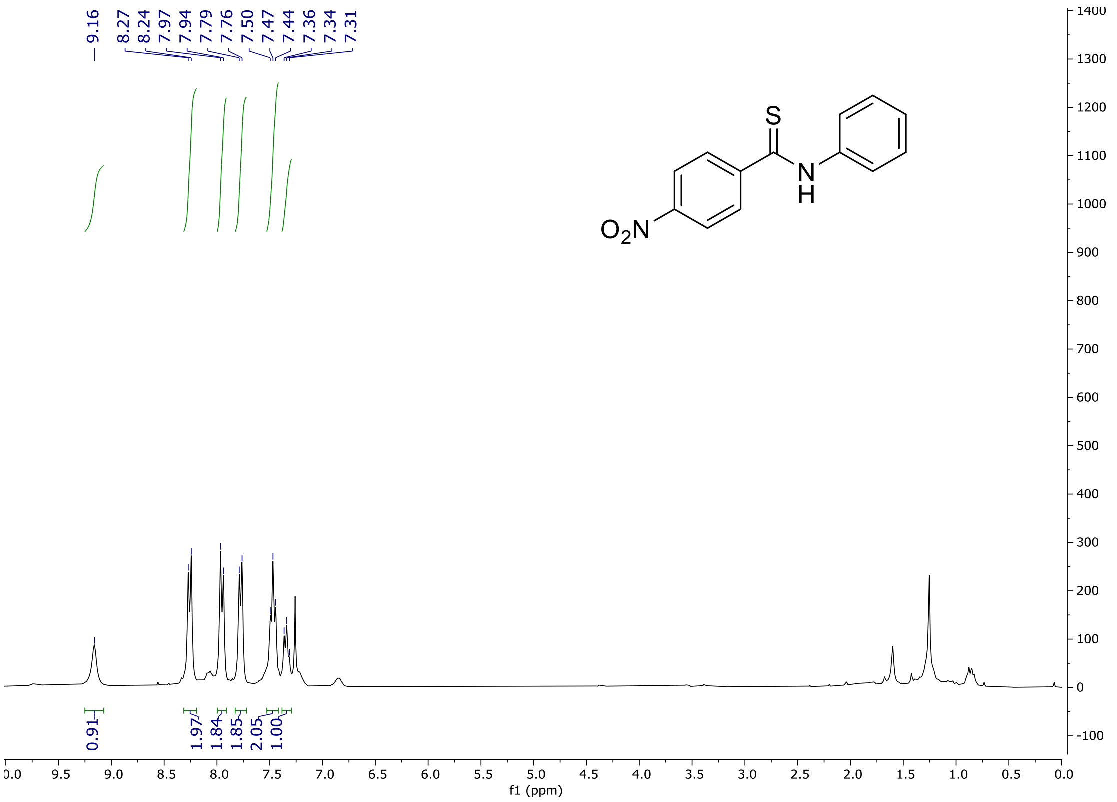

Figure SI_153: ^{1}H-NMR for **3j** in $CDCl_3$ (300 MHz).

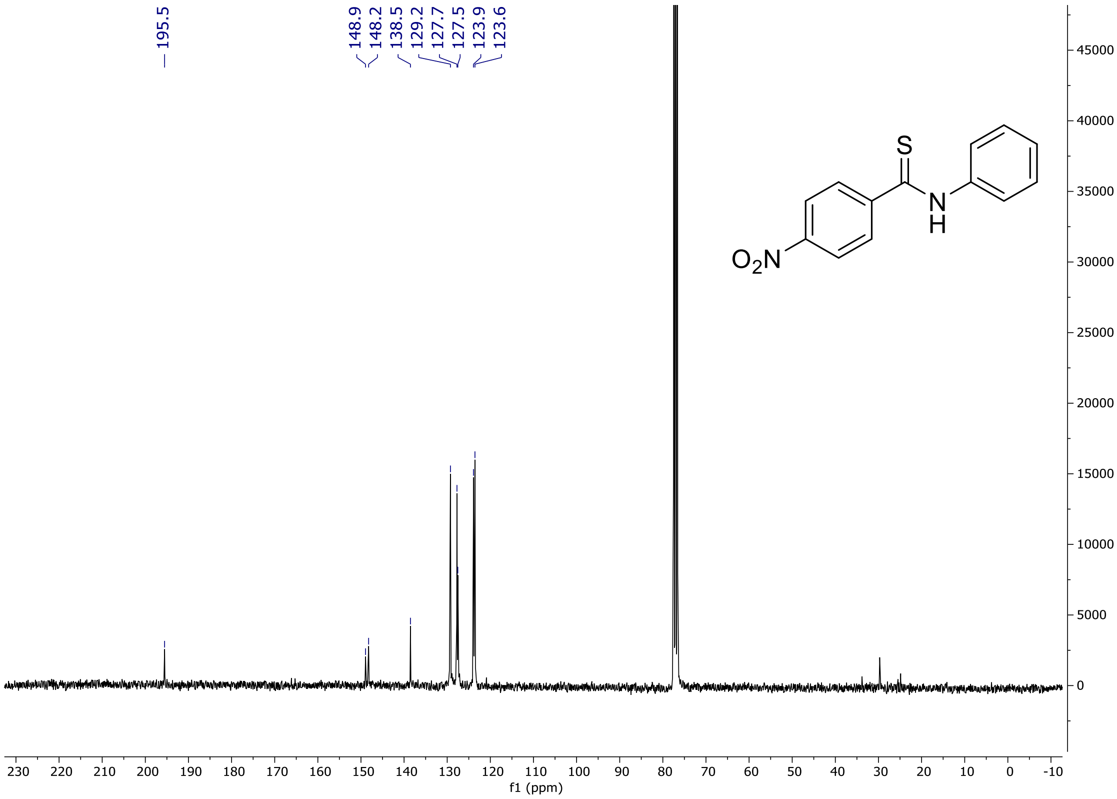

Figure SI_154: ^{13}C-NMR for **3j** in $CDCl_3$ (75 MHz).

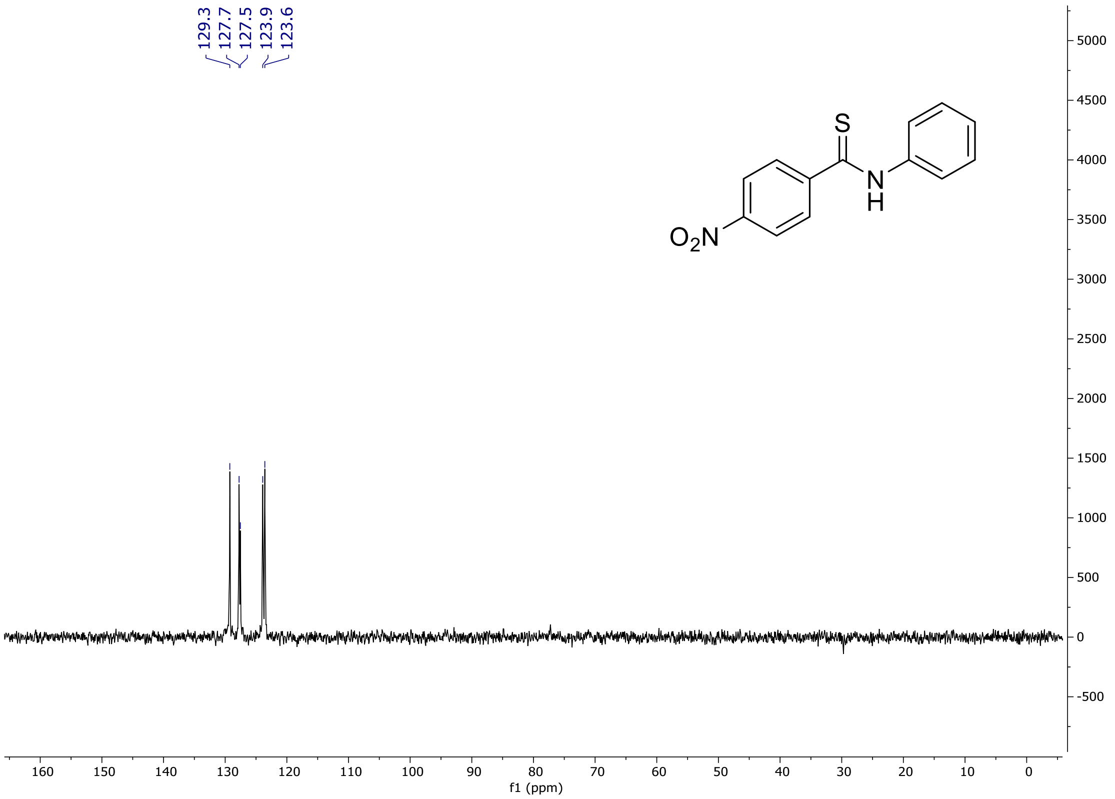

Figure SI_155: DEPT 135-NMR for **3j** in $CDCl_3$ (75 MHz).

4-Cyano-*N*-phenylbenzothioamide (3k)

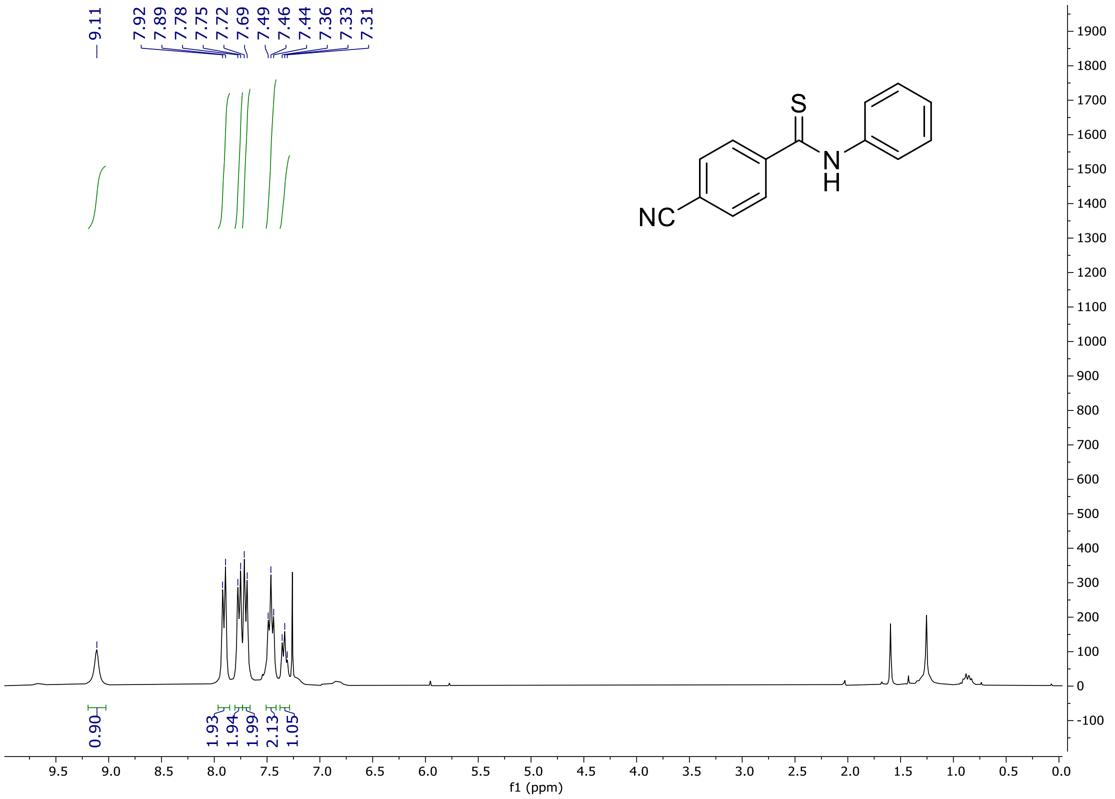

Figure SI_156: ^{1}H-NMR for **3k** in $CDCl_3$ (300 MHz).

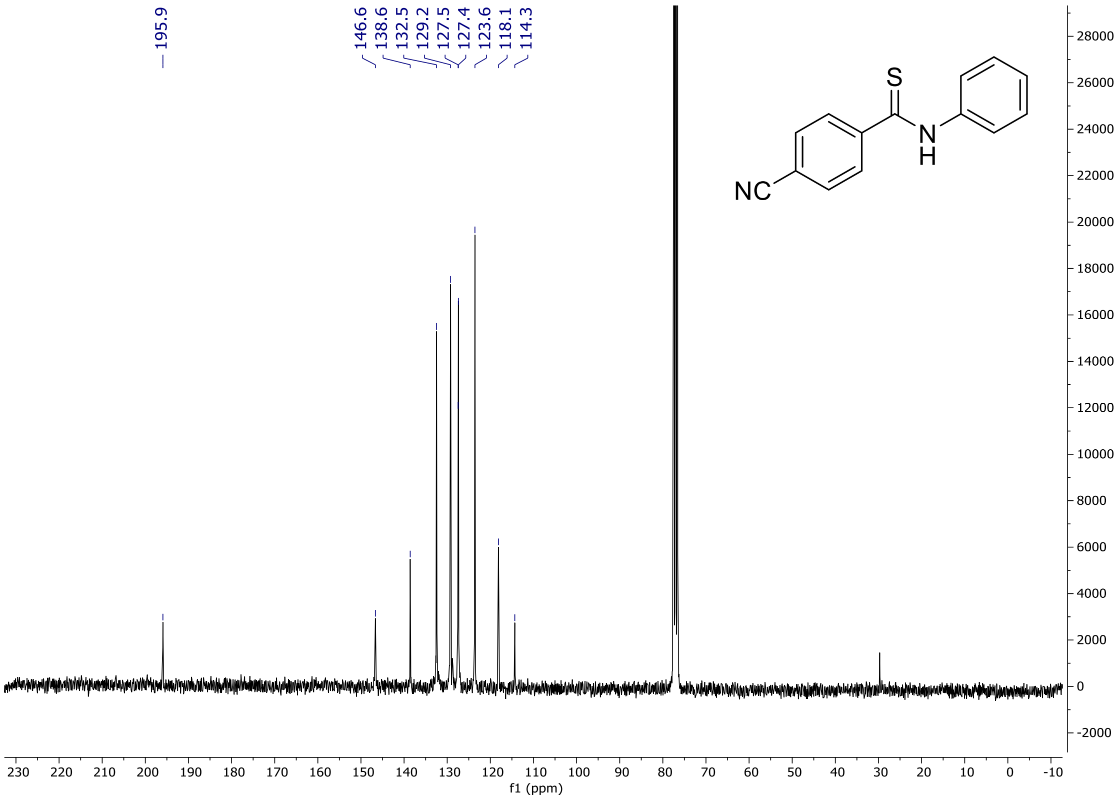

Figure SI_157: ^{13}C-NMR for **3k** in $CDCl_3$ (75 MHz).

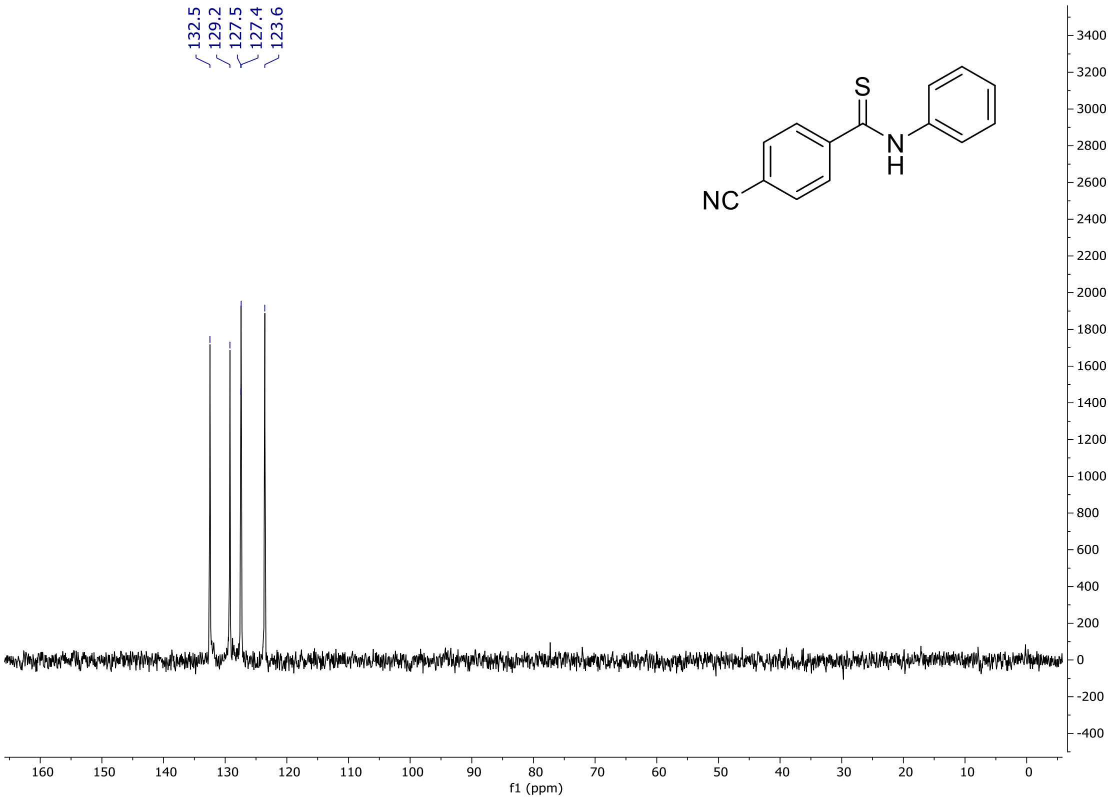

Figure SI_158: DEPT 135-NMR for **3k** in $CDCl_3$ (75 MHz).

4-Ethynyl-*N*-phenylbenzothioamide (3l)

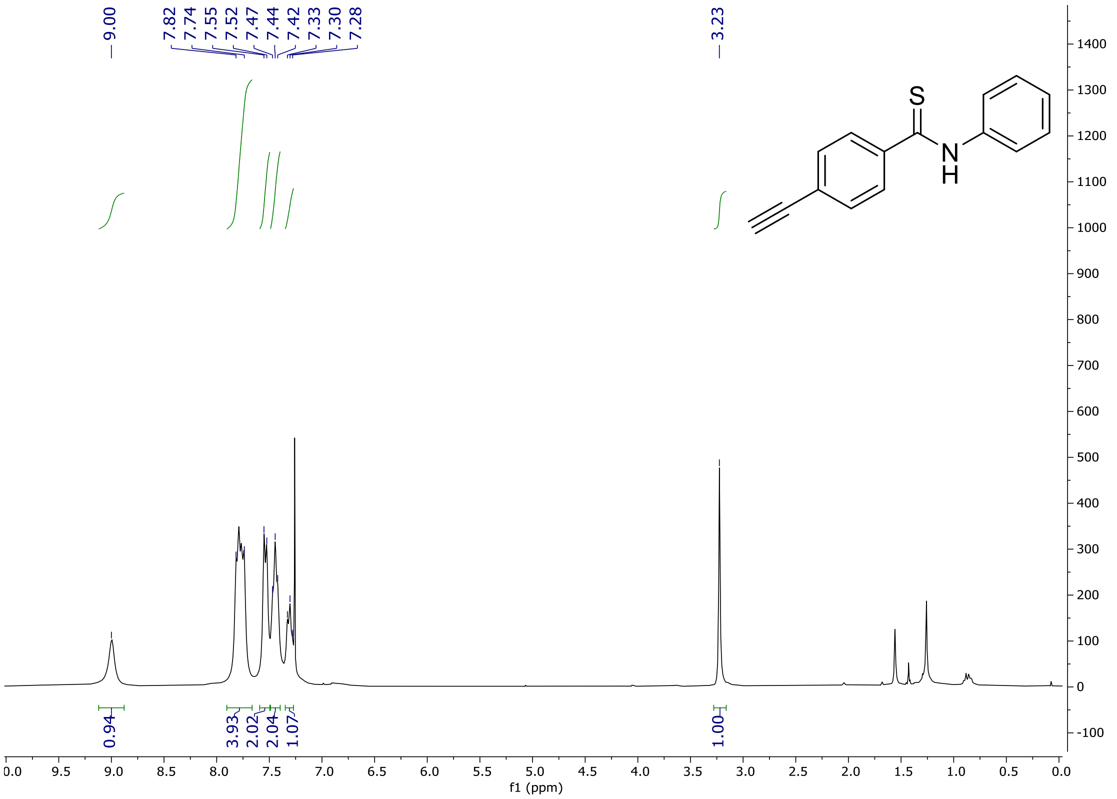

Figure SI_159: ^{1}H-NMR for **3l** in $CDCl_3$ (300 MHz).

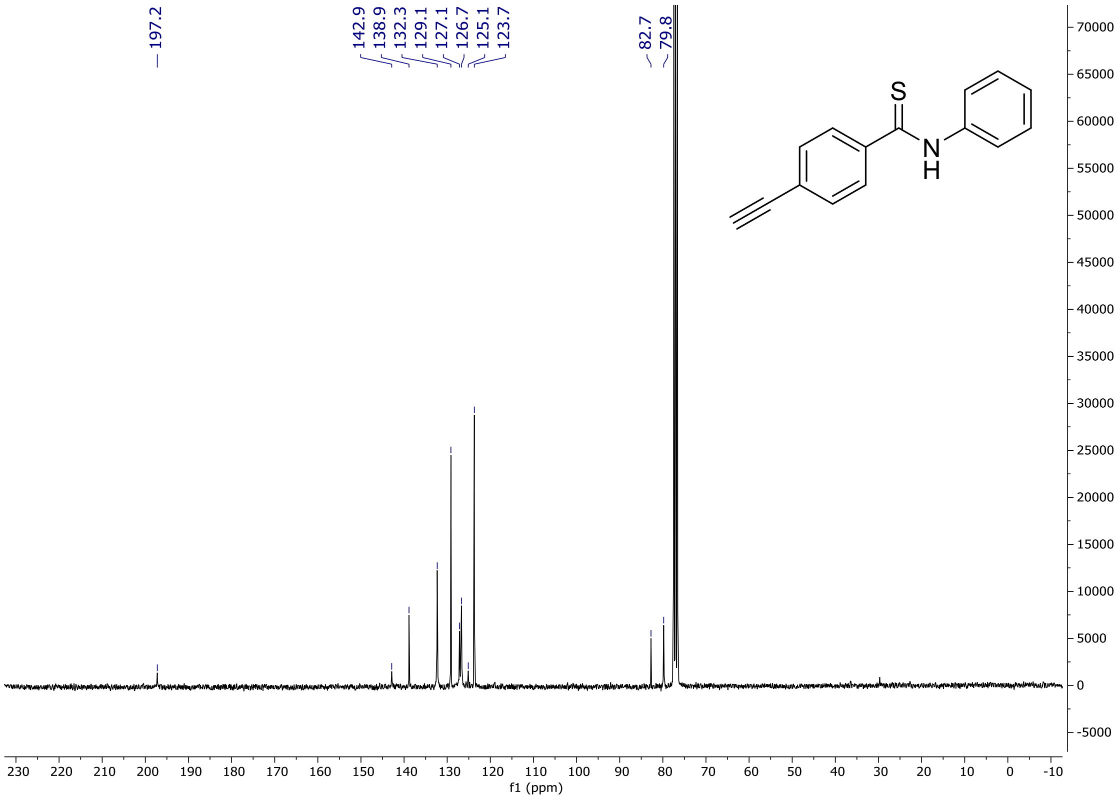

Figure SI_160: ^{13}C-NMR for **3l** in $CDCl_3$ (75 MHz).

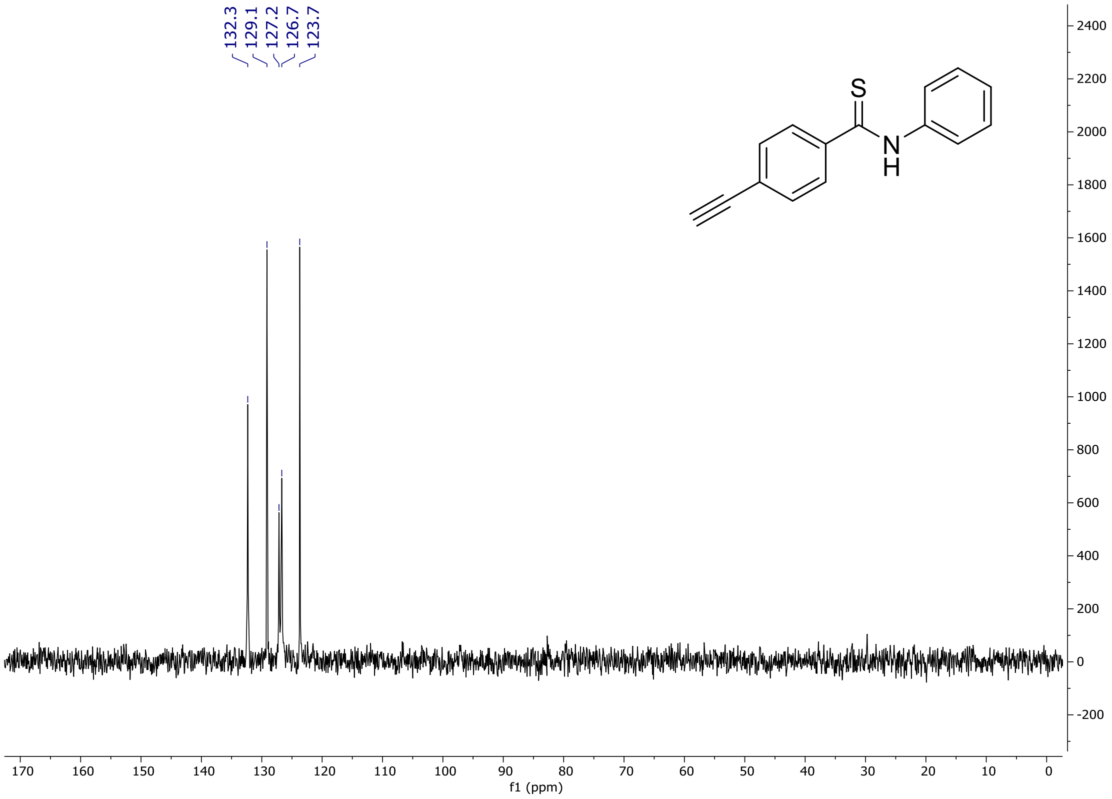

Figure SI_161: DEPT 135-NMR for **3l** in $CDCl_3$ (75 MHz).

Acquisition Parameter

Source Type	ESI	Ion Polarity	Positive	Set Nebulizer	2.4 Bar
Focus	Active	Set Capillary	3500 V	Set Dry Heater	250 °C
Scan Begin	50 m/z	Set End Plate Offset	-500 V	Set Dry Gas	6.0 l/min
Scan End	1500 m/z	Set Charging Voltage	2000 V	Set Divert Valve	Source
		Set Corona	0 nA	Set APCI Heater	0 °C

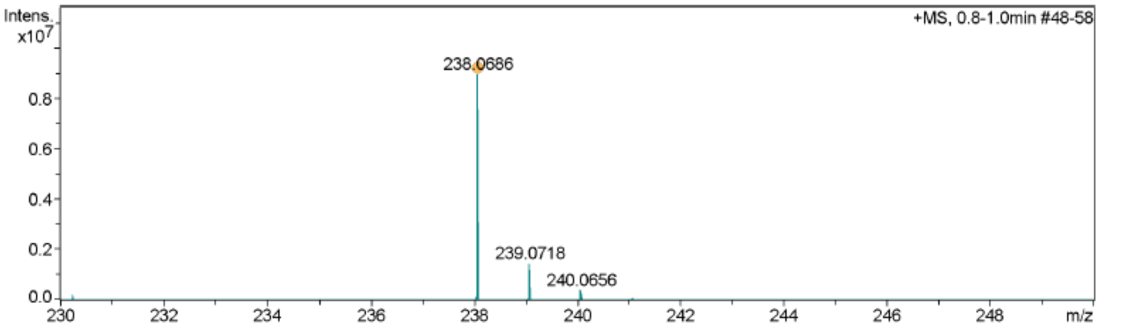

Meas. m/z	#	Ion Formula	m/z	err [ppm]	mSigma	# mSigma	Score	rdb	e¯ Conf	N-Rule	Adduct
238.0686	1	C15H12NS	238.0685	-0.5	9.9	1	100.00	11.0	even	ok	M+H

Figure SI_162: HRMS (ESI+, m/z) analysis of **3l**.

_N_-Phenyl-4-(4,4,5,5-tetramethyl-1,3,2-dioxaborolan-2-yl)benzothioamide (3m)

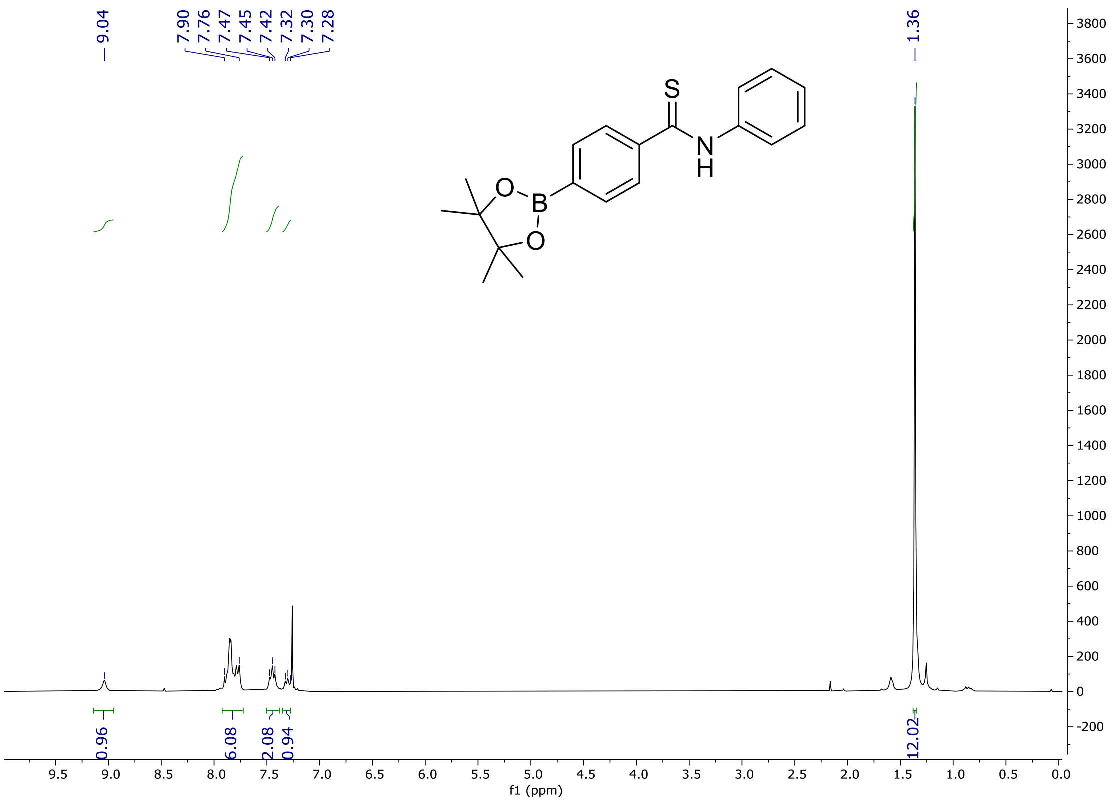

Figure SI_163: ^{1}H-NMR for **3m** in $CDCl_3$ (300 MHz).

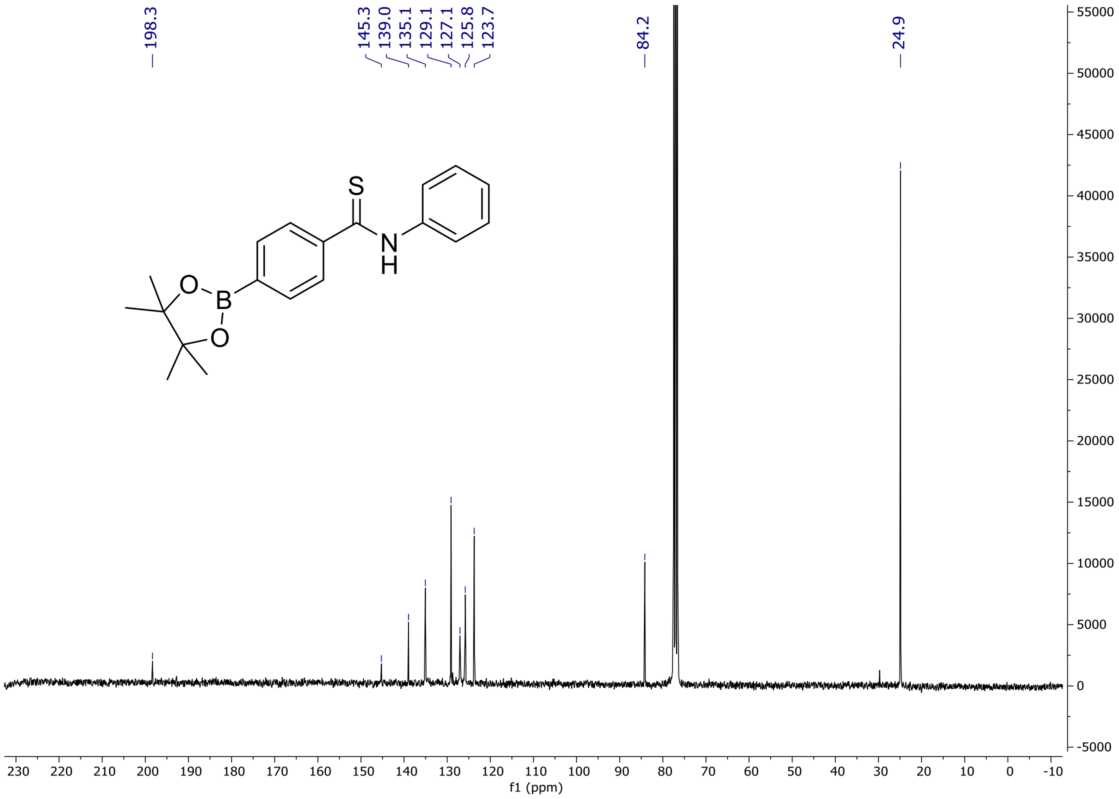

Figure SI_164: ^{13}C-NMR for **3m** in $CDCl_3$ (75 MHz).

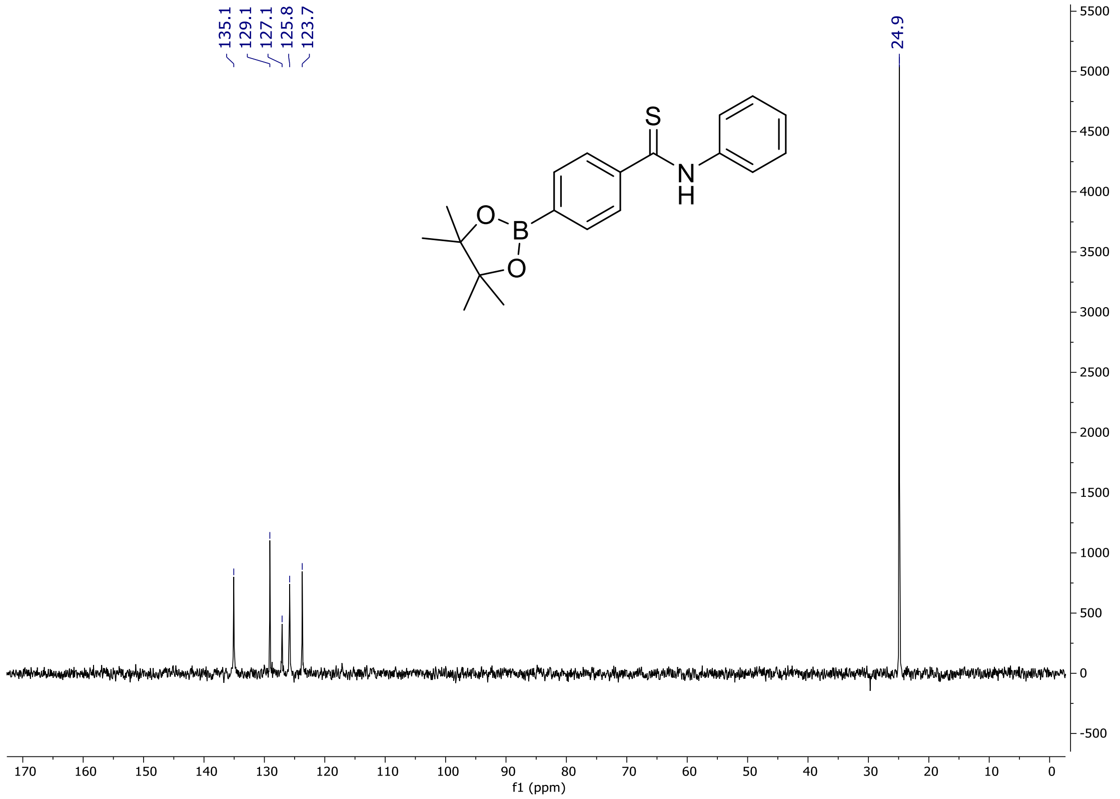

Figure SI_165: DEPT 135-NMR for **3m** in $CDCl_3$ (75 MHz).

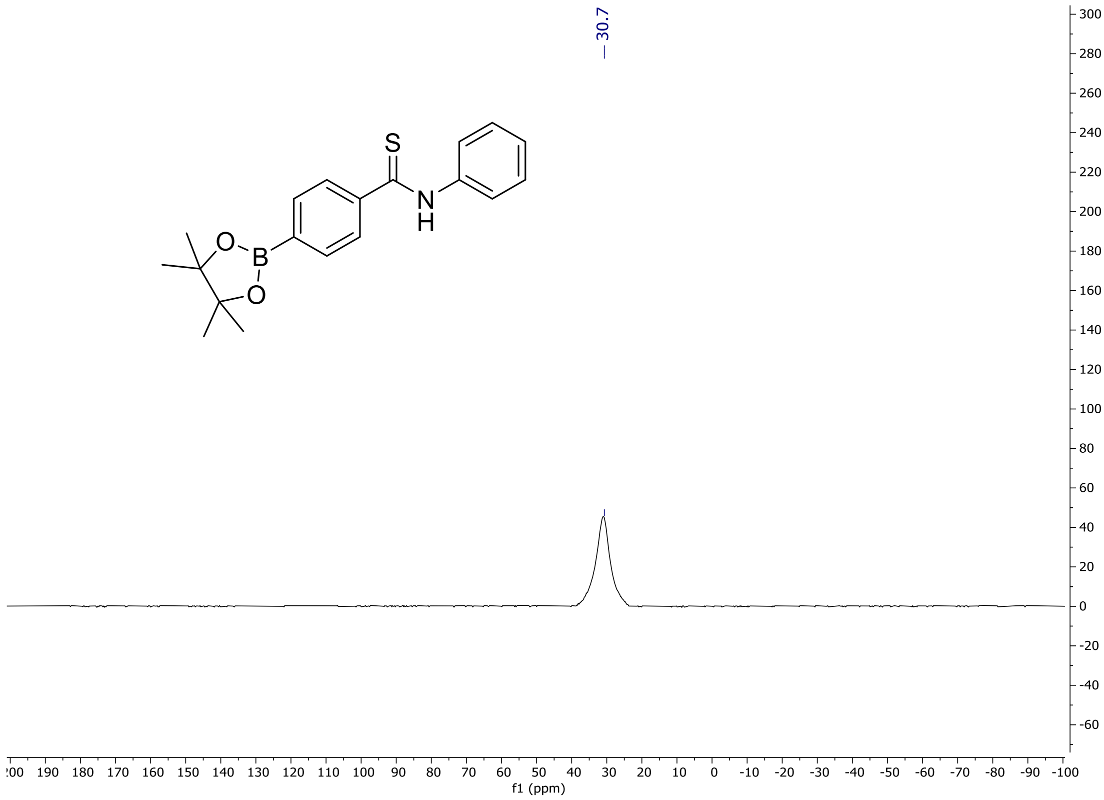

Figure SI_166: ^{11}B-NMR for **3m** in $CDCl_3$ (129 MHz).

Acquisition Parameter

Source Type	ESI	Ion Polarity	Positive	Set Nebulizer	2.4 Bar
Focus	Active	Set Capillary	4000 V	Set Dry Heater	250 °C
Scan Begin	50 m/z	Set End Plate Offset	-500 V	Set Dry Gas	6.0 l/min
Scan End	1500 m/z	Set Charging Voltage	2000 V	Set Divert Valve	Source
		Set Corona	0 nA	Set APCI Heater	0 °C

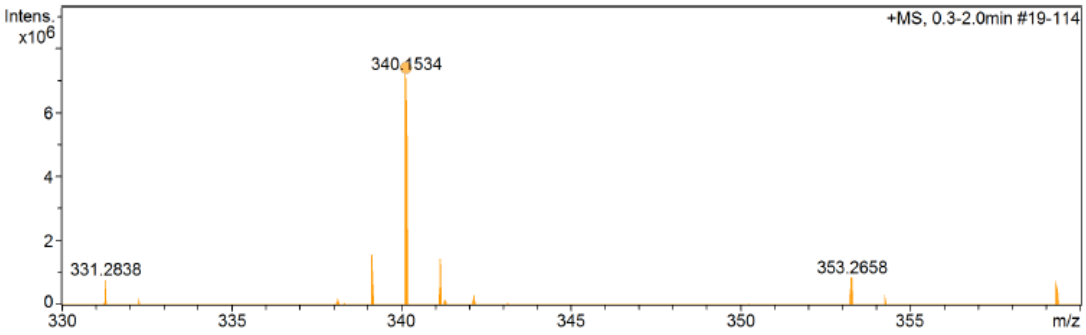

Meas. m/z	#	Ion Formula	m/z	err [ppm]	mSigma	# mSigma	Score	rdb	e⁻ Conf	N-Rule	Adduct
340.1534	1	C19H23BNO2S	340.1537	1.8	16.9	1	100.00	10.0	even	ok	M+H

Figure SI_167: HRMS (ESI$^+$, m/z) analysis of **3m**.

4-Acetyl-*N*-phenylbenzothioamide (3n)

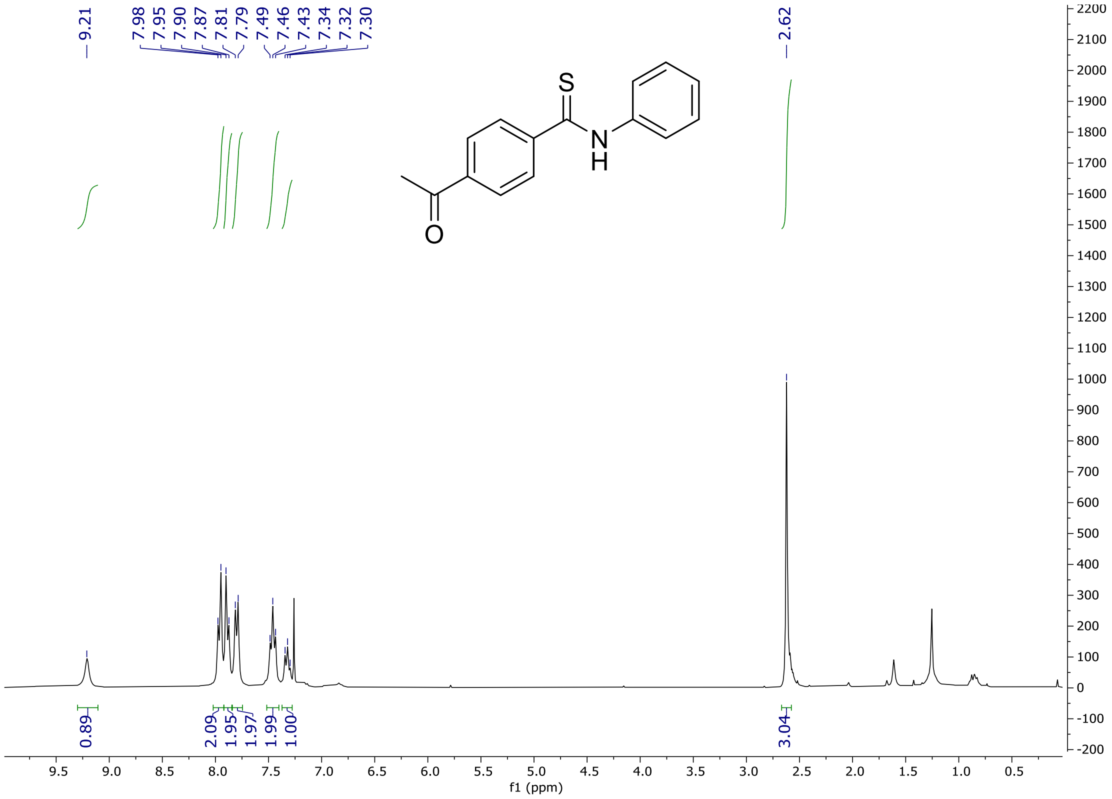

Figure SI_168: ^{1}H-NMR for **3n** in $CDCl_3$ (300 MHz).

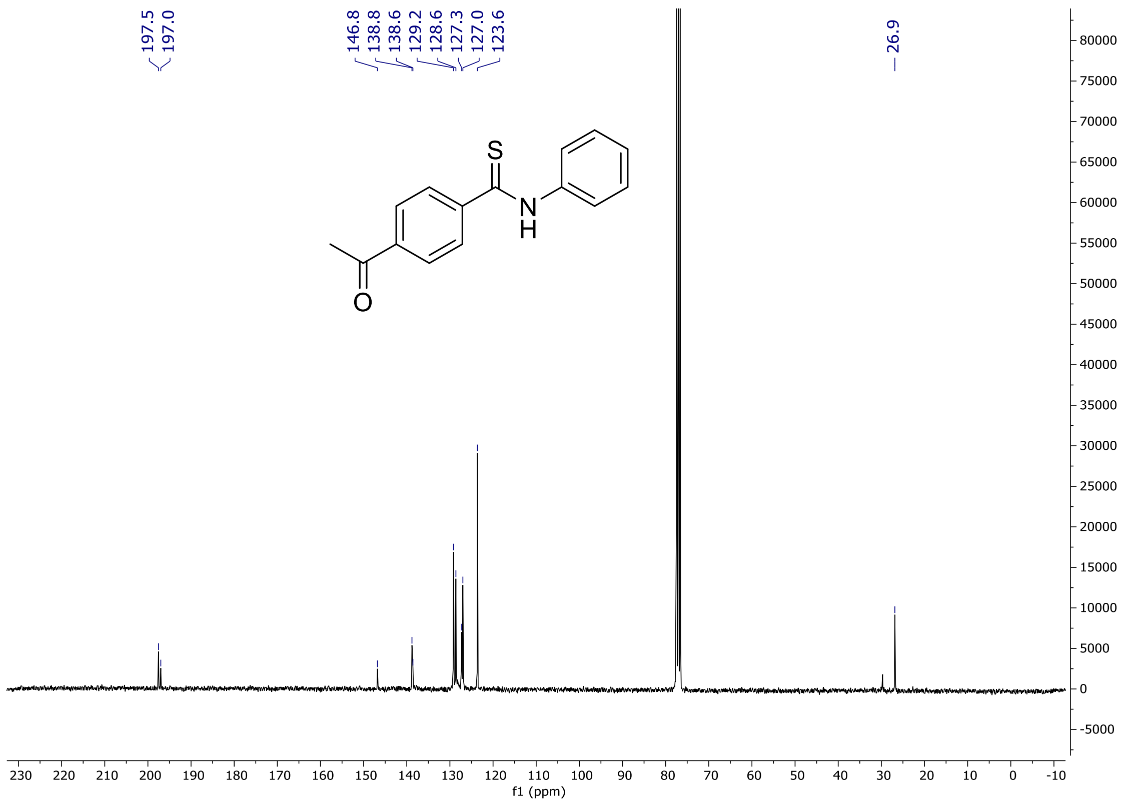

Figure SI_169: ^{13}C-NMR for **3n** in $CDCl_3$ (75 MHz).

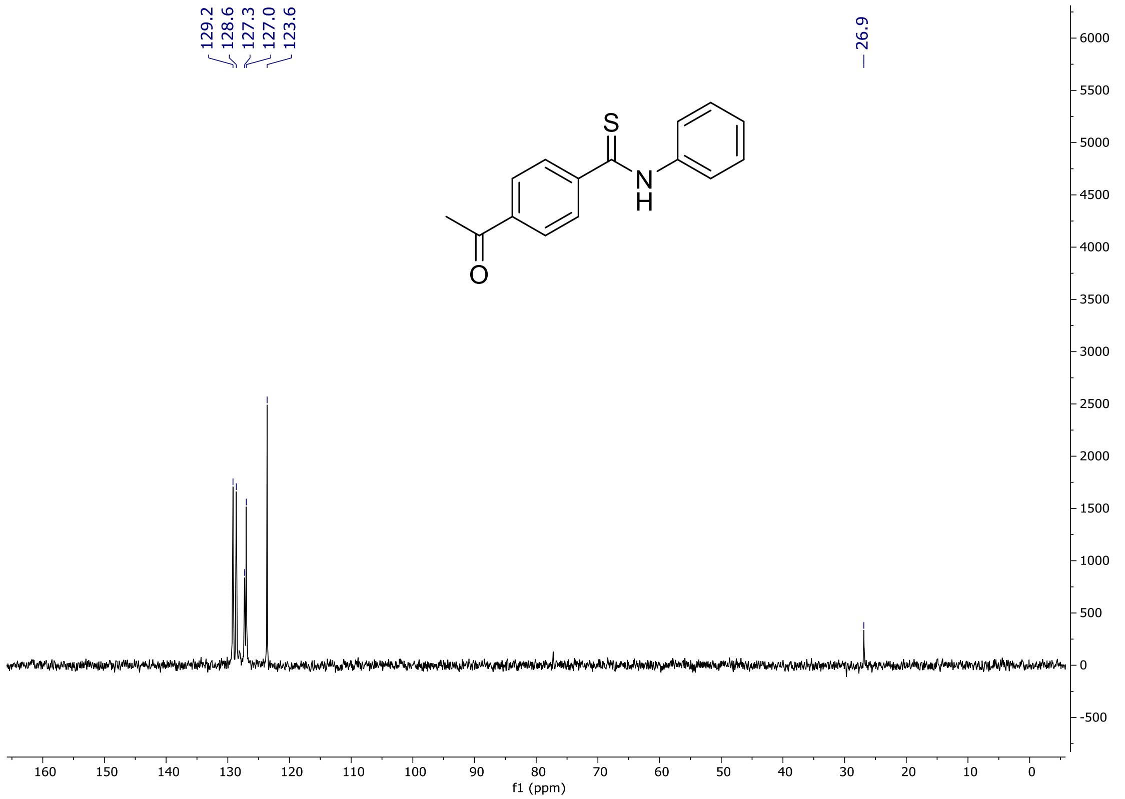

Figure SI_170: DEPT 135-NMR for **3n** in $CDCl_3$ (75 MHz).

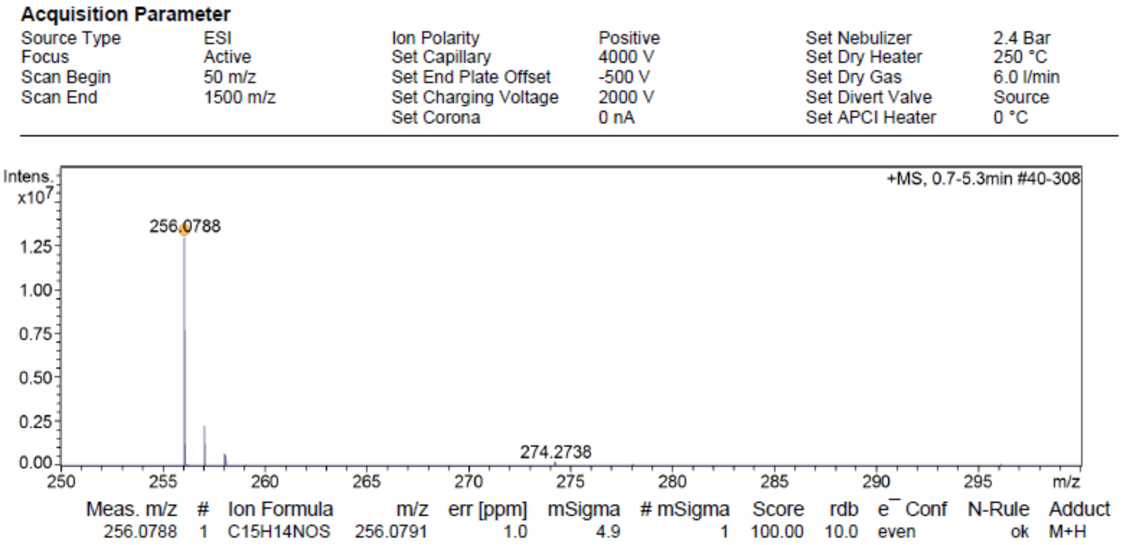

Figure SI_171: HRMS (ESI^+, m/z) analysis of **3n**.

4-(Methoxycarbonyl)-*N*-phenylbenzothioamide (3o)

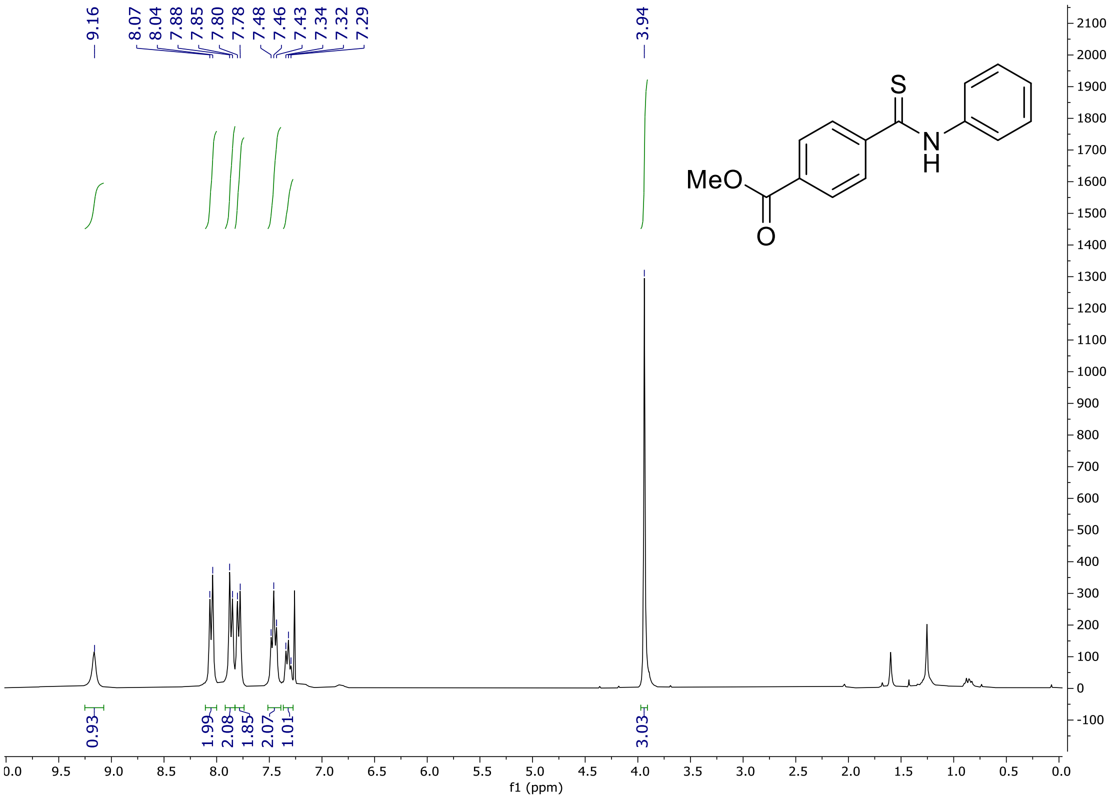

Figure SI_172: ^{1}H-NMR for **3o** in $CDCl_3$ (300 MHz).

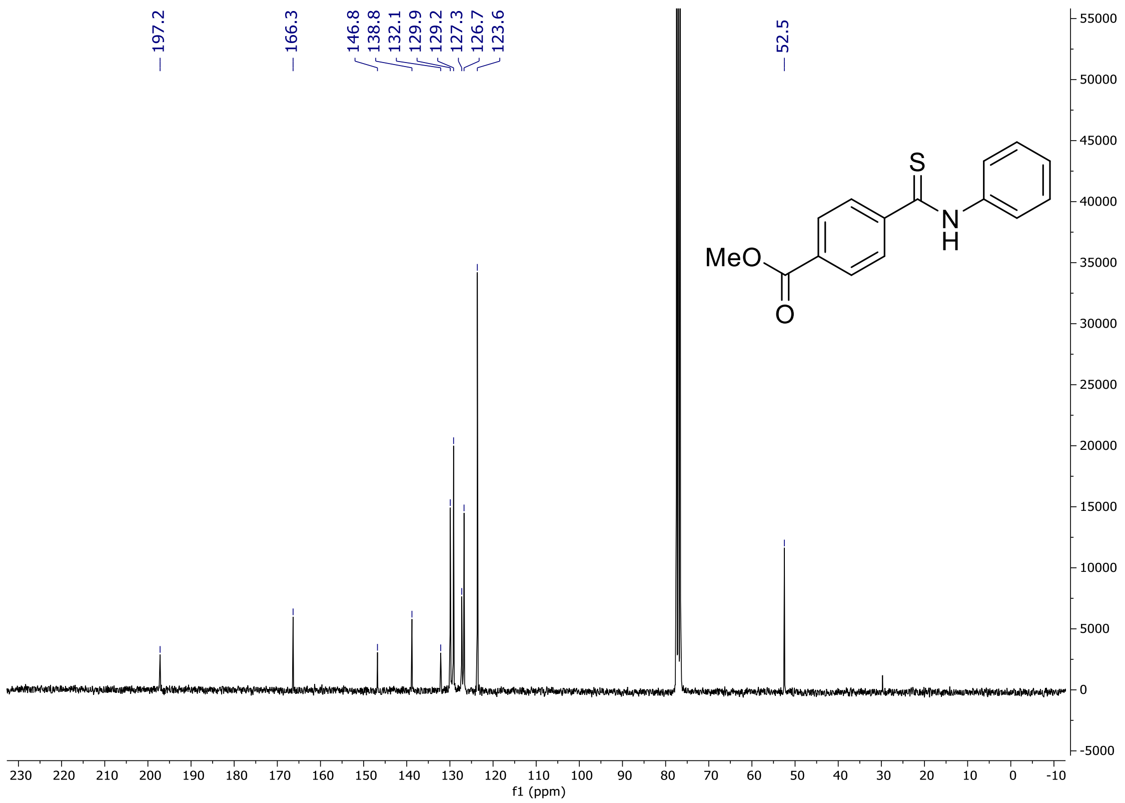

Figure SI_173: ^{13}C-NMR for **3o** in $CDCl_3$ (75 MHz).

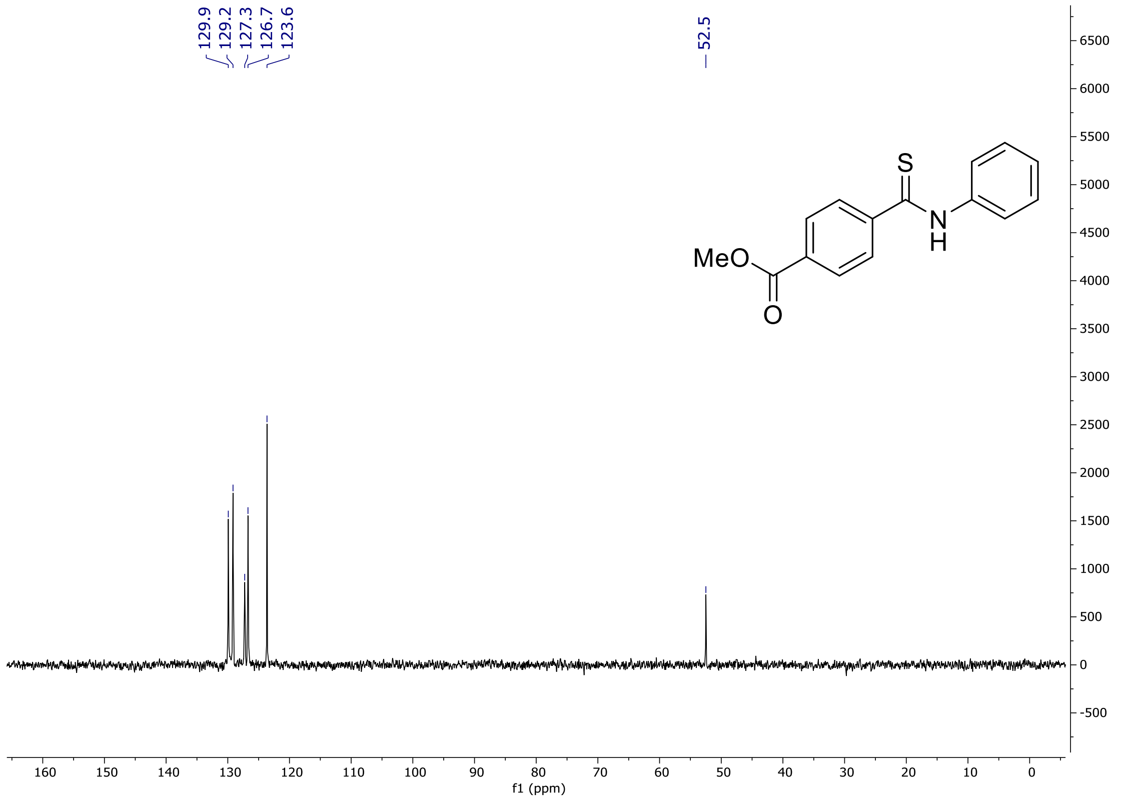

Figure SI_174: DEPT 135-NMR for **3o** in $CDCl_3$ (75 MHz).

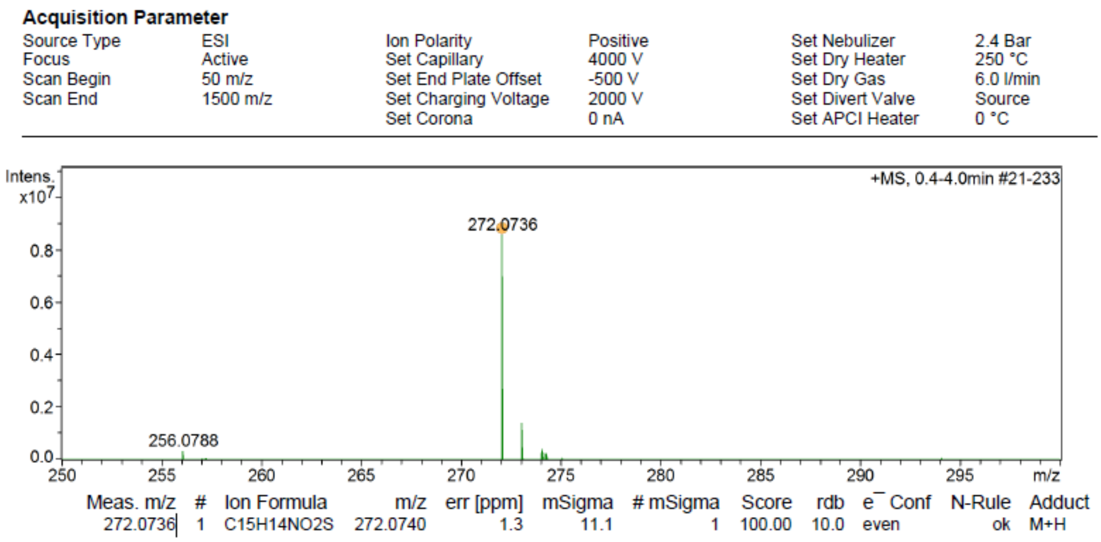

Figure SI_175: HRMS (ESI$^+$, m/z) analysis of **3o**.

4-(Isopropylcarbamoyl)-*N*-phenylbenzothioamide (3p)

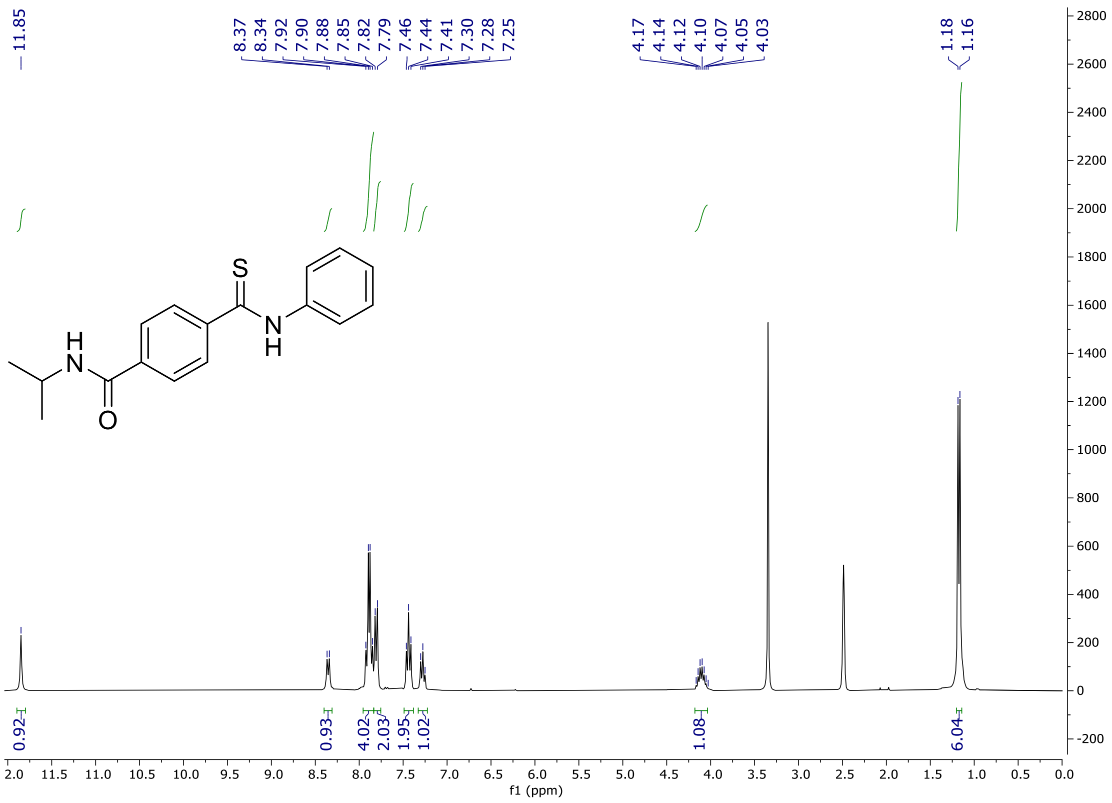

Figure SI_176: ^{1}H-NMR for **3p** in DMSO-d^6 (300 MHz).

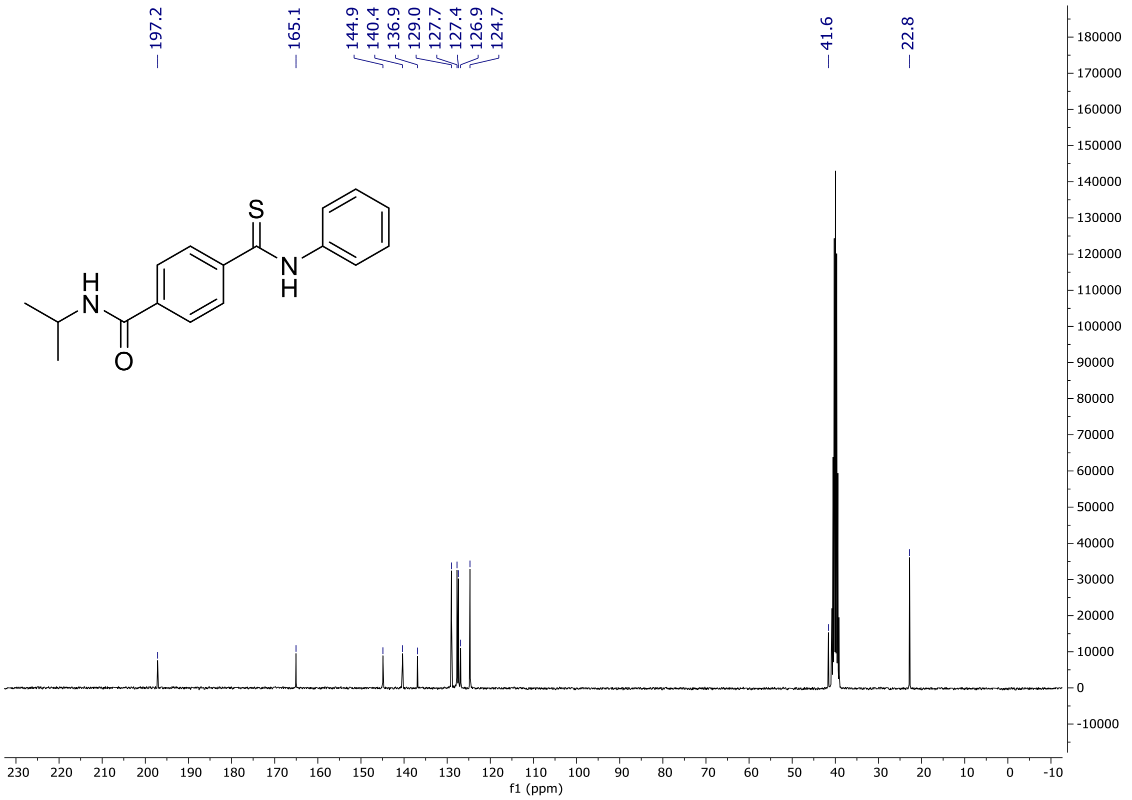

Figure SI_177: ^{13}C-NMR for **3p** in DMSO-*d*^{6} (75 MHz).

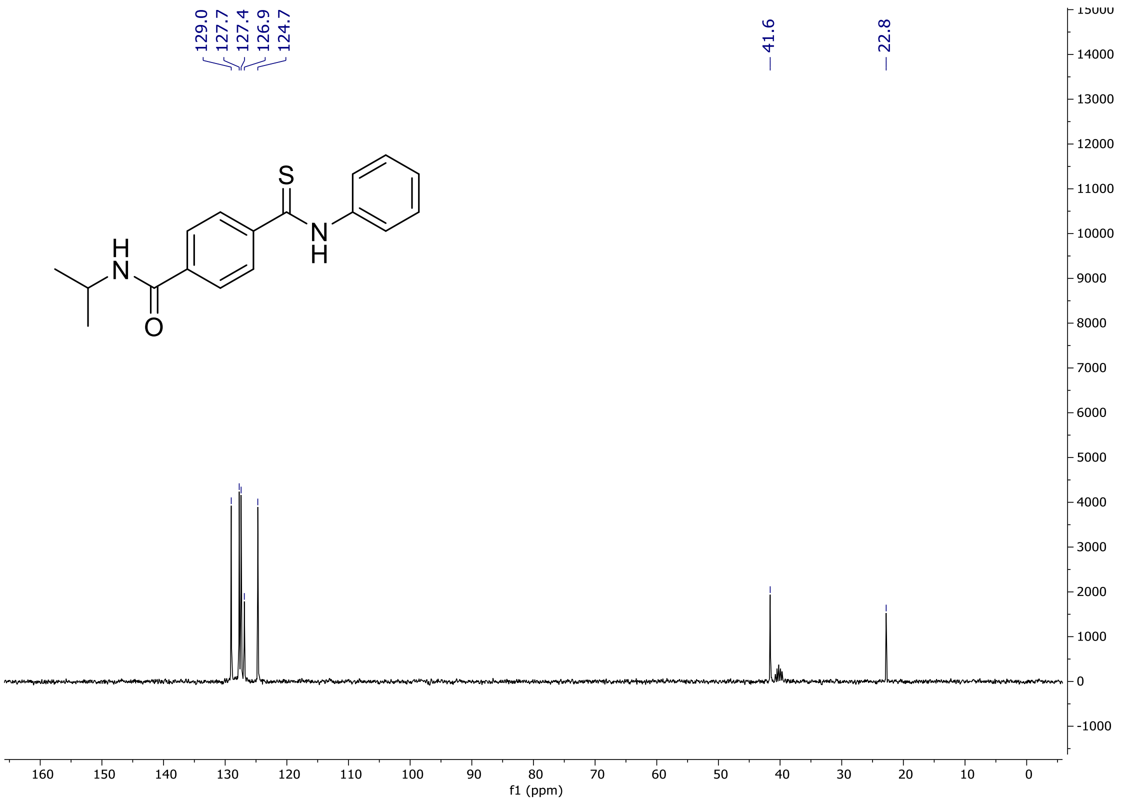

Figure SI_178: DEPT 135-NMR for **3p** in DMSO-*d*^{6} (75 MHz).

Acquisition Parameter

Source Type	ESI	Ion Polarity	Positive	Set Nebulizer	2.4 Bar
Focus	Active	Set Capillary	4000 V	Set Dry Heater	250 °C
Scan Begin	50 m/z	Set End Plate Offset	-500 V	Set Dry Gas	6.0 l/min
Scan End	1500 m/z	Set Charging Voltage	2000 V	Set Divert Valve	Source
		Set Corona	0 nA	Set APCI Heater	0 °C

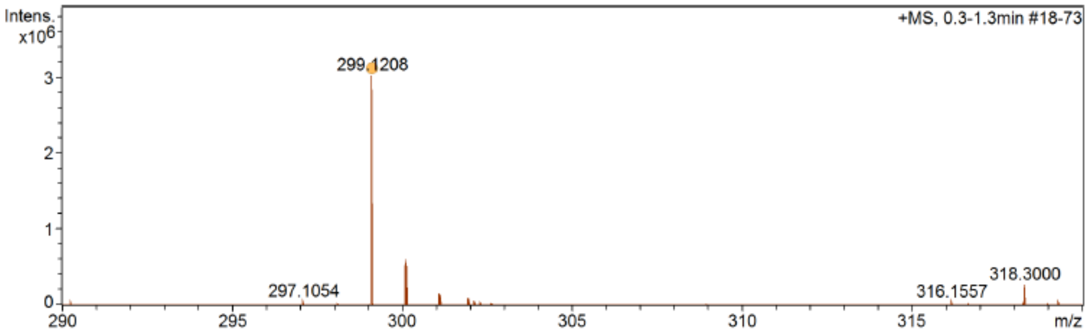

Meas. m/z	#	Ion Formula	m/z	err [ppm]	mSigma	# mSigma	Score	rdb	e⁻ Conf	N-Rule	Adduct
299.1208	1	C17H19N2OS	299.1213	1.4	13.6	1	100.00	10.0	even	ok	M+H

Figure SI_179: HRMS (ESI$^+$, m/z) analysis of **3p**.

N-Phenyl-4-(pyrrolidine-1-carbonyl)benzothioamide (3q)

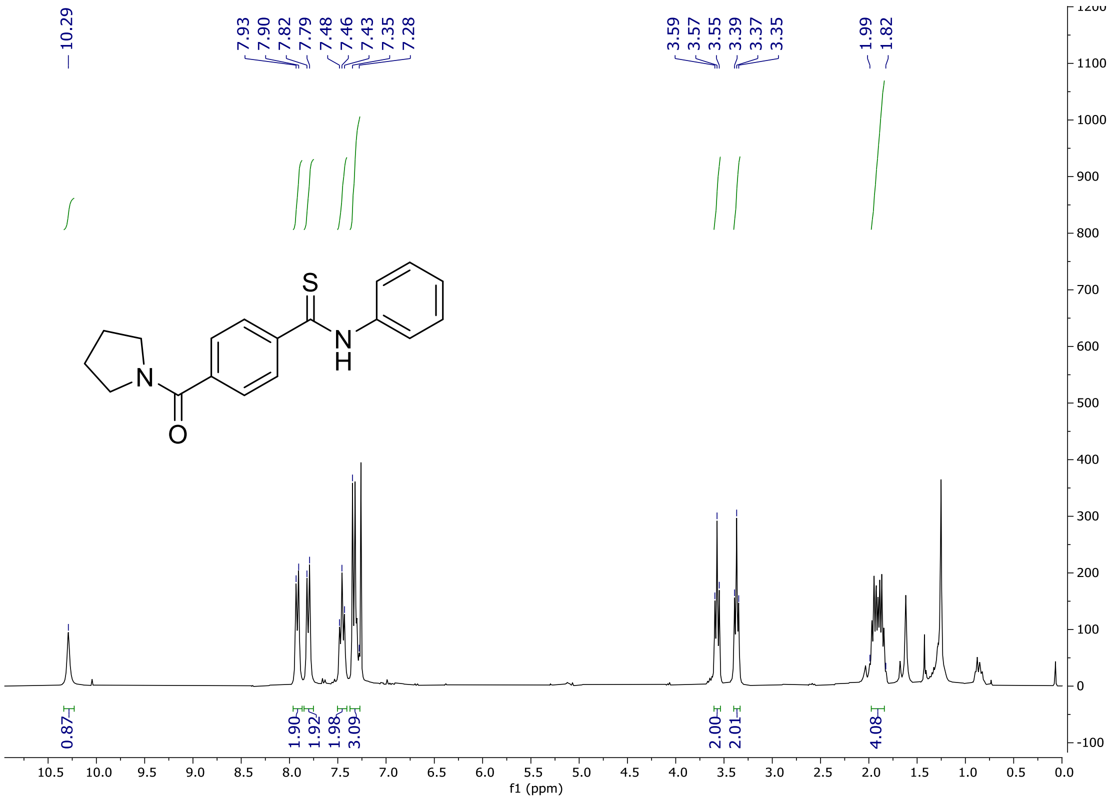

Figure SI_180: ^{1}H-NMR for **3q** in $CDCl_3$ (300 MHz).

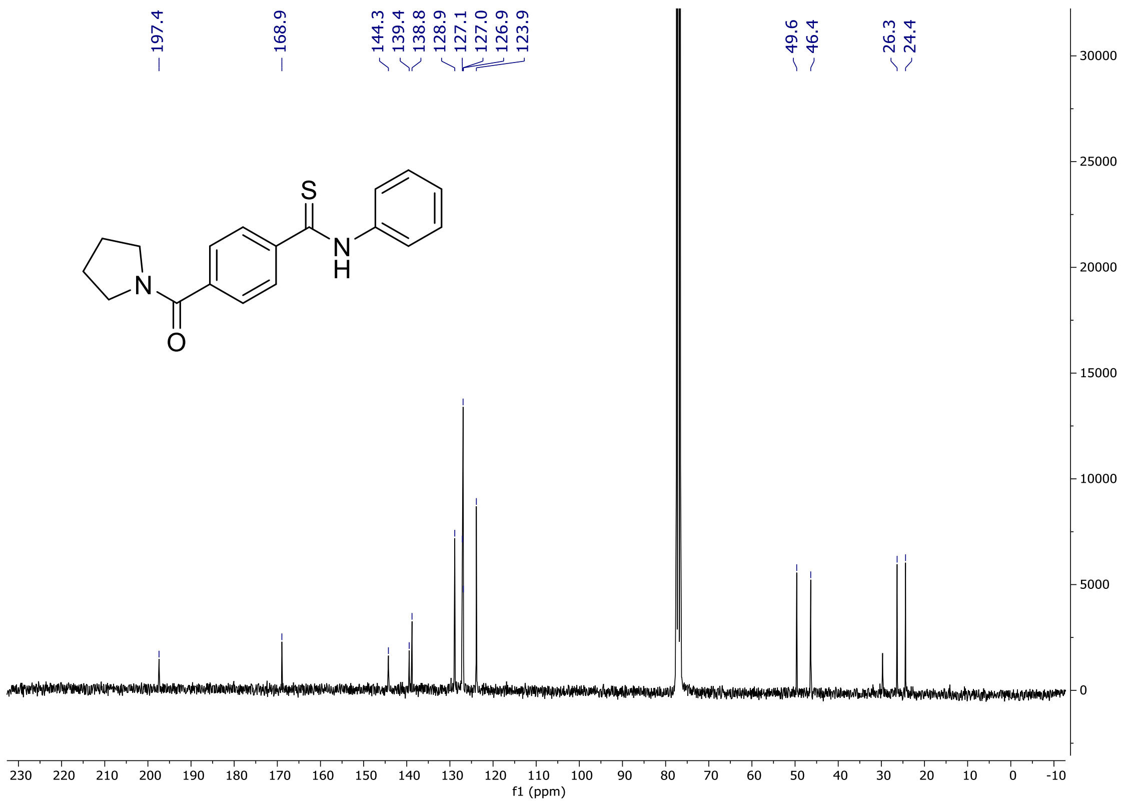

Figure SI_181: ^{13}C-NMR for **3q** in $CDCl_3$ (75 MHz).

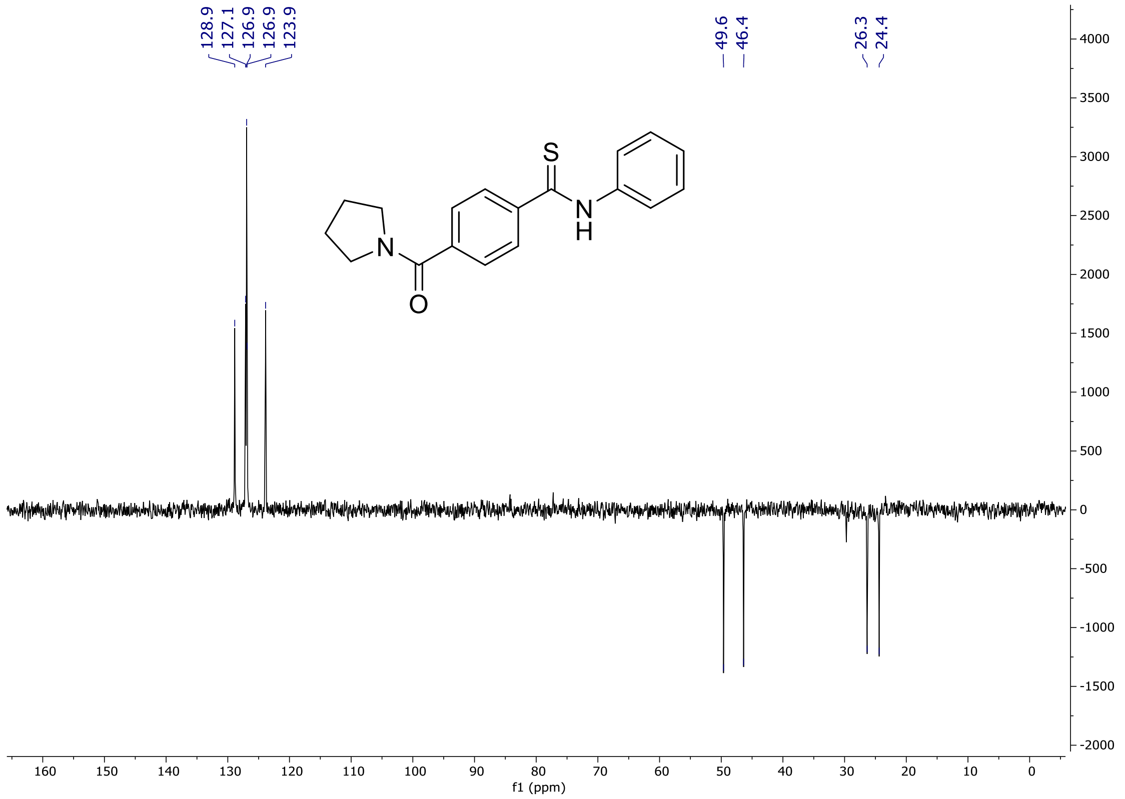

Figure SI_182: DEPT 135-NMR for **3q** in $CDCl_3$ (75 MHz).

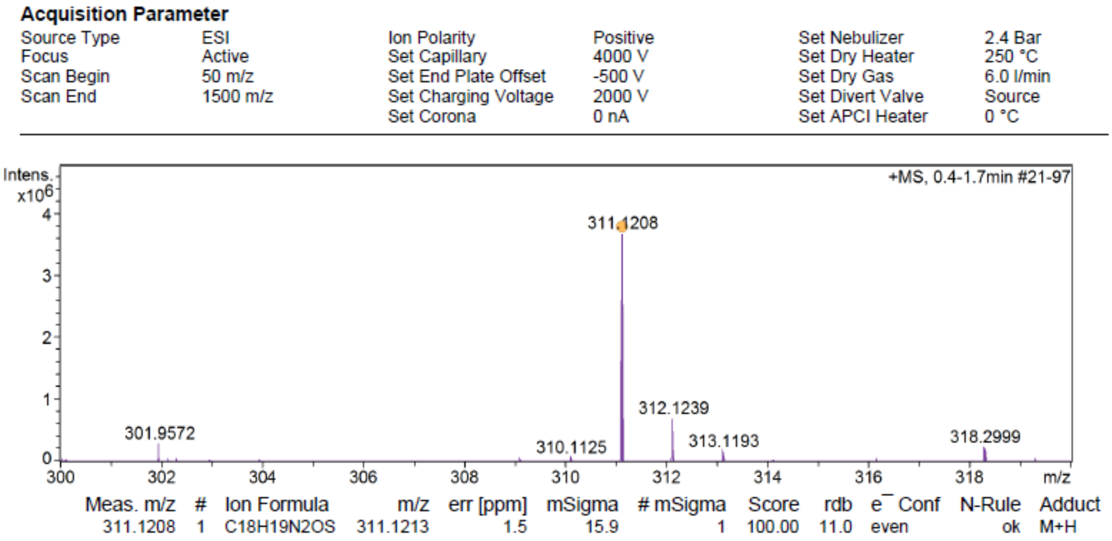

Figure SI_183: HRMS (ESI$^+$, m/z) analysis of **3q**.

4-Methyl-*N*-phenylbenzothioamide (3r)

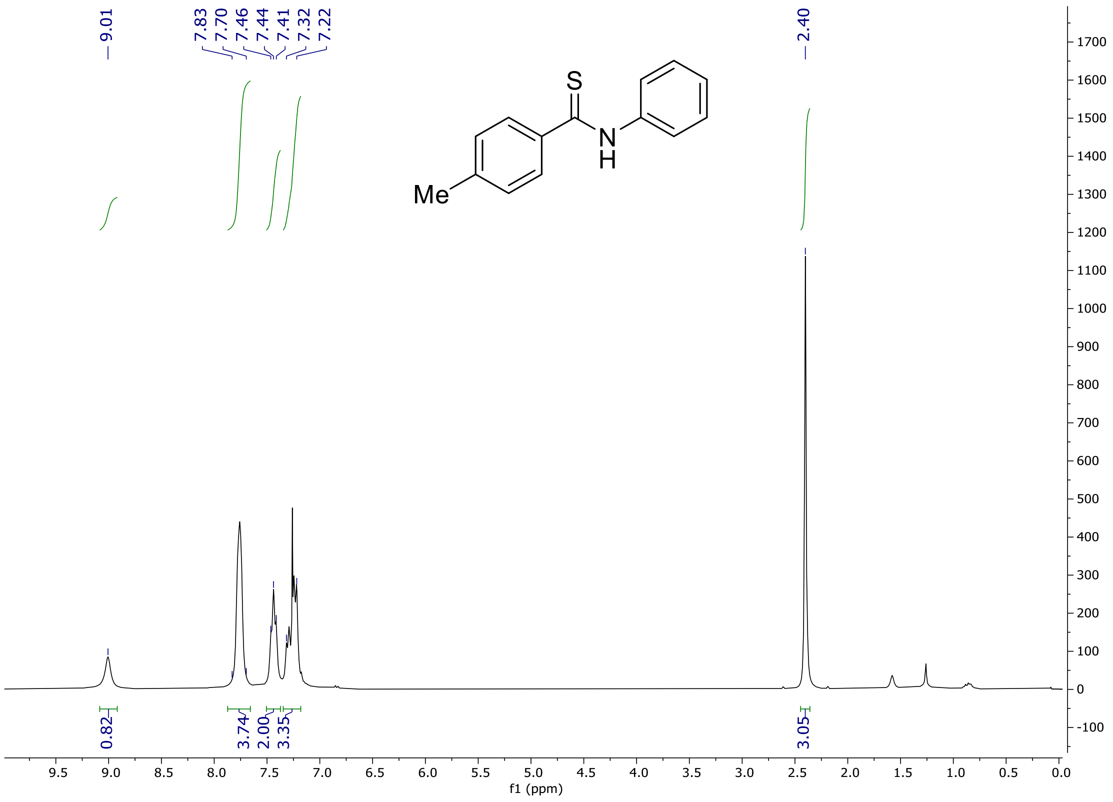

Figure SI_184: ^{1}H-NMR for **3r** in $CDCl_3$ (300 MHz).

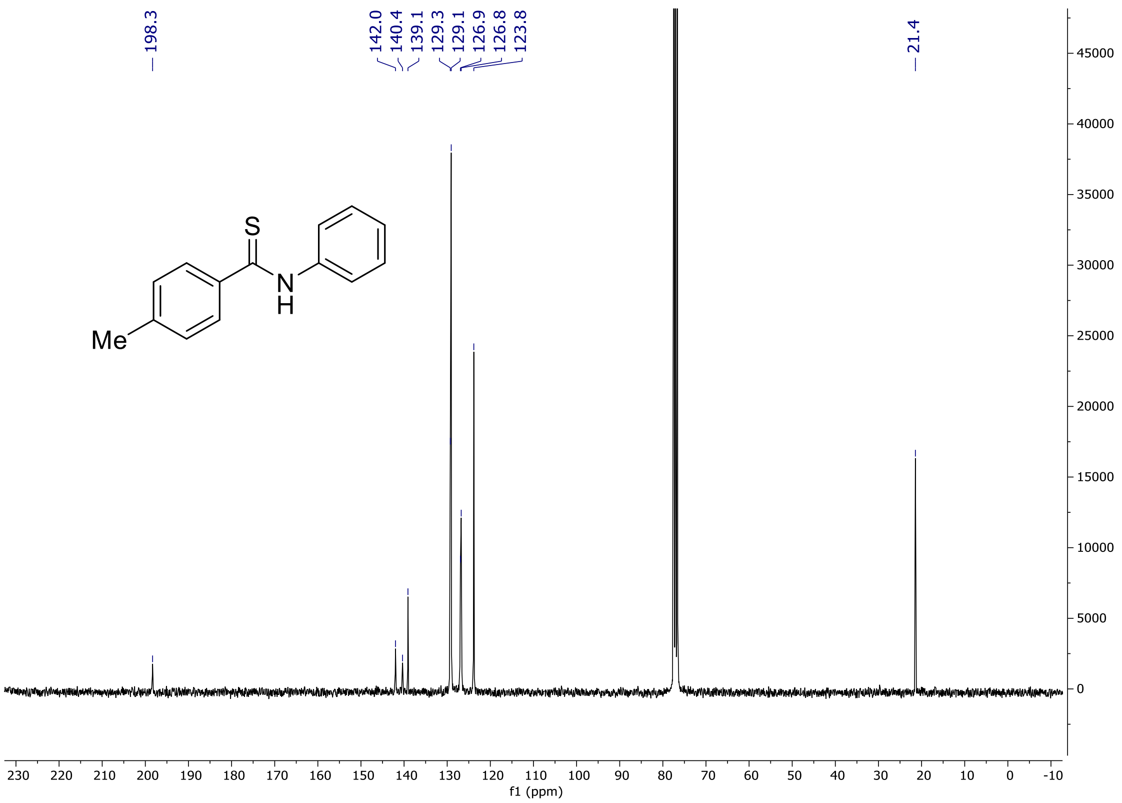

Figure SI_185: ^{13}C-NMR for **3r** in $CDCl_3$ (75 MHz).

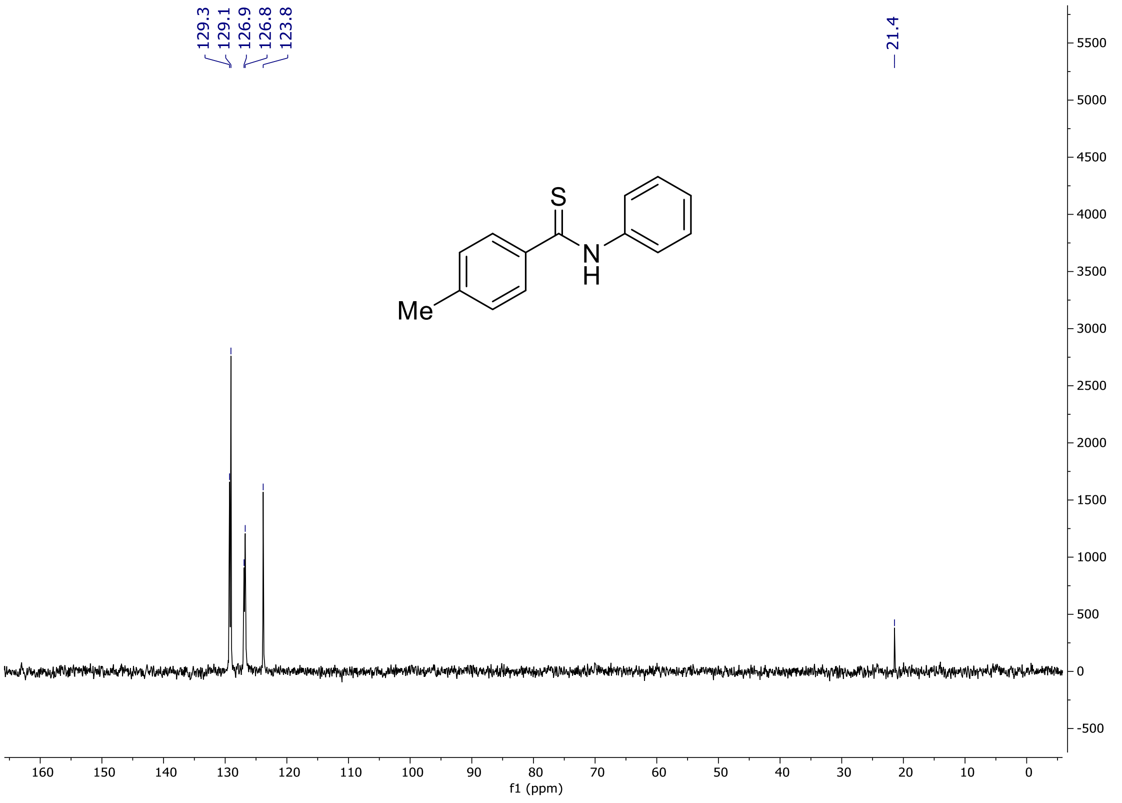

Figure SI_186: DEPT 135-NMR for **3r** in $CDCl_3$ (75 MHz).

4-Hydroxy-*N*-phenylbenzothioamide (3s)

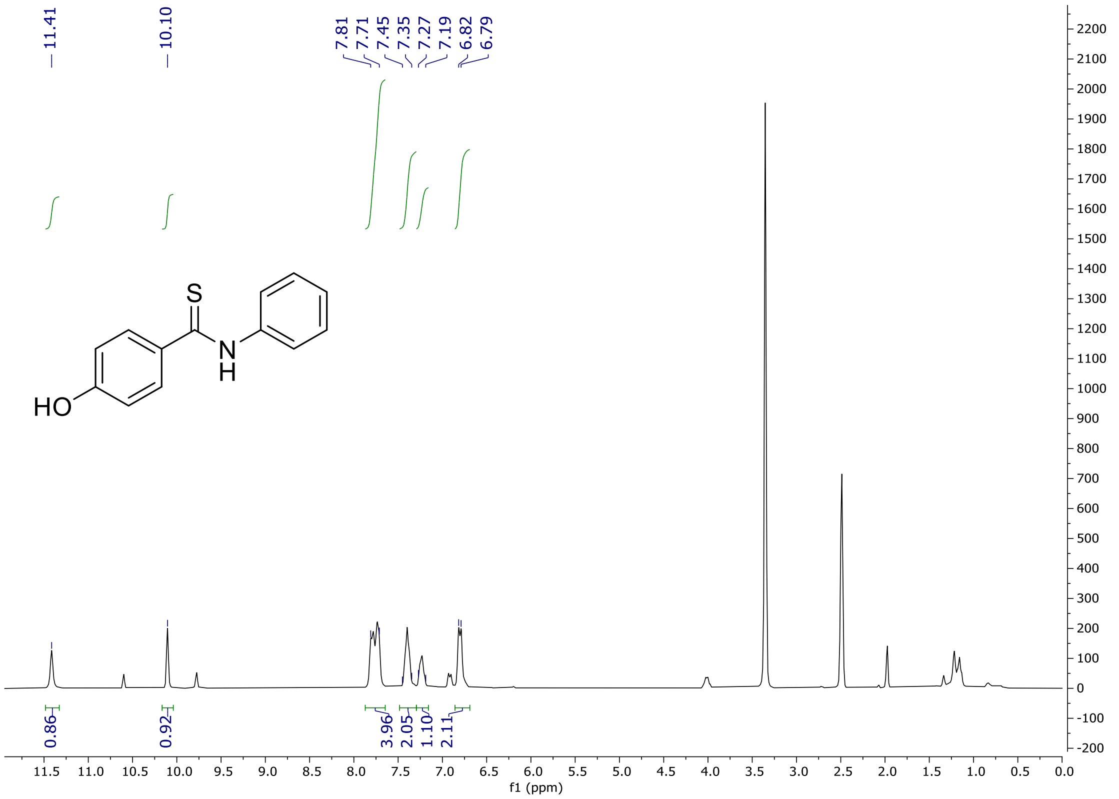

Figure SI_187: ^{1}H-NMR for **3s** in DMSO-d^6 (300 MHz).

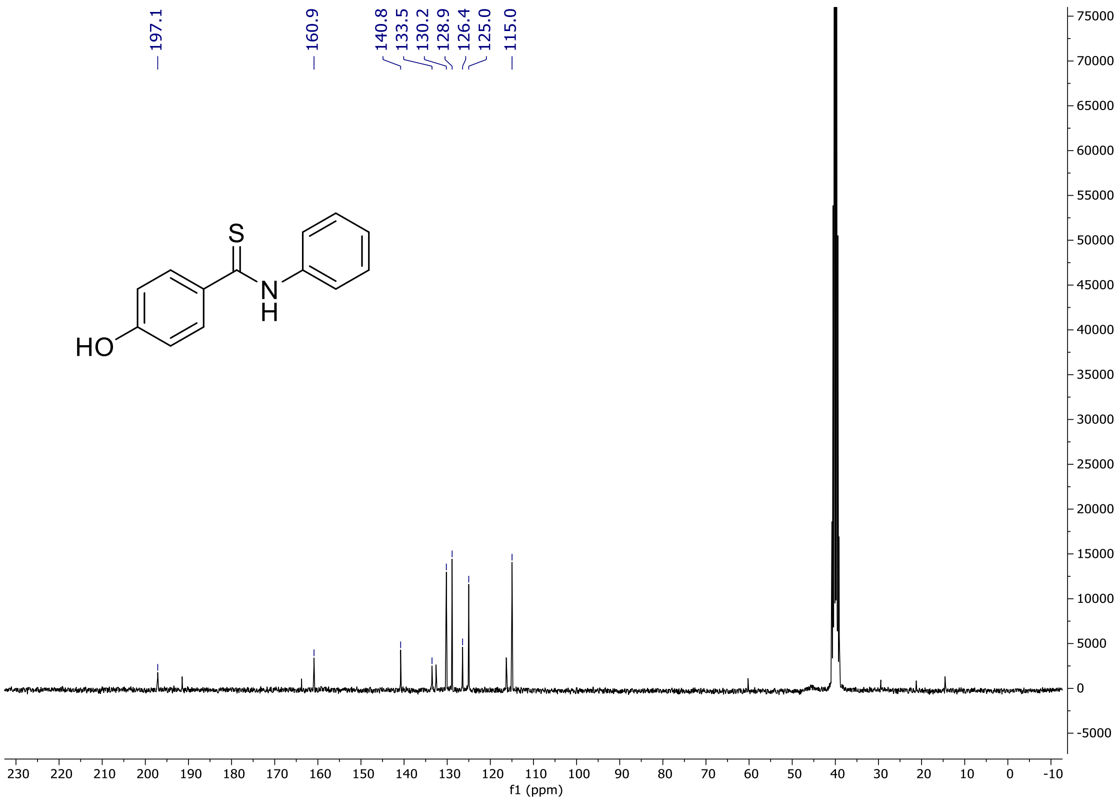

Figure SI_188: ^{13}C-NMR for **3s** in DMSO-d^6 (75 MHz).

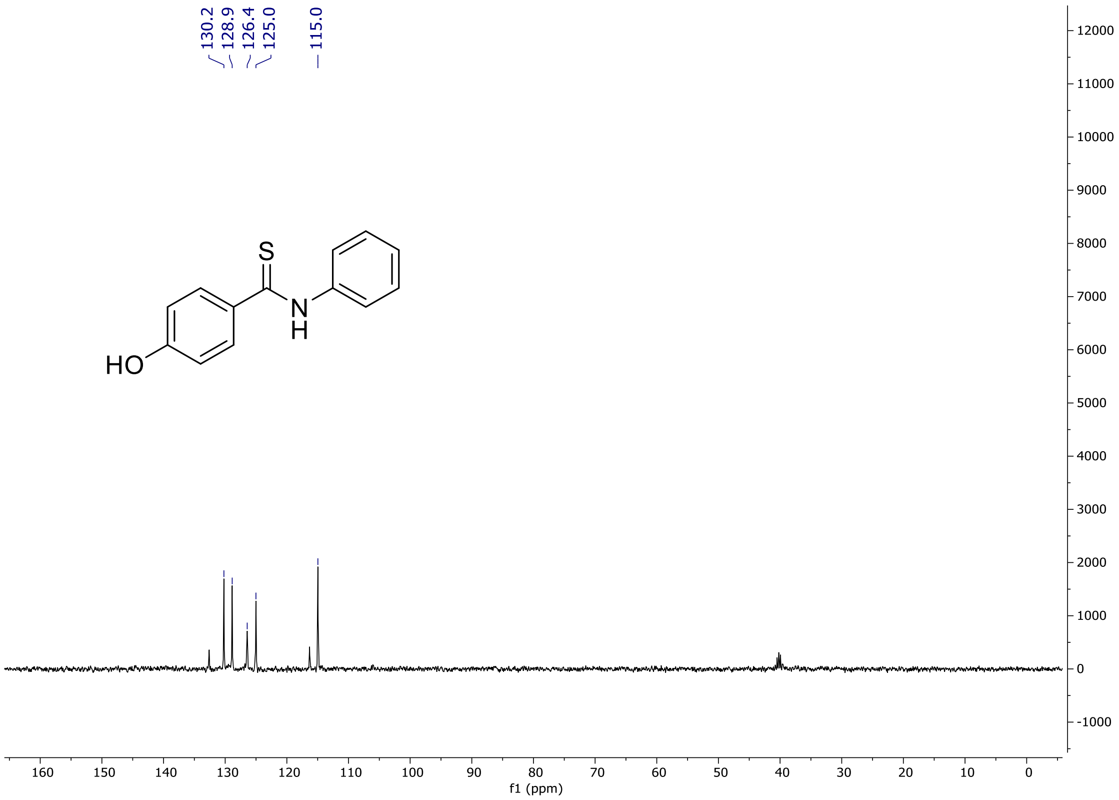

Figure SI_189: DEPT 135-NMR for **3s** in DMSO-d^6 (75 MHz).

4-Methoxy-*N*-phenylbenzothioamide (3t)

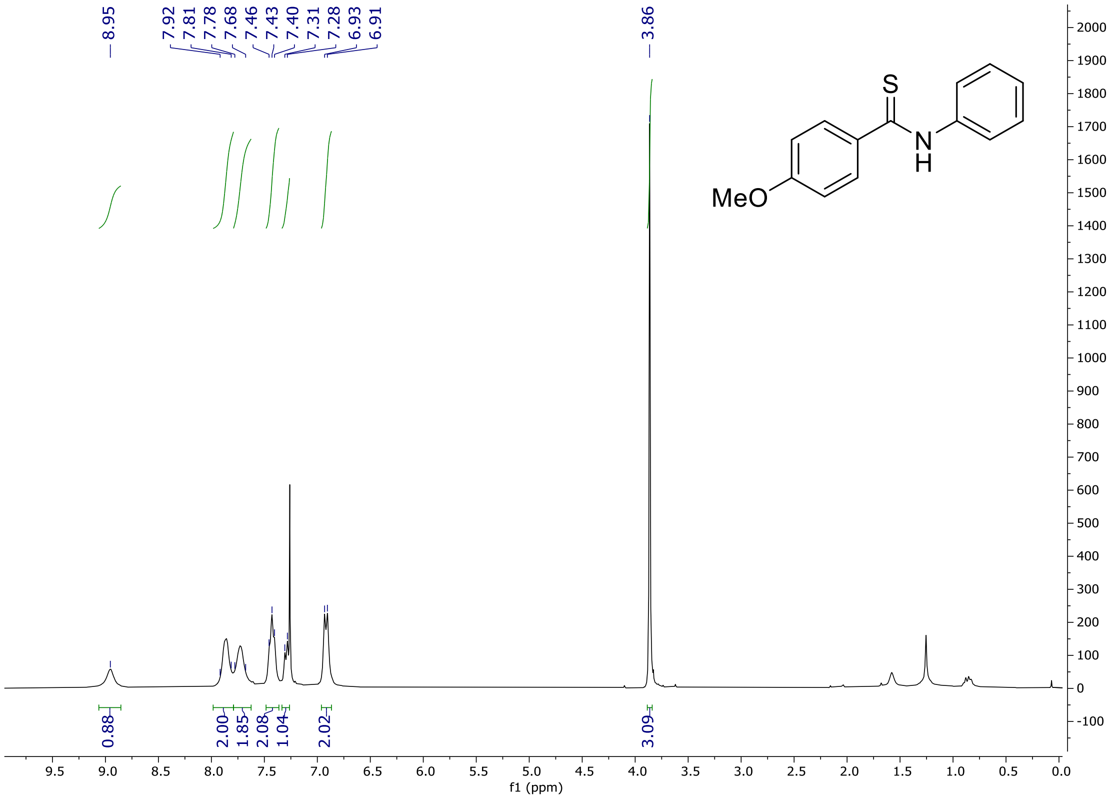

Figure SI_190: ^{1}H-NMR for **3t** in $CDCl_3$ (300 MHz).

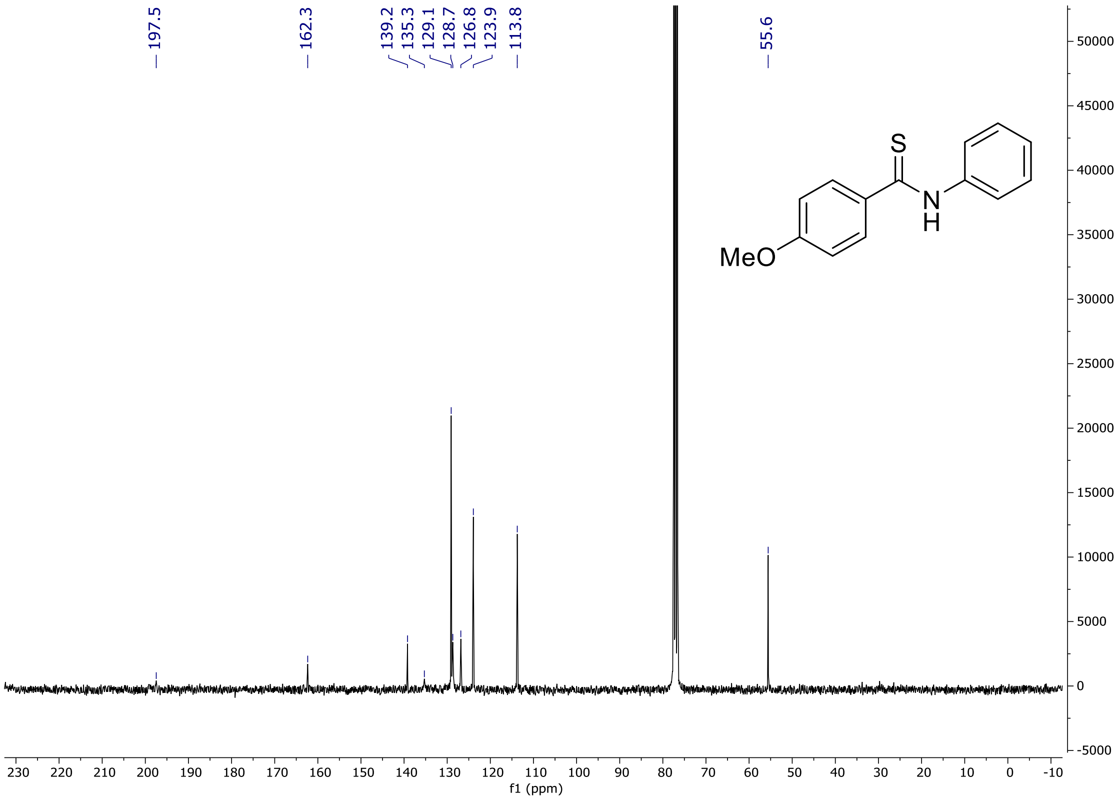

Figure SI_191: ^{13}C-NMR for **3t** in $CDCl_3$ (75 MHz).

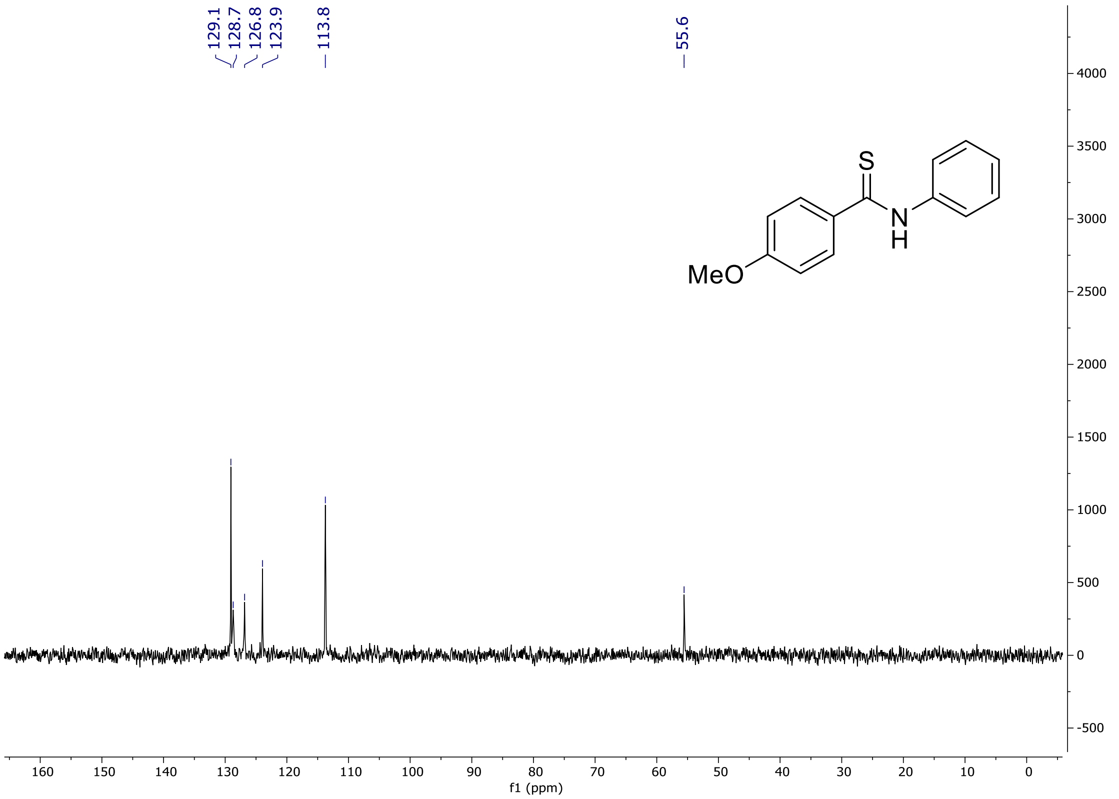

Figure SI_192: DEPT 135-NMR for **3t** in $CDCl_3$ (75 MHz).

4-Acetamido-*N*-phenylbenzothioamide (3u)

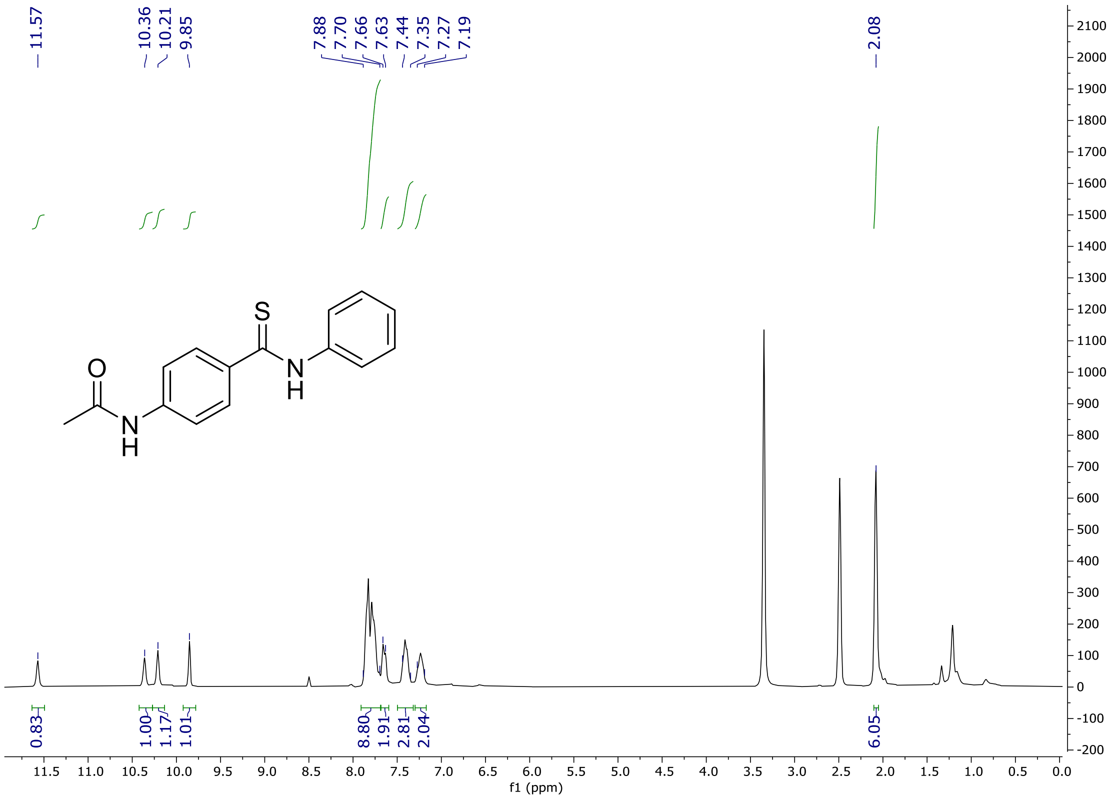

Figure SI_193: ^{1}H-NMR for **3u** in DMSO-d^6 (300 MHz).

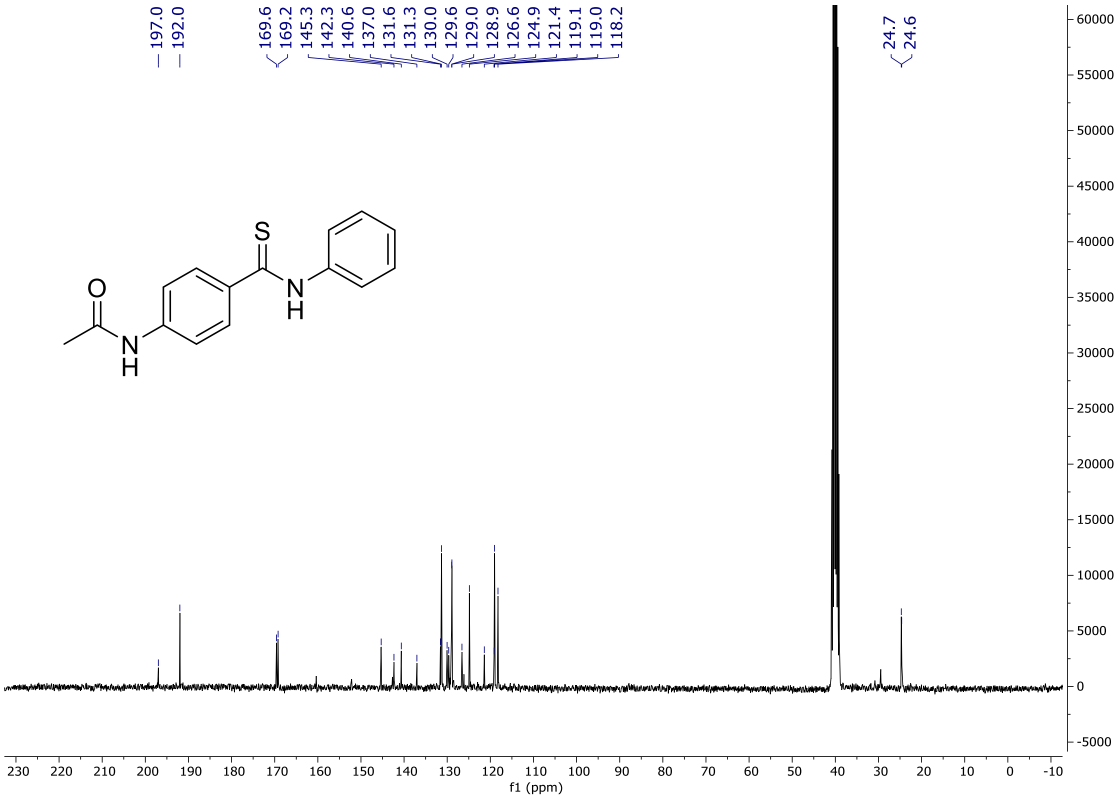

Figure SI_194: ^{13}C-NMR for **3u** in DMSO-d^6 (75 MHz).

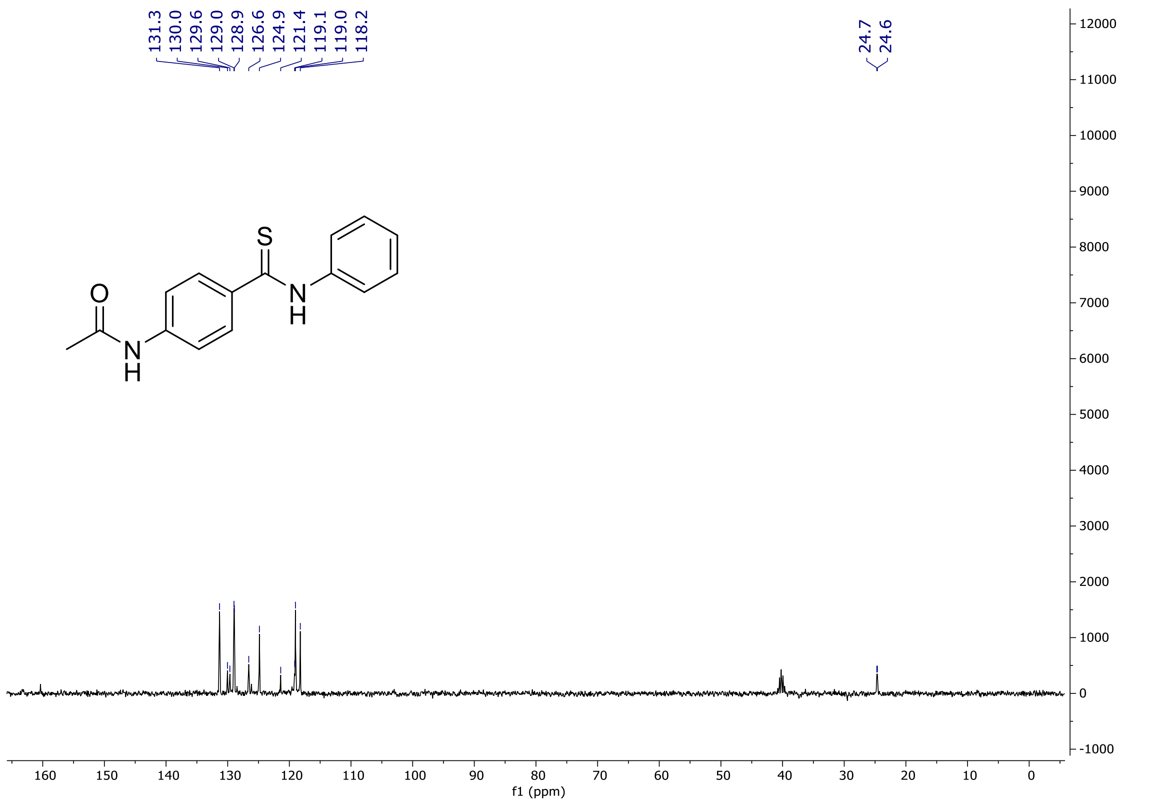

Figure SI_195: DEPT 135-NMR for **3u** in DMSO-*d*^{6} (75 MHz).

Acquisition Parameter

Source Type	ESI	Ion Polarity	Positive	Set Nebulizer	2.4 Bar
Focus	Active	Set Capillary	4000 V	Set Dry Heater	250 °C
Scan Begin	50 m/z	Set End Plate Offset	-500 V	Set Dry Gas	6.0 l/min
Scan End	1500 m/z	Set Charging Voltage	2000 V	Set Divert Valve	Source
		Set Corona	0 nA	Set APCI Heater	0 °C

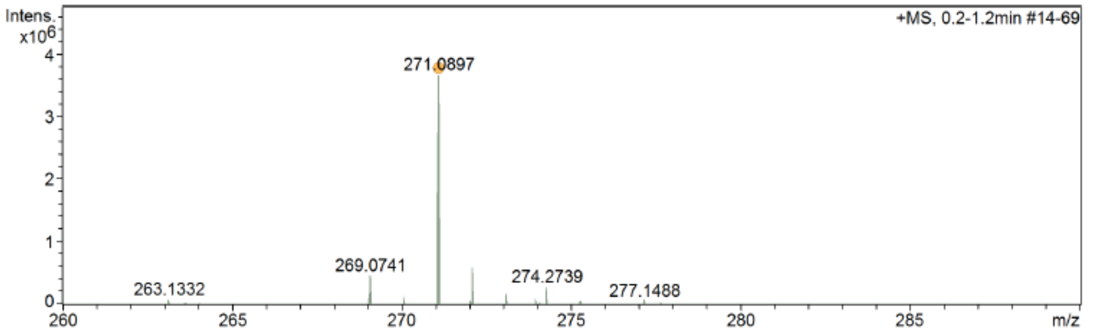

Meas. m/z	#	Ion Formula	m/z	err [ppm]	mSigma	# mSigma	Score	rdb	e¯ Conf	N-Rule	Adduct
271.0897	1	C15H15N2OS	271.0900	1.0	11.0	1	100.00	10.0	even	ok	M+H

Figure SI_196: HRMS (ESI$^+$, m/z) analysis of **3u**.

***N*-Phenylfuran-2-carbothioamide (3v)**

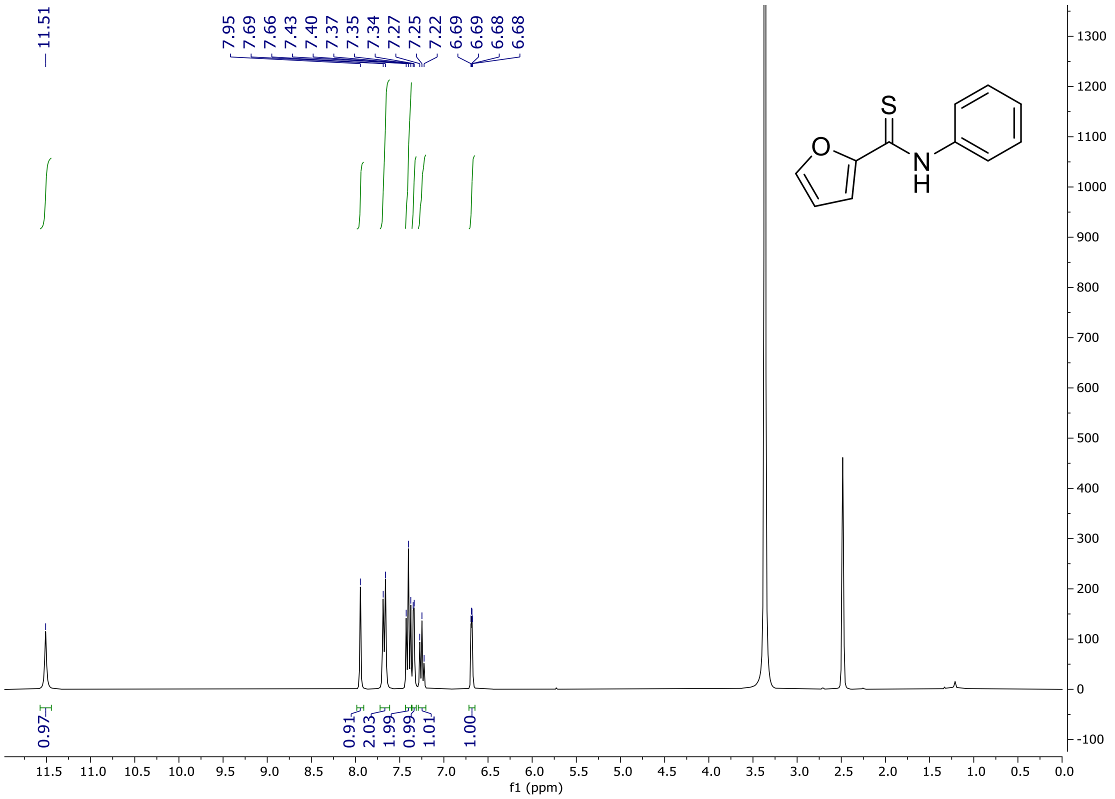

Figure SI_197: ^{1}H-NMR for **3v** in DMSO-d^{6} (300 MHz).

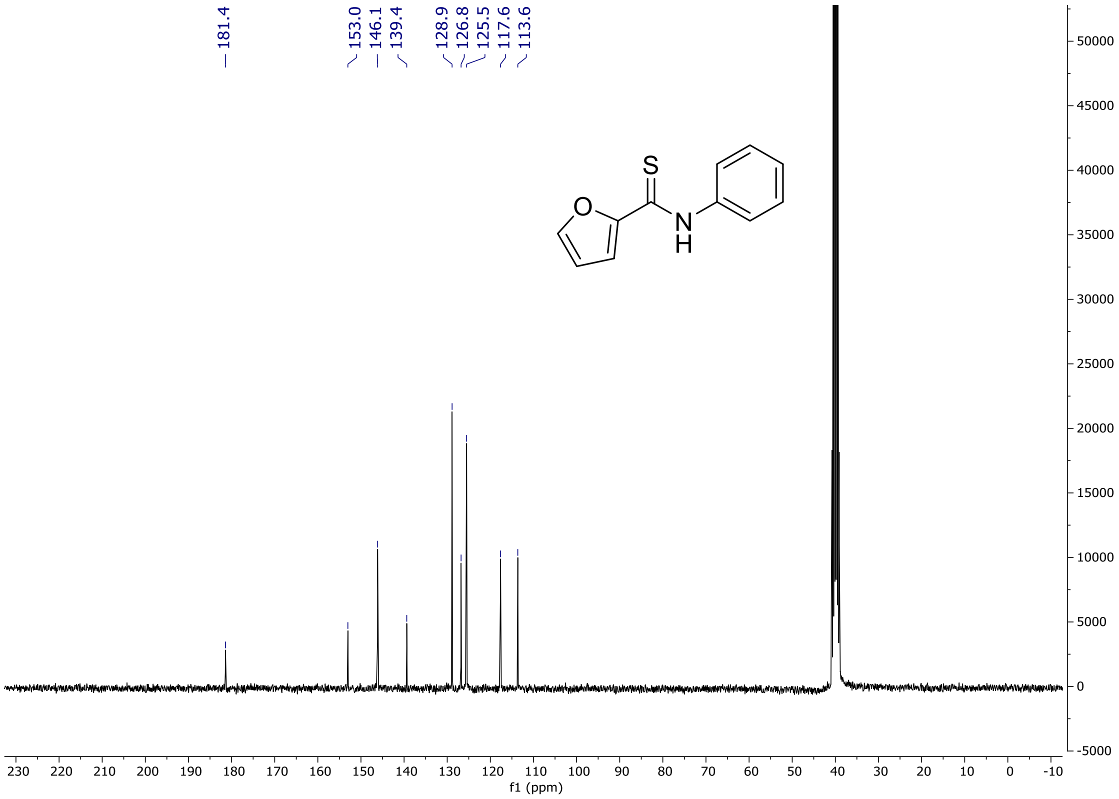

Figure SI_198: ^{13}C-NMR for **3v** in DMSO-d^{6} (75 MHz).

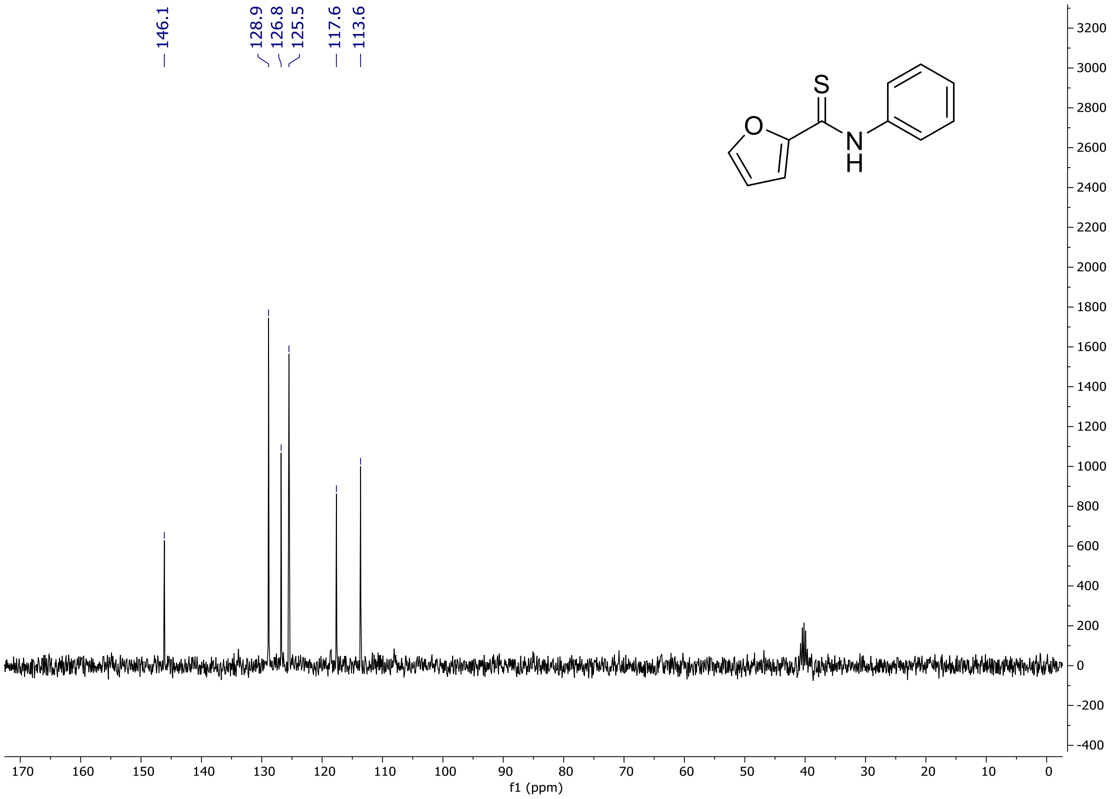

Figure SI_199: DEPT 135-NMR for **3v** in DMSO-d^6 (75 MHz).

***N*-Phenylthiophen-2-carbothioamide (3w)**

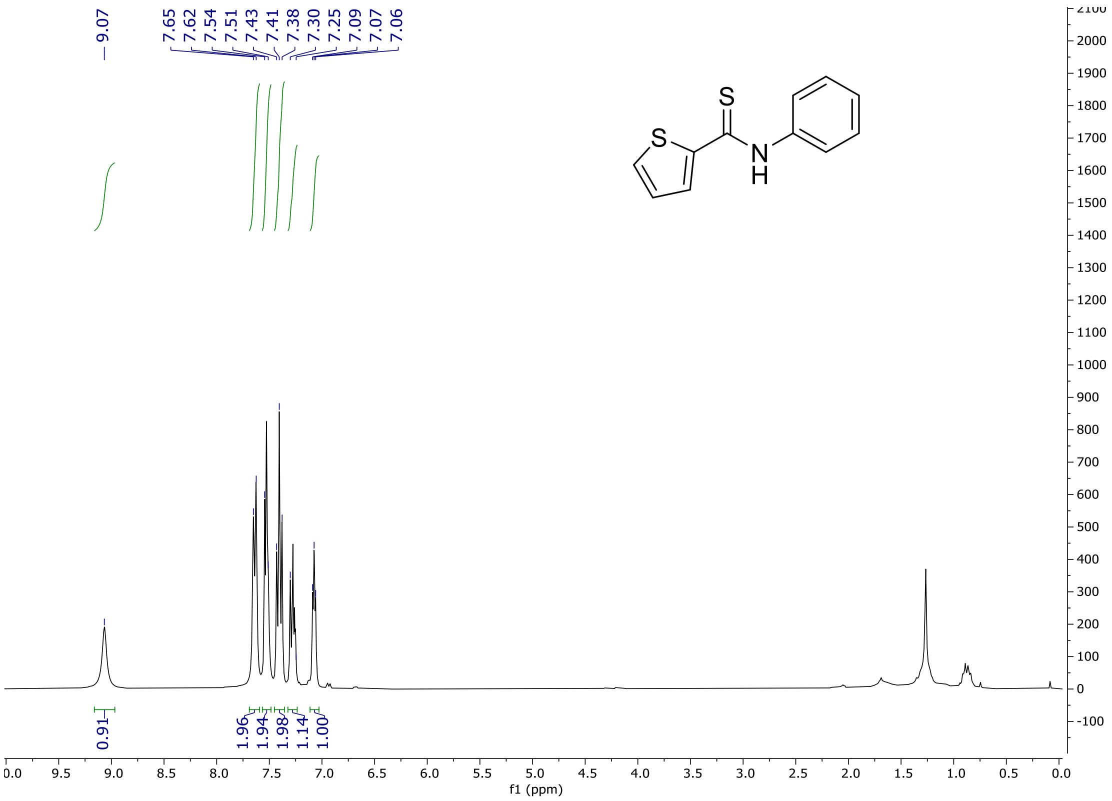

Figure SI_200: ^{1}H-NMR for **3w** in $CDCl_3$ (300 MHz).

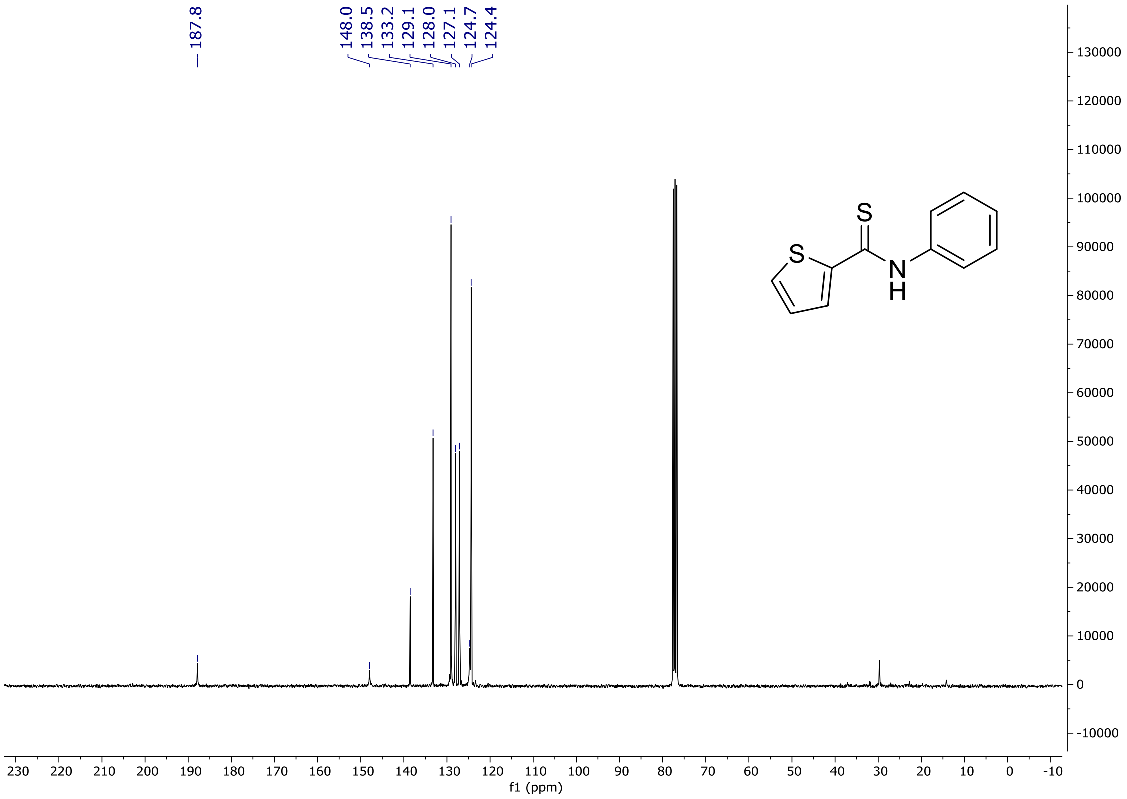

Figure SI_201: ^{13}C-NMR for **3w** in $CDCl_3$ (75 MHz).

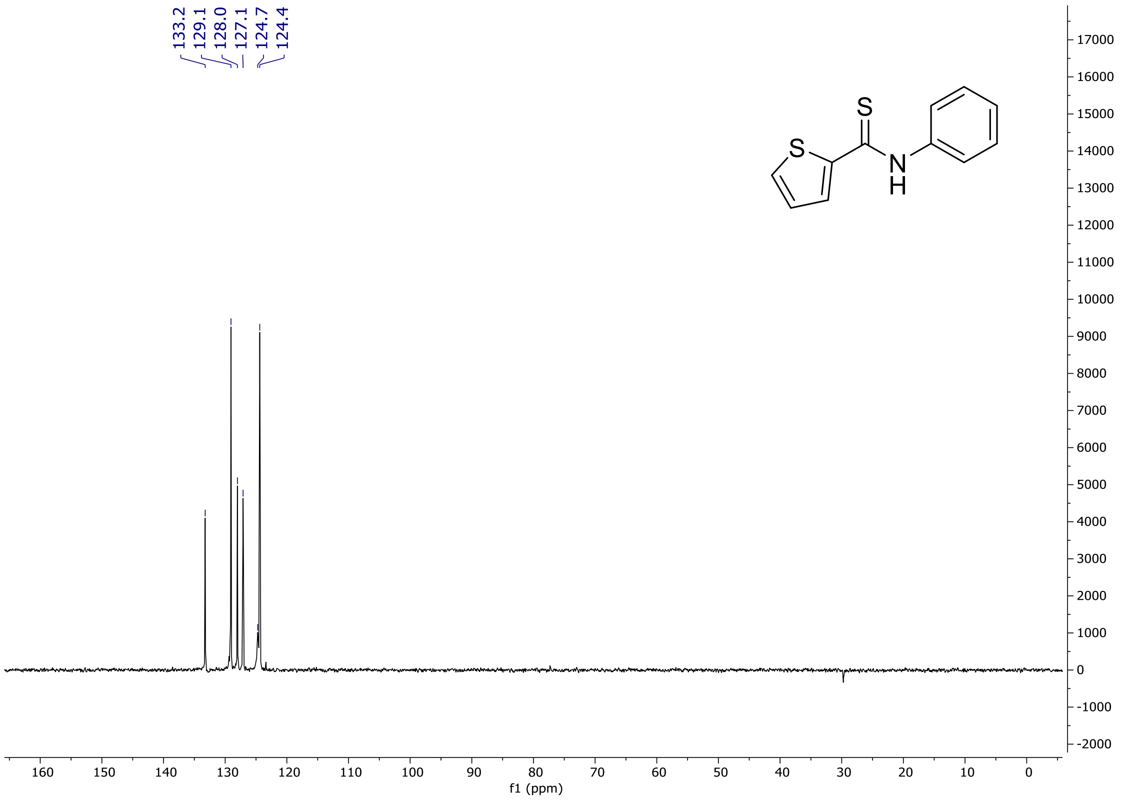

Figure SI_202: DEPT 135-NMR for **3w** in $CDCl_3$ (75 MHz).

***N*-Phenylpyridin-3-carbothioamide (3x)**

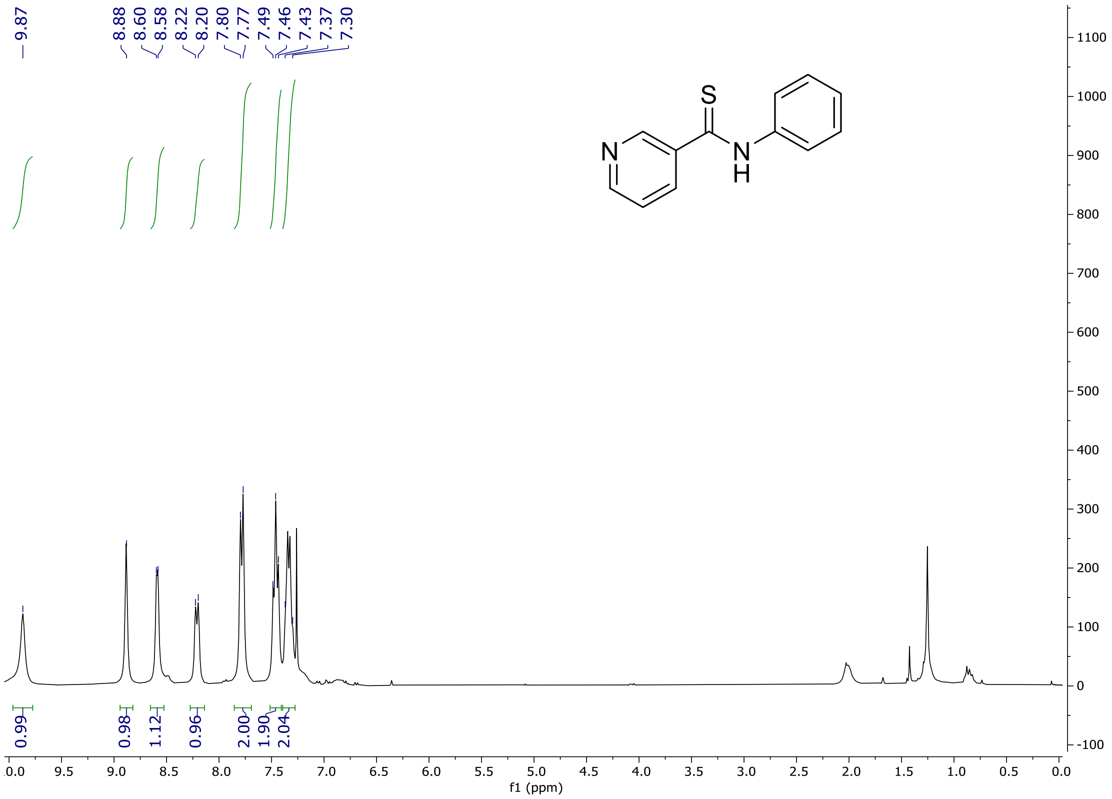

Figure SI_203: ^{1}H-NMR for **3x** in $CDCl_3$ (300 MHz).

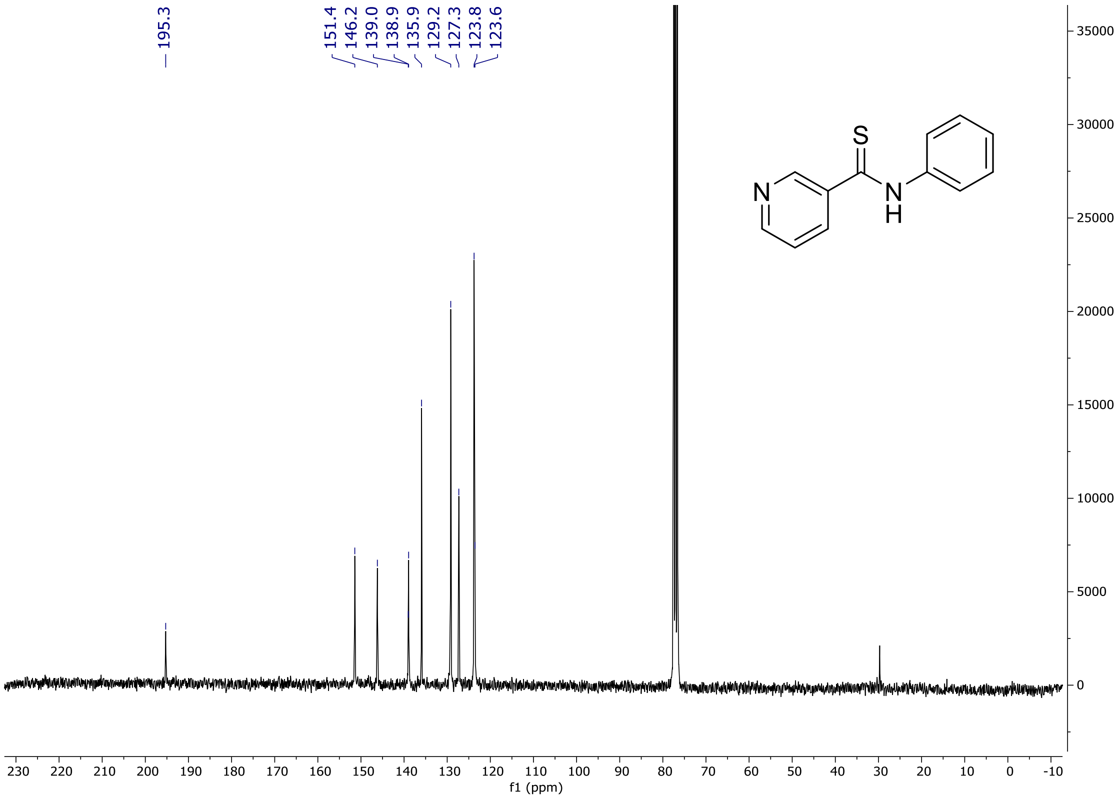

Figure SI_204: ^{13}C-NMR for **3x** in $CDCl_3$ (75 MHz).

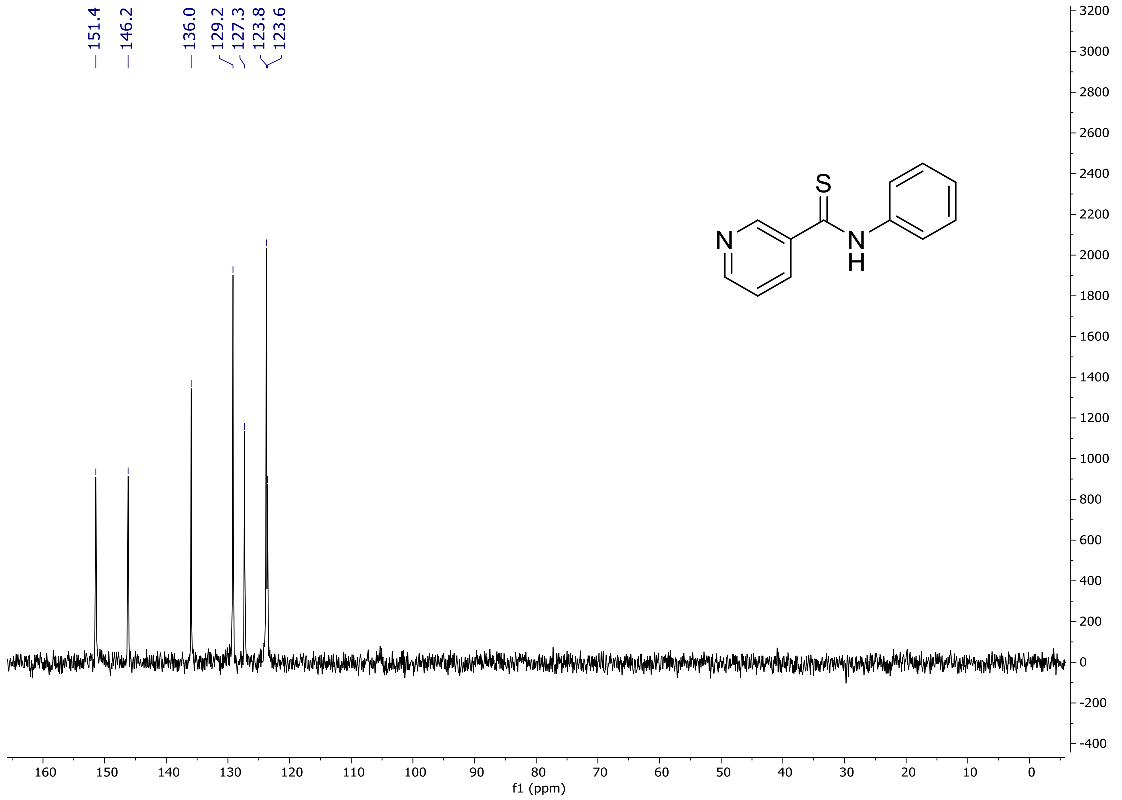

Figure SI_205: DEPT 135-NMR for **3x** in $CDCl_3$ (75 MHz).

Acquisition Parameter

Source Type	ESI	Ion Polarity	Positive	Set Nebulizer	2.4 Bar
Focus	Active	Set Capillary	4000 V	Set Dry Heater	250 °C
Scan Begin	50 m/z	Set End Plate Offset	-500 V	Set Dry Gas	6.0 l/min
Scan End	1500 m/z	Set Charging Voltage	2000 V	Set Divert Valve	Source
		Set Corona	0 nA	Set APCI Heater	0 °C

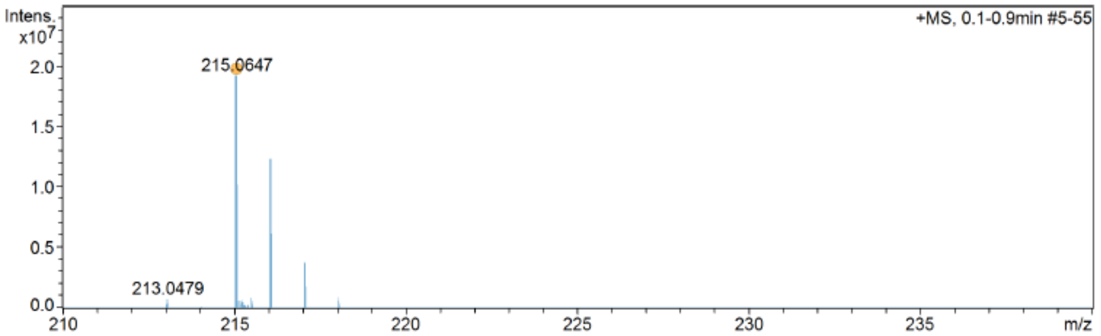

Meas. m/z	#	Ion Formula	m/z	err [ppm]	mSigma	# mSigma	Score	rdb	e⁻ Conf	N-Rule	Adduct
215.0647	1	C12H11N2S	215.0637	-4.2	258.1	1	100.00	9.0	even	ok	M+H

Figure SI_206: HRMS (ESI^+, m/z) analysis of **3x**.

N-Phenylnaphthalen-1-carbothioamide (3y)

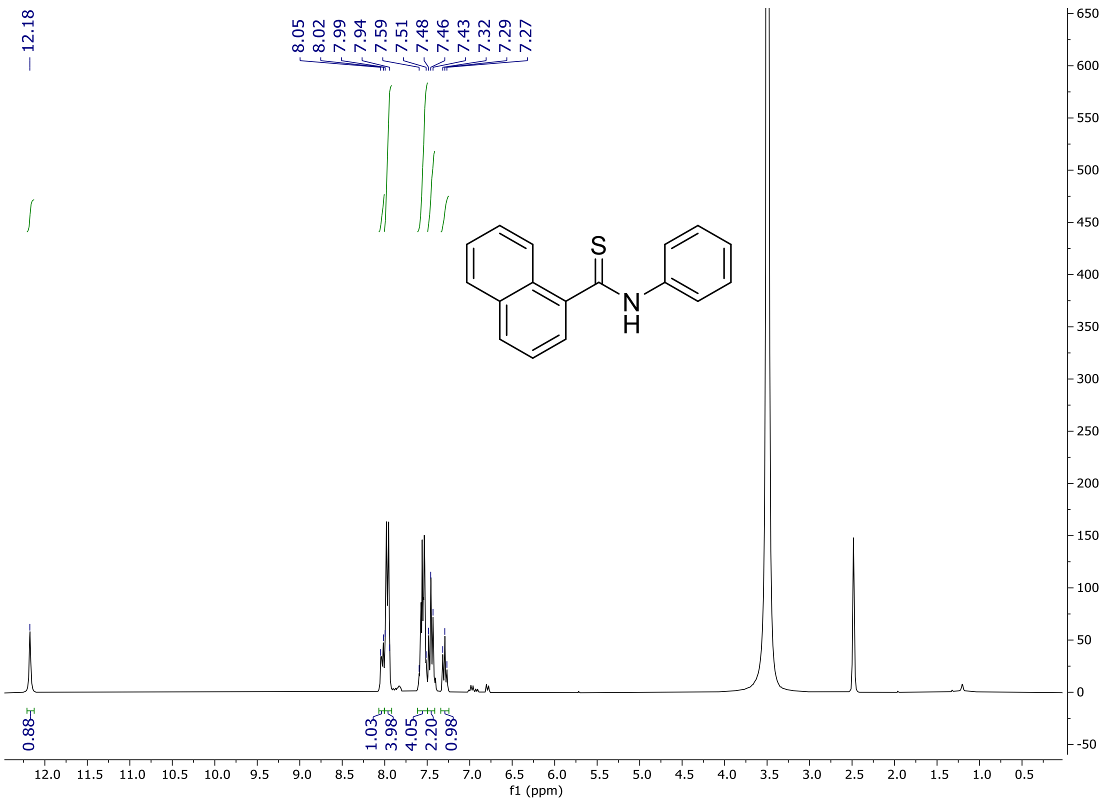

Figure SI_207: ^{1}H-NMR for **3y** in DMSO-d^{6} (300 MHz).

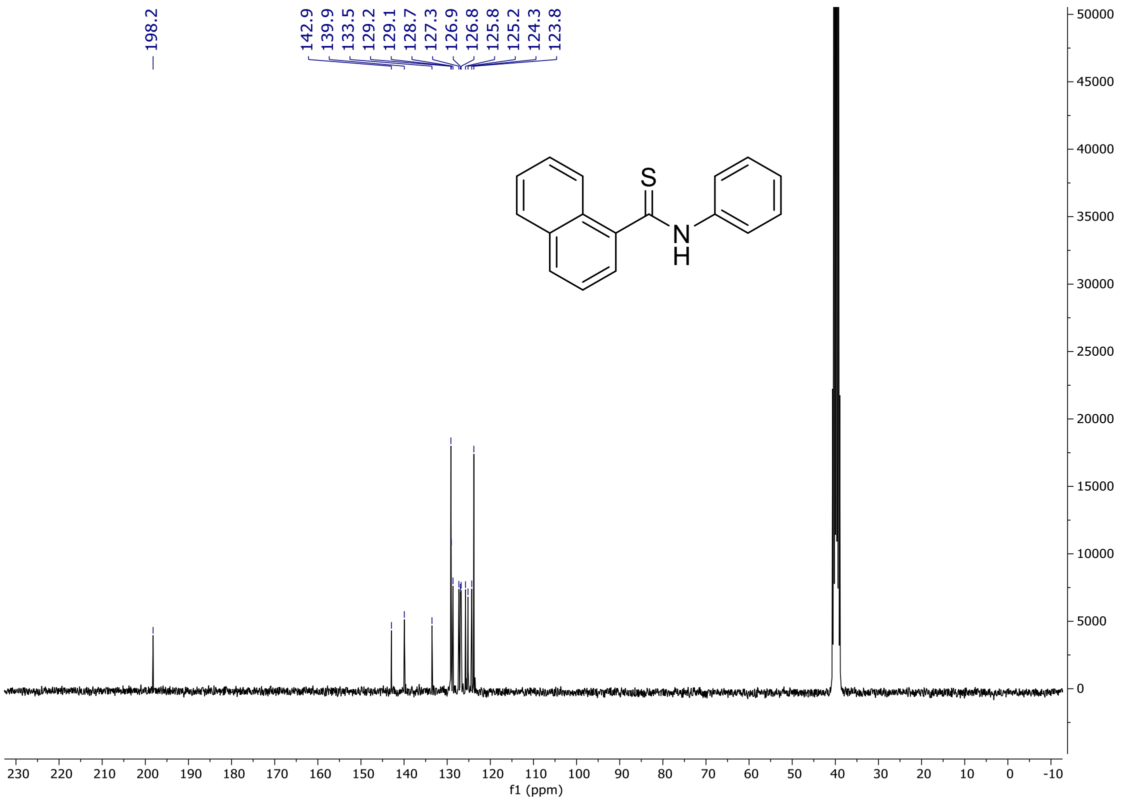

Figure SI_208: ^{13}C-NMR for **3y** in DMSO-d^{6} (75 MHz).

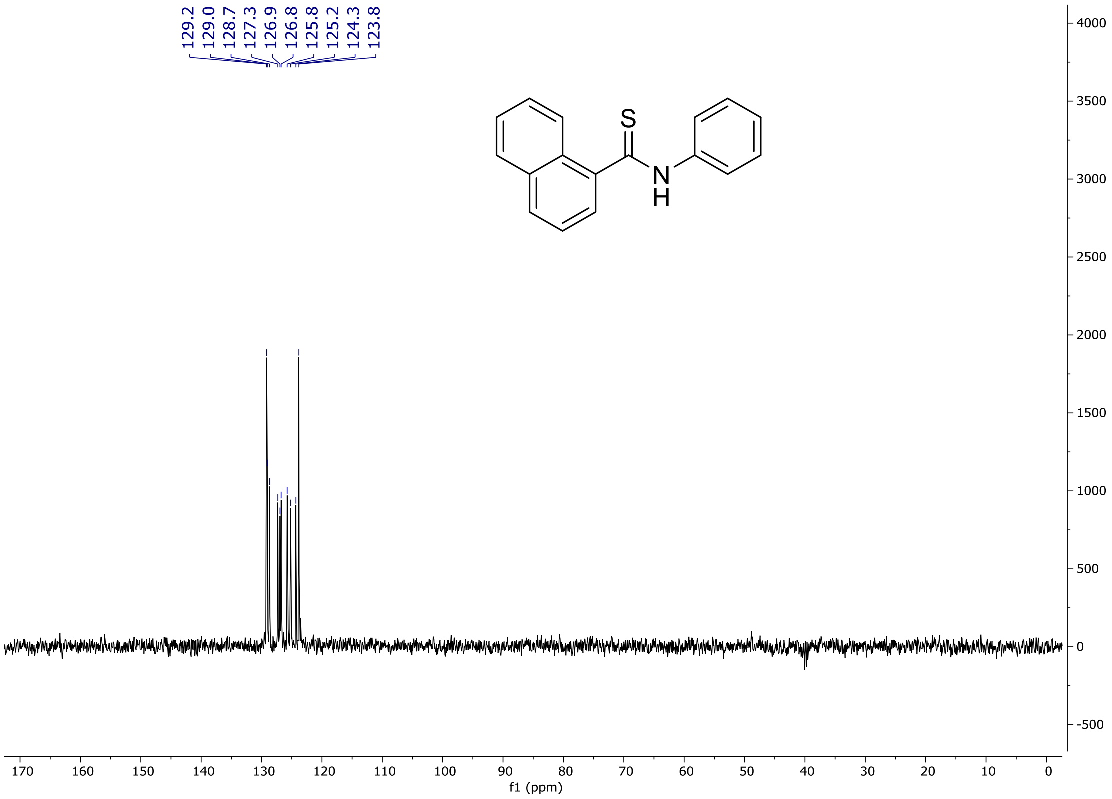

Figure SI_209: DEPT 135-NMR for **3y** in DMSO-d^6 (75 MHz).

Acquisition Parameter

Source Type	ESI	Ion Polarity	Positive	Set Nebulizer	2.4 Bar
Focus	Active	Set Capillary	4000 V	Set Dry Heater	250 °C
Scan Begin	50 m/z	Set End Plate Offset	-500 V	Set Dry Gas	6.0 l/min
Scan End	1500 m/z	Set Charging Voltage	2000 V	Set Divert Valve	Source
		Set Corona	0 nA	Set APCI Heater	0 °C

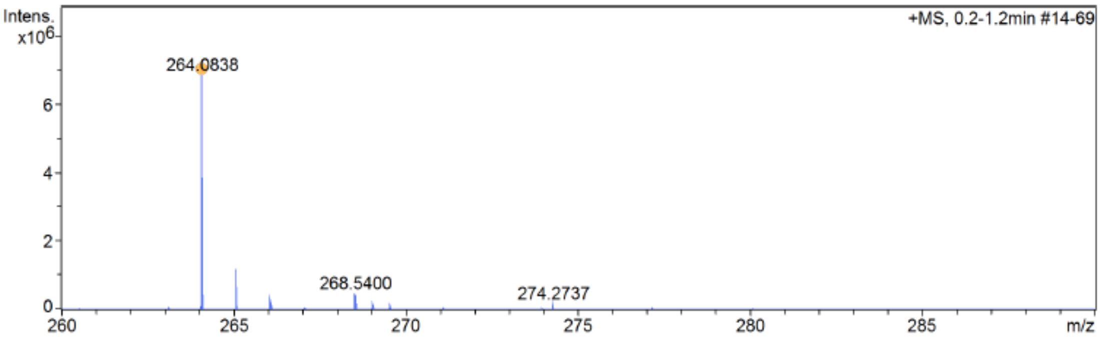

Meas. m/z	#	Ion Formula	m/z	err [ppm]	mSigma	# mSigma	Score	rdb	e¯ Conf	N-Rule	Adduct
264.0838	1	C17H14NS	264.0841	1.3	14.4	1	100.00	12.0	even	ok	M+H

Figure SI_210: HRMS (ESI$^+$, m/z) analysis of **3y**.

(*E*)-*N*-Phenylcinnamthioamide (3z)

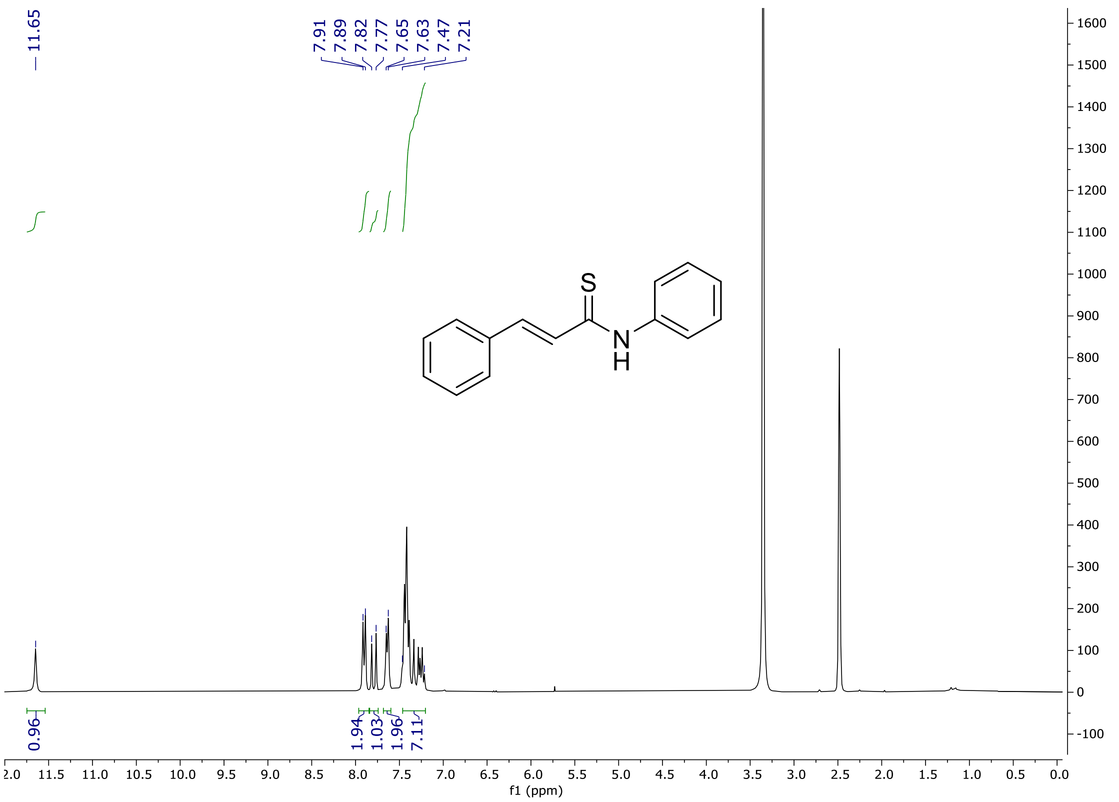

Figure SI_211: ^{1}H-NMR for **3z** in DMSO-d^{6} (300 MHz).

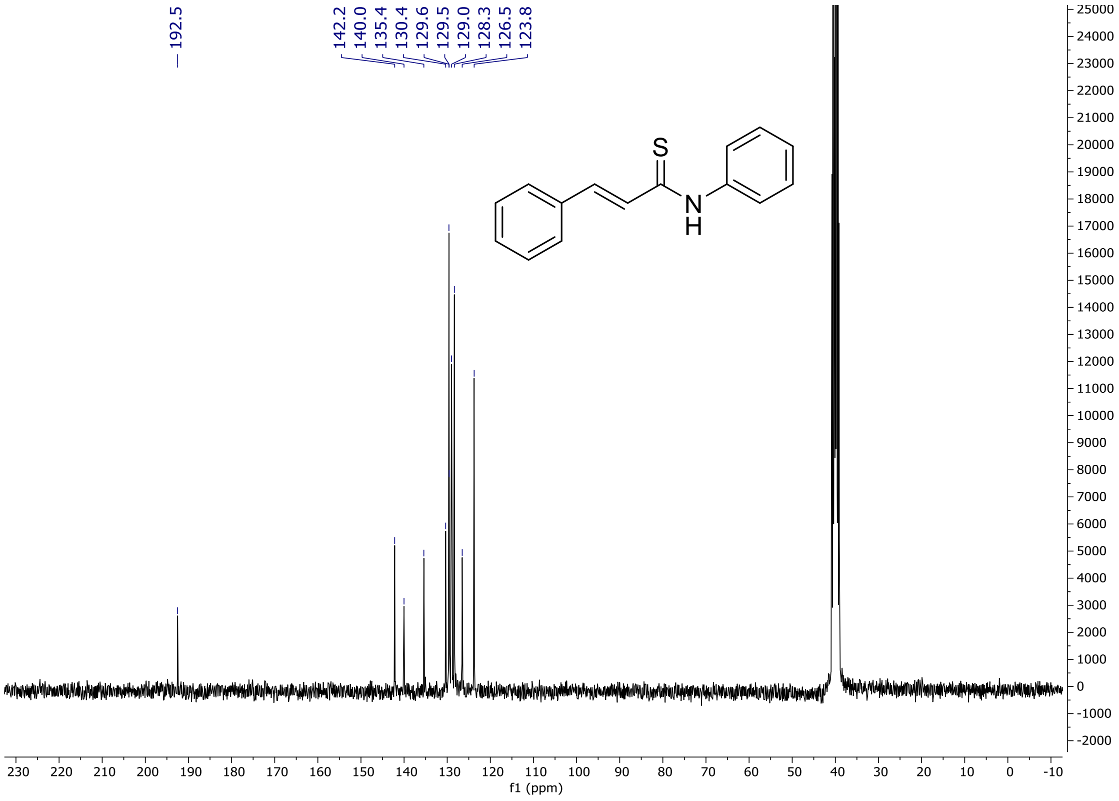

Figure SI_212: ^{13}C-NMR for **3z** in DMSO-d^{6} (75 MHz).

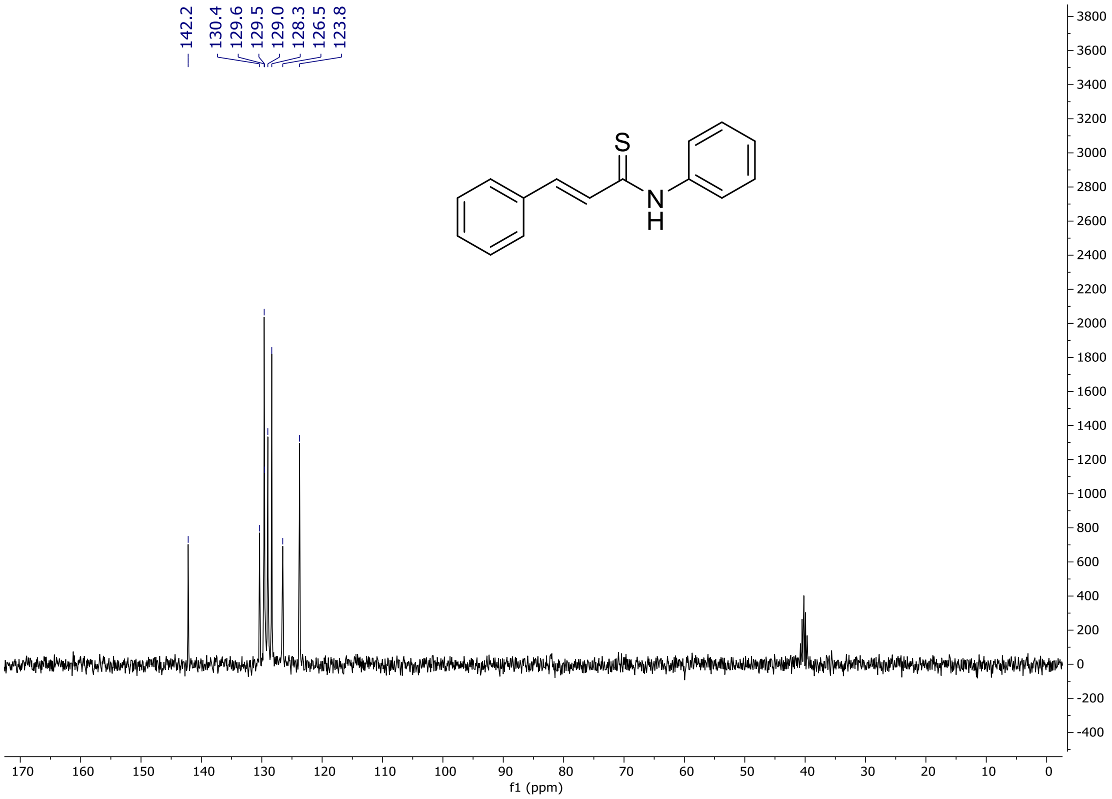

Figure SI_213: DEPT 135-NMR for **3z** in DMSO-d^6 (75 MHz).

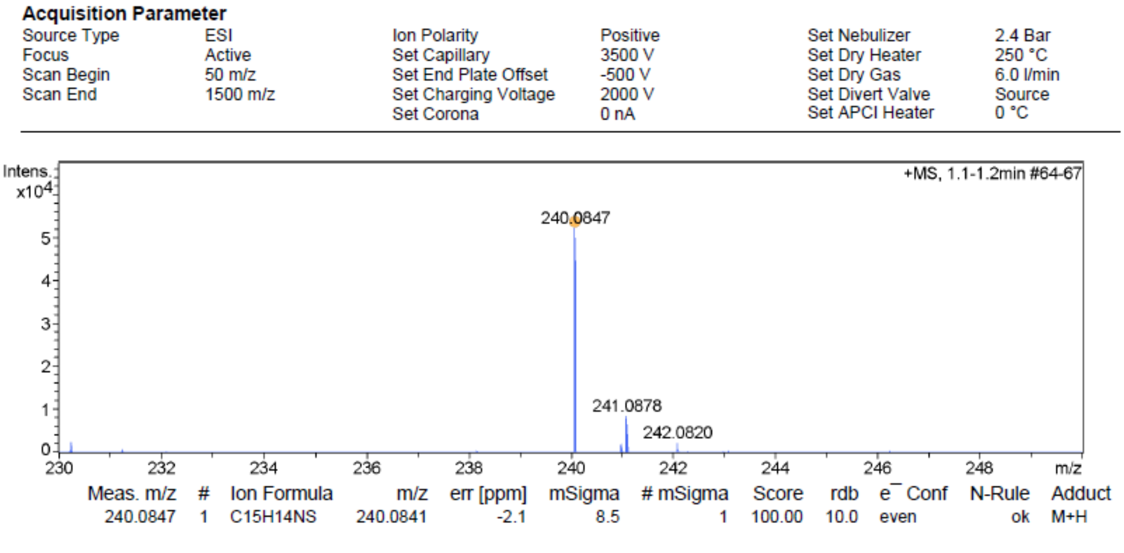

Figure SI_214: HRMS (ESI$^+$, m/z) analysis of **3z**.

2-Methyl-*N*-phenylpropanthioamide (3aa)

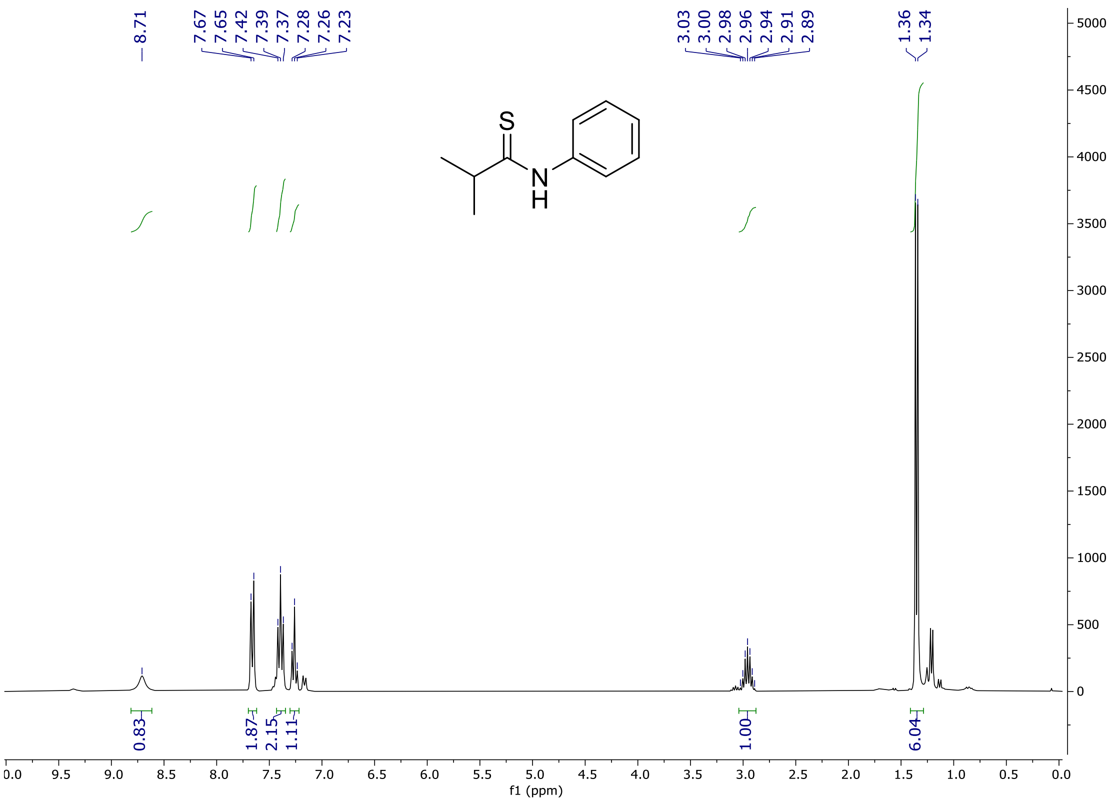

Figure SI_215: ^{1}H-NMR for **3aa** in $CDCl_3$ (300 MHz).

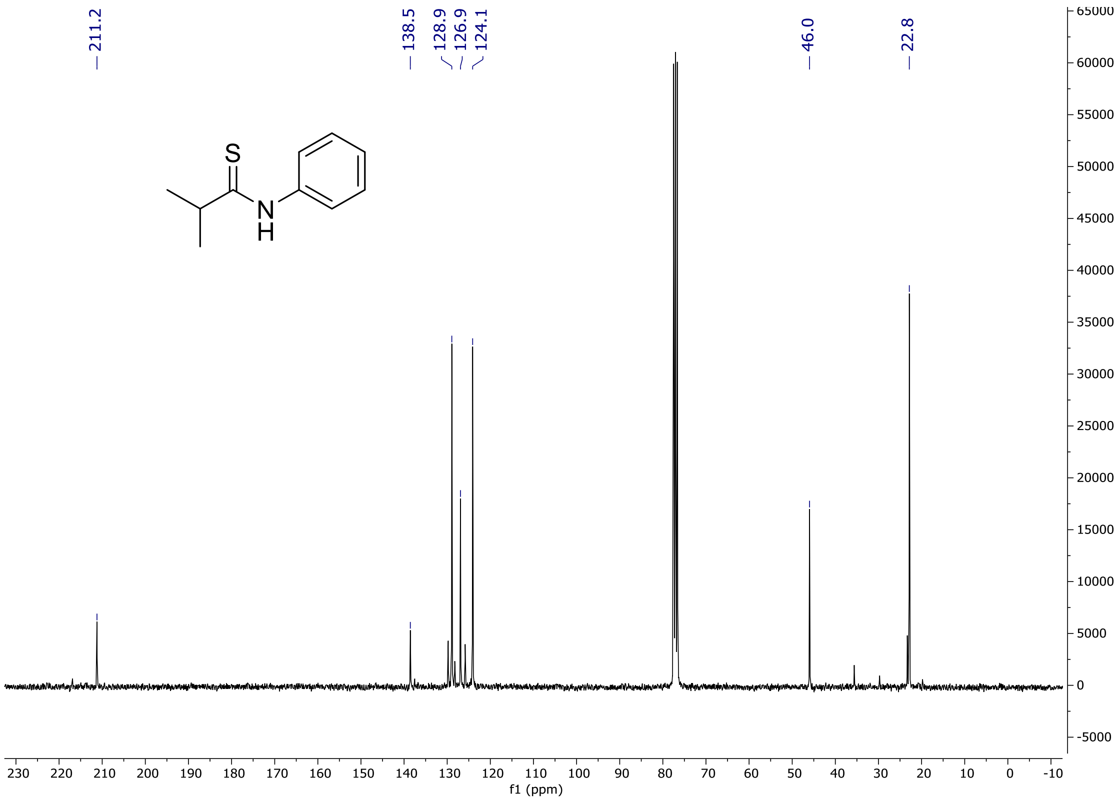

Figure SI_216: ^{13}C-NMR for **3aa** in $CDCl_3$ (75 MHz).

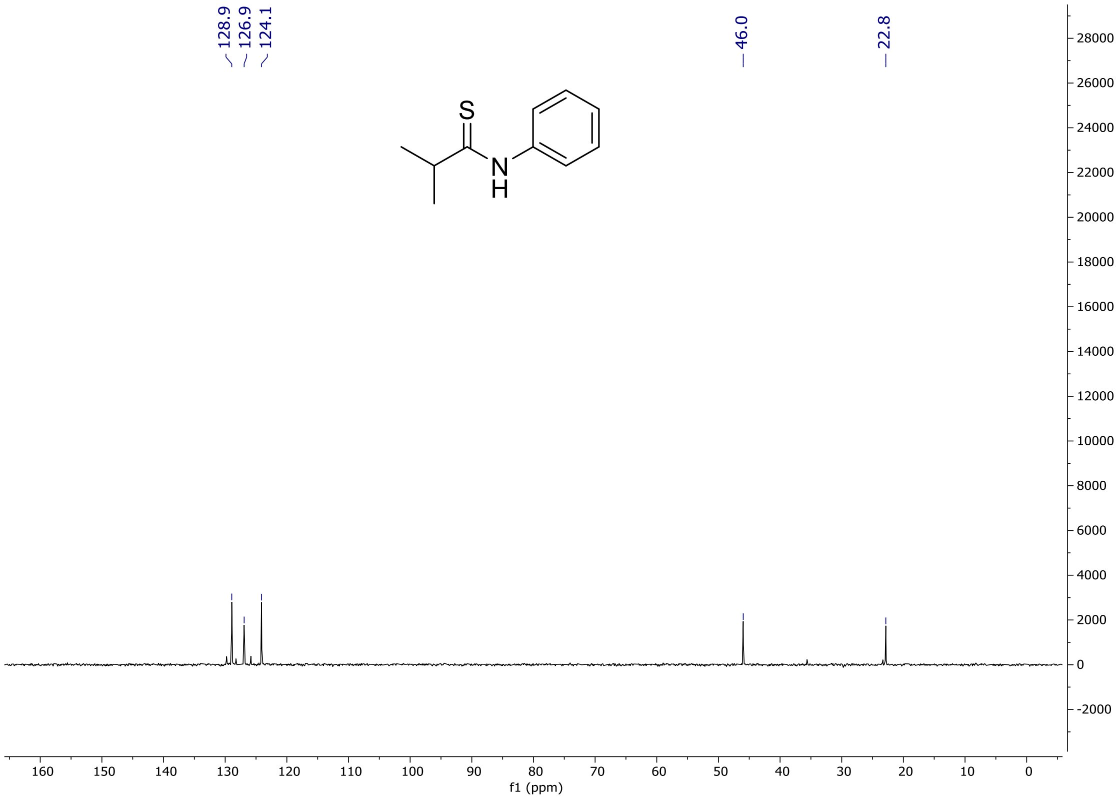

Figure SI_217: DEPT 135-NMR for **3aa** in $CDCl_3$ (75 MHz).

***N*-(4-Fluorophenyl)benzothioamide (3ab)**

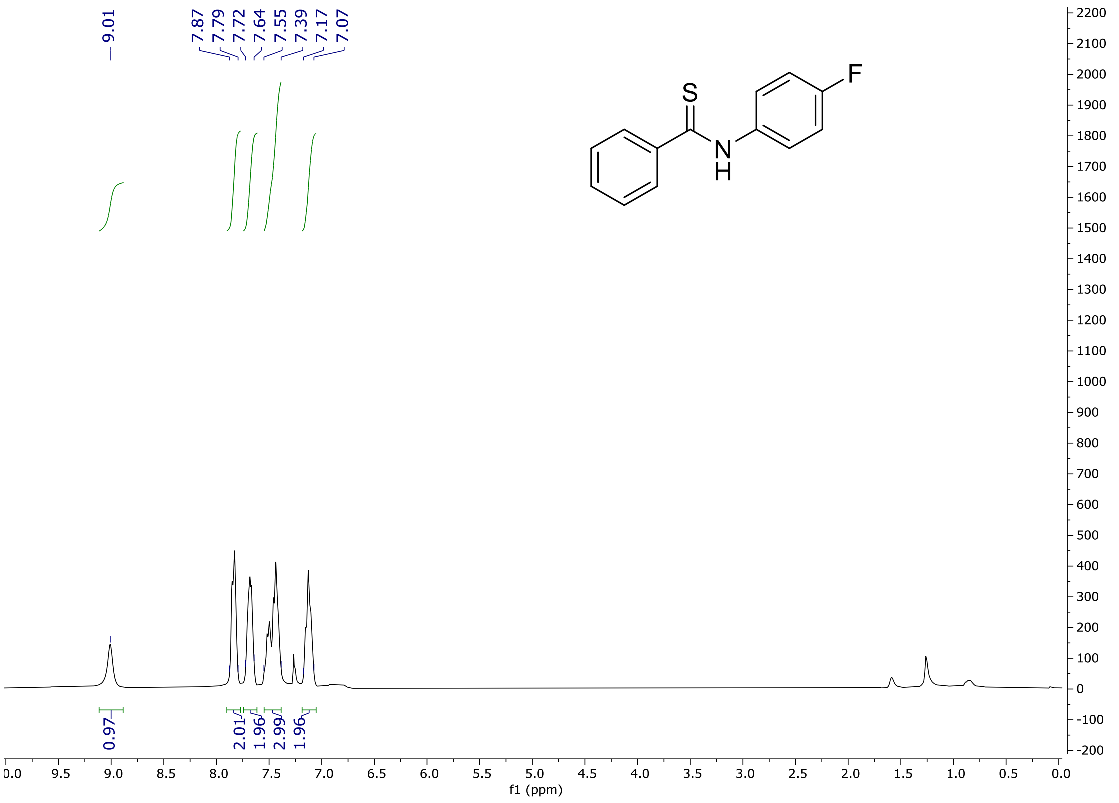

Figure SI_218: ^{1}H-NMR for **3ab** in $CDCl_3$ (300 MHz).

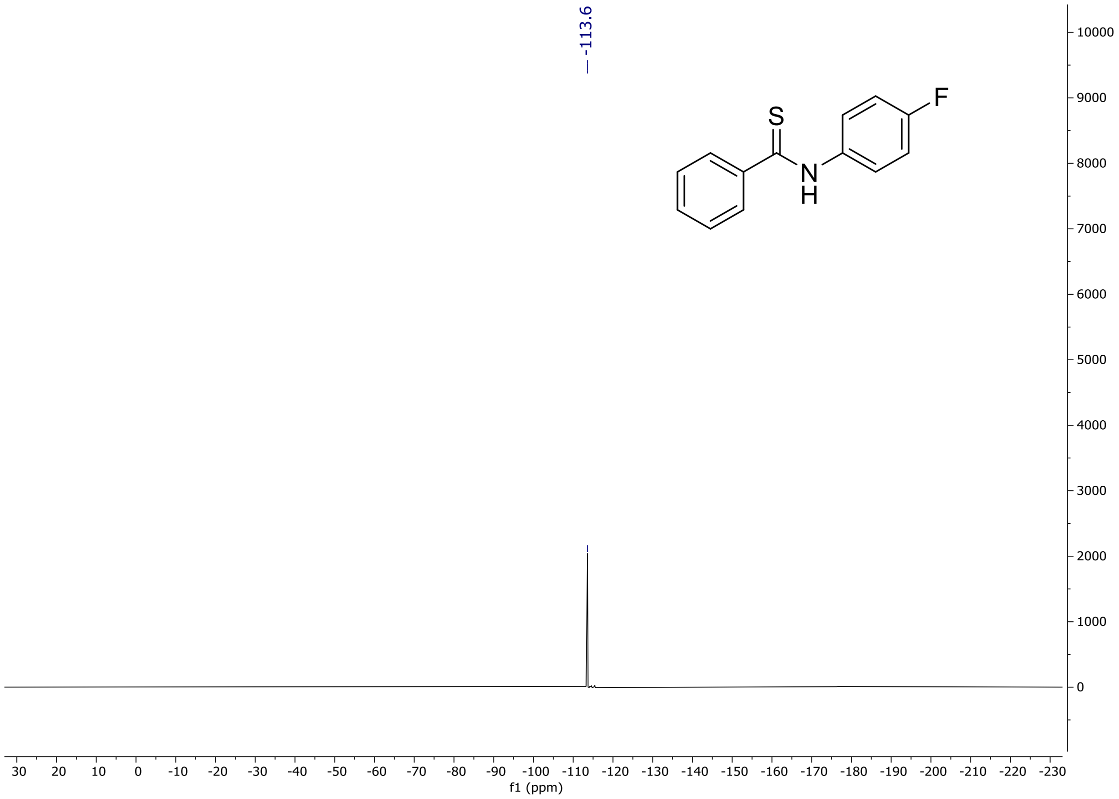

Figure SI_219: ^{19}F-NMR for **3ab** in $CDCl_3$ (282 MHz).

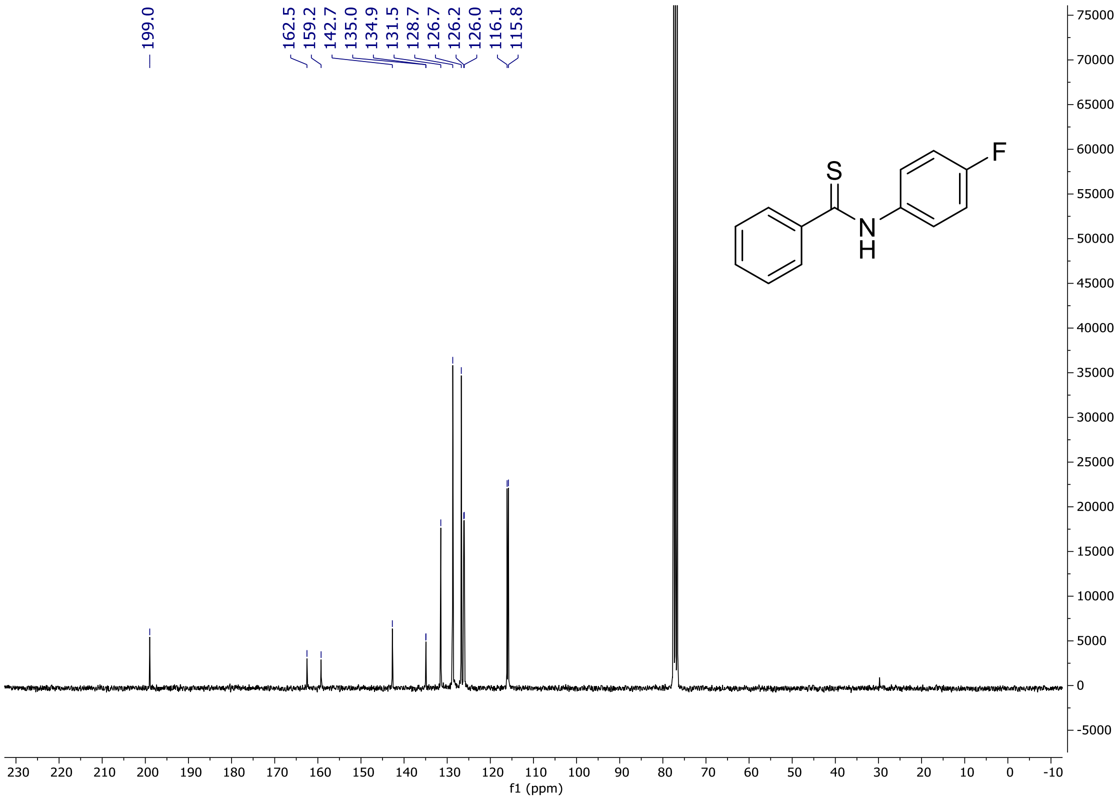

Figure SI_220: ^{13}C-NMR for **3ab** in $CDCl_3$ (75 MHz).

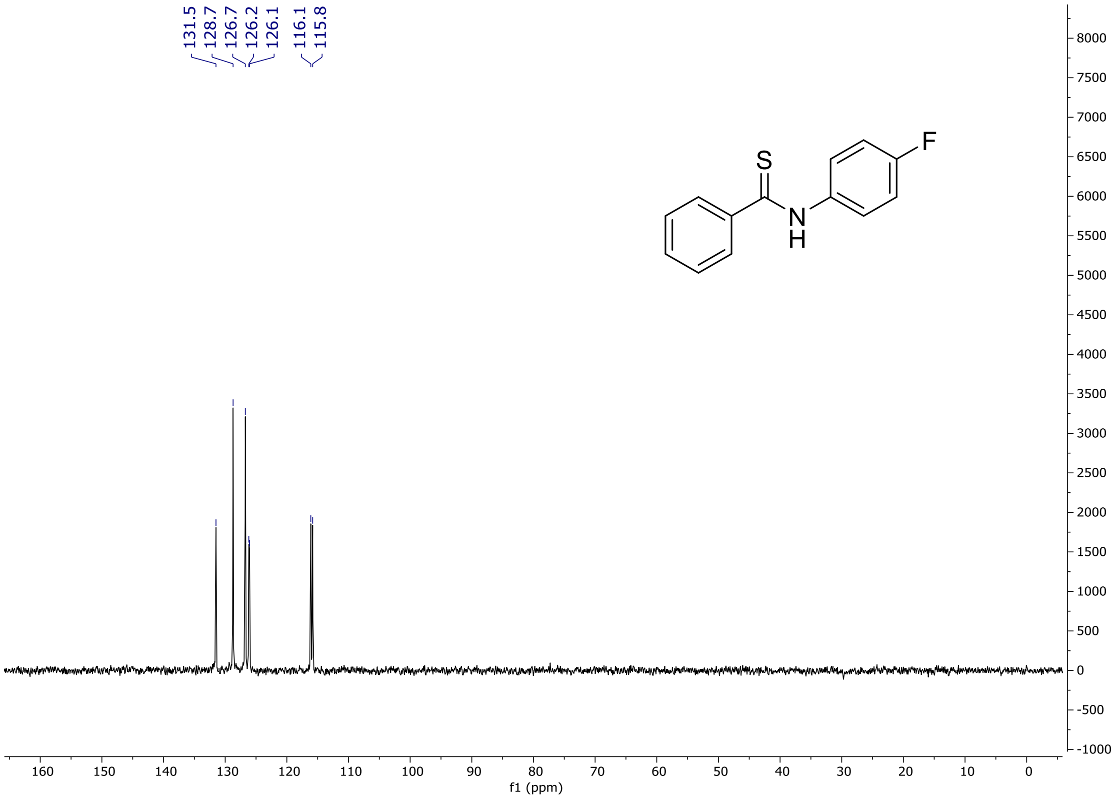

Figure SI_221: DEPT 135-NMR for **3ab** in $CDCl_3$ (75 MHz).

***N*-(4-Bromophenyl)benzothioamide (3ac)**

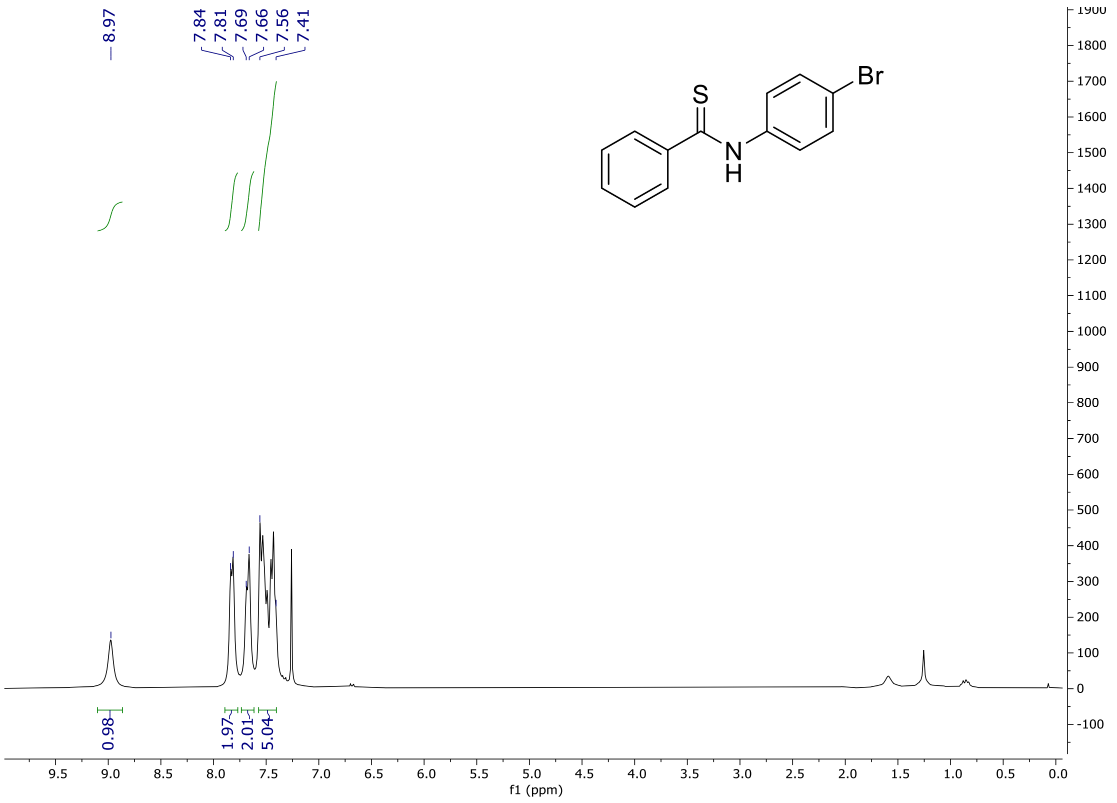

Figure SI_222: ^{1}H-NMR for **3ac** in $CDCl_3$ (300 MHz).

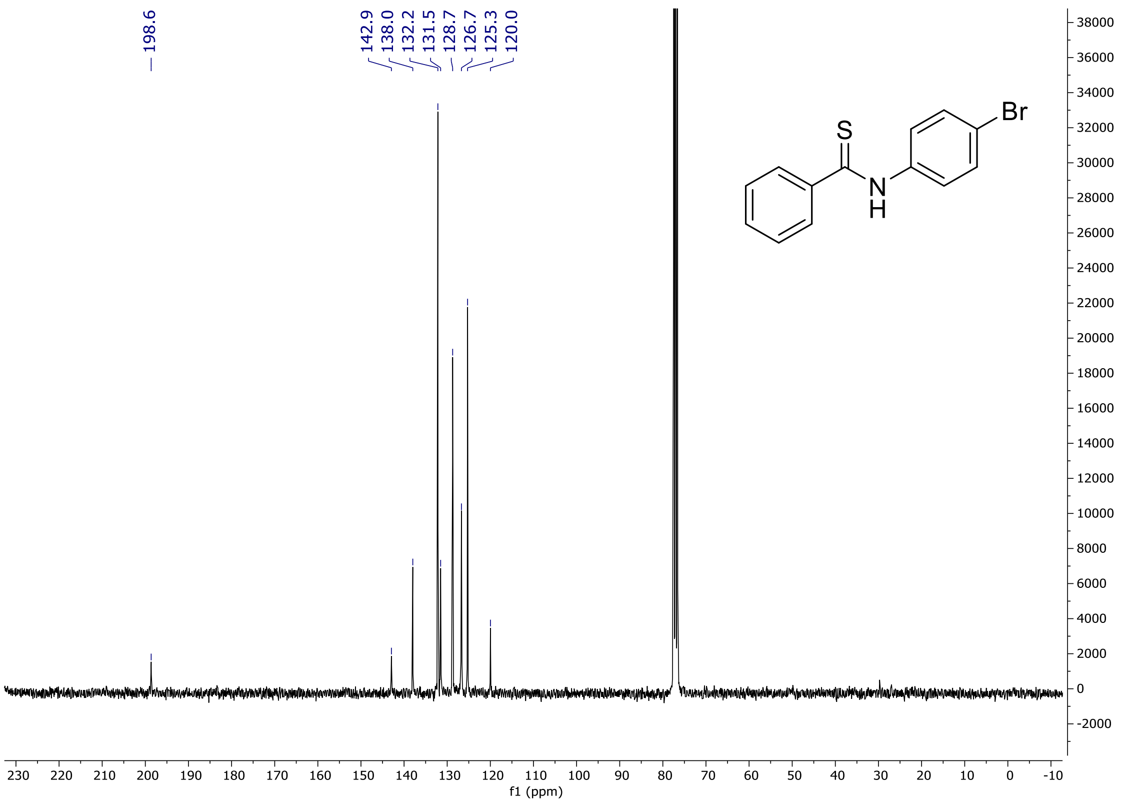

Figure SI_223: ^{13}C-NMR for **3ac** in $CDCl_3$ (75 MHz).

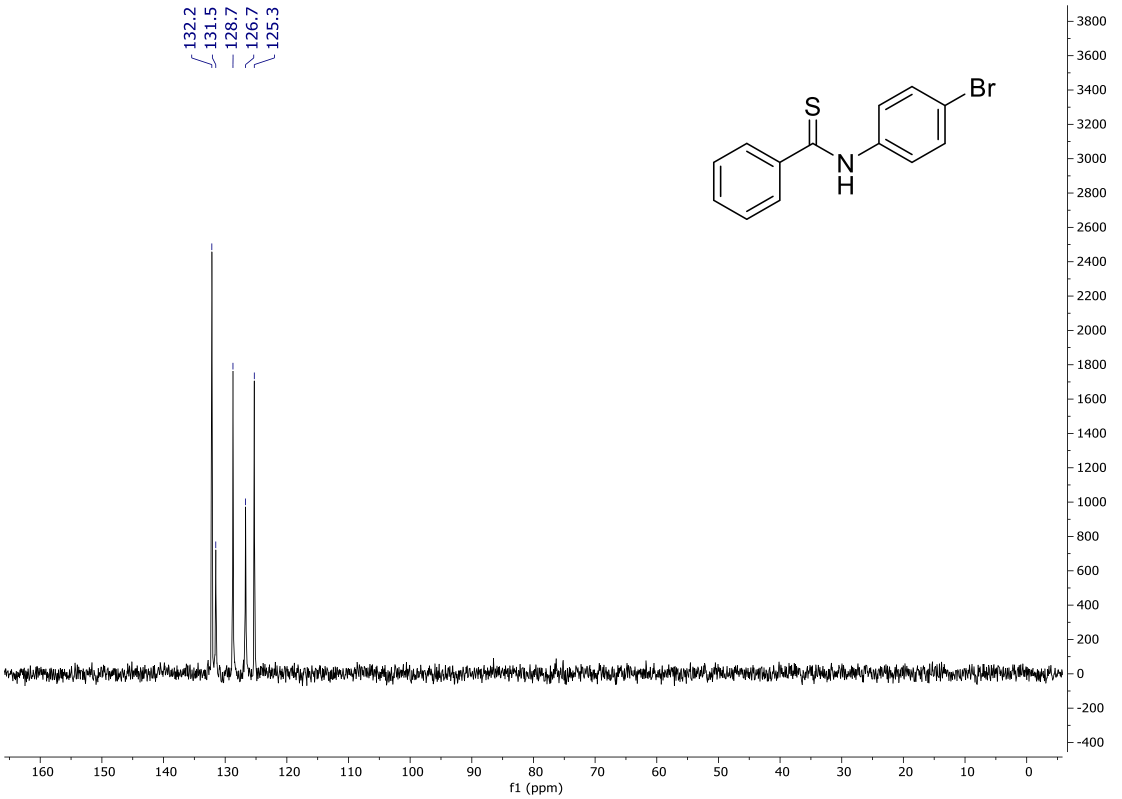

Figure SI_224: DEPT 135-NMR for **3ac** in $CDCl_3$ (75 MHz).

N-Methylbenzothioamide (3ad)

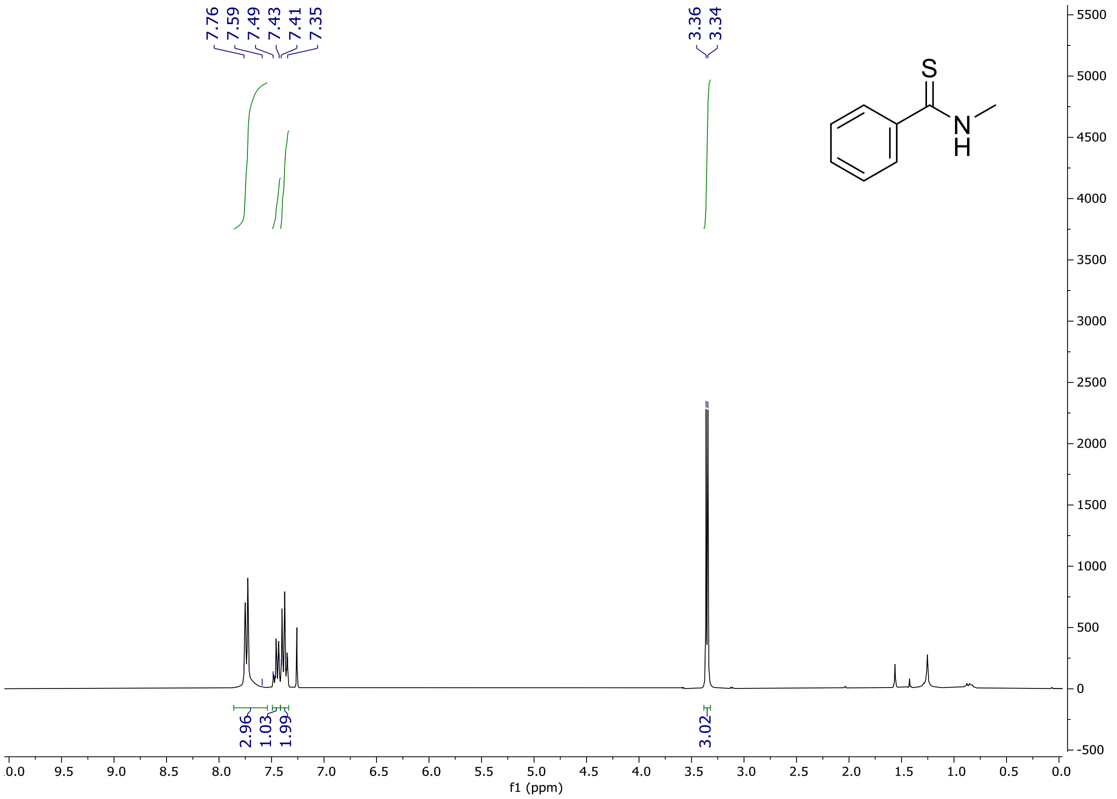

Figure SI_225: ^{1}H-NMR for **3ad** in $CDCl_3$ (300 MHz).

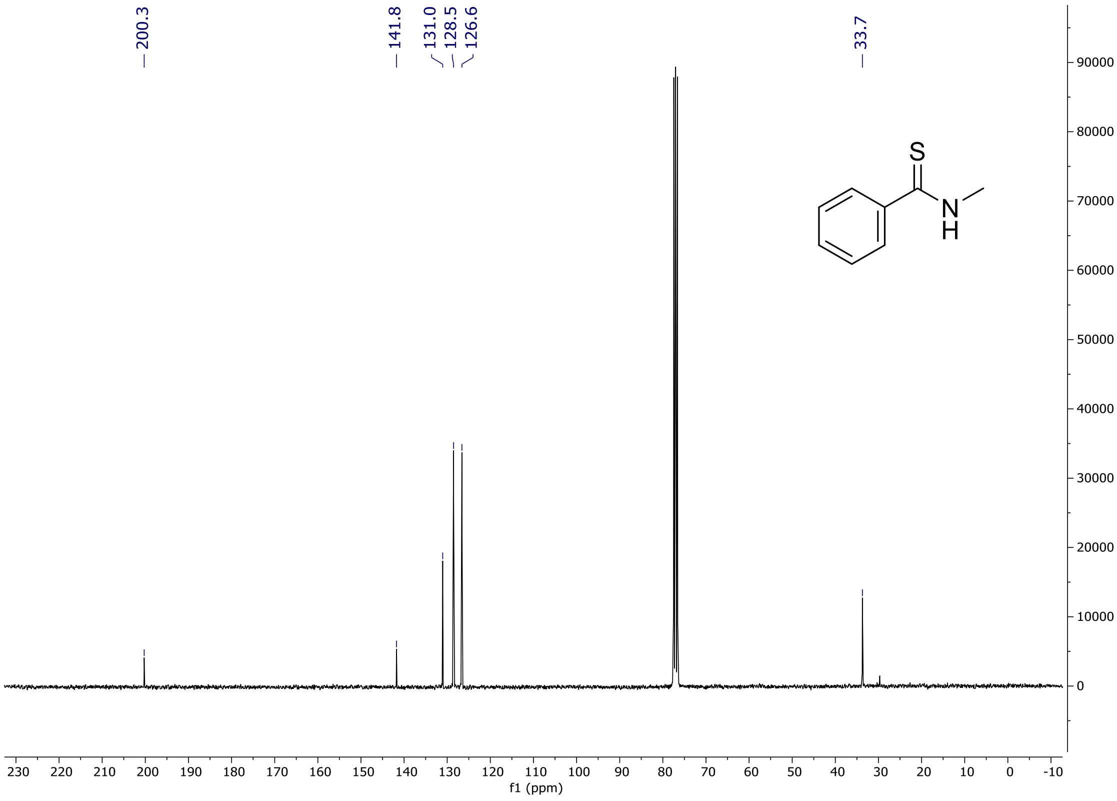

Figure SI_226: ^{13}C-NMR for **3ad** in $CDCl_3$ (75 MHz).

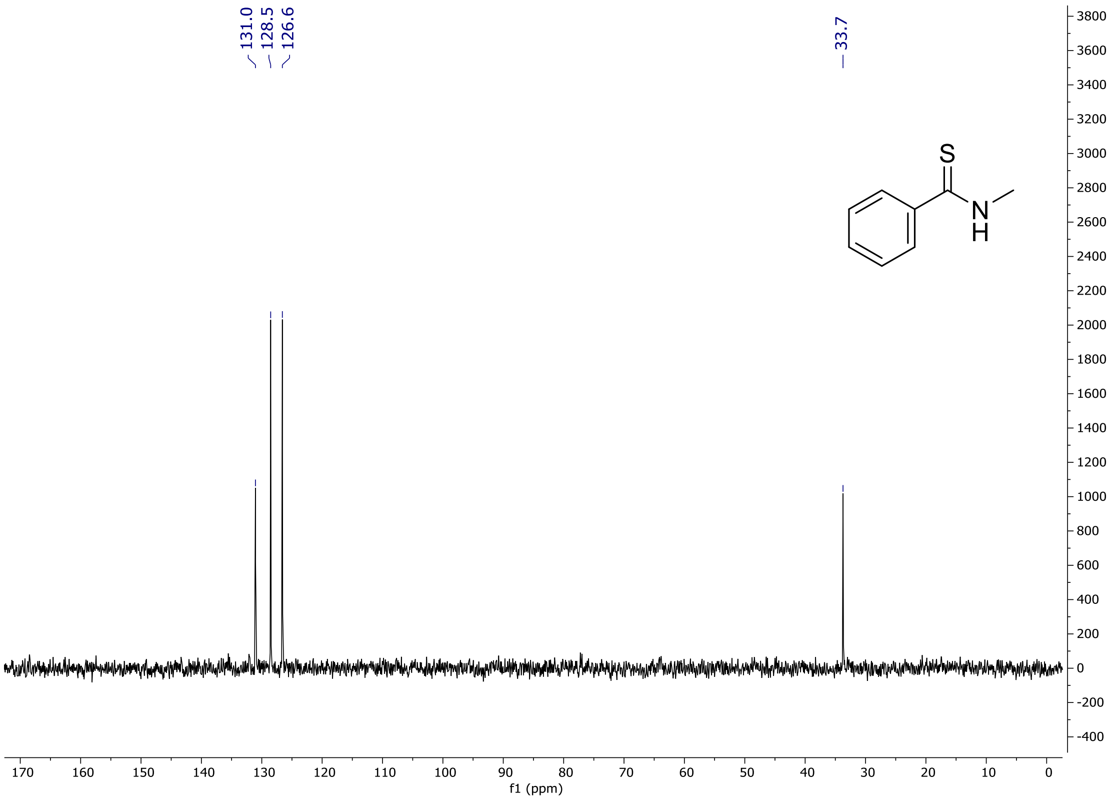

Figure SI_227: DEPT 135-NMR for **3ad** in $CDCl_3$ (75 MHz).

***N*-Benzylbenzothioamide (3ae)**

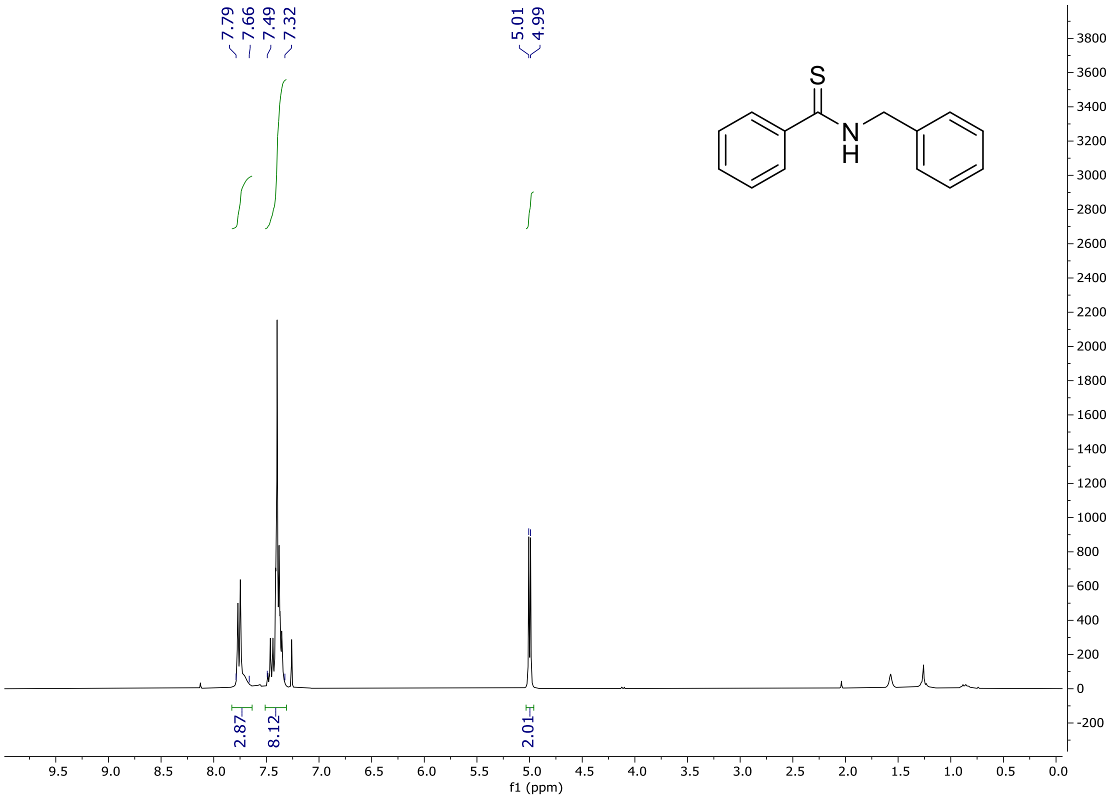

Figure SI_228: ^{1}H-NMR for **3ae** in $CDCl_3$ (300 MHz).

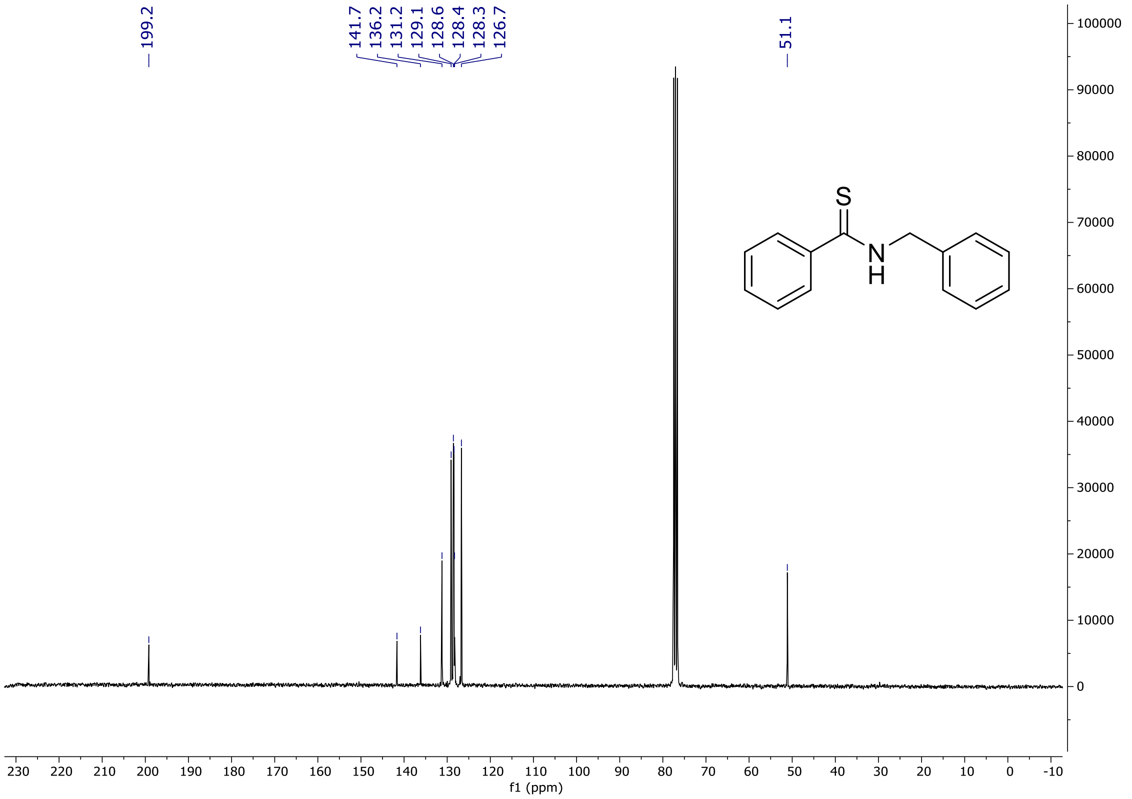

Figure SI_229: ^{13}C-NMR for **3ae** in $CDCl_3$ (75 MHz).

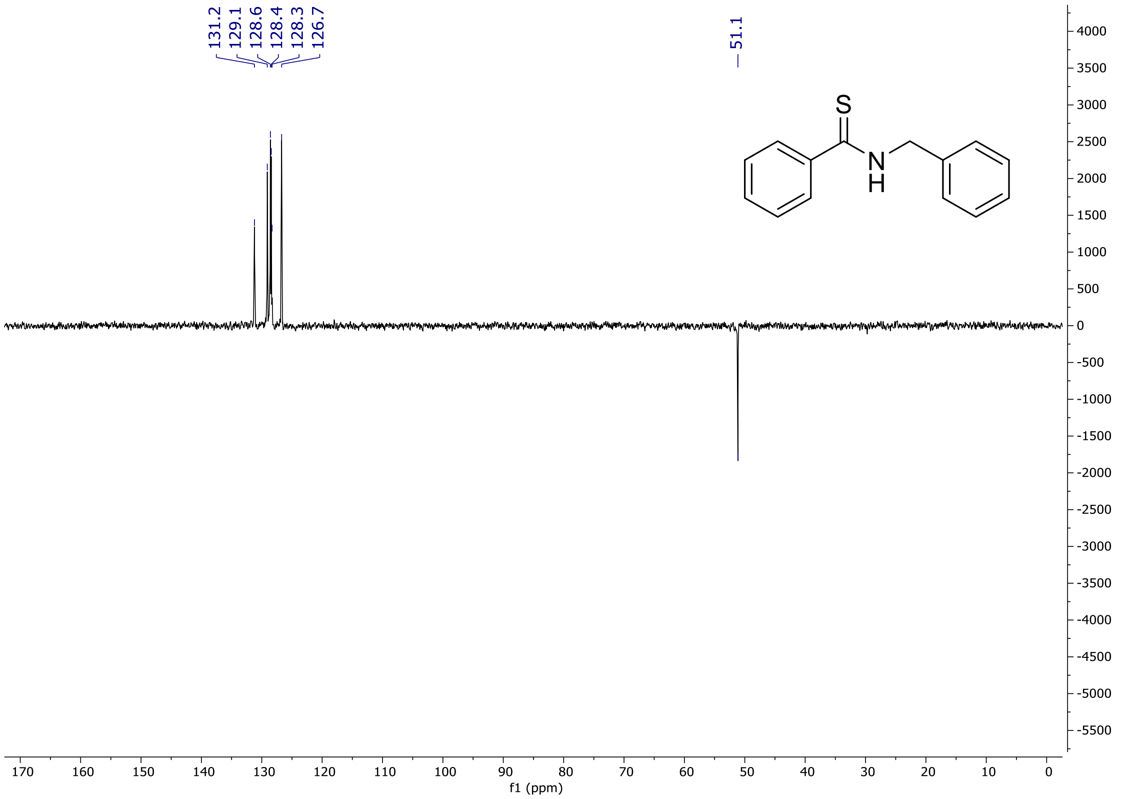

Figure SI_230: DEPT 135-NMR for **3ae** in $CDCl_3$ (75 MHz).

3,4-Dihydroisoquinoline-1-thione (3af)

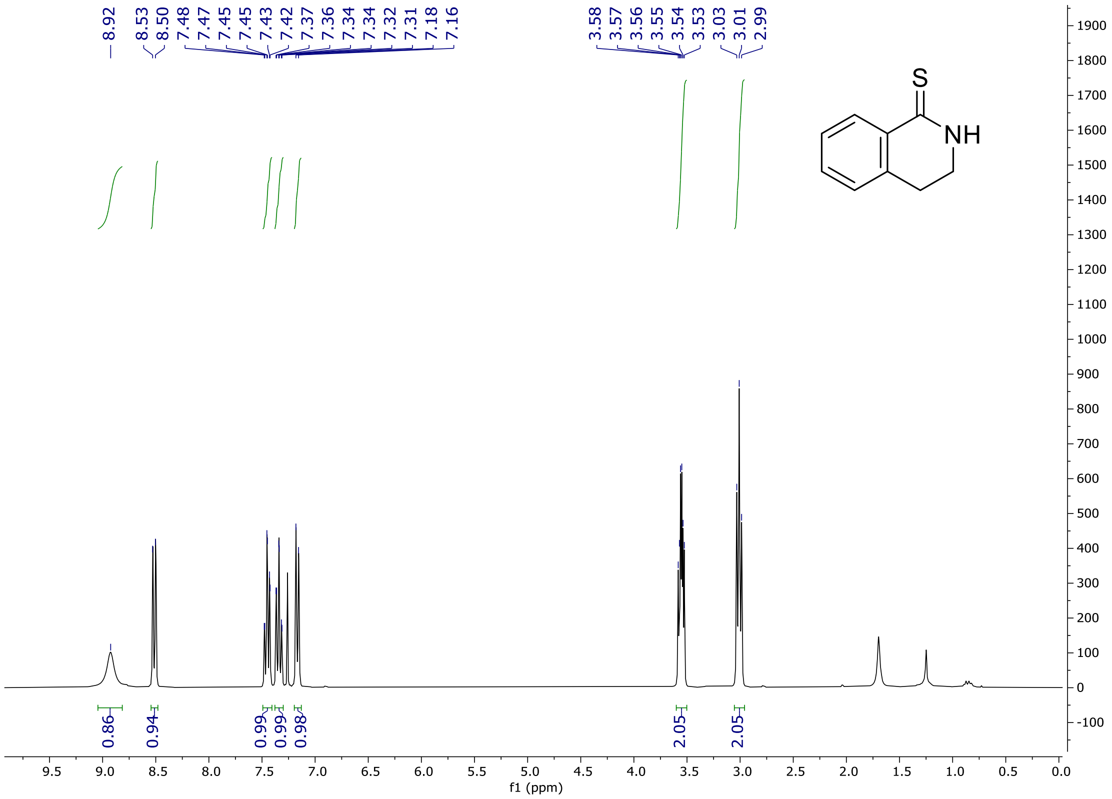

Figure SI_231: ^{1}H-NMR for **3af** in $CDCl_3$ (300 MHz).

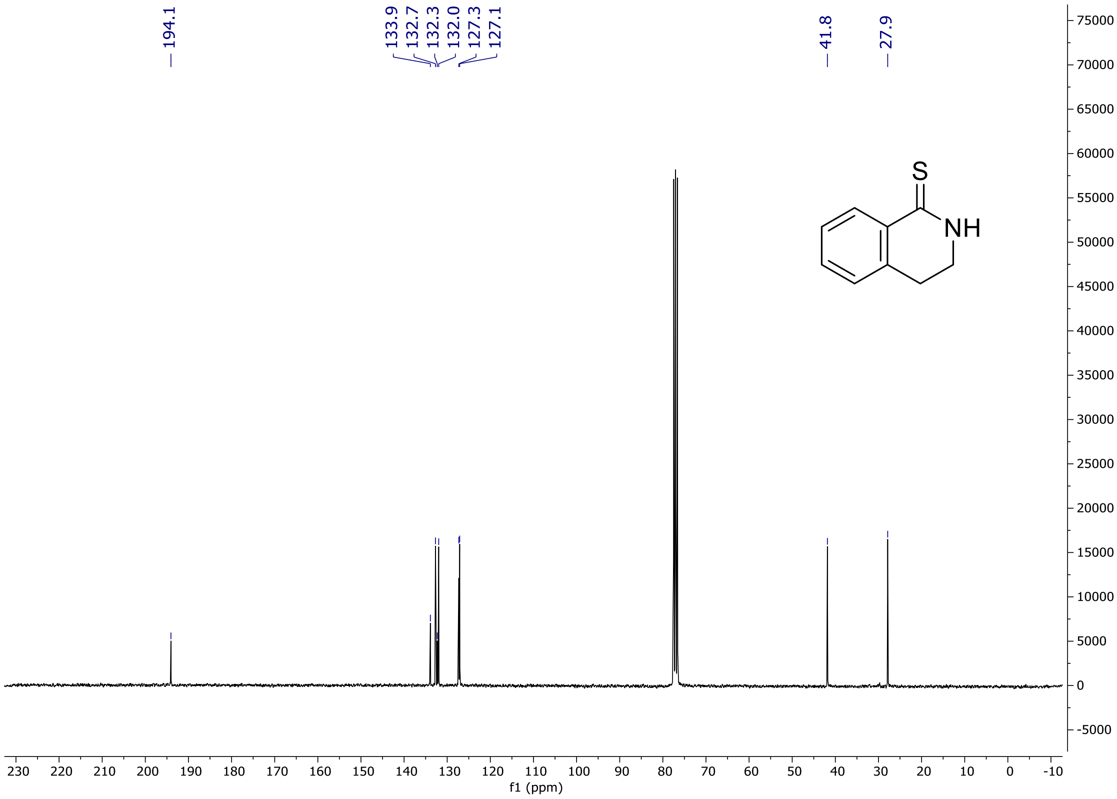

Figure SI_232: ^{13}C-NMR for **3af** in $CDCl_3$ (75 MHz).

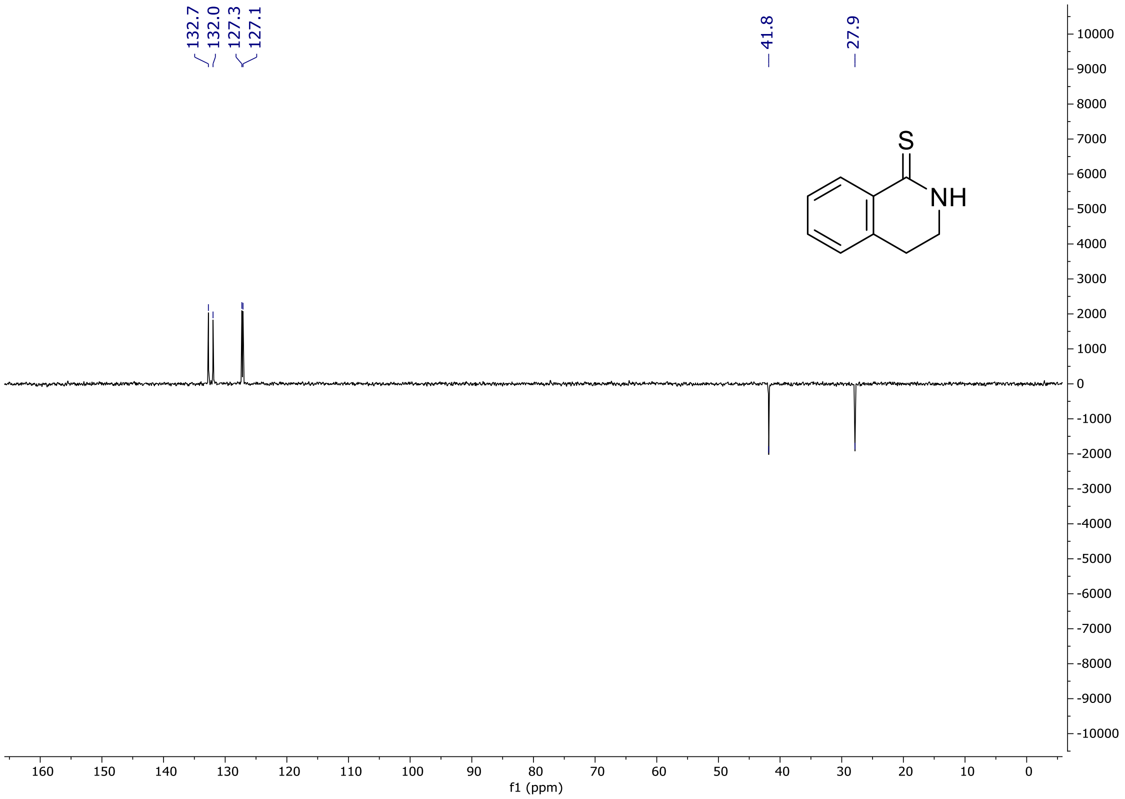

Figure SI_233: DEPT 135-NMR for **3af** in $CDCl_3$ (75 MHz).

Pyrrolidine-2-thione (3ag)

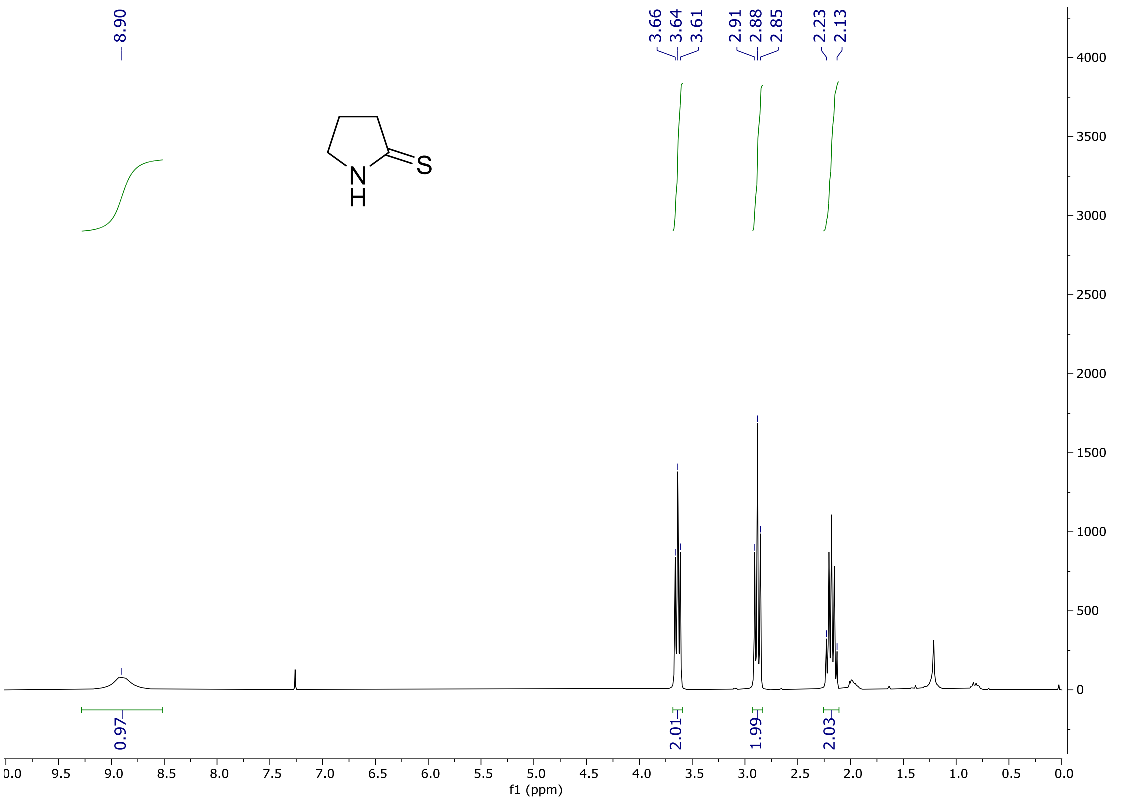

Figure SI_234: ^{1}H-NMR for **3ag** in $CDCl_3$ (300 MHz).

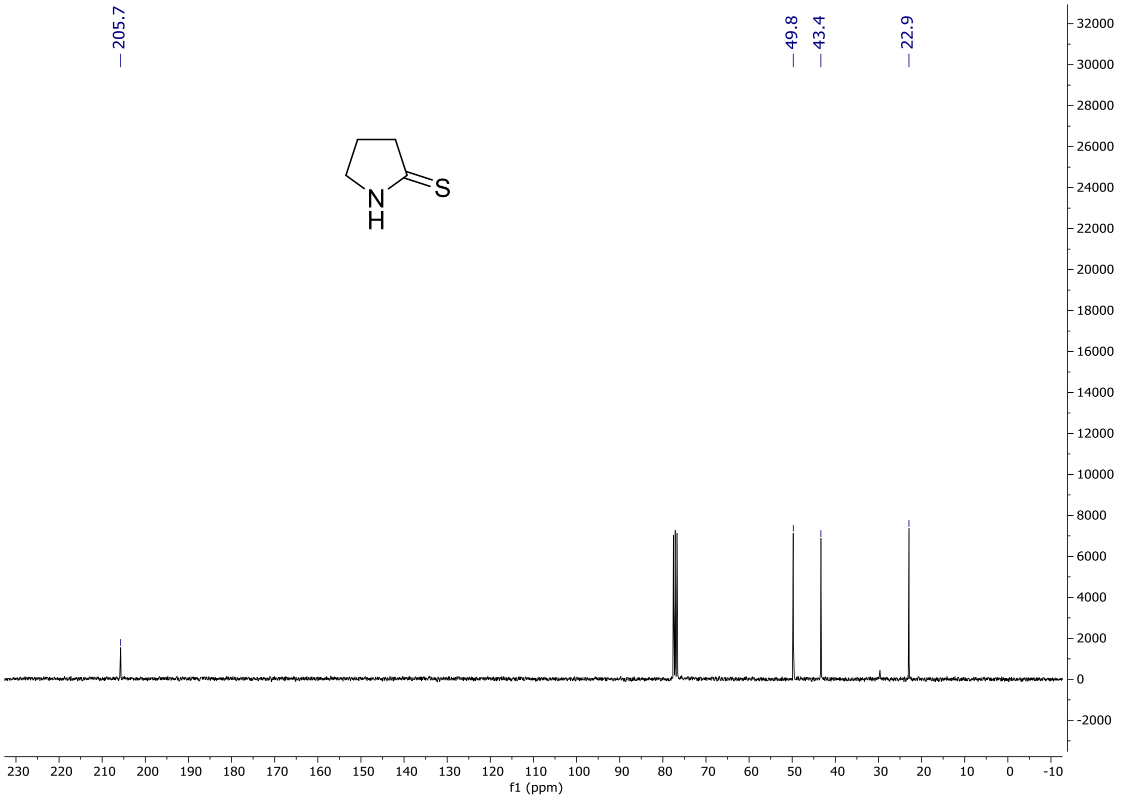

Figure SI_235: ^{13}C-NMR for **3ag** in $CDCl_3$ (75 MHz).

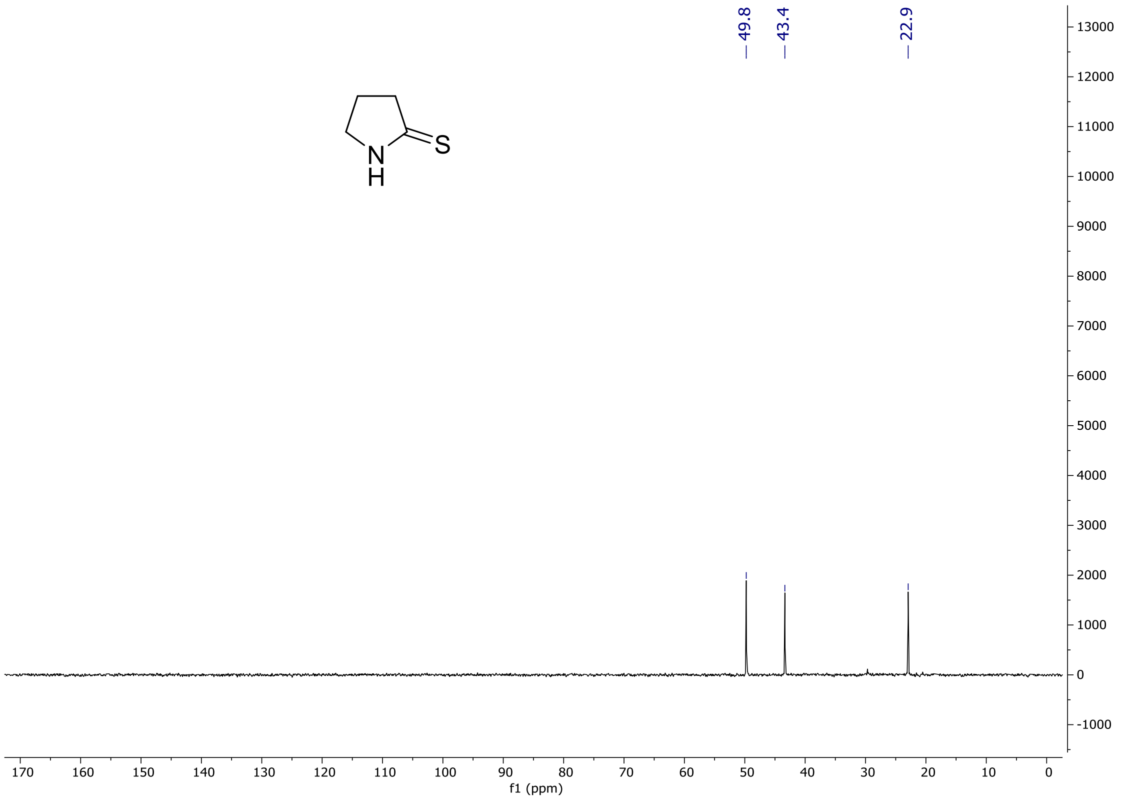

Figure SI_236: DEPT 135-NMR for **3ag** in $CDCl_3$ (75 MHz).

***N*-Ethylthioacetamide (3ah)**

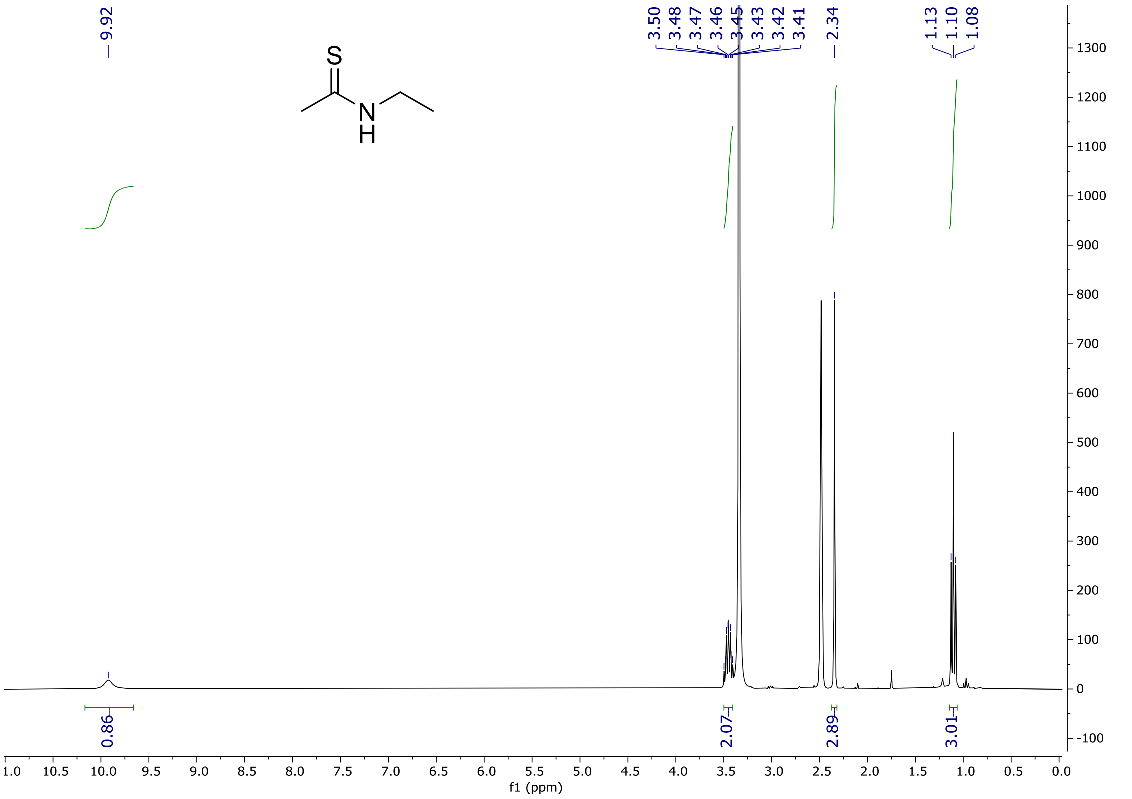

Figure SI_237: ^{1}H-NMR for **3ah** in DMSO-d^{6} (300 MHz).

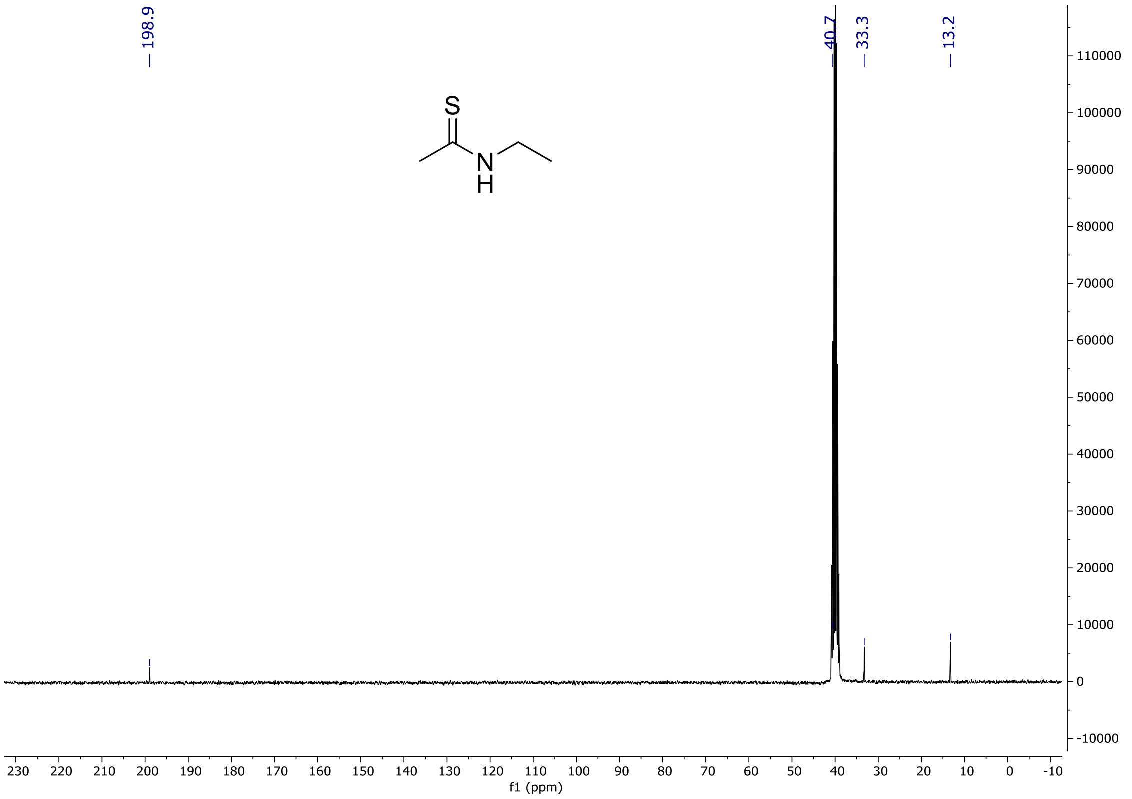

Figure SI_238: ^{13}C-NMR for **3ah** in DMSO-d^{6} (75 MHz).

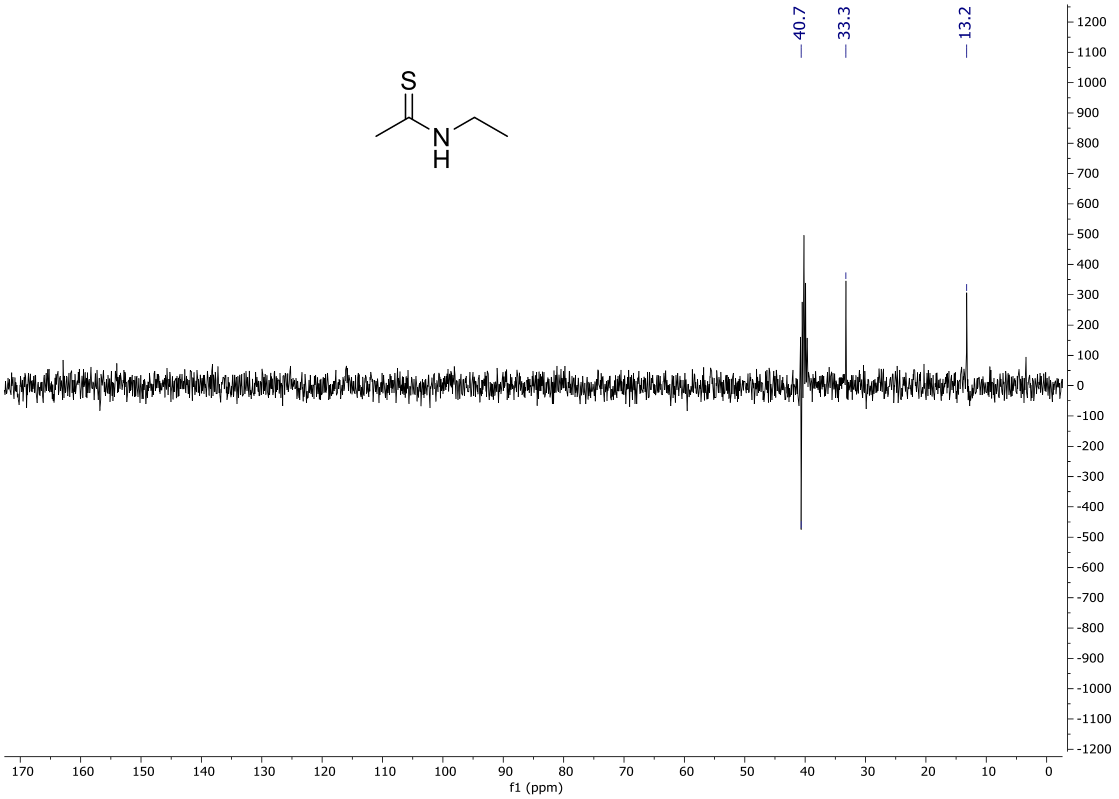

Figure SI_239: DEPT 135-NMR for **3ah** in DMSO-d^6 (75 MHz).

1-Chloro-2-((3-methylbut-2-en-1-yl)oxy)-4-nitrobenzene (5)

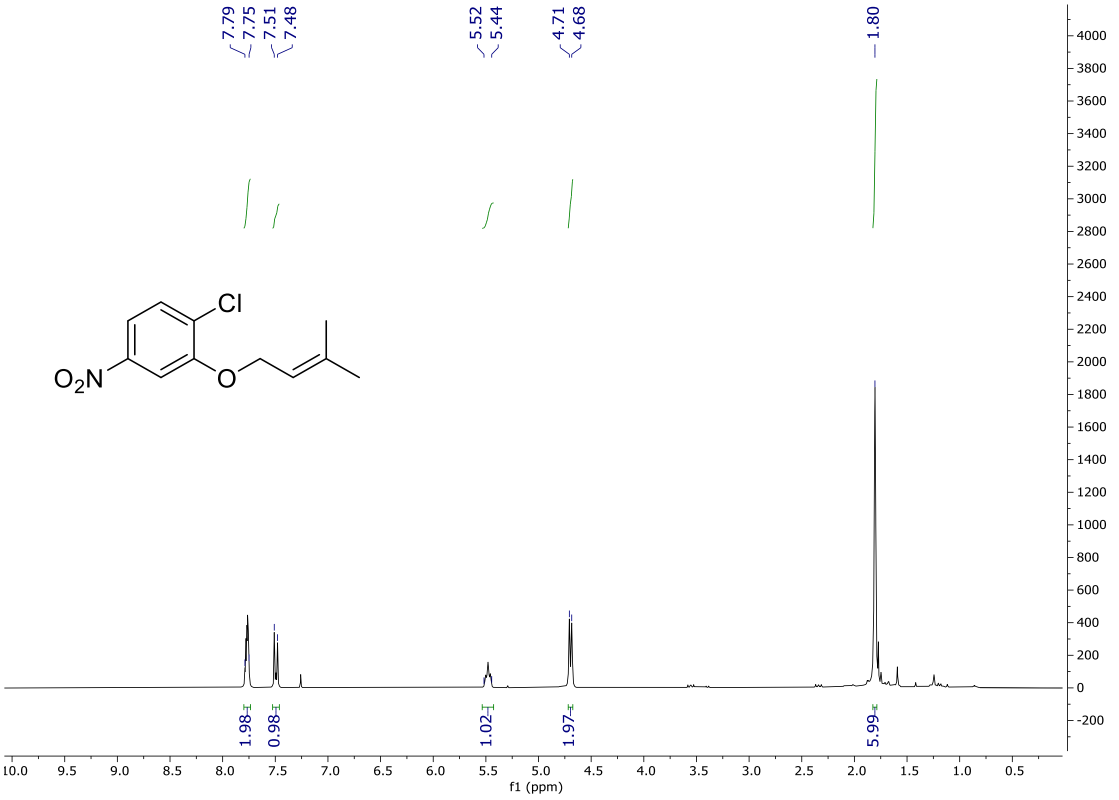

Figure SI_240: ^{1}H-NMR for **5** in $CDCl_3$ (300 MHz).

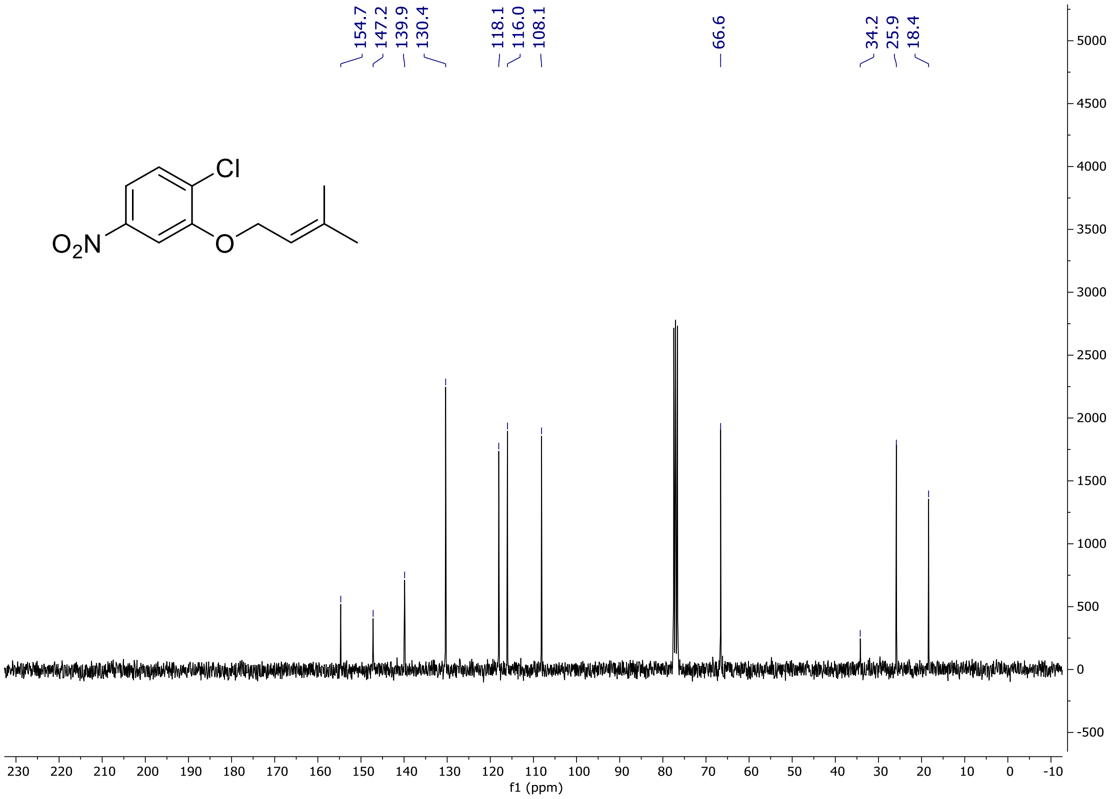

Figure SI_241: ^{13}C-NMR for **5** in $CDCl_3$ (75 MHz).

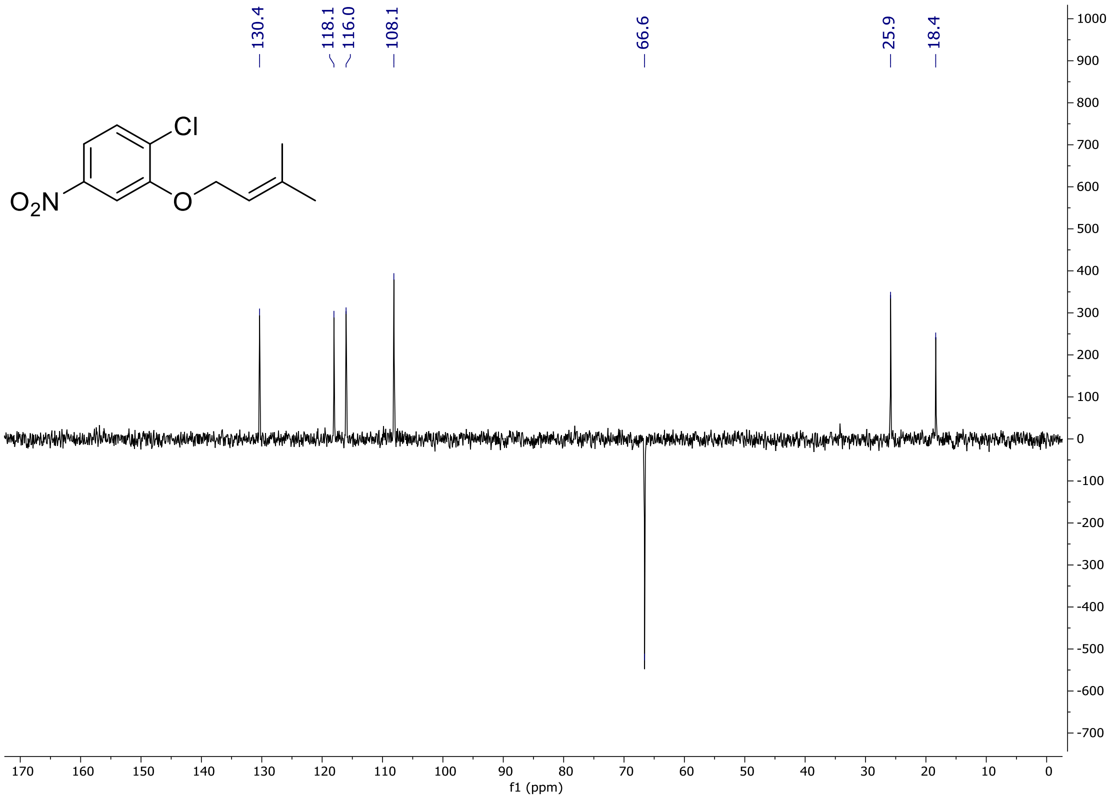

Figure SI_242: DEPT 135-NMR for **5** in $CDCl_3$ (75 MHz).

2-Methylfuran-3-carbaldehyde (7)

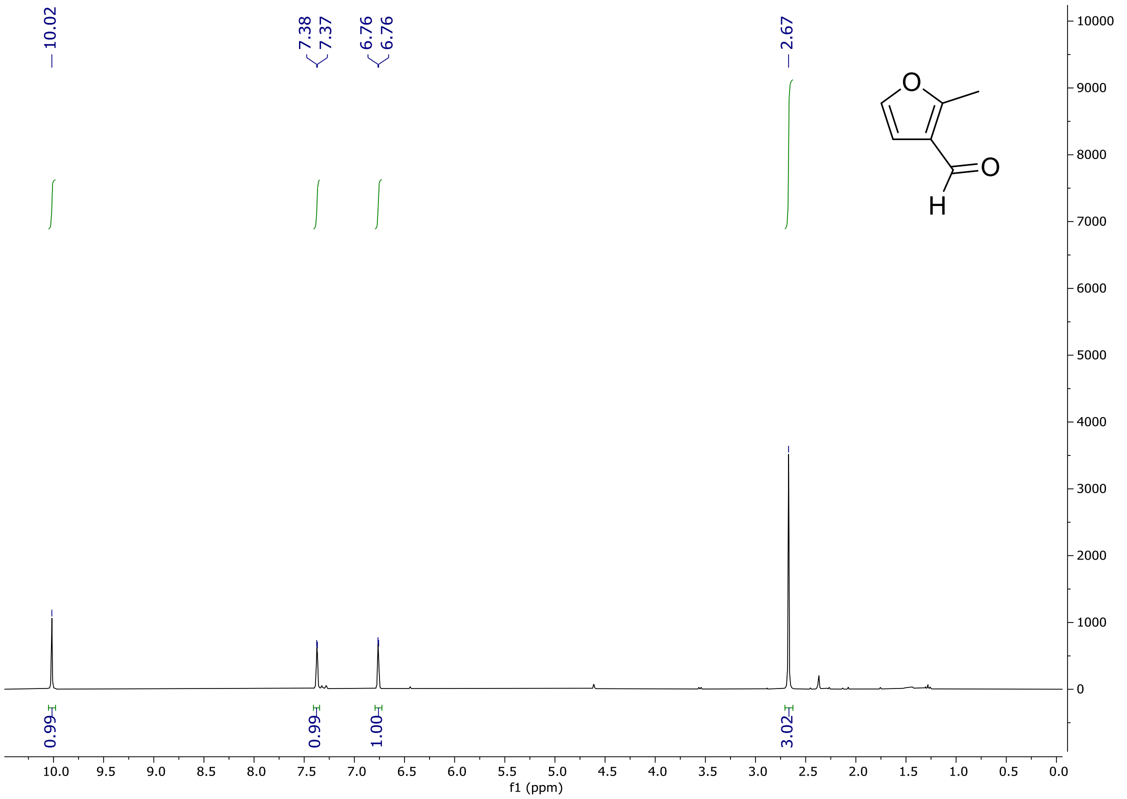

Figure SI_243: ^{1}H-NMR for **7** in $CDCl_3$ (300 MHz).

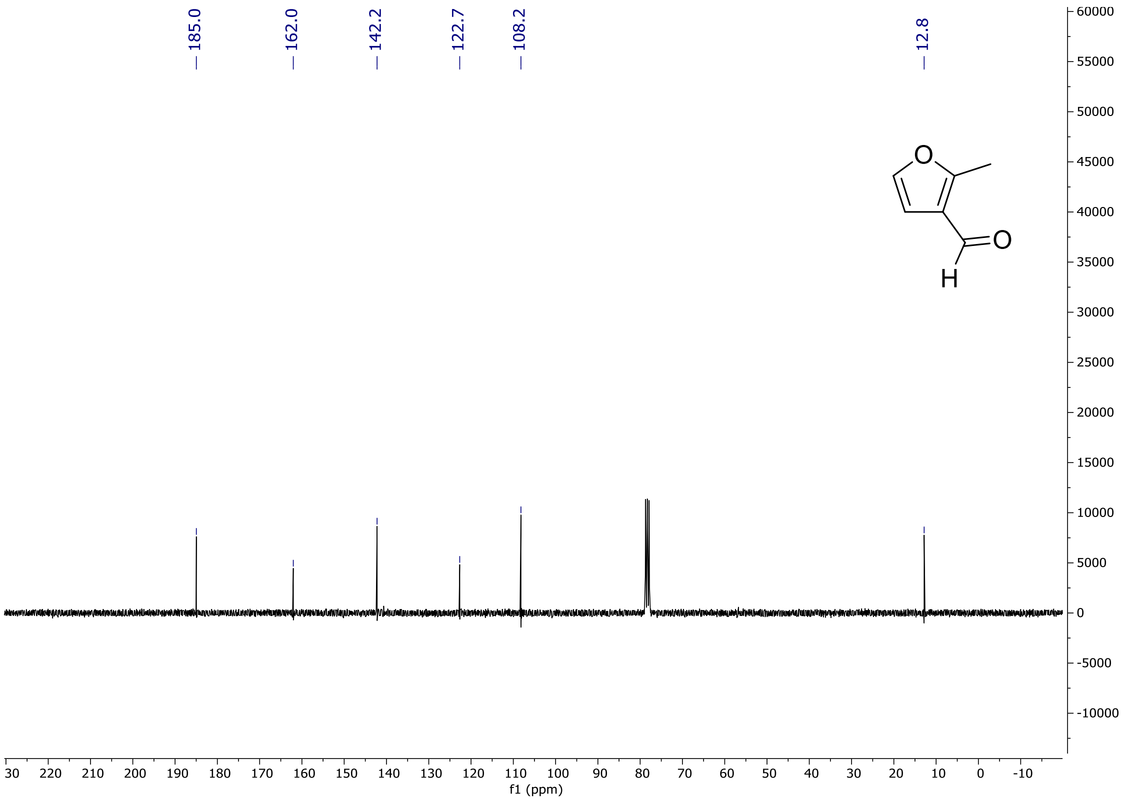

Figure SI_244: ^{13}C-NMR for **7** in $CDCl_3$ (75 MHz).

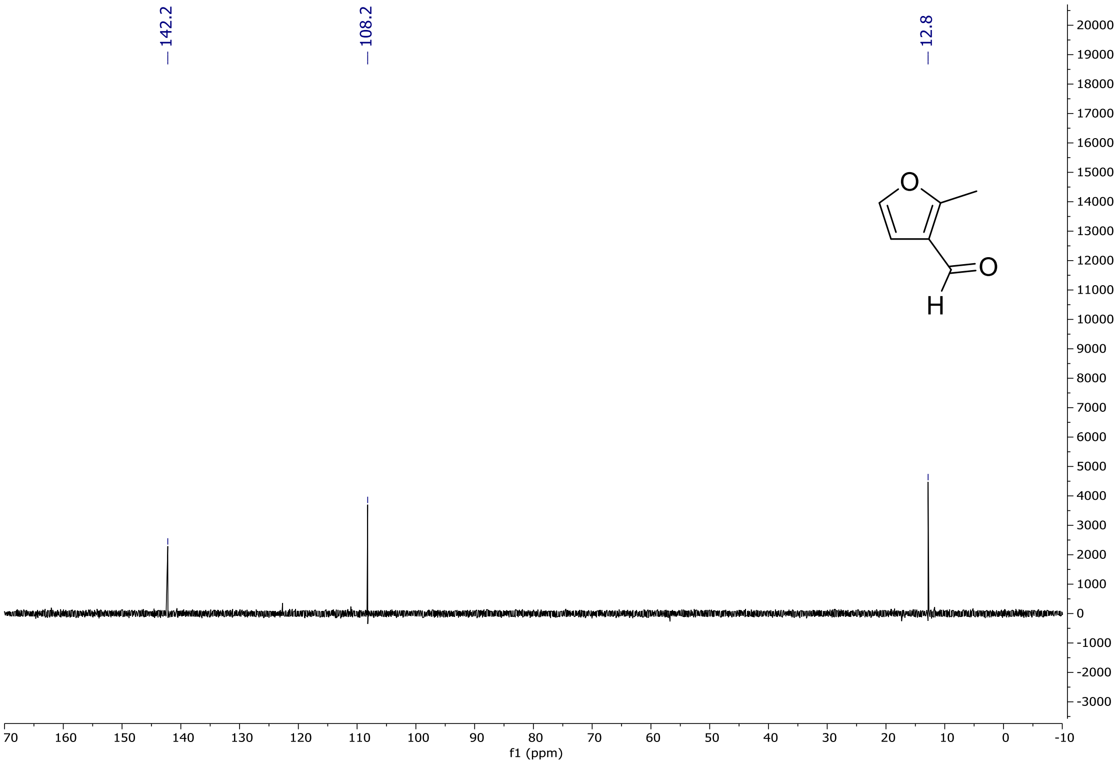

Figure SI_245: DEPT 135-NMR for **7** in $CDCl_3$ (75 MHz).

4-Chloro-3-((3-methylbut-2-en-1-yl)oxy)-*N*-(2-methylfuran-3-yl-methylidine)aniline oxide (1ai)

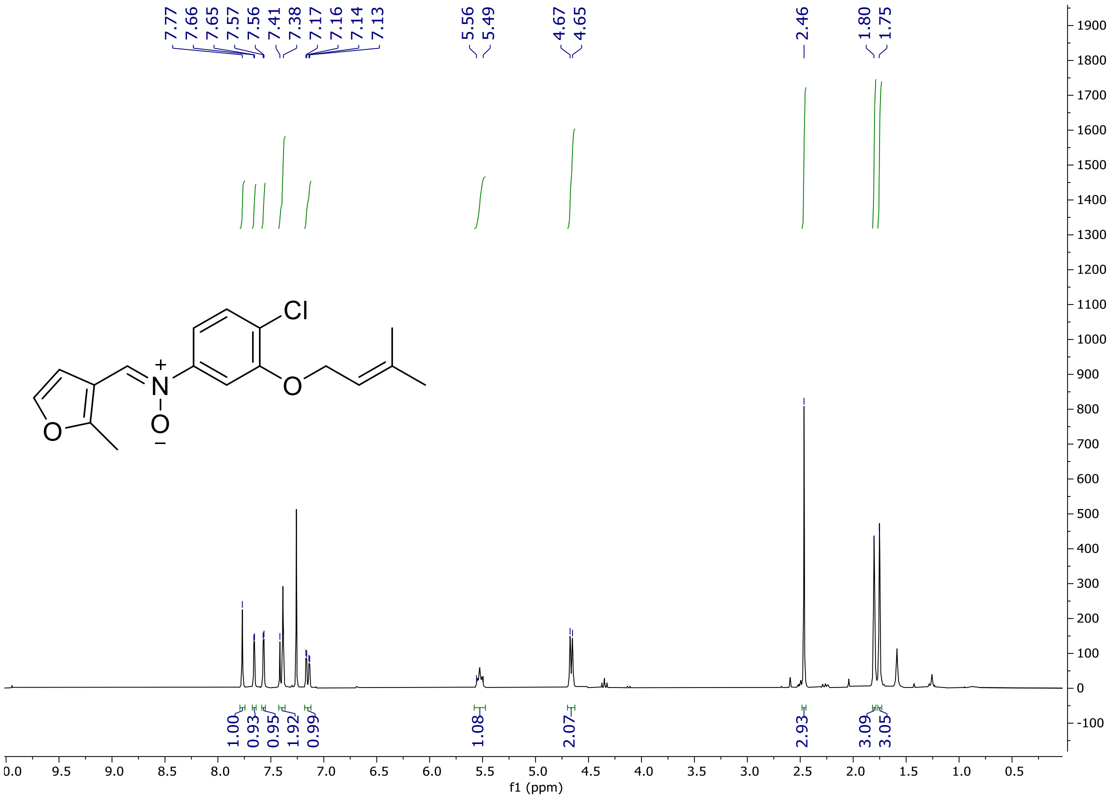

Figure SI_246: ¹H-NMR for **1ai** in $CDCl_3$ (300 MHz).

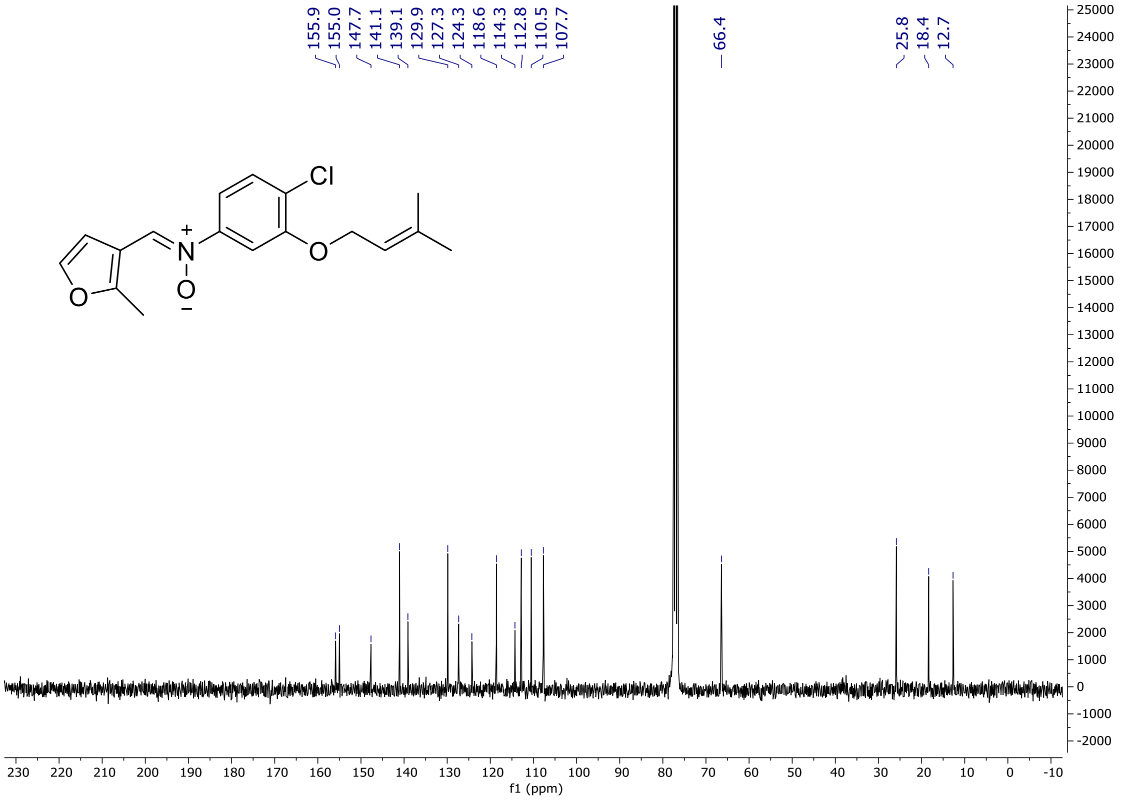

Figure SI_247: ^{13}C-NMR for **1ai** in $CDCl_3$ (75 MHz).

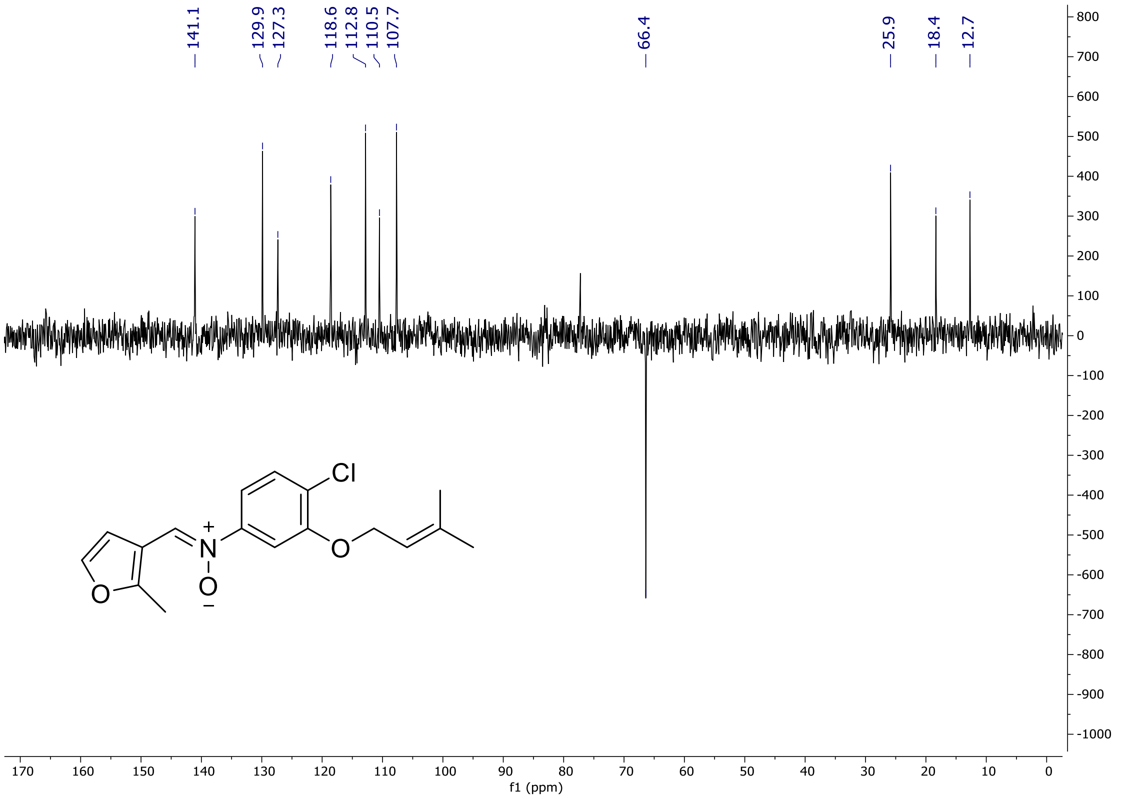

Figure SI_248: DEPT 135-NMR for **1ai** in $CDCl_3$ (75 MHz).

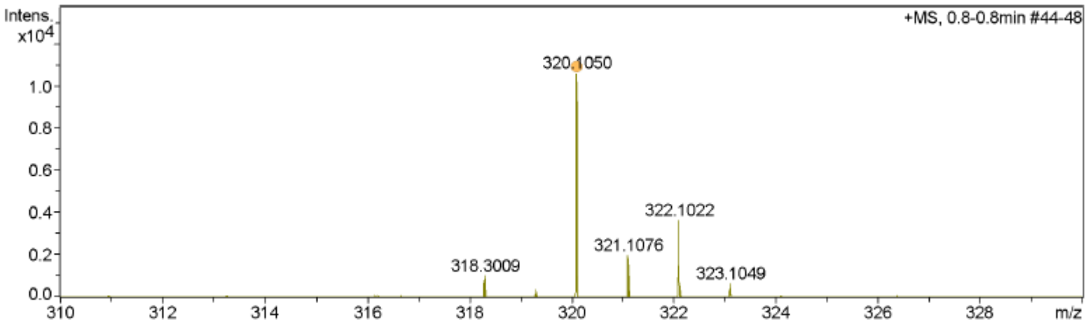

Meas. m/z	#	Ion Formula	m/z	err [ppm]	mSigma	# mSigma	Score	rdb	e⁻ Conf	N-Rule	Adduct
320.1050	1	C17H19ClNO3	320.1048	-0.5	3.6	1	100.00	9.0	even	ok	M+H

Figure SI_249: HRMS (ESI$^+$, m/z) analysis of **1ai**.

UC-781 (3ai)

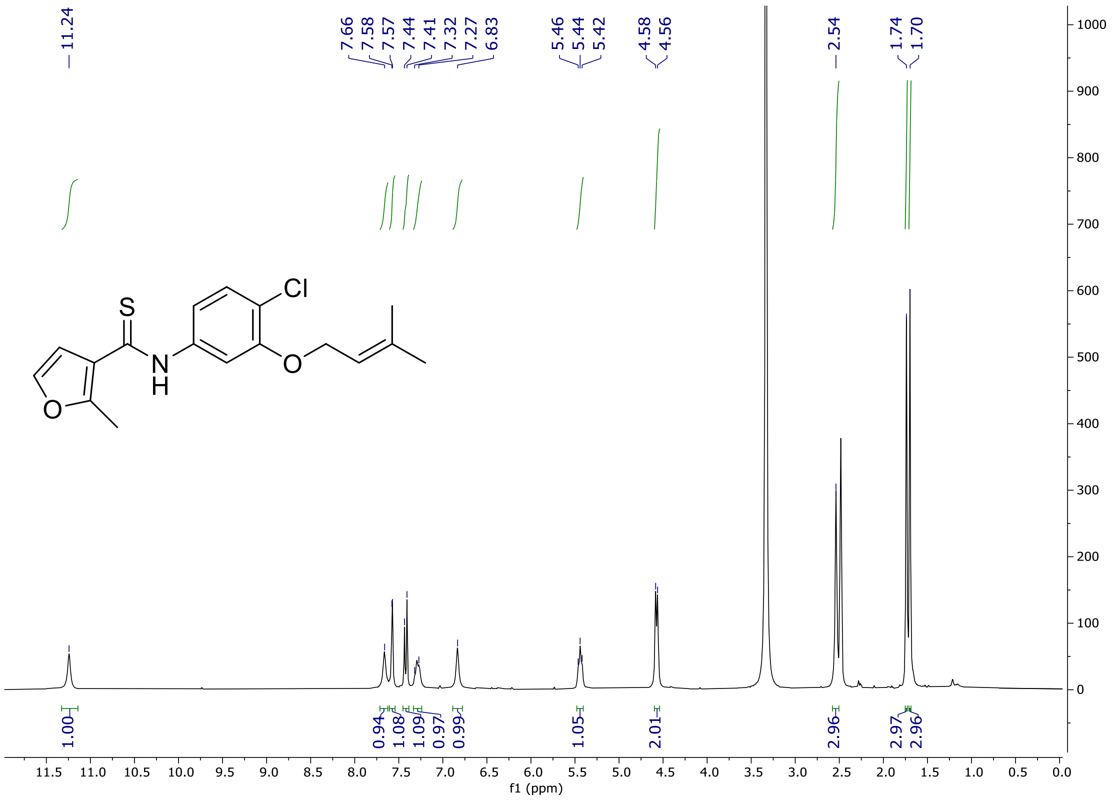

Figure SI_250: ^{1}H-NMR for **3ai** in DMSO-d^6 (300 MHz).

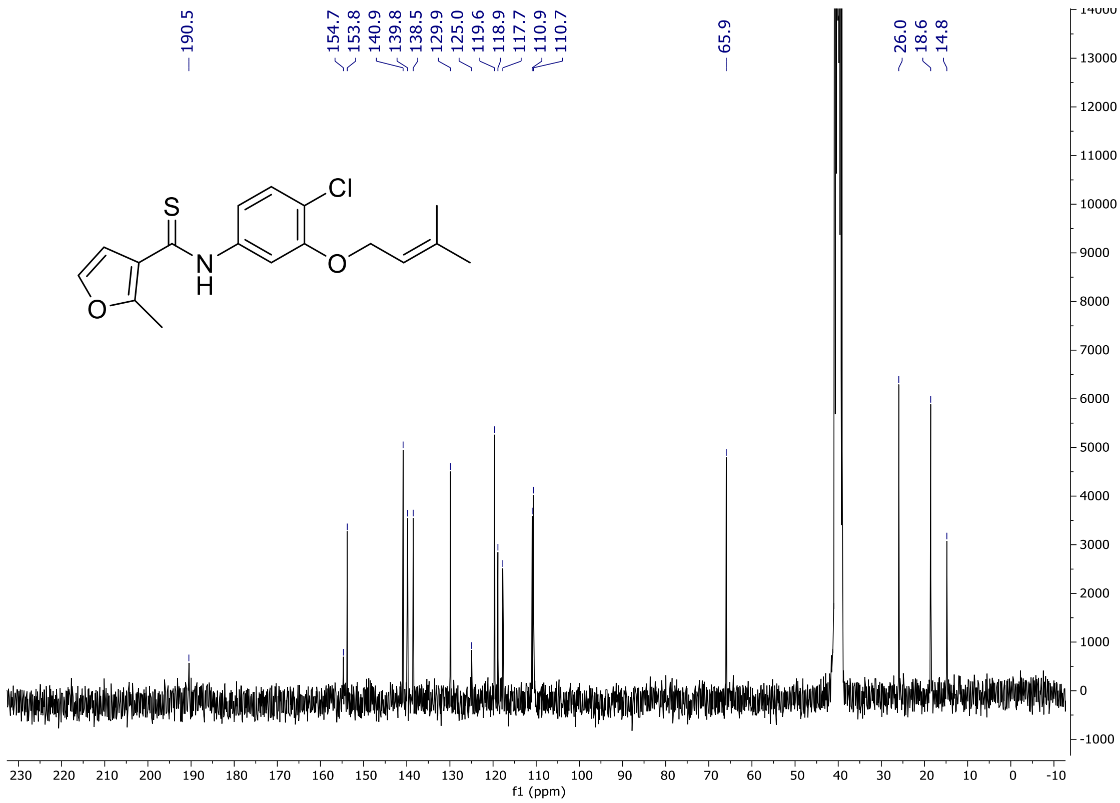

Figure SI_251: ^{13}C-NMR for **3ai** in DMSO-d^6 (75 MHz).

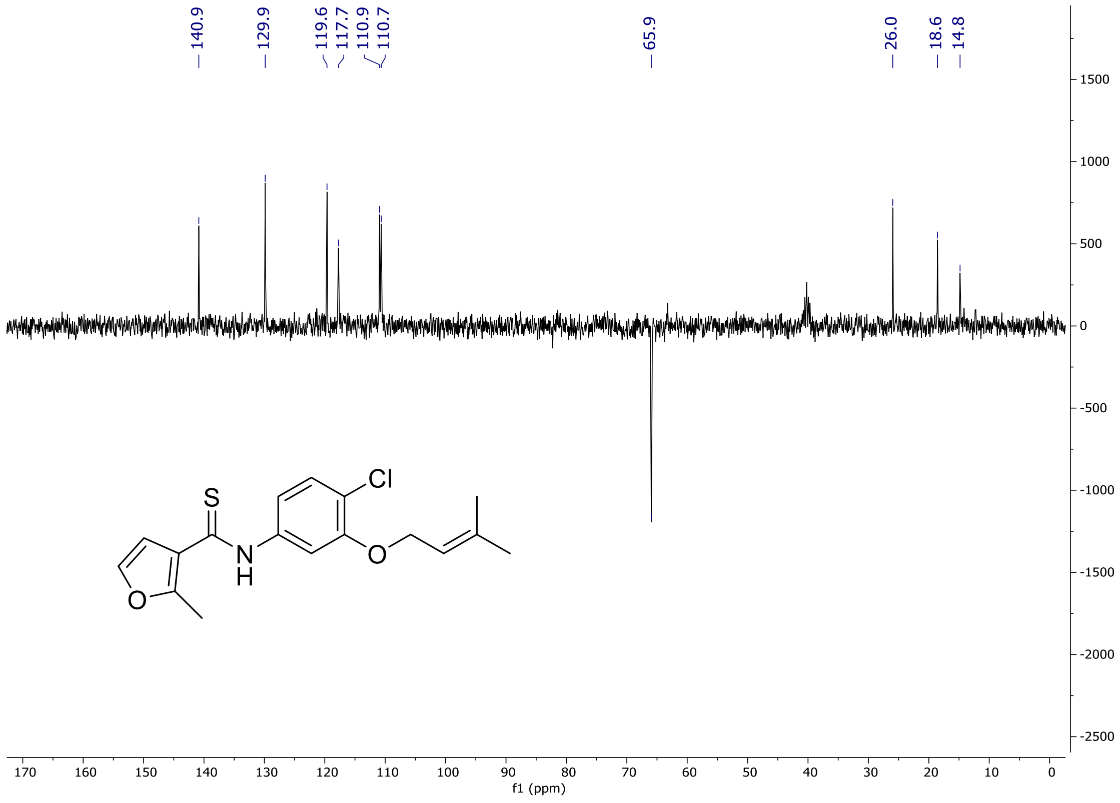

Figure SI_252: DEPT 135-NMR for **3ai** in DMSO-d^6 (75 MHz).

Acquisition Parameter

Source Type	ESI	Ion Polarity	Positive	Set Nebulizer	2.4 Bar
Focus	Active	Set Capillary	3500 V	Set Dry Heater	250 °C
Scan Begin	50 m/z	Set End Plate Offset	-500 V	Set Dry Gas	6.0 l/min
Scan End	1500 m/z	Set Charging Voltage	2000 V	Set Divert Valve	Source
		Set Corona	0 nA	Set APCI Heater	0 °C

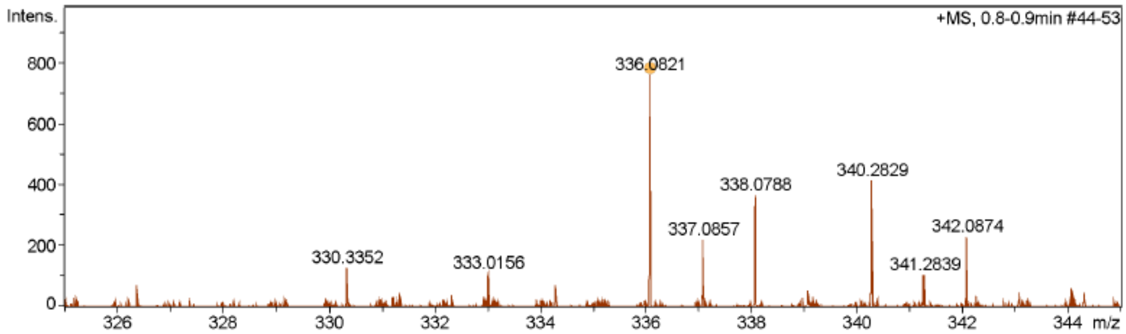

Meas. m/z	#	Ion Formula	m/z	err [ppm]	mSigma	# mSigma	Score	rdb	e¯ Conf	N-Rule
336.0821	1	C17H19ClNO2S	336.0820	-0.3	68.8	2	37.60	9.0	even	ok

Figure SI_253: HRMS (ESI$^+$, m/z) analysis of **3ai**.
